# Synthesis and Demonstration of the Biological Relevance of sp^3^‐rich Scaffolds Distantly Related to Natural Product Frameworks

**DOI:** 10.1002/chem.201704169

**Published:** 2017-10-06

**Authors:** Daniel J. Foley, Philip G. E. Craven, Patrick M. Collins, Richard G. Doveston, Anthony Aimon, Romain Talon, Ian Churcher, Frank von Delft, Stephen P. Marsden, Adam Nelson

**Affiliations:** ^1^ Astbury Centre for Structural Molecular Biology University of Leeds Leeds LS2 9JT UK; ^2^ School of Chemistry University of Leeds Leeds LS2 9JT UK; ^3^ Diamond Light Source Ltd Harwell Science and Innovation Campus Didcot OX11 0QX UK; ^4^ Structural Genomics Consortium, Nuffield Department of Medicine University of Oxford, Roosevelt Drive Oxford OX3 7DQ UK; ^5^ GlaxoSmithKline Medicines Research Centre Stevenage SG1 2NY UK,BenevolentBio, Churchway London NW1 1LW UK

**Keywords:** chemical biology, fragments, molecular diversity, natural products, proteins

## Abstract

The productive exploration of chemical space is an enduring challenge in chemical biology and medicinal chemistry. Natural products are biologically relevant, and their frameworks have facilitated chemical tool and drug discovery. A “top‐down” synthetic approach is described that enabled a range of complex bridged intermediates to be converted with high step efficiency into 26 diverse sp^3^‐rich scaffolds. The scaffolds have local natural product‐like features, but are only distantly related to specific natural product frameworks. To assess biological relevance, a set of 52 fragments was prepared, and screened by high‐throughput crystallography against three targets from two protein families (ATAD2, BRD1 and JMJD2D). In each case, 3D fragment hits were identified that would serve as distinctive starting points for ligand discovery. This demonstrates that frameworks that are distantly related to natural products can facilitate discovery of new biologically relevant regions within chemical space.

## Introduction

Small molecules continue to dominate Man's ability to treat disease, and can transform our understanding of fundamental biology. Yet historically, the exploration of chemical space has been highly uneven,^1^, ^2^ in part because a narrow toolkit of reliable reactions has underpinned molecular discovery.^3^ Natural products can facilitate the identification of biologically relevant chemical space^4^ since they have arisen through the function‐driven evolution of biosynthetic pathways.^5^ Indeed, around a third of recent small molecule drugs have been inspired by natural products.^6^ In biology‐oriented synthesis,^7^ natural product frameworks^4^ inform the design of productive small molecule screening collections and fragment sets that can be exploited in the discovery of ligands for unrelated protein targets.^8^, ^9^ In addition, synthetic approaches have been developed to convert specific natural products into alternative complex frameworks.^10^ Natural product‐inspired compounds can provide highly distinctive starting points for discovery that contrast starkly with most synthetic screening compounds:^11^ in particular, the typically high fraction of sp^3^‐hybridised carbons (Fsp^3^) is attractive since it correlates with the successful translation of clinical candidates.^12^

**Scheme 1:**
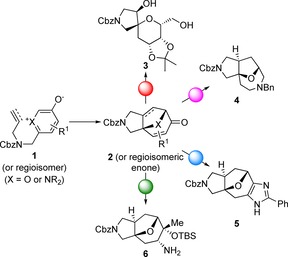
Overview of our unified approach to diverse natural product‐like scaffolds. Intramolecular [5+2] cycloaddition would yield alternative complex intermediates (e.g. **2**) that would be converted into diverse scaffolds by ring cleavage (red; for example, →**3**), ring expansion (magenta; for example, →**4**), annulation (blue; for example, →**5**) or addition/modification (green; for example, →**6**).

We envisaged a “top‐down” synthetic approach in which alternative complex, yet readily accessible, intermediates would be converted into many natural product‐like scaffolds (Scheme 1). Initially, bridged scaffolds of general structure **2** would be prepared using intramolecular [5+2] cycloaddition reactions^13^, ^14^ (e.g. **1**→**2**): ring cleavage (red; for example, **2**→**3**), ring expansion (magenta; for example, **2**→**4**), annulation (blue; for example, **2**→**5**), or functional group modification (green; for example, **2**→**6**) would then yield diverse scaffolds. The approach contrasts with diversity‐oriented strategies^15^, ^16^ in which building blocks are prepared (“built”) and linked (“coupled”) to yield intermediates which are then cyclised (“paired”) to yield alternative scaffolds. Although diversity‐oriented approaches to sp^3^‐rich fragments have been developed,^17^ their biological relevance has only rarely17b been demonstrated. To undertake a preliminary assessment of biological relevance, we have therefore prepared and screened a fragment set that is based on many of the scaffolds that are accessible using our synthetic approach.

## Results and Discussion

### Synthesis of natural product‐like scaffolds

**Scheme 2:**
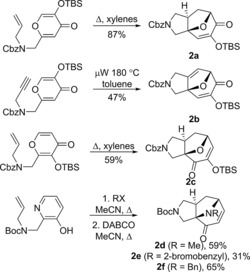
Synthesis of complex intermediate cycloadducts.

The bridged scaffolds **2 a**–**f** were prepared using intramolecular [5+2] cycloaddition chemistry (Scheme 2). The oxygen‐bridged cycloadducts were prepared by silyl transfer‐induced [5+2] cycloaddition of 3‐*tert*‐butyldimethylsilyloxy 4*H*‐pyran‐4‐ones (→**2 a**–**c**).^14^ In a similar vein, the nitrogen‐bridged cycloadducts were prepared by intramolecular cycloaddition of 3‐oxidopyridinium ylides (→**2 d**–**f**).^18^

**Scheme 3:**
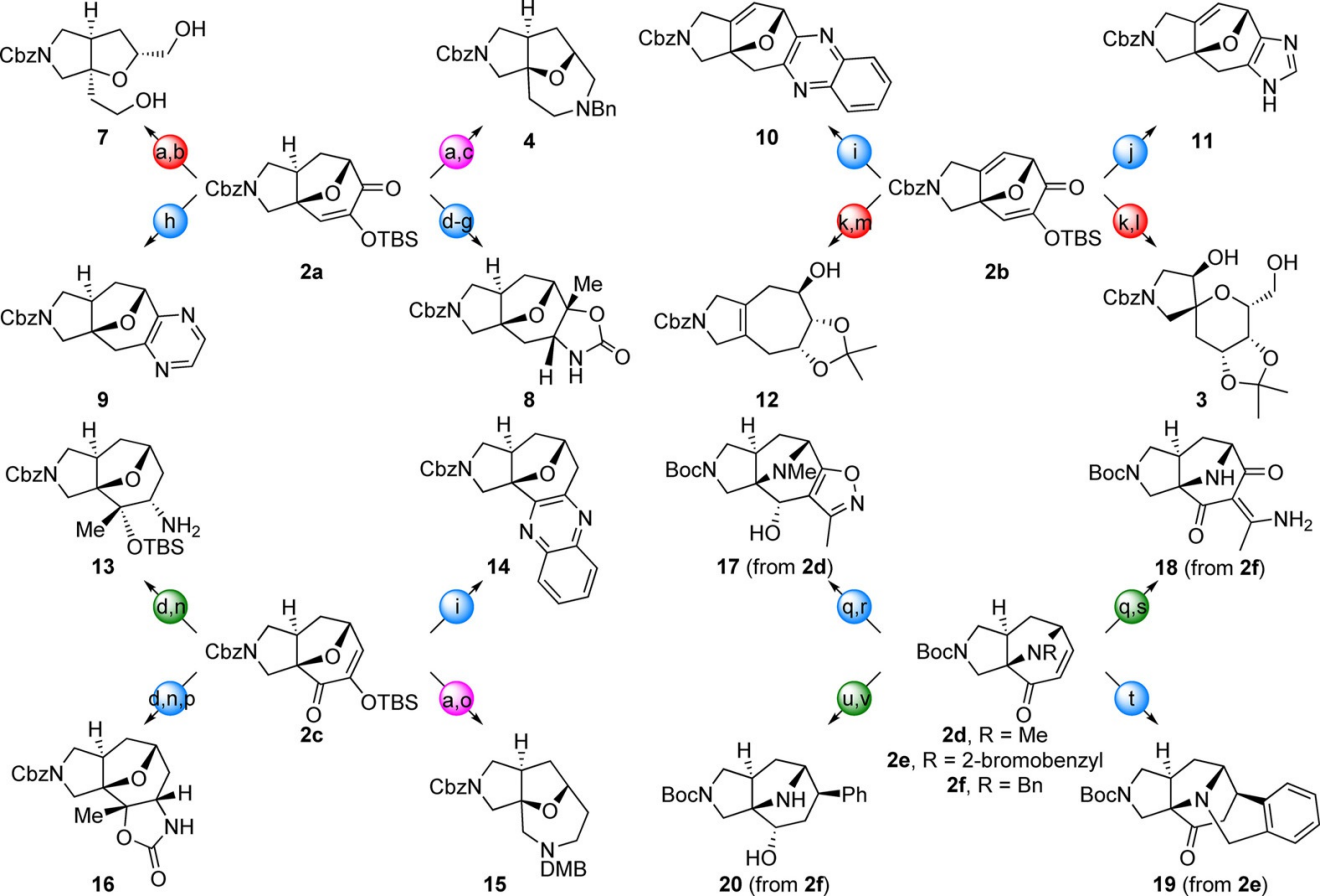
Representative syntheses of natural product‐like scaffolds. Scaffolds were prepared from cycloadducts by ring cleavage (red), ring expansion (magenta), ring formation (blue) or substitution (green). Typical conditions (see Supporting Information for full details): (a) NaBH_4_ then CSA (from **2 a**: 77 %; from **2 c**: 34 %); (b) NaIO_4_ then NaBH_4_, 44 %; (c) NaIO_4_ then BnNH_2_, NaBH(OAc)_3_, 32 %; (d) MeLi (from **2 a**: 91 %; from **2 c**: 53 %); (e) NH_3_, Ti(O*i*Pr)_4_, NaBH_4_, 77 %; (f) MeOCOCl, Et_3_N, 63 %; (g) TBAF then NaH, 29 %; (h) ethylene diamine, AcOH, μW, 180 °C, 40 %; (i) 1,2‐diaminobenzene, AcOH, heat (from **2 b**: 21 %; from **2 c**: 32 %); (j) NH_4_OAc, paraformaldehyde, 60 °C, 38 %; (k) NaBH_4_ then 2,2‐dimethoxypropane, *p*‐TsOH, 60 °C, 71 %; (l) O_3_ then Me_2_S then NaBH_4_, 44 %; (m) LiAlH_4_, THF, Δ, 75 %; (n) NH_3_, Ti(O*i*Pr)_4_, NaBH_4_, 22 %; (o) NaIO_4_ then DMBNH_2_, NaBH(OAc)_3_, 30 %; (p) MeOCOCl, Et_3_N then TBAF then NaH, 34 %; (q) EtNO_2_, PhNCO, NEt_3_ thenq DDQ, (from **2 d**: 37 %; from **2 f**: 26 %); (r) NaBH_4_, CeCl_3_⋅7 H_2_O, −78 °C, 87 %; (s) H_2_, Pd(OH)_2_/C, HCl, 89 %; (t) 20 mol % Pd(OAc)_2_, 40 mol % PPh_3_, NEt_3_, 11 %; (u) PhB(OH)_2_, NEt_3_, 1 mol % [Rh(cod)Cl)]_2_ then H_2_, Pd(OH)_2_/C, HCl, 46 %; (v) NaBH_4_, 19 % (plus 19 % epimer).

The complex bridged intermediates **2** were transformed into diverse molecular scaffolds (Scheme 3 and Supporting Information). Cleavage of specific bonds enabled scaffold simplification. Specifically, we exploited 1,2‐diol cleavage (e.g.→the fused bicyclic scaffold **7**); alkene ozonolysis (e.g.→the spiro‐fused scaffold **3**; and reductive allylic ether cleavage (→the fused bicyclic scaffold **12**). Interception of dialdehydes formed by 1,2‐diol cleavage enabled overall ring expansion. Thus, cleavage of the regioisomeric 1,2‐diols formed from **2 a** and **2 c**, followed by cyclative double reductive amination, yielded the tricyclic scaffolds **4** and **15**, respectively.

Annulation enabled more complex scaffolds to be prepared. For example, fusion of alternative heterocycles was possible either by condensation–aromatisation of the masked diketone of **2 a**, **2 b** or **2 c** (e.g. → the pyrazine **9**, the quinoxalines **10** and **14** or the imidazole **11**); or regioselective [3+2] annulation to the enone **2 d** (→the pyrrole **17**). Alternatively, treatment of the regioisomeric intermediates **2 a** and **2 c** with MeLi effected 1,2‐addition; reductive amination and oxazolidinone formation then gave the regioisomeric tetracycles **8** and **16**. Furthermore, intramolecular reductive Heck reaction^19^ of **2 e** gave the complex tetracycle **19**. Finally, substituted analogues were prepared in which the framework of the initial cycloadduct had been retained (e.g. **13**). Overall, the 26 scaffolds^20^ were prepared from commercially available starting materials in a total of just 64 steps (processes conducted in a single reaction vessel).

**Figure 1:**
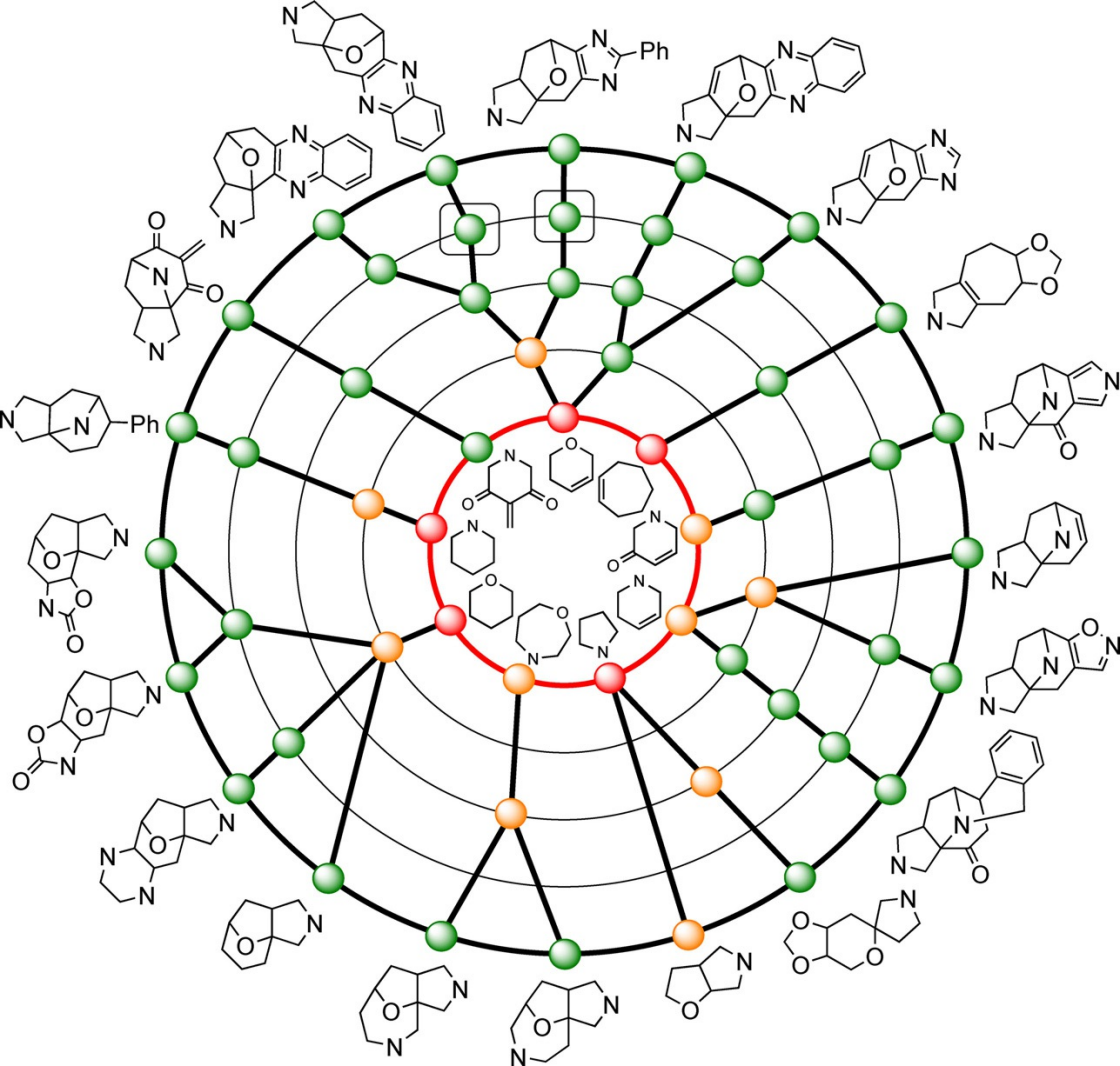
Hierarchical scaffold tree. The circles represent frameworks at the graph‐node‐bond level (22 frameworks represented in the 26 scaffolds prepared, outer ring and boxed; simplified frameworks, other circles). The 22 frameworks are related to nine parent (monocyclic) frameworks (identified using an established protocol, ref. 21). At each level of hierarchy, occurrence as substructures of natural products is indicated (green, not found; orange, found in <1 % of natural products; red, found in >1 % of natural products).

An hierarchical tree was constructed to capture the relationship between the scaffolds (Figure 1). Here, scaffolds were systematically simplified using an established protocol by removal of rings until a parent monocyclic ring system was ultimately obtained.^21^ Twenty two different graph‐node‐bond frameworks (capturing atom and bond type) were represented, which were then simplified to give nine parent monocycles. The scaffolds are based on a wide range of parent ring systems and there is thus significant diversity at each level of hierarchy of the scaffold tree. The exploitation of several different complex intermediates **2** was critical to the diversity that was possible, for example by enabling variation of regiochemistry (e.g. **4**/**15**, **8**/**16** and **10**/**14**) and heteroatom position and identity (e.g. **11**/**17**). Such an approach would unlikely be possible by modification of natural products, since several related starting materials would be required in multi‐gram quantities.

**Figure 2:**
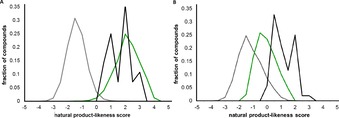
Natural product likeness of scaffolds and fragments. Panel A: Natural product‐likeness scores for the 26 scaffolds (black), 4,460 natural products (green) and a commercial screening collection (278,365 largely synthetic compounds, grey). Panel B: Natural product likeness scores for the 52 fragments prepared (black), 1,236 commercially‐available fragments (grey) and 128 natural product‐inspired fragments (green). Compounds are binned into 0.5 unit bins.

To compare with other screening sets, we determined natural product likeness scores^11^ for the deprotected scaffolds, a natural product screening library (4,460 compounds) and a commercial screening collection (278,365 largely synthetic compounds) (Figure 2, Panel A). The distribution of the scores for the scaffolds was broadly similar to that of the natural product screening library but highly distinctive from that of the large screening collection. The local structural features of our scaffolds are thus reminiscent of those found in natural products. Despite the high natural product likeness, however, only one of the 22 graph‐node‐bond frameworks is actually a substructure in the roughly 281,000 compounds in the Dictionary of Natural Products^22^ (Figure 1 and Supporting Information). Indeed, significant simplification to mono‐ or bicyclic frameworks is required before sub‐structures of natural products are found. Our scaffolds thus have high natural product likeness, but their frameworks lie in branches that augment the scaffold trees of natural product frameworks.

### Synthesis of a fragment set and high‐throughput crystallographic screens

To enable preliminary assessment of biological relevance, we prepared 52 racemic fragments based on 23 of the scaffolds. Here, a fragment‐based approach was exploited to enable efficient exploration of chemical space accessible using our synthetic approach. The set was designed to have high shape diversity, and to comprise fragments with controlled^23^ size (13 to 19 heavy atoms) and lipophilicity (−1.5<*c*log*P*<3) (Supporting Information). The fragment set was significantly more three‐dimensional (Supporting Information),^24^ and more natural product‐like (Figure 2, Panel B), than commercially available fragments with the same heavy atom range.

The fragment set was screened against three protein targets from two different mechanistic classes involved in epigenetic biology: the ATAD2 and BRD1 (also known as BRPF2) bromodomains, and the histone demethylase JMJD2D (also known as KDM4D). Here, the objective was to perform a preliminary assessment of biological relevance rather than to provide specific starting points for discovery. High expression levels of ATAD2^25^ and JMJD2 family members^26^ correlate with poor outcomes in several cancers, whilst BRD1 is a member of the BRPF family of scaffolding proteins whose role in acute myeloid leukemia is now emerging.^27^ The two bromodomains are contrasting targets: ATAD2 has a shallow *N*‐acetyl lysine binding site and has been suggested to have particularly low druggability.^28^

**Figure 3:**
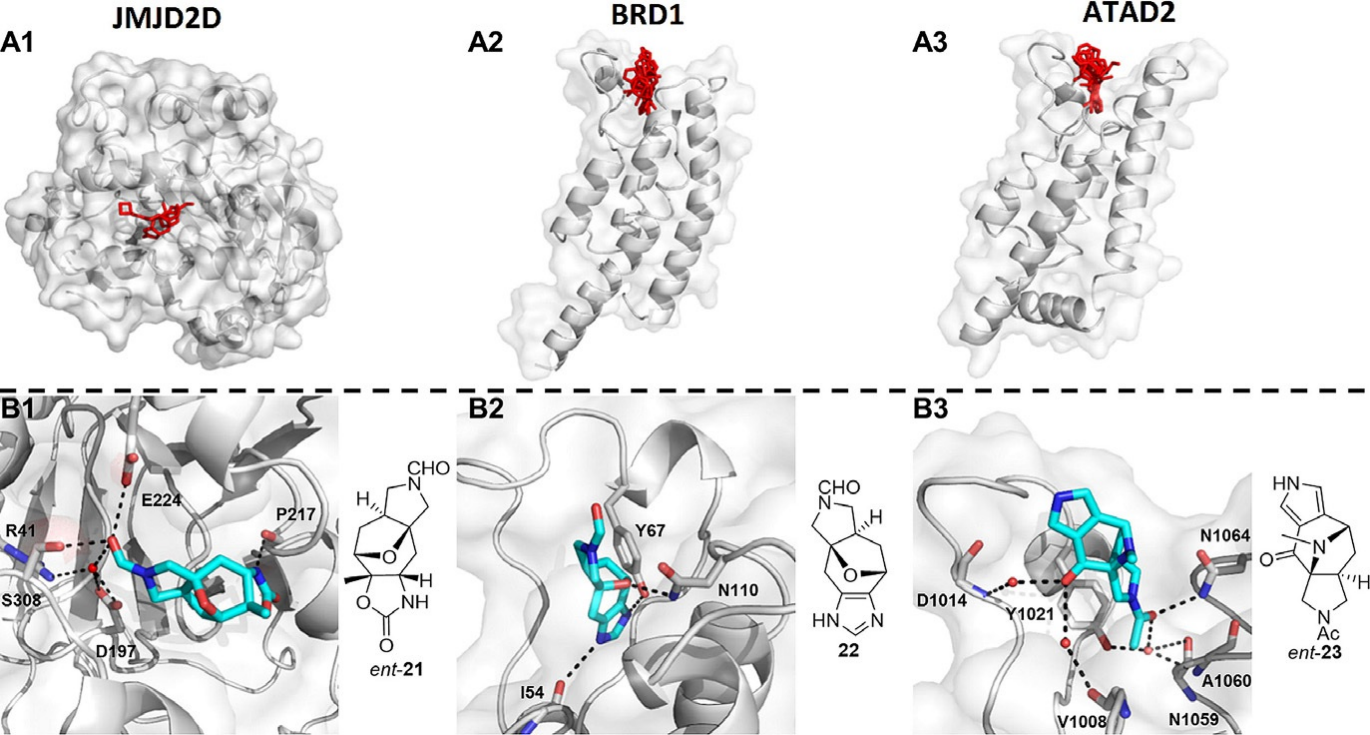
Fragment hits for three epigenetic targets. Panel A: Overview of fragment hit binding sites in JMJD2D (A1), the BRD1 bromodomain (A2) and the ATAD2 bromodomain (A3); the active site of JMJD2D is buried. Panels B: Interaction of exemplar fragment hits with JMJD2D (B1), BRD1 (B2) and ATAD2 (B3).

The three target proteins were all amenable to high‐throughput protein crystallography. Protein crystals were soaked with the 52 individual racemic fragments,^29^ picked and then subjected to automated X‐ray diffraction. Fragment hits were identified through detection of additional electron density,^30^ and inspection for polar interactions with the protein. Fragment hits were successfully identified for each of the target proteins: two hits for JMJD2D, eight hits for the BRD1 bromodomain and seven hits for the ATAD2 bromodomain (Figure 3 and Supporting Information).

The fragment screen against JMJD2D revealed two hits, both of which targeted a peripheral binding site (Panel B1, Figure 3 and Supporting Information). The hits complement those found in previous fragment screens against JMJD2D:^26^, ^31^ specifically, X‐ray crystallography had revealed 23 fragments all target the enzyme active site. The functional importance of this peripheral binding site could now be investigated, for example to reveal opportunities for allosteric modulation of the enzyme.

For the BRD1 bromodomain, the eight fragment hits targeted the *N*‐acetyl lysine binding site (Panel B2, Figure 3 and Supporting Information; see Ref. [27] and references therein for known ligands). The fragment hits contained several different *N*‐acetyl mimetics that interacted with both N110 and, either directly or via a bridging water, Y67.

**Figure 4:**
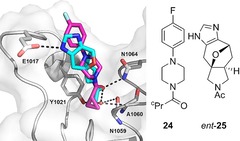
Exemplar fragment hits from high‐throughput crystallographic screens of conventional (hit: **24**; magenta) and our natural product‐like (hit: *ent*‐**25**; cyan) fragment sets against the ATAD2 bromodomain.

For the ATAD2 bromodomain, the fragment screen yielded seven hits (based on six distinct frameworks) that targeted the *N*‐acetyl lysine binding site (Panel B3, Figure 3 and Supporting Information). Six of the hits mirrored the binding mode of the *N*‐acetyl lysine side chain,28a interacting directly with N1064 and, via a bridging water, with Y1021. Four hits were in common with BRD1 although, in three cases, the interaction networks were more extensive: for example *ent*‐**23** made water‐mediated interactions with V1008 and D1014 (Panel B3, top, Figure 3) and *ent*‐**25** interacted directly with E1017 (Figure 4).

To compare directly with a more conventional fragment set, we also screened 700 commercially available fragments against ATAD2 by high‐throughput crystallography and identified nine hits that targeted *N*‐acetyl lysine binding site (Figure 4 and Supporting Information). As a group, the interactions of these fragments parallel those of other sp^2^‐rich fragments: they interact directly with N1064 and/or, via a bridging water, with Y1021 but make few additional polar contacts.^25^, ^28^, ^32^ For screens against many targets, flatter fragments have been observed to have higher hit rates.^33^ With ATAD2, however, a significantly higher hit rate was observed with our shape‐diverse natural product‐like fragments (7/52) than with more conventional^34^ flatter fragment sets (9/700). This outcome is consistent with the low hit rate observed in a previous fragment screen by NMR (65/13800, subsequently triaged to yield 12 hits with *K*_d_<1 mm).28b

## Conclusion

We have developed a “top‐down” synthetic approach in which alternative complex, yet readily accessible, intermediates were converted into many diverse scaffolds. These scaffolds have local natural product‐like features, but are only distantly related to specific natural product frameworks. A set of 52 fragments based on 23 of the scaffolds was screened against three epigenetic targets from two distinct protein families. In each case, hits were obtained that may provide distinctive opportunities for subsequent fragment growth. We have therefore demonstrated that frameworks that are distantly related to natural products can facilitate identification of novel regions of biologically relevant chemical space. Synthetic approaches to such frameworks may thus help identify fertile chemical space for bioactive small molecule discovery that is inaccessible to existing compound collections and biosynthetic pathways.

## Conflict of interest

The authors declare no conflict of interest.

## Supporting information

As a service to our authors and readers, this journal provides supporting information supplied by the authors. Such materials are peer reviewed and may be re‐organized for online delivery, but are not copy‐edited or typeset. Technical support issues arising from supporting information (other than missing files) should be addressed to the authors.

Supplementary
