# Supplementary material for: Synthesis and Demonstration of the Biological Relevance of sp3‐rich Scaffolds Distantly Related to Natural Product Frameworks
Source: Chemistry. 2017 Oct 6;23(60):15227–32. doi: 10.1002/chem.201704169 (PMC5703167; doi:10.1002/chem.201704169)
Supplement: Supplementary file 1 — Supplementary [file CHEM-23-15227-s001.pdf]

CHEMISTRY
A European Journal

Supporting Information

Synthesis and Demonstration of the Biological Relevance of sp^3-rich Scaffolds Distantly Related to Natural Product Frameworks

Daniel J. Foley,[a, b] Philip G. E. Craven,[a, b] Patrick M. Collins,[c] Richard G. Doveston,[a, b] Anthony Aimon,[a, b] Romain Talon,[d] Ian Churcher,[e] Frank von Delft,*[c, d] Stephen P. Marsden,*[b] and Adam Nelson*[a, b]

chem_201704169_sm_miscellaneous_information.pdf

Synthesis and Demonstration of the Biological Relevance of sp^3-rich Scaffolds Distantly Related to Natural Product Frameworks

Dr Daniel J. Foley, Dr Philip G. E. Craven, Dr Patrick Collins,
Dr Richard G. Doveston, Dr Anthony Aimon, Dr Romain Talon, Prof. Ian Churcher,
Prof. Frank von Delft, Prof. Stephen P Marsden*, Prof. Adam Nelson**

Supplementary Information

Contents:

1.0 Compound indices 4
1.1 By Fragment 4
1.2 By Scaffold 11
1.3 By Framework 15
2.0 Supplementary Figures 17
3.0 Supplementary Tables 24
4.0 Computational analysis of fragments and scaffolds 39
4.1 Molecular properties and natural product-likeness scores of the fragments 40
4.2 Natural product-likeness scores of the deprotected scaffolds 46
4.3 Summary of natural product-likeness scores of the fragments and scaffolds 49
4.4 Natural product-likeness scores of commercial libraries 49
5.0 Experimental 52
5.1 General experimental 52
5.2 General procedures 53
5.3 A note on NMR assignments 55
5.4 Compound data 56
5.4.1 Preparation of cycloaddition precursors and cycloadducts 56
5.4.1.1 Preparation of each intermediate in the synthesis of *O*-bridged cycloadduct 2a 56
5.4.1.2 Telescoped synthesis of *O*-bridged cycloadducts 2a-b 59
5.4.1.3 Preparation of *O*-bridged cycloadducts 2a and 2b 61
5.4.1.4 Preparation of *O*-bridged cycloadduct 2c 63
5.4.1.5 Preparation of precursors to *N*-bridged cycloadducts 2d-g 65
5.4.1.6 Preparation of the *N*-bridged cycloadducts 2d-g 67
5.4.2 Preparation of scaffolds 70
5.4.2.1 Scaffolds derived from cycloadduct 2a 70
5.4.2.1.1 Preparation of cyclic amine 4, and diols 7 and S13 71
5.4.2.1.2 Preparation of imidazole scaffolds 5 and S16 74

5.4.2.1.3 Preparation of silyl-protected amino alcohol scaffold 6 76
5.4.2.1.4 Preparation of oxazolidinone scaffold 8 79
5.4.2.1.5 Preparation of pyrazine scaffold 9 81
5.4.2.1.6 Preparation of piperazine scaffolds S19-S20 82
5.4.2.1.7 Preparation of quinoxaline scaffold S21 84
5.4.2.2 Scaffolds derived from cycloadduct 2b 85
5.4.2.2.1 Preparation of spirocyclic scaffold 3 and scaffold 12 85
5.4.2.2.2 Preparation of quinoxaline scaffold 10 90
5.4.2.2.3 Preparation of imidazole scaffold 11 91
5.4.2.3 Scaffolds derived from cycloadduct 2c 92
5.4.2.3.1 Preparation of silyl-protected amino alcohol scaffold 13 and alcohol S24 93
5.4.2.3.2 Preparation of quinoxaline scaffold 14 96
5.4.2.3.3 Preparation of diol S25 and cyclic amine 15 97
5.4.2.3.4 Preparation of oxazolidinone scaffold 16 99
5.4.2.4 Scaffolds derived from cycloadduct 2d 101
5.4.2.4.1 Preparation of scaffold 17 101
5.4.2.4.2 Preparation of scaffold S27 102
5.4.2.5 Scaffold derived from cycloadduct 2e 103
5.4.2.5.1 Preparation of scaffold 19 103
5.4.2.6 Scaffolds derived from cycloadduct 2f 105
5.4.2.6.1 Preparation of scaffold 18 106
5.4.2.6.2 Preparation of scaffolds 20 and S32-S34 107
5.4.2.6.3 Preparation of scaffold S35 113
5.4.3 Preparation of fragments 114
5.4.3.1 Preparation of fragments derived from cycloadduct 2g 120
5.4.3.2 Preparation of fragments derived from scaffold 3 123
5.4.3.3 Preparation of fragments derived from scaffold 7 124
5.4.3.4 Preparation of fragments derived from scaffold 8 131
5.4.3.5 Preparation of fragments derived from scaffold 9 134
5.4.3.6 Preparation of fragments derived from scaffold 13 136
5.4.3.7 Preparation of fragments derived from scaffold 14 140
5.4.3.8 Preparation of fragments derived from scaffold 15 141
5.4.3.9 Preparation of fragments derived from scaffold 16 144
5.4.3.10 Preparation of fragments derived from scaffold 17 145
5.4.3.11 Preparation of fragment derived from scaffold 18 147
5.4.3.12 Preparation of a fragment derived from scaffold 19 147
5.4.3.13 Preparation of fragments derived from scaffold 20 148

5.4.3.14 Preparation of fragments derived from scaffold S16 149
5.4.3.15 Preparation of a fragment derived from scaffold S19 153
5.4.3.16 Preparation of a fragment derived from scaffold S20 155
5.4.3.17 Preparation of a fragment derived from scaffold S21 156
5.4.3.18 Preparation of fragments derived from scaffold S24 157
5.4.3.19 Preparation of fragments derived from scaffold S25 160
5.4.3.20 Preparation of fragments derived from scaffold S27 168
5.4.3.21 Preparation of fragments derived from scaffold S32 171
5.4.3.22 Preparation of fragments derived from scaffold S33 and S34 171
6.0 High-throughput protein crystallography 173
7.0 Processed NMR spectra 174
8.0 References 428

1.0 Compound indices

1.1 By Fragment

Note that the following fragments from the manuscript are detailed throughout the Supporting Information using the aliases given below in parentheses:

- Compound **12** (**F12**)
- Compound **21** (**F8**)
- Compound **22** (**F31**)
- Compound **23** (**F48**)
- Compound **24** (**CF1**)
- Compound **25** (**F32**)

Entry	Fragment	Based on framework	Derivative of scaffold ##	Synthetically derived from…
1	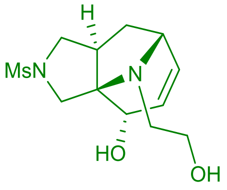 **F1**	**fw-22**	**S35**	2g → S36 → **F1**
2	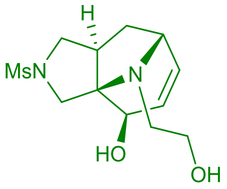 **F2**	**fw-22**	**S35**	2g → S36 → **F2**
3	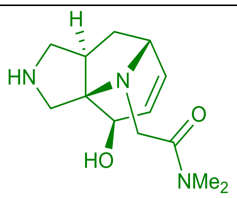 **F3**	**fw-22**	**S35**	2g → S37 → **F3**
4	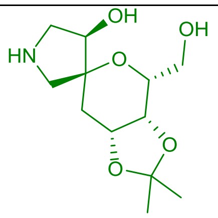 **F4**	**fw-1**	**3**	**3** → **F4**
5	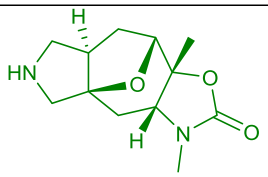	**fw-6**	**8**	**8** → **F5**

Entry	Fragment	Based on framework	Derivative of scaffold ##	Synthetically derived from…
	**F5**			
6	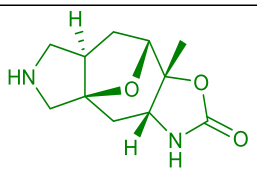 **F6**	**fw-6**	**8**	**F5** → **F6**
7	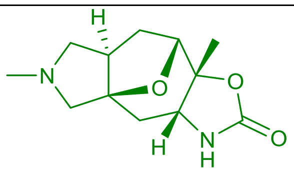 **F7**	**fw-6**	**8**	**F6** → **F7**
8	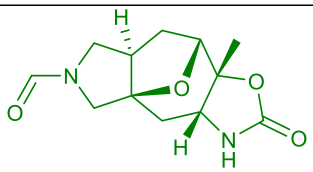 **F8 ≡ 21**	**fw-6**	**8**	**F6** → **F8**
9	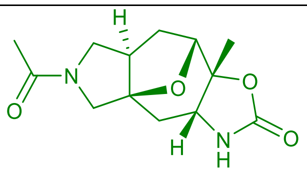 **F9**	**fw-6**	**8**	**F6** → **F9**
10	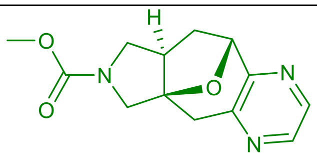 **F10**	**fw-7**	**9**	**9** → **F10**
11	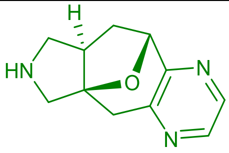 **F11**	**fw-7**	**9**	**9** → **F11**
12	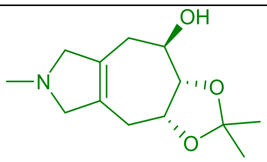 **F12 ≡ 12**	**fw-10**	**12**	2b → S22 → **F12**
13	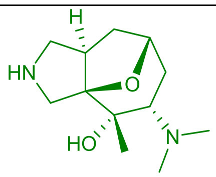 **F13**	**fw-4**	**13**	**13** → S38 → **F13**
14	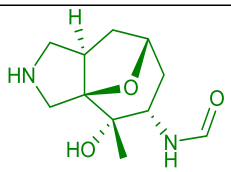 **F14**	**fw-4**	**13**	**13** → S39 → **F14**

Entry	Fragment	Based on framework	Derivative of scaffold ##	Synthetically derived from…
15	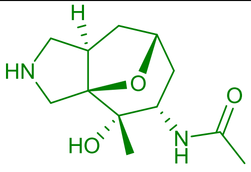 **F15**	**fw-4**	**13**	**13** → S40 → **F15**
16	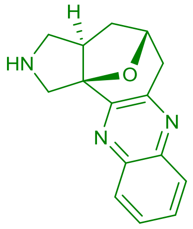 **F16**	**fw-11**	**14**	**14** → **F16**
17	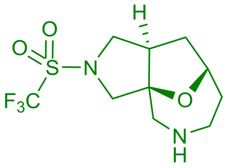 **F17**	**fw-12**	**15**	**15** → S41 → S42 → **F17**
18	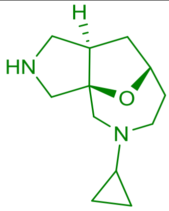 **F18**	**fw-12**	**15**	**S25** → S43 → **F18**
19	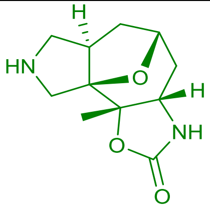 **F19**	**fw-13**	**16**	**16** → **F19**
20	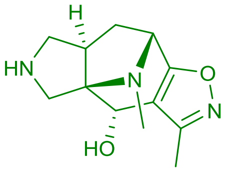 **F20**	**fw-14**	**17**	**17** → **F20**
21	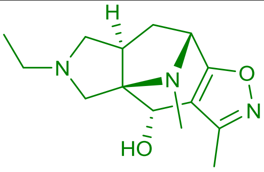 **F21**	**fw-14**	**17**	**F20** → **F21**
22	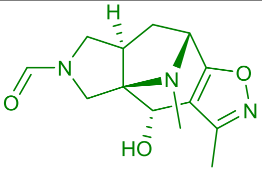 **F22**	**fw-14**	**17**	**F20** → **F22**

Entry	Fragment	Based on framework	Derivative of scaffold ##	Synthetically derived from…
23	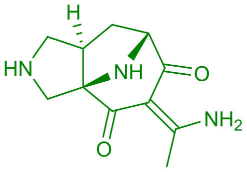<br>**F23**	**fw-15**	**18**	**18** → **F23**
24	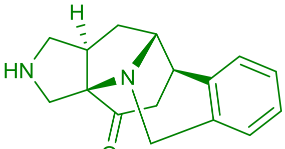<br>**F24**	**fw-16**	**19**	**19** → **F24**
25	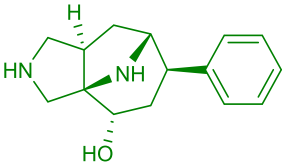<br>**F25**	**fw-17**	**20**	**20** → **F25**
26	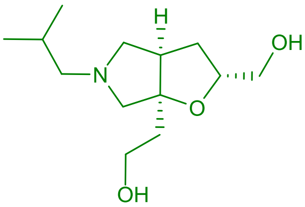<br>**F26**	**fw-5**	**7**	**7** → S44 → S45 → S46 → **F26**
27	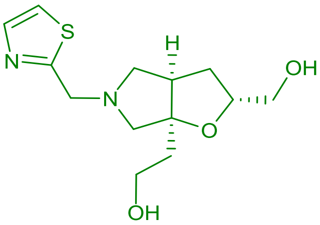<br>**F27**	**fw-5**	**7**	**7** → S44 → S45 → S47 → **F27**
28	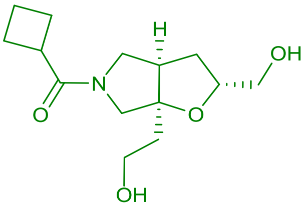<br>**F28**	**fw-5**	**7**	**7** → S44 → S45 → S48 → **F28**
29	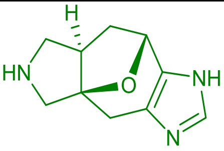<br>**F29**	**fw-18**	**S16**	**11** → **F29**<br>and/or<br>**S16** → **F29**
30	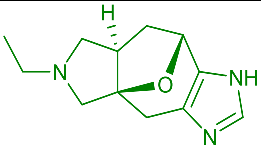<br>**F30**	**fw-18**	**S16**	**F29** → **F30**

Entry	Fragment	Based on framework	Derivative of scaffold ##	Synthetically derived from…
31	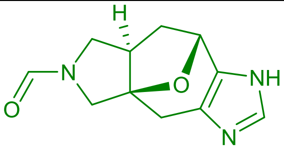 **F31 ≡ 22**	**fw-18**	**S16**	**F29** → **F31**
32	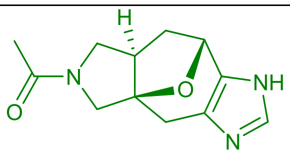 **F32 ≡ 25**	**fw-18**	**S16**	**F29** → **F32**
33	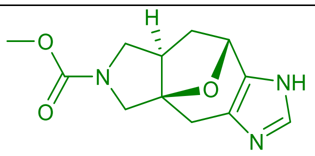 **F33**	**fw-18**	**S16**	**F29** → **F33**
34	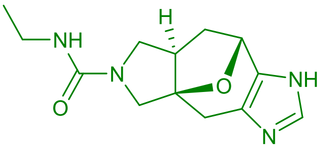 **F34**	**fw-18**	**S16**	**F29** → **F34**
35	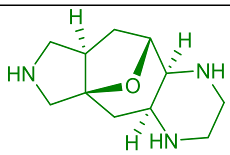 **F35**	**fw-19**	**S19**	**S19** → **F35**
36	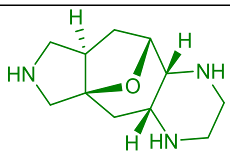 **F36**	**fw-19**	**S20**	**S20** → **F36**
37	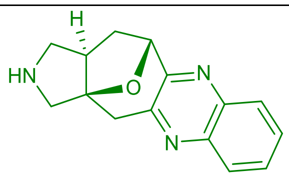 **F37**	**fw-20**	**S21**	**S21** → **F37**
38	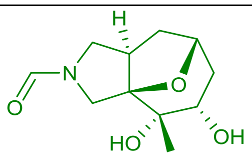 **F38**	**fw-4**	**S24**	**S24** → S49 → **F38**
39	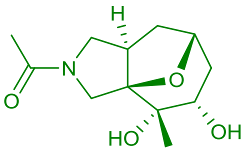 **F39**	**fw-4**	**S24**	**S24** → S49 → **F39**

Entry	Fragment	Based on framework	Derivative of scaffold ##	Synthetically derived from…
40	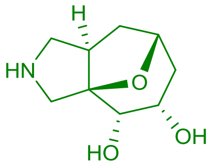 **F40**	**fw-4**	**S25**	**S25** → **F40**
41	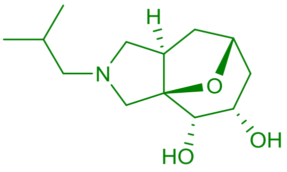 **F41**	**fw-4**	**S25**	2c → S50 and S51 → S52 and S53 → **F41**
42	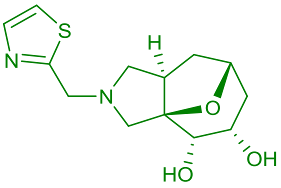 **F42**	**fw-4**	**S25**	2c → S50 and S51 → S52 and S53 → S54 → **F42**
43	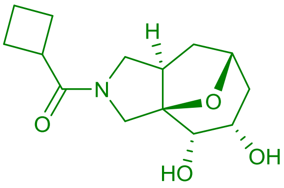 **F43**	**fw-4**	**S25**	2c → S50 and S51 → S52 and S53 → **F43**
44	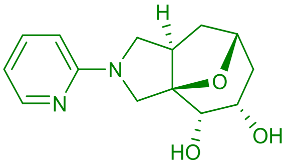 **F44**	**fw-4**	**S25**	2c → S50 and S51 → S52 and S53 → **F44**
45	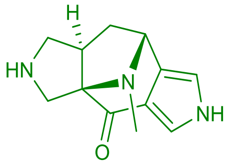 **F45**	**fw-21**	**S27**	**S27** → **F45**
46	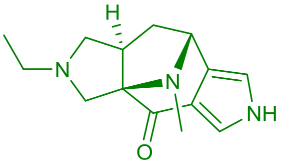 **F46**	**fw-21**	**S27**	**F45** → **F46**
47	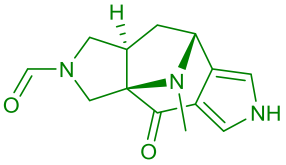 **F47**	**fw-21**	**S27**	**F45** → **F47**
48	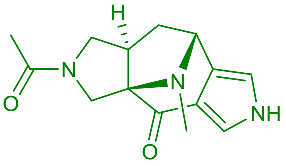	**fw-21**	**S27**	**F45** → **F48**

Entry	Fragment	Based on framework	Derivative of scaffold ##	Synthetically derived from…
	F48 ≡ 23			
49	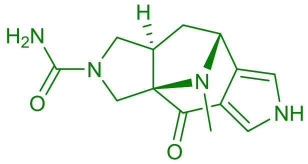<br>F49	fw-21	S27	F45 → F49
50	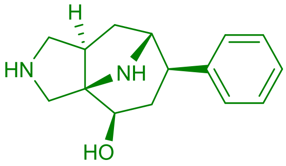<br>F50	fw-16	S32	S32 → F50
51	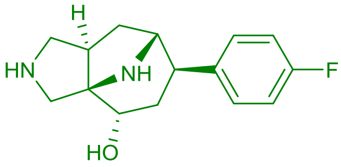<br>F51	fw-16	S33	S33 → F51
52	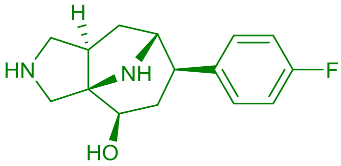<br>F52	fw-16	S34	S34 → F52

1.2 By Scaffold

See Supplementary Figure 1 for a full summary of the synthetic routes used to prepare the scaffolds.

To count the scaffolds prepared in this study we have considered some closely related scaffolds to be equivalent. The following groups of compounds are counted as a sinlge scaffold:

- **20 ≡ S32 ≡ S33 ≡ S34**
- **S19 ≡ S20**

Entry	Scaffold	Based on framework	Derivative fragments
1	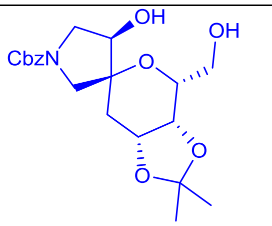 **3**	**fw-1**	**F4**
2	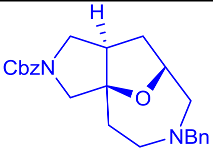 **4**	**fw-2**	**-**
3	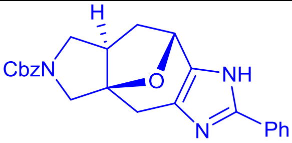 **5**	**fw-3**	**-**
4	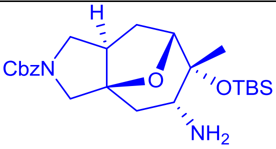 **6**	**fw-4**	**-**
5	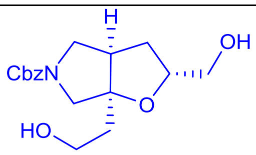 **7**	**fw-5**	**F26-F28**
6	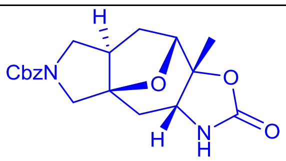 **8**	**fw-6**	**F5-F9**

Entry	Scaffold	Based on framework	Derivative fragments
7	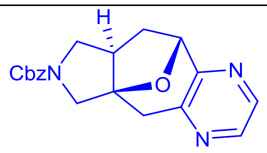 **9**	**fw-7**	**F10-F11**
8	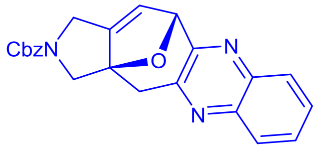 **10**	**fw-8**	**-**
9	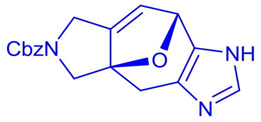 **11**	**fw-9**	**-**
10	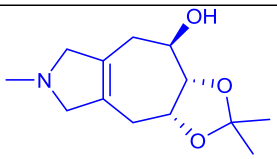 **12**	**fw-10**	**F12**
11	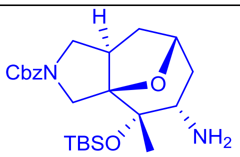 **13**	**fw-4**	**F13-F15**
12	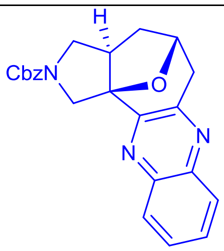 **14**	**fw-11**	**F16**
13	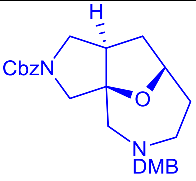 **15**	**fw-12**	**F17-F18**
14	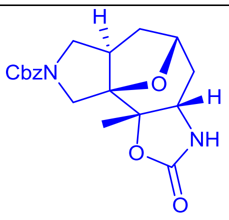 **16**	**fw-13**	**F19**
15	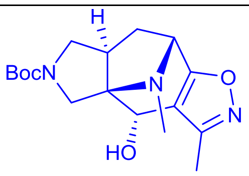 **17**	**fw-14**	**F20-F22**

Entry	Scaffold	Based on framework	Derivative fragments
16	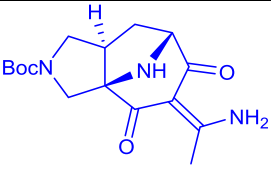 **18**	**fw-15**	**F23**
17	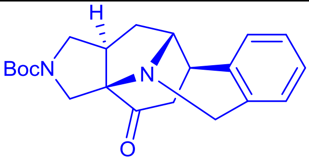 **19**	**fw-16**	**F24**
18	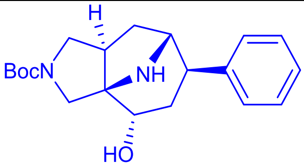 **20**	**fw-17**	**F25**
19	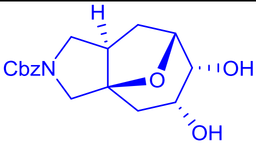 **S13**	**fw-4**	**-**
20	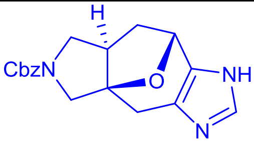 **S16**	**fw-18**	**F29-F34**
21	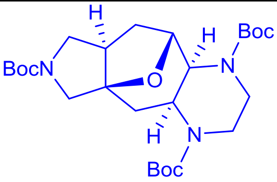 **S19**	**fw-19**	**F35**
22	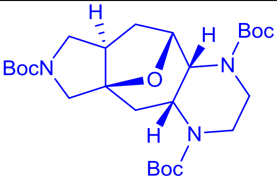 **S20**	**fw-19**	**F36**
23	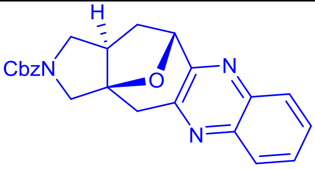 **S21**	**fw-20**	**F37**
24	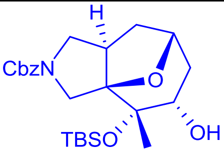 **S24**	**fw-4**	**F38-F39**

Entry	Scaffold	Based on framework	Derivative fragments
25	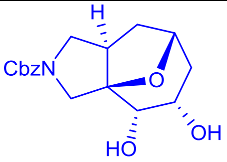 **S25**	**fw-4**	**F40-F44**
26	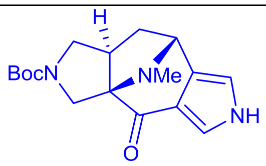 **S27**	**fw-21**	**F45-F49**
27	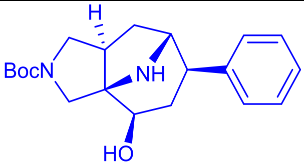 **S32**	**fw-17**	**F50**
28	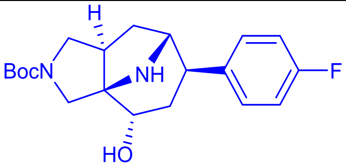 **S33**	**fw-17**	**F51**
29	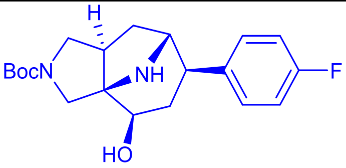 **S34**	**fw-17**	**F52**
30	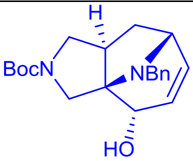 **S35**	**fw-22**	**F1-F3**

1.3 By Framework

Framework number	Graph-node-bond framework	Derivative Scaffold(s)	Derivative Fragments
**fw-1**	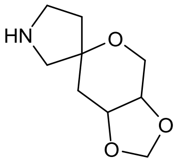	**3**	**F4**
**fw-2**	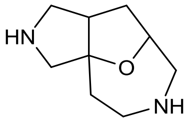	**4**	**-**
**fw-3**	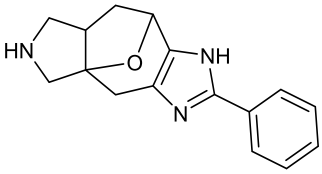	**5**	**-**
**fw-4**	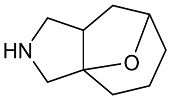	**6, 13, S13, S24-S25**	**F13-F15, F38-F44**
**fw-5**	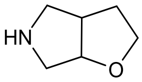	**7**	**F26-28**
**fw-6**	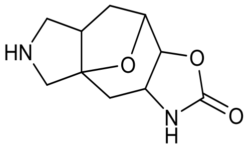	**8**	**F5-F9**
**fw-7**	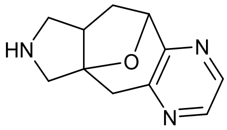	**9**	**F10-F11**
**fw-8**	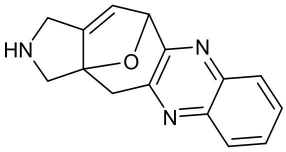	**10**	**-**
**fw-9**	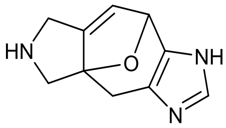	**11**	**-**
**fw-10**	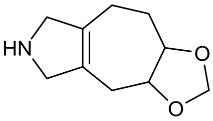	**12**	**F12**
**fw-11**	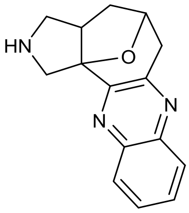	**14**	**F16**
**fw-12**	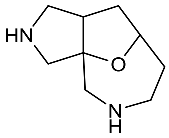	**15**	**F17-F18**

**fw-13**	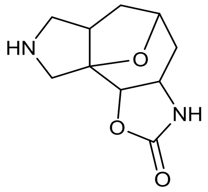	**16**	**F19**
**fw-14**	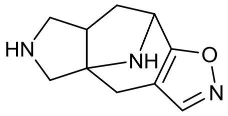	**17**	**F20-F22**
**fw-15**	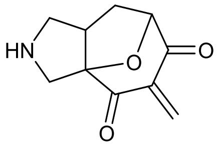	**18**	**F23**
**fw-16**	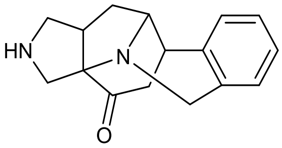	**19**	**F24**
**fw-17**	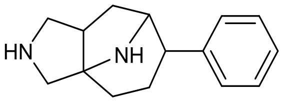	**20, S32, S33-S34**	**F25, F50-52**
**fw-18**	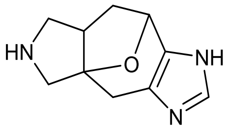	**S16**	**F29-F34**
**fw-19**	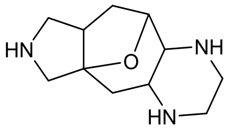	**S19- S20**	**F35-F36**
**fw-20**	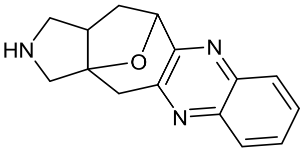	**S21**	**F37**
**fw-21**	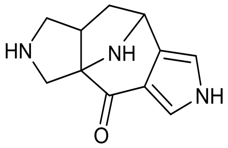	**S27**	**F45-F49**
**fw-22**	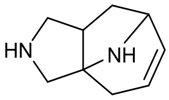	**S35**	**F1-F3**

2.0 Supplementary Figures

Supplementary Figure 1 Synthesis of the 26 natural product-like scaffolds (blue).

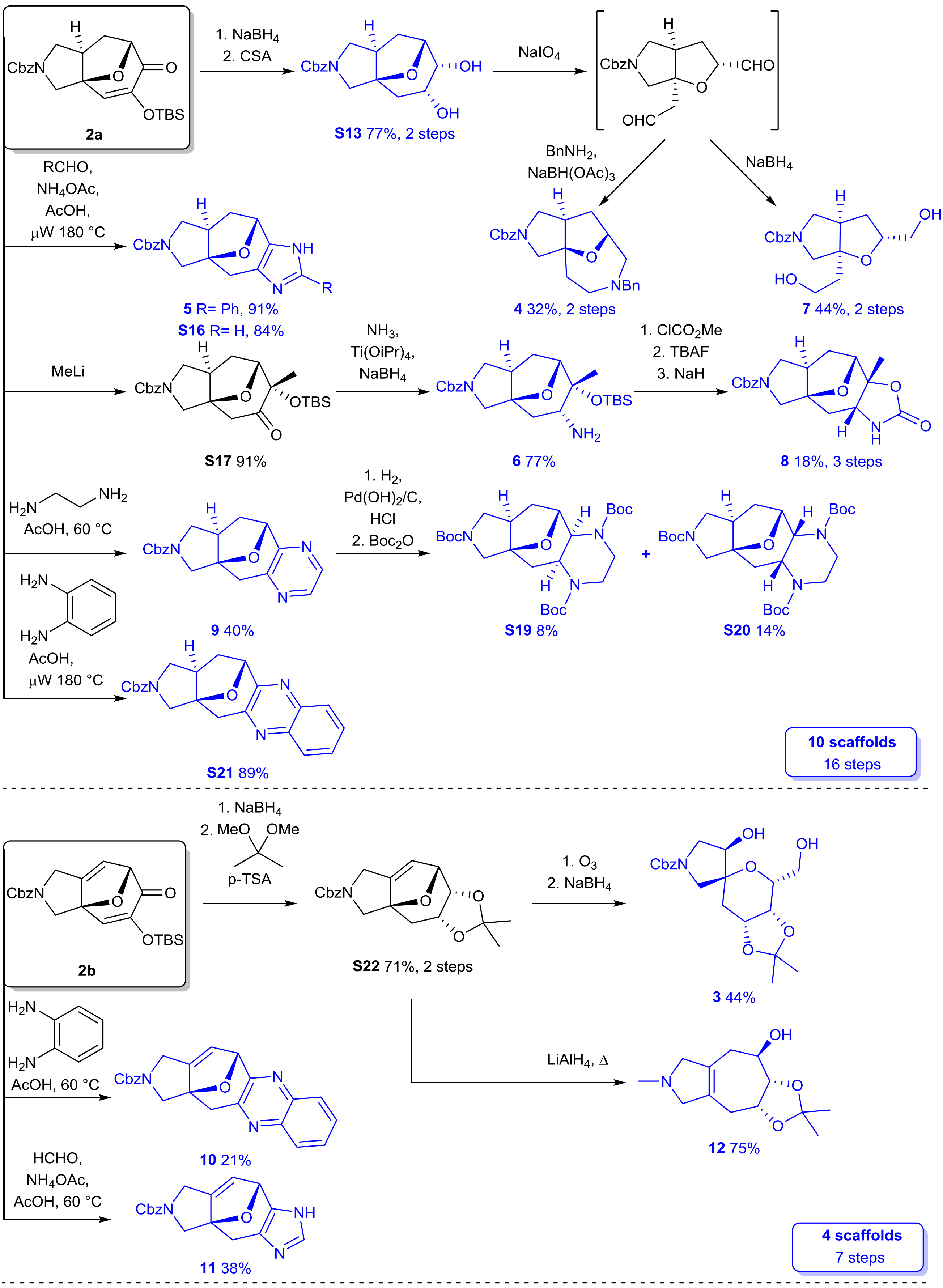

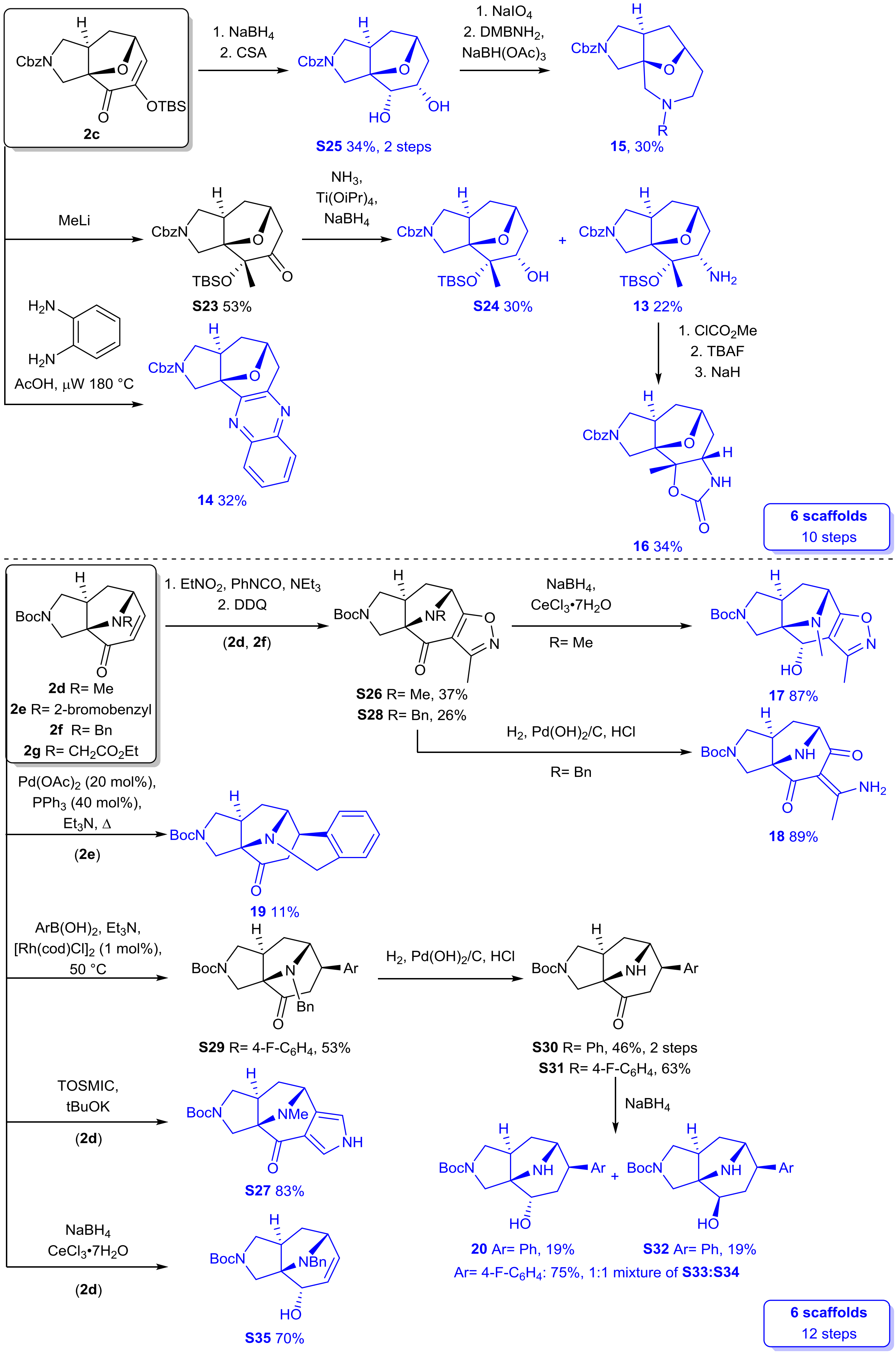

2c
1. NaBH4
2. CSA
S25 34%, 2 steps
1. NaIO4
2. DMBNH2,
NaBH(OAc)3
15, 30%
MeLi
S23 53%
NH3,
Ti(OiPr)4,
NaBH4
S24 30%
13 22%
1. ClCO2Me
2. TBAF
3. NaH
16 34%
AcOH, μW 180 °C
14 32%
6 scaffolds
10 steps
2d R= Me
2e R= 2-bromobenzyl
2f R= Bn
2g R= CH2CO2Et
1. EtNO2, PhNCO, NEt3
2. DDQ
(2d, 2f)
S26 R= Me, 37%
S28 R= Bn, 26%
NaBH4,
CeCl3•7H2O
R= Me
17 87%
H2, Pd(OH)2/C, HCl
R= Bn
18 89%
Pd(OAc)2 (20 mol%),
PPh3 (40 mol%),
Et3N, Δ
(2e)
19 11%
ArB(OH)2, Et3N,
[Rh(cod)Cl]2 (1 mol%),
50 °C
S29 R= 4-F-C6H4, 53%
H2, Pd(OH)2/C, HCl
S30 R= Ph, 46%, 2 steps
S31 R= 4-F-C6H4, 63%
NaBH4
TOSMIC,
tBuOK
(2d)
S27 83%
NaBH4
CeCl3•7H2O
(2d)
S35 70%
20 Ar= Ph, 19%
S32 Ar= Ph, 19%
Ar= 4-F-C6H4: 75%, 1:1 mixture of S33:S34
6 scaffolds
12 steps

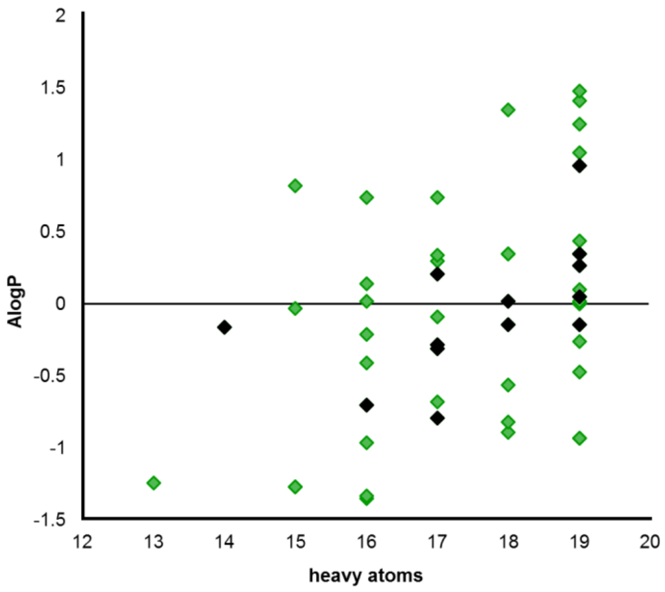

Supplementary Figure 2 Molecular properties of the 52 fragments prepared. Hits are shown in black.

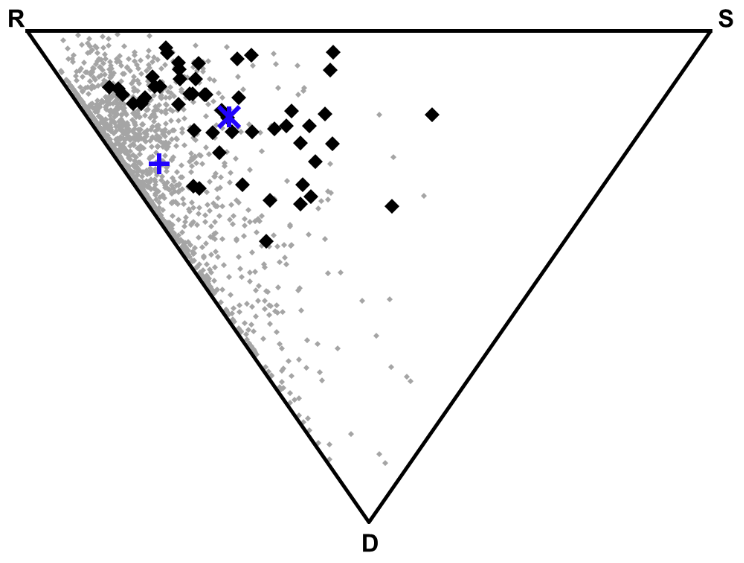

Supplementary Figure 3 Shape diversity of the 52 fragments prepared and 1,236 commerically-available fragments. R, rod; S, sphere; D, disk.

Supplementary Figure 4 Interactions between seven fragment hits based on the natural product-like scaffolds and the bromodomain of ATAD2. See Supplementary Table 2 (entries 2-8) for further details. H-bonding interactions of ≤ 3.5 Å are shown.

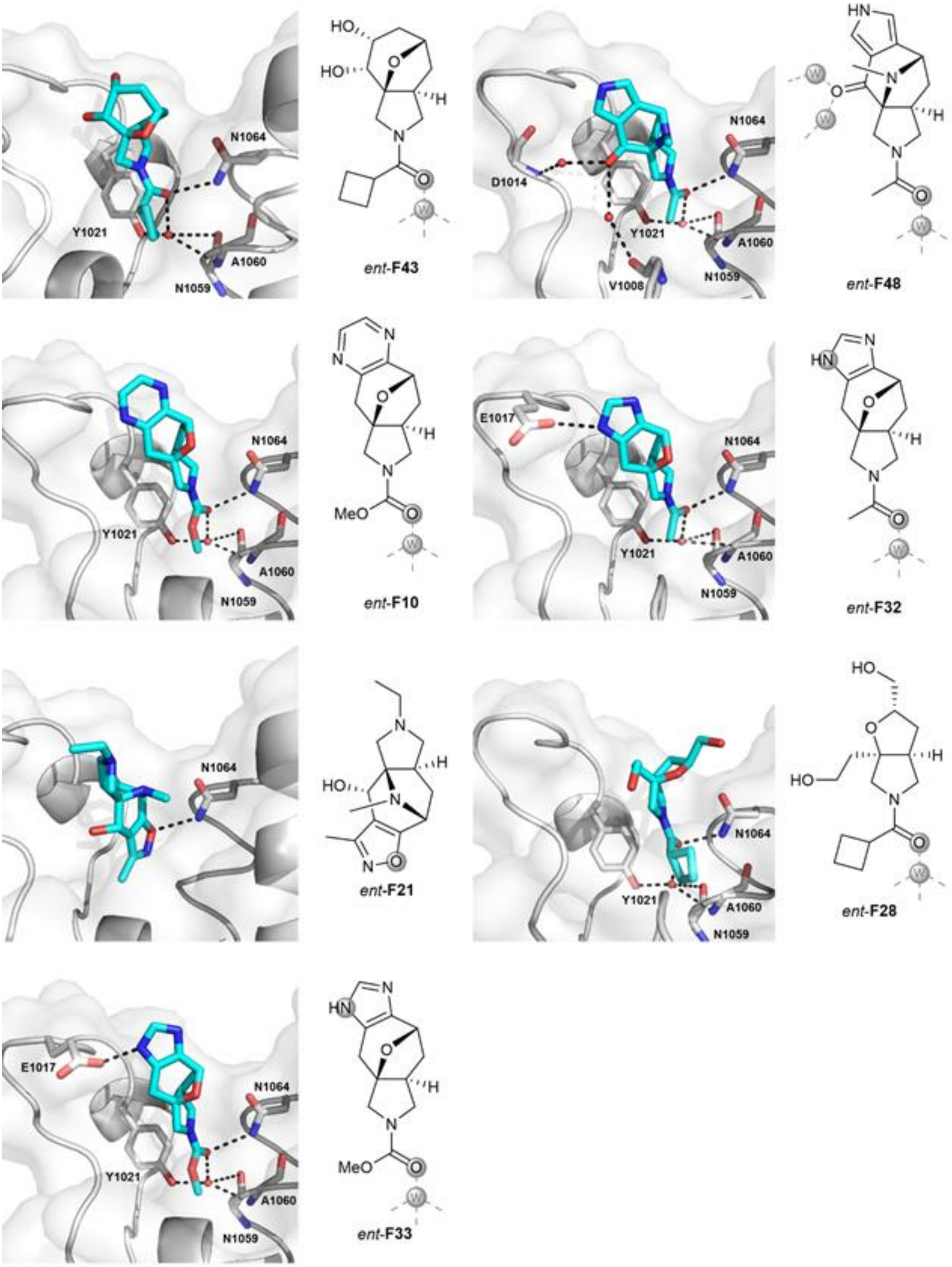

Supplementary Figure 5 Interactions between eight fragment hits based on the natural product-like scaffolds and the bromodomain of BRD1. See Supplementary Table 3 for further details. H-bonding interactions of ≤ 3.5 Å are shown.

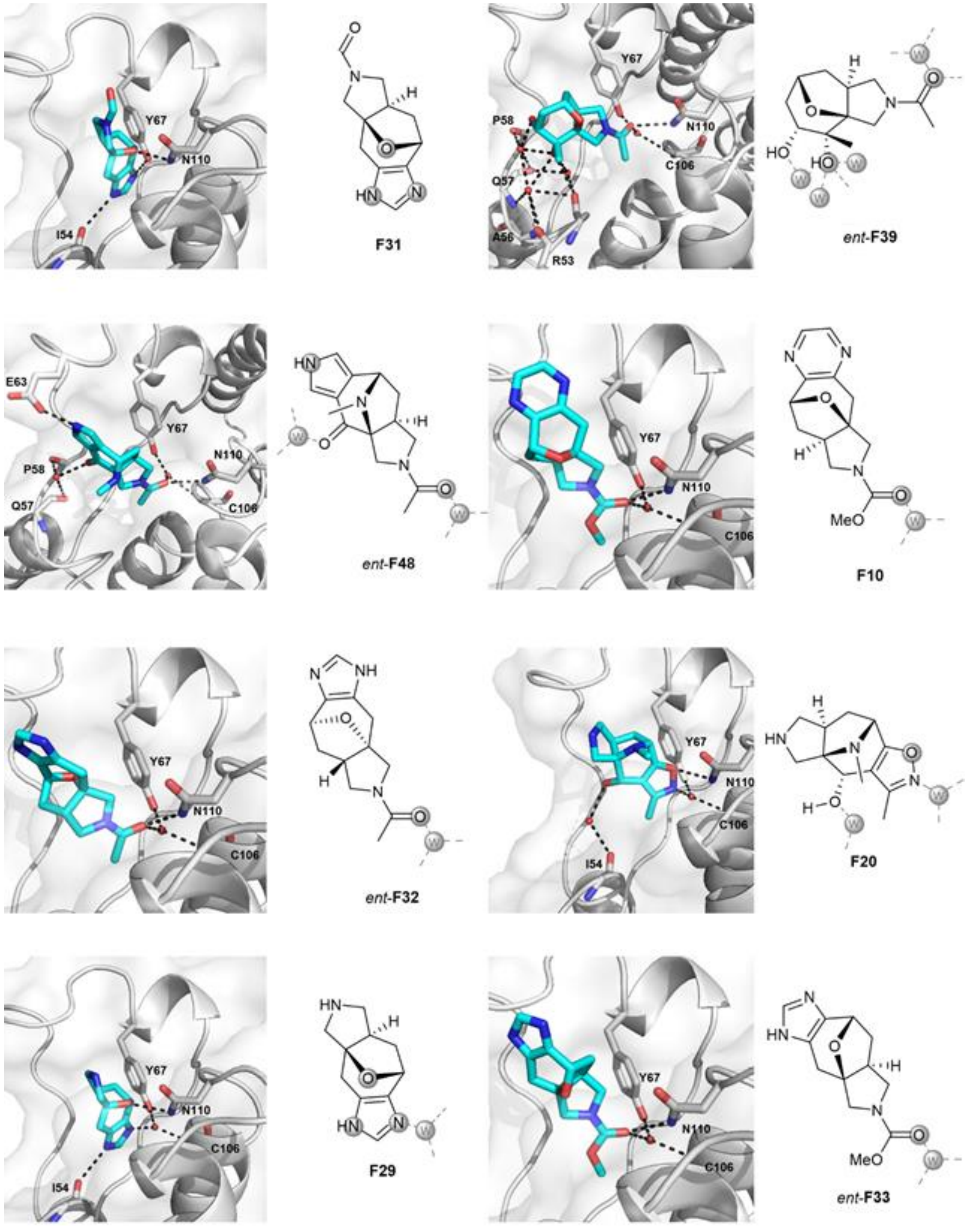

Supplementary Figure 6 Interactions between two fragment hits **based on the natural product-like scaffolds** and a peripheral binding pocket of JMJD2D. See Supplementary Table 4 for further details. H-bonding interactions of ≤ 3.5 Å are shown.

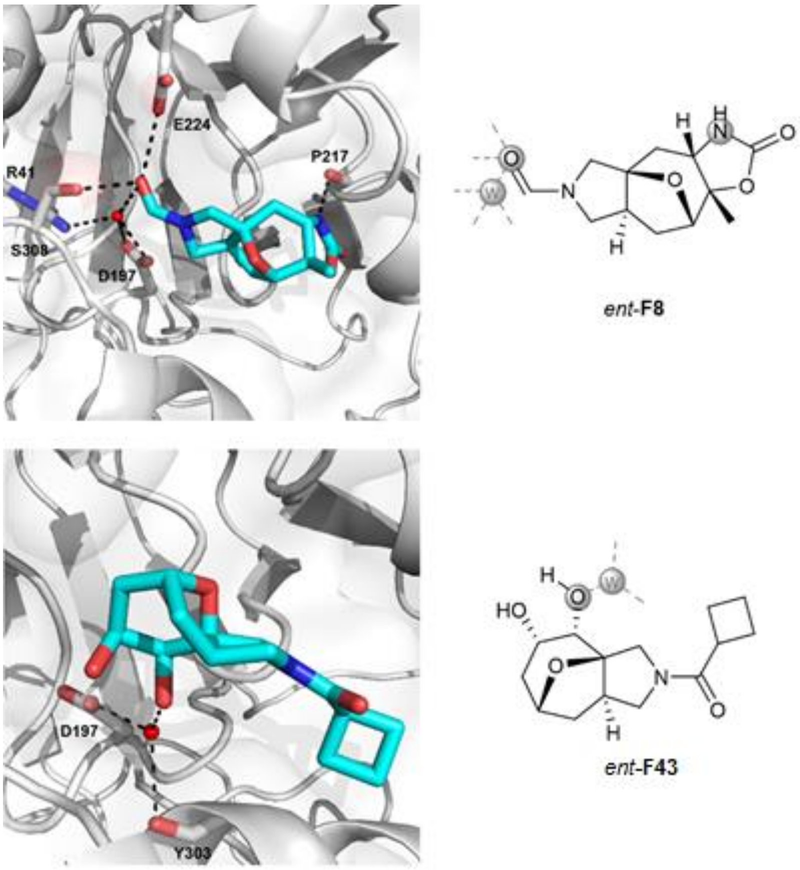

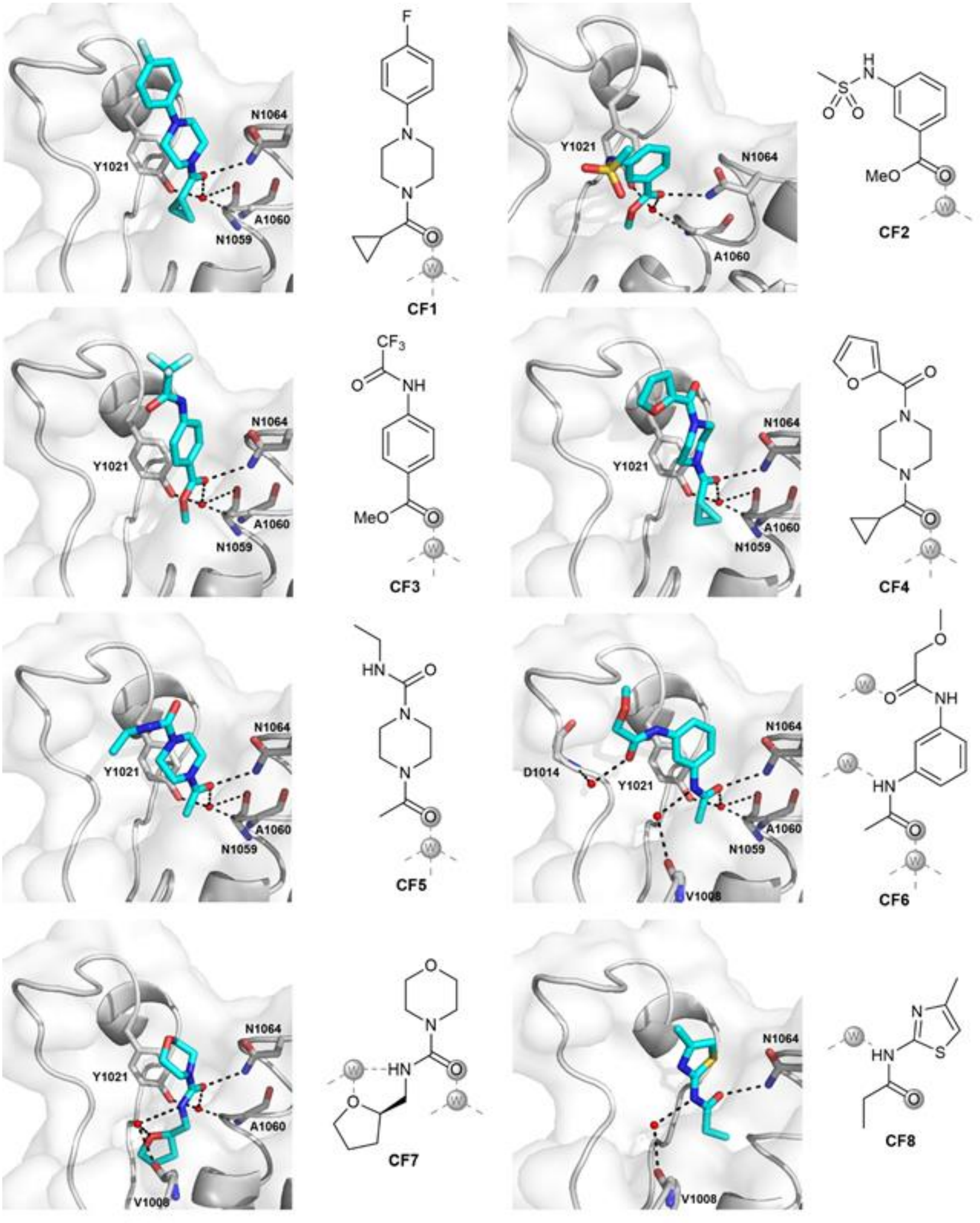

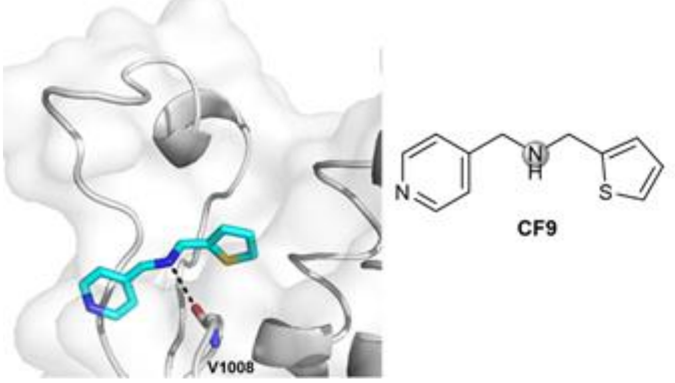

Supplementary Figure 7 Interactions of nine hits from a screen of 700 commercially-available fragments against the bromodomain of ATAD2. See Supplementary Table 2 (entries 9-17) for further details. H-bonding interactions of ≤ 3.5 Å are shown.

3.0 Supplementary Tables

Supplementary Table 1 Substructure occurrence of parent and daughter frameworks of the 26 scaffolds within the 281,897 structures found in the Dictionary of Natural Products (accessed 02/11/16). All other frameworks (Figure 3) produced no hits in the sub-structure search.

Scaffold	No. NPs containing framework	% of NPs containing framework
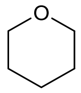	55131	19.6
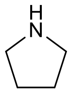	9150	3.2
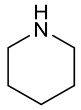	9080	3.2
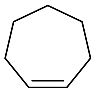	4421	1.6
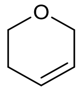	3279	1.2
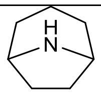	805	0.29
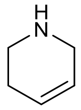	767	0.27
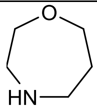	264	0.09
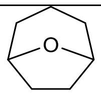	251	0.09
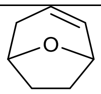	75	0.03
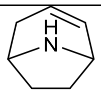	16	0.006
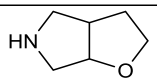	14	0.005
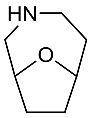	13	0.005
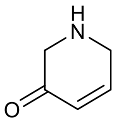	3	0.0001
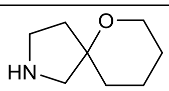	2	0.0007

Supplementary Table 2 Summary of interactions between fragment hits and ATAD2. Ligands include the truncated acetyl lysine ligand (Entry 1)[S1], the seven fragment hits based on the natural product-like scaffolds that were identified in this work using high-throughput X-ray crystallography (Entries 2-8), nine commercially available fragment hits identified in this work using high-throughput X-ray crystallography (Entries 9-17), other known fragment hits (criteria: 12 ≤ HA ≤ 19; cyclic) and published hits that were identified using a range of methods (conventional hits entries 18-25;[S1-S4] and thymidine hits entries 26-28[S1]). For comparison, the interactions made by a recently identified chemical probe (Entry 29)[S5] are also provided. H-bonding interactions of ≤ 3.5 Å are listed. Where bridging waters interact with more than one side chain, the interaction containing the shortest H-bond forms the title interaction, and any remaining interactions are detailed in parentheses.

Entry	Ligand Structure	N1064, γ-O	N1064, γ-NH_2	H_2O (→Y1021, Ar-OH)	E1017, δ-O	H_2O (→D1014, α-N)	H_2O (→V1008, β-O)	V1008, β-O	D1071, γ -O	D1014, α-N	I1056, β-O	K1011, β-O	M1029, β-O	R1007, ζ-NH_2	H_2O (→I1056, β-O)	H_2O (→P1012, β-O)	H_2O (→M1029, β-O)
**Natural ligand (truncated)**																	
1	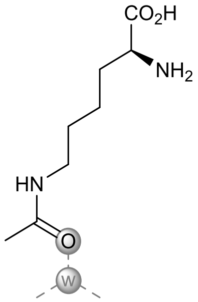 **PDB: 4QSP**		2.7	2.7 (& →A1060, α-N)													
**Fragments based on natural product paralogues**																	
2	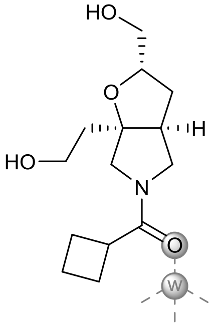 *ent*-**F28**		2.9	2.8		3.5 (& → E1017, δ-O)											

Entry	Ligand Structure	N1064, γ-O	N1064, γ-NH_2	H_2O (→Y1021, Ar-OH)	E1017, δ-O	H_2O (→D1014, α-N)	H_2O (→V1008, β-O)	V1008, β-O	D1071, γ -O	D1014, α-N	I1056, β-O	K1011, β-O	M1029, β-O	R1007, ζ-NH_2	H_2O (→I1056, β-O)	H_2O (→P1012, β-O)	H_2O (→M1029, β-O)
3	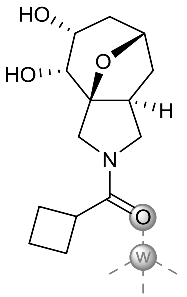 *ent*-**F43**		2.9	2.8		3.5											
4	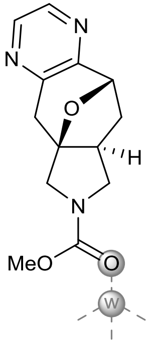 *ent*-**F10**		3.1	2.8 (& →N1059, β-O)	3.1												
5	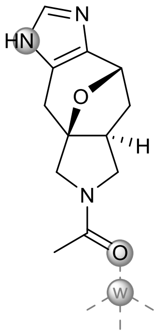 *ent*-**F32**		3.0	2.8 (& →N1059, β-O)	3.1												

Entry	Ligand Structure	N1064, γ-O	N1064, γ-NH_2	H_2O (→Y1021, Ar-OH)	E1017, δ-O	H_2O (→D1014, α-N)	H_2O (→V1008, β-O)	V1008, β-O	D1071, γ -O	D1014, α-N	I1056, β-O	K1011, β-O	M1029, β-O	R1007, ζ-NH_2	H_2O (→I1056, β-O)	H_2O (→P1012, β-O)	H_2O (→M1029, β-O)
6	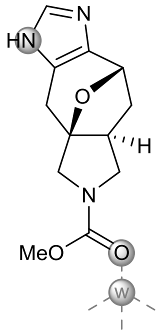 *ent*-**F33**		3.1	2.8 (& →N1059, β-O)	2.8												
7	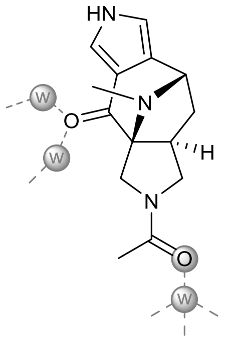 *ent*-**F48**		3.0	2.7	2.9, 3.1	2.3	3.3										
8	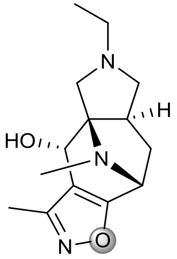 *ent*-**F21**		3.0														

Entry	Ligand Structure	N1064, γ-O	N1064, γ-NH_2	H_2O (→Y1021, Ar-OH)	E1017, δ-O	H_2O (→D1014, α-N)	H_2O (→V1008, β-O)	V1008, β-O	D1071, γ -O	D1014, α-N	I1056, β-O	K1011, β-O	M1029, β-O	R1007, ζ-NH_2	H_2O (→I1056, β-O)	H_2O (→P1012, β-O)	H_2O (→M1029, β-O)
**Diamond-SGC Poised Library (DSPL) hits**																	
9	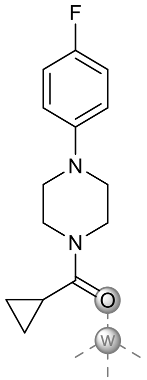<br>**24 ≡ CF1**		3.1	2.7<br>(&<br>→A1060,<br>α-N;<br>N1059,<br>β-O)													
10	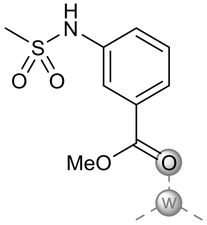<br>**CF2**		3.0	2.8<br>(&<br>→A1060,<br>α-N)													
11	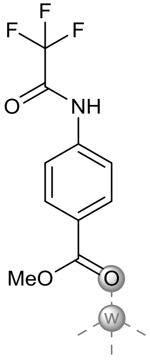<br>**CF3**		3.1	2.8<br>(&<br>→A1060,<br>α-N;<br>N1059,<br>β-O)													

Entry	Ligand Structure	N1064, γ-O	N1064, γ-NH_2	H_2O (→Y1021, Ar-OH)	E1017, δ-O	H_2O (→D1014, α-N)	H_2O (→V1008, β-O)	V1008, β-O	D1071, γ -O	D1014, α-N	I1056, β-O	K1011, β-O	M1029, β-O	R1007, ζ-NH_2	H_2O (→I1056, β-O)	H_2O (→P1012, β-O)	H_2O (→M1029, β-O)
12	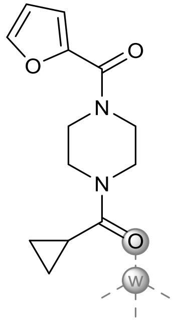 **CF4**		3.1	2.8 (& →A1060, α-N; N1059, β-O)													
13	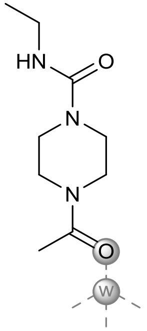 **CF5**		3.0	2.8 (& →A1060, α-N; N1059, β-O)													
14	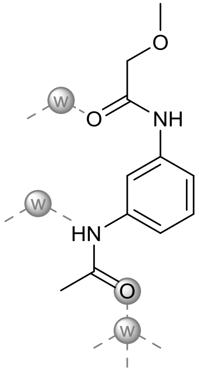 **CF6**		3.2	2.8 (& →A1060, α-N; N1059, β-O)		2.8	3.1										

Entry	Ligand Structure	N1064, γ-O	N1064, γ-NH_2	H_2O (→Y1021, Ar-OH)	E1017, δ-O	H_2O (→D1014, α-N)	H_2O (→V1008, β-O)	V1008, β-O	D1071, γ -O	D1014, α-N	I1056, β-O	K1011, β-O	M1029, β-O	R1007, ζ-NH_2	H_2O (→I1056, β-O)	H_2O (→P1012, β-O)	H_2O (→M1029, β-O)
15	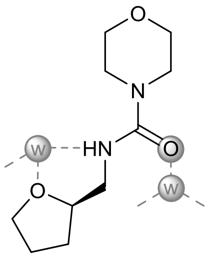 **CF7**		3.0	2.7 (& →A1060, α-N)			2.7, 3.5										
16	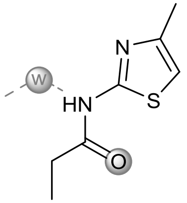 **CF8**		3.1				2.9										
17	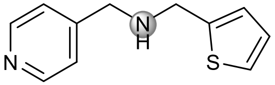 **CF9**							2.9									

Entry	Ligand Structure	N1064, γ-O	N1064, γ-NH_2	H_2O (→Y1021, Ar-OH)	E1017, δ-O	H_2O (→D1014, α-N)	H_2O (→V1008, β-O)	V1008, β-O	D1071, γ -O	D1014, α-N	I1056, β-O	K1011, β-O	M1029, β-O	R1007, ζ-NH_2	H_2O (→I1056, β-O)	H_2O (→P1012, β-O)	H_2O (→M1029, β-O)
**Other literature hits (13 ≤ HA ≤ 19)**																	
18	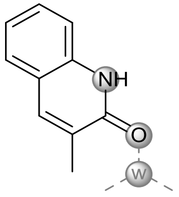 **PDB: 5A5O**	3.1	2.7	2.7 (& →A1060, α-N)													
19	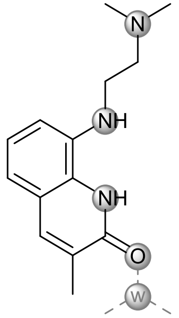 **PDB: 5A5P**	3.1; 3.1	2.7	2.7 (& →A1060, α-N)					2.9								
20	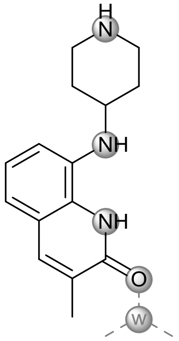 **PDB: 5A5Q**	3.0; 2.9	2.8	2.7 (& →A1060, α-N)					2.6								

Entry	Ligand Structure	N1064, γ-O	N1064, γ-NH_2	H_2O (→Y1021, Ar-OH)	E1017, δ-O	H_2O (→D1014, α-N)	H_2O (→V1008, β-O)	V1008, β-O	D1071, γ -O	D1014, α-N	I1056, β-O	K1011, β-O	M1029, β-O	R1007, ζ-NH_2	H_2O (→I1056, β-O)	H_2O (→P1012, β-O)	H_2O (→M1029, β-O)
21	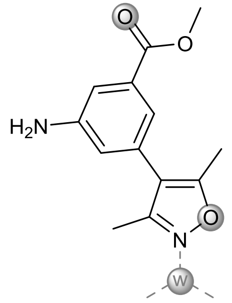 **PDB: 4TTE**		3.0	2.7 (& →A1060, α-N)										3.5			
22	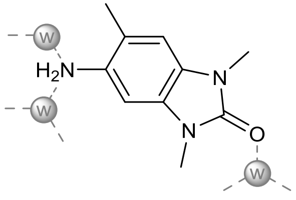 **PDB: 4TYL**		3.1	2.7 (& →A1060, α-N; N1059, β-O)		3.2										2.8 (& →K1011, β-O)	
23	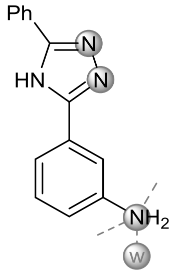 **PDB: 4TZ2**		2.6; 3.1	2.7							2.9		3.4				
**24**	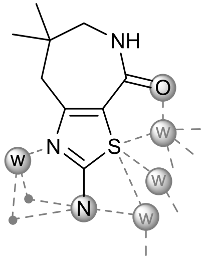 **PDB: 4TZ8**		3.0	3.2; 3.1 (& →A1060, α-N; N1059, β-O)			2.9 (& → K1011, β-O)	3.0				3.1			3.5		2.6, 3.3

Entry	Ligand Structure	N1064, γ-O	N1064, γ-NH_2	H_2O (→Y1021, Ar-OH)	E1017, δ-O	H_2O (→D1014, α-N)	H_2O (→V1008, β-O)	V1008, β-O	D1071, γ -O	D1014, α-N	I1056, β-O	K1011, β-O	M1029, β-O	R1007, ζ-NH_2	H_2O (→I1056, β-O)	H_2O (→P1012, β-O)	H_2O (→M1029, β-O)
**25**	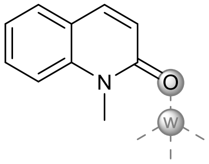 **PDB: 4QST**		2.8	2.5 (& →A1060, α-N; N1059, β-O)													
**Thymidine literature hits**																	
**26**	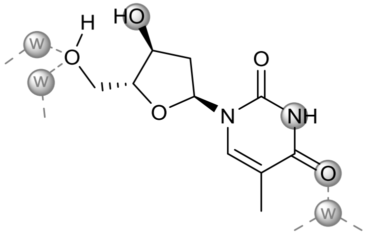 **PDB: 4QSV**	3.1	2.9	2.6 (& →A1060, α-N)	3.4	2.8	2.6										
**27**	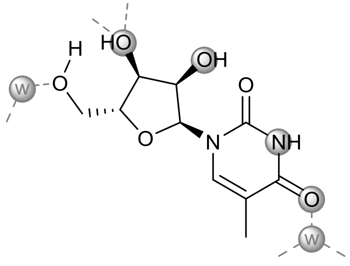 **PDB: 4QSW**	3.1	2.8	2.6 (& →A1060, α-N)	3.2; 3.1		2.5			3.2							
**28**	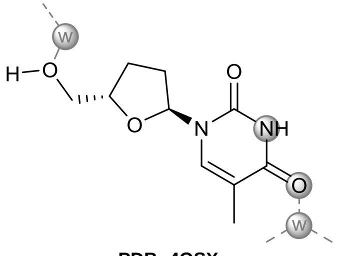 **PDB: 4QSX**	2.8	2.8	2.7 (& →A1060, α-N)		2.9											

Entry	Ligand Structure	N1064, γ-O	N1064, γ-NH_2	H_2O (→Y1021, Ar-OH)	E1017, δ-O	H_2O (→D1014, α-N)	H_2O (→V1008, β-O)	V1008, β-O	D1071, γ -O	D1014, α-N	I1056, β-O	K1011, β-O	M1029, β-O	R1007, ζ-NH_2	H_2O (→I1056, β-O)	H_2O (→P1012, β-O)	H_2O (→M1029, β-O)
**Established chemical probe**																	
**29**	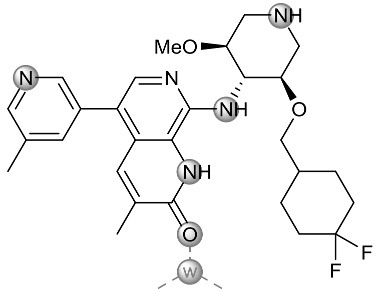 **PDB: 5LJ0**	2.9; 2.8	2.8	2.7 (& →A1060, α-N)					2.6	3.1							

Supplementary Table 3 Summary interactions between eight fragment hits based on the natural product-like scaffolds and BRD1, identified using high-throughput X-ray crystallography.

Entry	Ligand Structure	N110, α-N	H_2O (→Y67, Ar-OH)	I54, β-O	H_2O (→I54, β-O)	H_2O (→P58, β-O)	H2O (→Q57, α-N)	H2O (→Q57, β-N)	Y67, Ar-OH	E63, δ-O	Notes
1	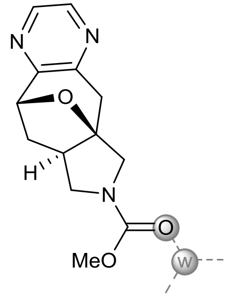 **26** (≡ **F10**)	3.0	2.8 [& C106, α-N]								**The enantiomer was an ATAD2 hit**
2	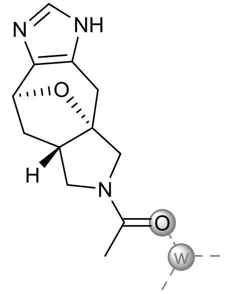 *ent*-**28** (≡ *ent*-**F32**)	3.2	2.7 [& C106, α-N]								**ATAD2 hit**
3	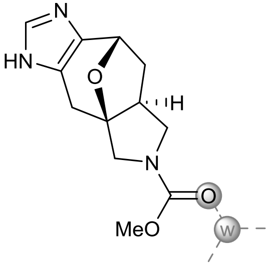 *ent*-**F33**	3.1	2.9 [& C106, α-N]]								**ATAD2 hit**

Entry	Ligand Structure	N110, α-N	H_2O (→Y67, Ar-OH)	I54, β-O	H_2O (→I54, β-O)	H_2O (→P58, β-O)	H2O (→Q57, α-N)	H2O (→Q57, β-N)	Y67, Ar-OH	E63, δ-O	Notes
4	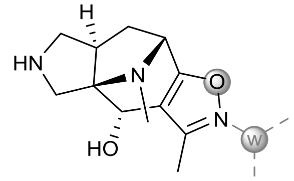 **F20**	3.0	2.8 [& C106, α-N]		2.7						
5	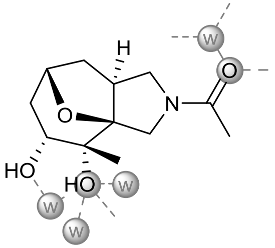 *ent*-**24** (≡ *ent*-**F39**)	3.0	2.8 [& C106, α-N]	3.2		2.6, 2.6 [& →Q57, β-O; P58, β-O]	3.1 [& → R53, I54 and Q57, β-O; & A56, Q57, α-N]	3.4 [& →I54, β-O]			
6	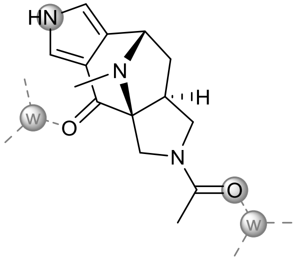 *ent*-**25** (≡ *ent*-**F48**)	3.0	2.7 [& C106, α-N]			2.7 [& →Q57, β-O]				2.5	**ATAD2 hit**
7	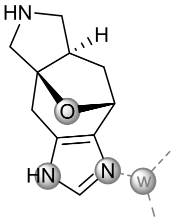 **F29**	3.0	2.2 [& C106, α-N]	3.2							

Entry	Ligand Structure	N110, α-N	H_2O (→Y67, Ar-OH)	I54, β-O	H_2O (→I54, β-O)	H_2O (→P58, β-O)	H2O (→Q57, α-N)	H2O (→Q57, β-N)	Y67, Ar-OH	E63, δ-O	Notes
8	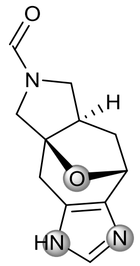<br>**23** (≡ **F31**)	2.8		3.3					2.9		

Supplementary Table 4 Summary interactions between two fragment hits based on the natural product-like scaffolds and JMJD2D, identified using high-throughput X-ray crystallography.

Entry	Ligand Structure	S308, β-OH	E224, δ-O	P217, β-O	H_2O (→D197, γ-O)
1	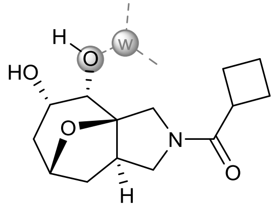 **22**				2.5 [→Y303, β-O]
2	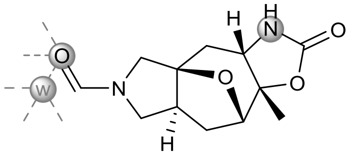 *ent*-**F8**	2.7	3.4	3.2	2.7, 3.5 [& →R41, ζ-NH_2)

4.0 Computational analysis of fragments and scaffolds

Natural product likeness scores[S6] were calculated using the implementation in RDKit v2015.09.2 (Greg Landrum; Open Source Cheminformatics; http://www.rdkit.org; last accessed 27/04/2016).

For the preparation of Figure 2 , databases of commercially available screening compounds were downloaded and filtered for 13 ≤ heavy atoms ≤ 19 before comparison with the fragments. The databases used were:

a) BIONET – Fragments from Nature (128 compounds; http://www.keyorganics.net/downloads/; downloaded 24/08/2016).

b) The Enamine General Fragment Library (12,486 compounds; http://www.enamine.net/index.php?option=com_content&task=view&id=11; downloaded 17/03/2016).

For the preparation of Supporting Figure 2, the following two libraries were used for comparison with the scaffolds:

a) The AnalytiCon MEGx Library (4460 natural product screening compounds from plants and microorganisms; http://www.ac-discovery.com/content/Products&Technologies/MEGAbolite.php; downloaded 17/03/2016).

b) The Enamine Advanced Collection (278,365 compounds; http://www.enamine.net/index.php?option=com_content&task=view&id=11; downloaded 17/03/2016).

4.1 Molecular properties and natural product-likeness scores of the fragments

Entry	Fragment No.	Fragment	Natural product-likeness score	AlogP	HA	RMM /Da
1	**F1**	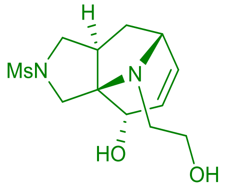	0.28	-0.93	19	288.363
2	**F2**	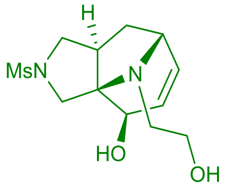	0.28	-0.93	19	288.363
3	**F3**	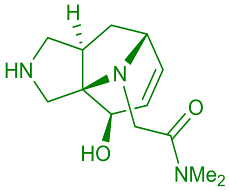	0.34	-0.82	18	251.325
4	**F4**	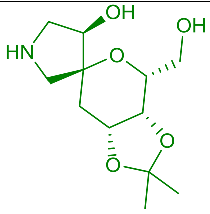	1.8	-0.89	18	259.299
5	**F5**	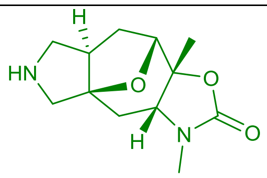	1.7	-0.09	17	238.283
6	**F6**	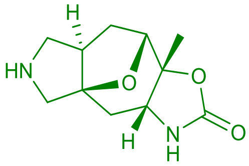	2.1	-0.41	16	224.256
7	**F7**	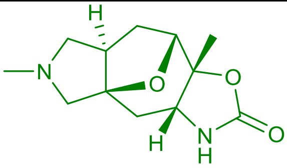	1.9	0.3	17	238.283
8	**F8**	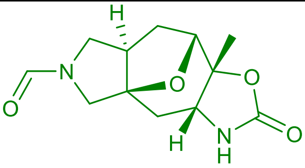	1.4	-0.14	18	252.266

Entry	Fragment No.	Fragment	Natural product-likeness score	AlogP	HA	RMM /Da
9	**F9**	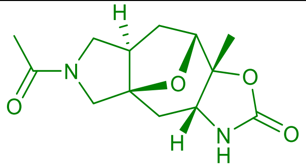	1.7	-0.26	19	266.293
10	**F10**	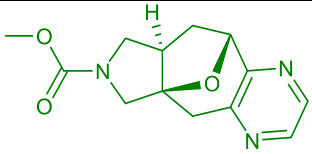	0.28	0.27	19	261.276
11	**F11**	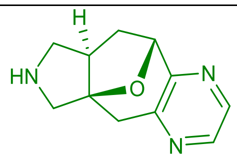	0.92	-0.03	15	203.24
12	**F12**	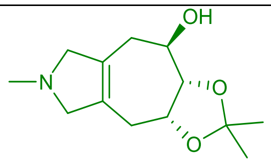	1.5	0.21	17	239.311
13	**F13**	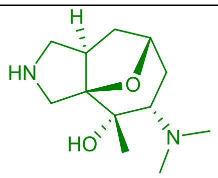	1.8	0.02	16	226.315
14	**F14**	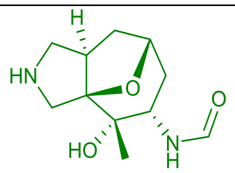	1.9	-0.96	16	226.272
15	**F15**	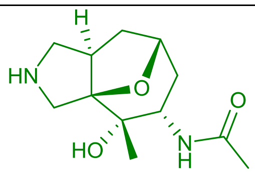	1.6	-0.68	17	240.299
16	**F16**	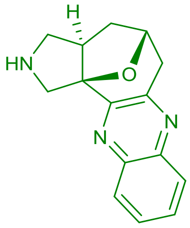	0.6	1.48	19	253.299
17	**F17**	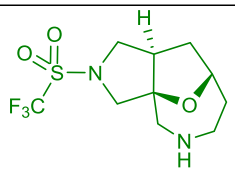	0.81	1.05	19	300.298
18	**F18**	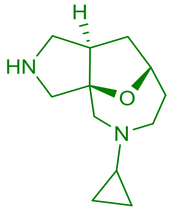	1.2	0.82	15	208.3

Entry	Fragment No.	Fragment	Natural product-likeness score	AlogP	HA	RMM /Da
19	**F19**	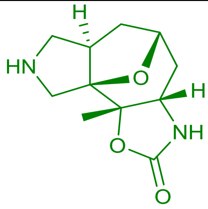	1.9	-0.21	16	224.256
20	**F20**	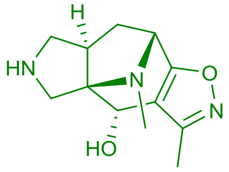	0.33	-0.31	17	235.282
21	**F21**	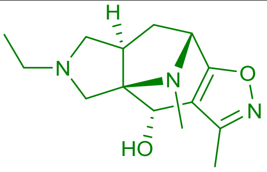	−0.067	0.96	19	263.335
22	**F22**	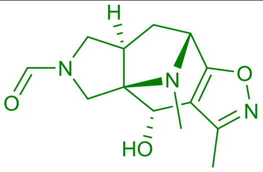	0.2	-0.47	19	263.292
23	**F23**	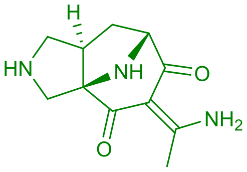	0.88	-1.33	16	221.256
24	**F24**	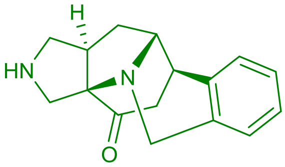	0.9	1.25	19	254.327
25	**F25**	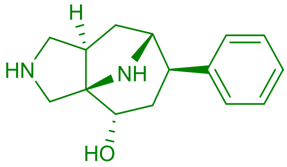	1.3	0.35	18	244.332
26	**F26**	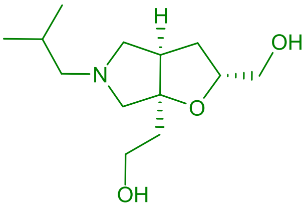	0.74	0.74	17	243.342
27	**F27**	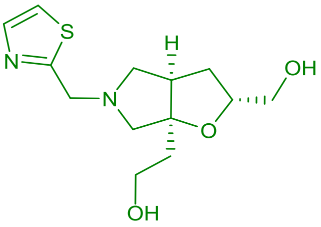	−0.2	0.44	19	284.375

Entry	Fragment No.	Fragment	Natural product-likeness score	AlogP	HA	RMM /Da
28	**F28**	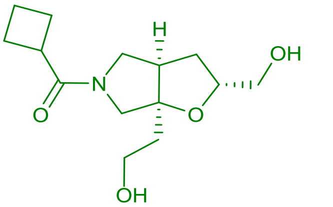	0.4	0.05	19	269.337
29	**F29**	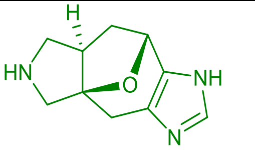	0.74	-0.16	14	191.23
30	**F30**	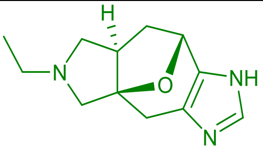	0.16	0.74	16	219.283
31	**F31**	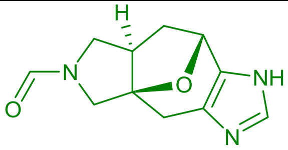	0.48	-0.7	16	219.24
32	**F32**	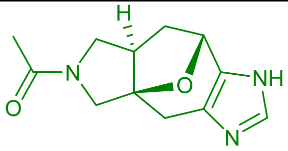	0.19	-0.28	17	233.266
33	**F33**	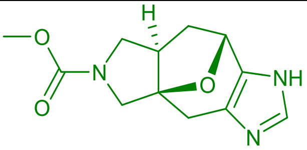	0.1	0.02	18	249.266
34	**F34**	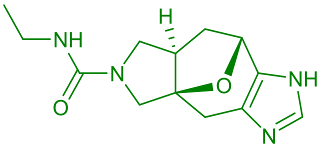	−0.42	0.01	19	262.308
35	**F35**	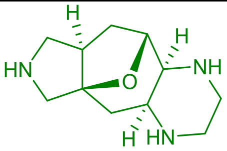	1.9	-1.27	15	209.288
36	**F36**	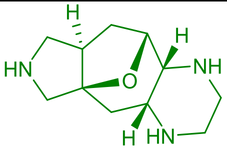	1.9	-1.27	15	209.288
37	**F37**	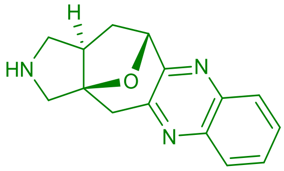	0.49	1.41	19	253.299
38	**F38**	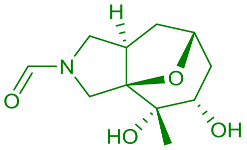	2.0	-1.35	16	227.257

Entry	Fragment No.	Fragment	Natural product-likeness score	AlogP	HA	RMM /Da
39	**F39**	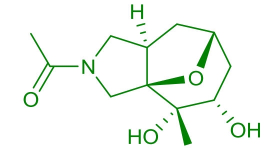	1.7	-0.79	17	241.284
40	**F40**	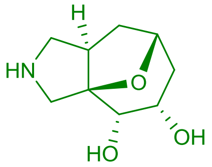	2.7	-1.24	13	185.22
41	**F41**	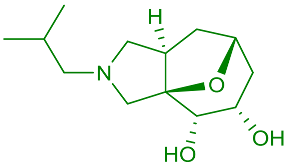	1.5	0.34	17	241.327
42	**F42**	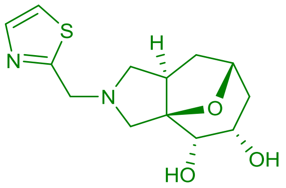	0.47	0	19	282.359
43	**F43**	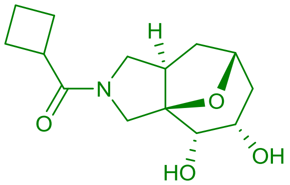	1.1	-0.14	19	267.321
44	**F44**	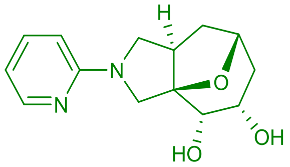	0.69	0.1	19	262.304
45	**F45**	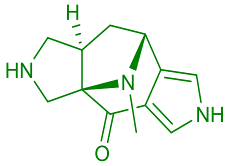	0.54	0.14	16	217.267
46	**F46**	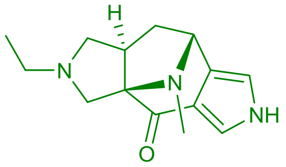	0.032	1.35	18	245.32
47	**F47**	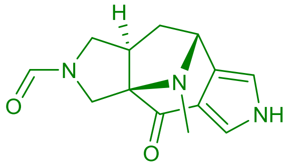	0.32	-0.56	18	245.277
48	**F48**	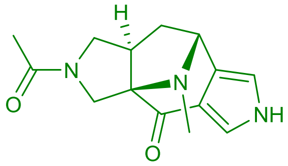	0.066	0.35	19	259.304

Entry	Fragment No.	Fragment	Natural product-likeness score	AlogP	HA	RMM /Da
49	**F49**	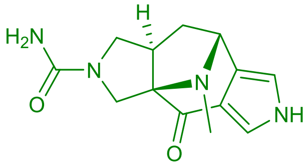	−0.0083	0.02	19	260.292
50	**F50**	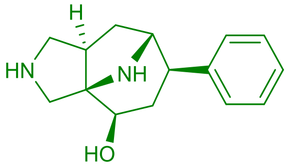	1.3	0.35	18	244.332
51	**F51**	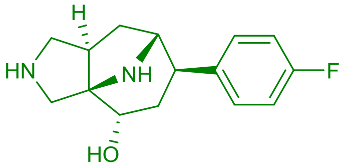	0.8	0.35	19	262.323
52	**F52**	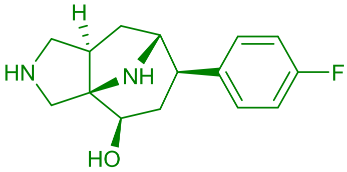	0.8	0.35	19	262.323

4.2 Natural product-likeness scores of the deprotected scaffolds

Entry	Scaffold No.	Scaffold (deprotected)	Natural product-likeness score
1	**3**	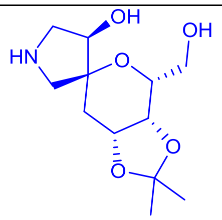	1.8
2	**4**	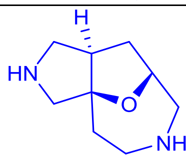	1.6
3	**5**	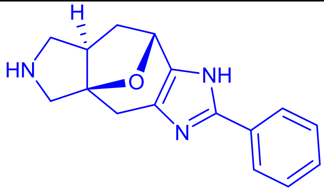	0.39
4	**6**	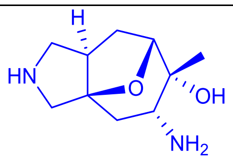	2.1
5	**7**	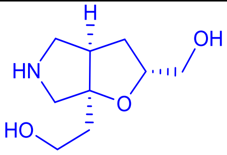	1.7
6	**8**	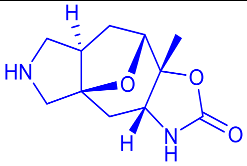	2.1
7	**9**	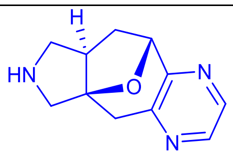	0.92
8	**10**	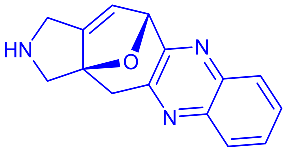	0.33
9	**11**	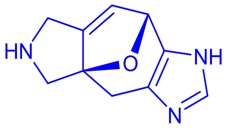	0.52
10	**12**	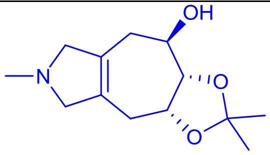	1.5

11	**13**	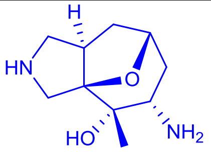	2.0
12	**14**	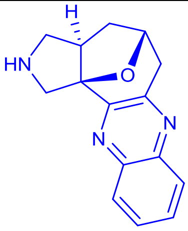	0.6
13	**15**	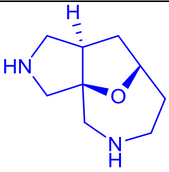	2.0
14	**16**	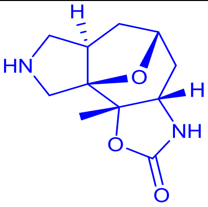	1.9
15	**17**	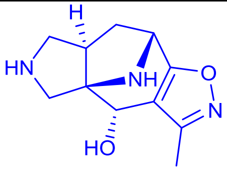	0.33
16	**18**	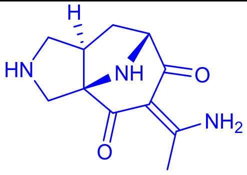	0.88
17	**19**	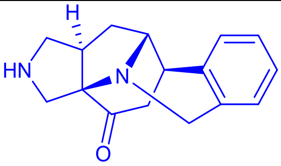	0.9
18	**20/S33**	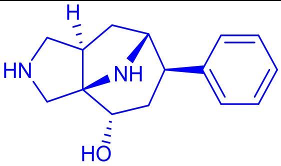	1.3
19	**S13**	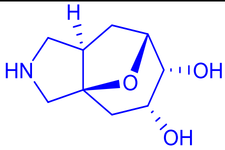	2.7
20	**S16**	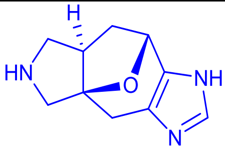	0.74

21	**S19**	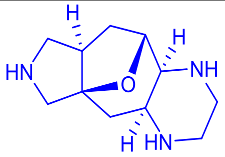	1.9
22	**S20**	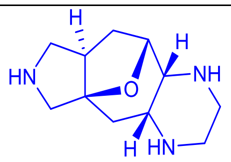	1.9
23	**S21**	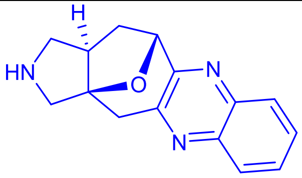	0.49
24	**S24**	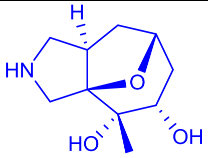	2.5
25	**S25**	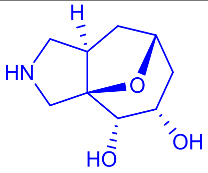	2.7
26	**S27**	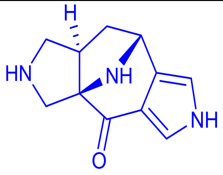	0.54
27	**S32/S34**	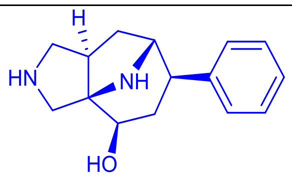	1.3
28	**S35**	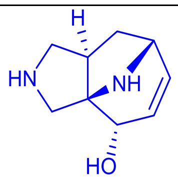	1.6

4.3 Summary of natural product-likeness scores of the fragments and scaffolds

After scoring the libraries, the data was sorted in bins of 0.5 increments (i.e. −5 ≤ NP < −4.5, −4.5 ≤ NP < −4, etc.). Below are the tables of the binned results for each library.

Bin	Fragments		Scaffolds	
	Frequency	Fraction	Frequency	Fraction
−5 ≤ NP < −4.5	0	0	0	0
−4.5 ≤ NP < −4	0	0	0	0
−4 ≤ NP < −3.5	0	0	0	0
−3.5 ≤ NP < −3	0	0	0	0
−3 ≤ NP < −2.5	0	0	0	0
−2.5 ≤ NP < −2	0	0	0	0
−2 ≤ NP < −1.5	0	0	0	0
−1.5 ≤ NP < −1	0	0	0	0
−1 ≤ NP < −0.5	0	0	0	0
−0.5 ≤ NP < −0	0	0	0	0
0 ≤ NP < 0.5	3	0.074074	0	0
0.5 ≤ NP < 1	17	0.314815	4	0.133333
1 ≤ NP < 1.5	11	0.203704	7	0.233333
1.5 ≤ NP < 2	6	0.12963	2	0.1
2 ≤ NP < 2.5	13	0.240741	10	0.333333
2.5 ≤ NP < 3	1	0.018519	2	0.1
3 ≤ NP < 3.5	1	0.018519	3	0.1
3.5 ≤ NP < 4	0	0	0	0
4 ≤ NP < 4.5	0	0	0	0
4.5 ≤ NP < 5	0	0	0	0
**SUM**	**52**	**1**	**28**	**1**

4.4 Natural product-likeness scores of commercial libraries

Bin	BIONET – Fragments from Nature	
	Frequency	Fraction
−5 ≤ NP < −4.5	0	0
−4.5 ≤ NP < −4	0	0
−4 ≤ NP < −3.5	0	0
−3.5 ≤ NP < −3	0	0
−3 ≤ NP < −2.5	0	0
−2.5 ≤ NP < −2	0	0
−2 ≤ NP < −1.5	0	0
−1.5 ≤ NP < −1	3	0.023438
−1 ≤ NP < −0.5	21	0.164063
−0.5 ≤ NP < −0	33	0.257813
0 ≤ NP < 0.5	30	0.234375
0.5 ≤ NP < 1	22	0.171875
1 ≤ NP < 1.5	13	0.101563
1.5 ≤ NP < 2	6	0.046875
2 ≤ NP < 2.5	0	0

Bin	BIONET – Fragments from Nature	
	Frequency	Fraction
2.5 ≤ NP < 3	0	0
3 ≤ NP < 3.5	0	0
3.5 ≤ NP < 4	0	0
4 ≤ NP < 4.5	0	0
4.5 ≤ NP < 5	0	0
**SUM**	**128**	**1**

Bin	Enamine General Fragment Library	
	Frequency	Fraction
−5 ≤ NP < −4.5	0	0
−4.5 ≤ NP < −4	0	0
−4 ≤ NP < −3.5	0	0
−3.5 ≤ NP < −3	11	0.000881
−3 ≤ NP < −2.5	166	0.013295
−2.5 ≤ NP < −2	942	0.075444
−2 ≤ NP < −1.5	2213	0.177239
−1.5 ≤ NP < −1	3086	0.247157
−1 ≤ NP < −0.5	2394	0.191735
−0.5 ≤ NP < −0	1836	0.147045
0 ≤ NP < 0.5	1076	0.086177
0.5 ≤ NP < 1	564	0.045171
1 ≤ NP < 1.5	176	0.014096
1.5 ≤ NP < 2	18	0.001442
2 ≤ NP < 2.5	4	0.00032
2.5 ≤ NP < 3	0	0
3 ≤ NP < 3.5	0	0
3.5 ≤ NP < 4	0	0
4 ≤ NP < 4.5	0	0
4.5 ≤ NP < 5	0	0
**SUM**	**12486**	**1**

Bin	AnalytiCon MEGx Library	
	Frequency	Fraction
−5 ≤ NP < −4.5	0	0
−4.5 ≤ NP < −4	0	0
−4 ≤ NP < −3.5	0	0
−3.5 ≤ NP < −3	0	0
−3 ≤ NP < −2.5	0	0
−2.5 ≤ NP < −2	0	0
−2 ≤ NP < −1.5	0	0
−1.5 ≤ NP < −1	1	0.000224
−1 ≤ NP < −0.5	1	0.000224
−0.5 ≤ NP < −0	14	0.003139
0 ≤ NP < 0.5	53	0.011883
0.5 ≤ NP < 1	174	0.039013
1 ≤ NP < 1.5	424	0.095067
1.5 ≤ NP < 2	688	0.15426
2 ≤ NP < 2.5	1111	0.249103

Bin	AnalytiCon MEGx Library	
	Frequency	Fraction
2.5 ≤ NP < 3	923	0.206951
3 ≤ NP < 3.5	645	0.144619
3.5 ≤ NP < 4	372	0.083408
4 ≤ NP < 4.5	54	0.012108
4.5 ≤ NP < 5	0	0
**SUM**	**4460**	**1**

Bin	Enamine Advanced Collection	
	Frequency	Fraction
−5 ≤ NP < −4.5	0	0
−4.5 ≤ NP < −4	0	0
−4 ≤ NP < −3.5	14	5.02937E-05
−3.5 ≤ NP < −3	264	0.000948395
−3 ≤ NP < −2.5	3039	0.010917321
−2.5 ≤ NP < −2	19398	0.069685485
−2 ≤ NP < −1.5	57552	0.20675013
−1.5 ≤ NP < −1	85558	0.307359043
−1 ≤ NP < −0.5	68775	0.247067699
−0.5 ≤ NP < −0	31687	0.113832558
0 ≤ NP < 0.5	9482	0.03406319
0.5 ≤ NP < 1	2057	0.007389578
1 ≤ NP < 1.5	421	0.001512403
1.5 ≤ NP < 2	82	0.000294577
2 ≤ NP < 2.5	26	9.34025E-05
2.5 ≤ NP < 3	7	2.51468E-05
3 ≤ NP < 3.5	2	7.18481E-06
3.5 ≤ NP < 4	0	0
4 ≤ NP < 4.5	1	3.59241E-06
4.5 ≤ NP < 5	0	0
**SUM**	**278365**	**1**

5.0 Experimental

5.1 General experimental

All non-aqueous reactions were performed under an atmosphere of nitrogen unless otherwise stated. Water-sensitive reactions were performed in oven-dried glassware, cooled under nitrogen before use. THF, CH_2Cl_2, PhMe and MeCN were dried and purified by means of a Pure Solv MD solvent purification system (Innovative Technology Inc.). Anhydrous DMF was obtained in a SureSeal bottle from Sigma-Aldrich. All other solvents used were of chromatography or analytical grade. Petrol refers to petroleum spirit (b.p. 40-60 °C). Commercially available starting materials were obtained from Sigma-Aldrich, Fluka, Acros, Alfa Aesar or Fluorochem and were used without purification.

Thin layer chromatography (TLC) was carried out on aluminium backed silica plates (Merck silica gel 60 F254). Visualisation of the plates was achieved using an ultraviolet lamp (λ_{max} = 254 nm) and $KMnO_4$. Flash chromatography was carried out using silica gel 60 (60-63 μm particles) supplied by Merck. Columns with solvent gradients were carried out using a Biotage Flashmaster II on pre-packed Redisep normal-phase silica or cyanosilica cartridges (as specified). Strong cation exchange solid phase extraction (SCX SPE) was carried out using pre-packed Discovery DSC-SCX cartridges supplied by Supelco, see the general procedure below.

Melting points were measured on a Reichert hot stage apparatus and are uncorrected. Infrared spectra were recorded on a Bruker Alpha Platinum-ATR, with absorption reported in wavenumbers (cm^{-1}). High resolution mass spectra (HRMS) were recorded on a Bruker MaXis Impact spectrometer with electrospray ionisation (ESI) source. Low resolution mass spectra (LRMS) were recorded by HP-LCMS, which was generally carried out on an Agilent 1200 series LC system comprising a Bruker HCT Ultra ion trap mass spectrometer. The solvent system used was CH_3CN/H_2O + 0.1% formic acid with a Phenomenex Luna C18 50 × 2 mm 5 micron column.

Proton (^1H) and carbon (^{13}C) NMR spectral data were collected on Bruker Advance 500, Bruker DPX500, Bruker Advance 400 and Bruker DPX300 spectrometers. Chemical shifts (δ) are quoted in parts per million (ppm) and referenced to the residual solvent peak. Coupling constants (*J*) are quoted in Hertz (Hz) and splitting patterns reported in an abbreviated manner: app. (apparent), s (singlet), d (doublet), t (triplet), q (quartet), m (multiplet). All fully characterised products were assigned with the aid of COSY, DEPT-135 and HMQC experiments. Where stated HMBC and NOESY experiments were also used to aid assignments. Compounds are numbered with respect to their IUPAC names. Where necessary, coloured text was used to distinguish similar protons and carbons. Diastereomeric ratios were calculated by analysis of the ^1H NMR spectra and

diastereomers were assigned through the interpretation of coupling constants, NOESY spectra, and through small molecule crystallographic studies. Small molecule X-ray crystallography studies were performed by Dr Christopher Pask.

5.2 General procedures

General procedure A: Strong cation exchange solid phase extraction (SCX SPE)

TfOH (0.5 M in MeOH, 10 mL / 5 g SCX SPE) was dripped through the SCX SPE cartridge prior to use. MeOH (20 mL) was then flushed through using pressurised air. The crude residue (3.5 mmol / 5 g SCX SPE silica) was loaded in the minimum amount of MeOH. The cartridge was flushed with MeOH and the fractions were collected and monitored by TLC. The cartridge was then flushed with sat. NH_3/MeOH and the fractions were collected and monitored by TLC. Fractions containing product were combined and concentrated.

General Procedure B: Hydrogenation catalysed by Pd/C or Pd(OH_2)/C

The substrate (1.0 eq.) was dissolved in MeOH or EtOH (~20 mL g^{-1}) and added *via* syringe to a round-bottomed flask containing 10 wt% Pd/C (% w/w as specified) or 20 wt% $Pd(OH)_2$/C (% w/w as specified) pre-submerged in minimal solvent (MeOH or EtOH) under N_2. If required, conc. HCl (~12 M) was added as specified. The head space of the flask was exposed to a sequence of vacuum/H_2 flushes (×3), then exposed to an atmosphere of H_2 (balloon). The reaction was monitored by TLC until complete. At this point the balloon was removed and the reaction mixture was fitted with a gas outlet (wide bore syringe needle) and purged with N_2 for 5 minutes. The reaction mixture was filtered through Celite eluting with MeOH, then concentrated *in vacuo*. The product was typically used in the next step without further purification.

General procedure C: Reductive amination

$NaBH(OAc)_3$ (3.0 eq.) was added to a stirred solution of amine (1.0 eq.) and aldehyde (2.5-5.0 eq., as specified) in CH_2Cl_2 (0.2 M). The reaction mixture was stirred at rt (unless otherwise specified) for 15 h, then flushed through a pad of Celite eluting with CH_2Cl_2 and concentrated *in vacuo*. Products were purified by flash chromatography.

General procedure D: *N*-Formylation

Ac_2O (10-30 eq., as specified) was added to a stirred solution of amine (1.0 eq.) in 30:70 CH_2Cl_2–HCO_2H acid (0.1 M). The reaction mixture was stirred at rt for 1 h, then concentrated *in vacuo*. Products were purified by flash chromatography.

General procedure E: *N*-Acylation

Ac_2O (2.0 eq.) was added to a stirred solution of substrate (1.0 eq.) and Et_3N (3.0 eq.) in CH_2Cl_2 (0.2 M) at 0 °C. The reaction mixture was warmed to rt, stirred for 1 h, then concentrated *in vacuo*. Products were purified by flash chromatography.

General procedure F: Methyl carbamate formation

Methyl chloroformate (10 eq.) was added to a stirred solution of amine (1.0 eq.) and Et_3N (10 eq.) in CH_2Cl_2 (0.1 M) at 0 °C. The reaction mixture was warmed to rt and stirred for 1 h. The reaction mixture was concentrated *in vacuo*. Products were purified by SCX SPE or flash chromatography.

General procedure G: *O*-Silyl deprotection using (±)-camphorsulfonic acid

(±)-Camphorsulfonic acid (4.0-10 eq., as specified) was added to a stirred suspension of amine (1.0 eq.) in MeOH (0.3 M). The reaction mixture heated at 45 °C for 15 h, then concentrated *in* vacuo. Products were purified by flash chromatography.

General procedure H: *O*-Silyl deprotection using TBAF

TBAF (1.0 M in THF, 2.0 eq.) was added a stirred solution of substrate (1.0 eq.) in THF (0.1 M). The reaction mixture was stirred for 0.5 h, then concentrated *in vacuo*. Products were purified by flash chromatography.

General procedure I: Deprotection of *tert*-butylcarbamoyl (Boc) amines

To a solution of Boc-amine (1.0 eq.) in CH_2Cl_2 (0.5 M) was added TFA (1 volume) and the resulting solution was stirred at rt for 1 h then concentrated *in vacuo*. Products were purified by SCX SPE eluting with MeOH, then sat. NH_3/MeOH.

5.3 A note on NMR assignments

Where compounds have been assigned through analysis of the corresponding NOESY spectrum, protons labelled 'A' are on the 'bottom' face of the molecules (as drawn), while protons labelled 'B' are on the 'top' face of the molecules (as drawn), see compound **S13** below as an example.

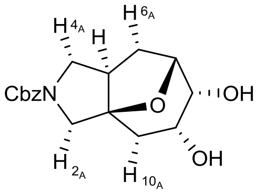

S13

'A' protons

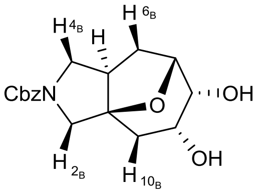

S13

'B' protons

Where the polycyclic assemblies **were not** assigned using NOESY the 'A' and 'B' descriptors are reported through analysis of the coupling constants or otherwise arbitrarily.

5.4 Compound data

5.4.1 Preparation of cycloaddition precursors and cycloadducts

5.4.1.1 Preparation of each intermediate in the synthesis of *O*-bridged cycloadduct 2a

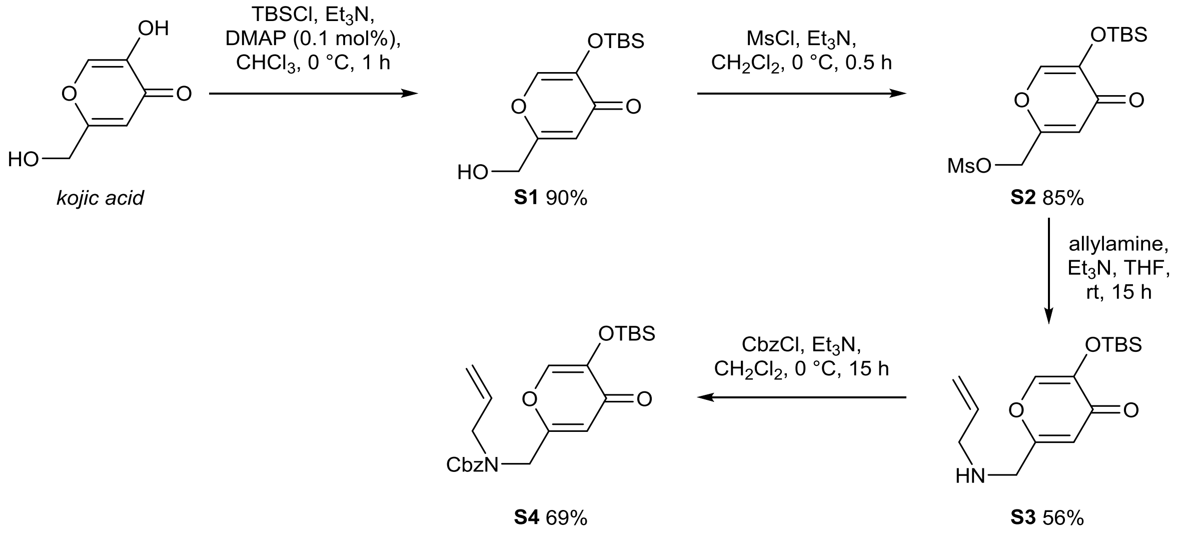

5-[(*tert*-Butyldimethylsilyl)oxy]-2-(hydroxymethyl)-4H-pyran-4-one S1

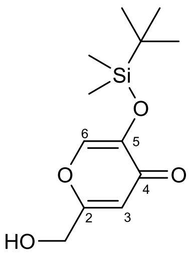

S1

Following a procedure by Miyazaki,[S7] TBSCl (5.3 g, 35 mmol, 1.0 eq.) was added to a stirred suspension of kojic acid (5.0 g, 35 mmol, 1.0 eq.), Et_3N (7.4 mL, 100 mmol, 2.90 eq.) and DMAP (5 mg, 0.04 mmol, 0.001 eq.) in $CHCl_3$ (50 mL) at 0 °C. The reaction mixture was stirred at this temperature for 1 h then aqueous $KHSO_4$ (5 wt%, 50 mL) was added. The phases were separated and the organic phase was washed with brine (50 mL), dried, filtered and concentrated *in vacuo*. Flash chromatography eluting with 1:1 pentane–EtOAc gave the title compound **S1** (8.1 g, 32 mmol, 90%) as a colourless amorphous solid.* **R_f** 0.57 (1:1 petrol–EtOAc). **^{1}H NMR** (500 MHz, $CDCl_3$): δ 7.65 (1H, s, 6-H), 6.47 (1H, s, 3-H), 4.46 (2H, d, *J* 6.3, CH_2OH), 3.13 (1H, t, *J* 6.3, O*H*), 0.95 (9H, s, $SiC(CH_3)_3$), 0.21 (6H, s, 2 × $SiCH_3$). **^{13}C NMR** (125 MHz, $CDCl_3$): δ 176.1 (4-C), 166.6 (2-C), 144.6 (5-C), 144.2 (6-C), 112.4 (3-C), 61.1 (CH_2OH), 25.8 (SiC(CH_3)$_3$), 18.7 (SiC_q), −4.4 (2 × $SiCH_3$). **IR** ν_{max}(film)/cm^{-1} 3358 (br., OH), 2954, 2857, 1651 (CO), 1629, 1268, 1211, 874. **LRMS** (HPLC-MS): $C_{12}H_{21}O_4Si$; found 257.1 $[M+H]^+$. Spectral data are consistent with the literature values.[S8]

{5-[(*tert*-Butyldimethylsilyl)oxy]-4-oxo-4H-pyran-2-yl}methyl methanesulfonate S2

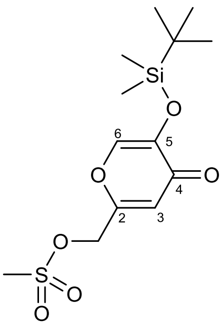

S2

Et_3N (3.30 mL, 23.4 mmol, 2.00 eq.) was added to a stirred solution of silyl protected kojic acid **S1** (3.00 g, 11.7 mmol, 1.00 eq.) in CH_2Cl_2 (24 mL). The reaction mixture was cooled to 0 °C, then methanesulfonyl chloride (1.1 mL, 14 mmol, 1.2 eq.) was added dropwise. The reaction mixture was stirred at 0 °C for 0.5 h, then warmed to rt and partitioned with H_2O (25 mL). The phases were separated and the aqueous phase was extracted with CH_2Cl_2 (25 mL). The combined organic phases were dried over $MgSO_4$, filtered and concentrated *in vacuo* to give the *title compound* **S2** (3.31 g, 9.89 mmol, 85% mass recovery) which was used subsequently without further purification. **R_f** 0.62 (1:1 petrol–EtOAc). **^{1}H NMR** (300 MHz, $CDCl_3$, characteristic peaks): δ 7.69 (1H, s, 6-H), 6.48 (1H, s, 3-H), 4.97 (2H, s, CH_2), 3.11 (3H, s, SO_2CH_3), 0.95 (9H, s, $SiC(CH_3)_3$), 0.23 (6H, s, 2 × $SiCH_3$).

---

* Compound **S1** and related silylated pyranone derivatives **S2-S6** slowly decomposed on standing in air or in mildly acidic solvents (e.g. $CDCl_3$). Compounds of this type should be stored in a freezer at −18 °C. N.b. derived cycloadducts **2a-c** were bench stable at rt for several weeks.

5-[(*tert*-Butyldimethylsilyl)oxy]-2-{[(prop-2-en-1-yl)amino]methyl}-4*H*-pyran-4-one S3

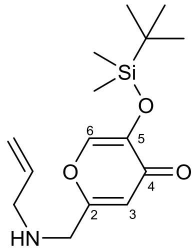

S3

Et_3N (3.5 mL, 35 mmol, 1.0 eq.) was added to a stirred solution of mesylate **S2** (11.8 g, 35 mmol, 1.0 eq.) in THF (120 mL). Allylamine (8.0 mL, 106 mmol, 3.0 eq.) was added and the reaction mixture was stirred for 15 h, then concentrated *in vacuo*. The resulting residue was diluted in EtOAc (50 mL) and washed with sat. aq. $NaHCO_3$ solution (50 mL). The phases were separated and the aqueous phase was extracted with EtOAc (50 mL). The combined organics were washed with brine (50 mL), dried over $MgSO_4$, filtered and concentrated *in vacuo*. The residue was washed through a pad of silica with 9:1 EtOAc–MeOH to give the *title compound* **S3** (5.9 g, 20.0 mmol, 56%) as a dark brown oil. **R_f** 0.57 (1:1 petrol–EtOAc). **^{1}H NMR** (500 MHz, $CDCl_3$): δ 7.64 (1H, s, 6-H), 6.36 (1H, s, 3-H), 5.86 (1H, ddt, *J* 16.8, 10.3, 6.0, C*H*=CH_2), 5.20 (1H, app. dq, *J* 16.8, 1.4, CH=CH_AH_B), 5.14 (1H, ddd, *J* 10.3, 2.7, 1.4, C*H*=CH_AH_B), 3.62 (2H, s, C_qCH_2NH), 3.27 (2H, dt, *J* 6.0, 1.4, NHCH_2CH=CH_2), 0.96 (9H, s, $SiC(CH_3)_3$), 0.23 (6H, s, 2 × $SiCH_3$). **^{13}C NMR** (125 MHz, $CDCl_3$): δ 175.7 (4-C), 165.7 (2-C), 145.5 (5-C), 144.2 (6-C), 135.9 (*C*H=CH_2), 117.1 (CH=*C*H_2), 113.7 (3-C), 51.5 (*C*H_2CH=CH_2), 49.8 (C_q*C*H_2NH), 25.8 (SiC(*C*H_3)$_3$), 18.7 (SiC_q), −4.3 (2 × $SiCH_3$). **IR** ν_{max}(film)/cm^{-1} 2954, 2930, 2857, 1651 (CO), 1232, 919, 879, 786. **LRMS** (HPLC-MS): $C_{15}H_{25}NO_3Si$; found 296.1 $[M+H]^+$.

Benzyl *N*-({5-[(*tert*-butyldimethylsilyl)oxy]-4-oxo-4*H*-pyran-2-yl}methyl)-*N*- (prop-2-en-1-yl)carbamate S4

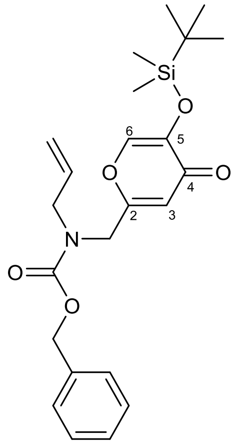

S4

Benzyl chloroformate (180 μL, 1.28 mmol, 2.6 eq.) was added to a stirred solution of the amine **S3** (145 mg, 0.49 mmol, 1.0 eq.) and Et_3N (180 μL, 1.28 mmol, 2.6 eq.) in CH_2Cl_2 (5.0 mL) at 0 °C. The reaction mixture warmed to rt and stirred for 15 h, then concentrated *in vacuo*. Flash chromatography eluting with 9:1 EtOAc–MeOH gave the *title compound* **S4** (145 mg, 0.34 mmol, 69%) as a pale yellow oil. **R_f** 0.82 (1:1 petrol–EtOAc). **See Section 5.4.1.2 for the spectral data and optimised route to prepare this compound.**

5.4.1.2 Telescoped synthesis of *O*-bridged cycloadducts 2a-b

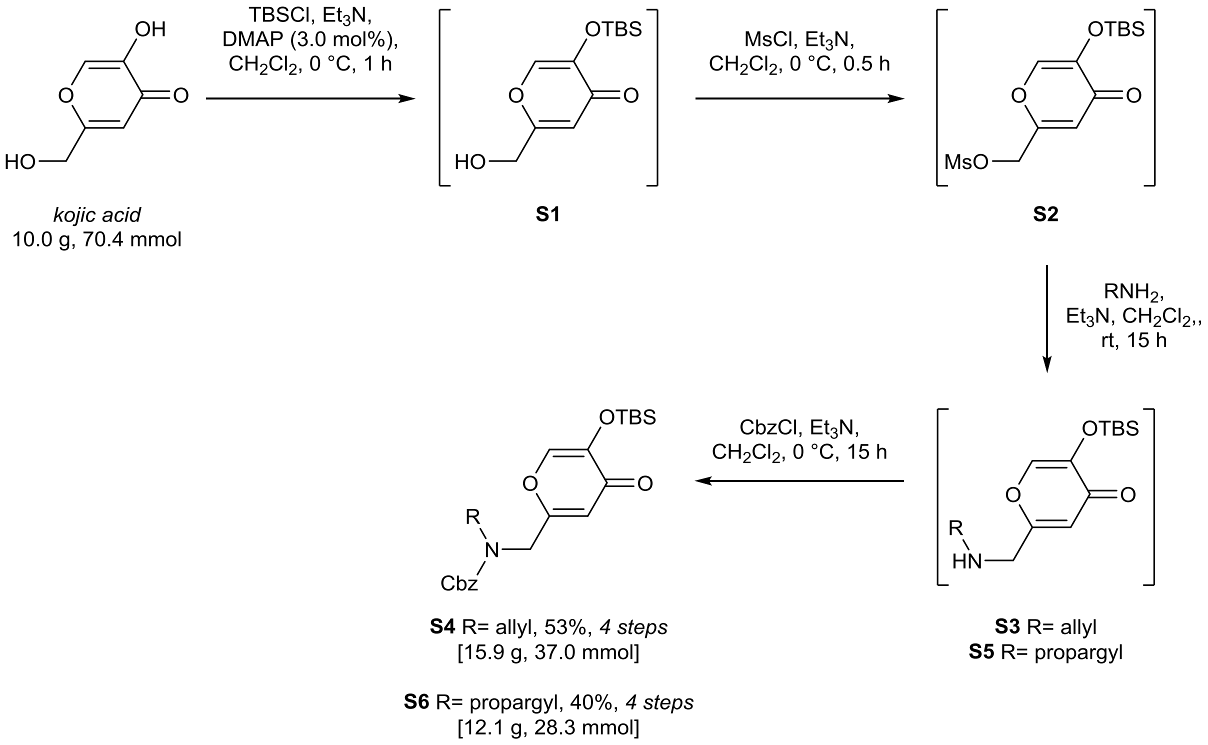

General procedure J: Four-step telescoped procedure to prepare cycloaddition precursors S4 and S6

TBSCl (10.7 g, 71.0 mmol, 1.01 eq.) was added to a stirred solution of kojic acid (10.0 g, 70.4 mmol, 1.00 eq.), Et_3N (10.8 mL, 77.9 mmol, 1.10 eq.) and DMAP (258 mg, 2.11 mmol, 0.03 eq.) in CH_2Cl_2 (150 mL) at 0 °C. The reaction mixture was warmed to rt and stirred for 45 min. The reaction mixture was quenched with sat. aq. NH_4Cl solution (100 mL) and H_2O (100 mL). After separation, the aqueous phase was extracted with CH_2Cl_2 (2 × 150 mL). The organic extracts were combined, dried over Na_2SO_4 and concentrated *in vacuo*. To the residue **S1** (70.4 mmol) in CH_2Cl_2 (150 mL) at 0 °C was added Et_3N (12.0 mL, 86.5 mmol, 1.23 eq.) and methanesulfonyl chloride (6.0 mL, 78 mmol, 1.1 eq.) dropwise. The reaction mixture was warmed to rt and stirred for 0.5 h, then quenched with water (150 mL). After phase separation, the aqueous phase was extracted using CH_2Cl_2 (2 × 150 mL). The organic extracts were combined, dried over Na_2SO_4 and concentrated *in vacuo*. To the residue **S2** (70.4 mmol) in CH_2Cl_2 (150 mL) at 0 °C was added Et_3N (10.0 mL, 72.0 mmol, 1.02 eq.) and amine (as specified below, 3.80 eq.). The reaction mixture warmed to rt, stirred for 15 h, then quenched with H_2O (150 mL). After phase separation, the aqueous phase was extracted using CH_2Cl_2 (2 × 150 mL). The organic extracts were combined, dried over Na_2SO_4 and concentrated *in vacuo*. To the residue **S3** or **S5** (70.4 mmol) in CH_2Cl_2 (150 mL) at 0 °C was added Et_3N (17.0 mL, 121 mmol, [1.72 eq.]) followed by the very slow addition (*exothermic – gas outlet*

necessary!) of benzyl chloroformate (15.0 mL, 106 mmol, 1.50 eq.). The reaction mixture warmed to rt and stirred 2 h. The reaction mixture was quenched with sat. aq. NH_4Cl solution (150 mL) and H_2O (150 mL). After phase separation, the aqueous phase was extracted with CH_2Cl_2 (2 × 150 mL). The organic extracts were combined, dried over Na_2SO_4 and concentrated *in vacuo*.

Benzyl *N*-({5-[(*tert*-butyldimethylsilyl)oxy]-4-oxo-4*H*-pyran-2-yl}methyl)-*N*- (prop-2-en-1-yl)carbamate S4

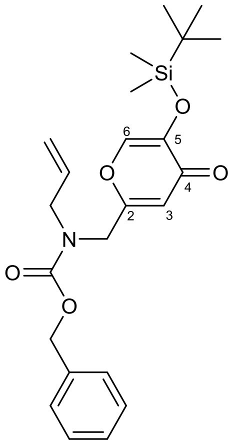

S4

General procedure **J** was followed using kojic acid (10.0 g, 70.4 mmol, 1.00 eq) and allylamine (20.0 mL, 267 mmol, 3.80 eq.). Flash chromatography eluting with 9:1 to 8:2 pentane–EtOAc gave the *title compound* **S4** (15.9 g, 37.0 mmol, 53%, 4 steps) as a pale yellow oil. **R_f** 0.82 (1:1 petrol–EtOAc). **^{1}H NMR** (500 MHz, $CDCl_3$, 330 K): δ 7.56 (1H, s, 6-H), 7.39-7.27 (5H, m, Cbz Ar-H), 6.23 (1H, s, 3-H), 5.81-5.70 (1H, m, C*H*=CH_2), 5.21-5.10 (4H, m, CH=C*H*$_2$ and OC*H*$_2$Ph), 4.26 (2H, s, C_qC*H*$_2$N), 3.96 (2H, s, NC*H*$_2$CH=CH_2), 0.97 (9H, s, SiC(CH_3)$_3$), 0.24 (6H, s, 2 × $SiCH_3$). **^{13}C NMR** (125 MHz, $CDCl_3$, 330 K): δ 175.3 (4-C), 163.3 (2-C), 156.1 (N(CO)O), 145.8 (5-C), 144.0 (6-C), 136.5 (*C*H=CH_2), 132.9 (Ar-C_q), 128.7 (Ar-C), 128.4 (Ar-C), 128.2 (Ar-C), 118.2 (CH=*C*H$_2$), 113.7 (3-C), 65.6 (O*C*H$_2$Ph), 50.3 (*C*H$_2$CH=CH_2), 47.6 (C_q*C*H$_2$NH), 25.8 (SiC(*C*H$_3$)$_3$), 18.7 (SiC_q), −4.3 (2 × $SiCH_3$). **IR** ν_{max}(film)/cm^{-1} 2953, 2929, 2857, 1702 (CO), 1649, 1460, 1410, 1210. **HRMS** (ESI): $C_{23}H_{32}NO_5Si$ [M+H]$^+$; calculated 430.2058, found 430.2044.

Benzyl *N*-({5-[(tert-butyldimethylsilyl)oxy]-4-oxo-4*H*-pyran-2-yl}methyl)-N-(prop-2-yn-1-yl)carbamate S6

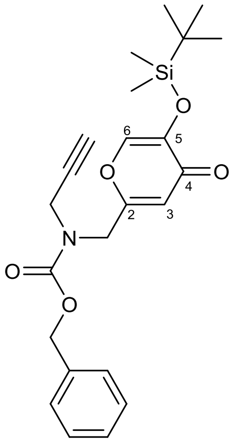

S6

General procedure **J** was followed using kojic acid (10.0 g, 70.4 mmol, 1.00 eq) and propargylamine (17.0 mL, 267 mmol, 3.80 eq.).* Flash chromatography eluting with 9:1 to 8:2 pentane–EtOAc gave the *title compound* **S6** (12.1 g, 28.3 mmol, 40%, 4 steps) as a pale yellow oil. **R_f** 0.20 (4:1 petrol–EtOAc). **^{1}H NMR** (500 MHz, $CDCl_3$, 329 K): δ 7.57 (1H, s, 6-H), 7.38-7.29 (5H, m, Cbz Ar-H), 6.28 (1H, s, 3-H), 5.19 (2H, s, OC*H*$_2$Ph), 4.42 (2H, s, C_qC*H*$_2$N), 4.18 (2H, br. s, NC*H*$_2$C≡CH), 2.26 (1H, t, *J* 2.5, C≡CH), 0.97 (9H, s, SiC(CH_3)$_3$), 0.24 (6H, s, 2 × $SiCH_3$). **^{13}C NMR** (125 MHz, $CDCl_3$, 329 K): δ 175.3 (4-C), 162.7 (2-C), 155.5 (N(CO)O), 145.9 (5-C), 144.0 (6-C), 136.2 (Ar-C_q),

* Crude amine **S5** characteristic ^{1}H NMR peaks (300 MHz, $CDCl_3$): δ 7.65 (1H, s, 6-H), 6.39 (1H, s, 3-H), 3.73 (2H, s, C_qC*H*$_2$NH), 3.47 (2H, d, *J* 2.4, NHC*H*$_2$C≡CH), 2.27 (1H, t, *J* 2.4, C≡CH), 0.95 (9H, s, SiC(CH_3)$_3$), 0.23 (6H, s, 2 × $SiCH_3$).

128.8 (Ar-C), 128.5 (Ar-C), 128.3 (Ar-C), 114.0 (3-C), 78.1 (CH_2*C*≡CH), 73.3 (CH_2C≡*C*H), 68.5 (O*C*H_2Ph), 47.5 (C_q*C*H_2N), 37.2 (*C*H_2C≡CH), 25.9 (SiC(*C*H_3)$_3$), 18.7 (SiC_q), −4.3 (2 × SiCH_3). **IR** ν_{max}(film)/cm^{-1} 2953, 2930, 2857, 1708 (CO), 1650, 1498, 1455, 1216. **HRMS** (ESI): $C_{23}H_{30}NO_5Si$ [M+H]$^+$; calculated 428.1888, found 428.1889.

5.4.1.3 Preparation of *O*-bridged cycloadducts 2a and 2b

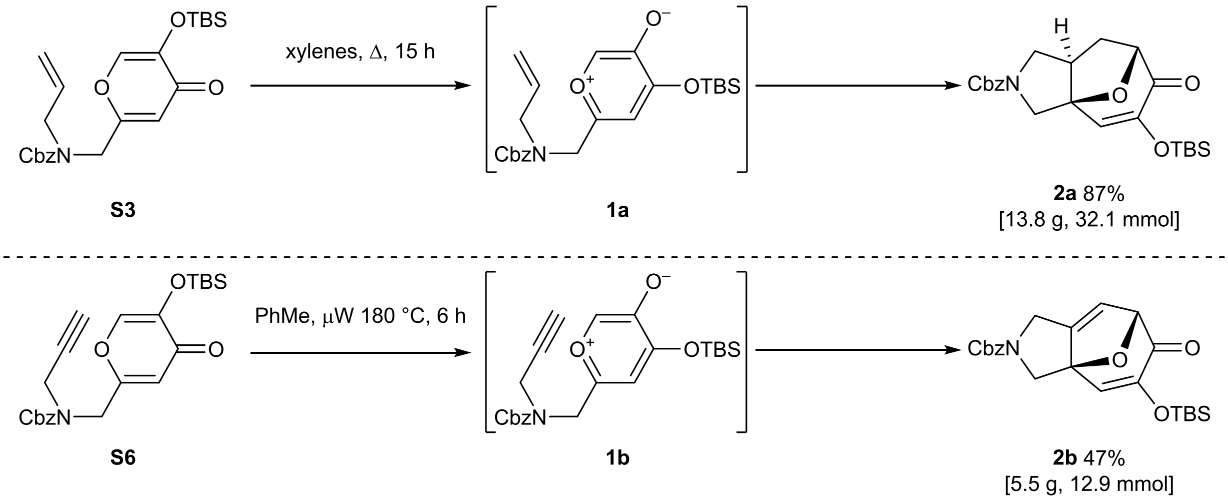

Benzyl (*1R**,*5S**,*7S**)-9-[(tert-butyldimethylsilyl)oxy]-8-oxo-11-oxa-3-zatricyclo[5.3.1.0^{1,5}]undec-9-ene-3-carboxylate 2a

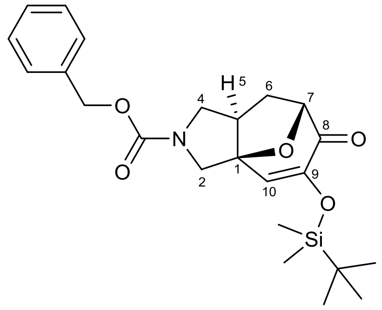

2a

A stirred solution of compound **S3** (15.9 g, 37.0 mmol) in xylenes (36 mL) was heated to reflux (155 °C) and stirred for 15 h. The reaction mixture was cooled to rt then concentrated *in vacuo*. Flash chromatography eluting with 9:1 to 8:2 pentane–EtOAc gave the *title compound* **2a** (13.8 g, 32.1 mmol, 87%) as a colourless amorphous solid.[*] **M.p.** 96-98 °C, colourless plates, hexane–EtOAc. **R*f*** 0.18 (4:1 petrol–EtOAc). **^{1}H NMR** (500 MHz, $CDCl_3$, 50:50 mixture of rotamers): δ 7.41-7.29 (5H, m, Cbz Ar-H), 6.29 (0.5H, s, 10-H), 6.26 (0.5H, s, 10-H), 5.15 (1H, app. d, *J* 12.0, OC*H*$_A$H$_B$Ph), 5.12 (1H, app. d, *J* 12.0, OCH$_A$*H*$_B$Ph), 4.78 (1H, d, *J* 8.2, 7-H), 4.04-3.90 (2H, m, 2-H$_B$ and 4-H$_A$), 3.68 (0.5H, d, *J* 12.8, 2-H$_A$), 3.64 (0.5H, d, *J* 12.8, 2-H$_A$), 3.22-3.13 (1H, m, 4-H$_B$), 2.84-2.74 (1H, m, 5-H), 2.34-2.21 (1H, m, 6-H$_B$), 1.89 (1H, app. td, *J* 13.2, 8.2, 6-H$_A$), 0.94 (4H, s, SiC(CH_3)$_3$), 0.93 (5H, s, SiC(CH_3)$_3$), 0.16 (6H, m, 2 × SiCH_3). **^{13}C NMR** (125 MHz, $CDCl_3$, mixture of two rotamers): δ 193.7 (8-C), 154.5 (N(CO)O), 154.3

[*] Compound **2a** and related cycloadducts **2b-c** were stable for several months when stored in a freezer at −18 °C.

(N(CO)O), 148.1 (9-C), 136.8 (Ar-C_q), 138.7 (Ar 1-C), 128.7 (Ar-C), 128.3 (Ar-C), 128.2 (Ar-C), 128.1 (Ar-C), 127.3 (10-C), 127.2 (10-C), 90.6 (1-C), 89.8 (1-C), 83.4 (7-C), 67.2 (O*C*H_2Ph), 53.9 (2-C or 4-C), 53.5 (2-C or 4-C), 53.1 (2-C or 4-C), 52.7 (2-C or 4-C), 47.1 (5-C), 46.2 (5-C), 31.6 (6-C), 31.5 (6-C), 25.7 (SiC(*C*H_3)$_3$), 18.6 (SiC_q), −4.5 (2 × SiCH_3) [28 of 36 expected peaks observed]. **IR** ν_{max}(film)/cm^{-1} 2954, 2953, 1703 (CO), 1652, 1419, 1347, 1163, 919. **HRMS** (ESI): $C_{23}H_{32}NO_5Si$ [M+H]$^+$; calculated 430.2044, found 430.2048. **X-ray crystallography**: CCDC 1526777 contains the supplementary crystallographic data for this compound. Crystals were grown by slow evaporation from diethyl ether.

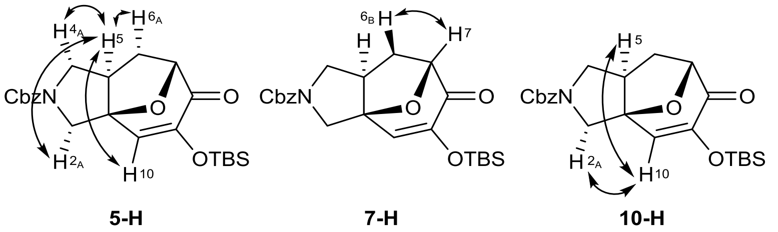

2a NOESY correlations:
5-H: 2-H_A; 4-H_A; 6-H_A; 10-H
7-H: 6-H_B
10-H: 2-H_A; 5-H

2a Crystal Structure:

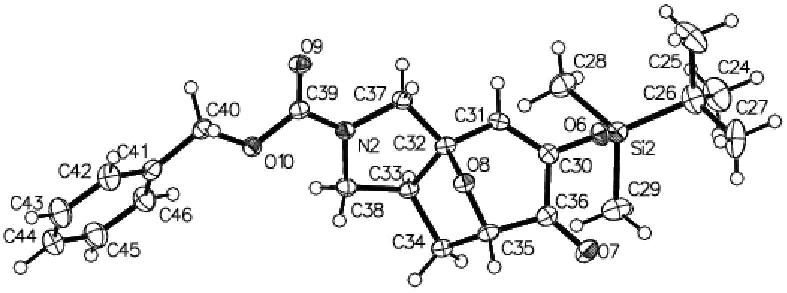

Benzyl (*1R,*7R**)-9-[(*tert*-butyldimethylsilyl)oxy]-8-oxo-11-oxa-3-azatricyclo[5.3.1.0^{1,5}]undeca-5,9-diene-3-carboxylate 2b**

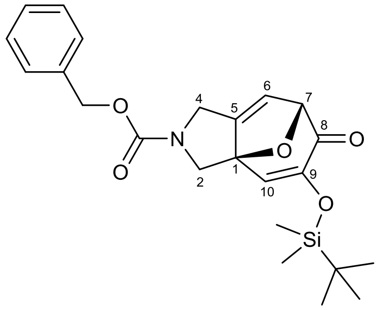

2b

In three batches, stirred solutions of compound **S6** (3 × 3.9 g, 3 × 9.1 mmol [9.1 mmol in each batch, 27.3 mmol over three batches]) in PhMe (3 × 10 mL [10 mL in each batch, 30 mL overall]) were heated at 180 °C under microwave irradiation for 6 h. The three batches were combined and concentrated *in vacuo*. Flash chromatography eluting with 9:1 pentane–EtOAc gave the *title compound* **2b** (5.5 g, 12.9 mmol, 47%) as a colourless amorphous solid. **R_f** 0.39 (4:1 petrol–EtOAc). **^{1}H NMR** (500 MHz, $CDCl_3$, 329 K): δ 7.40-7.30 (5H, m, Cbz Ar-H), 6.36 (1H, s, 10-H), 6.04 (1H, s, 6-H), 5.19 (2H, s, OC*H*$_2$Ph), 5.17-5.15 (1H, m, 7-H), 4.25 (1H, d, *J* 16.7, 2-H_A), 4.14 (1H, app. dd, *J* 16.7, 1.4, 2-H_B), 4.05-3.87 (1H, m, 4-H_A), 3.51 (1H, d, *J* 11.3, 4-H_B), 0.94 (9H, s, SiC(CH_3)$_3$), 0.17 (3H, s, SiCH_3), 0.16 (3H, s, SiCH_3). **^{13}C NMR** (125 MHz, $CDCl_3$, mixture of two rotamers): δ 190.8

(2 peaks, 8-C), 156.4 (9-C), 155.6 (9-C), 154.7 (N(CO)O), 154.5 (N(CO)O), 143.7 (5-C), 136.3 (Ar-C_q), 128.6 (Ar-C), 128.3 (Ar-C), 128.1 (Ar-C), 127.5 (10-C), 127.4 (10-C), 118.0 (6-C), 93.9 (7-C), 92.2 (1-C), 91.4 (1-C), 67.4 (O*C*H_2Ph), 51.9 (2 peaks, 4-C), 43.6 (2-C), 43.5 (2-C), 25.5 (SiC(*C*H_3)$_3$), 18.4 (SiC_q), −4.6 (SiCH_3), −4.7 (SiCH_3) [26 of 36 expected peaks observed]. **IR** ν_{max}(film)/cm^{-1} 2955, 2930, 2887, 2856, 1704, 1606, 1412, 1358. **HRMS** (ESI): $C_{23}H_{30}NO_5Si$ [M+H]$^+$; calculated 428.1888, found 428.1889.

5.4.1.4 Preparation of *O*-bridged cycloadduct 2c

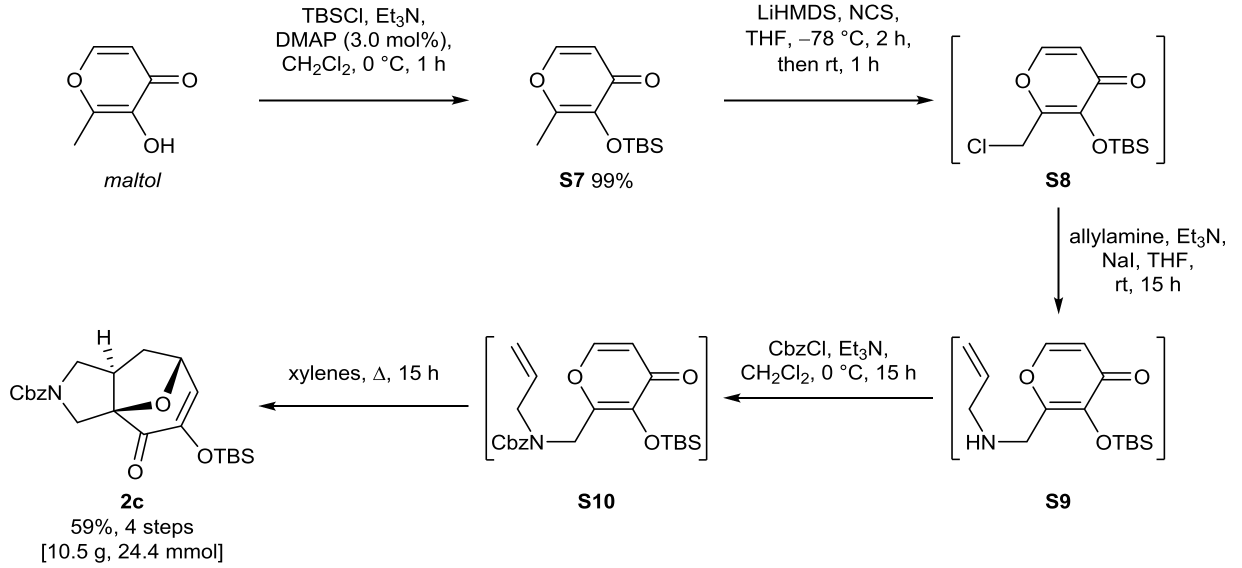

3-[(*tert*-Butyldimethylsilyl)oxy]-2-methyl-4H-pyran-4-one S7

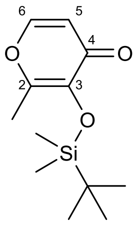

S7

TBSCl (12.1 g, 80.1 mmol, 1.01 eq.) was added to a stirred suspension of maltol (10.0 g, 79.3 mmol, 1.0 eq.), Et_3N (12.2 mL, 87.2 mmol, 1.1 eq.) and DMAP (290 mg, 2.40 mmol, 0.03 eq.) in CH_2Cl_2 (150 mL) at 0 °C. The ice-bath was removed and the reaction mixture was stirred for 3 h. The reaction mixture was poured into a solution of 1:1 sat. aq. NH_4Cl: H_2O (200 mL). The phases were separated and the aqueous phase was extracted with CH_2Cl_2 (2 × 50 mL), dried, filtered and concentrated *in vacuo* to give the title compound **S7** (19.0 g, 79.0 mmol, 99%) as a colourless amorphous solid which was not purified further. **^{1}H NMR** (300 MHz, $CDCl_3$, characteristic peaks): δ 7.57 (1H, d, *J* 5.7, 6-H), 6.30 (1H, d, *J* 5.7, 5-H), 2.31 (3H, s, CH_3), 0.97 (9H, s, SiC(CH_3)$_3$), 0.26 (6H, s, 2 × SiCH_3). Spectral data are consistent with the literature values.[S9]

Benzyl (*1R,*5R**,*7R**)-9-[(*tert*-butyldimethylsilyl)oxy]-10-oxo-11-oxa-3-azatricyclo[5.3.1.0^{1,5}]undec-8-ene-3-carboxylate 2c**

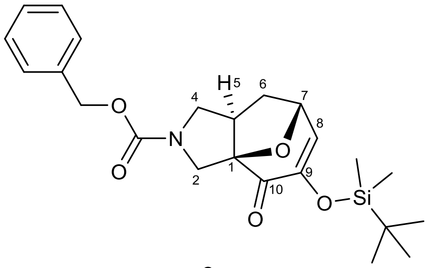

2c

LiHMDS (46.0 mL, 46.0 mmol, 1.10 eq.) was added dropwise to a stirred solution of compound **S7** (10.0 g, 41.6 mmol, 1.00 eq.) in THF (150 mL) at −78 °C. The reaction mixture was stirred at −78 °C for 1 h, then transferred dropwise *via* cannula to a stirred solution of NCS (5.60 g, 41.6 mmol, 1.00 eq.) in THF (150 mL) at −78 °C. The reaction mixture was stirred for 1 h at −78 °C, then slowly warmed to rt. H_2O (10 mL) was added, then the reaction mixture was concentrated *in vacuo*. The residue was partitioned between pentane[*] (100 mL) and sat. aq. $NaHCO_3$ solution (100 mL). The phases were separated and the aqeuos layer was further extracted with pentane (4 × 25 mL). The combined organic layers were dried over $MgSO_4$, filtered, and concentrated *in vacuo* to give the crude chloride **S8** as an orange oil (10.8 g), which was not purified further {characteristic ^{1}H NMR peaks (500 MHz, $CDCl_3$): δ 7.57 (1H, d, *J* 5.5, 6-H), 6.25 (1H, d, *J* 5.5, 5-H), 4.46 (2H, s, CH_2Cl), 0.88 (9H, s, $SiC(CH_3)_3$), 0.20 (6H, s, 2 × $SiCH_3$)}. To the residue **S8** (41.6 mmol) in THF (100 mL) was added Et_3N (5.8 mL, 41.6 mmol, 1.0 eq.), allylamine (4.7 mL, 108 mmol, 2.6 eq.) and NaI (6.2 g, 41.6 mmol, 1.0 eq.) at rt, in the order stated. The reaction mixture was stirred at rt for 15 h, then concentrated *in vacuo*. The resulting residue was diluted in EtOAc (100 mL) and washed with H_2O (100 mL). The aqueous layer was extracted with EtOAc (4 × 25 mL). The combined organic layers were washed with brine (50 mL), dried over $MgSO_4$, filtered, and concentrated *in vacuo* to give the crude amine **S9** as a brown oil which was not purifed further {characteristic ^{1}H NMR peaks (500 MHz, $CDCl_3$): δ 7.56 (1H, d, *J* 5.6, 6-H), 6.23 (1H, d, *J* 5.6, 5-H), 5.79 (1H, ddt, *J* 16.8, 10.3, 6.1, $CH_2CH{=}CH_AH_B$), 5.10 (1H, dd, *J* 16.8, 1.3, $CH{=}CH_AH_B$), 5.03 (1H, dd, *J* 10.3, 1.3, $CH{=}CH_AH_B$), 3.75 (2H, s, C_qCH_2NH), 3.17 (2H, d, *J* 6.1, $NHCH_2CH{=}CH_2$), 0.86 (9H, s, $SiC(CH_3)_3$), 0.18 (6H, s, 2 × $SiCH_3$)}. To the residue **S9** (41.6 mmol) in CH_2Cl_2 (100 mL) was added Et_3N (10.0 mL, 136 mmol, 3.30 eq.) followed by the very slow addition (*exothermic – gas outlet necessary!*) of CbzCl (8.8 mL, 43.1 mmol, 1.05 eq.) at 0 °C. The reaction mixture was warmed to rt and stirred for 2 h. The reaction mixture was quenched with sat. aq. NH_4Cl solution (150 mL) and H_2O (150 mL). After phase separation, the aqueous phase was extracted with CH_2Cl_2 (2 × 150 mL). The organic extracts were combined, dried over Na_2SO_4 and concentrated *in vacuo*. The resulting residue was flushed through a pad of SiO_2 eluting with EtOAc to give the crude Cbz-protected amine **S10** (15.0 g) as a brown oil {characteristic ^{1}H NMR peaks (300 MHz, $CDCl_3$, 50:50 mixture of rotamers): 7.61 (0.5H,

[*] Analysis of the crude reaction product by ^{1}H NMR spectroscopy at 300 MHz suggested that extraction with pentane removed the majority of the unreacted *N*-chlorosuccinimide.

br. s, 6-H), 7.48 (0.5H, br. s, 6-H), 7.41-7.27 (5H, m, Cbz Ar-H), 6.30 (1H, br. s, 5-H), 5.86-5.62 (1H, m, C*H*=CH_2), 5.23-4.99 (2H, m, CH=C*H*$_2$), 5.18 (2H, s, OC*H*$_2$Ph), 4.77-4.51 (2H, m, NC*H*$_2$CH=CH_2), 3.93 (1H, s, C*H*$_2$N^{rotA}), 3.86 (1H, s, C*H*$_2$N^{rotB}), 0.94 (9H, s, SiC(CH_3)$_3$), 0.26 (6H, s, SiCH_3)}. The residue **S10** was diluted in xylenes (30 mL) and heated at 155 °C for 15 h. The reaction mixture was cooled to rt and concentrated *in vacuo*. Flash chromatography eluting with 4:1 pentane–EtOAc gave the *title compound* **2c** (10.5 g, 24.4 mmol, 59%, 4 steps) as a yellow oil. **R*f*** 0.11 (4:1 petrol–EtOAc). **^{1}H NMR** (500 MHz, $CDCl_3$, 60:40 mixture of rotamers): δ 7.39–7.34 (4H, m, Cbz Ar-H), 7.34–7.28 (1H, m, Cbz Ar-H), 6.37–6.32 (1H, m, 8-H), 5.13 (2H, s, OC*H*$_2$Ph), 5.00 (1H, app. t, *J* 5.8, 7-H), 4.41–4.33 (1H, m, 2-H_A, includes at δ 4.36: 0.6H, d, *J* 13.0), 4.10–3.97 (1H, m, 4-H_A), 3.72–3.60 (1H, m, 2-H_B, includes at δ 3.69: 0.4H, d, *J* 13.0; and at δ 3.64: 0.6 H, d, *J* 13.0), 3.41–3.30 (1H, m, 4-H_B), 2.73–2.63 (1H, m, 5-H), 2.23–2.13 (1H, m, 6-H_A), 2.12–2.00 (1H, m, 6-H_B), 0.93 (9H, s, SiC(CH_3)$_3$), 0.18–0.12 (6H, m, 2 × SiCH_3, includes at δ 0.16: 3.6H, s; and at δ 0.14: 2.4H, s). **^{13}C NMR** (125 MHz, $CDCl_3$, mixture of two rotamers, 328 K): δ 191.6 (10-C), 154.6 (N(CO)O), 146.1 (9-C), 137.0 (Ar-C_q), 129.4 (8-C), 128.6 (Ar-C), 128.1 (Ar-C), 128.0 (Ar-C), 97.2 (1-C), 76.2 (7-C), 67.2 (O*C*H$_2$Ph), 53.4 (4-C), 49.5 (2-C), 43.9 (5-C), 43.1 (5-C), 37.7 (6-C), 25.7 (SiC(*C*H$_3$)$_3$), 18.5 (SiC_q), -4.5 (SiCH_3), -4.6 (SiCH_3) [20 of 36 expected peaks observed]. **IR** ν_{max}(film)/cm^{-1} 2955, 2931, 2885, 2858, 1704 (CO), 1622, 1419, 1361, 1338, 1261. **HRMS** (ESI): $C_{23}H_{32}NO_5Si$ [M+H]$^+$; calculated 430.2044, found 430.2050.

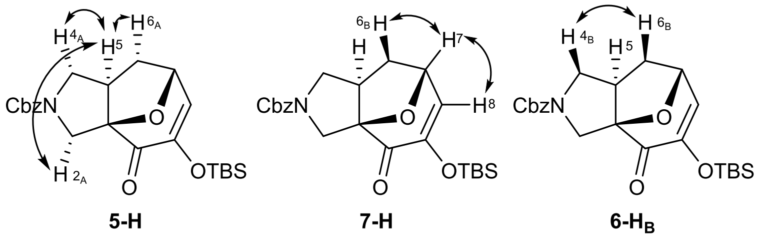

5-H **7-H** **6-H$_B$**

2c NOESY correlations:
5-H: 2-H_A; 4-H_A; 6-H_A
6-H$_B$: 4-H_B; 7-H
7-H: 6-H_B; 8-H

5.4.1.5 Preparation of precursors to *N*-bridged cycloadducts 2d-g

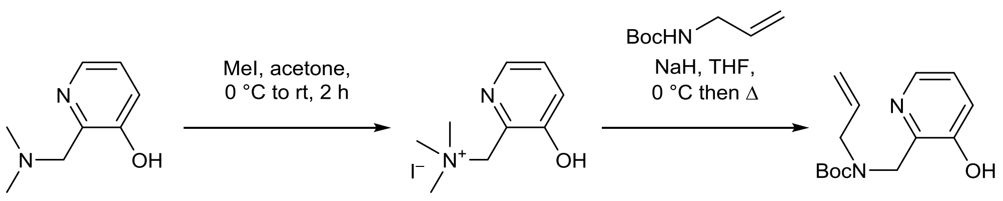

S11 88% **S12** 55% [9.5 g, 3.6 mmol]

[(3-Hydroxypyridin-2-yl)methyl]trimethylazanium iodide S11

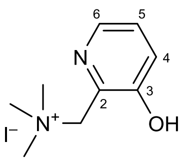

S11

Methyl iodide (8.40 mL, 132 mmol, 1.00 eq.) was added to stirred solution of 2-(dimethylaminomethyl)-3-hydroxypyridine (20.1 g, 132 mmol, 1.00 eq.) in acetone (66 mL) at 0 °C. The resulting mixture was warmed to rt and stirred for 2 h, during which time a pale yellow precipitate formed. The solid was collected by filtration to give the title compound **S11** (34.1 g, 115.9, 88%) as a pale yellow solid. **M.p.** Decomposition observed above 164 °C. **^{1}H NMR** (D_2O, 400 MHz): 8.21 (1H, dd, *J* 3.9, 2.1, 5-H), 7.56-7.47 (2H, m, 4-H and 6-H), 4.61 (2H, s, CH_2Ar), 3.21 (9H, s, $N^+(CH_3)_3$). **^{13}C NMR** (D_2O, 100 MHz): 154.3, 141.1, 134.7, 127.6, 125.7, 64.5, 53.2. **IR** ν_{max}(film)/cm^{-1} 3381, 1629, 1583, 1484, 1462, 1300. **HRMS** (ESI): $C_9H_{15}N_2O$ [M]$^+$; calculated 167.1184, found 167.1186.

***tert*-Butyl *N*-[(3-hydroxypyridin-2-yl)methyl]-*N*-(prop-2-en-1-yl)carbamate S12**

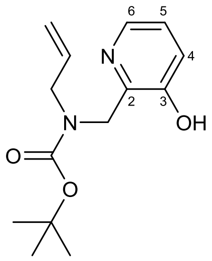

S12

N-Boc-allylamine (10.2 g, 65.1 mmol, 1.00 eq.) was added to a stirred suspension of NaH (60% dispersion in mineral oil, 5.47 g, 137 mmol, 2.10 eq.) in THF (280 mL) at 0 °C. The resulting suspension was stirred at rt for 2 h. After this time, the suspension was cooled to 0 °C and trimethyl ammonium salt **S11** (21.1 g, 71.6 mmol, 1.10 eq.) was added in one portion. The suspension was stirred at reflux for 2 h, then cooled to rt and quenched with sat. aq. NH_4Cl solution (100 mL). EtOAc (100 mL) was added and the layers were separated. The aqueous layer was extracted with EtOAc (2 x 100 mL). The combined organic layers were dried over $MgSO_4$, filtered, and concentrated *in vacuo* to deliver a crude brown oil. Flash chromatography eluting with (4:5:1 petrol–CH_2Cl_2–EtOAc) gave the *title compound* **S12** (9.47 g, 35.8 mmol, 55%) as a colourless solid. **^{1}H NMR** (MeOD-d_4, 500 MHz, 333 K): 7.87 (1H, dd, *J* 4.4, 1.7, 6-H), 7.08 (1H, dd, *J* 8.1, 1.6, 4-H), 7.05 (1H, dd, *J* 8.1, 4.4, 5-H), 5.67 (1H, ddt, *J* 17.0, 10.4, 5.6, C*H*=CH_2), 4.98 (1H, app. dq, *J* 17.0, 1.7, CH=CH_AH_B), 4.96 (1H, app dq, *J* 10.4, 1.5, CH=CH$_AH_B$), 4.37 (2H, s, CH_2Ar), 3.80 (2H, d, *J* 5.6, NCH_2CH=CH_2), 1.33 (9H, s, $C_q(CH_3)_3$). **^{13}C NMR** (MeOD-d_4, 125 MHz, 333 K): 158.3, 153.7, 146.0, 140.6, 134.9, 124.9, 124.3, 116.7, 81.7, 50.9, 48.7, 28.7. **IR** ν_{max}(film)/cm^{-1} 3271, 1651, 1447, 1414, 1161. **HRMS** (ESI): $C_{14}H_{21}N_2O_3$ [MH]$^+$; calculated 265.1547, found 265.1551.

5.4.1.6 Preparation of the *N*-bridged cycloadducts 2d-g

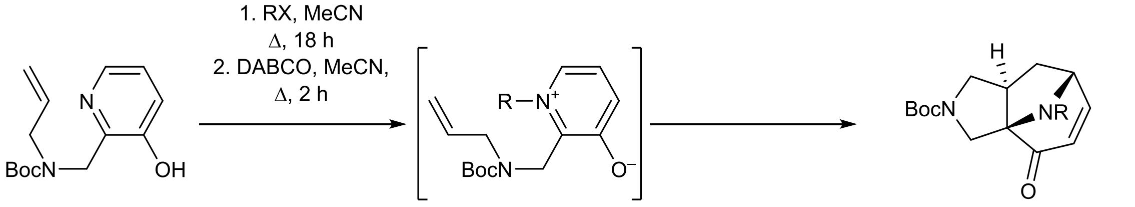

S12 **1d-g**

2d R= Me, 59%
2e R= 2-bromobenzyl, 31%
2f R= Bn, 65%
2g R= CH_2CO_2Et, 34%

General procedure K: Synthesis of *N*-bridged cycloadducts 2d-g

Alkyl halide (RX, 1.1 eq.) was added to a stirred solution of compound **S12** (1.0 eq.) in MeCN (0.5 M) and the resulting solution was stirred at reflux for 18 h. After cooling to rt, DABCO (3.0 eq.) was added in one portion and the resulting suspension was stirred at reflux for 2 h, then cooled to rt. H_2O (3 volumes) and CH_2Cl_2 (3 volumes) were added and the layers were separated. The aqueous layer was extracted with CH_2Cl_2 (3 volumes) and the combined organic layers were dried over $MgSO_4$, filtered, and concentrated *in vacuo* to give a crude material. The crude products were purified by flash chromatography.

***tert*-Butyl (*1R**,*5R**,*7R**)-11-methyl-10-oxo-3,11-diazatricyclo[5.3.1.$0^{1,5}$]undec-8-ene-3-carboxylate 2d**

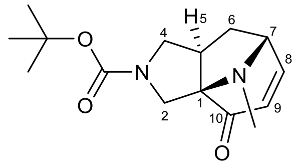

2d

General procedure **K** was followed using compound **S12** (7.8 g, 29.5 mmol) and methyl iodide. Flash chromatography eluting with 4:5:1 petrol–CH_2Cl_2–EtOAc gave the *title compound* **2d** (4.88 g, 17.5 mmol, 59%) as a yellow solid. **^{1}H NMR** (MeOD-d_4, 500 MHz, 333 K): 6.95 (1H, dd, *J* 9.8, 4.8, 8-H), 5.90 (1H, d, *J* 9.8, 9-H), 3.96 (1H, d, *J* 12.4, 2-H_A), 3.86 (1H, td, *J* 5.2, 0.9, 7-H), 3.81 (1H, dd, *J* 10.9, 9.4, 4-H_B), 3.21 (1H, dd, *J* 10.9, 8.1, 4-H_A), 3.18 (1H, d, *J* 12.4, 2-H_B), 2.57-2.47 (1H, m, 5-H), 2.25 (3H, s, NCH_3), 1.98-1.89 (2H, m, 6-H), 1.37 (9H, s, $C_q(CH_3)_3$). **^{13}C NMR** (MeOD-d_4, 125 MHz, 333 K): 197.4, 156.0, 151.3, 127.2, 82.7, 81.1, 64.9, 54.3, 47.1, 45.7, 35.6, 32.5, 28.8. **IR** ν_{max}(film)/cm^{-1} 1675, 1403, 1365, 1165, 1140, 1125, 1113, 881. **HRMS** (ESI): $C_{15}H_{23}N_2O_3$ $[MH]^+$; calculated 279.1703, found 279.1704. **X-ray crystallography**: CCDC 1526780 contains the supplementary crystallographic data for this compound. Crystals were grown by slow evaporation from dichloromethane.

2d Crystal Structure:

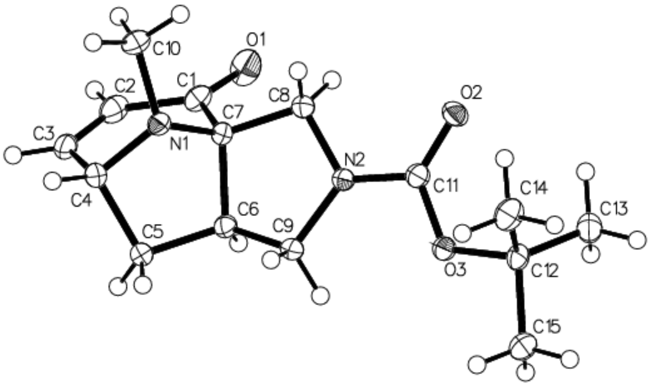

***tert*-Butyl (1*R**,5*R**,7*R**)-11-[(2-bromophenyl)methyl]-10-oxo-3,11-diazatricyclo[5.3.1.0^{1,5}]undec-8-ene-3-carboxylate 2e**

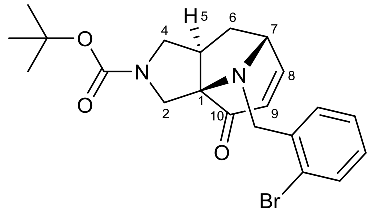

2e

General procedure **K** was followed using compound **S12** (1.2 g, 4.5 mmol) and 2-bromobenzyl bromide. Flash chromatography eluting with 9:1 CH_2Cl_2–EtOAc gave the *title compound* **2e** (617 mg, 1.42 mmol, 31%) as a yellow oil. **^{1}H NMR** (MeOD-d$_4$, 500 MHz, 333 K): 7.43 (1H, dd, *J* 8.0, 1.1, Ar 3-H), 7.26 (1H, dd, *J* 7.6, 1.6, Ar 6-H), 7.19 (1H, app. td, *J* 7.5, 1.1, Ar 5-H), 7.05 (1H, app. td, *J* 7.7, 1.6, Ar 4-H), 7.04 (1H, dd, *J* 9.8, 4.7, 8-H), 6.00 (1H, d, *J* 9.8, 9-H), 3.99 (1H, d, *J* 12.6, 2-H$_A$), 3.82 (1H, dd, *J* 10.8, 9.2, 4-H$_B$), 3.76 (1H, d, *J* 14.1, NC*H*$_A$H$_B$Ar), 3.76-3.71 (1H, m, 7-H), 3.65 (1H, d, *J* 14.1, NCH$_A$*H*$_B$Ar), 3.40 (1H, d, *J* 12.6, 2-H$_B$), 3.30 (1H, dd, *J* 10.8, 8.0, 4-H$_A$), 2.55 (1H, app. qd, *J* 8.4, 4.5, 5-H), 1.93 (1H, dd, *J* 12.2, 8.5, 6-H$_A$), 1.90-1.78 (1H, m, 6-H$_B$), 1.35 (9H, s, C$_q$(CH$_3$)$_3$). **^{13}C NMR** (MeOD-d$_4$, 125 MHz, 333 K): 197.0, 156.1, 152.7, 139.3, 134.0, 131.8, 130.0, 128.6, 127.8, 125.1, 82.7, 81.0, 61.3, 54.5, 50.3, 47.4, 45.4, 35.5, 28.8. **IR** ν_{max}(film)/cm^{-1} 1676, 1406, 1167, 1122, 887. **HRMS** (ESI): $C_{21}H_{26}{}^{79}BrN_2O_3$ [MH]$^+$; calculated 433.1121, found 433.1115.

tert-Butyl (1R*,5R*,7R*)-11-benzyl-10-oxo-3,11-diazatricyclo[$5.3.1.0^{1,5}$]undec-8-ene-3-carboxylate 2f

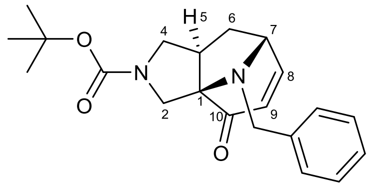

2f

General procedure **K** was followed using compound **S12** (8.57 g, 32.4 mmol) and benzyl bromide. Flash chromatography eluting with 4:5:1 petrol–CH_2Cl_2–EtOAc gave the *title compound* **2f** (7.42 g, 20.9 mmol, 65%) as a yellow oil. **^{1}H NMR** (MeOD-d$_4$, 500 MHz, 333 K): 7.21-7.09 (5H, m, Bn Ar-H), 6.95 (1H, dd, *J* 9.8, 4.8, 8-H), 5.99 (1H, d, *J* 9.8, 9-H), 3.98 (1H, d, *J* 12.5, 2-H$_A$), 3.82 (1H, dd, *J* 10.9, 9.2, 4-H$_B$), 3.76-3.69 (1H, m, 7-H), 3.63 (1H, d, *J* 13.7, NCH_AH$_B$Ph), 3.56 (1H, d, *J* 13.7, NCH$_A$$H_B$Ph), 3.31 (1H, dd, *J* 10.9, 7.9, 4-H$_A$), 3.30-3.23 (1H, m, 2-H$_B$), 2.55 (1H, app. qd, *J* 8.5, 4.7, 5-H), 1.93 (1H, dd, *J* 12.1, 8.5, 6-H$_A$), 1.80-1.89 (1H, m, 6-H$_B$), 1.36 (9H, s, C$_q$(CH_3)$_3$). **^{13}C NMR** (MeOD-d$_4$, 125 MHz, 333 K): 197.3, 156.1, 152.1, 140.3, 129.4, 129.3, 128.2, 127.9, 82.7, 81.1, 61.4, 54.3, 50.4, 47.6, 45.8, 35.3, 28.7. **IR** ν_{max}(film)/cm^{-1} 1681, 1403, 1365, 1167, 1125, 882. **HRMS** (ESI): $C_{21}H_{27}N_2O_3$ [MH]$^+$; calculated 355.2016, found 355.2023.

tert-Butyl (1R*,5R*,7R*)-11-(2-ethoxy-2-oxoethyl)-10-oxo-3,11-diazatricyclo[$5.3.1.0^{1,5}$]undec-8-ene-3-carboxylate 2g

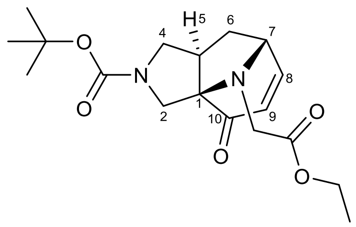

2g

General procedure **K** was followed using compound **S12** (2.44 g, 9.23 mmol) and ethyl bromoacetate. Flash chromatography eluting with 3:5:2 petrol–CH_2Cl_2–EtOAc gave the *title compound* **2g** (1.10 g, 3.14 mmol, 34%) as a yellow oil. **^{1}H NMR** (MeOD-d$_4$, 500 MHz, 333 K): 7.01 (1H, dd, *J* 9.8, 4.8, 8-H), 5.93 (1H, d, *J* 9.8, 9-H), 4.20-4.12 (1H, m, 7-H), 4.11-4.02 (2H, m, $CO_2CH_2CH_3$), 3.99 (1H, d, *J* 12.5, 2-H$_A$), 3.82 (1H, dd, *J* 10.8, 9.2, 4-H$_B$), 3.33 -3.24 (2H, m, 4-H$_A$ and NCH_AH$_B$CO$_2$Et), 3.18 (1H, *J* 16.6, NCH$_A$$H_BCO_2$Et), 3.17 (1H, d, *J* 12.5, 2-H$_B$), 2.51 (1H, app. qd, *J* 8.5, 4.9, 5-H), 2.03-1.91 (2H, m, 6-H), 1.36 (9H, s, C$_q$(CH_3)$_3$), 1.15 (3H, t, *J* 7.1, $CO_2CH_2CH_3$). **^{13}C NMR** (MeOD-d$_4$, 125 MHz, 333 K, one C not observed): 196.3, 172.3, 156.1, 152.5, 127.9, 81.2, 62.7, 62.0, 54.2, 48.6, 47.7, 47.6, 35.2, 28.8, 14.4. **IR** ν_{max}(film)/cm^{-1} 1746, 1682, 1406, 1164, 1128, 766. **HRMS** (ESI): $C_{18}H_{27}N_2O_5$ [MH]$^+$; calculated 351.1914, found 351.1917.

5.4.2 Preparation of scaffolds

5.4.2.1 Scaffolds derived from cycloadduct 2a

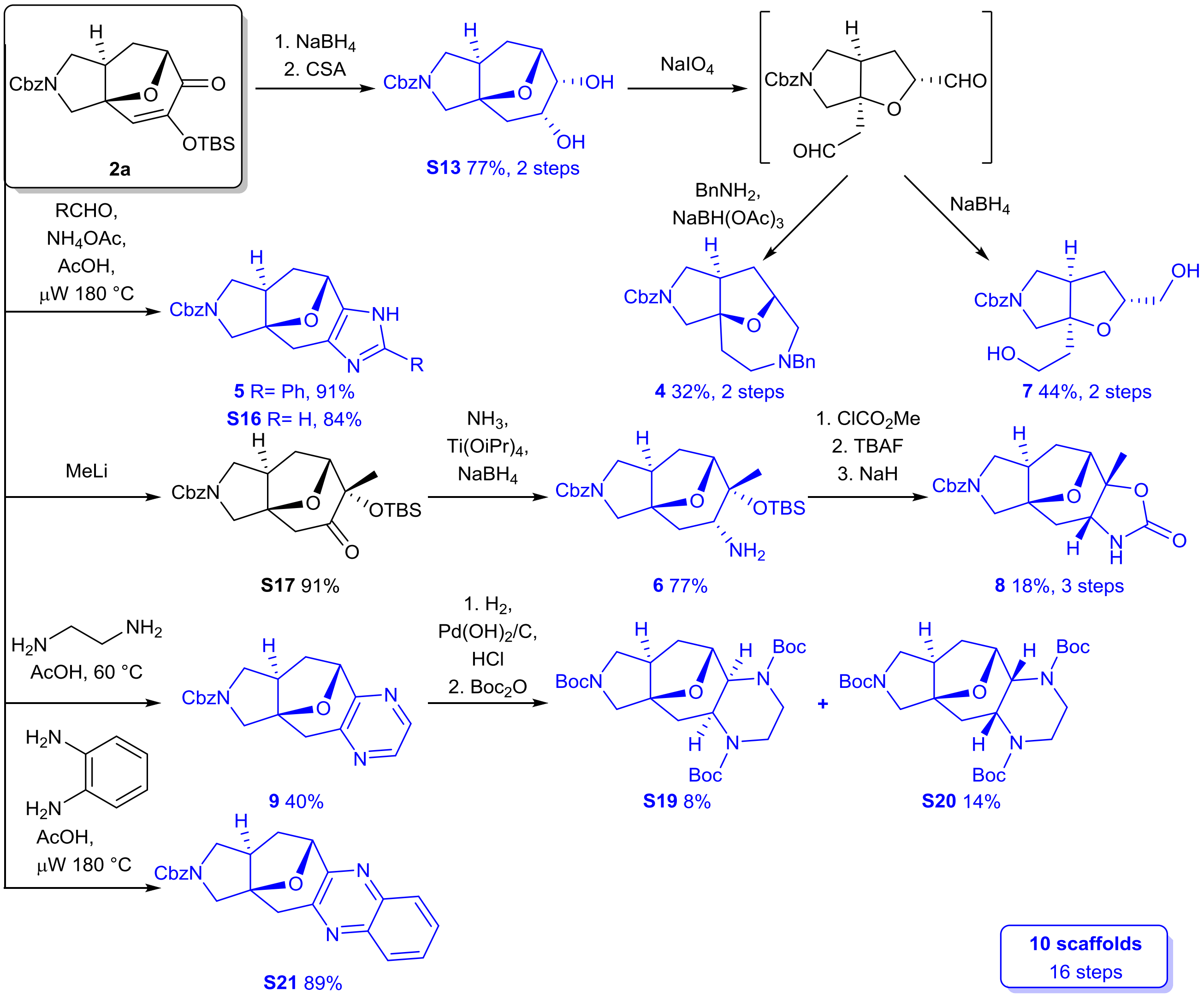

5.4.2.1.1 Preparation of cyclic amine 4, and diols 7 and S13

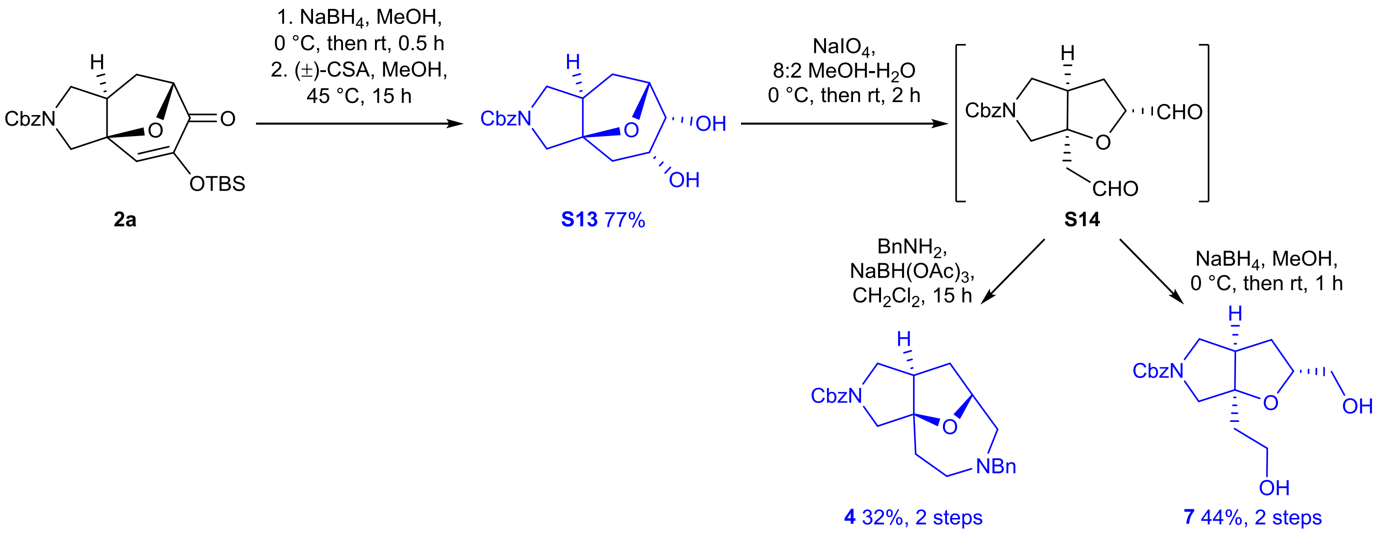

Benzyl (*1R*,5R*,7R*,8R*,9R)-8,9-dihydroxy-11-oxa-3-azatricyclo[5.3.1.0^{1,5}]undecane-3-carboxylate S13**

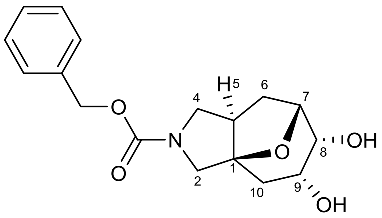

S13

$NaBH_4$ (832 mg, 22.0 mmol, 2.20 eq.) was added to a stirred solution of cycloadduct **2a** (4.30 g, 10.0 mmol, 1.0 eq.) in MeOH (60 mL) at 0 °C. The reaction mixture was stirred at 0 °C for 5 min, warmed to rt and stirred for 0.5 h, then concentrated *in vacuo*. The residue was diluted in CH_2Cl_2 (50 mL) and washed with 1N HCl (50 mL). The aqueous phase was extracted with CH_2Cl_2 (50 mL). The organic extracts were combined, washed with brine (50 mL), dried over Na_2SO_4, filtered, and concentrated *in vacuo*. The residue (10.0 mmol) was dissolved in MeOH (60 mL) and (±)-camphorsulfonic acid (3.02 g, 13.0 mmol, 1.30 eq.) was added. The reaction mixture was heated at 45 °C for 15 h, then concentrated *in vacuo*. The residue was diluted in CH_2Cl_2 (50 mL) and washed with 1:1 sat. aq. $NaHCO_3$:H_2O (100 mL). The aqueous phase was extracted with CH_2Cl_2 (2 × 50 mL). The organic extracts were combined, dried over Na_2SO_4 and concentrated *in vacuo*. Flash chromatography eluting with 0-10% MeOH in EtOAc gave the *title compound* **S13** (2.45 g, 7.67 mmol, 77%, 2 steps) as a colourless oil. **R*f*** 0.58 (9:1 EtOAc–MeOH). **^{1}H NMR** (500 MHz, $CDCl_3$, 50:50 mixture of rotamers): δ 7.41-7.27 (5H, m, Cbz Ar-H), 5.11 (2H, s, OC*H*$_2$Ph), 4.35 (1H, dd, *J* 7.2, 4.8, 7-H), 4.15-4.10 (1H, m, 9-H), 3.93-3.85 (1H, m, 4-H_A, includes at δ 3.91: 0.5H, d, *J*, 10.5; and at δ 3.87: 0.5H, d, *J*, 10.5), 3.86-3.80 (1H, m, 8-H), 3.79-3.71 (1H, m, 2-H_A, includes at δ 3.76: 0.5H, d, *J*, 12.6; and at δ 3.74: 0.5H, d, *J*, 12.6), 3.41 (0.5H, d, *J* 12.6, 2-H_B), 3.36 (0.5H, d, *J* 12.6, 2-H_B), 3.24-3.15 (1H, m, 4-H_B), 3.08-3.00 (1H, m, 5-H), 2.63 (1H, app. td, *J* 12.7, 8.5, 6-H_A), 2.49 (2H, br. s, 2 × OH), 2.19 (0.5H, dd, *J* 14.7, 4.3, 10-H_B), 2.13

(0.5H, dd, *J* 14.7, 4.3, 10-H_B), 1.97-1.90 (1H, m, 10-H_A, includes at δ 1.95: 0.5H, d, *J* 14.7; and at δ 1.93: 0.5H, d, *J* 14.7), 1.78-1.66 (1H, m, 6-H_B). **^{13}C NMR** (125 MHz, DMSO-d_6, mixture of two rotamers): δ 153.5 (N(CO)O), 153.4 (N(CO)O), 137.1 (Ar-C_q), 128.4 (Ar-C), 127.7 (Ar-C), 127.5 (Ar-C), 88.7 (1-C), 87.7 (1-C), 79.0 (7-C), 68.0 (8-C), 65.9 (9-C), 65.7 (O*C*H_2Ph), 54.8 (2-C or 4-C), 54.5 (2-C or 4-C), 54.2 (2-C or 4-C), 54.0 (2-C or 4-C), 44.2 (5-C), 43.2 (5-C), 38.0 (10-C), 37.9 (10-C), 32.7 (6-C), 32.6 (6-C) [22 of 30 expected peaks observed]. **IR** ν_{max}(film)/cm^{-1} 3423 (OH), 2948, 2884, 1683 (CO), 1425, 1350, 1149, 1107. **HRMS** (ESI): $C_{17}H_{22}NO_5$ [M+H]$^+$; calculated 320.1495, found 320.1496.

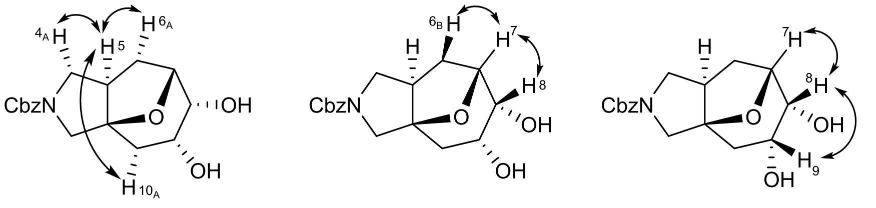

5-H **7-H** **8-H**

S13 NOESY correlations:
5-H: OH; 4-H_A; 6-H_A; 10-H_A
7-H: 6-H_B; 8-H
8-H: OH; 7-H; 9-H

Benzyl (*1R,*5R**,*7R**)-9-benzyl-12-oxa-3,9-diazatricyclo[5.4.1.0^{1,5}]dodecane-3-carboxylate 4**

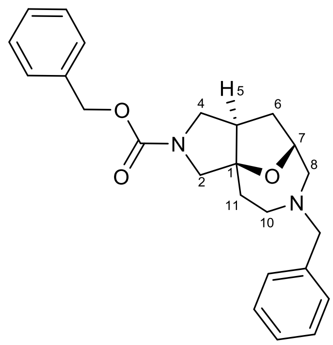

4

$NaIO_4$ (105 mg, 0.490 mmol, 2.00 eq.) was added to a stirred solution of diol **S13** (78 mg, 0.24 mmol, 1.0 eq.) in 8:2 MeOH–H_2O (10 mL) at 0 °C. The reaction mixture was warmed to rt an stirred for 2 h. The reaction mixture was concentrated *in vacuo* and the residue was diluted in CH_2Cl_2 (10 mL), and washed with H_2O (10 mL). The phases were separated, and the aqueous phase extracted with CH_2Cl_2 (2 × 10 mL). The organic extracts were combined, washed with brine, dried over Na_2SO_4 and concentrated *in vacuo*. The resulting crude dialdehyde was dissolved in CH_2Cl_2 (10 mL). $BnNH_2$ (26 µL, 0.25 mmol, 1.0 eq.), $NaBH(OAc)_3$ (153 mg, 0.72 mmol, 3.0 eq.) and 4 Å MS (10 mg) were added. The reaction mixture was stirred for 15 h then filtered through Celite and concentrated *in vacuo*. The resulting residue was diluted in EtOAc (25 mL) and washed with brine (25 mL). The aqueous phase was extracted with EtOAc (2 × 10 mL). The combined organic phase was dried over $MgSO_4$, filtered and concentrated *in vacuo*. Flash chromatography eluting with 0-100% EtOAc in pentane gave the *title compound* **4** (30 mg, 76 µmol, 32%, 2 steps) as a colourless oil. **R_f** 0.74 (1:1 petrol–EtOAc). **^{1}H NMR** (500 MHz, $CDCl_3$, 50:50 mixture of rotamers): δ 7.39-7.21 (10H, m, Ar-H), 5.11 (2H, s, OC*H*$_2$Ph), 4.43-4.38 (1H, m, 7-H, includes at δ 4.41: 0.5H, d, *J* 8.1; and at δ 4.40: 0.5H, d, *J* 8.1), 3.89 (1H, d, *J* 12.3, 2-H_A), 3.64-3.53 (3H, includes: 1H, m, 4-H_A; at δ 3.61: 1H, d, *J* 13.3, NC*H*$_A$$H_B$Ph; and at δ 3.55: 1H, d, *J* 13.3, NCH_A*H*$_B$Ph), 3.52-3.33 (1H, m, 4-H_B), 3.22-3.06 (1H, m, 2-H_B), 2.90-2.80 (1H, m, 5-H), 2.77-2.68 (1H, m, 10-H_A), 2.58-2.44 (2H, includes: 1H, m, 10-H_B; and at δ 2.52: 1H, d, *J* 12.4, 8-H_A), 2.43-2.36 (1H, m, 8-H_B, includes at δ 2.40: 0.5H, d, *J* 12.4;

and at δ 2.39: 0.5H, d, *J* 12.4), 2.28-2.21 (1H, m, 6-H_A), 1.92-1.74 (3H, m, 6-H_B and 11-H). **^{13}C NMR** (125 MHz, $CDCl_3$, mixture of two rotamers): δ 155.1 (N(CO)O), 139.9 (Ar-C_q) 137.1 (Ar-C_q), 128.8 (Ar-C), 128.6 (Ar-C), 128.5 (Ar-C), 128.0 (2 peaks, 2 × Ar-C), 127.2 (Ar-C), 93.2 (1-C), 92.2 (1-C), 80.2 (7-C), 66.9 (O*C*H_2Ph), 64.3 (N*C*H_2Ph), 63.6 (8-C), 57.9 (2-C), 57.5 (2-C), 54.0 (4-C), 53.8 (4-C), 53.6 (10-C), 50.1 (5-C), 38.3 (11-C), 36.6 (6-C) [23 of 40 expected peaks observed]. **IR** ν_{max}(film)/cm^{-1} 2930, 2865, 1702 (CO), 1451, 1419, 1360, 1217, 1143. **HRMS** (ESI): $C_{24}H_{29}N_2O_3$ $[M+H]^+$; calculated 393.2173, found 393.2185.

Benzyl (*2R*,3aR*,6aR)-6a-(2-hydroxyethyl)-2-(hydroxymethyl)-hexahydro-2H-furo[2,3-c]pyrrole-5-carboxylate 7**

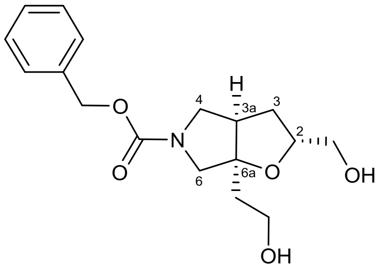

7

$NaIO_4$ (4.30 g, 20.1 mmol, 2.60 eq.) was added to a stirred solution of diol **S13** (2.45 g, 7.67 mmol, 1.0 eq.) in 2:1 MeOH–H_2O (60 mL) at 0 °C. The reaction mixture was warmed to rt, stirred for 2 h, then concentrated *in vacuo*. The residue was diluted in CH_2Cl_2 (50 mL) and washed with H_2O (50 mL). The aqueous phase was extracted with EtOAc (2 × 50 mL). The combined organic extracts were dried over $MgSO_4$, filtered and concentrated *in vacuo* to give crude aldehyde **S14** {characteristic ^{1}H NMR peaks (300 MHz, $CDCl_3$): δ 9.82 (1H, t, *J* 1.9, CH_2C*H*O), 9.64 (1H, d, *J* 1.4, CHC*H*O), 7.42-7.28 (5H, m, Cbz Ar-H), 5.13 (2H, s, OC*H*$_2$Ph)}. $NaBH_4$ (720 mg, 18.6 mmol, 2.40 eq.) was added to a stirred solution of the crude aldehyde **S14** in MeOH (50 mL) at 0 °C. The reaction mixture was warmed to rt, stirred 1 h, then concentrated *in vacuo*. The residue was diluted in CH_2Cl_2 (50 mL) and washed with brine (50 mL). The aqueous phase was extracted with EtOAc (3 × 50 mL). The combined organic phase was dried over $MgSO_4$, filtered and concentrated *in vacuo*. Flash chromatography eluting with 9:1 EtOAc–MeOH gave the *title compound* **7** (1.10 g, 3.40 mmol, 44%, 2 steps) as a colourless oil. **R_f** 0.43 (9:1 EtOAc–MeOH). **^{1}H NMR** (500 MHz, $CDCl_3$, 2 × OH not observed): δ 7.40-7.28 (5H, m, Cbz Ar-H), 5.13 (2H, s, OC*H*$_2$Ph), 4.33-4.26 (1H, m, 2-H), 3.93-3.79 (4H, m, 6-H_A, CHC*H*$_A$$H_B$OH and CH_2C*H*$_2$OH), 3.74 (1H, dd, *J* 11.4, 9.1, 4-H_B), 3.50 (1H, dd, *J* 12.5, 3.0, CHCH_A*H*$_B$OH), 3.47-3.28 (2H, m, 4-H_A and 6-H_B), 2.71-2.64 (1H, m, 3a-H), 2.21 (1H, ddd, *J* 12.8, 9.7, 7.3, 3-H_A), 2.03-1.95 (1H, m, C*H*$_A$$H_B$$CH_2$OH), 1.89-1.76 (2H, m, 3-H_B and CH_A*H*$_B$$CH_2$OH). **^{13}C NMR** (125 MHz, $CDCl_3$, one C_q not observed): δ 154.9 (N(CO)O), 137.1 (Ar-C_q), 128.7 (Ar-C), 128.2 (Ar-C), 128.1 (Ar-C), 80.1 (2-C), 67.2 (O*C*H_2Ph), 64.1 (CH*C*H_2OH), 60.2 (CH_2*C*H_2OH), 57.1 (6-C), 51.6 (4-C), 47.3 (3a-C), 39.8 (3-C), 33.0 (*C*$H_2$$CH_2$OH). **IR** ν_{max}(film)/cm^{-1} 3401 (OH), 2938, 2880, 1684 (CO), 1422, 1351,1217, 1100. **HRMS** (ESI): $C_{17}H_{24}NO_5$ $[M+H]^+$; calculated 322.1649, found 322.1649.

5.4.2.1.2 Preparation of imidazole scaffolds 5 and S16

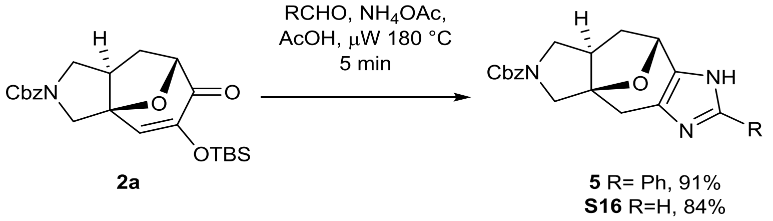

Benzyl (*1R,*8R**)-5-phenyl-14-oxa-4,6,12-triazatetracyclo[6.5.1.0^{1,10}.0^{3,7}]tetradeca-3(7),4-diene-12-carboxylate 5**

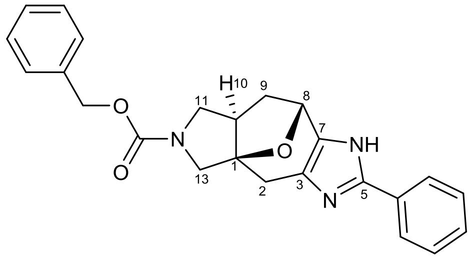

5

PhCHO (18 μL, 0.17 mmol, 1.0 eq.) and NH_4OAc (135 mg, 1.70 mmol, 10.0 eq.) were added to a suspension of cycloadduct **2a** (75 mg, 0.17 mmol, 1.0 eq.) in AcOH (3.0 mL). The resulting mixture was heated under microwave irradiation at 180 °C for 5 min. The reaction mixture was concentrated *in vacuo*, then partitioned between CH_2Cl_2 (25 mL) and $NaHCO_3$ (25 mL). The phases were separated and the aqueous phase was extracted with CH_2Cl_2 (2 × 10 mL). The combined organic extracts were dried, filtered and concentrated *in vacuo*. Flash chromatography eluting with 0-100% EtOAc in pentane gave the *title compound* **5** (64 mg, 0.16 mmol, 91%) as a pale brown oil. **R*f*** 0.12 (1:1 petrol–EtOAc). **^{1}H NMR** (500 MHz, $CDCl_3$, imidazole NH not observed): δ 7.76 (2H, d, *J* 7.3, Ar-H), 7.43-7.28 (8H, m, Ar-H), 5.28 (1H, d, *J* 5.7, 8-H), 5.25-5.17 (2H, m, OC*H*$_2$Ph), 4.07 (1H, d, *J* 12.6, 13-H_A), 3.85-3.73 (1H, m, 11-H_A), 3.55-3.36 (2H, m, 11-H_B and 13-H_B), 3.28-3.16 (1H, m, 2-H_A), 2.73-2.64 (1H, m, 10-H), 2.61 (1H, d, *J* 15.4, 2-H_B), 2.58-2.47 (1H, m, 9-H_A), 2.16-2.05 (1H, m, 9-H_B). **^{13}C NMR** (125 MHz, $CDCl_3$, mixture of two rotamers, 2 × imidazole C_q not observed): δ 154.9 (N(CO)O), 145.6 (5-C), 136.7 (Ar-C_q), 130.4 (Ar-C_q), 129.1 (Ar-C), 128.6 (Ar-C), 128.2 (Ar-C), 128.0 (2 peaks, Ar-C), 125.1 (Ar-C), 91.1 (1-C), 90.1 (1-C), 77.4 (8-C), 67.2 (O*C*H_2Ph), 55.4 (13-C), 55.0 (13-C), 53.6 (11-C), 53.5 (11-C), 47.1 (10-C), 46.1 (10-C), 45.8 (9-C), 45.6 (9-C), 32.8 (2-C) [23 of 40 expected peaks observed]. **IR** ν_{max}(film)/cm^{-1} 3274, 2241, 1682 (CO), 1448, 1418, 1348, 1116, 909. **HRMS** (ESI): $C_{24}H_{24}N_3O_3$ [M+H]$^+$; calculated 402.1812, found 402.1825.

Benzyl (*1R,*8R**,*10R**)-14-oxa-4,6,12-triazatetracyclo[6.5.1.0^{1,10}.0^{3,7}]tetradeca-3(7),4-diene-12-carboxylate S16**

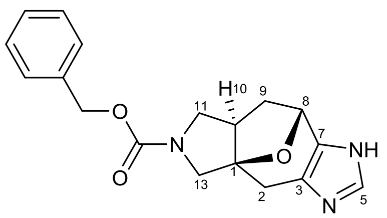

S16

In two equally sized batches, p-formaldehyde (2 × 245 mg, 2 × 8.20 mmol, 2.00 eq. [8.20 mmol in each batch, 16.4 mmol overall]) and NH_4OAc (2 × 3.10 g, 2 × 41.0 mmol, 2 × 10.0 eq. [41.0 mmol in each batch, 82.0 mmol overall]) were added to a suspension of cycloadduct **2a** (2 × 1.75 g, 2 × 4.10 mmol, 2 × 1.00 eq. [4.10 mmol in each batch, 8.20 mmol overall]) in AcOH (2 × 5 mL [5 mL in each batch, 10 mL overall]). Each reaction mixture was stirred at rt for 10 mins, then heated at heated at 180 °C under microwave irradiation for 5 min. The two batches were combined and concentrated *in vacuo*. The residue was diluted in EtOAc (50 mL) and washed with sat. aq. $NaHCO_3$ (50 mL). The aqeuous layer was extracted with EtOAc (4 × 20 mL). The combined organic layers were washed with H_2O (2 × 25 mL) and brine (25 mL), then dried over $MgSO_4$, filtered and concentrated *in vacuo*. Purification by SCX SPE following general procedure **A**, eluting with MeOH, then sat. NH_3/MeOH, followed by flash chromatography eluting with 90:9:1 CH_2Cl_2–EtOH–NH_3/MeOH gave the *title compound* **S16** (2.24 g, 6.9 mmol, 84%) as a pale brown foam. **R*f*** 0.64 (50:8:1 CH_2Cl_2–EtOH–NH_3/MeOH). **^{1}H NMR** (400 MHz, $CDCl_3$, imidazole NH not observed): δ 7.43 (1H, s, 5-H), 7.40-7.28 (5H, m, Cbz Ar-H), 5.25 (1H, d, *J* 5.9, 8-H), 5.16 (1H, d, *J* 13.0, OC$H_A$$H_B$Ph), 5.11 (1H, d, *J* 13.0, OC$H_A$$H_B$Ph), 4.06 (1H, d, *J* 12.7, 13-H_A), 3.82-3.71 (1H, m, 11-H_A), 3.54-3.34 (2H, m, 11-H_B and 13-H_B), 3.26-3.10 (1H, m, 2-H_A), 2.70-2.61 (1H, m, 10-H), 2.58 (1H, d, *J* 15.4, 2-H_B), 2.53-2.42 (1H, m, 9-H_A), 2.15-2.03 (1H, m, 9-H_B). **^{13}C NMR** (100 MHz, $CDCl_3$, mixture of two rotamers, 2 × imidazole C_q not observed): δ 154.9 (N(CO)O), 136.8 (Cbz Ar-C_q), 136.7 (Cbz Ar-C_q), 133.6 (2 peaks, Ar-C), 128.6 (Ar-C), 128.2 (Ar-C), 128.0 (Ar-C), 91.1 (1-C), 90.2 (1-C), 76.4 (8-C), 67.2 (OCH_2Ph), 55.4 (13-C), 55.0 (13-C), 53.6 (11-C), 47.1 (10-C), 46.1 (10-C), 45.8 (9-C), 45.7 (9-C), 32.7 (2-C) [20 of 32 expected peaks observed]. **IR** ν_{max}(film)/cm^{-1} 2958, 1694 (CO), 1423, 1352, 1239, 1218, 1115, 732. **HRMS** (ESI): $C_{18}H_{20}N_3O_3$ [M+H]$^+$; calculated 326.1499, found 326.1500.

5.4.2.1.3 Preparation of silyl-protected amino alcohol scaffold 6

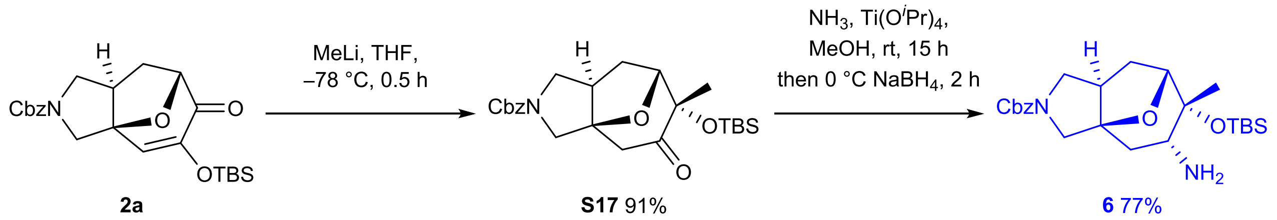

Benzyl (*1R**,*5R**,*7R**,*8S**)-8-[(*tert*-butyldimethylsilyl)oxy]-8-methyl-9-oxo-11-oxa-3-azatricyclo[5.3.1.0^{1,5}]undecane-3-carboxylate S17

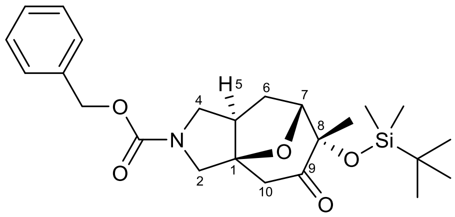

S17

MeLi (1.6 M in Et_2O, 0.37 mL, 0.60 mmol, 1.30 eq.) was added to a stirred solution of cycloadduct **2a** (200 mg, 0.46 mmol, 1.00 eq.) in THF (15 mL) at −78 °C. The reaction mixture was stirred at this temperature for 0.5 h, then sat. aq. brine (1 mL) was added. The reaction mixture was warmed to rt, then partitioned between EtOAc (25 mL) and brine (25 mL). The aqueous phase was extracted with EtOAc (2 × 10 mL). The combined organic extracts were dried, filtered and concentrated *in vacuo*. Flash chromatography eluting with 95:5 pentane–EtOAc gave the *title compound* **S17** (187 mg, 0.420 mmol, 91%) as a yellow oil. **R*f*** 0.30 (3:1 petrol–EtOAc). **^{1}H NMR** (500 MHz, $CDCl_3$, 50:50 mixture of rotamers): δ 7.39-7.28 (5H, m, Cbz Ar-H), 5.12 (2H, s, OC*H*$_2$Ph), 4.21-4.15 (1H, m, 7-H), 3.95-3.83 (2H, m, 2-H$_B$ and 4-H$_A$), 3.43 (0.5H, d, *J* 12.6, 2-H$_A$), 3.38 (0.5H, d, *J* 12.6, 2-H$_A$), 3.20-3.09 (1H, m, 4-H$_B$), 2.92 (0.5H, d, *J* 15.3, 10-H$_B$), 2.86 (0.5H, d, *J* 15.3, 10-H$_B$), 2.55-2.46 (1H, m, 5-H), 2.37 (0.5H, d, *J* 3.3, 10-H$_A$), 2.34 (0.5H, d, *J* 3.3, 10-H$_A$), 2.27-2.14 (1H, m, 6-H$_A$), 1.91-1.76 (1H, m, 6-H$_B$), 1.46 (1.5H, s, C$_q$C*H*$_3$), 1.45 (1.5H, s, C$_q$C*H*$_3$), 0.85 (9H, s, SiC(CH_3)$_3$), 0.17 (3H, s, SiCH_3), 0.13 (3H, s, SiCH_3). **^{13}C NMR** (125 MHz, $CDCl_3$, mixture of two rotamers): δ 208.0 (9-C), 207.9 (9-C), 154.4 (N(CO)O), 136.8 (Ar-C$_q$), 128.6 (Ar-C), 128.2 (Ar-C), 128.12 (Ar-C), 91.5 (1-C), 90.7 (1-C), 85.4 (7-C), 81.4 (8-C), 67.1 (OCH_2Ph), 54.2 (2-C or 4-C), 53.8 (2-C or 4-C), 53.7 (2-C or 4-C), 47.3 (10-C), 45.7 (5-C), 44.8 (5-C), 31.7 (6-C), 31.4 (6-C), 26.0 (SiC(*C*H$_3$)$_3$), 24.4 (C$_q$*C*H$_3$), 18.5 (SiC$_q$), −2.3 (SiCH_3), −2.6 (SiCH_3) [25 of 38 expected peaks observed]. **IR** ν_{max}(film)/cm^{-1} 2954, 2953, 2930, 2887, 1702 (CO), 1629,1593, 1419 **HRMS** (ESI): $C_{24}H_{36}NO_5Si$ [M+H]$^+$; calculated 446.2357, found 446.2360.

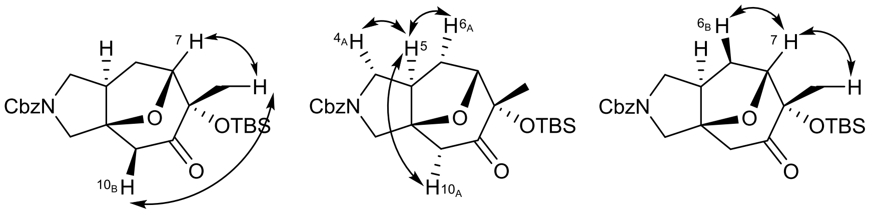

C_qCH_3 **5-H** **7-H**

S17 NOESY correlations:
C_qCH_3: 7-H; 10-H_B
5-H: 4-H_A; 6-H_A; 10-H_A
7-H: C_qCH_3; 6-H_B

Benzyl (*1R,*5R**,*7R**,*8R**,*9R**)-9-amino-8-[(*tert*-butyldimethylsilyl)oxy]-8-methyl-11-oxa-3-azatricyclo[5.3.1.0^{1,5}]undecane-3-carboxylate 6**

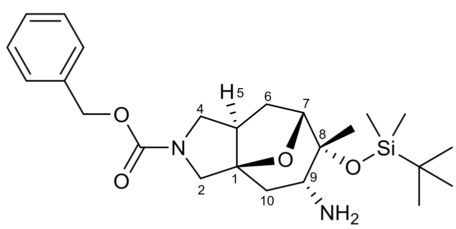

6

$Ti(O^iPr)_4$ (4.30 mL, 14.4 mmol, 2.00 eq.) was added to a stirred solution of compound **S17** (3.21 g, 7.20 mmol, 1.00 eq.) in sat. NH_3/MeOH (100 mL). The reaction mixture was stirred for 15 h then $NaBH_4$ (409 mg, 10.8 mmol, 1.5 eq.) was added at 0 °C. The reaction mixture was warmed to rt, stirred for 2 h then concentrated *in vacuo*. The residue was diluted in EtOAc (50 mL) and sat. aq. brine (50 mL) and stirred vigorously. The phases were separated and the aqueous phase was extracted with EtOAc (2 × 25 mL). The combined organic phase was dried over $MgSO_4$, filtered, and concentrated *in vacuo*. Flash chromatography eluting with EtOAc, then 9:1 EtOAc–MeOH, gave the *title compound* **6** (2.46 g, 5.51 mmol, 77%) as a colourless oil. **^{1}H NMR** (500 MHz, $CDCl_3$, 50:50 mixture of rotamers, NH_2 not observed): δ 7.38-7.27 (5H, m, Cbz Ar-H), 5.10 (2H, s, OCH_2Ph), 4.00 (1H, d, *J* 7.5, 7-H), 3.92-3.82 (1H, m, 4-H_A), 3.75-3.68 (1H, m, 2-H_B, includes at δ 3.72: 0.5H, d, *J* 12.5; and at δ 3.71: 0.5H, d, *J* 12.5), 3.38 (0.5H, d, *J* 12.5, 2-H_A), 3.33 (0.5H, d, *J* 12.5, 2-H_A), 3.23-3.12 (2H, m, 4-H_B and 9-H), 3.11-3.03 (1H, m, 5-H), 2.96-2.87 (1H, m, 6-H_A), 2.16 (0.5H, dd, *J* 14.2, 5.4, 10-H_B), 2.11 (0.5H, dd, *J* 14.2, 5.4, 10-H_B), 1.72-1.60 (1H, m, 6-H_B), 1.57-1.50 (1H, m, 10-H_A, includes at δ 1.54: 0.5H, d, *J* 14.2; and at δ 1.53: 0.5H, d, *J* 14.2), 1.37 (1.5H, s, C_qCH_3), 1.36 (1.5H, s, C_qCH_3), 0.91 (9H, s, $SiC(CH_3)_3$), 0.13 (3H, s, $SiCH_3$), 0.12-0.10 (3H, m, $SiCH_3$). **^{13}C NMR** (125 MHz, DMSO-d_6, mixture of two rotamers): δ 153.6 (N(CO)O), 153.4 (N(CO)O), 137.1 (Ar-C_q), 128.4 (Ar-C), 127.7 (Ar-C), 127.5 (Ar-C), 89.3 (1-C), 88.4 (1-C), 83.2 (7-C), 73.1 (8-C), 65.7 (OCH_2Ph), 54.7 (2-C or 4-C), 54.6 (2-C or 4-C), 54.1 (2-C or 4-C), 54.0 (2-C or 4-C), 53.8 (9-C), 44.4 (5-C), 43.4 (5-C), 36.7 (10-C), 36.6 (10-C), 32.9 (6-C), 32.8 (6-C), 27.5 (C_qCH_3), 25.8 ($SiC(CH_3)_3$), 18.0 (SiC_q), −2.0 ($SiCH_3$), −2.2 ($SiCH_3$) [27 of 38 expected peaks observed]. **IR** ν_{max}(film)/cm^{-1} 2952, 2931, 2882, 2856, 1704 (CO), 1419, 1362, 1346. **HRMS** (ESI): $C_{24}H_{39}N_2O_4Si$ $[M+H]^+$; calculated 447.2674, found 447.2679.

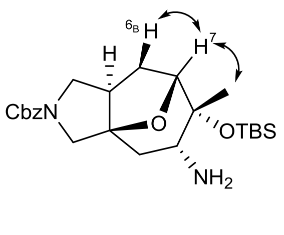

7-H

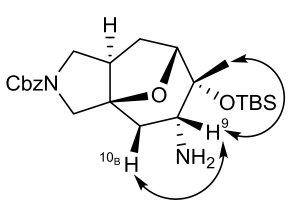

9-H

6 NOESY correlations:

Me: 7-H; 9-H

7-H: Me; 6-H_B

9-H: Me; 10-H_B

5.4.2.1.4 Preparation of oxazolidinone scaffold 8

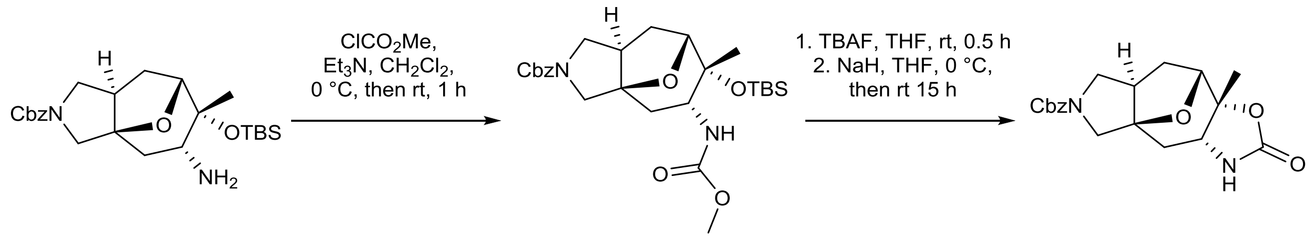

6 **S18** 63% **8** 29%

Benzyl (*1R,*5R**,*7R**,*8R**,*9R**)-8-[(*tert*-butyldimethylsilyl)oxy]-9-[(methoxycarbonyl)amino]-8-methyl-11-oxa-3-azatricyclo[5.3.1.0^{1,5}]undecane-3-carboxylate S18**

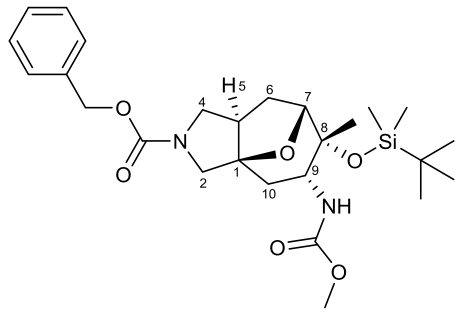

S18

Methyl chloroformate (2.1 mL, 28 mmol, 5.0 eq.) was added to a stirred solution of compound **6** (2.5 g, 5.5 mmol, 1.0 eq.) and Et_3N (3.8 mL, 28 mmol, 5.0 eq.) in CH_2Cl_2 (50 mL) at 0 °C. The reaction mixture was warmed to rt and stirred for 1 h. The reaction mixture was washed with sat. NH_4Cl solution (25 mL). The aqueous layer was extracted with CH_2Cl_2 (2 × 20 mL). The combined organic phases were washed with brine (25 mL), then dried over $MgSO_4$, filtered, and concentrated *in vacuo*. Flash chromatography eluting with 0-20% EtOAc in pentane gave the *title compound* **S18** (1.74 g, 3.44 mmol, 63%) as a colourless oil. **R*f*** 0.19 (4:1 pentane–EtOAc). **^{1}H NMR** (400 MHz, $CDCl_3$, 50:50 mixture of rotamers): δ 7.38-7.28 (5H, m, Cbz Ar-H), 5.50 (1H, s, NH), 5.10 (2H, s, OCH_2Ph), 4.00 (1H, d, *J* 7.3, 7-H), 3.93-3.79 (1H, m, 4-H_A), 3.77-3.60 (4H, m, includes: 1H, 2-H_A; and at δ 3.66: 3H, s, $NHCO_2CH_3$), 3.57-3.50 (1H, m, 9-H), 3.37 (0.5H, d, *J* 12.6, 2-H_B), 3.33 (0.5H, d, *J* 12.6, 2-H_B), 3.23-3.10 (1H, m, 4-H_B), 2.94-2.81 (1H, m, 5-H), 2.50-2.37 (1H, m, 6-H_A), 2.27-2.16 (1H, m, 10-H_A), 2.16-2.01 (1H, m, 10-H_B), 1.84-1.69 (1H, m, 6-H_B), 1.47 (3H, s, C_qCH_3), 0.91 (9H, s, $SiC(CH_3)_3$), 0.13 (3H, s, $SiCH_3$), 0.11 (3H, s, $SiCH_3$). **^{13}C NMR** (100 MHz, $CDCl_3$, mixture of two rotamers): δ 157.5 (NH(CO)O), 154.4 (N(CO)O), 137.0 (Ar-C_q), 128.6 (Ar-C), 128.1 (Ar-C), 128.0 (Ar-C), 90.0 (1-C), 89.1 (1-C), 84.2 (7-C), 71.8 (8-C), 66.9 (OCH_2Ph), 55.1 (2-C or 4-C), 55.0 (2-C or 4-C), 54.7 (2-C or 4-C), 54.6 (2-C or 4-C), 54.3 (9-C), 52.2 ($NHCO_2CH_3$), 43.9 (5-C), 43.0 (5-C), 34.4 (10-C), 33.1 (6-C), 32.9 (6-C), 27.5 (C_qCH_3), 26.0 ($SiC(CH_3)_3$), 18.4 (SiC_q), −1.7 ($SiCH_3$), −2.2 ($SiCH_3$) [27 of 42 expected peaks observed]. **IR** ν_{max}(film)/cm^{-1} 2953, 2884, 2857, 1728, 1707, 1419, 1055, 834. **HRMS** (ESI): $C_{26}H_{41}N_2O_6Si$ [M+H]$^+$; calculated 505.2728, found 505.2735.

Benzyl (*1R,*3R**,*7R**,*8R**,*10R**)-7-methyl-5-oxo-6,14-dioxa-4,12-diazatetracyclo[6.5.1.0^{1,10}.0^{3,7}]tetradecane-12-carboxylate 8**

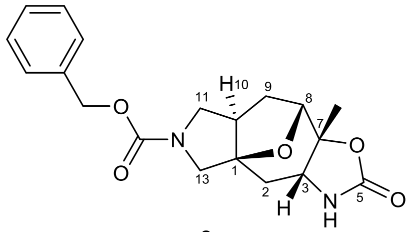

8

TBAF (1.0 M in THF, 6.7 mL, 6.7 mmol, 2.0 eq.) was added to a stirred solution of compound **S18** (1.70 g, 3.37 mmol, 1.00 eq) in THF (50 mL). The reaction mixture was stirred for 0.5 h then concentrated *in vacuo*. The residue was subjected to by SCX SPE following general procedure **A**, eluting with MeOH. The resulting residue was dissolved in DMF (50 mL), and NaH (60% dispersion in oil, 408 mg, 10.2 mmol, 3.0 eq.) was added at 0 °C. The reaction mixture was warmed to rt and stirred for 15 h. The reaction mixture was quenched by the addition of sat. aq. NH_4Cl solution (5 mL). The reaction mixture was diluted in EtOAc (50 mL) and washed with H_2O (50 mL). The aqueous layer was extracted with EtOAc (2 × 25 mL). The combined organic layers were dried over dried over $MgSO_4$, filtered, and concentrated *in vacuo*. Flash chromatography eluting with 98:2:0.1 CH_2Cl_2–EtOH–NH_3/MeOH gave the *title compound* **8** (356 mg, 0.99 mmol, 29%) as a colourless oil. **R_f** 0.30 (98:2:0.1 CH_2Cl_2–EtOH–NH_3/MeOH). **^{1}H NMR** (500 MHz, $CDCl_3$, 50:50 mixture of rotamers): δ 7.37-7.28 (5H, m, Cbz Ar-H), 6.01 (0.5H, s, NH), 5.91 (0.5H, s, NH), 5.11 (1H, 2 peaks, 2 × s, OCH_2Ph), 4.29 (1H, d, *J* 7.7, 8-H), 3.93-3.85 (1H, m, 11-H_A), 3.85-3.77 (2H, m, 3-H and 13-H_A), 3.39 (0.5H, d, *J* 12.6, 13-H_B), 3.33 (0.5H, d, *J* 12.6, 13-H_B), 3.30-3.17 (1H, m, 11-H_B), 2.88-2.78 (1H, m, 10-H), 2.45-2.34 (1H, m, 9-H_A), 2.17 (0.5H, app. dd, *J* 15.0, 5.7, 2-H_A), 2.11 (0.5H, app. dd, *J* 15.0, 5.7, 2-H_A), 1.94-1.80 (2H, m, includes: 1H, 9-H_B; and at δ 1.85: 1H, d, *J* 15.0, 2-H_B), 1.58 (3H, s, C_qCH_3). **^{13}C NMR** (125 MHz, $CDCl_3$, mixture of two rotamers): δ 159.4 (5-C), 154.5 (N(CO)O), 136.8 (Ar-C_q), 128.6 (Ar-C), 128.2 (Ar-C), 128.1 (Ar-C), 89.4 (1-C), 88.6 (1-C), 82.7 (8-C), 78.9 (7-C), 67.1 (OCH_2Ph), 55.4 (3-C), 55.2 (11-C or 13-C), 55.1 (11-C or 13-C), 54.8 (11-C or 13-C), 54.7 (11-C or 13-C), 45.1 (10-C), 44.1 (10-C), 34.0 (2-C), 33.4 (9-C), 33.1 (9-C), 24.7 (C_qCH_3) [22 of 34 expected peaks observed]. **IR** ν_{max}(film)/cm^{-1} 3282, 2939, 2884, 1759, 1701, 1421, 1109. **HRMS** (ESI): $C_{19}H_{23}N_2O_5$ [M+H]$^+$; calculated 359.1601, found 359.1603.

5.4.2.1.5 Preparation of pyrazine scaffold 9

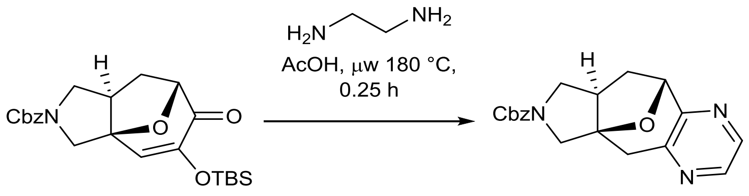

Benzyl (*1R*,9R*,11R)-15-oxa-4,7,13-triazatetracyclo[7.5.1.0^{1,11}.0^{3,8}]pentadeca-3(8),4,6-triene-13-carboxylate 9**

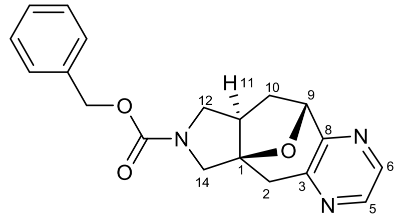

9

In two equally sized batches, ethylene diamine (2 × 0.55 mL, 2 × 8.20 mmol, 2 × 2.00 eq. [8.20 mmol in each batch, 16.4 mmol overall]) was added to a suspension of cycloadduct **2a** (2 × 1.75 g, 2 × 4.10 mmol, 2 × 1.0 eq. [4.10 mmol in each batch, 8.20 mmol overall]) in AcOH (2 × 5 mL [5 mL in each batch, 10 mL overall]). Each reaction mixture was stirred at rt for 10 mins, then heated at 180 °C under microwave irradiation for 15 min. The two batches were combined and concentrated *in vacuo*. The residue was diluted in EtOAc (50 mL) and washed with sat. aq. $NaHCO_3$ (50 mL). The aqeuous layer was extracted with EtOAc (4 × 20 mL). The combined organic layers were washed with H_2O (2 × 25 mL) and brine (25 mL), then dried over $MgSO_4$, filtered and concentrated *in vacuo*. Flash chromatography eluting with 98:2:0.1 CH_2Cl_2–EtOH–NH_3/MeOH gave the *title compound* **9** (1.1 g, 3.3 mmol, 40%) as a colourless amorphous solid. **R_f** 0.17 (98:2:0.1 CH_2Cl_2–EtOH–NH_3/MeOH). **^{1}H NMR** (400 MHz, $CDCl_3$): δ 8.41 (1H, d, *J* 2.6, 5-H or 6-H), 8.30 (1H, d, *J* 2.6, 5-H or 6-H), 7.41-7.28 (5H, m, Cbz Ar-H), 5.30 (1H, d, *J* 6.8, 9-H), 5.21-5.08 (2H, m, OCH_2Ph), 4.09 (1H, d, *J* 12.7, 14-H_A), 3.90 (1H, dd, *J* 11.4, 9.3, 12-H_B), 3.62-3.34 (3H, m, 2-H_A, 12-H_A and 14-H_B), 2.93 (1H, d, *J* 17.6, 2-H_B), 2.78-2.64 (1H, m, 11-H), 2.48-2.34 (1H, m, 10-H_A), 2.34-2.19 (1H, m, 10-H_B). **^{13}C NMR** (100 MHz, $CDCl_3$, mixture of two rotamers): δ 155.4 (8-C), 154.6 (N(CO)O), 149.3 (3-C), 143.6 (5-C or 6-C), 141.7 (5-C or 6-C), 136.8 (Ar-C_q), 128.6 (Ar-C), 128.2 (Ar-C), 128.1 (Ar-C), 91.2 (1-C), 90.3 (1-C), 80.6 (9-C), 67.2 (OCH_2Ph), 66.9 (OCH_2Ph), 55.1 (14-C), 54.7 (14-C), 54.4 (12-C), 54.1 (12-C), 46.7 (11-C), 45.7 (11-C), 43.4 (10-C), 43.2 (10-C), 39.5 (2-C) [23 of 34 expected peaks observed]. **IR** ν_{max}(film)/cm^{-1} 2953, 2880, 1703 (CO), 1420, 1349, 1190, 699. **HRMS** (ESI): $C_{19}H_{20}N_3O_3$ [M+H]$^+$; calculated 338.1499, found 338.1503.

5.4.2.1.6 Preparation of piperazine scaffolds S19-S20

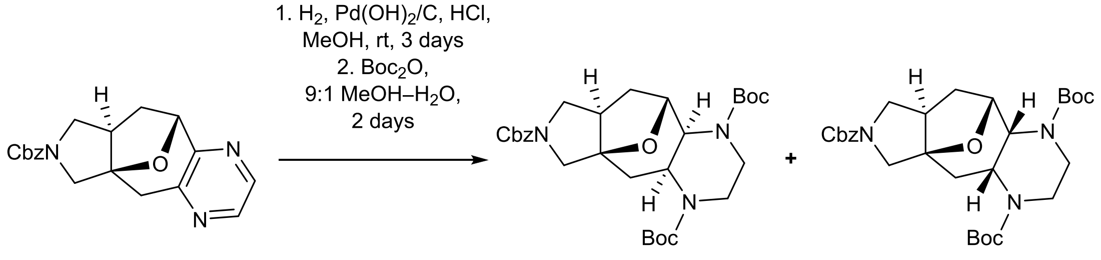

9 **S19** 8% **S20** 14%

4,7,13-Tri-*tert*-butyl (*1R,*3S**,*8S**,*9R**,*11R**)-15-oxa-4,7,13-triazatetracyclo[7.5.1.0^{1,11}.0^{3,8}]pentadecane-4,7,13-tricarboxylate S19 and 4,7,13-tri-*tert*-butyl (*1R**,*3R**,*8R**,*9R**,*11R**)-15-oxa-4,7,13-triazatetracyclo[7.5.1.0^{1,11}.0^{3,8}]pentadecane-4,7,13-tricarboxylate S20**

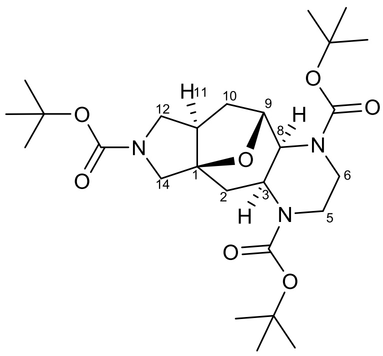

S19

+

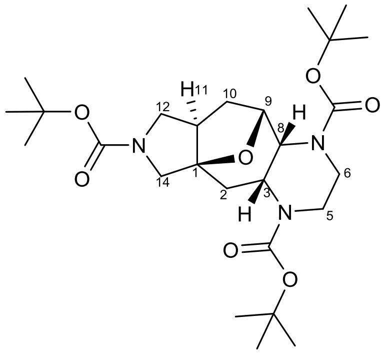

S20

Hydrogenation was carried out following general procedure **B**, using pyrazine **9** (1.1 g, 3.3 mmol, 1.0 eq.), $Pd(OH)_2/C$ (2 × 200 mg,[*] 20% w/w) and conc. HCl (12 M, 0.4 mL) in MeOH (20 mL) over 4 days. The residue was purified by SCX SPE following general procedure **A**, eluting first with MeOH, then sat. NH_3/MeOH to give a brown oil. The residue (404 mg, [estimate 1.90 mmol of the triamine, 1.00 eq.]) was dissolved in 9:1 MeOH–H_2O (25 mL) and Boc_2O (1.90 g, 8.70 mmol, 4.50 eq.) was added. The reaction mixture was stirred for 2 days, then concentrated *in vacuo*. Flash chromatography eluting with 4:6 pentane–EtOAc gave the *title compounds* **S19** (134 mg, 0.26 mmol, 8%, 2 steps) and **S20** (234 mg, 0.46 mmol, 14%) as colourless amorphous solids.

4,7,13-Tri-*tert*-butyl(*1R,*3S**,*8S**,*9R**,*11R**)-15-oxa-4,7,13 -triazatetracyclo[7.5.1.0^{1,11}.0^{3,8}]pentadecane-4,7,13-tricarboxylate S19:** **R*f*** 0.55 (4:6 pentane–EtOAc). **^{1}H NMR** (500 MHz, MeOD-d_4, 333 K): δ 4.77 (1H, d, *J* 8.1, 9-H), 4.65-4.48 (1H, m, 3-H), 4.20-4.07 (1H, m, 5-H_A), 3.82 (1H, dd, *J* 10.9, 9.9, 12-H_A), 3.70 (1H, app. d, *J* 7.1, 8-H), 3.65 (1H, d, *J* 12.6, 14-H_A), 3.55-3.40 (2H, m, 5-H_B and 6-H_A), 3.34-3.29 (2H, 6-H_B and 14-H_B), 3.03 (1H, dd, *J* 10.9, 7.9, 12-H_B), 2.80-2.57 (1H, m, 11-H), 2.11-1.89 (4H, m, 2-H and 10-H), 1.48 (9H, s, $NCO_2C(CH_3)_3$), 1.47 (9H, s,

---

[*] The reaction mixture was filtered and exposed to fresh catalyst after 3 days.

$NCO_2C(CH_3)_3$), 1.45 (9H, s, $NCO_2C(CH_3)_3$). **^{13}C NMR** (100 MHz, MeOD-d_4, 298 K, mixture of 2 rotamers): δ 156.9 (N(*C*O)O), 156.4 (N(*C*O)O), 155.9 (N(*C*O)O), 91.0 (1-C), 90.1 (1-C), 81.9 ($NCO_2C_q(CH_3)_3$), 81.8 ($NCO_2C_q(CH_3)_3$), 81.6 (9-C), 81.1 ($NCO_2C_q(CH_3)_3$), 56.3 (8-C), 56.2 (14-C), 56.1 (14-C), 55.7 (12-C), 55.3 (12-C), 45.0 (11-C), 44.1 (11-C), 43.8 (6-C), 43.7 (3-C), 43.3 (6-C), 39.2 (5-C), 37.4 (10-C), 36.9 (10-C), 34.6 (2-C), 34.5 (2-C), 28.7 ($NCO_2C(CH_3)_3$), 28.6 (2 peaks, 2 × $NCO_2C(CH_3)_3$) [27 of 40 expected peaks observed]. **IR** ν_{max}(film)/cm^{-1} 2974, 2932, 1683 (CO), 1392, 1364, 1157, 1112, 729. **HRMS** (ESI): $C_{26}H_{44}N_3O_7$ [M+H]$^+$; calculated 510.3174, found 510.3190.

4,7,13-Tri-*tert*-butyl(*1R,*3R**,*8R**,*9R**,*11R**)-15-oxa-4,7,13-triazatetracyclo[7.5.1.0^{1,11}.0^{3,8}]pentadecane-4,7,13-tricarboxylate S20:** **R_f** 0.46 (4:6 pentane–EtOAc). **^{1}H NMR** (500 MHz, $CHCl_3$, 330 K): δ 4.84 (1H, app. t, *J* 7.6, 9-H), 4.44 (1H, dd, *J* 17.3, 8.1, 3-H), 4.36 (1H, app. t, *J* 7.8, 8-H), 4.01 (1H, d, *J* 13.3, 6-H_A), 3.83 (1H, d, *J* 12.2, 14-H_A), 3.77 (1H, app. t, *J* 9.8, 12-H_A), 3.56 (1H, ddd, *J* 12.6, 10.8, 5.4, 5-H_A), 3.37 (1H, dt, *J* 12.6, 4.0, 5-H_B), 3.29-3.21 (2H, m, 6-H_B and 14-H_B), 3.15-3.06 (1H, m, 12-H_B), 2.50 (1H, qd, *J* 8.2, 3.0, 11-H), 2.39 (1H, dd, *J* 14.1, 7.8, 2-H_A), 2.26 (1H, dd, *J* 13.4, 8.4, 10-H_A), 1.82 (1H, dd, *J* 14.1, 9.3, 2-H_B), 1.78-1.70 (1H, m, 10-H_B), 1.48 (9H, s, $NCO_2C(CH_3)_3$), 1.48 (9H, s, $NCO_2C(CH_3)_3$), 1.45 (9H, s, $NCO_2C(CH_3)_3$). **^{13}C NMR** (100 MHz, $CHCl_3$, 298 K, mixture of 2 rotamers): δ 155.1 (N(CO)O), 154.8 (N(CO)O), 154.1 (N(CO)O), 88.6 (1-C), 87.8 (1-C), 80.7 ($NCO_2C_q(CH_3)_3$), 80.4 (9-C), 79.5 ($NCO_2C_q(CH_3)_3$), 78.5 ($NCO_2C_q(CH_3)_3$), 55.6 (12-C), 55.3 (12-C), 54.4 (14-C), 54.0 (14-C), 52.8 (8-C), 48.4 (11-C), 47.4 (11-C), 44.0 (3-C), 42.4 (5-C and 6-C), 35.7 (2-C), 31.0 (10-C), 30.6 (10-C), 28.5 ($NCO_2C(CH_3)_3$), 28.5 ($NCO_2C(CH_3)_3$), 28.4 ($NCO_2C(CH_3)_3$) [24 of 40 expected peaks observed]. **IR** ν_{max}(film)/cm^{-1} 2976, 2933, 2882, 1682, 1393, 1365, 1160, 1114, 909, 727. **HRMS** (ESI): $C_{26}H_{44}N_3O_7$ [M+H]$^+$; calculated 510.3174, found 510.3183.

5.4.2.1.7 Preparation of quinoxaline scaffold S21

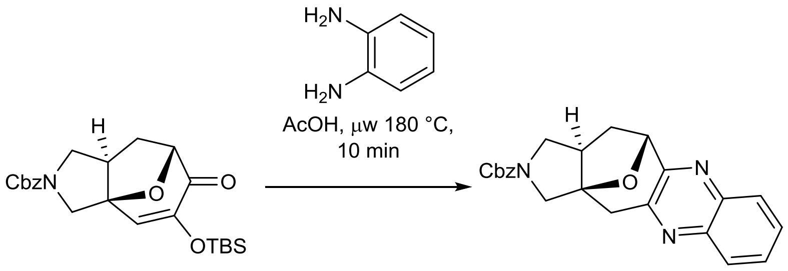

2a **S21** 89%

Benzyl (*1R,*13R**,*15R**)-19-oxa-4,11,17-triazapentacyclo [11.5.1.0^{1,15}.0^{3,12}.0^{5,10}]nonadeca-3,5(10),6,8,11-pentaene-17-carboxylate S21**

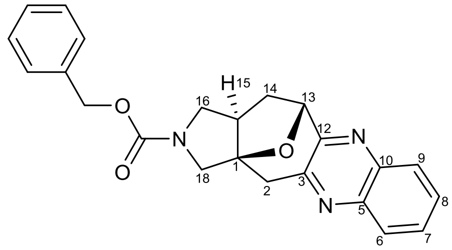

S21

1,2-Diaminobenzene (270 mg, 2.50 mmol, 1.10 eq.) was added to a stirred suspension of cycloadduct **2a** (1.0 g, 2.3 mmol, 1.0 eq.) in AcOH (10 mL). The reaction mixture was heated under microwave irradiation at 180 °C for 10 min. The reaction mixture was concentrated *in vacuo* then partitioned between CH_2Cl_2 (25 mL) and sat. aq. $NaHCO_3$ (25 mL). The aqeuous phase was extracted with CH_2Cl_2 (2 × 10 mL). The combined organic phases were dried, filtered and concentrated *in vacuo*. Flash chromatography eluting with 0-100% EtOAc in pentane gave the *title compound* **S21** (789 mg, 2.04 mol, 89%) as a colourless oil. **R_f** 0.51 (1:2 petrol–EtOAc). **^{1}H NMR** (500 MHz, $CDCl_3$): δ 8.02-7.97 (2H, m, 6-H and 9-H), 7.75-7.69 (2H, m, 7-H and 8-H), 7.42-7.30 (5H, m, Cbz Ar-H), 5.50 (1H, d, *J* 6.8, 13-H), 5.17 (1H, s, OCH_2Ph), 5.16 (1H, s, OCH_2Ph), 4.14 (1H, d, *J* 12.7, 18-H_A), 3.93 (1H, dd, *J* 11.3, 9.4, 16-H_A), 3.71-3.53 (2H, m, 2-H_A and 18-H_B), 3.53-3.37 (1H, m, 16-H_B), 3.15 (1H, d, *J* 17.8, 2-H_B), 2.83-2.70 (1H, m, 15-H), 2.49-2.30 (2H, m, 14-H). **^{13}C NMR** (125 MHz, $CDCl_3$, mixture of two rotamers): δ 154.9 (2 peaks, Ar-C_q), 154.7 (N(CO)O), 150.0 (Ar-C_q), 142.3 (Ar-C_q), 140.7 (Ar-C_q), 136.8 (Cbz Ar-C_q), 129.9 (2 peaks, 2 × Ar-C), 129.0 (Ar-C), 128.7 (2 peaks, 2 × Ar-C), 128.2 (2 peaks, 2 × Ar-C), 91.4 (1-C), 90.5 (1-C), 81.3 (13-C), 67.2 (OCH$_2$Ph), 55.3 (16-C or 18-C), 54.9 (16-C or 18-C), 54.5 (16-C or 18-C), 54.2 (16-C or 18-C), 46.6 (15-C), 45.6 (15-C), 42.8 (2-C), 42.4 (2-C), 40.4 (14-C) [27 of 42 expected peaks observed]. **IR** ν_{max}(film)/cm^{-1} 2952, 2884, 1702 (CO), 1421, 1358, 1274, 1112, 769. **HRMS** (ESI): $C_{23}H_{22}N_3O_3$ [M+H]$^+$; calculated 388.1656, found 388.1660.

5.4.2.2 Scaffolds derived from cycloadduct 2b

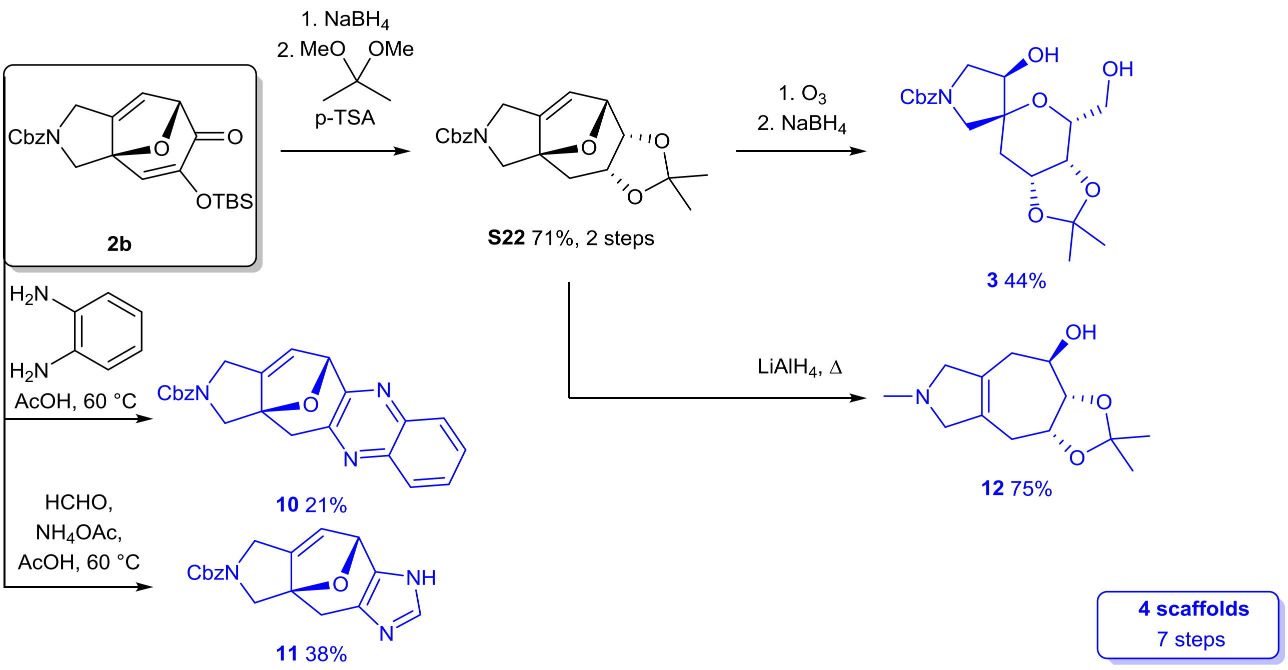

5.4.2.2.1 Preparation of spirocyclic scaffold 3 and scaffold 12

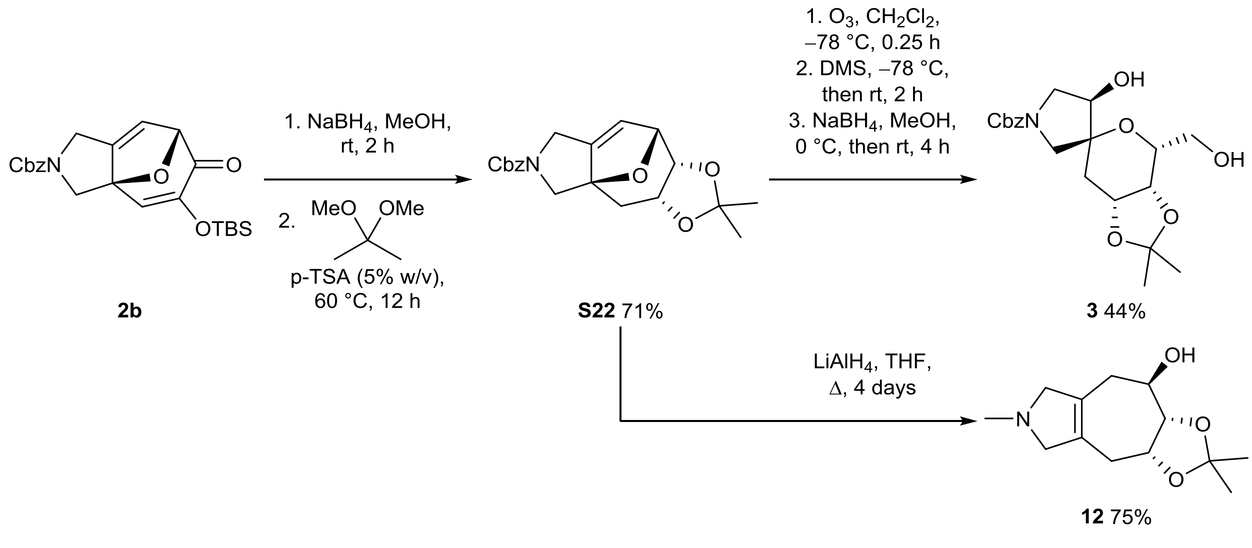

Benzyl (1*R,3*R**,7*R**,8*R**)-5,5-dimethyl-4,6,14-trioxa-12-azatetracyclo[6.5.1.0^{1,10}.0^{3,7}]tetradec-9-ene-12-carboxylate S22**

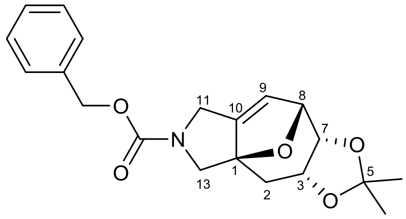

S22

$NaBH_4$ (0.196 g, 5.20 mmol, 2.0 eq) was added slowly over 10 min to a solution of cycloadduct **2b** (1.11 g, 2.60 mmol, 1.0 eq) in MeOH (43 mL) at room temperature (n.b the substrate is not soluble in cold solvent). The reaction mixture was stirred for 2 h before being quenched by the addition of H_2O (1.0 mL) and concentrated *in vacuo*. The resulting product was then suspended in 2,2-dimethoxy propane (43 mL) and to this was added p-toluenesulfonic acid (2.15 g, 5% w/v). The reaction mixture heated at 60 °C for 12 h before being diluted with EtOAc (50 mL) and washed with saturated aqueous $NaHCO_3$ (100 mL). The phases were separated and the aqueous phase extracted with EtOAc (50 mL). The combined organic phase was then washed with water (100 mL) and brine (100 mL), dried ($MgSO_4$), filtered and concentrated *in vacuo*. The crude product was purified by flash column chromatography (dry load), eluting with 65:35 petrol–EtOAc to furnish the *title compound* **S22** (662 mg, 1.85 mmol, 71%) as a pale yellow amorphous solid. **R_f** 0.30 (7:3 petrol–EtOAc). **^{1}H NMR** (500 MHz, $CDCl_3$): δ (500 MHz, DMSO-d_6, 343 K) 7.37-7.30 (5H, m, Cbz Ar-H), 5.94 (1H, app. s, 9-H), 5.10 (2H, s, OCH_2Ph), 4.90 (1H, dd, *J* 5.4, 1.5, 8-H), 4.52 (1H, app. t, *J* 6.8, 3-H), 4.18 (1H, dd, *J* 6.6, 5.7, 7-H), 4.09 (1H, d, *J* 14.4, 11-H_A), 3.90 (1H, d, *J* 14.4, 11-H_B), 3.50 (1H, d, *J* 10.9, 13-H_A), 3.29-3.24 (1H, m, 13-H_B), 2.16 (1H, dd, *J* 15.0, 6.8, 2-H_B), 1.94 (1H, app. d, *J* 15.0, 2-H_A), 1.32 (3H, s, acetonide CH_3), 1.23 (3H, s, acetonide CH_3). **^{13}C NMR** (125 MHz, DMSO-d_6, 343 K): δ 153.6 (N(CO)O), 136.5 (Ar-C_q), 127.9 (Ar-C), 127.3 (Ar-C), 126.9 (Ar-C), 122.1 (9-C), 107.7 (5-C), 85.7 (8-C), 71.6 (3-C), 68.1 (7-C), 65.7 (O*C*H_2Ph), 52.1 (13-C), 42.9 (11-C), 29.2 (2-C), 25.5 (C(*C*H_3)$_2$), 24.4 (C(*C*H_3)$_2$) – signals for 1-C and 10-C are not observed in DMSO-d_6 at 343 K due to rotameric effects; two signals were observed for each carbon on a spectrum recorded at 75 MHz in $CDCl_3$ at 300 K: 148.0 and 147.3 (10-C), 90.0 and 89.3 (1-C). **IR** ν_{max}(neat)/cm^{-1} 2936, 1710, 1409, 1353, 1214, 1136, 1122, 1069. **HRMS** (ESI): $C_{20}H_{23}NNaO_5$ $[M+Na]^+$; calculated 380.1468, found 380.1469. **X-Ray crystallography:** CCDC 1526779 contains the supplementary crystallographic data for this compound. Crystals were grown by slow evaporation of EtOH.

S22 Crystal structure:

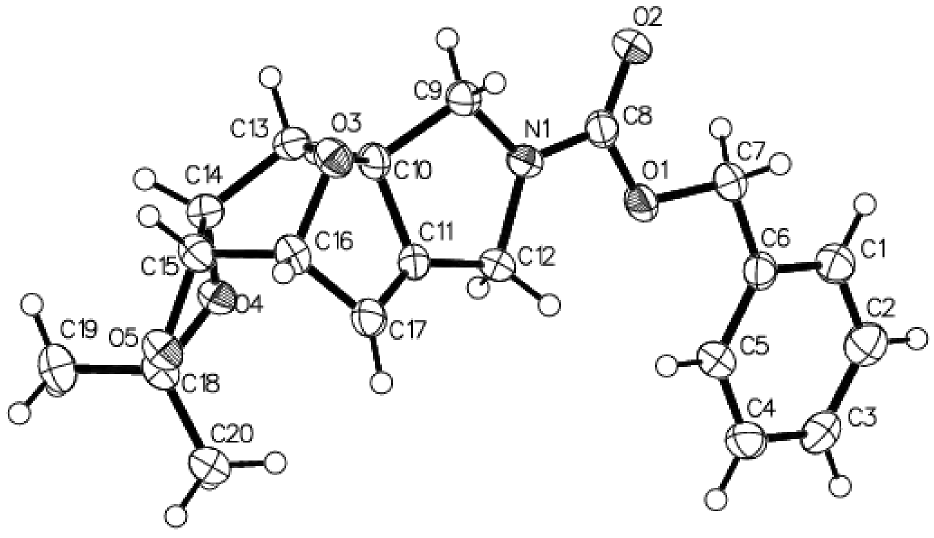

Benzyl (3a*R,4*R**,4'*S**,6*S**,7a*R**)-4'-hydroxy-4-(hydroxymethyl)-2,2-dimethyl-tetrahydro-2H-spiro[[1,3]dioxolo[4,5-c]pyran-6,3'-pyrrolidine]-1'-carboxylate 3**

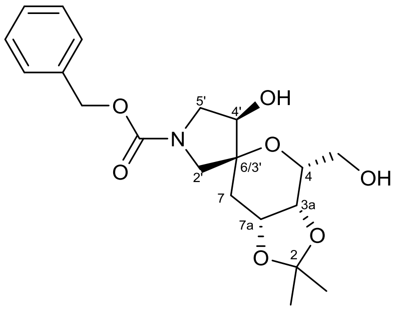

3

O_3 was bubbled through a solution of acetonide **S22** (200 mg, 0.56 mmol, 1.00 eq) in CH_2Cl_2 (6.0 mL) at –78 °C. After 15 min the solution turned blue in colour and O_3 was exchanged for N_2 for 20 min. Dimethyl sulfide (62.0 μL, 0.840 mmol, 1.5 eq) was then added at –78 °C. After 10 min the reaction mixture was warmed to rt and stirred for 2 h. After establishing no peroxides were present (starch iodine paper) the reaction mixture was concentrated *in vacuo* to furnish a keto-aldehyde that was used immediately with no further purification. $NaBH_4$ (63.0 mg, 1.68 mmol, 3.0 eq) was added to a solution of the crude keto-aldehyde in MeOH (6.0 mL) at 0 °C. The reaction mixture was warmed to rt with stirring over 4 h before being quenched by the addition of H_2O (0.10 mL) and concentrating *in vacuo*. The resulting crude product was suspended in 9:1 EtOAc–MeOH with sonication and filtered through celite. The filtrate was concentrated *in vacuo* and purified by flash chromatography, eluting with EtOAc to furnish the *title compound* **3** (103 mg, 0.25 mmol, 44%, *dr* 92:8) as a colourless waxy solid. **R_f** 0.31 (EtOAc). **^{1}H NMR** (500 MHz, MeOD-d_4, 333 K): δ 7.36-7.28 (5H, m, Cbz Ar-H), 5.11 (2H, s, OCH_2Ph), 4.49-4.45 (1H, m, 7a-H), 4.16 (1H, app. d, *J* 5.9, 3a-H), 4.02 (1H, app. t, *J* 5.9, 4'-H), 3.75-3.69 (3H, m, 4-H and CH_2OH), 3.65-3.59 (2H, m, 2'-H_A and 5'-H_A), 3.38-3.35 (2H, m, 2'-H_B and 5'-H_B), 1.94 (1H, dd, *J* 14.7, 6.2, 7-H_A), 1.86 (1H, dd, *J* 14.7, 4.5, 7-H_B), 1.47 (3H, s, acetonide CH_3), 1.32 (3H, s, acetonide CH_3). **^{13}C NMR** (125 MHz, MeOD-d_4, 333 K, mixture of two rotamers): δ 156.9 (N(CO)O), 138.2 (Ar-C_q), 129.5 (Ar-C), 129.0 (Ar-C), 128.8 (Ar-C), 110.2 (2-C), 80.7 (2 peaks, 6/3'-C), 76.9 (4'-C), 76.3 (4'-C), 74.0 (4-C), 73.5 (3a-C), 72.2 (7a-C), 68.2 (OCH$_2$Ph), 62.9 (CH$_2$OH), 52.9 (5'-C), 51.8 (2'-C), 51.6 (2'-C), 32.4 (7-C), 27.3 (acetonide CH$_3$), 25.6 (acetonide CH$_3$) [21 of 36 expected peaks observed]. **IR** ν_{max}(neat)/cm^{-1} 3423, 2934, 1695, 1420, 1355, 1213, 1090. **HRMS** (ESI): $C_{20}H_{27}NNaO_7$ $[M+Na]^+$; calculated 416.1679, found 416.1675.

3 NOESY correlations:
4'-H: 7-H_A and 7-H_B
7-H_A: 4'-H
7-H_B: 4'-H

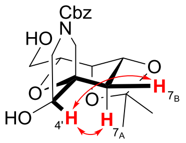

(3*R,7*S**,8*R**)-5,5,12-Trimethyl-4,6-dioxa-12-azatricyclo[8.3.0.0^{3,7}]tridec-1(10)-en-8-ol 12 (≡ F12)**

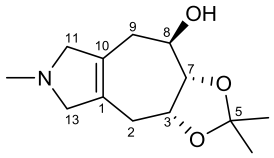

12 ≡ F12

$LiAlH_4$ (1.0 M solution in THF, 5.60 mL, 5.60 mmol, 10.0 eq) was added drop-wise over 15 min to a solution of acetonide **S22** (200 mg, 0.56 mmol, 1.00 eq) in THF (4.0 mL) at rt. Upon completion of addition the reaction mixture was heated at reflux for 4 days before being cooled rt and quenched by the sequential slow addition of H_2O (0.2 mL), 2 M aqueous NaOH (0.2 mL) and water (0.6 mL). The resulting suspension was stirred vigorously for 2 h before being diluted with MeOH (20 mL), filtered through celite and concentrated *in vacuo*. The crude product was purified by SCX SPE to furnish the *title compound* **12** (≡ **F12**: 100 mg, 0.42 mmol, 75%) as an off-white waxy solid. **R_f** 0.34 (9:1 CH_2Cl_2–sat. NH_3/MeOH). **^{1}H NMR** (500 MHz, $CDCl_3$): δ 4.29 (1H, ddd, *J* 11.3, 6.8, 4.3, 3-H), 4.12 (1H, ddd, *J* 11.1, 9.0, 3.5, 8-H), 4.07 (1H, dd, *J* 9.0, 6.8, 7-H), 3.47-3.35 (4H, m, 11-H and 13-H), 2.41 (3H, s, NCH_3), 2.39-2.31 (2H, m, 2-H_A and 9-H_B), 2.20 (1H, dd, *J* 11.1, 3.5, 9-H_A), 2.13 (1H, dd, *J* 15.4, 4.3, 2-H_B), 1.47 (3H, s, acetonide C*H*$_3$), 1.35 (3H, s, acetonide C*H*$_3$). **^{13}C NMR** (125 MHz, $CDCl_3$) 130.9 (1-C or 10-C), 128.4 (1-C or 10-C), 108.3 (5-C), 82.2 (8-C), 74.9 (3-C), 68.3 (7-C), 66.6 (11-C or 13-C), 66.4 (11-C or 13-C), 42.0 (N*C*H_3), 33.0 (9-C), 28.6 (2-C), 27.8 (C(*C*H_3)$_2$), 25.0 (C(*C*H_3)$_2$). **IR** ν_{max}(neat)/cm^{-1} 3340, 2922, 2772, 1450, 1375, 1240, 1211, 1167, 1142, 1114, 1069, 1031. **HRMS** (ESI) $C_{13}H_{22}NO_3$ [M+Na]$^+$; calculated 240.1594, found 240.1595.

5.4.2.2.2 Preparation of quinoxaline scaffold 10

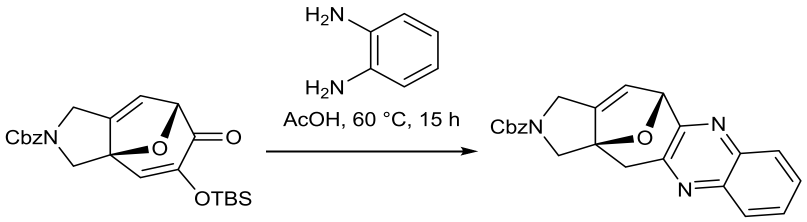

2b **10** 21%

Benzyl (*1R,*13R**)-19-oxa-4,11,17-triazapentacyclo[11.5.1.0^{1,15}.0^{3,12}.0^{5,10}]nonadeca-3,5(10),6,8,11,14-hexaene-17-carboxylate 10**

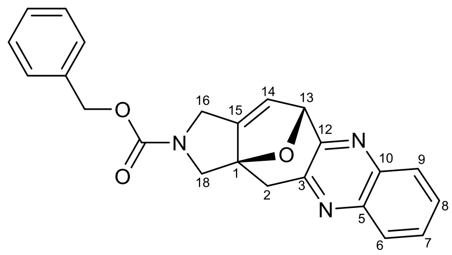

10

1,2-Diaminobenzene (28 mg, 0.26 mmol, 1.1 eq.) was added to a stirred suspension of cycloadduct **2b** (100 mg, 0.23 mmol, 1.00 eq.) in AcOH (3 mL). The reaction mixture was heated in a sealed tube at 60 °C for 15 h.* The reaction mixture was concentrated *in vacuo* then partitioned between CH_2Cl_2 (10 mL) and sat. aq. $NaHCO_3$ (10 mL). The aqeuous phase was extracted with CH_2Cl_2 (2 × 10 mL). The combined organic phases were dried over $MgSO_4$, filtered and concentrated *in vacuo*. Flash chromatography eluting with 4:1 pentane–EtOAc gave the *title compound* **10** (19 mg, 49 μmol, 21%) as a colourless oil. **R_f** 0.07 (4:1 petrol–EtOAc). **^{1}H NMR** (500 MHz, $CDCl_3$, 50:50 mixture of rotamers): δ 8.02-7.95 (2H, m, 6-H and 9-H), 7.76-7.65 (2H, m, 7-H and 8-H), 7.45-7.29 (5H, m, Cbz Ar-H), 6.37 (0.5H, app. s, 14-H), 6.33 (0.5H, app. s, 14-H), 5.82 (1H, app. s, 13-H), 5.25-5.14 (2H, m, OCH_2Ph), 4.25 (1H, app. dd, *J* 16.1, 11.5, 16-H_A), 4.12 (1H, app. dd, *J* 16.1, 8.6, 16-H_B), 3.97 (0.5H, d, *J* 11.2, 18-H_A), 3.90 (0.5H, d, *J* 11.2, 18-H_B), 3.71 (1H, d, *J* 11.2, 18-H_B), 3.59 (1H, app. t, *J* 18.1, 2-H_A), 3.19 (1H, d, *J* 18.1, 2-H_B). **^{13}C NMR** (125 MHz, $CDCl_3$, mixture of two rotamers): δ 155.0 (N(CO)O), 154.8 (N(CO)O), 152.9 (Ar-C_q), 151.4 (Ar-C_q), 151.3 (Ar-C_q), 147.4 (Ar-C_q or 15-C), 146.7 (Ar-C_q or 15-C), 142.1 (Ar-C_q), 139.9 (Ar-C_q), 136.6 (Ar-C_q), 129.8 (Ar-C), 129.6 (Ar-C), 128.9 (Ar-C), 128.7 (Ar-C), 128.6 (Ar-C), 128.4 (Ar-C), 128.2 (Ar-C), 128.2 (Ar-C), 126.5 (2 peaks, 14-C), 91.7 (1-C), 90.9 (1-C), 89.1 (13-C), 67.5 (OCH_2Ph), 53.5 (18-C), 43.6 (16-C), 43.4 (16-C), 37.6 (2-C), 37.5 (2-C) [28 of 42 expected peaks observed]. **IR** ν_{max}(film)/cm^{-1} 2928, 1704, 1415, 1358, 1343, 1126, 1100, 764. **HRMS** (ESI): $C_{23}H_{20}N_3O_3$ $[M+H]^+$; calculated 386.1499, found 386.1499.

---

* N.b. under μW irradiation this reaction mainly gave decomposition products at 180 °C (as judged by analysis of the crude reaction product by ^{1}H NMR spectroscopy at 300 MHz).

5.4.2.2.3 Preparation of imidazole scaffold 11

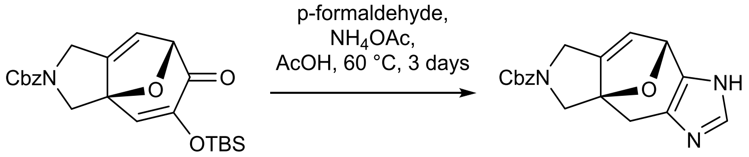

2b **11**
38%

Benzyl (*1R,*8R**)-14-oxa-4,6,12-triazatetracyclo[6.5.1.0^{1,10}.0^{3,7}]tetradeca-3(7),4,9-triene-12-carboxylate 11**

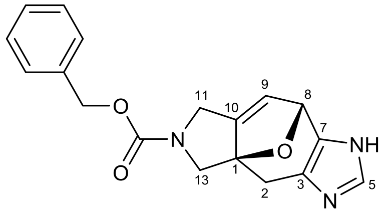

11

p-Formaldehyde (15 mg, 0.50 mmol, 2.0 eq.) and NH_4OAc (177 mg, 2.29 mmol, 10.0 eq.) were added to a suspension of cycloadduct **2b** (100 mg, 0.23 mmol, 1.0 eq.) in AcOH (3.0 mL). The reaction mixture was heated in a sealed tube at 60 °C for 3 days.* The reaction mixture was concentrated *in vacuo*, then partitioned between EtOAc (10 mL) and $NaHCO_3$ (10 mL). The phases were separated and the aqueous phase was extracted with EtOAc (2 × 10 mL). The combined organic extracts were dried over $MgSO_4$, filtered and concentrated *in vacuo*. Flash chromatography eluting with 90:9:1 CH_2Cl_2–EtOH–NH_3/MeOH gave the *title compound* **11** (28 mg, 87 μmol, 38%) as a colourless foam. **R_f** 0.15 (90:9:1 CH_2Cl_2–EtOH–NH_3/MeOH). **^{1}H NMR** (500 MHz, $CDCl_3$, imidazole NH not observed): δ 7.43-7.29 (6H, m, 5-H and Cbz Ar-H), 6.45 (0.5H, app. s, 9-H), 6.43 (0.5H, app. s, 9-H), 5.65 (1H, app. s, 8-H), 5.21-5.11 (2H, m, OC*H*$_2$Ph), 4.16 (1H, app. dd, *J* 15.8, 6.1, 11-H_A), 4.08 (1H, app. ddd, *J* 15.8, 4.2, 2.2, 11-H_B), 3.87 (0.5H, d, *J* 11.2, 13-H_A), 3.79 (0.5H, d, *J* 11.2, 13-H_A), 3.62 (1H, app. dd, *J* 11.2, 3.1, 13-H_B), 3.19 (1H, app. t, *J* 16.2, 2-H_A), 2.75 (1H, d, *J* 16.2, 2-H_B). **^{13}C NMR** (125 MHz, $CDCl_3$, mixture of two rotamers, 2 × imidazole C_q not observed): δ 155.1 (N(CO)O), 154.9 (N(CO)O), 146.0 (10-C), 136.7 (Ar-C_q), 132.1 (Ar-C), 131.4 (Ar-C), 128.7 (Ar-C), 128.3 (Ar-C), 128.2 (Ar-C), 91.2 (1-C), 90.4 (1-C), 84.8 (9-C), 77.3 (8-C), 67.4 (O*C*H_2Ph), 53.9 (13-C), 43.5 (11-C), 43.4 (11-C), 30.2 (2-C), 30.1 (2-C) [19 of 32 expected peaks observed]. **IR** ν_{max}(film)/cm^{-1} 2957, 1701 (CO), 1417, 1358, 1216, 1115, 733. **HRMS** (ESI): $C_{18}H_{18}N_3O_3$ [M+H]$^+$; calculated 324.1343, found 324.1342.

* N.b. **under μW irradiation** this reaction mainly gave decomposition products at 180 °C (as judged by analysis of the crude reaction product by ^{1}H NMR spectroscopy at 300 MHz).

5.4.2.3 Scaffolds derived from cycloadduct 2c

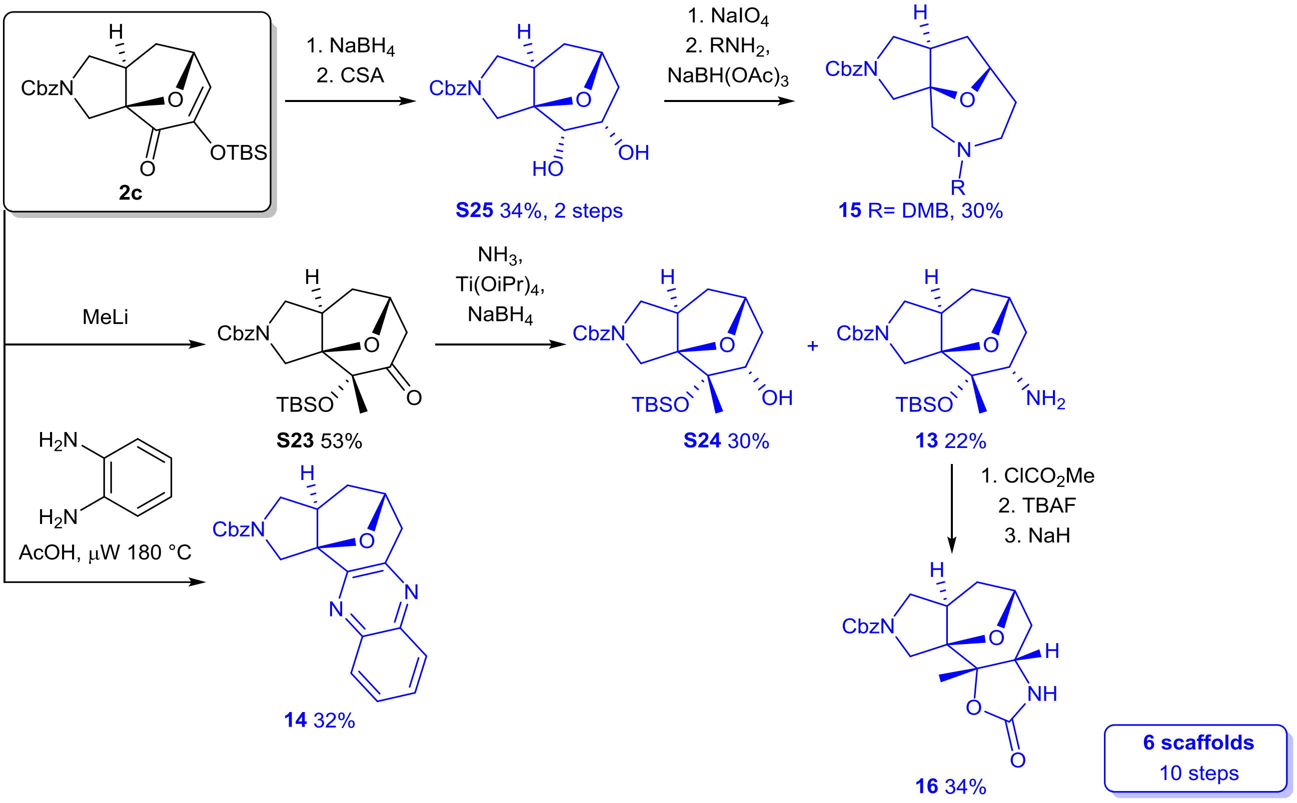

5.4.2.3.1 Preparation of silyl-protected amino alcohol scaffold 13 and alcohol S24

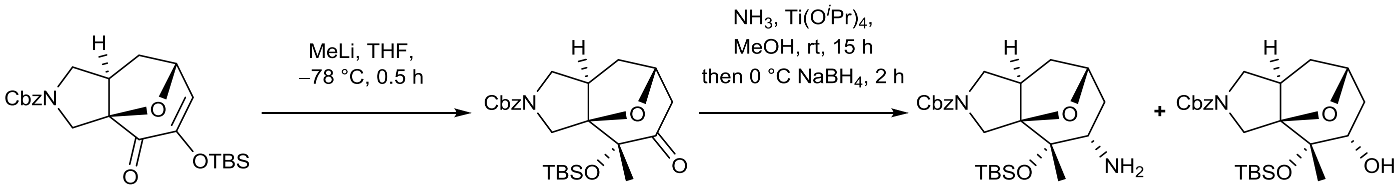

Benzyl (*1R*,5R*,7R*,10R)-10-[(*tert*-butyldimethylsilyl)oxy]-10-methyl-9-oxo-11-oxa-3-azatricyclo[5.3.1.0^{1,5}]undecane-3-carboxylate S23**

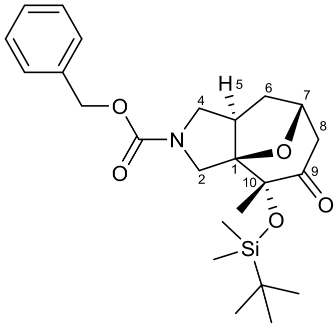

S23

MeLi (1.6 M in Et_2O, 5.9 mL, 9.5 mmol, 1.2 eq.) was added to a stirred solution of cycloadduct **2c** (3.4 g, 7.9 mmol, 1.0 eq.) in THF (125 mL) at −78 °C. The reaction mixture was stirred at this temperature for 1 h, then warmed to rt. H_2O (5 mL) was added then the reaction mixture was concentrated *in vacuo*. The residue was diluted in EtOAc (50 mL) and washed with brine (50 mL). The aqueous phase was extracted with EtOAc (2 × 25 mL). The combined organic extracts were dried, filtered and concentrated *in vacuo*. Flash chromatography eluting with 4:1 pentane–EtOAc gave the *title compound* **S23** (1.86 g, 4.20 mmol, 53%) as a colourless oil. **R_f** 0.10 (4:1 petrol–EtOAc). **^{1}H NMR** (500 MHz, $CHCl_3$, 50:50 mixture of rotamers): δ 7.39-7.27 (5H, m, Cbz Ar-H), 5.13 (2H, s, OCH_2Ph), 4.79-4.72 (1H, m, 7-H), 3.95-3.65 (3H, m, 2-H and 4-H_A), 3.27-3.18 (1H, m, 5-H), 2.90-2.72 (2H, m, 4-H_B and 8-H_B), 2.29 (1H, d, *J* 15.0, 8-H_A), 2.01-1.87 (2H, m, 6-H), 1.44 (1.5H, s, C_qCH_3), 1.40 (1.5H, s, C_qCH_3), 0.85 (4.5H, s, $SiC_q(CH_3)_3$), 0.83 (4.5H, s, $SiC_q(CH_3)_3$), 0.17 (3H, s, $(SiCH_3)_A$), 0.16 (1.5H, s, $(SiCH_3)_B$), 0.15 (1.5H, s, $(SiCH_3)_B$). **^{13}C NMR** (125 MHz, $CHCl_3$, mixture of two rotamers, 1-C not observed): δ 207.8 (9-C), 154.6 (N(CO)O), 137.0 (Ar-C_q), 128.6 (Ar-C), 128.1 (Ar-C), 127.1 (Ar-C), 81.7 (10-C), 77.1 (7-C), 67.1 (OCH_2Ph), 54.5 (2-C or 4-C), 54.2 (2-C or 4-C), 50.5 (2-C or 4-C), 50.1 (2-C or 4-C), 47.2 (8-C), 40.9 (5-C), 37.9 (6-C), 37.7 (6-C), 26.0 ($SiC_q(CH_3)_3$), 22.2 (C_qCH_3), 18.7 (SiC_q), −2.6 ($SiCH_3$) [21 of 38 expected peaks observed]. **IR** ν_{max}(film)/cm^{-1} 2953, 2930, 2883, 2855, 1707 (CO), 1418, 1359, 1345. **HRMS** (ESI): $C_{24}H_{36}NO_5Si$ [M+H]$^+$; calculated 446.2357, found 446.2360.

Benzyl (*1R,*5R**,*7R**,*9S**,*10S**)-9-amino-10-[(*tert*-butyldimethylsilyl)oxy]-10-methyl-11-oxa-3-azatricyclo[5.3.1.0^{1,5}]undecane-3-carboxylate 13 and benzyl (*1R**,*5R**,*7R**,*9S**,*10S**)-10-[(*tert*-butyldimethylsilyl)oxy]-9-hydroxy-10-methyl-11-oxa-3-azatricyclo[5.3.1.0^{1,5}]undecane-3-carboxylate S24**

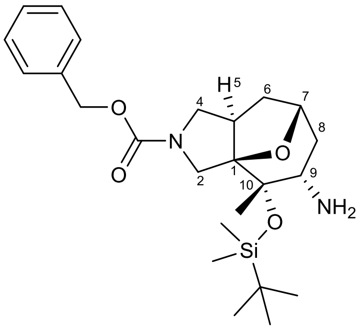

13

+

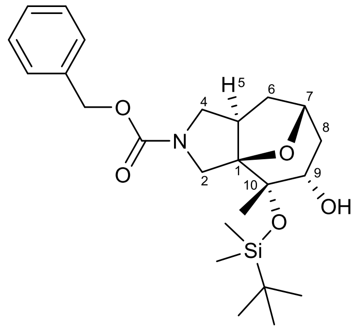

S24

Ti(O^{i}Pr)$_4$ (2.4 mL, 8.1 mmol, 2.0 eq.) was added to a stirred solution of ketone **S23** (1.8 g, 4.1 mmol, 1.0 eq.) in sat. NH_3/MeOH (50 mL). The reaction mixture was stirred for 15 h then $NaBH_4$ (230 mg, 6.09 mmol, 1.5 eq.) was added at 0 °C. The reaction mixture was warmed to rt, stirred for 2 h then concentrated *in vacuo*.* The residue was diluted in EtOAc (50 mL) and sat. aq. brine (50 mL) was added and the mixture was stirred vigorously. The phases were separated and the aqueous phase was extracted with EtOAc (2 × 25 mL). The combined organic phase was washed with H_2O (25 mL), then dried over $MgSO_4$, filtered, and concentrated *in vacuo*. Flash chromatography eluting with 98:2:0.1 CH_2Cl_2–EtOH–NH_3/MeOH gave the *title compounds* **13** (416 mg, 0.93 mmol, 22%) and **S24** (542 mg, 1.21 mmol, 30%) as colourless oils.

Benzyl (*1R,*5R**,*7R**,*9S**,*10S**)-9-amino-10-[(*tert*-butyldimethylsilyl) oxy]-10-methyl-11-oxa-3-azatricyclo[5.3.1.0^{1,5}]undecane-3-carboxylate 13** **R_f** 0.10 (98:2:0.1 CH_2Cl_2–EtOH–NH_3/MeOH). **^{1}H NMR** (500 MHz, $CHCl_3$, 50:50 mixture of rotamers, NH_2 not observed) δ 7.38-7.28 (5H, m, Cbz Ar-H), 5.18-5.05 (2H, m, OCH_2Ph), 4.50-4.44 (1H, m, 7-H), 3.93 (0.5H, app. t, *J* 10.2, 4-H_A), 3.86 (0.5H, app. t, *J* 10.2, 4-H_A), 3.74 (1H, app. t, *J* 12.5, 2-H_A), 3.59-3.44 (2H, m, includes: 1H, 9-H; and at δ 3.56: 0.5H, d, *J* 11.9, 2-H_B; and at δ 3.48: 0.5H, d, *J* 11.9, 2-H_B), 3.20 (1H, dd, *J* 10.2, 7.9, 4-H_B), 3.16 (1H, app. t, *J* 5.6, 5-H), 2.58 (1H, dd, *J* 11.9, 8.9, 8-H_B), 2.14-2.05 (1H, m, 6-H_A), 1.85-1.73 (1H, m, 8-H_A), 1.59-1.42 (1H, m, 6-H_B), 1.38 (1.5H, s, C_qCH_3), 1.34 (1.5H, s, C_qCH_3), 0.90 (4.5H, s, $SiC_q(CH_3)_3$), 0.88 (4.5H, s, $SiC_q(CH_3)_3$), 0.19 (1.5H, s, $SiCH_3$), 0.16 (1.5H, s, $SiCH_3$), 0.15 (1.5H, s, $SiCH_3$), 0.13 (1.5H, s, $SiCH_3$). **^{13}C NMR** (100 MHz, $CHCl_3$, mixture of two rotamers): δ 154.6 (N(CO)O), 137.3 (Ar-C_q), 128.6 (Ar-C), 128.0 (2 peaks, Ar-C), 127.9 (Ar-C), 95.4 (1-C), 94.4 (1-C), 78.1 (7-C), 77.9 (7-C), 74.8 (10-C), 74.7 (10-C), 66.8 (O*C*H_2Ph), 55.7 (9-C), 55.1 (4-C), 54.8 (4-C), 51.6 (2-C), 51.1 (2-C), 41.2 (5-C), 40.4 (5-C), 38.6 (8-C), 38.4 (8-C), 36.8 (6-C), 26.0 (2 peaks, $SiC_q(CH_3)_3$), 25.0 (C_qCH_3), 18.6 (SiC_q), −1.6 ($SiCH_3$), −2.3 ($SiCH_3$) [29 of 38 expected

* Analysis of the crude reaction product using ^{1}H NMR at 300 MHz suggested that a 1:1 mixture of **13**:**S24** was formed.

peaks observed]. **IR** ν_{max}(film)/cm^{-1} 2951, 2931, 2883, 2856, 1704 (CO), 1419, 1160, 937, 773. **HRMS** (ESI): $C_{24}H_{39}N_2O_4Si$ [M+H]$^+$; calculated 447.2674, found 447.2680.

Benzyl (*1R,*5R**,*7R**,*9S**,*10S**)-10-[(*tert*-butyldimethylsilyl)oxy]-9-hydroxy-10-methyl-11-oxa-3-azatricyclo[5.3.1.01,5]undecane-3-carboxylate S24** **R*f*** 0.28 (98:2:0.1 CH_2Cl_2–EtOH–NH_3/MeOH). **^{1}H NMR** (500 MHz, $CHCl_3$, 50:50 mixture of rotamers, OH not observed): δ 7.39-7.27 (5H, m, Cbz Ar-H), 5.12 (2H, s, OC*H*$_2$Ph), 4.52-4.44 (1H, m, 7-H), 3.99-3.89 (1H, m, 4-H_A), 3.87 (0.5H, d, *J* 12.1, 2-H_A), 3.82 (0.5H, d, *J* 12.1, 2-H_A), 3.69 (1H, d, *J* 4.3, 9-H), 3.60 (0.5H, d, *J* 12.1, 2-H_B), 3.54 (0.5H, d, *J* 12.1, 2-H_B), 3.25-3.05 (2H, m, 4-H_B and 5-H), 2.44-2.35 (1H, m, 8-H_B), 2.19-2.10 (1H, m, 6-H_A), 1.86-1.74 (1H, m, 6-H_B), 1.68 (1H, d, *J* 14.3, 8-H_A), 1.29 (1.5H, s, C_qCH_3), 1.26 (1.5H, s, C_qCH_3), 0.94 (4.5H, s, $SiC_q(CH_3)_3$), 0.94 (4.5H, s, $SiC_q(CH_3)_3$), 0.12 (6H, s, 2 × $SiCH_3$). **^{13}C NMR** (125 MHz, $CHCl_3$, mixture of two rotamers, N(*C*O)O not observed): 137.2 (Ar-C_q), 128.6 (Ar-C), 128.1 (Ar-C), 128.0 (Ar-C), 95.4 (1-C), 76.8 (2 peaks, 7-C), 74.5 (9-C), 70.0 (10-C), 69.9 (10-C), 67.0 (O*C*H_2Ph), 66.9 (O*C*H_2Ph), 55.2 (4-C), 54.9 (4-C), 51.2 (2-C), 50.7 (2-C), 40.7 (5-C), 39.8 (5-C), 37.3 (8-C), 37.0 (6-C), 25.9 ($SiC_q(CH_3)_3$), 22.4 (C_qCH_3), 18.1 (SiC_q), −4.3 ($SiCH_3$), −5.3 ($SiCH_3$) [25 of 38 expected peaks observed]. **IR** ν_{max}(film)/cm^{-1} 2951, 2930, 2885, 2856, 1703 (CO), 1419, 1085, 1072. **HRMS** (ESI): $C_{24}H_{38}NO_5Si$ [M+H]$^+$; calculated 448.2514, found 448.2518.

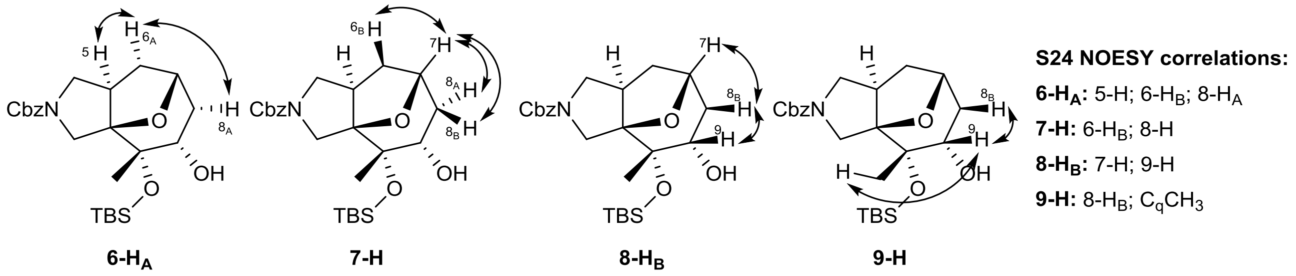

5.4.2.3.2 Preparation of quinoxaline scaffold 14

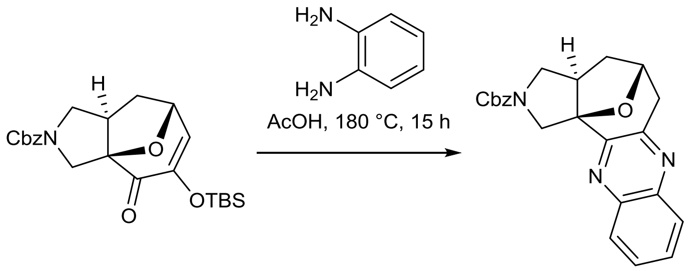

2c **14** 32%

Benzyl (*1R,*13R**,*15R**)-19-oxa-3,10,17-triazapentacyclo[11.5.1.0^{1,15}.0^{2,11}.0^{4,9}]nonadeca-2(11),3,5,7,9-pentaene-17-carboxylate 14**

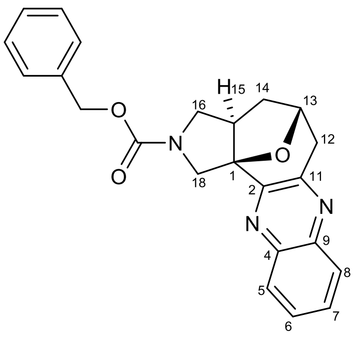

14

1,2-Diaminobenzene (270 mg, 2.50 mmol, 1.10 eq.) was added to a stirred suspension of cycloadduct **2c** (1.0 g, 2.3 mmol, 1.0 eq.) in AcOH (10 mL). The reaction mixture was heated under microwave irradiation at 180 °C for 10 min. The reaction mixture was concentrated *in vacuo* then partitioned between CH_2Cl_2 (25 mL) and sat. aq. $NaHCO_3$ (25 mL). The aqeuous phase was extracted with CH_2Cl_2 (2 × 10 mL). The combined organic phases were dried, filtered and concentrated *in vacuo*. Flash chromatography eluting with 1:1 pentane–EtOAc in pentane gave the *title compound* **14** (281 mg, 0.73 mmol, 32%) as an amorphous orange solid. **R*f*** 0.61 (4:1 petrol–EtOAc). **^{1}H NMR** (500 MHz, $CDCl_3$, 50:50 mixture of rotamers): δ 8.04-7.94 (2H, m, 5-H and 8-H), 7.77-7.64 (2H, m, 6-H and 7-H), 7.48-7.30 (5H, m, Cbz Ar-H), 5.23-5.15 (2H, m, OCH_2Ph), 5.12 (1H, app. t, *J* 6.6, 13-H), 5.04 (0.5H, d, *J* 12.8, 18-H_B), 4.96 (0.5H, d, *J* 12.8, 18-H_B), 4.14-4.02 (1H, m, 18-H_A), 3.99 (1H, dd, *J* 12.7, 7.8, 16-H_B), 3.70 (1H, dd, *J* 18.0, 5.3, 12-H_B), 3.40-3.26 (1H, m, 16-H_A), 3.04-2.91 (2H, m, 12-H_A and 15-H), 2.37-2.21 (1H, m, 14-H_B), 2.12-2.00 (1H, m, 14-H_A). **^{13}C NMR** (125 MHz, $CDCl_3$, mixture of two rotamers): 154.8 (N(CO)O), 152.2 (Ar-C_q), 150.6 (Ar-C_q), 142.3 (Ar-C_q), 140.5 (Ar-C_q), 137.0 (Ar-C_q), 130.1 (Ar-C), 129.7 (Ar-C), 129.4 (Ar-C), 129.3 (Ar-C), 128.6 (Ar-C), 128.5 (Ar-C), 128.1 (Ar-C), 92.9 (1-C), 92.2 (1-C), 76.5 (13-C), 67.1 (OCH_2Ph), 54.1 (16-C), 53.7 (16-C), 51.8 (15-C), 50.9 (15-C), 50.8 (18-C), 50.3 (18-C), 40.6 (12-C), 36.3 (14-C), 36.1 (14-C) [26 of 42 expected peaks observed]. **IR** ν_{max}(film)/cm^{-1} 2954, 2888, 1701 (CO), 1447, 1359, 1348, 1319, 1116. **HRMS** (ESI): $C_{23}H_{21}N_3O_3$ $[M+H]^+$; calculated 388.1656, found 388.1653.

5.4.2.3.3 Preparation of diol S25 and cyclic amine 15

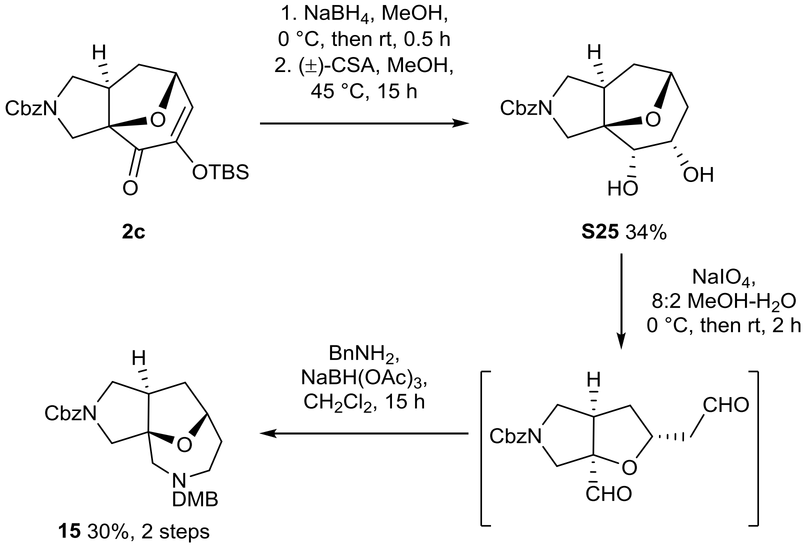

Benzyl (*1R,*5R**,*7R**,*9S**,*10S**)-9,10-dihydroxy-11-oxa-3-azatricyclo[5.3.1.0^{1,5}]undecane-3-carboxylate S25**

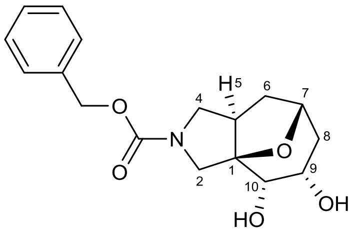

S25

$NaBH_4$ (1.40 g, 35.8 mmol, 2.20 eq.) was added to a stirred solution of cycloadduct **2c** (7.0 g, 16.3 mmol, 1.0 eq.) in MeOH (100 mL) at 0 °C. The reaction mixture was stirred at 0 °C for 5 min, warmed to rt, stirred for 1 h, then concentrated *in vacuo*. The residue was diluted in CH_2Cl_2 (100 mL) and washed with 1N HCl (100 mL). The aqueous phase was extracted with CH_2Cl_2 (50 mL). The organic extracts were combined, washed with brine (50 mL), dried over Na_2SO_4 and concentrated *in vacuo*. The residue (16.3 mmol) was dissolved in MeOH (100 mL) and (±)-camphorsulfonic acid (4.90 g, 21.2 mmol, 1.30 eq.) was added. The reaction mixture was heated at 45 °C for 15 h, then concentrated *in vacuo*. The residue was diluted in CH_2Cl_2 (50 mL). Saturated aqueous $NaHCO_3$ (50 mL) and H_2O (50 mL) were added and the phases were separated. The aqueous phase was extracted using CH_2Cl_2 (2 × 50 mL). The organic extracts were combined, dried over Na_2SO_4 and concentrated *in vacuo*. Flash chromatography eluting with 100% EtOAc gave the *title compound* **S25** (1.75 g, 5.48 mmol, 34%, 2 steps) as a pale brown oil. **R_f** 0.08 (2:3 pentane–EtOAc). **^{1}H NMR** (500 MHz, $CDCl_3$, 50:50 mixture of rotamers, 2 × OH not observed): δ 7.38-7.27 (5H, m, Cbz Ar-H), 5.10 (1H, 2 × s, OCH_2Ph), 4.48 (1H, dd, *J* 7.1, 4.5, 7-H), 4.17-4.08 (1H, m, 9-H), 3.97-3.82 (2H, m, includes: 1H, 2-H_A; and at δ 3.90: 0.5H, dd, *J* 12.2, 4-H_A and at δ 3.85: 0.5H, dd, *J* 12.2, 4-H_A), 3.79 (0.5H, d, *J* 4.6, 10-H), 3.75 (0.5H, d, *J* 4.6, 10-H), 3.56 (1H, dd, *J* 12.2, 5.9, 4-H_B), 3.23-3.11 (2H, m, 2-H_B and 5-H), 2.52-2.44 (1H, m, 6-H_A), 2.10-2.00 (1H, m, 8-H_B), 1.96-1.75 (2H, m, includes: 1H, 6-H_B; and at

δ 1.87: 1H, app. d, *J* 14.3, 8-H_A). **^{13}C NMR** (125 MHz, $CDCl_3$, mixture of two rotamers): δ 154.9 (N(CO)O), 154.7 (N(CO)O), 137.0 (Ar-C_q), 136.8 (Ar-C_q), 128.6 (Ar-C), 128.1 (Ar-C), 128.1 (Ar-C), 128.1 (Ar-C), 128.0 (2 peaks, Ar-C), 92.5 (1-C), 91.5 (1-C), 76.7 (7-C), 76.5 (7-C), 68.2 (10-C), 67.8 (10-C), 67.4 (9-C), 67.3 (9-C), 67.1 (O*C*H_2Ph), 67.0 (O*C*H_2Ph), 55.2 (2-C), 54.8 (2-C), 52.1 (4-C), 51.5 (4-C), 40.8 (5-C), 39.8 (5-C), 38.1 (8-C), 37.6 (8-C), 37.0 (6-C), 36.7 (6-C) [30 of 30 expected peaks observed]. **IR** ν_{max}(film)/cm^{-1} 3414 (br., OH), 2948, 2885, 1675, 1424, 1349, 1114, 1076. **HRMS** (ESI): $C_{17}H_{22}NO_5$ $[M+H]^+$; calculated 320.1492, found 320.1494.

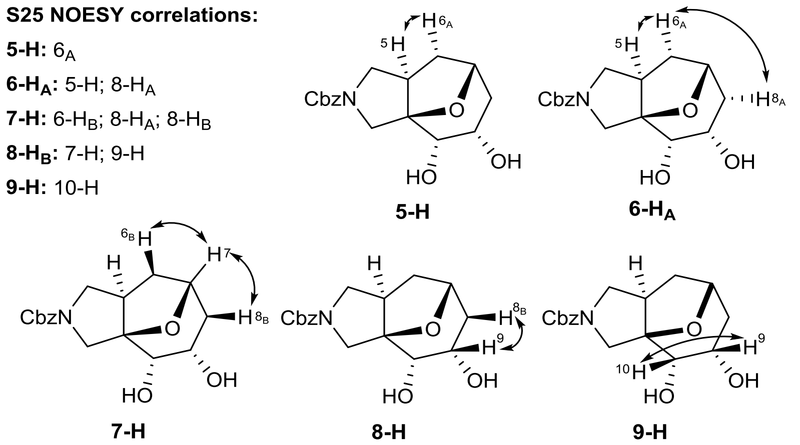

(*1S,*5R**,*7R**)-10-[(2,4-dimethoxyphenyl)methyl]-12-oxa-3,10-diazatricyclo[5.4.1.0^{1,5}]dodecane-3-carboxylate 15**

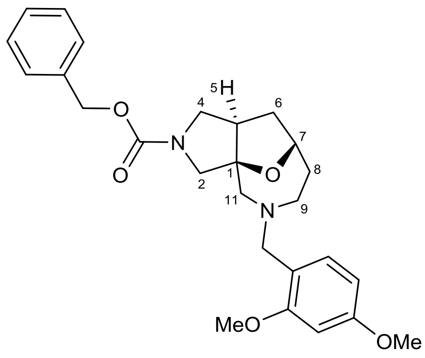

15

$NaIO_4$ (2.14 g, 10.0 mmol, 2.00 eq.) was added to a stirred solution of diol **S25** (1.6 g, 5.0 mmol, 1.0 eq.) in 2:1 MeOH–H_2O (45 mL) at 0 °C. The reaction mixture was warmed to rt and stirred for 15 h. The reaction mixture was concentrated *in vacuo*, then diluted in CH_2Cl_2 (50 mL) and washed with brine (50 mL). The phases were separated and the aqueous layer was extracted with CH_2Cl_2 (3 × 25 mL). The resulting crude dialdehyde was dissolved in CH_2Cl_2 (50 mL), and (2,4-dimethoxyphenyl)methanamine (1.0 mL, 6.3 mmol, 1.3 eq.) and $NaBH(OAc)_3$ (2.76 g, 13.0 mmol, 2.60 eq.) were added. The reaction mixture was stirred for 15 h then filtered through Celite and concentrated *in vacuo*. The resulting reside was diluted in EtOAc (50 mL) and washed with brine (50 mL). The aqueous phase was extracted with EtOAc (2 × 25 mL). The combined organic phase was dried over $MgSO_4$, filtered and concentrated *in vacuo*. Flash chromatography eluting with 98:2:0.1 CH_2Cl_2–EtOH–NH_3/MeOH gave the *title compound* **15** (685 mg, 1.51 mmol, 30%, 2 steps) as a colourless oil. **R_f** 0.51 (29:1:0.1 CH_2Cl_2–EtOH–NH_3/MeOH). **^{1}H NMR** (500 MHz, $CDCl_3$): δ 7.38-7.27 (6H, m, Cbz Ar-H and DMB 6-H), 6.47 (1H, dd, *J* 8.3, 2.2, DMB 5-H), 6.44 (1H, d, *J* 2.2, DMB 3-H), 5.09 (2H, s, OC*H*$_2$Ph),

4.62 (1H, td, *J* 8.3, 3.5, 7-H), 3.81 (3H, s, OCH_3), 3.79-3.74 (4H, m, includes: 1H, 4-H_A; and at δ 3.78: 3H, s, OCH_3), 3.65 (1H, d, *J* 12.2, 2-H_A), 3.56 (1H, d, *J* 13.9, NCH_AH_BAr), 3.53 (1H, d, *J* 13.9, NCH_AH_BAr), 3.33-3.11 (2H, m, 2-H_B and 4-H_B), 2.77 (1H, dd, *J* 13.3, 6.8, 9-H_A), 2.69 (1H, d, *J* 12.2, 11-H_A), 2.66-2.46 (3H, m, 5-H, 9-H_B and 11-H_B), 2.34-2.23 (1H, m, 6-H_A), 2.14-2.01 (1H, m, 6-H_B), 1.98-1.89 (1H, m, 8-H_A), 1.63-1.52 (1H, m, 8-H_B). **^{13}C NMR** (125 MHz, $CDCl_3$, 329 K, mixture of two rotamers, DMB 1-C not observed): δ 159.9 (DMB 2-C or 4-C), 158.8 (DMB 2-C or 4-C), 154.7 (N(CO)O), 137.1 (Ar-C_q), 130.4 (Ar-C), 128.6 (Ar-C), 128.0 (Ar-C), 120.4 (DMB 6-C), 104.2 (DMB 5-C), 98.5 (DMB 3-C), 94.0 (1-C), 77.7 (7-C), 66.9 (O*C*H_2Ph), 66.1 (O*C*H_2Ph), 64.4 (11-C), 64.2 (11-C), 57.1 (N*C*H_2Ar), 55.5 (2 peaks, 2 × OCH_3), 55.3 (2-C), 54.9 (2-C), 53.6 (4-C), 53.2 (4-C), 53.0 (9-C), 46.3 (5-C), 45.3 (5-C), 40.9 (6-C), 40.5 (6-C), 36.8 (8-C) [29 of 48 expected peaks observed]. **IR** ν_{max}(film)/cm^{-1} 2935, 1704 (CO), 1612, 1587, 1505, 1455, 1208. **HRMS** (ESI): $C_{26}H_{33}N_2O_5$ [M+H]$^+$; calculated 453.2384, found 453.2392.

5.4.2.3.4 Preparation of oxazolidinone scaffold 16

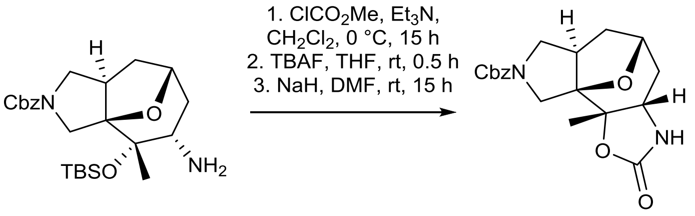

13 22% **16** 34%, 3 steps

Benzyl (*1R*,2S*,6S*,8R*,10R)-2-methyl-4-oxo-3,14-dioxa-5,12-diazatetracyclo[6.5.1.0^{1,10}.0^{2,6}]tetradecane-12-carboxylate 16**

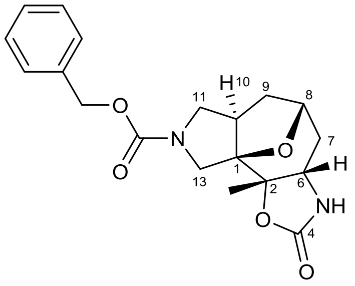

16

Methyl chloroformate (130 μL, 1.70 mmol, 10.0 eq.) was added to a stirred solution of compound **13** (74 mg, 0.17 mmol, 1.00 eq.) and Et_3N (240 μL, 1.70 mmol, 10.0 eq.) in CH_2Cl_2 at 0 °C. The reaction mixture was warmed to rt and stirred for 15 h. The reaction mixture was diluted with CH_2Cl_2 (20 mL) and washed with sat. aq. NH_4Cl solution (25 mL). The aqueous layer was extracted with CH_2Cl_2 (2 × 10 mL). The combined organic phases were washed with brine (25 mL), then dried over $MgSO_4$, filtered, and concentrated *in vacuo* to give a colourless oil (75 mg), which was carried on to the next step without further purification. The residue was diluted in THF (5 mL) and TBAF (1.0 M in THF, 0.38 mL, 0.38 mmol, 2.25 eq.) was added. The reaction mixture was stirred for 0.5 h, then concentrated *in vacuo*. Purification by SCX SPE following general procedure **A**, eluting with MeOH gave a colourless oil (39 mg), which was carried on to the next step without further purification. The residue was diluted

in DMF (3.0 mL) and NaH (60% dispersion in oil, 20 mg, 0.5 mmol, 3.0 eq.) was added. The reaction mixture was stirred at rt for 15 h, then sat. aq. NH_4Cl solution (0.2 mL) was added. The reaction mixture was diluted in EtOAc (25 mL) and washed with brine (10 mL). The aqueous phase was extracted with EtOAc (2 × 10 mL), then then dried over $MgSO_4$, filtered, and concentrated *in vacuo*. Flash chromatography eluting with 98:2:0.1 CH_2Cl_2–EtOH–NH_3/MeOH gave the *title compound* **16** (20 mg, 56 μmol, 34%, [three steps]) as a colourless oil. **R_f** 0.07 (98:2:0.1 CH_2Cl_2–EtOH–NH_3/MeOH). **^{1}H NMR** (500 MHz, $CHCl_3$, 50:50 mixture of rotamers): δ 7.41-7.28 (5H, m, Cbz Ar-H), 5.12 (2H, s, OC*H*2Ph), 5.10 (1H, s, NH), 4.64-4.58 (1H, m, 8-H), 4.00-3.89 (1H, m, 11-H_A), 3.88-3.81 (1H, m, 6-H), 3.80-3.62 (2H, m, 13-H), 3.31-3.17 (1H, m, 11-H_B), 3.07-2.92 (1H, m, 10-H), 2.33-2.23 (1H, m, 9-H_A), 2.17-2.09 (1H, m, 7-H_A), 2.03-1.91 (1H, m, 9-H_B), 1.76 (1H, dd, *J* 15.8, 3.2, 7-H_B), 1.56 (1.5H, s, CH_3), 1.55 (1.5H, s, CH_3). **^{13}C NMR** (100 MHz, $CHCl_3$, mixture of two rotamers): δ 159.1 (oxazolidinone N(CO)O), 154.5 (Cbz N(CO)O), 136.9 (Ar-C_q), 128.6 (Ar-C), 128.1 (Ar-C), 128.1 (Ar-C), 93.2 (1-C), 92.3 (1-C), 80.1 (2-C), 80.0 (2-C), 76.2 (8-C), 76.1 (8-C), 67.1 (O*C*H_2Ph), 56.4 (6-C), 55.0 (11-C), 54.6 (11-C), 51.5 (13-C), 51.0 (13-C), 41.4 (10-C), 40.5 (10-C), 37.4 (9-C), 37.3 (9-C), 32.6 (7-C), 21.7 (C_q*C*H_3) [24 of 34 expected peaks observed]. **IR** ν_{max}(film)/cm^{-1} 3275 (NH), 2954, 2884, 1757 (CO), 1697, 1420, 1114, 958. **HRMS** (ESI): $C_{19}H_{23}N_2O_5$ [M+H]$^+$; calculated 359.1601, found 359.1602.

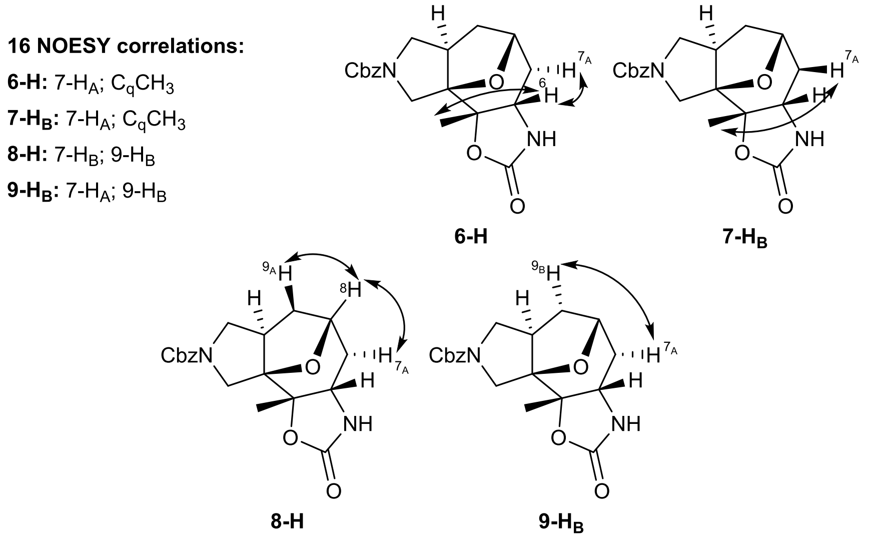

5.4.2.4 Scaffolds derived from cycloadduct 2d

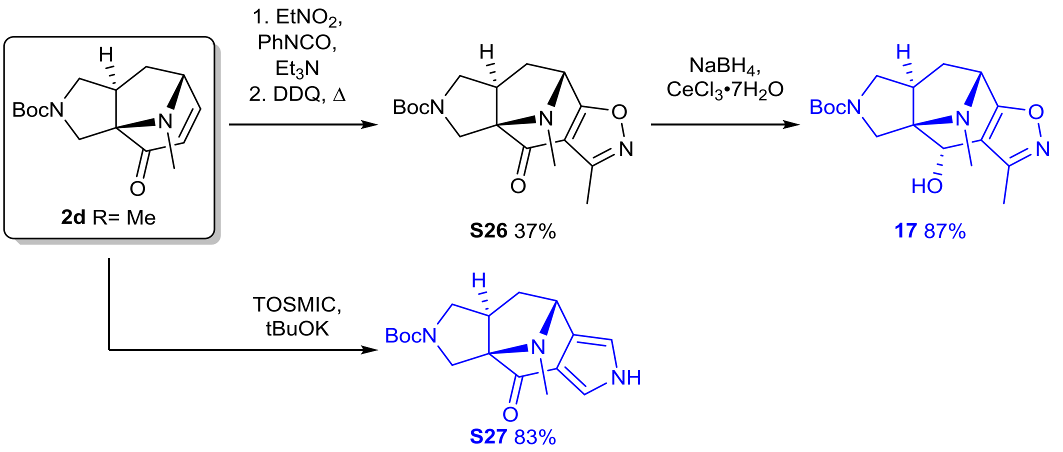

5.4.2.4.1 Preparation of scaffold 17

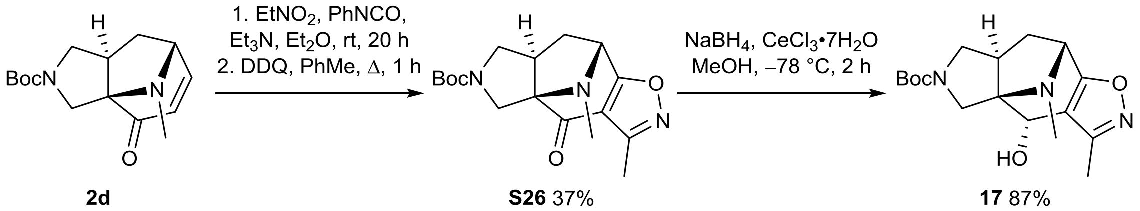

***tert*-Butyl (1*R**,8*R**,10*R**)-4,14-dimethyl-2-oxo-6-oxa-5,12,14-triazatetracyclo[6.5.1.0^{1,10}.0^{3,7}]tetradeca-3(7),4-diene-12-carboxylate S26**

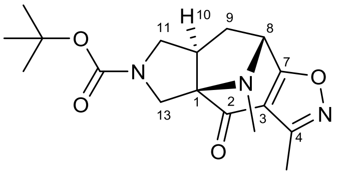

S26

To a stirred solution of enone **2d** (1.21 g, 4.35 mmol, 1.00 eq.) in Et_2O (9.0 mL) at rt was added nitroethane (0.62 mL, 8.70 mmol, 2.00 eq.), trimethylamine (30 μL, 0.22 mmol, 0.05 eq.) and phenylisocyanate (1.89 mL, 17.4 mmol, 4.00 eq.). The resulting solution was stirred for 20 h and then filtered and concentrated *in vacuo* to give a crude orange oil. The crude material was dissolved in PhMe (20 mL) and DDQ (1.97 g, 8.70 mmol, 2.00 eq.) was added. The resulting suspension was heated at reflux for 1 h, cooled, filtered through celite and concentrated *in vacuo* to give a crude oil. Flash chromatography eluting with 9:1 CH_2Cl_2–EtOAc gave the *title compound* **S26** (531 mg, 1.59 mmol, 36%) as a pale yellow oil. **^{1}H NMR** (MeOD-d_4, 500 MHz, 333 K): δ 4.56 (1H, d, *J* 6.3, 8-H), 4.05 (1H, d, *J* 12.5, 13-H), 3.79 (1H, dd, *J* 10.8, 9.4, 11-H_B), 3.29 (1H, d, *J* 12.5, 13-H_A), 3.23 (1H, dd, *J* 10.8, 7.6, 11-H_A), 2.58 (1H, app. qd, *J* 8.7, 4.8, 10-H), 2.33 (3H, s, NCH_3), 2.20 (3H, s, C_qCH_3), 2.17 (1H, ddd, *J* 12.7, 6.3, 4.6, 9-H_B), 2.02 (1H, dd, *J* 12.7, 8.6, 9-H_A), 1.38 (9H, s, $C_q(CH_3)_3$). **^{13}C NMR** (MeOD-d_4,

125 MHz, 333 K, one C not observed): 192.2, 182.5, 158.2, 156.0, 112.0, 82.9, 81.2, 64.0, 54.5, 46.8, 46.3, 36.1, 32.5, 28.6, 10.4. **IR** ν_{max}(film)/cm^{-1} 1692, 1646, 1407, 1167. **HRMS** (ESI): $C_{17}H_{24}N_3O_4$ [M+H]$^+$; calculated 334.1761, found 334.1764. The regioselectivity was confirmed by determination of the structure of the fragment **F21** in complex with ATAD2, and is consistent with precedent.[S10]

tert-Butyl (1_R_*,2_S_*,8_R_*,10_R_*)-2-hydroxy-4,14-dimethyl-6-oxa-5,12,14-triazatetracyclo[6.5.1.0^{1,10}.0^{3,7}]tetradeca-3(7),4-diene-12-carboxylate 17

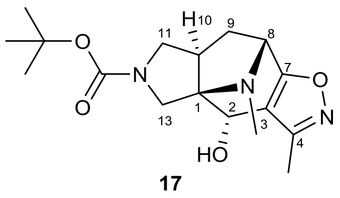

17

To a stirred solution of isoxazole **S26** (498 mg, 1.48 mmol, 1.00 eq.) in MeOH (7.0 mL) at –78 °C was added $CeCl_3 \cdot 7H_2O$ (664 mg, 1.78 mmol, 1.20 eq.) followed by $NaBH_4$ (67 mg, 1.78 mmol, 1.20 eq.). After 2 h, the solvent was removed *in vacuo* and the residue dissolved in CH_2Cl_2 (10 mL) and H_2O (10 mL). The layers were separated and the aqueous layer extracted with CH_2Cl_2 (2 x 10 mL). The combined organic layers were dried over $MgSO_4$, filtered and concentrated *in vacuo* to give a crude colourless oil. Flash chromatography eluting with 3:2 CH_2Cl_2–EtOAc gave the *title compound* **17** (431 mg, 1.29 mmol, 87%) as a colourless oil. **^{1}H NMR** (MeOD-d$_4$, 500 MHz, 333 K): δ 4.91 (1H, d, *J* 8.0, 2-H), 4.20 (1H, d, *J* 5.7, 8-H), 3.82-3.67 (1H, m, 11-H$_A$), 3.60 (1H, d, *J* 12.0, 13-H$_A$), 3.51 (1H, app. t, *J* 10.1, 11-H$_B$), 3.39-3.25 (1H, m, 13-H$_B$), 3.14-3.00 (1H, m, 10-H), 2.38 (3H, s, CH_3), 2.33 (3H, s, CH_3), 2.19-2.07 (1H, m, 9-H$_B$), 2.06-1.96 (1H, m, 9-H$_A$), 1.49 (9H, s, $C_q(CH_3)_3$). **^{13}C NMR** (MeOD-d$_4$, 125 MHz, 333 K, 2 × C_q and 2 × depressed pyrrolidine carbons not observed): 158.5, 154.9, 111.1, 79.7, 62.7, 61.2, 38.1, 30.5, 27.3, 8.9. **IR** ν_{max}(film)/cm^{-1} 3334, 1649, 1419, 1166. **HRMS** (ESI): $C_{17}H_{26}N_3O_4$ [M+H]$^+$; calculated 336.1923, found 336.1918.

5.4.2.4.2 Preparation of scaffold S27

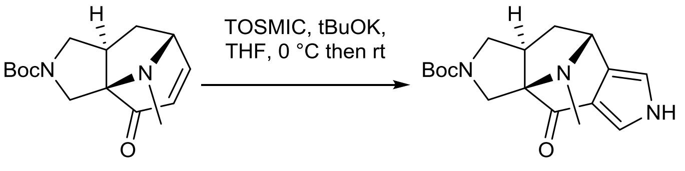

2d **S27** 83%

***tert*-Butyl (1*R**,8*R**,10*R**)-14-methyl-2-oxo-5,12,14-triazatetracyclo[6.5.1.0^{1,10}.0^{3,7}]tetradeca-3,6-diene-12-carboxylate S27**

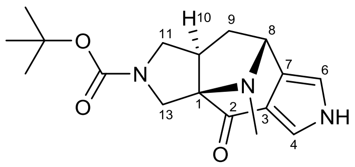

S27

To a solution of cycloadduct **2d** (1.80 g, 6.47 mmol, 1.00 eq.) in THF (32 mL) at 0 °C was added TOSMIC (1.26 g, 6.47 mmol, 1.00 eq) followed by ^{t}BuOK (2.18 g, 19.4 mmol, 3.00 eq.). The resulting suspension was warmed to rt and stirred for 2 h. Sat. aq. NH_4Cl solution (50 mL) and EtOAc (50 mL) were added and the layers were separated. The aqueous layer was extracted with EtOAc (2 x 50 mL). The combined organic layers were dried over $MgSO_4$, filtered and concentrated *in vacuo* to give a yellow oil. Flash chromatography 97:3:0.3 CH_2Cl_2–EtOAc–NH_4OH gave the *title compound* **S27** (1.71 g, 5.39 mmol, 83%) as a yellow oil. **^{1}H NMR** (500 MHz, MeOD-d$_4$, 333 K): δ 7.27 (1H, d, *J* 1.7, 4-H), 6.53 (1H, d, *J* 1.7, 6-H), 4.26 (1H, d, *J* 6.0, 8-H), 4.13 (1H, d, *J* 12.3, 13-H$_A$), 3.77 (1H, dd, 10.9, 9.5, 11-H$_B$), 3.29-3.18 (2H, m, 11-H$_A$ and 13-H$_B$), 2.59 (1H, app. qd, *J* 8.8, 5.1, 10-H), 2.14 (3H, s, NCH_3), 2.02-2.09 (1H, m, 9-H$_B$), 1.87 (1H, dd, *J* 12.0, 8.8, 9-H$_A$), 1.38 (9H, s, $C_q(CH_3)_3$). **^{13}C NMR** (125 MHz, MeOD-d$_4$, 333 K, one C not observed): 194.6, 156.3, 128.9, 121.7, 118.6, 114.9, 81.0, 63.1, 54.7, 47.5, 46.8, 39.4, 33.0, 28.7. **IR** ν_{max}(film)/cm^{-1} 3271, 2972, 1655, 1518, 1476, 1448, 1417, 1392, 1349, 1166, 1121. **HRMS** (ESI): $C_{17}H_{24}N_3O_3$ [M+H]$^+$; calculated 318.1812, found 318.1820.

5.4.2.5 Scaffold derived from cycloadduct 2e

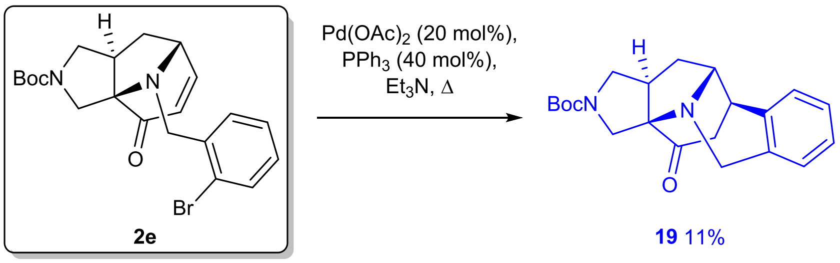

2e **19** 11%

5.4.2.5.1 Preparation of scaffold 19

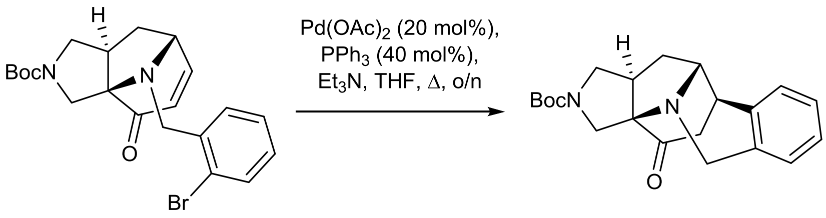

2e **19** 11%

***tert*-Butyl (1*R**,10*R**,13*R**,17*R**)-12-oxo-2,15-diazapentacyclo[8.8.0.0^{2,13}.0^{4,9}.0^{13,17}]octadeca-4(9),5,7-triene-15-carboxylate 19**

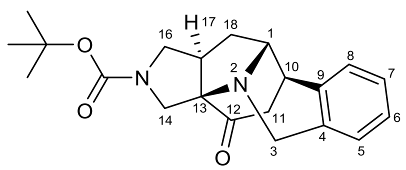

19

To a solution of cycloadduct **2e** (140 mg, 0.32 mmol, 1.00 eq) in THF (1.0 mL) was added $Pd(OAc)_2$ (15 mg, 65 μmol, 20 mol%), PPh_3 (34 mg, 0.129 mmol, 40 mol%) and Et_3N (90 μL, 0.65 mmol, 2.00 eq.). The resulting suspension was heated to reflux, stirred over night, then concentrated *in vacuo.* Flash chromatography eluting with 9:1 CH_2Cl_2:EtOAc, followed by mass-directed preparative HPLC (50-90% MeOH–H_2O) gave the *title compound* **19** (12 mg, 34 μmol, 11%) as a brown oil. **^{1}H NMR** (500 MHz, MeOD-d_4, 333 K): δ 7.10-7.01 (2H, m, Ar-H), 6.95-6.90 (2H, m, Ar-H), 4.24 (1H, d, *J* 18.6, 3-H_A), 3.93 (1H, d, *J* 18.6, 3-H_B), 3.83 (1H, dd, *J* 10.3, 9.6, 16-H_B), 3.74 (1H, d, *J* 12.6, 14-H_A), 3.67 (1H, d, *J* 6.6, 1-H), 3.31 (1H, d, *J* 12.6, 14-H_B), 3.24 (1H, d, *J* 6.3, 10-H), 3.17 (1H, d, *J* 9.6, 16-H_A), 3.02 (1H, dd, *J* 15.0, 6.3, 11-H_B), 2.82 (1H, app. qd, *J* 9.1, 3.1, 17-H), 2.30 (1H, dd, *J* 13.4, 8.8, 18-H_A), 2.10 (1H, ddd, *J* 13.4, 6.6, 3.1, 18-H_B), 2.04 (1H, d, *J* 15.0, 11-H_A), 1.37 (9H, s, $C_q(CH_3)_3$). **^{13}C NMR** (125 MHz, MeOD-d_4, 333 K): δ 197.8, 156.1, 140.3, 133.0, 128.9, 128.2, 127.8, 126.7, 93.4, 81.1, 60.8, 49.4, 49.2, 47.3, 46.3, 44.8, 33.5, 28.7 (2 peaks). **LRMS** (HPLC-MS): $C_{21}H_{27}N_2O_3$; found 355.2 $[M+H]^+$.

5.4.2.6 Scaffolds derived from cycloadduct 2f

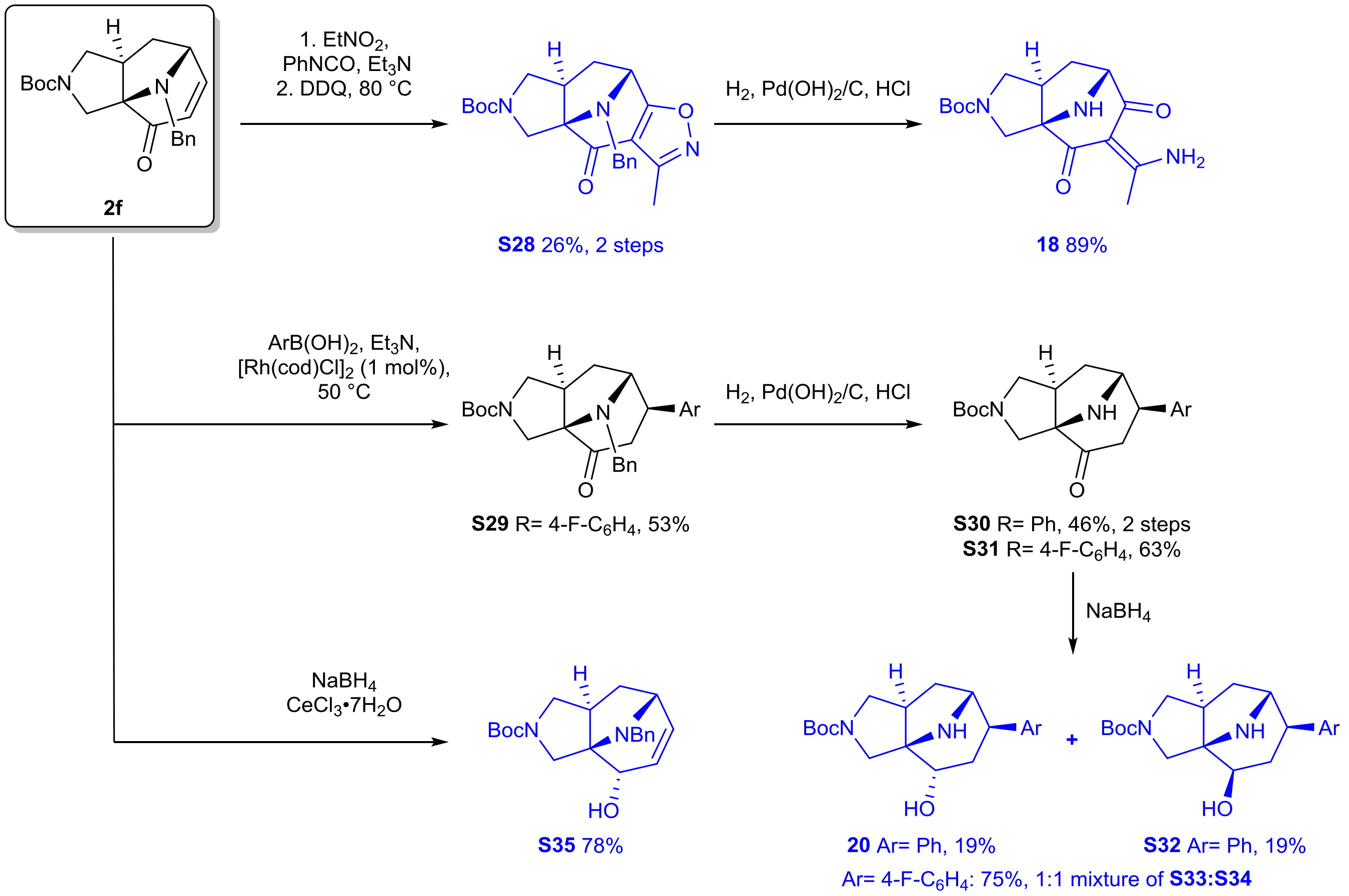

5.4.2.6.1 Preparation of scaffold 18

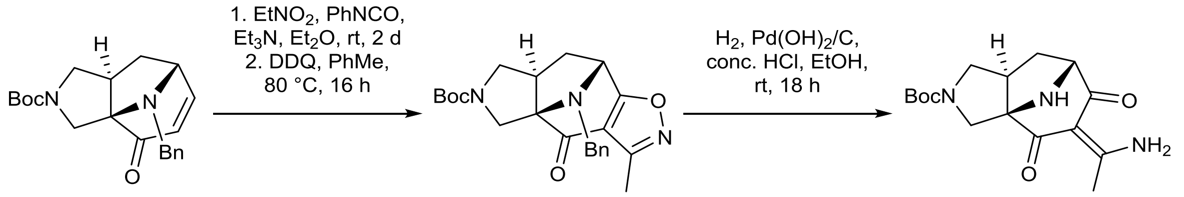

***tert*-Butyl (1*R**,8*R**,10*R**)-14-benzyl-4-methyl-2-oxo-6-oxa-5,12,14-triazatetracyclo[6.5.1.0^{1,10}.0^{3,7}]tetradeca-3(7),4-diene-12-carboxylate S28**

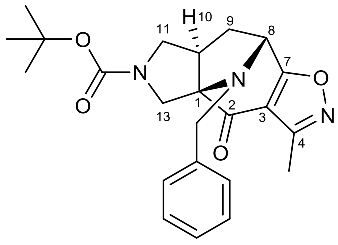

S28

To a stirred solution of cycloadduct **2f** (1.80 g, 5.08 mmol, 1.00 eq.) in Et_2O (10 mL) at rt was added $EtNO_2$ (0.73 mL, 10.2 mmol, 2.00 eq.), PhNCO (2.21 mL, 20.3 mmol, 4.00 eq.) and Et_3N (0.05 mL, 0.254 mmol, 5 mol%). The resulting solution was stirred at rt for 2 days (during which time a colourless solid precipitated). The suspension was filtered (washing with Et_2O) and the filtrate was evaporated to give a brown oil. The residue was dissolved in PhMe (25 mL) and DDQ (2.31 g, 10.2 mmol, 2.00 eq.) was added. The resulting suspension was heated to 80 °C for 16 h, then filtered through celite and concentrated *in vacuo* to give a crude brown oil. Flash chromatography eluting with 4:1 CH_2Cl_2–EtOAc gave the *title compound* **S28** (549 mg, 1.34 mmol, 26%) as a yellow oil. **^{1}H NMR** (500 MHz, MeOD-d_4, 333 K): δ 7.32-7.22 (3H, m, Bn Ar-H), 7.19-7.12 (2H, m, Bn Ar-H), 4.50-4.44 (1H, m, 8-H), 4.19 (1H, d, *J* 12.6, 13-H_A), 3.91 (1H, dt, *J* 11.0, 9.2, 11-H_B), 3.84 (1H, d, *J* 13.9, C*H*$_A$H$_B$Ph), 3.53 (1H, d, *J* 12.6, 13-H_B), 3.43 (1H, dd, *J* 11.0, 7.4, 11-H_A), 3.41 (1H, d, *J* 13.9, CH$_A$*H*$_B$Ph), 2.72 (1H, dddd, *J* 9.2, 8.6, 7.4, 5.3, 10-H), 2.46 (3H, s, CH_3), 2.21 (1H, dd, *J* 12.7, 5.3, 9-H_A), 2.11 (1H, dd, *J* 12.7, 5.3, 9-H_B), 1.48 (9H, s, $C_q(CH_3)_3$). **^{13}C NMR** (125 MHz, MeOD-d_4, 333 K): δ 192.7, 182.9, 158.3, 156.1, 139.1, 129.6, 129.3, 128.6, 112.7, 82.5, 81.3, 60.7, 54.1, 50.8, 47.5, 47.0, 36.0, 28.7, 10.3. **IR** ν_{max}(film)/cm^{-1} 1691, 1455, 1406, 1365, 1167, 1139, 1113, 869. **HRMS** (ESI): $C_{23}H_{28}N_3O_4$ $[M+H]^+$; calculated 410.2074, found 410.2080.

***tert*-Butyl (1*R**,5*R**,7*R**,9*E*)-9-(1-aminoethylidene)-8,10-dioxo-3,11-diazatricyclo[5.3.1.0^{1,5}]undecane-3-carboxylate 18**

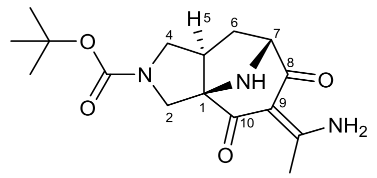

18

Hydrogenation was carried out following general procedure **B**, using isoxazole **S28** (537 mg, 1.31 mmol, 1.00 eq), $Pd(OH)_2$/C (54 mg, 10% w/w) and conc. HCl (0.1 mL) in EtOH (7.0 mL) over 18 h. Filtration followed by concentration gave the *title compound* **18** (373 mg, 1.16 mmol, 89%) as a clear, colourless oil. **^{1}H NMR** (500 MHz, MeOD-d$_4$, 333 K, 3 × NH not observed): δ 4.20 (1H, d, *J* 12.1, 2-H$_A$), 3.78 (1H, dd, *J* 10.8, 9.4, 4-H$_A$), 3.77-3.73 (1H, m, 7-H), 3.24 (1H, d, *J* 12.1, 2-H$_B$), 3.10 (1H, dd, *J* 10.8, 8.3, 4-H$_B$), 2.64-2.53 (1H, m, 5-H), 2.39 (3H, s, C$_q$CH$_3$), 1.98-1.91 (2H, m, 6-H), 1.37 (9H, s, C$_q$(CH$_3$)$_3$). **^{13}C NMR** (125 MHz, MeOD-d$_4$, 333 K): δ 201.4, 196.9, 177.6, 156.1, 103.9, 80.9, 79.2, 69.0, 54.7, 50.9, 46.8, 35.6, 28.9, 24.3. **LRMS** (HPLC-MS): $C_{16}H_{23}NNa_3O_4$ [M+Na]$^+$; found 344.2.

5.4.2.6.2 Preparation of scaffolds 20 and S32-S34

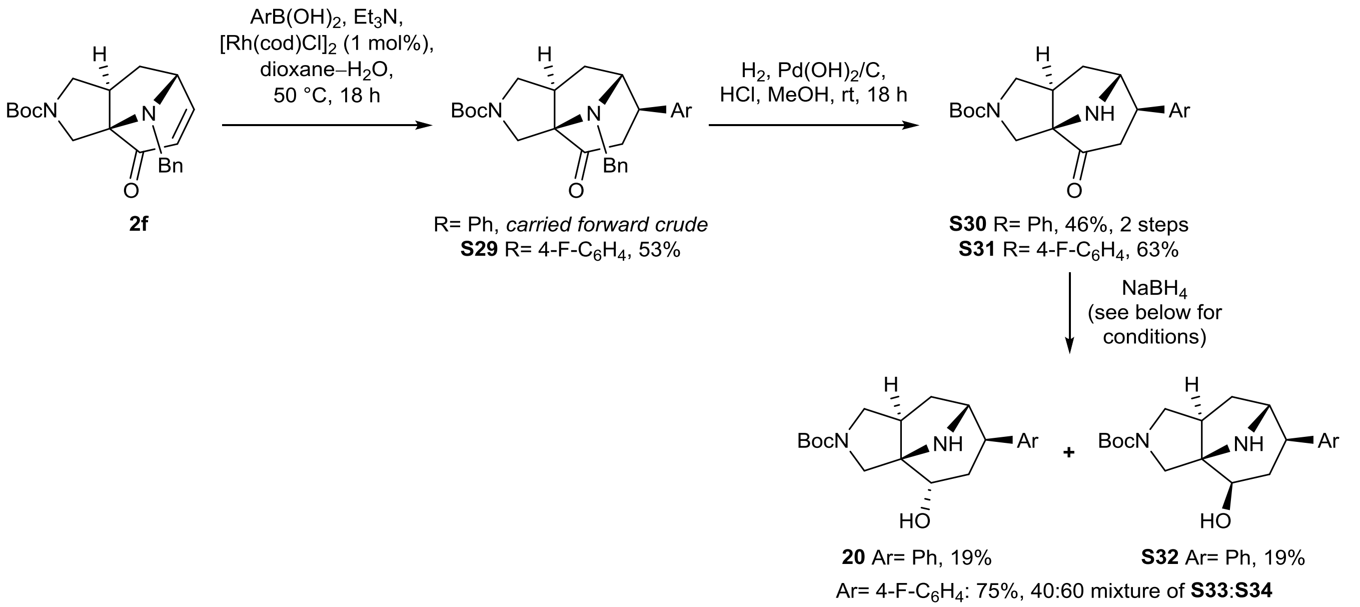

General procedure L: Arylation of α,β-unsaturated ketone 2f

To a solution of cycloadduct **2f** (1.0 eq.) in 86:14 dioxane–H_2O (0.3 M) was added $ArB(OH)_2$ (1.5 eq.), Et_3N (1.0 eq.) and $[Rh(cod)Cl]_2$ (1 mol%). The resulting solution was warmed to 50 °C for 2 h before being concentrated *in vacuo*. The products were purified by flash chromatography.

tert-Butyl (1R^*,5R^*,7R^*,8R^*)-11-benzyl-8-(4-fluorophenyl)-10-oxo-3,11-diazatricyclo[5.3.1.0^{1,5}]undecane-3-carboxylate S29

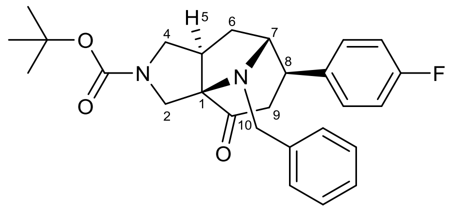

S29

General procedure **L** was followed using cycloadduct **2f** (512 mg, 1.44 mmol) and 4-fluorophenylboronic acid. Flash chromatography eluting with 95:5 CH_2Cl_2–EtOAc gave the *title compound* **S29** (342 mg, 0.76 mmol, 53%) as a colourless oil. **^{1}H NMR** (500 MHz, DMSO-d$_6$, 343 K): δ 7.13-6.93 (7H, m, includes 5H, Bn Ar-H; and 2H, Ar 2-H), 6.84 (2H, d, *J* 7.0, Ar 3-H), 3.84 (1H, app. t, *J* 10.4, 4-H$_A$), 3.81 (1H, d, *J* 13.3, 2-H$_A$), 3.65 (1H, d, *J* 14.2, NC*H*$_A$H$_B$Ph), 3.60-3.45 (2H, m, 2-H$_B$ and 7-H), 3.36 (1H, d, *J* 14.2, NCH$_A$*H*$_B$Ph), 3.27 (1H, app. d, *J* 8.6, 8-H), 3.18 (1H, dd, *J* 11.0, 7.6, 4-H$_B$), 3.11-3.05 (1H, m, 5-H), 3.01 (1H, dd, *J* 16.5, 8.7, 9-H$_A$), 2.55-2.45 (1H, m, 9-H$_B$), 2.27 (1H, dd, *J* 13.1, 8.6, 6-H$_A$), 2.19 (1H, ddd, *J* 13.1, 6.5, 4.7, 6-H$_B$), 1.35 (9H, s, $C_q(CH_3)_3$). **^{13}C NMR** (125 MHz, DMSO-d$_6$, 343 K, one C not observed): δ 204.4, 160.8 (d, *J* 238), 161.7, 159.8, 153.1, 140.1 (d, *J* 164), 129.2 (d, *J* 8.0), 128.2, 127.6, 126.4, 114.4 (d, *J* 21), 78.6, 68.8, 54.4, 52.1, 46.5, 45.2, 38.9, 35.6, 28.1. **IR** ν_{max}(film)/cm^{-1} 1680, 1023, 990, 826. **HRMS** (ESI): $C_{27}H_{32}FN_2O_3$ [M+H]$^+$; calculated 451.2396, found 451.2396.

tert-Butyl (1R^*,5R^*,7R^*,8R^*)-10-oxo-8-phenyl-3,11-diazatricyclo[5.3.1.0^{1,5}]undecane-3-carboxylate S30

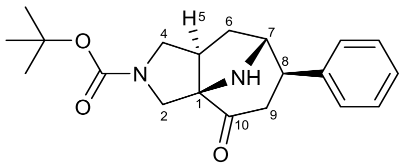

S30

General procedure **L** was followed using cycloadduct **2f** (484 mg, 1.37 mmol) and $PhB(OH)_2$. Flash chromatography eluting with 9:1 CH_2Cl_2–EtOAc gave the crude ketone, which was contaminated with $PhB(OH)_2$ {**^{1}H NMR peaks** (MeOD-d$_4$, 500 MHz, 333 K, characteristic peaks): δ 7.24-7.09 (5H, m, Bn Ar-H), 7.10-6.98 (3H, m, Ar-H), 6.89-6.73 (2H, m, Ar-H), 4.00-3.84 (2H, m, 2-H$_A$ and 4-H$_A$), 3.73 (1H, d, *J* 14.1, C*H*$_A$H$_B$Ph), 3.73 (1H, s, 7-H), 3.67-3.53 (1H, m, 8-H), 3.40 (1H, d, *J* 14.2, CH$_A$*H*$_B$Ph), 3.33-3.23 (2H, m, 2-H$_B$ and 4-H$_B$), 3.15-3.04 (1H, m, 5-H), 3.00 (1H, dd, *J* 16.5, 8.6, 9-H$_B$), 2.76 (1H, ddd, *J* 16.5, 2.3, 1.4, 9-H$_A$), 2.34 (1H, dd, *J* 13.1, 8.9, 6-H$_A$), 2.23 (1H, ddd, *J* 13.1, 6.4, 5.0, 6-H$_B$), 1.44 (9H, s, $C_q(CH_3)_3$). **LRMS** (HPLC-MS): $C_{20}H_{27}N_2O_3$ [M+H]$^+$; found 433.5}. Hydrogenation of the crude residue was carried out following general procedure **B**, using $Pd(OH)_2$/C (44 mg, 10 w/w%) and conc. HCl (0.1 mL) in MeOH (5 mL) over 18 h. Flash chromatography eluting with 1:1 CH_2Cl_2–EtOAc gave the amine **S30** (218 mg, 0.64 mmol, 46% over two steps) as a colourless oil. **^{1}H NMR** (500 MHz, MeOD-d$_4$, 333 K, NH not observed): δ 7.23 (2H, dd, *J* 8.2, 7.2, Ar-H), 7.15-7.10 (3H, m, Ar-H), 3.93 (1H, d, *J* 12.4, 2-H$_A$), 3.81 (1H, dd, *J* 10.8, 9.5, 4-H$_B$), 3.61 (1H, d, *J* 6.9, 7-H), 3.36 (1H, d, *J* 8.2, 8-H), 3.19 (1H, d, *J* 12.4, 2-

H_B), 3.10 (1H, dd, *J* 10.8, 8.6, 4-H_A), 2.87 (1H, dd, *J* 16.7, 8.2, 9-H_B), 2.80 (1H, app. qd, *J* 8.8, 3.7, 5-H), 2.52 (1H, d, *J* 16.7, 9-H_A), 2.24 (1H, dd, *J* 13.5, 8.5, 6-H_A), 2.03 (1H, ddd, *J* 13.5, 6.9, 3.7, 6-H_B), 1.35 (9H, s, $C_q(CH_3)_3$). **^{13}C NMR** (125 MHz, MeOD-d_4, 333 K): δ 208.3, 156.0, 145.0, 129.7, 128.4, 127.7, 93.4, 81.0, 64.5, 54.8, 51.1, 50.7, 46.5, 39.7, 37.1, 28.7. **IR** ν_{max}(film)/cm^{-1} 3290, 1648, 1406, 1171, 1118.

***tert*-Butyl (1*R**,5*R**,7*R**,8*R**)-8-(4-fluorophenyl)-10-oxo-3,11-diazatricyclo[5.3.1.0^{1,5}]undecane-3-carboxylate S31**

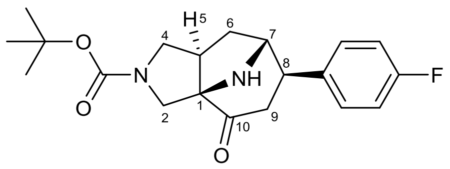

S31

Hydrogenation was carried out following general procedure **B**, using compound **S29** (284 mg, 0.63 mmol), $Pd(OH)_2$/C (28 mg, 10% w/w) and conc. HCl (0.1 mL) in EtOH (3 mL) over 18 h. Flash chromatography eluting with 1:1 CH_2Cl_2–EtOAc gave the *title compound* **S31** (225 mg, 0.62 mmol, 99%) as a colourless oil. **^{1}H NMR** (500 MHz, DMSO-d_6, 343 K, NH not observed): δ 7.45 (2H, app. dd, *J* 8.1, 5.5, Ar 2-H), 7.16 (2H, app. t, *J* 8.7, Ar 3-H), 4.13 (1H, app. d, *J* 5.6, 7-H), 4.00 (1H, d, *J* 13.0, 2-H_A), 3.84 (1H, t, *J* 10.0, 4-H_A), 3.57 (1H, d, *J* 13.0, 2-H_B), 3.49-3.39 (1H, m, 8-H), 3.34-3.27 (1H, m, 4-H_B), 3.11-3.02 (2H, m, 9-H), 3.00 (1H, app. dd, *J* 17.3, 7.7, 5-H), 2.43 (1H, dd, *J* 13.2, 8.7, 6-H_A), 2.24-2.11 (1H, m, 6-H_B), 1.41 (9H, s, $C_q(CH_3)_3$). **^{13}C NMR** (500 MHz, DMSO-d_6, 343 K, one C not observed): δ 201.8, 161.3 (d, *J* 244), 152.9, 138.4, 129.6 (d, *J* 8.0), 115.3 (d, *J* 21.0), 79.1, 63.5, 52.4, 47.2, 44.6, 42.6, 39.6, 36.0, 28.2. **IR** ν_{max}(film)/cm^{-1} 3350, 1678, 1511, 1407, 1366, 1225, 1163, 1133, 1075. **HRMS** (ESI): $C_{16}H_{18}FN_2O_3$ [MH-^{t}Bu]$^+$; calculated 305.1301, found 305.1297.

***tert*-Butyl (1*R**,5*R**,7*R**,8*R**,10*S**)-10-hydroxy-8-phenyl-3,11-diazatricyclo[5.3.1.0^{1,5}]undecane-3-carboxylate 20 and *tert*-Butyl (1*R**,5*R**,7*R**,8*R**,10*R**)-10-hydroxy-8-phenyl-3,11-diazatricyclo[5.3.1.0^{1,5}]undecane-3-carboxylate S32**

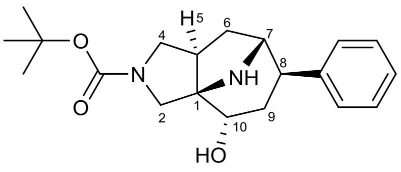

20

+

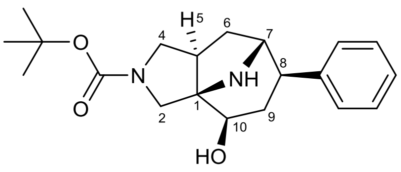

S32

To a stirred solution of compound **S31** (213 mg, 0.62 mmol, 1.00 eq.) in MeOH (3.0 mL) at rt was added $NaBH_4$ (26 mg, 0.69 mmol, 1.10 eq.). The resulting solution was stirred for 2 h. The reaction was quenched by addition of sat. aq. NH_4Cl solution (5 mL). CH_2Cl_2 (5 mL) was added and the layers were separated. The aqueous layer was extracted with CH_2Cl_2 (2 x 5 mL). The combined organic layers were

dried over $MgSO_4$, filtered and concentrated *in vacuo*.* Flash chromatography eluting with 9:1 CH_2Cl_2–EtOAc gave the *title compounds* **20** (40 mg, 0.12 mmol, 19%) and **S32** (40 mg, 0.12 mmol, 19%), both as a colourless solids.

***tert*-Butyl (1*R**,5*R**,7*R**,8*R**,10*S**)-10-hydroxy-8-phenyl-3,11-diazatricyclo[5.3.1.0^{1,5}]undecane-3-carboxylate 20:**

^{1}H NMR (500 MHz, MeOD-d_4, 333 K): δ 7.23-7.10 (5H, m, Ar-H), 3.87-3.78 (1H, m, 10-H), 3.68 (1H, app. t, *J* 10.0, 4-H_A), 3.57 (1H, d, *J* 11.9, 2-H_A), 3.55 (1H, d, *J* 7.4, 7-H), 3.07 (1H, d, *J* 11.9, 2-H_B), 2.96 (1H, dd, *J* 10.9, 8.5, 4-H_B), 2.85 (1H, app. d, *J* 7.3, 8-H), 2.72 (1H, app. qd, 8.9, 8.9, 3.8, 5-H), 2.21-2.10 (1H, m, 9-H_A), 1.97-1.87 (1H, m, 6-H_A), 1.87-1.78 (1H, m, 6-H_B), 1.71 (1H, ddd, *J* 14.4, 10.9, 7.2, 9-H_B), 1.28 (9H, s, $C_q(CH_3)_3$). **^{13}C NMR** (125 MHz, MeOD-d_4, 333 K, one C not observed): δ 156.2, 144.7, 129.5, 128.8, 127.1, 80.7, 67.8, 64.1, 63.9, 55.2, 52.0, 47.7, 40.0, 32.6, 28.8. **IR** ν_{max}(film)/cm^{-1} 3408, 1671, 1411, 1172, 1103. **HRMS** (ESI): $C_{20}H_{29}N_2O_3$ $[M+H]^+$; calculated 345.2178, found 345.2174.

***tert*-Butyl (1*R**,5*R**,7*R**,8*R**,10*R**)-10-hydroxy-8-phenyl-3,11-diazatricyclo[5.3.1.0^{1,5}]undecane-3-carboxylate S32:**

^{1}H NMR (500 MHz, MeOD-d_4, 333 K): δ 7.63 (2H, d, *J* 7.8, Ar-H), 7.29 (2H, t, *J* 7.8, Ar-H), 7.17 (1H, t, *J* 7.8, Ar-H), 3.84 (1H, dd, *J* 11.0. 9.7, 4-H_B), 3.79-3.72 (2H, m, 7-H and 10-H), 3.48 (1H, d, *J* 12.2, 2-H_A), 3.36 (1H, d, *J* 12.2, 2-H_B), 3.17 (1H, dd, *J* 11.0, 8.3, 4-H_A), 2.70 (1H, d, *J* 8.2, 8-H), 2.52 (1H, app. qd, *J* 8.7, 3.9, 5-H), 2.26 (1H, ddd, *J* 15.7, 8.1, 4.7, 9-H_B), 2.15-1.87 (3H, m, 6-H and 9-H_A), 1.44 (9H, s, $C_q(CH_3)_3$). **^{13}C NMR** (125 MHz, MeOD-d_4, 333 K): δ 155.1, 144.9, 128.4, 128.3, 125.9, 79.9, 74.3, 69.9, 63.1, 55.2, 54.8, 43.6, 42.4, 36.2, 29.9, 27.0. **IR** ν_{max}(film)/cm^{-1} 3410, 1672, 1411, 1170, 1108. **HRMS** (ESI): $C_{20}H_{29}N_2O_3$ $[M+H]^+$; calculated 345.2178, found 345.2174. **X-ray crystallography**: CCDC 1526778 contains the supplementary crystallographic data for this compound. Crystals were grown by slow evaporation from dichloromethane.

---

* Analysis of the crude product by ^{1}H NMR spectroscopy at 500 MHz showed a 1:1 mixture of **20** and **S32**.

S32 Crystal structure:

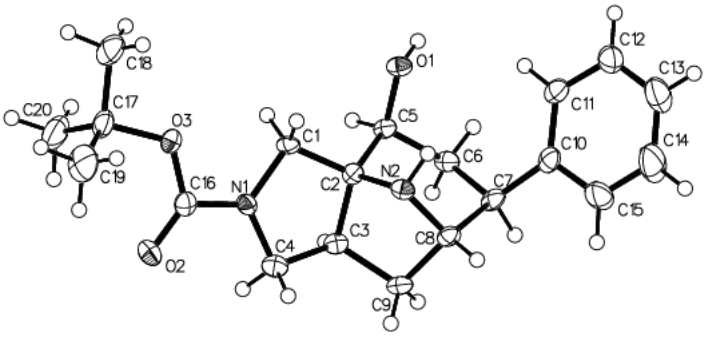

tert-Butyl (1_R_*,5_R_*,7_R_*,8_R_*,10_S_*)-8-(4-fluorophenyl)-10-hydroxy-3,11-diazatricyclo[5.3.1.0^{1,5}]undecane-3-carboxylate S33 _and tert_-Butyl (1_R_*,5_R_*,7_R_*,8_R_*,10_R_*)-8-(4-fluorophenyl)-10-hydroxy-3,11-diazatricyclo[5.3.1.0^{1,5}]undecane-3-carboxylate S34

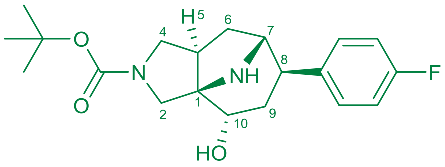

S33

+

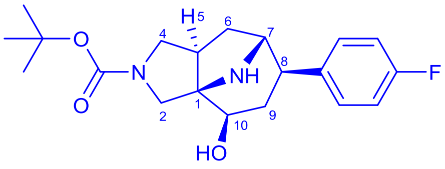

S34

S33:**S34** 40:60

To a stirred solution of compound **S31** (220 mg, 0.61 mmol, 1.00 eq.) in MeOH (3.0 mL) at –78 °C was added $CeCl_3\cdot7H_2O$ (327 mg, 0.88 mmol, 1.40 eq.) and $NaBH_4$ (33 mg, 0.88 mmol, 1.40 eq.). The resulting solution was stirred at rt for 2 h. The reaction was quenched by addition of sat. aq. NH_4Cl solution (5 mL). CH_2Cl_2 (5 mL) was added and the layers were separated. The aqueous layer was extracted with CH_2Cl_2 (2 x 5 mL). The combined organic layers were dried over $MgSO_4$, filtered and concentrated *in vacuo*.* Flash chromatography eluting with 9:1 CH_2Cl_2–EtOAc gave the *title compounds* **S33** and **S34** (165 mg, 0.46 mmol, 75%, 40:60 mixture of diastereomers) as a colourless oil. **^{1}H NMR** (500 MHz, MeOD-d$_4$, 333 K): δ 7.60-7.50 (1.2H, m, major Ar 3-H), 7.29-7.22 (0.8H, m, minor Ar 3-H), 6.98-6.82 (2H, m, major and minor Ar 2-H), 3.87 (0.4H, dd, *J* 10.4, 5.6, minor 10-H), 3.74 (0.6H, dd, *J* 11.0, 9.6, major 4-H$_B$), 3.73 (0.4H, dd, *J* 10.8, 9.9, minor 4-H$_B$), 3.67 (0.6H, dd, *J* 4.7, 1.2, major 10-H), 3.63 (0.4H, d, *J* 11.7, minor 2-H$_A$), 3.62 (0.6H, d, *J* 6.2, major 7-H), 3.58 (0.4H, d, *J* 6.6, minor 7-H), 3.38 (0.6H, d, *J* 12.2, major 2-H$_A$), 3.26 (0.6H, d, *J* 12.2, major 2-H$_B$), 3.14 (0.4H, d, *J* 11.7, minor 2-H$_B$), 3.06 (0.6H, dd, *J* 11.0, 8.2, major 4-H$_A$), 3.00 (0.4H, dd, *J* 11.0, 8.4, minor 4-H$_A$), 2.88 (0.4H, d, *J* 6.9, minor 8-H), 2.77 (0.4H, app. qd, *J* 8.7, 3.7, minor 5-H), 2.71 (0.6H, d, *J* 8.1, major 8-H), 2.52 (0.6H, app. qd, *J* 8.6, 3.8, major 5-H), 2.25 (0.6H, ddd, *J* 15.8, 8.2, 4.7, major 9-H$_A$), 2.16 (0.4H, app. ddt, *J* 14.4, 5.9, 1.3, minor 9-H$_A$), 2.00 (0.6H, dd, *J* 13.2, 8.5, major 6-H$_A$), 1.97-1.84 (2H, m, major 6-H$_B$ and 9-H$_B$; minor 6-H$_A$ and 6-H$_B$), 1.76 (0.4H, 14.4, 10.9, 7.2, minor 9-H$_B$), 1.34 (5.4H, s, major C$_q$(CH$_3$)$_3$), 1.34 (3.6H, s, minor C$_q$(CH$_3$)$_3$). **IR** ν_{max}(film)/cm^{-1} 3350, 1678, 1511, 1407, 1366, 1225, 1163, 1133, 1075. **HRMS** (ESI): $C_{20}H_{27}FN_2O_3$ [MH]$^+$; calculated 363.2078, found 363.2068.

---

[*] Analysis of the crude product by ^{1}H NMR spectroscopy at 500 MHz showed a 60:40 mixture of **S33** and **S34**.

5.4.2.6.3 Preparation of scaffold S35

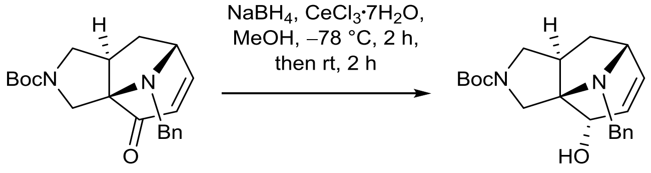

2f **S35** 70%

***tert*-Butyl (1*R**,5*R**,7*R**,10*S**)-11-benzyl-10-hydroxy-3,11-diazatricyclo[5.3.1.0^{1,5}]undec-8-ene-3-carboxylate S35**

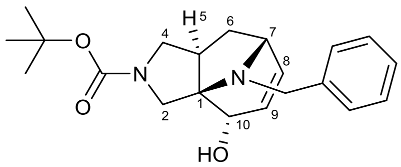
S35

To a solution of cycloadduct **2f** (2.52 g, 7.11 mmol, 1.00 mmol) in MeOH (35 mL) at –78 °C was added $CeCl_3 \cdot 7H_2O$ (3.18 g, 8.53 mmol, 1.20 eq.) followed by $NaBH_4$ (323 mg, 8.53 mmol, 1.20 eq.). The resulting solution was stirred at –78 °C for 2 h and then warmed to rt over 2 h. EtOAc (100 mL) and H_2O (50 mL) were added and the layers were separated. The aqueous layer was extracted with EtOAc (2 x 50 mL). The combined organic layers were washed with brine (100 mL), dried over $MgSO_4$, filtered, and concentrated *in vacuo*. Flash chromatography eluting with 4:1 CH_2Cl_2–EtOAc gave the *title compound* **S35** (1.97 g, 5.53 mmol, 70%) as a colourless solid. **Mp:** 159– 162 °C. **^{1}H NMR** (500 MHz, $CDCl_3$): 7.26-7.04 (5H, m, Bn Ar-H), 5.74 (1H, dd, *J* 9.7, 4.6, 8-H), 5.56 (1H, d, *J* 9.7, 9-H), 4.34 (1H, app. s, 10-H), 3.78 (1H, d, *J* 19.3, C$H_A$$H_B$Ph), 3.69-3.57 (2H, m, C$H_A$$H_B$Ph and 4-$H_A$), 3.50-3.41 (1H, m, 2-$H_A$), 3.33 (1H, d, *J* 11.8, 2-H_B), 3.28-3.16 (2H, m, 4-H_B and 7-H), 3.09-2.96 (1H, m, 5-H), 1.89 (1H, app. t, *J* 10.0, 6-H_A), 1.62-1.48 (1H, m, 6-H_B), 1.36 (9H, s, $C_q(CH_3)_3$). **^{13}C NMR** (100 MHz, $CDCl_3$, mixture of two rotamers): 154.7 (N(CO)O), 154.6 (N(CO)O), 140.3 (2 peaks, Ar-C_q), 133.3 (8-C), 132.8 (8-C), 128.4 (Ar-C), 128.3 (Ar-C), 128.2 (Ar-C), 127.7 (9-C), 127.4 (9-C), 127.0 (Ar-C), 79.4 ($C_q(CH_3)_3$), 75.4 (1-C), 74.5 (1-C), 67.0 (10-C), 66.8 (10-C), 58.4 (7-C), 58.2 (7-C), 54.5 (4-C), 54.1 (4-C), 49.8 (2-C), 48.7 (2-C), 40.4 (5-C), 39.6 (5-C), 38.5 (6-C), 38.2 (6-C), 28.7 ($C_q(CH_3)_3$) [28 of 34 expected peaks observed]. **IR** ν_{max}(film)/cm^{-1} 3400, 2973, 1693, 1669, 1417, 1171. **HRMS** (ESI): $C_{21}H_{29}N_2O_3$ [MH]$^+$; calculated 357.2173, found 357.2169.

5.4.3 Preparation of fragments

Note that the following fragments from the manuscript are detailed here using the aliases given below in parentheses:

- Compound **12** (**F12**)
- Compound **21** (**F8**)
- Compound **22** (**F31**)
- Compound **23** (**F48**)
- Compound **24** (**CF1**)
- Compound **25** (**F32**)

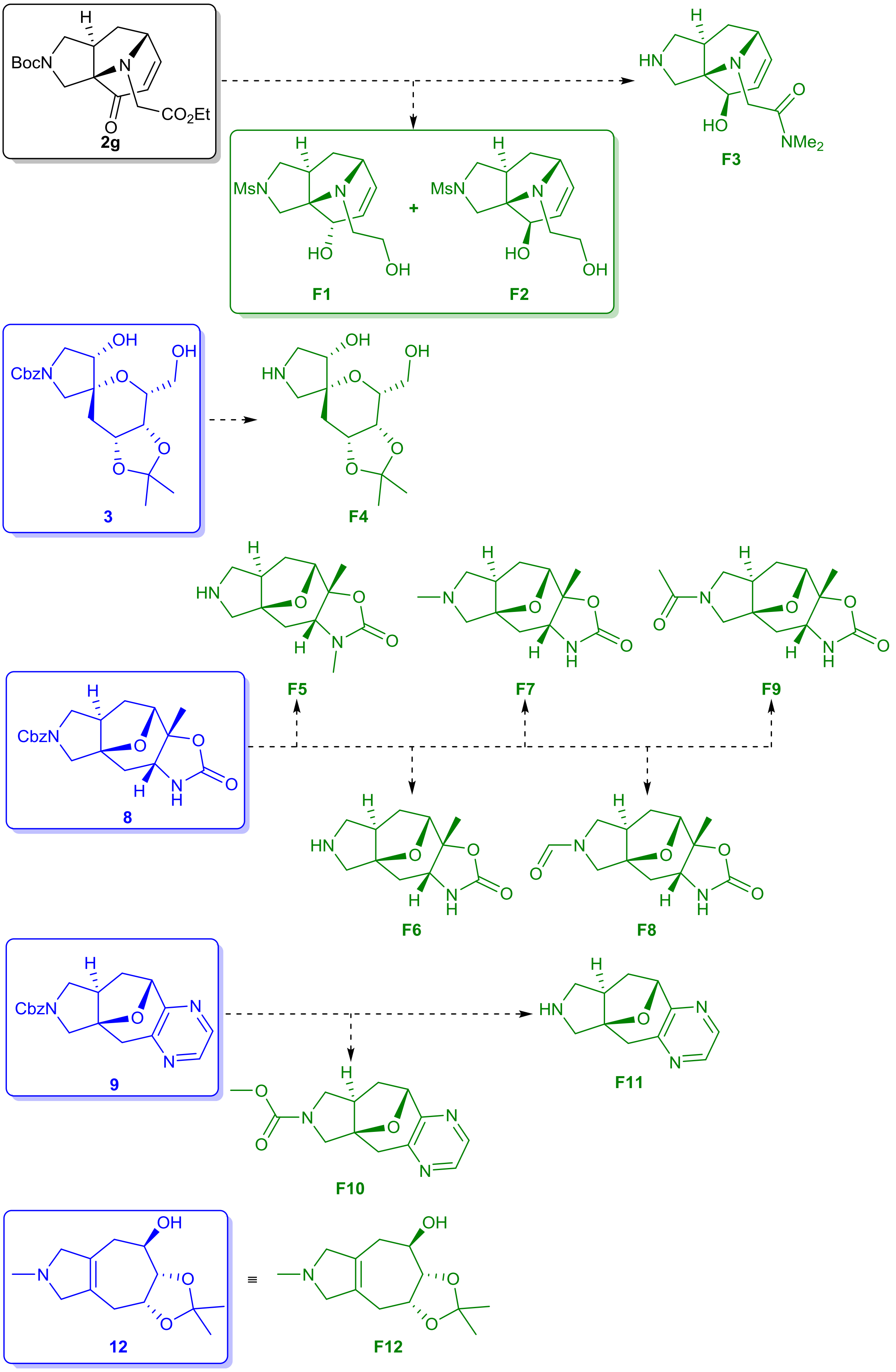
BocN
H
N
O
CO2Et
2g
HN
H
N
HO
O
NMe2
F3
MsN
H
N
HO
OH
F1
+
MsN
H
N
HO
OH
F2
CbzN
OH
O
OH
O
O
3
HN
OH
O
OH
O
O
F4
HN
H
O
O
H
N
O
F5
N
H
O
O
H
N
H
O
F7
O
N
H
O
O
H
N
H
O
F9
CbzN
H
O
O
H
N
H
O
8
HN
H
O
O
H
N
H
O
F6
O
N
H
O
O
H
N
H
O
F8
CbzN
H
O
N
N
9
HN
H
O
N
N
F11
O
O
N
H
O
N
N
F10
N
OH
O
O
12
≡
N
OH
O
O
F12

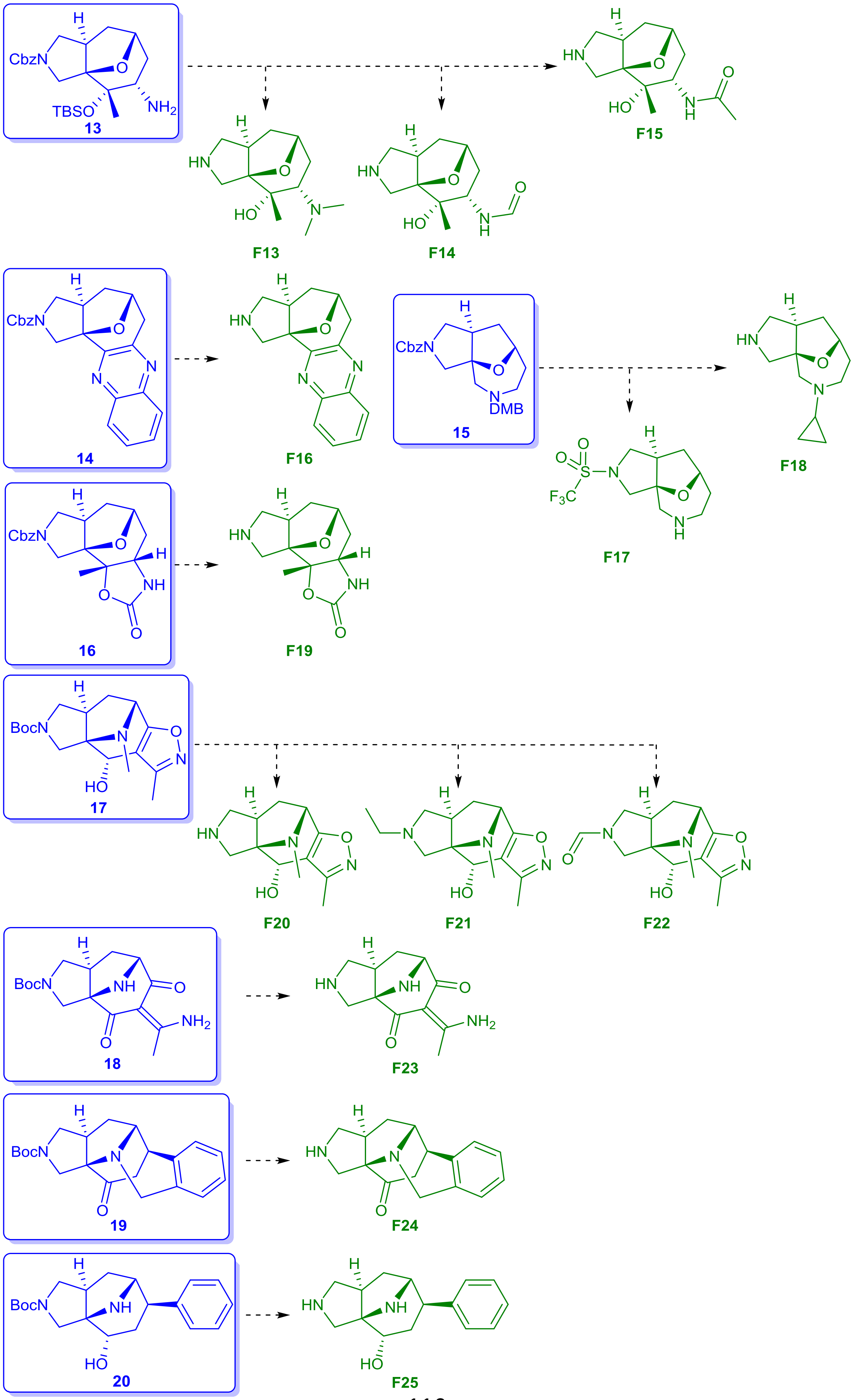
CbzN
TBSO
NH2
13
F13
F14
F15
14
F16
15
DMB
F17
F18
16
F19
BocN
17
F20
F21
F22
18
F23
19
F24
20
F25

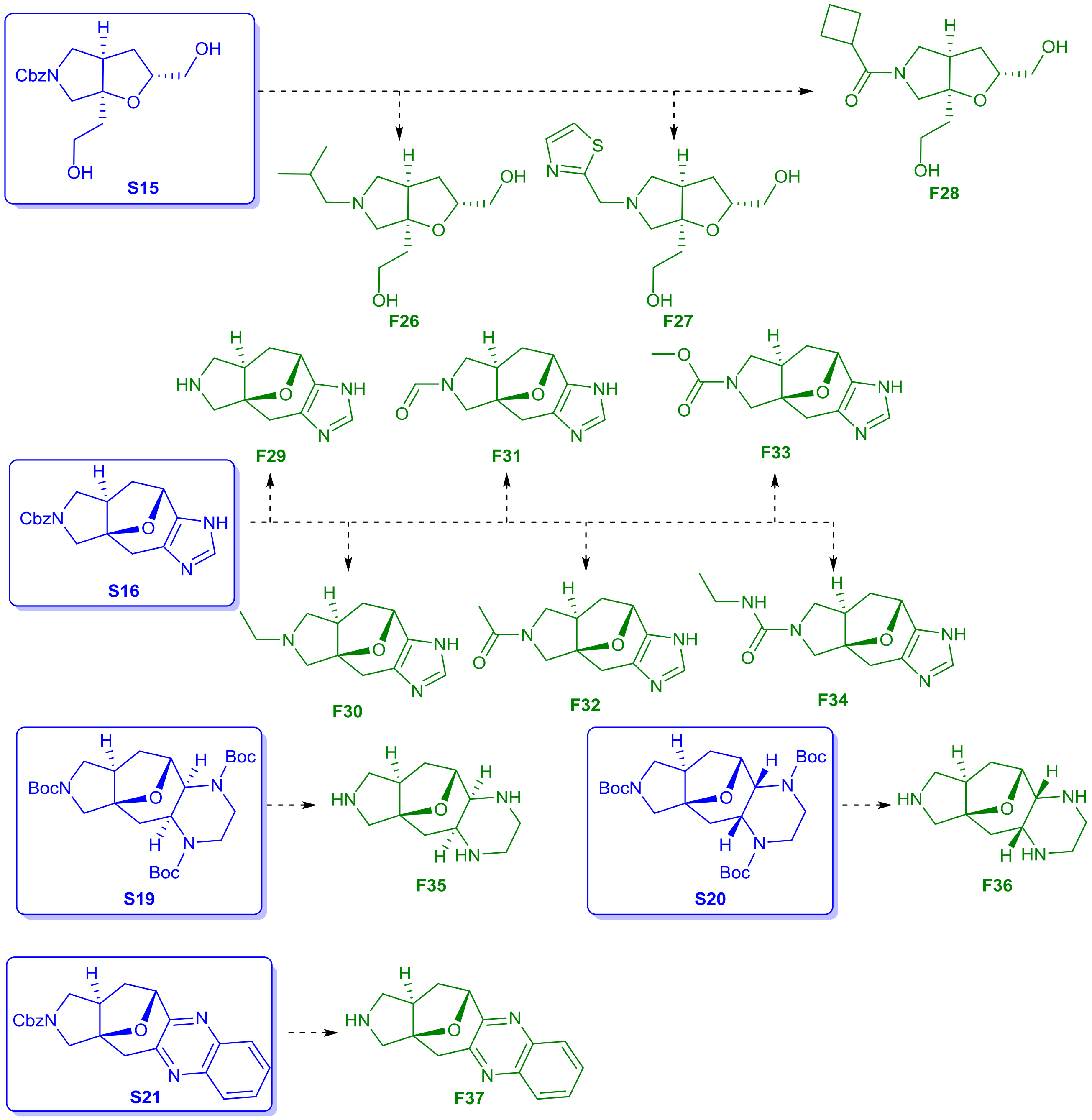

S15
F26
F27
F28
S16
F29
F30
F31
F32
F33
F34
S19
F35
S20
F36
S21
F37

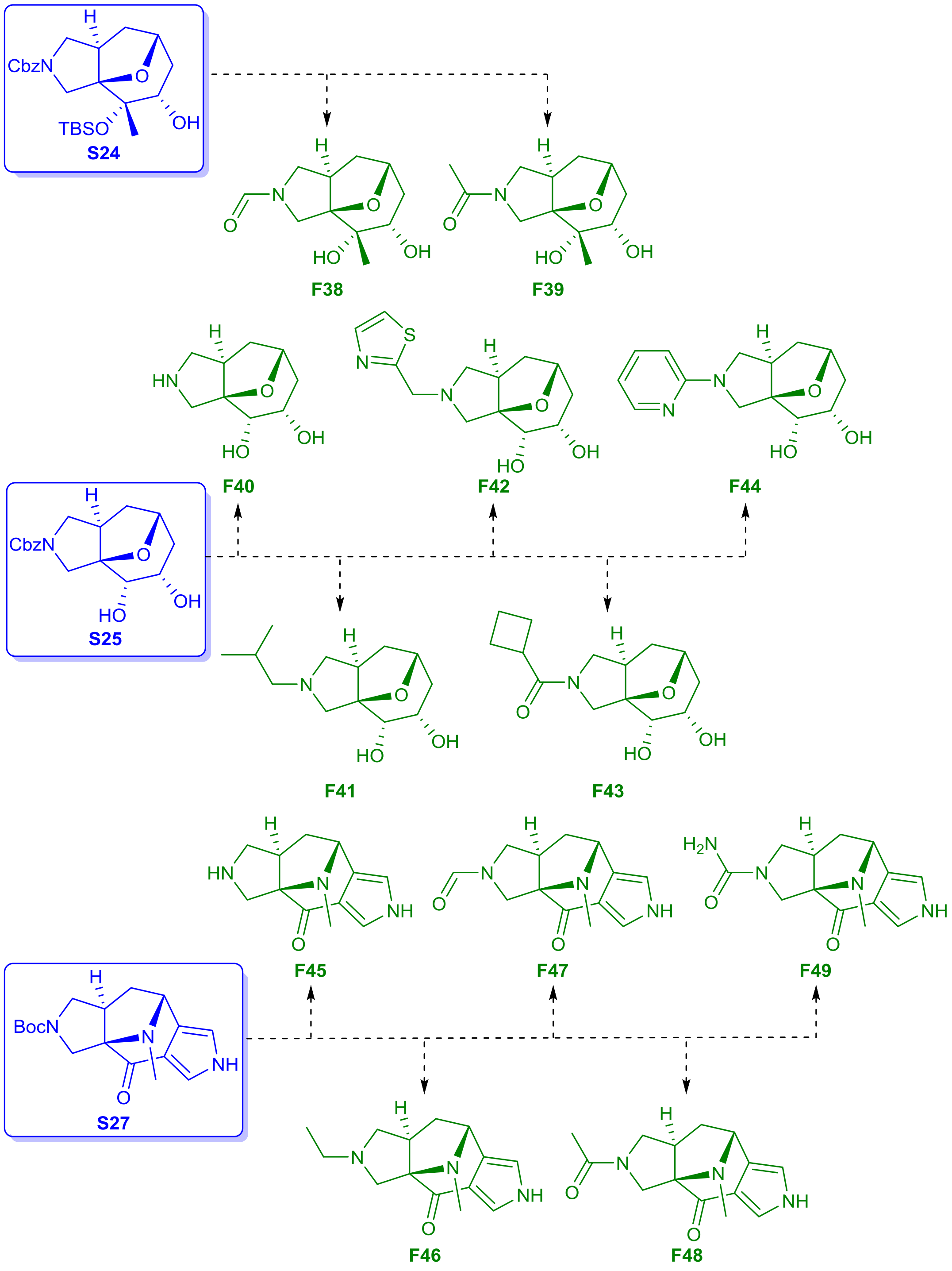
CbzN
TBSO
OH
S24
F38
F39
HN
F40
F42
F44
S25
F41
F43
F45
F47
H_2N
F49
BocN
NH
S27
F46
F48

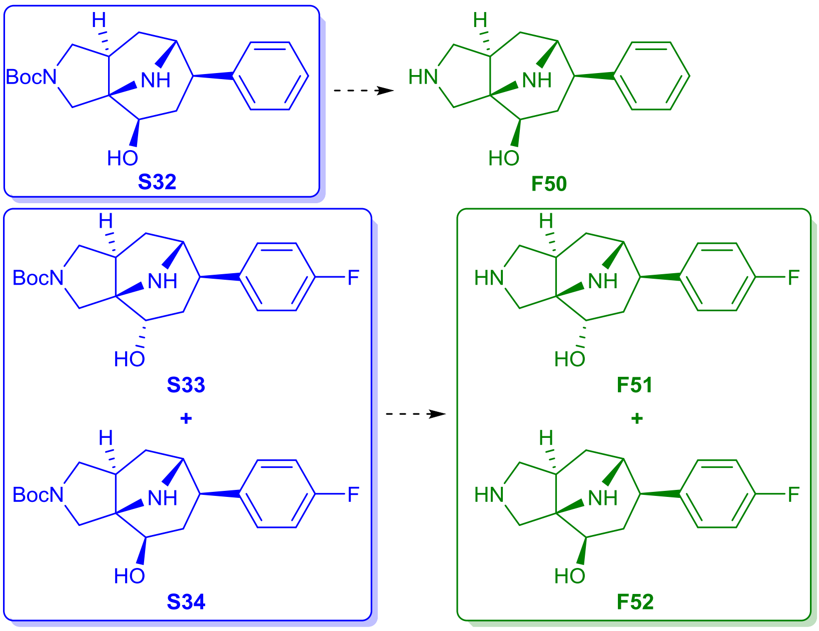
BocN
NH
HO
H
S32
HN
F50
S33
+
S34
F
F51
F52

5.4.3.1 Preparation of fragments derived from cycloadduct 2g

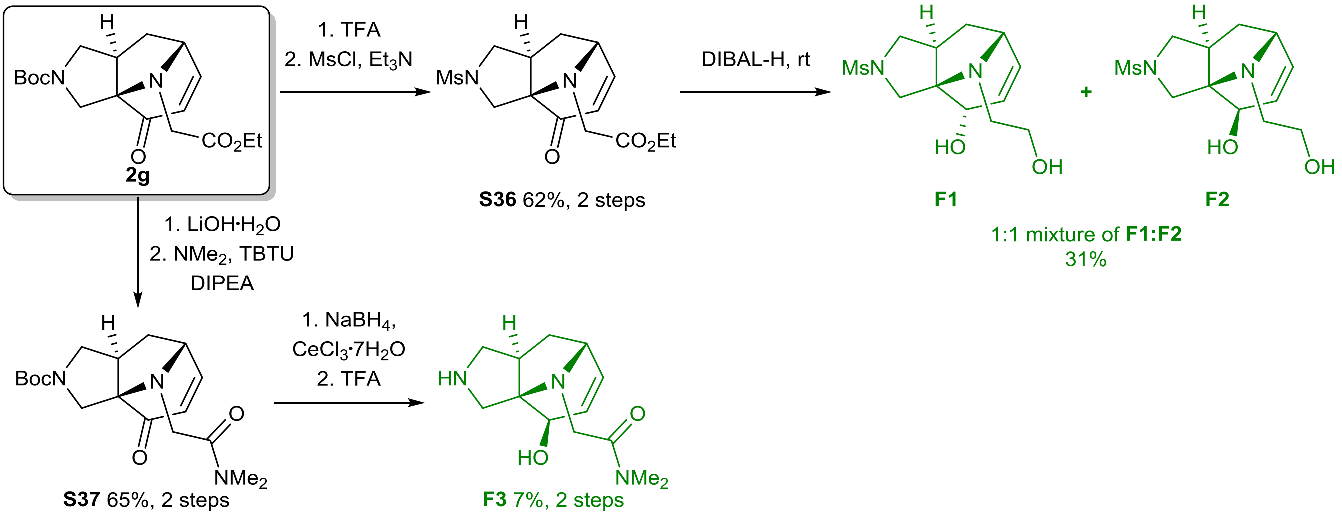

Ethyl 2-[(1*R,5*R**,7*R**)-3-methanesulfonyl-10-oxo-3,11-diazatricyclo[5.3.1.0^{1,5}]undec-8-en-11-yl]acetate S36**

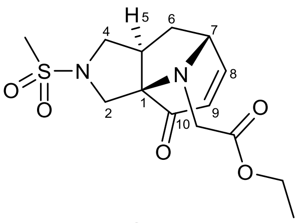

S36

Deprotection of the Boc-protected cycloadduct **2g** (119 mg, 0.74 mmol) was carried out by following general procedure **I**. The reaction mixture was concentrated *in vacuo* and the crude residue was used directly in the next step. MsCl (0.11 mL, 1.47 mmol, 3.20 eq.) was added to a stirred solution of the residue and Et_3N (0.61 mL, 9.7 eq.). The resulting solution was stirred at rt for 2 h. H_2O (10 mL) and CH_2Cl_2 (10 mL) were added and the phases separated. The aqueous phase was extracted with CH_2Cl_2 (2 x 10 mL) and the combined organic layers were dried over $MgSO_4$, filtered and evaporated *in vacuo* to give a crude brown oil. Flash chromatography eluting with 3:2 CH_2Cl_2–EtOAc gave the *title compound* **S36** (150 mg, 0.46 mmol, 62% over two steps). **^{1}H NMR** (500 MHz, MeOD-d_4, 333 K): δ 7.01 (1H, dd, *J* 9.8, 4.7, 8-H), 5.96 (1H, d, *J* 9.8, 9-H), 4.17 (1H, app. t, *J* 5.4, 7-H), 4.07 (2H, q, *J* 7.1, $CO_2CH_2CH_3$), 4.02 (1H, d, *J* 11.8, 2-H_A), 3.77 (1H, dd, *J* 10.2, 8.6, 4-H_B), 3.31 (1H, d, *J* 16.6, $NCH_AH_BCO_2Et$), 3.27 (1H, dd, *J* 10.2, 8.5, 4-H_A), 3.22 (1H, d, *J* 16.6, $NCH_AH_BCO_2Et$), 3.12 (1H, *J* 11.8, 2-H_B), 2.81 (3H, s, NSO_2CH_3), 2.61 (1H, app. qd, *J* 8.5, 5.0, 5-H), 2.01 (1H, dd, *J* 12.3, 5.3, 6-H_A), 1.95 (1H, dd, *J* 12.3, 8.5, 6-H_B), 1.15 (3H, t, *J* 7.1, $CO_2CH_2CH_3$). **^{13}C NMR** (125 MHz, MeOD-d_4, 333 K): δ 195.9, 172.2, 152.2, 127.8, 83.2, 63.0, 62.1, 55.5, 49.8, 48.4, 46.9, 35.3, 34.1, 14.4. **IR** ν_{max}(film)/cm^{-1} 1737, 1677, 1329, 1150, 1022. **HRMS** (ESI): $C_{14}H_{21}N_2O_5S$ $[M+H]^+$; calculated 329.1171, found 329.1166.

(1*R,5*R**,7*R**,10*S**)-11-(2-Hydroxyethyl)-3-methanesulfonyl-3,11-diazatricyclo[5.3.1.0^{1,5}]undec-8-en-10-ol F1 and (1*R**,5*R**,7*R**,10*R**)-11-(2-Hydroxyethyl)-3-methanesulfonyl-3,11-diazatricyclo[5.3.1.0^{1,5}]undec-8-en-10-ol F2**

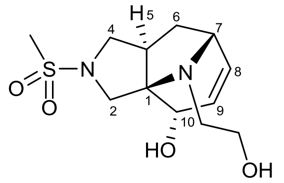

F1

+

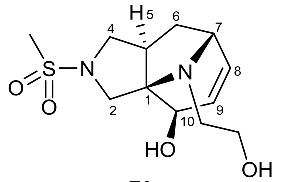

F2

To a solution of compound **S36** (150 mg, 0.46 mmol, 1.00 eq.) in THF (2.5 mL) at rt was added DIBAL (1.0 M in hexanes, 2.29 mL, 2.29 mmol, 5.00 eq.). The resulting solution was stirred for 2 h and then Rochelle's salt (sat. aq. solution, 10 mL) and CH_2Cl_2 (10 mL) were added. The resulting biphasic solution was stirred rapidly for 2 h before the layers were separated. The aqueous phase was extracted with CH_2Cl_2 (2 x 10 mL) and the combined organic phases were washed with brine (10 mL), dried over $MgSO_4$, filtered and evaporated *in vacuo* to give a crude colourless oil. Flash chromatohraphy eluting with 16:1:0.1 CH_2Cl_2–EtOH–NH_4OH gave the *title compounds* **F1** and **F2** (41 mg, 0.14 mmol, 31%) as a 1:1 mixture of diastereomers. **^{1}H NMR** (500 MHz, MeOD-d$_4$, 333 K, 2 × OH not observed): δ 5.96 (0.45H, ddd, *J* 9.8, 5.0, 1.3, 8-H$_{min}$), 5.85 (0.55H, ddd, *J* 9.8, 4.4, 1.6, 8-H$_{maj}$), 5.79 (0.45H, dd, *J* 9.8, 4.0, 9-H$_{min}$), 5.60 (0.55H, dd, 9.8, 2.2, 9-H$_{maj}$), 4.34 (0.55H, app. s, 10-H$_{maj}$), 3.82 (0.45H, app. t, *J* 5.5, 7-H$_{min}$), 3.71-3.47 (4.9H, m, 4-H$_{B\text{-}maj}$, 4-H$_{B\text{-}min}$, 7-H$_{maj}$, 10-H$_{min}$, NC*H*$_2$CH$_2$OH$_{min}$, NCH$_2$C*H*$_2$OH$_{min}$ and NCH$_2$C*H*$_2$OH$_{maj}$), 3.39 (0.55H, d, *J* 11.0, NC*H*$_A$H$_B$CH$_2$OH$_{maj}$), 3.35 (0.55H, d, *J* 11.0, NCH$_A$*H*$_B$CH$_2$OH$_{maj}$), 3.23 (0.55H, dd, *J* 10.0, 6.8, 4-H$_{A\text{-}maj}$), 3.20 (0.45H, dd, *J* 9.8, 7.3, 4-H$_{A\text{-}min}$), 3.18-3.11 (0.55, m, 5-H$_{maj}$), 2.95-2.84 (1.45H, m, 2-H$_{A\text{-}maj}$, 2-H$_{min}$), 2.91 (1.35H, s, NSO$_2$CH$_{3(min)}$)), 2.90 (1.65H, s, NSO$_2$CH$_{3(maj)}$)), 2.79 (0.55H, ddd, *J* 13.3, 7.9, 6.0, 2-H$_{B\text{-}maj}$), 2.41-2.29 (0.45H, m, 5-H$_{min}$), 2.01 (0.55H, dd, *J* 11.6, 8.7, 6-H$_{A\text{-}maj}$), 1.91 (0.45H, dd, *J* 11.7, 8.6, 6-H$_{A\text{-}min}$), 1.78-1.68 (1H, m, 6-H$_{B\text{-}maj}$ and 6-H$_{B\text{-}min}$). **^{13}C NMR** (125 MHz, MeOD-d$_4$, 333 K, one C not observed): δ 133.5, 132.8, 129.5, 127.6, 77.1, 76.3, 70.2, 66.8, 62.1, 62.0, 60.7, 60.6, 56.3, 56.0, 54.4, 52.9, 48.0, 46.6, 41.9, 38.8, 38.2, 35.0, 34.8 [23 of 24 expected peaks observed]. **IR** ν_{max}(film)/cm^{-1} 3330, 1643, 1409, 1013. **HRMS** (ESI): $C_{12}H_{21}N_2O_4S$ [M+H]$^+$; calculated 289.1222, found 289.1229.

***tert*-Butyl (1*R**,5*R**,7*R**)-11-[(dimethylcarbamoyl)methyl]-10-oxo-3,11-diazatricyclo[5.3.1.0^{1,5}]undec-8-ene-3-carboxylate S37**

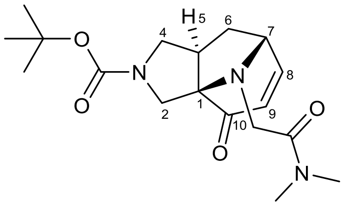

S37

To a stirred solution of cycloadduct **2g** (124 mg, 0.35 mmol, 1.00 eq.) in 1:1 THF–H_2O (2.0 mL) at rt was added LiOH·H_2O (74 mg, 1.77 mmol, 5.00 eq.). The resulting suspension was stirred at rt for 3 days. CH_2Cl_2 (10 mL) and H_2O (10 mL) was added and the phases separated. The aqueous phase was acidified to pH 3 and then extracted with 9:1 CH_2Cl_2–

MeOH (3 x 10 mL). The combined organic layers were dried over $MgSO_4$, filtered, and cncentrated *in vacuo* to give the crude carboxylic acid as a clear, colourless oil. This crude material was carried forward to the next reaction without further purification {**^{1}H NMR** (500 MHz, MeOD-d_4, 333 K, CO_2H not observed): δ 7.01 (1H, dd, *J* 9.8, 4.8, 8-H), 5.94 (1H, d, *J* 9.8, 9-H), 4.14 (1H, m, 7-H), 4.00 (1H, d, *J* 12.5, 2-H_A), 3.82 (1H, dd, *J* 10.9, 9.3, 4-H_B), 3.30 (1H, dd, *J* 10.9, 8.1, 4-H_A), 3.28 (1H, d, *J* 16.9, $NCH_AH_BCO_2H$), 3.17 (1H, d, *J* 12.5, 2-H_B), 3.17 (1H, d, *J* 16.9, $NCH_AH_BCO_2H$), 2.52 (1H, app. qd, *J* 8.6, 4.7, 5-H), 2.01 (1H, ddd, *J* 12.3, 6.1, 4.8, 6-H_B), 1.95 (1H, dd, *J* 12.3, 8.6, 6-H_A), 1.36 (9H, s, $C_q(CH_3)_3$). **LRMS** (HP-LCMS): $C_{16}H_{23}N_2O_5$ $[M+H]^+$; found 323.1}. To the crude residue in CH_2Cl_2 (2.0 mL) was added $NHMe_2$ (2.0 M in THF, 0.21 mL, 0.43 mmol, 1.20 eq.), TBTU (136 mg, 0.43 mmol, 1.20 eq.) and DIPEA (92 μL, 0.53 mmol, 1.50 eq.). The resulting solution was stirred at rt for 3 h. H_2O (5 mL) and CH_2Cl_2 (5 mL) were added and the phases separated. The aqueous phase was extracted with CH_2Cl_2 (2 x 5 mL) and the combined organic layers were dried over $MgSO_4$, filtered, and concentrated *in vacuo* to give a crude oil. Flash chromatography eluting with 80:10:1 CH_2Cl_2–EtOH–NH_4OH gave the *title compound* **S37** (80 mg, 0.23 mmol, 65%) as a yellow oil. **^{1}H NMR** (500 MHz, MeOD-d_4, 333 K): δ 7.11 (1H, dd, *J* 9.8, 4.8, 8-H), 6.06 (1H, d, *J* 9.8, 9-H), 4.14 (1H, app. td, *J* 4.9, 1.9, 7-H), 4.09 (1H, d, *J* 12.8, 2-H_A), 3.93 (1H, dd, *J* 10.7, 9.4, 4-H_B), 3.53 (1H, d, *J* 14.2, $NCH_AH_BCO_2(NCH_3)_2$), 3.41-3.36 (1H, m, 4-H_A), 3.40 (1H, d, *J* 12.8, 2-H_B), 3.36 (1H, d, *J* 14.2, $NCH_AH_BCO_2(NCH_3)_2$), 3.06 (3H, s, NCH_3), 2.94 (3H, s, NCH_3), 2.70-2.60 (1H, m, 5-H), 2.14-2.03 (2H, m, 6-H), 1.48 (9H, s, $C_q(CH_3)_3$). **^{13}C NMR** (125 MHz, MeOD-d_4, 333 K): δ 195.1, 170.5, 154.6, 150.8, 126.2, 88.0, 79.8, 60.8, 52.9, 45.7, 44.1, 36.1, 34.7, 34.0, 27.4. **IR** ν_{max}(film)/cm^{-1} 1635, 1633, 1409, 1166, 1129. **HRMS** (ESI): $C_{18}H_{27}N_3NaO_4$ $[M+Na]^+$; calculated 372.1894, found 372.1898.

2-[(1*R,5*R**,7*R**,10*R**)-10-Hydroxy-3,11-diazatricyclo[5.3.1.0^{1,5}]undec-8-en-11-yl]-N,N-dimethylacetamide F3**

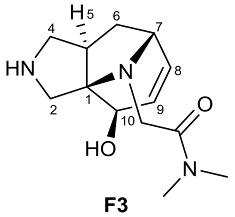

F3

To a solution of compound **S37** (136 mg, 0.39 mmol, 1.00 eq) in MeOH (2.0 mL) at –78°C was added $CeCl_3·7H_2O$ (174 mg, 0.47 mmol, 1.20 eq.) followed by $NaBH_4$ (18 mg, 0.47 mmol, 1.20 eq.). The resulting solution was stirred for 2 h, then H_2O (10 mL) and CH_2Cl_2 (10 mL) were added. The layers were separated and the aqueous phase was extracted with CH_2Cl_2 (2 x 10 mL). The combined organic phases were dried over $MgSO_4$, filtered, and concentrated *in vacuo*. The crude residue was purified by SCX SPE eluting with MeOH, then sat. NH_3/MeOH to give the crude Boc-protected aminoalcohol. The resulting Boc-protected amine was then deprotected by following general procedure **I**. Purification by SCX SPE eluting with MeOH, then sat. NH_3/MeOH, gave the *title compound* **F3** (13 mg, 52 μmol, 13%) as a brown oil. **^{1}H NMR** (500 MHz, MeOD-d_4, 333 K): δ 5.70

(1H, dd, *J* 9.7, 4.2, 8-H), 5.54 (1H, d, *J* 9.7, 9-H), 4.30 (1H, app. s, 10-H), 3.47-3.37 (3H, m, 7-H and C*H*$_2$CO$_2$(NCH$_3$)$_2$), 2.97 (3H, s, NCH$_3$), 2.90-2.79 (5H, m, 4-H$_B$, 5-H and NCH$_3$), 2.73-2.69 (1H, m, 4-H$_A$), 2.69 (1H, d, *J* 12.4, 2-H$_A$), 2.63 (1H, d, *J* 12.4, 2-H$_B$), 1.93 (1H, dd, *J* 11.4, 8.7, 6-H$_A$), 1.60 (1H, app. dt, *J* 11.4, 5.8, 6-H$_B$). **^{13}C NMR** (125 MHz, MeOD-d$_4$, 333 K): δ 173.2, 131.9, 130.1, 78.1, 66.3, 61.3, 55.0, 50.4, 47.9, 43.0, 39.6, 37.0, 36.0. **IR** ν_{max}(film)/cm^{-1} 13373, 1628, 1407, 1140. **HRMS** (ESI): $C_{13}H_{22}N_3O_2$ [M+Na]$^+$; calculated 252.1712, found 252.1718.

5.4.3.2 Preparation of fragments derived from scaffold 3

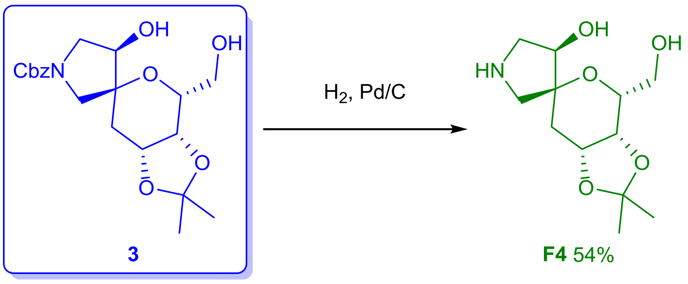

3
F4 54%

(*3aR,*4R**,*4'S**,*6R**,*7aR**)-4-(hydroxymethyl)-2,2-dimethyl-tetrahydro-2H-spiro[[1,3]dioxolo[4,5-c]pyran-6,3'-pyrrolidine]-4'-ol F4**

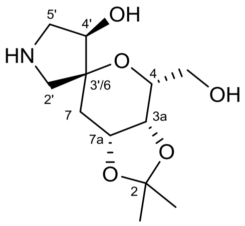

F4

Hydrogenation was carried out following general procedure **B**, using compound **3** (87 mg, 0.22 mmol) and Pd/C (10 mg, 10% w/w) in MeOH (10 mL) over 15 h. Filtration followed by concentration gave a colourless oil, which was triturated with pentane, then EtOAc, to give the *title compound* **F4** (31 mg, 0.12 mmol, 54%) as a colourless amorphous solid. **^{1}H NMR** (500 MHz, MeOD-d$_4$, 333 K, NH and one O*H* not observed): δ 4.50-4.47 (1H, m, 7a-H), 4.15 (1H, dd, *J* 6.8, 1.7, 3a-H), 3.92 (1H, dd, *J* 6.2, 5.3, 4'-H), 3.81-3.67 (3H, m, includes: 1H, 4-H; and 2H, C*H*$_2$OH), 3.08 (1H, d, *J* 12.0, 2'-H$_A$), 3.04 (1H, dd, *J* 11.5, 6.2, 5'-H$_A$), 2.84 (1H, dd, *J* 11.5, 5.3, 5'-H$_B$), 2.68 (1H, d, *J* 12.0, 2'-H$_B$), 1.95 (1H, dd, *J* 14.9, 5.9, 7-H$_A$), 1.89-1.82 (2H, m, 7-H$_B$ and OH), 1.48 (3H, s, (CH$_3$)$_A$), 1.32 (3H, s, (CH$_3$)$_B$). **^{13}C NMR** (125 MHz, MeOD-d$_4$, 333 K): δ 110.0 (2-C), 81.5 (4-C), 79.0 (3'-C/6-C), 73.8 (4'-C), 73.5 (3a-C), 72.4 (7a-C), 63.0 (CH$_2$OH), 54.4 (2'-C), 52.6 (5'-C), 32.7 (7-C), 27.0 ((CH$_3$)$_A$), 25.2 ((CH$_3$)$_B$). **IR** ν_{max}(film)/cm^{-1} 3312 (br., OH), 2983, 2933, 2876, 1543, 1417, 1382, 1216. **HRMS** (ESI): $C_{12}H_{22}NO_5$ [M+H]$^+$; calculated 260.1498, found 260.1491.

5.4.3.3 Preparation of fragments derived from scaffold 7

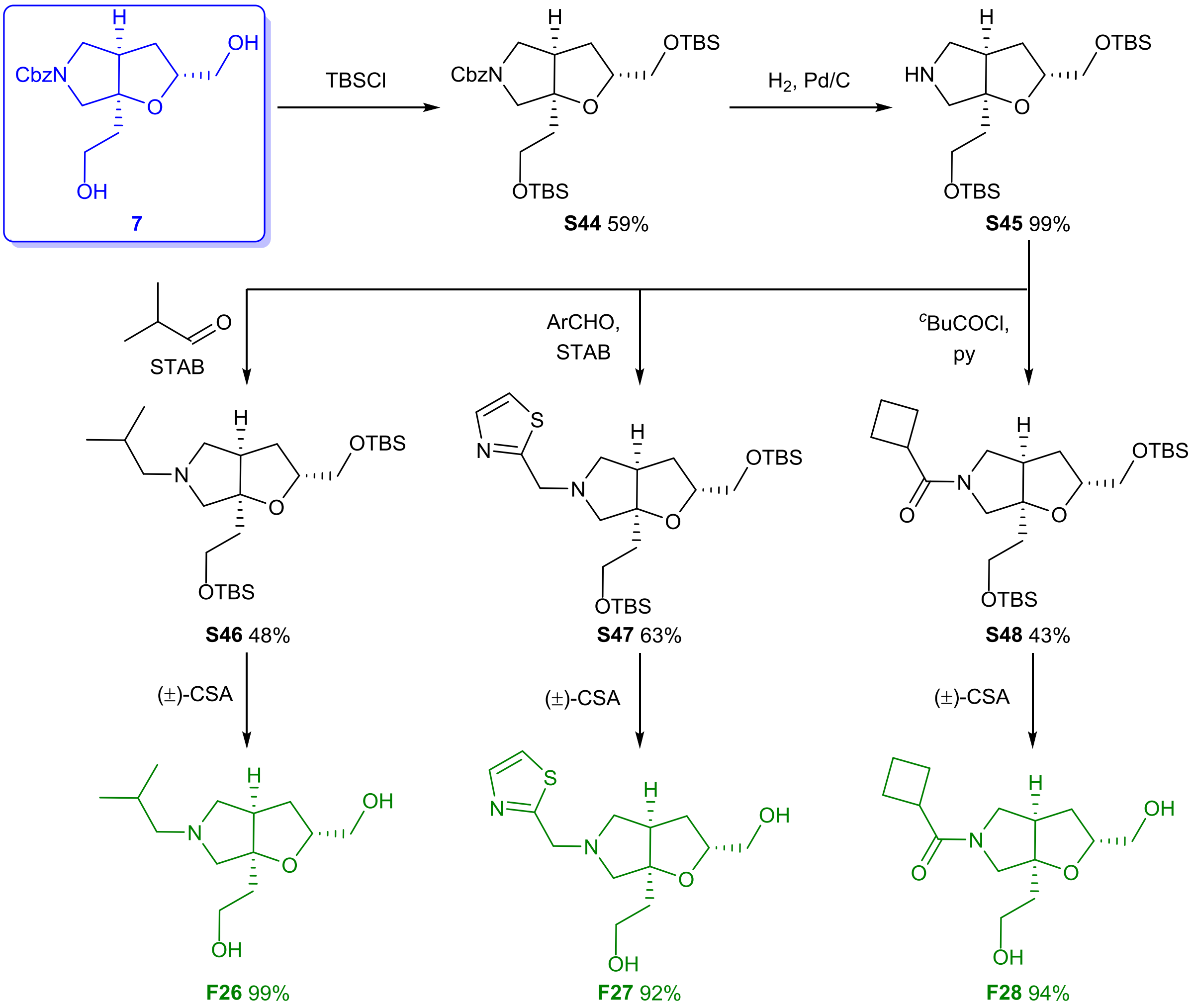

Benzyl (*2R*,3aR*,6aR)-6a-{2-[(*tert*-butyldimethylsilyl)oxy]ethyl}-2-{[(*tert*-butyldimethylsilyl)oxy]methyl}-hexahydro-2H-furo[2,3-c]pyrrole-5-carboxylate S44**

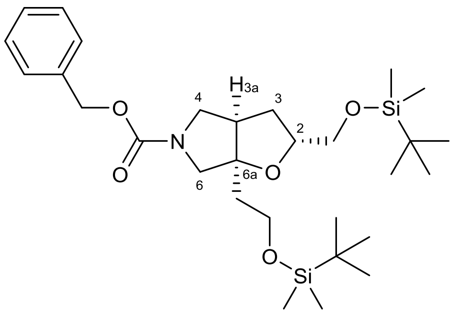

S44

TBSCl (1.03 g, 6.85 mmol, 2.2 eq.) and imidazole (548 mg, 8.10 mmol, 2.6 eq.) were added to a stirred solution of diol **7** (1.0 g, 3.1 mmol, 1.0 eq.) in CH_2Cl_2. The reaction mixture was stirred for 24 h, then partitioned with sat. aq. NH_4Cl (20 mL). The phases were separated and the aqeuous layer was washed with CH_2Cl_2 (2 × 10 mL). The combined organic phases were washed with brine (20 mL), then dried over Na_2SO_4 and concentrated *in vacuo*. Flash chromatography eluting with 9:1 pentane–EtOAc gave the *title compound* **S44** (1.01 g, 1.84 mmol, 59%) as a colourless oil. **R*f*** 0.40 (9:1 pentane–EtOAc). **^{1}H NMR** (500 MHz, $CDCl_3$, mixture of rotamers): δ 7.39-7.27 (5H, m, Cbz Ar-H), 5.16-5.05 (2H, m, OC*H*$_2$Ph), 4.29-4.11 (1H, m, 2-H), 3.81-3.66 (4H, m, 4-H$_A$, 6-H$_A$ and C$_q$CH$_2$C*H*$_2$OTBS), 3.66-3.57 (2H, m, CHC*H*$_2$OTBS),

3.51-3.41 (1H, m, 6-H_B), 3.36-3.19 (1H, m, 4-H_B), 2.76-2.59 (1H, m, 3a-H), 2.10-1.93 (1H, m, 3-H_A), 1.89-1.72 (3H, m, 3-H_B and $C_qCH_2CH_2$OTBS), 0.89 (12H, s, $SiC_q(CH_3)_3$), 0.85 (6H, s, $SiC_q(CH_3)_3$), 0.05 (3H, s, $SiCH_3$), 0.05 (4H, s, $SiCH_3$), 0.03 (5H, s, $SiCH_3$). **^{13}C NMR** (125 MHz, $CDCl_3$, mixture of two rotamers, one Ar-C not observed): δ 154.9 (N(CO)O), 154.7 (N(CO)O), 137.0 (Ar-C_q), 128.6 (Ar-C), 128.0 (Ar-C), 91.5 (6a-C), 90.6 (6a-C), 79.6 (2-C), 79.5 (2-C), 66.9 (O*C*H_2Ph), 65.6 (CH*C*H_2OTBS), 65.4 (CH*C*H_2OTBS), 59.5 (C_qCH_2*C*H_2OTBS), 59.4 (C_qCH_2*C*H_2OTBS), 57.1 (6-C), 56.9 (6-C), 51.2 (4-C), 51.0 (4-C), 47.2 (3a-C), 46.6 (3a-C), 40.9 (C_q*C*H_2CH_2OTBS), 40.7 (C_q*C*H_2CH_2OTBS), 33.5 (3-C), 33.2 (3-C), 26.1 ($SiC_q(CH_3)_3$), 26.0 ($SiC_q(CH_3)_3$), 18.5 (SiC_q), 18.3 (SiC_q), −5.2 ($SiCH_3$), −5.3 ($SiCH_3$) [30 of 42 expected peaks observed]. **IR** ν_{max}(film)/cm^{-1} 2953, 2928, 2857, 1708 (CO) 1418, 1254, 1097, 836. **HRMS** (ESI): $C_{29}H_{51}NNaO_5Si_2$ [M+H]$^+$; calculated 572.3204, found 572.3205.

(*2R*,3aR*,6aR)-6a-{2-[(*tert*-Butyldimethylsilyl)oxy]ethyl}-2-{[(*tert*-butyldimethylsilyl)oxy]methyl}-hexahydro-2H-furo[2,3-c]pyrrole S45**

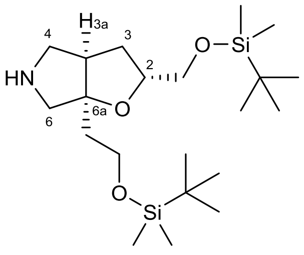

S45

Hydrogenation was carried out following general procedure **B**, using carbamate **S44** (1.15 g, 2.09 mmol) and Pd/C (100 mg, 10% w/w) in EtOH (20 mL) over 15 h. Filtration followed by concentration gave the *title compound* **S45** (867 mg, 2.09 mmol, 99%) as a colourless amorphous solid, which was not purified further. **^{1}H NMR** (500 MHz, $CDCl_3$, mixture of rotamers, NH not observed): δ 4.54-4.47 (1H, m, 2-H), 3.83-3.75 (1H, m, C_qCH_2C*H_A*H_BOTBS), 3.75-3.67 (2H, m, CHC*H_A*H_BOTBS and $C_qCH_2CH_A$*H_B*OTBS), 3.58-3.47 (3H, m, 4-H_A, 6-H_A and CHCH_A*H_B*OTBS), 3.27-3.20 (2H, m, 4-H_B and 6-H_B), 2.82-2.75 (1H, m, 3a-H), 2.21 (1H, ddd, *J* 12.8, 9.9, 7.3, 3-H_A), 1.93-1.82 (4H, m, 3-H_B and C_qC*H_2*CH_2OTBS), 0.90-0.86 (18H, m, $SiC_q(CH_3)_3$), 0.05-0.02 (12H, m, $SiCH_3$). **^{13}C NMR** (125 MHz, $CDCl_3$): δ 91.1 (6a-C), 80.3 (2-C), 64.2 (CH*C*H_2OTBS), 59.0 (C_qCH_2*C*H_2OTBS), 55.4 (6-C), 50.0 (4-C), 46.9 (3a-C), 39.7 (C_q*C*H_2CH_2OTBS), 32.4 (3-C), 26.1 (2 peaks, $SiC_q(CH_3)_3$), 18.5 (SiC_q), 18.4(SiC_q), −5.2 ($SiCH_3$), −5.3 (2 peaks, $SiCH_3$), −5.4 ($SiCH_3$). **IR** ν_{max}(film)/cm^{-1} 2953, 2928, 2857, 1471, 1463, 1254, 1094, 836. **HRMS** (ESI): $C_{21}H_{46}NO_3Si_2$ [M+H]$^+$; calculated 416.3016, found 416.3011.

($2R^*$,$3aR^*$,$6aR^*$)-6a-{2-[(*tert*-Butyldimethylsilyl)oxy]ethyl}-2-{[(*tert*-butyldimethylsilyl)oxy]methyl}-5-(2-methylpropyl)-hexahydro-2H-furo[2,3-c]pyrrole S46

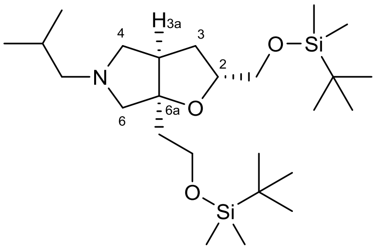

S46

Reductive amination was carried out by following general procedure **C**, using compound **S45** (293 mg, 0.71 mmol) and isobutyraldehyde (2.5 eq.). The crude reaction mixture was partitioned between EtOAc (25 mL) and brine (25 mL). The phases were separated and the aqueous phase was extracted with EtOAc (2 × 25 mL). The combined organic phases were dried over $MgSO_4$, filtered and concetrated *in vacuo*. Flash chromatography eluting with 100:2.5:1 pentane–EtOAc–NEt_3 gave the *title compound* **S46** (162 mg, 0.34 mmol, 48%) as a colourless oil. **R*f*** 0.30 (100:2.5:1 pentane–EtOAc–NEt_3). **^{1}H NMR** (500 MHz, $CDCl_3$): δ 4.29-4.20 (1H, m, 2-H), 3.78-3.69 (2H, m, $C_qCH_2CH_2$OTBS), 3.67 (1H, dd, *J* 10.6, 4.6, CHCH_AH_BOTBS), 3.59 (1H, dd, *J* 10.6, 4.9, CHCH_AH_BOTBS), 2.73 (1H, d, *J* 9.9, 6-H_A), 2.53-2.47 (2H, m, 3a-H and 4-H_A), 2.46-2.40 (1H, m, 4-H_B), 2.27 (1H, d, *J* 9.9, 6-H_B), 2.10-2.01 (2H, m, N$CH_2CH(CH_3)_2$), 1.95-1.87 (1H, m, $C_qCH_AH_BCH_2$OTBS), 1.87-1.74 (2H, m, 3-H_A and $C_qCH_AH_BCH_2$OTBS), 1.71-1.60 (2H, m, 3-H_B and $NCH_2CH(CH_3)_2$), 0.91-0.86 (24H, m, includes: 18H, $SiC_q(CH_3)_3$; and 6H, $NCH_2CH(CH_3)_2$), 0.05 (6H, s, $SiCH_3$), 0.04 (6H, s, $SiCH_3$). **^{13}C NMR** (125 MHz, $CDCl_3$, mixture of 2 rotamers): δ 91.1 (6a-C), 80.3 (2-C), 66.8 (6-C), 65.7 (CHCH_2OTBS), 64.4 (N$CH_2CH(CH_3)_2$), 61.8 (4-C), 60.1 ($C_qCH_2CH_2$OTBS), 46.7 (3a-C), 41.8 ($C_qCH_2CH_2$OTBS), 36.4 (3-C), 27.2 ($NCH_2CH(CH_3)_2$), 26.1 (2 peaks, $SiC_q(CH_3)_3$), 21.1 ($NCH_2CH(CH_3)_2$), 21.0 ($NCH_2CH(CH_3)_2$), 18.6 (SiC_q), 18.4 (SiC_q), −5.1 (2 peaks, $SiCH_3$) [19 of 36 expected peaks observed]. **IR** ν_{max}(film)/cm^{-1} 2953, 2928, 2857, 2771, 1472, 1463, 1253, 1092. **HRMS** (ESI): $C_{25}H_{54}NO_3Si_2$ [M+H]$^+$; calculated 472.3642, found 472.3647.

2-{[($2R^*$,$3aR^*$,$6aR^*$)-6a-{2-[(*tert*-Butyldimethylsilyl)oxy]ethyl}-2-{[(*tert*-butyldimethylsilyl)oxy]methyl}-hexahydro-2H-furo[2,3-c]pyrrol-5-yl]methyl}-1,3-thiazole S47

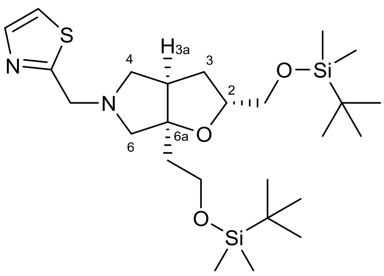

S47

Reductive amination was carried out by following general procedure **C**, using compound **S45** (270 mg, 0.65 mmol) and 1,3-thiazolecarbaldehyde (2.5 eq.). The crude reaction mixture was partitioned between EtOAc (25 mL) and brine (25 mL). The phases were separated and the aqueous phase was extracted with EtOAc (2 × 25 mL). The combined organic phases were dried over $MgSO_4$, filtered and concetrated *in vacuo*. Flash chromatography eluting with 4:1 pentane–EtOAc gave the *title compound* **S47** (209 mg, 0.41 mmol, 63%) as a colourless oil. **R*f*** 0.39 (4:1 pentane–EtOAc).

^{1}H NMR (500 MHz, $CDCl_3$, 50:50 mixture of rotamers): δ 7.68 (1H, d, *J* 3.3, thiazole 4-H), 7.27 (1H, d, *J* 3.3, thiazole 5-H), 4.41-4.26 (1H, m, 2-H), 3.91 (1H, d, *J* 15.0, 6-H_A), 3.86 (1H, d, *J* 15.0, 6-H_B), 3.79-3.71 (2H, m, $C_qCH_2CH_2OTBS$), 3.69 (1H, dd, *J* 10.7, 4.5, $CHCH_AH_BOTBS$), 3.62 (1H, dd, *J* 10.7, 4.8, $CHCH_AH_BOTBS$), 2.98 (1H, d, *J* 9.7, NCH_AH_BAr), 2.73 (1H, dd, *J* 9.1, 3.1, 4-H_B), 2.67 (1H, app. t, *J* 8.1, 4-H_A), 2.61 (1H, td, *J* 7.6, 2.6, 3a-H), 2.53 (1H, d, *J* 9.7, NCH_AH_BAr), 1.97-1.81 (3H, m, 3-H_A and $C_qCH_2CH_2OTBS$), 1.74 (1H, dd, *J* 12.4, 5.3, 3-H_B), 0.90 (9H, s, $SiC_q(CH_3)_3$), 0.88 (9H, s, $SiC_q(CH_3)_3$), 0.07 (6H, 2 × s, $SiCH_3$), 0.04 (6H, s, $SiCH_3$). **^{13}C NMR** (125 MHz, $CDCl_3$, mixture of 2 rotamers): δ 171.3 (thiazole 2-C), 142.2 (thiazole 4-C), 141.6 (thiazole 4-C), 119.5 (thiazole 5-C), 91.3 (6a-C), 80.7 (2-C), 66.6 (NCH_2Ar), 65.7 ($CHCH_2OTBS$), 61.3 (4-C), 59.9 ($C_qCH_2CH_2OTBS$), 56.8 (6-C), 47.1 (3a-C), 41.6 ($C_qCH_2CH_2OTBS$), 36.1 (3-C), 26.2 ($SiC_q(CH_3)_3$), 26.1 ($SiC_q(CH_3)_3$), 18.6 (SiC_q), 18.4 (SiC_q), −5.1 ($SiCH_3$), −5.2 ($SiCH_3$) [20 of 38 expected peaks observed]. **IR** ν_{max}(film)/cm^{-1} 2953, 2928, 2795, 1472, 1254, 1138, 1093, 836. **HRMS** (ESI): $C_{25}H_{49}N_2O_3SSi_2$ $[M+H]^+$; calculated 513.3002, found 513.3002.

(*2R,*3aR**,*6aR**)-6a-{2-[(*tert*-Butyldimethylsilyl)oxy]ethyl}-2-{[(*tert*-butyldimethylsilyl)oxy]methyl}-5-cyclobutanecarbonyl-hexahydro-2H-furo[2,3-c]pyrrole S48**

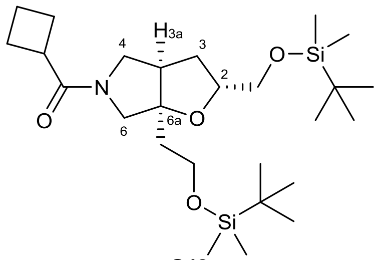

S48

Cyclobutanecarbonyl chloride (0.42 mL, 3.66 mmol, 5.00 eq.) was added to a stirred solution of compound **S45** (304 mg, 0.73 mmol, 1.00 eq.) in pyridine (10 mL) at 0 °C. The reaction mixture was warmed to rt then stirred for 15 h. The reaction mixture was concentrated *in vacuo*, then partitioned between EtOAc (25 mL) and sat. aq. $NaHCO_3$ solution (25 mL). The phases were separated and the aqueous phase was extracted with EtOAc (2 × 25 mL). The combined organic phases were dried over $MgSO_4$, filtered and concetrated *in vacuo*. Flash chromatography eluting with 7:3 pentane–EtOAc gave the *title compound* **S48** (155 mg, 0.31 mmol, 43%) as a colourless oil. **R_f** 0.15 (4:1 pentane–EtOAc).**^{1}H NMR** (500 MHz, $CDCl_3$, 330 K, 25:75 mixture of rotamers): δ 4.21-4.08 (1H, m, 2-H), 3.86-3.54 (6H, m, includes: 1H, 4-H_A; 1H, 6-H_A; 2H, $CHCH_2OTBS$; and 2H, $C_qCH_2CH_2OTBS$), 3.52-3.39 (1.75H, m, includes: 0.75H, 4-H_B; and 1H, 6-H_B), 3.26-3.08 (1.25H, m, includes: 0.25H, 4-H_B; and 1H, cyclobutyl 1-H), 2.81-2.67 (0.25H, m, 3a-H), 2.67-2.56 (0.75H, m, 3a-H), 2.41-2.24 (2H, m, cyclobutyl 2-H), 2.21-2.07 (2H, m, cyclobutyl 2-H), 2.07-1.74 (6H, m, includes: 2H, 3-H; 2H, cyclobutyl 3-H; and 2H, $C_qCH_2CH_2OTBS$), 0.92 (6H, s, $SiC_q(CH_3)_3$), 0.90 (12H, s, $SiC_q(CH_3)_3$) 0.09-0.04 (12H, m, $SiCH_3$). **^{13}C NMR** (125 MHz, $CDCl_3$, 300 K, mixture of rotamers): δ 173.2 (N(CO)CH), 172.9 (N(CO)CH), 91.4 (6a-C), 89.8 (6a-C), 79.7 (2-C), 79.2 (2-C),

65.5 (CHCH_2OTBS), 65.2 (CH*C*H_2OTBS), 59.3 ($C_q$$CH_2$*C*$H_2$OTBS), 59.2 ($C_q$$CH_2$*C*$H_2$OTBS), 57.0 (6-C), 56.2 (6-C), 50.9 (4-C), 50.4 (4-C), 47.2 (3a-C), 45.9 (3a-C), 40.8 (C_q*C*$H_2$$CH_2$OTBS), 40.5 ($C_q$*C*$H_2$$CH_2$OTBS), 38.2 (cyclobutyl 1-C), 38.1 (cyclobutyl 1-C), 33.6 (3-C), 33.1 (3-C), 25.9 (3 peaks, $SiC_q(CH_3)_3$), 24.7 (2 peaks, cyclobutyl 2-C), 24.6 (cyclobutyl 2-C), 18.4 (SiC_q), 18.2 (SiC_q), 18.1 (cyclobutyl 3-C), 18.0 (cyclobutyl 3-C), −5.4 (3 peaks, $SiCH_3$), −5.5 ($SiCH_3$). **IR** ν_{max}(film)/cm^{-1} 2952, 2929, 2857, 1645 (CO), 1432, 1254, 1095, 836. **HRMS** (ESI): $C_{26}H_{52}NO_4Si_2$ [M+H]$^+$; calculated 498.3435, found 498.3440.

2-[(*2R,*3aR**,*6aR**)-2-(Hydroxymethyl)-5-(2-methylpropyl)-hexahydro-2H-furo[2,3-c]pyrrol-6a-yl]ethan-1-ol F26**

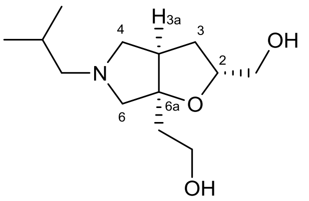

F26

O-Silyl deprotection was carried out by following general procedure **G**, using compound **S46** (270 mg, 0.65 mmol) and (±)-camphorsulfonic acid (6.0 eq.) Flash chromatography eluting with 50:8:1 CH_2Cl_2–EtOH–NH_3/MeOH gave the *title compound* **F26** (82 mg, 0.34 mmol, 99%) as a colourless oil. **R*f*** 0.50 (50:8:1 CH_2Cl_2–EtOH–NH_3/MeOH). **^{1}H NMR** (500 MHz, $CDCl_3$, 2 × OH not observed): δ 4.36-4.24 (1H, m, 2-H), 3.87-3.75 (3H, m, CHC*H*$_A$H$_B$OH and $C_q$$CH_2$C*H*$_2$OH), 3.56 (1H, dd, *J* 11.7, 4.4, CHCH$_A$*H*$_B$OH), 2.80-2.75 (1H, m, 4-H$_A$), 2.66-2.54 (3H, m, includes: 1H, 3a-H; at δ 2.64: 1H, d, *J* 9.9, 6-H$_A$; and at δ 2.57: 1H, d, *J* 9.9, 6-H$_B$), 2.34 (1H, dd, *J* 9.4, 5.0, 4-H$_B$), 2.13 (2H, d, *J* 7.3, NC*H*$_2$CH(CH_3)$_2$)), 2.01-1.90 (3H, m, 3-H$_A$ and C_qC*H*$_2$$CH_2$OH), 1.75-1.61 (2H, m, includes: 1H, NCH_2C*H*(CH_3)$_2$; and at δ 1.65: 1H, dd, *J* 12.4, 5.0, 3-H$_B$), 0.90 (6H, d, *J* 6.6, NCH_2CH(C*H*$_3$)$_2$). **^{13}C NMR** (125 MHz, $CDCl_3$): δ 93.1 (6a-C), 79.5 (2-C), 65.8 (6-C), 64.3 (N*C*H_2CH(CH_3)$_2$), 63.8 (CH*C*H_2OH), 61.6 (4-C), 60.2 ($C_q$$CH_2$*C*$H_2$OH), 46.5 (3a-C), 40.5 ($C_q$*C*$H_2$$CH_2$OH), 34.2 (3-C), 27.1 ($NCH_2$*C*H($CH_3$)$_2$), 21.0 (2 peaks, N$CH_2$CH(*C*$H_3$)$_2$). **IR** ν_{max}(film)/cm^{-1} 3383 (br. s, OH), 2952, 2869, 2787, 1466, 1081, 1043, 1002. **HRMS** (ESI): $C_{13}H_{26}NO_3$ [M+H]$^+$; calculated 244.1913, found 244.1909.

2-[(*2R,*3aR**,*6aR**)-2-(Hydroxymethyl)-5-(1,3-thiazol-2-ylmethyl)-hexahydro-2H-furo[2,3-c]pyrrol-6a-yl]ethan-1-ol F27**

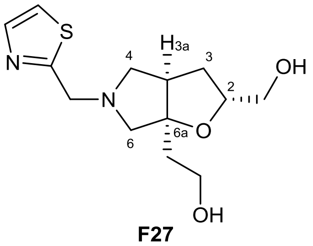

F27

O-Silyl deprotection was carried out by following general procedure **G**, using compound **S47** (208 mg, 0.41 mmol) and (±)-camphorsulfonic acid (10.0 eq.) over 30 h. The residue was partitioned between CH_2Cl_2 (20 mL) and sat. aq. $NaHCO_3$ (20 mL) and the phases were separated. The aqueous phase was extracted with CH_2Cl_2 (2 × 20 mL). The combined organic phase was dried over Na_2SO_4 and concentrated *in vacuo*. Flash chromatography eluting with 50:8:1

CH_2Cl_2–EtOH–NH_3/MeOH gave the *title compound* **F27** (107 mg, 0.38 mmol, 92%) as a yellow oil. **R_f** 0.64 (50:8:1 CH_2Cl_2–EtOH–NH_3/MeOH). **^{1}H NMR** (500 MHz, $CDCl_3$, 50:50 mixture of rotamers, 2 × OH not observed): δ 7.70 (1H, d, *J* 3.3, thiazole 4-H), 7.29 (1H, d, *J* 3.3, thiazole 5-H), 4.49-4.42 (1H, m, 2-H), 3.94 (1H, d, *J* 15.0, 6-H_A), 3.90 (1H, d, *J* 15.0, 6-H_B), 3.86 (0.5H, d, *J* 2.8, CHC$H_A$$H_B$OH), 3.84 (0.5H, d, *J* 2.8, CHC$H_A$$H_B$OH), 3.81 (2H, app. t, *J* 5.5, $C_q$$CH_2CH_2$OH), 3.58 (0.5H, d, *J* 4.2, CHC$H_A$$H_B$OH), 3.55 (0.5H, d, *J* 4.2, CHC$H_A$$H_B$OH), 3.02 (1H, d, *J* 9.6, NC$H_A$$H_B$Ar), 2.85-2.77 (1H, m, 4-H_A), 2.73 (0.5H, d, *J* 3.3, 4-H_B), 2.72 (0.5H, d, *J* 3.3, 4-H_B), 2.63 (2H, s, 3a-H and NC$H_A$$H_B$Ar), 2.11-1.98 (2H, m, 3-H_A and C_qC$H_A$$H_B$$CH_2$OH), 1.92 (0.5H, t, *J* 5.4, C_qC$H_A$$H_B$$CH_2$OH), 1.90 (0.5H, t, *J* 5.4, C_qC$H_A$$H_B$$CH_2$OH), 1.73 (0.5H, d, *J* 5.4, 3-H_B), 1.71 (0.5H, d, *J* 5.4, 3-H_B). **^{13}C NMR** (125 MHz, $CDCl_3$, one Ar-C_q not observed): 142.4 (thiazole 4-C), 119.7 (thiazole 5-C), 93.4 (6a-C), 80.7 (2-C), 66.1 (NCH_2Ar), 63.6 (CHCH_2OH), 61.2 (4-C), 60.1 ($C_q$$CH_2$$CH_2$OH), 56.4 (6-C), 47.3 (3a-C), 40.1 ($C_q$$CH_2$$CH_2$OH), 34.4 (3-C). **IR** ν_{max}(film)/cm^{-1} 3359 (br., OH), 2950, 2926, 2870, 2797, 1136, 1081, 1042. **HRMS** (ESI): $C_{13}H_{21}N_2O_3S$ [M+H]$^+$; calculated 285.1273, found 285.1271.

2-[(*2R,*3aR**,*6aR**)-5-Cyclobutanecarbonyl-2-(hydroxymethyl)-hexahydro-2H-furo[2,3-c]pyrrol-6a-yl]ethan-1-ol F28**

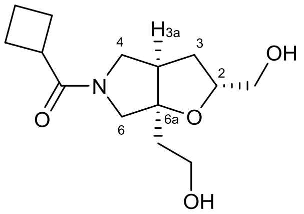

F28

O-Silyl deprotection was carried out by following general procedure **G**, using compound **S48** (166 mg, 0.330 mmol) and (±)-camphorsulfonic acid (5.0 eq.) Flash chromatography eluting with 50:8:1 CH_2Cl_2–EtOH–NH_3/MeOH gave the *title compound* **F28** (83 mg, 0.310 mmol, 94%) as a yellow oil. **R_f** 0.36 (50:8:1 CH_2Cl_2–EtOH–NH_3/MeOH). **^{1}H NMR** (500 MHz, $CDCl_3$, 330 K, 60:40 mixture of rotamers): δ 4.32-4.21 (1H, m, 2-H), 3.94-3.78 (3.4H, m, includes: 0.4H, 6-H_A; 2H, $C_q$$CH_2CH_2$OH; and 1H, CHC$H_A$$H_B$OH), 3.77-3.69 (1.2H, m, includes: 0.6H, 4-H_B; and 0.6H, 6-H_A), 3.66 (0.4H, dd, *J* 11.0, 8.8, 4-H_B), 3.54-3.41 (1.6H, m, includes: 0.6H, 4-H_A; and 1H, CHC$H_A$$H_B$OH), 3.45 (0.4H, d, *J* 12.4, 6-H_B), 3.34 (0.6H, d, *J* 12.4, 6-H_B), 3.25 (0.4H, dd, *J* 11.0, 6.1, 4-H_A), 3.21-3.11 (1H, m, cyclobutyl 1-H), 2.80 (2H, br. s, 2 × OH), 2.76-2.69 (0.4H, m, 3a-H), 2.68-2.61 (0.6H, m, 3a-H), 2.39-2.06 (5H, m, includes: 1H, 3-H_A; and 4H, cyclobutyl 2-H), 2.03-1.92 (2H, m, C_qC$H_2$$CH_2$OH), 1.92-1.76 (3H, m, includes: 1H, 3-H_B; and 2H, cyclobutyl 3-H). **^{13}C NMR** (125 MHz, $CDCl_3$, mixture of rotamers): δ 173.5 (N(*C*O)CH), 173.3 (N(*C*O)CH), 93.4 (6a-C), 92.0 (6a-C), 80.3 (2-C), 79.9 (2-C), 63.7 (2 peaks, CHCH_2OH), 60.2 ($C_q$$CH_2$$CH_2$OH), 60.0 ($C_q$$CH_2$$CH_2$OH), 57.0 (6-C), 56.3 (6-C), 51.1 (4-C), 50.9 (4-C), 47.9 (3a-C), 46.1 (3a-C), 39.4 ($C_q$$CH_2$$CH_2$OH), 39.3 ($C_q$$CH_2$$CH_2$OH), 38.3 (cyclobutyl 1-C), 38.2 (cyclobutyl 1-C), 33.0 (3-C), 32.4 (3-C), 24.9 (cyclobutyl 2-C), 24.8 (3 peaks, cyclobutyl 2-C), 18.2 (2 peaks, cyclobutyl 3-C) [28 of 28 expected peaks

observed]. **IR** ν_{max}(film)/cm^{-1} 3391, 2942, 2872, 1617, 1450, 1088, 1047. **HRMS** (ESI): $C_{14}H_{23}NNaO_4$ [M+Na]$^+$; calculated 292.1525, found 292.1525.

5.4.3.4 Preparation of fragments derived from scaffold 8

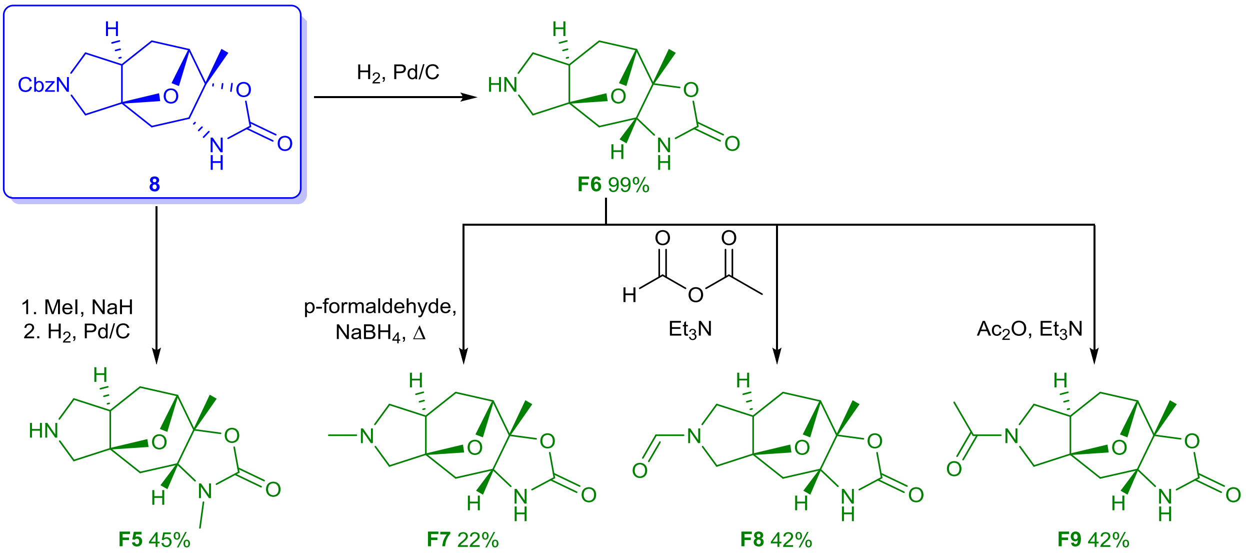

(*1R,*3R**,*7R**,*8R**,*10R**)-4,7-dimethyl-6,14-dioxa-4,12-diazatetracyclo[6.5.1.0^{1,10}.0^{3,7}]tetradecan-5-one F5**

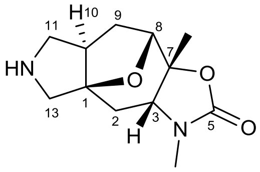

F5

NaH (60% dispersion in mineral oil, 4 mg, 100 μmol, 1.2 eq.) was added to a stirred solution of **8** (30 mg, 84 μmol, 1.0 eq.) in THF (10 mL) at 0 °C. MeI (50 μL, 0.80 mmol, 10.0 eq.) was added and the reaction mixture was stirred for 15 h. The reaction mixture was quenched by the addition of sat. aq. NH_4Cl solution (0.3 mL), then concentrated *in vacuo*. The resulting residue was flushed through a pad of SiO_2, eluting with 9:1 EtOAc–MeOH, and carried forward to the next step without further purification. The crude residue was subjected to hydrogenation following general procedure **B**, using Pd/C (10 mg, 33% w/w) and MeOH (10 mL) over 15 h. Flash chromatography eluting with 93:7:1 CH_2Cl_2–EtOH–NH_3/MeOH gave the *title compound* **F5** (9 mg, 38 μmol, 45%) as a colourless oil. **R*f*** 0.13 (50:8:1 CH_2Cl_2–EtOH–NH_3/MeOH). **^{1}H NMR** (400 MHz, MeOD-d$_4$, NH not observed): δ 4.25 (1H, d, *J* 7.2, 8-H), 3.67 (1H, dd, *J* 5.0, 1.4, 3-H), 3.11-3.01 (2H, m, includes at δ 3.08: 1H, d, *J* 12.6, 13-H$_A$; and at δ 3.05: 1H, dd, *J* 12.0, 8.4, 11-H$_B$), 2.84 (3H, s, O(CO)NCH$_3$), 2.74 (1H, dd, *J* 12.0, 3.7, 11-H$_A$), 2.64 (1H, d, *J* 12.6, 13-H$_B$), 2.52-2.44 (1H, m, 10-H), 2.32 (1H, dd, *J* 13.8, 9.2, 9-H$_A$), 2.14-2.08 (2H, m, 2-H), 1.81-1.72 (1H, m, 9-H$_B$), 1.60 (3H, s, C$_q$CH$_3$). **^{13}C NMR** (100 MHz, MeOD-d$_4$): δ 160.8 (5-C), 92.4 (1-C), 84.1 (8-C), 78.3 (7-C), 62.2 (3-C), 56.5 (11-C or 13-C), 56.2 (11-C or 13-C), 46.7 (10-C), 35.6 (9-C), 30.7 (2-C), 29.6 (O(CO)NCH$_3$), 24.8 (C$_q$CH$_3$). **IR** ν_{max}(film)/cm^{-1} 2935, 2874, 1748 (CO), 1658, 1426, 1399, 1277, 1046. **HRMS** (ESI): C12H19N2O3 [M+H]$^+$; calculated 239.1390, found 239.1392.

(*1R,*3R**,*7R**,*8R**,*10R**)-7-Methyl-6,14-dioxa-4,12-diazatetracyclo[6.5.1.0^{1,10}.0^{3,7}]tetradecan-5-on F6**

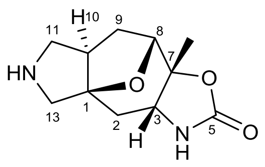

F6

Hydrogenation was carried out following general procedure **B**, using compound **8** (335 mg, 0.93 mmol, 1.0 eq.) and Pd/C (30 mg, 10% w/w) in MeOH (10 mL) over 15 h. Filtration followed by concentration gave the *title compound* **F6** (207 mg, 0.92 mmol, 99%) as a colourless foam which was carried on to the subsequent steps without further purification. **^{1}H NMR** (400 MHz, MeOD-d_4, 2 × NH not observed): δ 4.33 (1H, d, *J* 7.5, 8-H), 3.94 (1H, d, *J* 5.9, 3-H), 3.58-3.50 (2H, m, includes: 1H, 11-H_B; and at δ 3.53: 1H, d, *J* 12.4, 13-H_A), 3.23 (1H, d, *J* 12.4, 13-H_B), 3.19 (1H, dd, *J* 12.3, 3.9, 11-H_A), 3.14-3.04 (1H, m, 10-H), 2.51 (1H, dd, *J* 14.1, 9.2, 9-H_A), 2.25 (1H, dd, *J* 14.9, 5.9, 2-H_A), 2.03 (1H, d, *J* 14.9, 2-H_B), 1.96 (1H, ddd, *J* 14.1, 7.5, 4.9, 9-H_B), 1.59 (3H, s, C_qCH_3). **^{13}C NMR** (100 MHz, MeOD-d_4): δ 161.1 (5-C), 89.9 (1-C), 83.4 (8-C), 79.8 (7-C), 56.4 (3-C), 54.5 (11-C or 13-C), 54.1 (11-C or 13-C), 44.8 (10-C), 35.6 (2-C), 32.4 (9-C), 24.7 (C_qCH_3). **IR** ν_{max}(film)/cm^{-1} 3230, 2936, 2774, 1749, 1385, 1279, 1048. **HRMS** (ESI): $C_{11}H_{17}N_2O_3$ [M+H]$^+$; calculated 225.1234, found 225.1229.

(*1R,*3R**,*7R**,*8R**,*10R**)-7,12-Dimethyl-6,14-dioxa-4,12-diazatetracyclo[6.5.1.0^{1,10}.0^{3,7}]tetradecan-5-one F7**

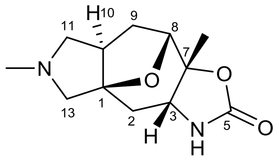

F7

Reductive amination was carried out by following general procedure **C**, using compound **F6** (37 mg, 0.17 mmol) and p-formaldehyde (5.0 eq.) at reflux. Flash chromatography eluting with 98:2:0.1 CH_2Cl_2–EtOH–NH_3/MeOH gave the *title compound* **F7** (9 mg, 38 µmol, 22%) as a colourless oil. **R_f** 0.55 (98:2:0.1 CH_2Cl_2–EtOH–NH_3/MeOH). **^{1}H NMR** (400 MHz, MeOD-d_4, NH not observed): δ 4.27 (1H, d, *J* 7.3, 8-H), 3.88 (1H, d, *J* 5.9, 3-H), 2.93-2.82 (2H, m, includes: 1H, 10-H; and at δ 2.89: 1H, d, *J* 10.7, 13-H_A), 2.72 (1H, dd, *J* 9.8, 8.6, 11-H_B), 2.58 (1H, dd, *J* 9.8, 4.8, 11-H_A), 2.47 (1H, d, *J* 10.7, 13-H_B), 2.39-2.31 (4H, m, includes: 1H, 9-H_A; and at δ 2.36: 3H, s, NCH_3), 2.14 (1H, dd, *J* 14.7, 5.9, 2-H_A), 1.92 (1H, d, *J* 14.7, 2-H_B), 1.87-1.79 (1H, m, 9-H_B), 1.58 (3H, s, C_qCH_3). **^{13}C NMR** (100 MHz, MeOD-d_4): δ 161.3 (5-C), 91.0 (1-C), 84.2 (8-C), 80.3 (7-C), 65.5 (13-C), 64.4 (11-C), 57.1 (3-C), 46.6 (10-C), 41.9 (NCH_3), 34.6 (2-C or 9-C), 34.4 (2-C or 9-C), 25.1 (C_qCH_3). **IR** ν_{max}(film)/cm^{-1} 3245, 2938, 2791, 1750 (CO), 1453, 1382, 1330. **HRMS** (ESI): $C_{12}H_{19}N_2O_3$ [M+H]$^+$; calculated 239.1390, found 239.1392.

(*1R,*3R**,*7R**,*8R**,*10R**)-7-Methyl-5-oxo-6,14-dioxa-4,12-diazatetracyclo[6.5.1.0^{1,10}.0^{3,7}]tetradecane-12-carbaldehyde F8**

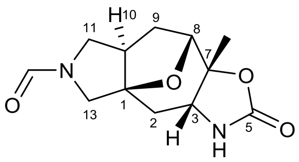

F8 ≡ 21

To pre-form acetic formic anhydride, Ac_2O (0.63 ml, 6.60 mmol) was added to HCO_2H (0.25 mL, 6.60 mmol) and the reaction mixture was heated at 60 °C for 1 h, then cooled to rt. A 0.1 mL aliquot was added to a stirred solution of **F6** (37 mg, 0.17 mmol, 1.00 eq.) and Et_3N (0.2 mL, 2.7 mmol, 16.0 eq.) in CH_2Cl_2 (10 mL) at 0 °C. The reaction mixture was warmed to rt, stirred for 1 h, then concentrated *in vacuo*. Flash chromatography eluting with 29:1:0.1 CH_2Cl_2–EtOH–NH_3/MeOH gave the *title compound* **F8** (18 mg, 71 μmol, 42%) as a colourless oil. **R_f** 0.47 (29:1:0.1 CH_2Cl_2–EtOH–NH_3/MeOH). **^{1}H NMR** (400 MHz, MeOD-d_4, NH not observed, two stable conformations observed at the pyrrolidine ring [50:50 mixture]): δ 8.14 (0.5H, s, NCHO), 8.13 (0.5H, s, NCHO), 4.33 (1H, app. t, *J* 7.6, 8-H), 4.02 (0.5H, dd, *J* 11.2, 9.2, 11-$H_{B\text{-conf1}}$), 3.94-3.88 (1.5H, m, includes: 1H, 3-H; and 0.5H, 13-$H_{A\text{-conf1}}$), 3.80 (0.5H, d, *J* 13.7, 13-$H_{A\text{-conf2}}$), 3.73 (0.5H, app. dd, *J* 12.4, 9.7, 11-$H_{B\text{-conf2}}$), 3.53 (0.5H, d, *J* 12.5, 13-$H_{B\text{-conf1}}$), 3.49-3.37 (1.5H, m, includes: 0.5H, 11-$H_{A\text{-conf1}}$; 0.5H, 11-$H_{A\text{-conf2}}$; and 0.5H, 13-$H_{B\text{-conf2}}$), 3.01-2.87 (1H, m, 10-H), 2.49 (0.5H, dd, *J* 14.2, 8.9, 9-H_A), 2.40 (0.5H, dd, *J* 14.2, 8.9, 9-H_A), 2.22 (1H, app. td, *J* 15.1, 6.1, 2-H_A), 1.98 (1H, dd, *J* 15.1, 3.5, 2-H_B), 1.97-1.85 (1H, m, 9-H_B), 1.60 (3H, s, C_qCH_3). **^{13}C NMR** (100 MHz, MeOD-d_4, mixture of two rotamers): δ 163.1 (NCHO), 162.9 (NCHO), 161.3 (2 peaks, 5-C), 89.9 (1-C), 89.4 (1-C), 84.0 (8-C), 83.7 (8-C), 80.0 (7-C), 79.9 (7-C), 56.6 (3-C), 55.6 (2 peaks, 11-C or 13-C), 52.8 (2 peaks, 11-C or 13-C), 45.5 (10-C), 45.1 (10-C), 35.5 (2-C or 9-C), 34.2 (2-C or 9-C), 33.6 (2-C or 9-C), 33.5 (2-C or 9-C), 24.7 (2 peaks, C_qCH_3) [23 of 24 expected peaks observed]. **IR** ν_{max}(film)/cm^{-1} 3270, 2936, 2886, 1751 (CO), 1652 (CO), 1429, 1387. **HRMS** (ESI): $C_{12}H_{17}N_2O_4$ [M+H]$^+$; calculated 253.1183, found 253.1184.

(*1R,*3R**,*7R**,*8R**,*10R**)-12-acetyl-7-methyl-6,14-dioxa-4,12-diazatetracyclo[6.5.1.0^{1,10}.0^{3,7}]tetradecan-5-one F9**

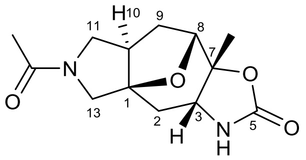

F9

N-Acylation was carried out by following general procedure **E**, using compound **F6** (37 mg, 0.17 mmol). Flash chromatography eluting with 98:2:0.1 CH_2Cl_2–EtOH–NH_3/MeOH gave the *title compound* **F9** (31 mg, 0.12 mmol, 68%) as a colourless oil. **R*f*** 0.10 (98:2:0.1 CH_2Cl_2–EtOH–NH_3/MeOH). **^{1}H NMR** (400 MHz, MeOD-d$_4$, NH not observed, 50:50 mixture of rotamers): δ 4.34 (1H, d, *J* 7.7, 8-H), 4.03 (0.5H, dd, *J* 10.7, 9.8, 11-H$_A$), 3.98-3.80 (2.5H, m, includes: 1H, 3-H; 0.5H, 11-H$_A$; and 1H, 13-H$_A$), 3.59 (0.5H, d, *J* 12.4, 13-H$_B$), 3.43-3.25 (1.5H, m, includes: 1H, 11-H$_B$; and 0.5H, 13-H$_B$), 3.04-2.84 (1H, m, 10-H), 2.43 (0.5H, app. t, *J* 7.3, 9-H$_A$), 2.40 (0.5H, app. t, *J* 7.3, 9-H$_A$), 2.23 (0.5H, dd, *J* 6.0, 5.2, 2-H$_A$), 2.20 (0.5H, dd, *J* 6.0, 5.2, 2-H$_A$), 2.10 (3H, s, Ac), 2.06 (3H, s, N(CO)C*H*$_3$), and 2.02-1.89 (2H, m, 2-H$_B$ and 9-H$_B$). **^{13}C NMR** (100 MHz, MeOD-d$_4$, mixture of two rotamers): δ 171.7 (N(CO)CH$_3$), 171.6 (N(CO)CH$_3$), 161.4 (5-C), 161.3 (5-C), 90.7 (1-C), 89.5 (1-C), 83.8 (8-C), 83.8 (8-C), 80.0 (7-C), 79.9 (7-C), 57.4 (13-C), 57.0 (13-C), 56.5 (3-C), 55.6 (11-C), 55.3 (11-C), 46.2 (10-C), 44.7 (10-C), 34.6 (2-C or 9-C), 34.2 (2-C or 9-C), 34.0 (2-C or 9-C), 33.9 (2-C or 9-C), 24.6 (N(CO)*C*H$_3$), 22.0 (C$_q$*C*H$_3$), 21.8 (C$_q$*C*H$_3$) [24 of 26 expected peaks observed]. **IR** ν_{max}(film)/cm^{-1} 3259, 2936, 2883, 1746, 1618, 1455, 972. **HRMS** (ESI): $C_{13}H_{19}N_2O_4$ [M+H]$^+$; calculated 267.1339, found 267.1343.

5.4.3.5 Preparation of fragments derived from scaffold 9

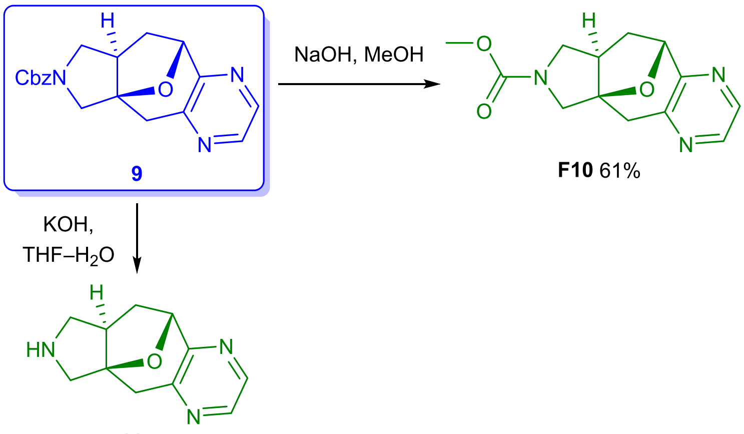

Methyl (*1R,*9R**,*11R**)-15-oxa-4,7,13-triazatetracyclo[7.5.1.0^{1,11}.0^{3,8}]pentadeca-3(8),4,6-triene-13-carboxylate F10**

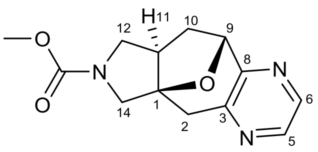

F10 ≡ 26

NaOH (250 mg, 6.25 mmol, 21.0 eq.) was added to a stirred solution of compound **9** (100 mg, 0.30 mmol, 1.00 eq.) in MeOH (3.0 mL). The reaction mixture was heated at reflux for 15 h. The reaction mixture was purified by SCX SPE eluting with MeOH, followed by flash chromatography eluting with 98:2:0.1 CH_2Cl_2–EtOH–NH_3/MeOH gave the *title compound* **F10** (48 mg, 0.18 mmol, 61%) as a pale yellow oil. **R_f** 0.04 (98:2:0.1 CH_2Cl_2–EtOH–NH_3/MeOH).**^{1}H NMR** (400 MHz, $CDCl_3$): δ 8.40 (1H, d, *J* 2.4, 5-H or 6-H), 8.29 (1H, d, *J* 2.4, 5-H or 6-H), 5.29 (1H, d, *J* 6.8. 9-H), 4.12-3.96 (1H, m, 12-H_A), 3.84 (1H, app. t, *J* 10.3, 14-H_A), 3.71 (3H, s, CO_2CH_3), 3.60-3.28 (3H, m, 2-H_A, 12-H_B and 14-H_B), 2.92 (1H, d, *J* 17.5, 2-H_B), 2.77-2.61 (1H, m, 11-H), 2.46-2.35 (1H, m, 10-H_A), 2.31-2.18 (1H, m, 10-H_B). **^{13}C NMR** (100 MHz, $CDCl_3$, mixture of two rotamers): δ 155.3 (8-C), 155.2 (N(CO)O), 149.2 (3-C), 143.5 (5-C or 6-C), 141.6 (5-C or 6-C), 91.0 (1-C), 90.1 (1-C), 80.4 (9-C), 54.9 (12-C), 54.6 (12-C), 54.1 (14-C), 54.0 (14-C), 52.6 (NCO_2CH_3), 46.6 (11-C), 45.6 (11-C), 43.2 (10-C), 39.4 (2-C) [17 of 26 expected peaks observed]. **IR** ν_{max}(film)/cm^{-1} 2953, 2876, 1696 (CO), 1450, 1390, 1117, 770, 730. **HRMS** (ESI): $C_{13}H_{16}N_3O_3$ [M+H]$^+$; calculated 262.1191, found 262.1187.

(*1R,*9R**,*11R**)-15-Oxa-4,7,13-triazatetracyclo[7.5.1.0^{1,11}.0^{3,8}]pentadeca-3(8),4,6-triene F11**

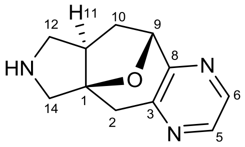

F11

KOH (340 mg, 6.10 mmol, 55.0 eq.) was added to a stirred solution of compound **9** (30 mg, 0.11 mmol, 1.00 eq.) in 7:3 THF–H_2O (2.4 mL). The reaction mixture was heated at reflux for 2 days. The reaction mixture was concentrated *in vacuo*. The residue was purified by SCX SPE following general procedure **A**, eluting with MeOH, then sat. NH_3/MeOH, followed by flash chromatography eluting with 50:8:1 CH_2Cl_2–EtOH–NH_3/MeOH to give the *title compound* **F11** (2.0 mg, 9.8 μmol, 9%) as a colourless oil. **^{1}H NMR** (400 MHz, MeOD-d_4, NH not observed): δ 8.46 (1H, d, *J* 2.7, 5-H or 6-H), 8.37 (1H, d, *J* 2.7, 5-H or 6-H), 5.19 (1H, d, *J* 6.4, 9-H), 3.43 (1H, d, *J* 17.4, 2-H_A), 3.29 (1H, d, *J* 12.7, 14-H_A), 3.10 (1H, dd, *J* 12.2, 7.7, 12-H_A), 2.98 (1H, d, *J* 17.4, 2-H_B), 2.87 (1H, app. d, *J* 12.2, 12-H_B), 2.79 (1H, d, *J* 12.7, 14-H_B), 2.62-2.46 (2H, m, 10-H_A and 11-H), 2.18-2.09 (1H, m, 10-H_B). **^{13}C NMR** (100 MHz, MeOD-d_4): δ 157.0 (8-C), 151.9 (3-C), 144.3 (5-C or 6-C), 142.6 (5-C or 6-C), 94.1 (1-C), 81.2 (9-C), 56.4 (12-C), 54.9 (14-C), 48.1 (11-C), 44.6 (10-C), 39.5 (2-C). **IR** ν_{max}(film)/cm^{-1} 3244, 3046, 2958, 2868, 1643, 1594, 1397, 1015. **HRMS** (ESI): $C_{11}H_{14}N_3O$ [M+H]$^+$; calculated 204.1131, found 204.1127.

5.4.3.6 Preparation of fragments derived from scaffold 13

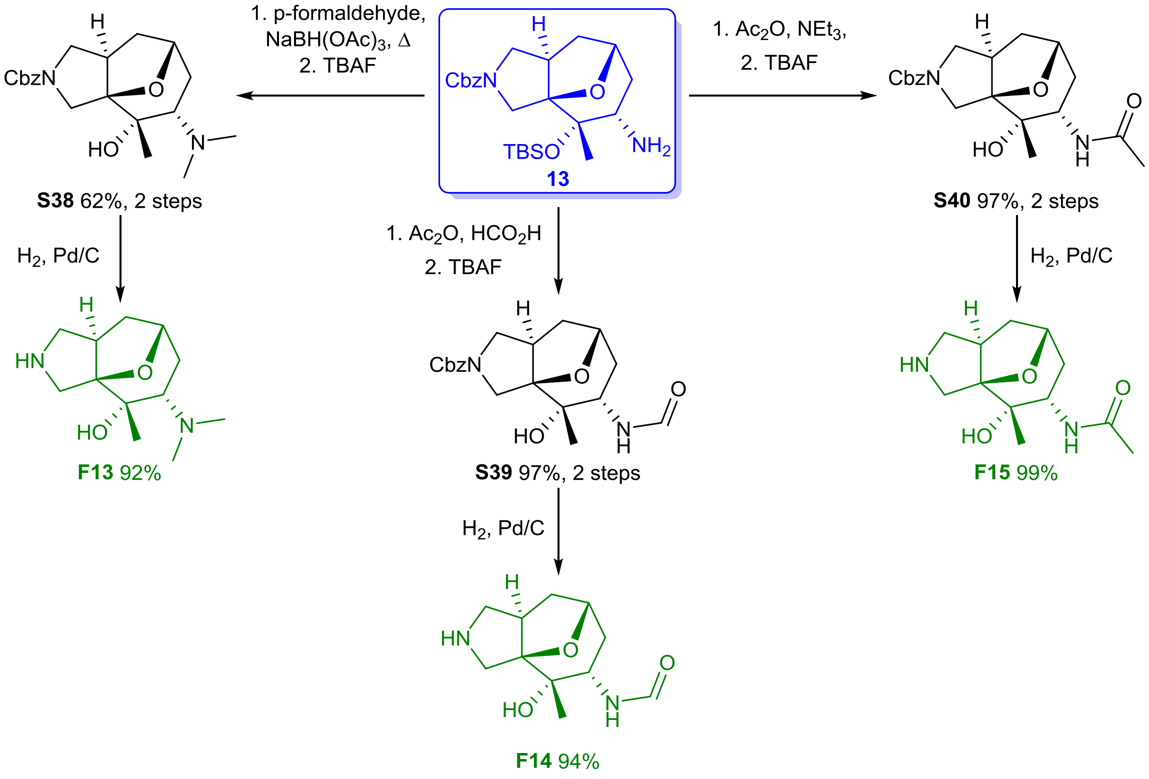

Benzyl (*1R*,5R*,7R*,9S*,10S)-9-(dimethylamino)-10-hydroxy-10-methyl-11-oxa-3-azatricyclo[5.3.1.0^{1,5}]undecane-3-carboxylate S38**

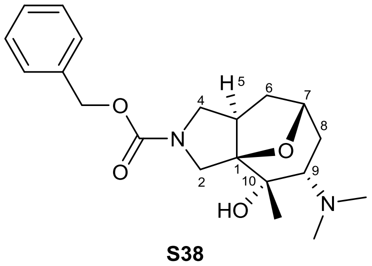

S38

Reductive amination was carried out by following general procedure **C**, using compound **13** (70 mg, 0.16 mmol), p-formaldehyde (5.0 eq.), and $NaBH(OAc)_3$ (5.0 eq.). The reaction mixture was heated at reflux for 15 h, then cooled to rt. The reaction mixture was partitioned with sat. aq. $NaHCO_3$ (10 mL). The phases were separated and the aqueous layer was extracted with CH_2Cl_2 (2 × 10 mL). The combined organic phases were washed with brine (10 mL), dried over $MgSO_4$, filtered, and concentrated *in vacuo*. The residue was diluted in THF (10 mL) and TBAF (1.0 M in THF, 0.32 mL, 0.32 mmol, 2.0 eq.) was added. The reaction mixture was stirred for 0.5 h, then concentrated *in vacuo*. Flash chromatography eluting with 29:1:0.1 CH_2Cl_2–EtOH–NH_3/MeOH gave the *title compound* **S38** (36 mg, 100 µmol, 62%, 2 steps) as a colourless oil. **R_f** 0.19 (29:1:0.1 CH_2Cl_2–EtOH–NH_3/MeOH). **^{1}H NMR** (400 MHz, $CHCl_3$, 50:50 mixture of rotamers, OH not observed) δ 7.41-7.23 (5H, m, Cbz Ar-H), 5.14 (1H, app. d, *J* 14.2, OC$H_A$$H_B$Ph), 5.08 (1H, app. d, *J* 14.2, OC$H_A$$H_B$Ph), 4.40 (1H, dd, *J* 11.5, 5.5, 7-H), 3.81-3.66 (1H, m, 4-H_A), 3.66-3.51 (2H, m, 2-H_A and 4-H_B), 3.51-3.35 (1H, m, 2-H_B), 3.14-2.95 (1H, m, 5-H), 2.48 (1H, dd, *J* 8.6, 3.2, 9-H), 2.36 (6H, s, $N(CH_3)_2$), 2.26-2.10 (2H, m, 6-H_A and 8-H_B),

1.80-1.62 (2H, m, 6-H_B and 8-H_A), 1.29 (3H, s, C_qCH_3). **^{13}C NMR** (100 MHz, $CHCl_3$, mixture of two rotamers): 155.2 (N(CO)O), 137.1 (Ar-C_q), 128.5 (Ar-C), 128.0 (2 peaks, Ar-C), 95.2 (1-C), 94.3 (1-C), 76.0 (7-C), 70.0 (10-C), 66.9 (O*C*H_2Ph), 65.9 (9-C), 54.2 (4-C), 54.0 (4-C), 52.0 (2-C), 51.5 (2-C), 45.9 (N(CH_3)$_2$), 40.7 (6-C), 39.7 (5-C), 38.8 (5-C), 27.1 (8-C), 26.1 (C_qCH_3) [21 of 36 expected peaks observed]. **IR** ν_{max}(film)/cm^{-1} 2948, 2875, 1699 (CO), 1419, 1357, 1228, 1114, 1085. **HRMS** (ESI): $C_{20}H_{29}N_2O_4$ [M+H]$^+$; calculated 361.2122, found 361.2126.

Benzyl (*1R*,5R*,7R*,9S*,10S)-9-formamido-10-hydroxy-10-methyl-11-oxa-3-azatricyclo[5.3.1.0^{1,5}]undecane-3-carboxylate S39**

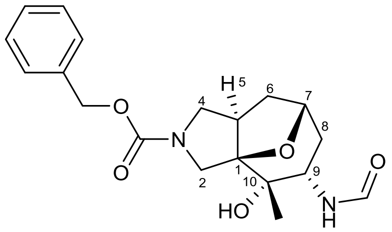

S39

N-Formylation was carried out by following general procedure **D**, using compound **13** (31 mg, 70 μmol) and Ac_2O (20 eq.). *O*-Silyl deprotection was carried out by following general procedure **H**, using the crude residue. Flash chromatography eluting with 29:1:0.1 CH_2Cl_2–EtOH–NH_3/MeOH gave the *title compound* **S39** (24 mg, 67 μmol, 97%, 2 steps) as a colourless oil. **R_f** 0.38 (29:1:0.1 CH_2Cl_2–EtOH–NH_3/MeOH). **^{1}H NMR** (400 MHz, $CHCl_3$, 50:50 mixture of rotamers, OH not observed): δ 8.19 (1H, d, *J* 3.4, NHC*H*O), 7.39-7.27 (5H, m, Cbz Ar-H), 6.42 (1H, d, *J* 8.8, CHN*H*CHO), 5.10 (2H, s, OC*H*$_2$Ph), 4.53 (1H, br. s, 7-H), 3.99-3.77 (3H, m, 2-H_A, 4-H_A and 9-H), 3.56 (1H, d, *J* 12.2, 2-H_B), 3.29-3.08 (2H, m, 4-H_B and 5-H), 2.28-2.11 (2H, m, 6-H_A, 8-H_B), 2.04 (0.5H, d, *J* 15.1, 8-H_A), 1.98-1.82 (1.5H, m, includes: 1H, 6-H_B; and at δ 1.90: 0.5H, d, *J*, 15.1, 8-H_A), 1.42 (1.5H, s, CH_3), 1.41 (1.5H, s, CH_3). **^{13}C NMR** (100 MHz, $CHCl_3$, mixture of two rotamers, 10-C not observed): δ 163.0 (NH(CO)H), 162.6 (NH(CO)H), 154.7 (N(CO)O), 136.9 (Ar-C_q), 128.6 (Ar-C), 128.2 (Ar-C), 128.1 (Ar-C), 128.0 (Ar-C), 94.6 (1-C), 93.7 (1-C), 70.5 (7-C), 69.9 (7-C), 67.1 (O*C*H_2Ph), 67.0 (O*C*H_2Ph), 55.3 (4-C), 54.9 (4-C), 53.1 (9-C), 52.9 (9-C), 51.2 (2-C), 50.5 (2-C), 40.7 (5-C), 39.5 (5-C), 37.1 (8-C), 36.6 (8-C), 33.9 (6-C), 33.6 (6-C), 25.7 (C_qCH_3), 25.5 (C_qCH_3) [28 of 34 expected peaks observed]. **IR** ν_{max}(film)/cm^{-1} 3317 (OH), 2952, 2886, 1668 (CO), 1423, 1121, 1085, 732. **HRMS** (ESI): $C_{19}H_{25}N_2O_5$ [M+H]$^+$; calculated 361.1758, found 361.1760.

Benzyl (*1R*,5R*,7R*,9S*,10S)-9-acetamido-10-hydroxy-10-methyl-11-oxa-3-azatricyclo[5.3.1.0^{1,5}]undecane-3-carboxylate S40**

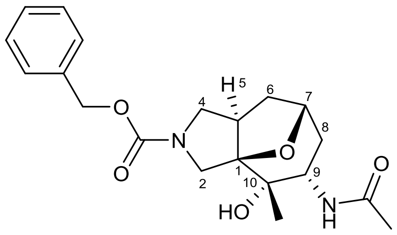

S40

N-Acylation was carried out by following general procedure **E**, using compound **13** (90 mg, 0.20 mmol). The reaction mixture was stirred at rt for 10 min, then sat. aq. NH_4Cl (10 mL) was added. The phases were separated and the aqueous layer was extracted with CH_2Cl_2 (2 × 10 mL). The combined organic phases were dried over $MgSO_4$, filtered, and concentrated *in vacuo*. *O*-Silyl deprotection was carried out by following general procedure **H**, using the crude residue. Flash chromatography eluting with 29:1:0.1 CH_2Cl_2–EtOH–NH_3/MeOH gave the *title compound* **S40** (59 mg, 0.16 mmol, 79%, 2 steps) as a colourless oil. **R_f** 0.10 (29:1:0.1 CH_2Cl_2–EtOH–NH_3/MeOH). **^{1}H NMR** (400 MHz, $CHCl_3$, 50:50 mixture of rotamers): δ 7.41-7.28 (5H, m, Cbz Ar-H), 6.10 (0.5H, app. s, NH), 6.03 (0.5H, app. s, NH), 5.11 (2H, s, OC*H*$_2$Ph), 4.53 (1H, br. s, 7-H), 4.00-3.75 (3H, m, 2-H_A, 4-H_A and 9-H), 3.57-3.55 (1H, m, 2-H_B, includes at δ 3.56: 0.5H, *J* 12.1; and at δ 3.55: 0.5H, *J* 12.1), 3.33-3.01 (3H, m, 4-H_B, 5-H and OH), 2.34-2.17 (1H, m, 8-H_B), 2.13 (1H, dd, *J* 13.2, 8.3, 6-H_A), 2.07-1.92 (4H, m includes: 1H, 6-H_B; at δ 2.03: 1.5H, s, NH(CO)C*H*$_3$, and at δ 2.01: 1.5H, s, NH(CO)C*H*$_3$), 1.89 (0.5H, d, *J* 15.0, 8-H_A), 1.78 (0.5H, d, *J* 15.0, 8-H_A), 1.42 (1.5H, s, C_qC*H*$_3$), 1.40 (1.5H, s, C_qC*H*$_3$). **^{13}C NMR** (100 MHz, $CHCl_3$, mixture of two rotamers, 10-C not observed): δ 172.4 (NH(*C*O)CH$_3$), 172.0 (NH(*C*O)CH$_3$), 154.7 (N(CO)O), 154.6 (N(CO)O), 136.9 (Ar-C_q), 136.8 (Ar-C_q), 128.6 (Ar-C), 128.1 (2 peaks, Ar-C), 127.0 (Ar-C), 94.6 (1-C), 93.7 (1-C), 70.6 (7-C), 70.0 (7-C), 67.0 (O*C*H$_2$Ph), 55.3 (4-C), 54.8 (4-C), 54.1 (9-C), 54.0 (9-C), 51.0 (2-C), 50.5 (2-C), 40.6 (5-C), 39.4 (5-C), 37.3 (6-C), 36.7 (6-C), 34.0 (8-C), 33.6 (8-C), 25.7 (C_q*C*H$_3$), 25.5 (C_q*C*H$_3$), 23.7 (NH(CO)*C*H$_3$), 23.6 (NH(CO)*C*H$_3$) [31 of 36 expected peaks observed]. **IR** ν_{max}(film)/cm^{-1} 3342 (OH), 2952, 2887, 1683 (CO), 1499, 1422, 1346, 731. **HRMS** (ESI): $C_{20}H_{27}N_2O_5$ [M+H]$^+$; calculated 375.1915, found 375.1916.

(*1R*,5R*,7R*,9S*,10S)-9-(Dimethylamino)-10-methyl-11-oxa-3-azatricyclo[5.3.1.0^{1,5}]undecan-10-ol F13**

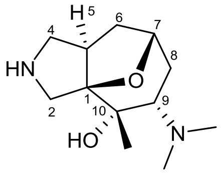

F13

Hydrogenation was carried out following general procedure **B**, using compound **S38** (36 mg, 100 μmol) and Pd/C (5 mg, 15% w/w) in MeOH (10 mL) over 15 h. Filtration followed by concentration gave the *title compound* **F13** (21 mg, 92 μmol, 92%) as a colourless oil. **^{1}H NMR** (400 MHz, MeOD-d$_4$, OH and NH not observed): 4.38 (1H, ddd, *J* 8.2, 5.4, 3.1, 7-H), 3.23-3.07 (1H, m, 5-H), 2.94 (1H, d, *J* 12.4, 2-H_A), 2.90-2.80 (2H, m, 4-H), 2.77 (1H, d, *J* 12.4, 2-H_B), 2.53 (1H, dd, *J* 9.0, 7.4, 9-H), 2.40 (6H, s, N(CH$_3$)$_2$), 2.19 (1H, dd, *J* 9.4, 6.2, 6-H_A), 2.16-2.11 (1H, m, 8-H_B), 1.63-1.51 (2H, m, 6-H_B and 8-H_A),

1.32 (3H, s, C_qCH_3). **^{13}C NMR** (100 MHz, MeOD-d_4): δ 97.7 (1-C), 76.3 (7-C), 73.6 (10-C), 66.3 (9-C), 54.6 (4-C), 53.1 (2-C), 45.2 (N$C$$H_3$)$_2$), 42.7 (6-C), 42.1 (5-C), 26.2 (8-C), 25.4 ($C_q$$C$$H_3$). **IR** ν_{max}(film)/cm^{-1} 3286 (br. OH, NH), 2954, 2871, 2820, 2778, 1647, 1460. **HRMS** (ESI): $C_{12}H_{22}N_2NaO_2$ [M+Na]$^+$; calculated 249.1573, found 249.1573.

***N*-[(*1R**,*5R**,*7R**,*9S**,*10S**)-10-Hydroxy-10-methyl-11-oxa-3-azatricyclo[5.3.1.0^{1,5}]undecan-9-yl]formamide F14**

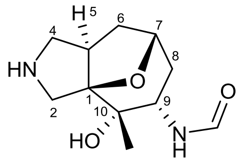

F14

Hydrogenation was carried out following general procedure **B**, using compound **S39** (24 mg, 66 μmol) and Pd/C (5 mg, 20 % w/w) in MeOH (10 mL) over 15 h. Filtration followed by concentration gave the *title compound* **F14** (14 mg, 62 μmol, 94%) as a colourless oil. **^{1}H NMR** (400 MHz, MeOD-d_4, OH and 2 × NH not observed): δ 8.11 (1H, d, *J* 0.9, NHC*H*O), 4.46 (1H, app. t, *J* 5.4, 7-H), 3.89 (1H, dd, *J* 6.4, 1.7, 9-H), 3.21-3.14 (1H, m, 5-H), 3.12 (1H, d, *J* 11.2, 4-H_A), 3.07 (1H, d, *J* 12.3, 2-H_A), 2.97 (1H, d, *J* 12.3, 2-H_B), 2.78 (1H, dd, *J* 11.2, 3.5, 4-H_B), 2.30 (1H, dd, *J* 12.7, 8.7, 6-H_A), 2.17 (1H, app. dt, *J* 14.7, 5.6, 8-H_B), 1.86 (1H, ddd, *J* 14.7, 2.4, 1.9, 8-H_A), 1.83-1.75 (1H, m, 6-H_B), 1.41 (3H, s, C_qCH_3). **^{13}C NMR** (100 MHz, MeOD-d_4): δ 164.5 (NH(CO)H), 97.2 (1-C), 77.9 (10-C), 70.5 (7-C), 56.0 (4-C), 53.5 (9-C), 52.1 (2-C), 42.6 (5-C), 38.9 (6-C), 34.8 (8-C), 25.3 (CH_3). **IR** ν_{max}(film)/cm^{-1} 3294 (br. OH, NH), 2930, 2873, 1661, 1511, 1383, 1167, 1146. **HRMS** (ESI): $C_{11}H_{19}N_2O$ [M+H]$^+$; calculated 227.1390, found 227.1287.

***N*-[(*1R**,*5R**,*7R**,*9S**,*10S**)-10-hydroxy-10-methyl-11-oxa-3-azatricyclo[5.3.1.0^{1,5}]undecan-9-yl]acetamide F15**

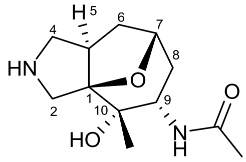

F15

Hydrogenation was carried out following general procedure **B**, using compound **S40** (59 mg, 0.16 mmol) and Pd/C (5 mg, 10% w/w) in MeOH (10 mL) over 15 h. Filtration followed by concentration gave the *title compound* **F15** (38 mg, 0.16 mol, 99%) as a colourless oil. **^{1}H NMR** (400 MHz, MeOD-d_4, OH and 2 × NH not observed): δ 4.49 (1H, t, *J* 5.5, 7-H), 3.80 (1H, dd, *J* 6.5, 2.4, 9-H), 3.52-3.41 (3H, m, includes: 1H, 4-H_A; 1H, 5-H; and at δ: 3.44: 1H, d, *J* 12.3, 2-H_A), 3.37 (1H, d, *J* 12.3, 2-H_B), 3.19-3.10 (1H, m, 4-H_B), 2.42 (1H, dd, *J* 12.8, 8.2, 6-H_A), 2.14 (1H, dt, *J* 14.7, 5.7, 8-H_B), 2.02 (3H, s, NH(CO)C*H*$_3$), 1.92-1.87 (1H, m, 6-H_B), 1.87-1.80 (1H, m, 8-H_A), 1.38 (3H, s, C_qCH_3). **^{13}C NMR** (100 MHz, MeOD-d_4): δ 174.6 (NH(*C*O)CH_3), 95.0 (1-C), 77.9 (10-C), 70.4 (7-C), 54.5 (9-C), 54.3 (4-C), 50.7 (2-C), 40.5 (5-C), 38.9 (6-C), 34.1 (8-C), 25.5 (C_q*C*H_3), 22.8 (NH(CO)*C*H_3). **IR** ν_{max}(film)/cm^{-1} 3333 (br. OH, NH), 2967, 2773, 1642, 1526, 1436, 1376, 1098. **HRMS** (ESI): $C_{12}H_{21}N_2O_3$ [M+H]$^+$; calculated 241.1547, found 241.1549.

5.4.3.7 Preparation of fragments derived from scaffold 14

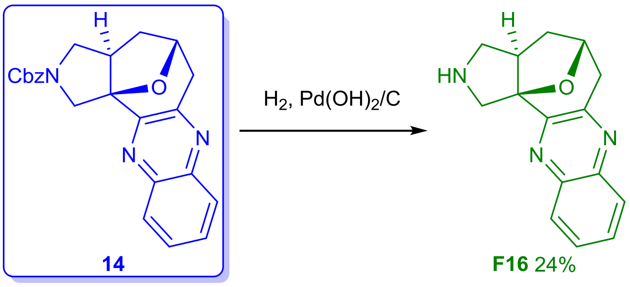

(*1R,*13R**,*15R**)-19-oxa-3,10,17-triazapentacyclo[11.5.1.0^{1,15}.0^{2,11}.0^{4,9}]nonadeca-2(11),3,5,7,9-pentaene F16**

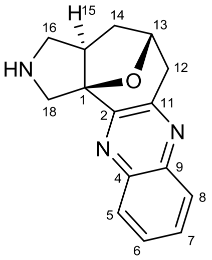

F16

Hydrogenation was carried out following general procedure **B**, using compound **14** (281 mg, 0.73 mmol) and Pd(OH)$_2$/C (60 mg, 10% w/w) in MeOH (10 mL) over 15 h. Flash chromatography eluting with 93:7:1 CH_2Cl_2–EtOH–NH_3/MeOH, then 50:8:1 CH_2Cl_2–EtOH–NH_3/MeOH, gave the *title compound* **F16** (44 mg, 0.17 mmol, 24%) as a colourless oil.* **R*f*** 0.35 (50:8:1 CH_2Cl_2–EtOH–NH_3/MeOH). **^{1}H NMR** (500 MHz, $CDCl_3$, NH not observed): δ 8.04-7.93 (1H, m, 5-H and 8-H), 7.72-7.66 (1H, m, 6-H and 7-H), 5.05 (1H, app. t, *J* 7.1, 13-H), 4.13 (1H, d, *J* 12.9, 18-H$_B$), 3.69 (1H, ddd, *J* 18.4, 6.8, 1.4, 16-H$_B$), 3.38 (1H, d, *J* 12.9, 18-H$_A$), 3.32 (1H, dd, *J* 11.8, 8.1, 12-H$_B$), 2.97 (1H, d, *J* 18.4, 16-H$_A$), 2.85 (1H, dd, *J* 11.8, 5.1, 12-H$_A$), 2.80-2.73 (1H, m, 15-H), 2.27 (1H, dd, *J* 13.0, 9.0, 14-H$_A$), 2.17-2.10 (1H, m, 14-H$_B$). **^{13}C NMR** (125 MHz, $CDCl_3$): 154.9 (Ar-C$_q$), 151.4 (Ar-C$_q$), 142.0 (Ar-C$_q$), 140.4 (Ar-C$_q$), 129.6 (Ar-C), 129.4 (Ar-C), 129.3 (Ar-C), 128.5 (Ar-C), 95.0 (1-C), 76.9 (13-C), 55.9 (18-C), 54.5 (15-C), 52.0 (16-C), 39.6 (12-C), 39.5 (14-C). **IR** ν_{max}(film)/cm^{-1} 2953, 2869, 1489, 1318, 1184, 1136, 763. **HRMS** (ESI): $C_{15}H_{16}N_3O$ [M+H]$^+$; calculated 254.1288, found 254.1276.

---

* N.b. another product was observed through analysis of the crude residue by NMR spectroscopy at 300 MHz and by LRMS. This was postulated to be an alcohol resulting from hydrogenolytic cleavage of the ether (HPLC-MS: $C_{15}H_{18}N_3O$; found 255.9 [M+H]$^+$). However, we were unable to cleanly isolate this material.

5.4.3.8 Preparation of fragments derived from scaffold 15

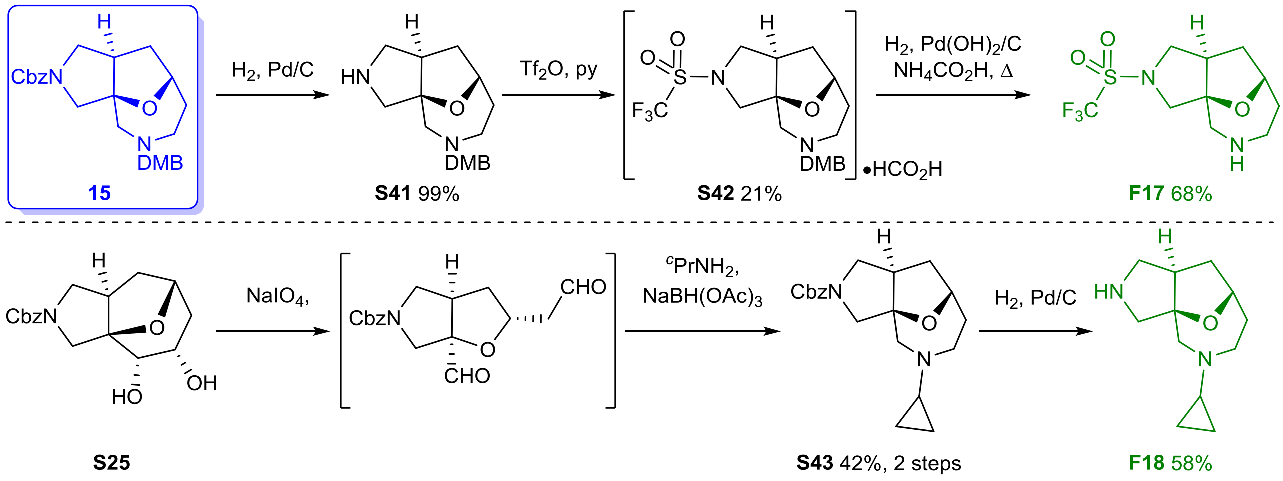

(*1R*,*5R*,*7R*)-10-[(2,4-dimethoxyphenyl)methyl]-12-oxa-3,10-diazatricyclo[5.4.1.$0^{1,5}$]dodecane S41

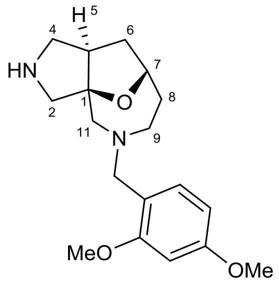

S41

Hydrogenation was carried out following general procedure **B**, using compound **15** (396 mg, 0.88 mmol) and Pd/C (40 mg, 10% w/w) in MeOH (25 mL) over 15 h. Filtration followed by concentration gave the *title compound* **S41** (278 mg, 0.87 mmol, 99%) as a colourless amorphous solid. **^{1}H NMR** (500 MHz, $CDCl_3$): δ 7.32 (1H, d, *J* 8.2, DMB 6-H), 6.48 (1H, dd, *J* 8.2, 2.4, DMB 5-H), 6.45 (1H, d, *J* 2.4, DMB 3-H), 4.50 (1H, app. t, *J* 7.8, 7-H), 3.81 (3H, s, OCH_3), 3.79 (3H, s, OCH_3), 3.64 (2H, s, NCH_2Ar), 3.05 (1H, d, *J* 12.3, 2-H_A), 2.94 (1H, dd, *J* 11.7, 7.3, 4-H_B), 2.79 (1H, d, *J* 11.7, 4-H_A), 2.76-2.67 (2H, m, includes: 1H, 5-H; and at δ 2.69: 1H, d, *J* 12.2, 11-H_A), 2.67-2.56 (3H, m, 9-H and 11-H_B), 2.47 (1H, d, *J* 12.3, 2-H_A), 2.16-2.10 (1H, m, 6-H_A), 2.03-1.95 (1H, m, 8-H_B), 1.82 (1H, app. dt, *J* 12.2, 7.2, 6-H_B), 1.52-1.43 (1H, m, 8-H_A). **^{13}C NMR** (125 MHz, $CDCl_3$, DMB 1-C not observed): δ 159.9 (DMB 2-C or 4-C), 158.8 (DMB 2-C or 4-C), 130.7 (DMB 6-C), 104.2 (DMB 5-C), 98.6 (DMB 3-C), 96.2 (1-C), 77.6 (7-C), 62.8 (11-C), 57.8 (2-C), 56.4 (N*C*H_2Ar), 55.5 (3 peaks, 2 × OCH_3 and 4-C), 51.4 (9-C), 47.7 (5-C), 43.0 (6-C), 37.1 (8-C). **IR** ν_{max}(film)/cm^{-1} 2933, 2833, 1612, 1588, 1505, 1456, 1290, 1207. **HRMS** (ESI): $C_{18}H_{26}N_2O_3$ [M+H]$^+$; calculated 319.2016, found 319.2016.

(*1S*,*5R*,*7R*)-10-[(2,4-dimethoxyphenyl)methyl]-3-trifluoromethanesulfonyl-12-oxa-3,10-diazatricyclo[5.4.1.0^{1,5}]dodecane formate S42

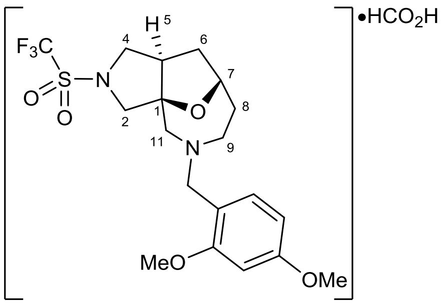

S42

Tf_2O (0.14 mL, 0.83 mmol, 2.00 eq.) was added to a stirred solution of compound **S41** (130 mg, 0.41 mmol, 1.00 eq.) in pyridine (5 mL) at 0 °C. The reaction mixture was warmed to rt then stirred for 2 h. The reaction mixture was concentrated *in vacuo*, then partitioned between EtOAc (25 mL) and sat. aq. $NaHCO_3$ solution (25 mL). The phases were separated and the aqueous phase was extracted with EtOAc (2 × 25 mL). The combined organic phases were dried over $MgSO_4$, filtered and concetrated *in vacuo*. Purification by mass directed liquid chromatography (binary gradient of MeOH and H_2O containing 0.1% HCO_2H) gave the *title compound* **S42** (43 mg, 87 μmol, 21%) as a bright yellow oil. **R*f*** 0.41 (29:1:0.1 CH_2Cl_2–EtOH–NH_3/MeOH). **^{1}H NMR** (500 MHz, MeOD-d$_4$): δ 8.24 (2H, s, *H*CO_2H and HCO_2*H*), 7.36 (1H, d, *J* 6.9, DMB 6-H), 6.62 (1H, d, *J* 1.9, DMB 3-H), 6.58 (1H, d, *J* 6.9, DMB 5-H), 4.72-4.60 (1H, m, 7-H), 4.28 (2H, s, C*H$_2$*Ar), 3.87 (3H, s, OCH_3), 3.84-3.79 (4H, m, includes: 1H, 2-H$_A$; and at δ 3.81: 3H, s, OCH_3), 3.75-3.68 (1H, m, 4-H$_A$), 3.52 (1H, d, *J* 10.5, 4-H$_B$), 3.48-3.37 (3H, m, 9-H and 11-H$_A$), 3.34 (1H, d, *J* 10.5, 2-H$_B$), 3.31-3.21 (1H, m, 11-H$_B$), 3.13-3.02 (1H, m, 5-H), 2.31-2.17 (2H, m, includes: 1H, 8-H$_B$; and at δ 2.27: 1H, dd, *J* 12.4, 9.2, 6-H$_A$), 2.08-1.98 (1H, m, 6-H$_B$), 1.73 (1H, d, *J* 13.5, 8-H$_A$). **^{13}C NMR** (125 MHz, MeOD-d$_4$): δ 166.7 (H*C*O_2H), 164.3 (DMB 2-C or 4-C), 161.1 (DMB 2-C or 4-C), 135.0 (DMB 6-C), 121.5 (q, *J* 322.0, CF_3), 111.5 (DMB 1-C), 106.7 (DMB 5-C), 99.7 (DMB 3-C), 91.1 (1-C), 80.5 (7-C), 60.2 (11-C), 58.6 (2-C), 57.4, (4-C and N*C*H_2Ar), 56.3 (OCH_3), 56.0 (OCH_3), 51.5 (9-C), 50.5 (5-C), 39.3 (6-C), 33.7 (8-C). **IR** ν_{max}(film)/cm^{-1} 1711, 1612, 1586, 1510, 1458, 1384, 1294, 1188. **HRMS** (ESI): $C_{19}H_{26}F_3N_2O_5S$ [M+H]$^+$; calculated 451.1509, found 451.1520.

(*1S*,*5R*,*7R*)-3-Trifluoromethanesulfonyl-12-oxa-3,10-diazatricyclo[5.4.1.0^{1,5}]dodecane F17

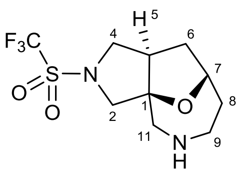

F17

To a stirred solution of compound **S42** (31 mg, 69 μmol, 1.0 eq.) in EtOH (2 mL) was added conc. HCl (12 M, 0.1 mL), 20 wt% $Pd(OH)_2$/C (15 mg, 50% w/w) and NH_4CO_2H (88 mg, 1.4 mmol, 10 eq.). The reaction mixture was heated for 24 h, then filtered through a pad of Celite eluting with EtOH and concentrated *in vacuo*. Purification by SCX SPE following general procedure **A**, eluting with MeOH, then sat. NH_3/MeOH, gave the *title compound* **F17** (14 mg, 47 μmol, 68%) as a colourless oil. **^{1}H NMR** (400 MHz, $CDCl_3$): δ 4.72-4.66 (1H, m, 7-H), 3.83-3.70 (2H, m, includes: 1H, 4-H$_A$; and at δ 3.76: 1H, d, *J* 11.4, 2-H$_A$), 3.41 (1H, dd, *J* 10.5, 6.1, 4-H$_B$), 3.34 (1H, d, *J* 11.4, 2-H$_B$),

3.01-2.88 (4H, m, includes: 2H, 9-H; and at δ 2.94: 2H, 11-H, s), 2.88-2.78 (1H, m, 5-H), 2.25-2.15 (1H, m, 6-H_A), 2.13-1.96 (3H, m, 6-H_B and 8-H_A and NH), 1.64-1.55 (1H, m, 8-H_B). **^{13}C NMR** (100 MHz, $CDCl_3$): δ 120.4 (q, *J* 323.4, CF_3), 94.7 (1-C), 78.3 (7-C), 57.2 (2-C), 56.4 (11-C), 55.9 (4-C), 47.1 (5-C), 45.7 (9-C), 40.3 (6-C), 38.3 (8-C). **IR** ν_{max}(film)/cm^{-1} 2936, 1385, 1290, 1227, 1186, 1149, 629, 589. **HRMS** (ESI): $C_{10}H_{16}F_3N_2O_3S$ [M+H]$^+$; calculated 301.0828, found 301.0831.

Benzyl (*1S,*5R**,*7R**)-10-cyclopropyl-12-oxa-3,10-diazatricyclo[5.4.1.0^{1,5}]dodecane-3-carboxylate S43**

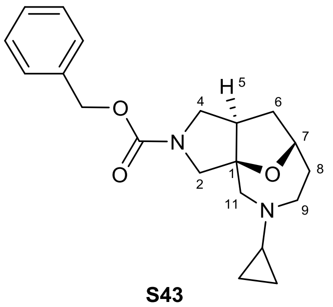

S43

$NaIO_4$ (188 mg, 0.880 mmol, 2.00 eq.) was added to a stirred solution of diol **S25** (140 mg, 0.44 mmol, 1.00 eq.) in 2:1 MeOH–H_2O (7.5 mL) at 0 °C. The reaction mixture was warmed to rt and stirred for 15 h. The reaction mixture was concentrated *in vacuo*, then diluted in CH_2Cl_2 (10 mL) and washed with brine (10 mL). The phases were separated and the aqueous layer was extracted with CH_2Cl_2 (3 × 10 mL). The resulting crude dialdehyde was dissolved in CH_2Cl_2 (5 mL), and cyclopropylamine (29 µL, 0.42 mmol, 1.30 eq.) and $NaBH(OAc)_3$ (176 mg, 0.83 mmol, 2.60 eq.) were added. The reaction mixture was stirred for 15 h then filtered through Celite and concentrated *in vacuo*. The resulting reside was diluted in EtOAc (10 mL) and washed with brine (10 mL). The aqueous phase was extracted with EtOAc (2 × 10 mL). The combined organic phase was dried over $MgSO_4$, filtered and concentrated *in vacuo*. Flash chromatography eluting with 2:3 pentane–EtOAc gave the *title compound* **S43** (46 mg, 0.13 mmol, 42%, 2 steps) as a colourless oil. **R_f** 0.55 (1:1 petrol–EtOAc). **^{1}H NMR** (500 MHz, $CDCl_3$, mixture of rotamers): δ 7.38-7.27 (5H, m, Cbz Ar-H), 5.11 (2H, s, OC*H*$_2$Ph), 4.58 (1H, app. td, *J* 8.0, 3.7, 7-H), 3.76 (1H, dd, *J* 11.0, 9.1, 4-H_B), 3.68 (1H, d, *J* 12.2, 2-H_A), 3.34 (0.5H, d, *J* 12.2, 2-H_B), 3.28 (0.5H, d, *J* 12.2, 2-H_B), 3.26-3.13 (1H, m, 4-H_A), 2.97 (1H, dd, *J* 12.9, 6.5, 9-H_A), 2.86 (1H, d, *J* 12.2, 11-H_A), 2.79-2.64 (2H, m, 9-H_B and 11-H_B), 2.55 (1H, app. qd, *J* 8.1, 3.3, 5-H), 2.09-2.00 (2H, m, 6-H), 2.00-1.92 (1H, m, 8-H_B), 1.92-1.85 (1H, m, cyclopropyl C*H*), 1.49 (1H, ddd, *J* 13.5, 11.2, 6.7, 8-H_A), 0.49-0.38 (3H, m, cyclopropyl (C*H*$_A$$H_B$)$_A$ and (CH_2)$_B$), 0.30-0.24 (1H, m, cyclopropyl (CH_A*H*$_B$)$_A$). **^{13}C NMR** (125 MHz, $CDCl_3$, 329 K, mixture of two rotamers, 1 Ar-C not observed): δ 154.8 (N(CO)O), 137.3 (Ar-C_q), 128.6 (Ar-C), 128.0 (Ar-C), 93.4 (1-C), 77.8 (7-C), 66.9 (O*C*H$_2$Ph), 64.7 (11-C), 55.2 (2-C), 53.6 (4-C or 9-C), 53.2 (4-C or 9-C), 46.0 (5-C), 41.0 (6-C), 40.1 (cyclopropyl *C*H), 37.1 (8-C), 8.2 (cyclopropyl (*C*H$_2$)$_A$), 7.4 (cyclopropyl (*C*H$_2$)$_B$) [17 of 34 expected peaks observed]. **IR** ν_{max}(film)/cm^{-1} 2933, 1701 (CO), 1450, 1415, 1364, 1350, 1121, 1089. **HRMS** (ESI): $C_{20}H_{27}N_2O_3$ [M+H]$^+$; calculated 343.2016, found 343.2021.

(*1R*,5R*,7R**)-10-cyclopropyl-12-oxa-3,10-diazatricyclo[5.4.1.0^{1,5}]dodecane F18

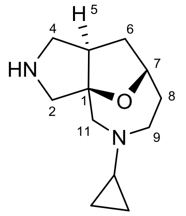

F18

Hydrogenation was carried out following general procedure **B**, using compound **S43** (34 mg, 100 μmol) and Pd/C (5 mg, 10% w/w) in MeOH (10 mL) over 15 h. Flash chromatography eluting with 98:2:0.1 CH_2Cl_2–EtOH–NH_3/MeOH gave the *title compound* **F18** (12 mg, 58 μmol, 58%) as a colourless oil. **R*f*** 0.42 (98:2:0.1 CH_2Cl_2–EtOH–NH_3/MeOH). **^{1}H NMR** (400 MHz, MeOD-d$_4$, NH not observed): 4.50 (1H, app. t, *J* 7.6, 7-H), 2.99 (1H, d, *J* 13.2, 11-H$_A$), 3.96-2.86 (2H, m, 4-H$_A$ and 9-H$_A$), 2.86-2.72 (4H, m, 2-H, 4-H$_B$, 9-H$_B$), 2.67 (1H, app. dd, *J* 14.3, 7.2, 5-H), 2.53 (1H, app. t, *J* 14.0, 11-H$_B$), 2.14-1.94 (3H, m, 6-H$_A$, 8-H$_A$ and cyclopropyl C*H*), 1.94-1.84 (1H, m, 6-H$_B$), 1.53-1.42 (1H, m, 8-H$_B$), 0.56-0.43 (3H, m, cyclopropyl (C*H*$_A$H$_B$)$_A$ and (CH$_2$)$_B$), 0.43-0.33 (1H, m, cyclopropyl (CH$_A$*H*$_B$)$_A$). **^{13}C NMR** (100 MHz, MeOD-d$_4$, 1-C not observed): δ 79.5 (7-C), 63.7 (11-C), 58.0 (2-C), 55.8 (4-C and 9-C), 52.7 (5-C), 42.6 (6-C), 40.4 (cyclopropyl CH), 37.6 (8-C), 8.0 (cyclopropyl CH$_2$), 7.5 (cyclopropyl CH$_2$). **IR** ν_{max}(film)/cm^{-1} 2925, 2853, 1650, 1450, 1365, 1035, 1015, 827. **HRMS** (ESI): $C_{12}H_{21}N_2O$ [M+H]$^+$; calculated 209.1648, found 209.1644.

5.4.3.9 Preparation of fragments derived from scaffold 16

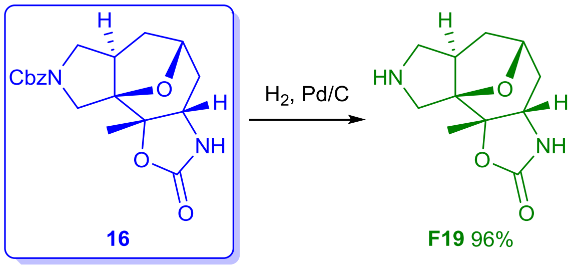

(*1R*,2S*,8R*,10R**)-2-Methyl-3,14-dioxa-5,12-diazatetracyclo[6.5.1.0^{1,10}.0^{2,6}]tetradecan-4-one F19

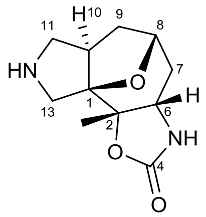

F19

Hydrogenation was carried out following general procedure **B**, using compound **16** (20 mg, 56 μmol) and Pd/C (5 mg, 25% w/w) in MeOH (10 mL) over 15 h. Filtration followed by concentration gave the *title compound* **F19** (12 mg, 54 μmol, 96%) as a colourless oil. **^{1}H NMR** (400 MHz, MeOD-d$_4$, 2 × NH not observed): δ 4.60-4.52 (1H, app. t, *J* 6.5, 8-H), 3.86 (1H, d, *J* 6.6, 6-H), 3.16-3.08 (2H, m, 11-H$_A$ and 13-H$_A$), 2.90 (1H, d, *J* 12.5, 13-H$_B$), 2.88-2.79 (2H, m, 11-H$_B$ and 10-H), 2.33 (1H, dd, *J* 12.5, 8.7, 9-H$_A$), 2.17 (1H, dt, *J* 15.1, 6.2, 7-H$_B$), 1.96-1.88 (1H, m, 9-H$_B$), 1.79 (1H, d, *J* 15.1, 7-H$_A$), 1.58 (3H, s, CH$_3$). **^{13}C NMR** (100 MHz, MeOD-d$_4$): δ 161.4 (4-C), 95.7 (1-C), 81.5 (2-C), 77.2 (8-C), 57.4 (6-C), 56.1 (11-C), 52.5 (13-C), 43.2 (10-C), 39.7 (9-C), 33.2 (7-C), 21.9 (CH$_3$).

IR ν_{max}(film)/cm^{-1} 3260 (NH), 2927, 2874, 1752 (CO), 1384, 1290, 1111, 961. **HRMS** (ESI): $C_{11}H_{17}N_2O_3$ [M+H]$^+$; calculated 225.1234, found 225.1232.

5.4.3.10 Preparation of fragments derived from scaffold 17

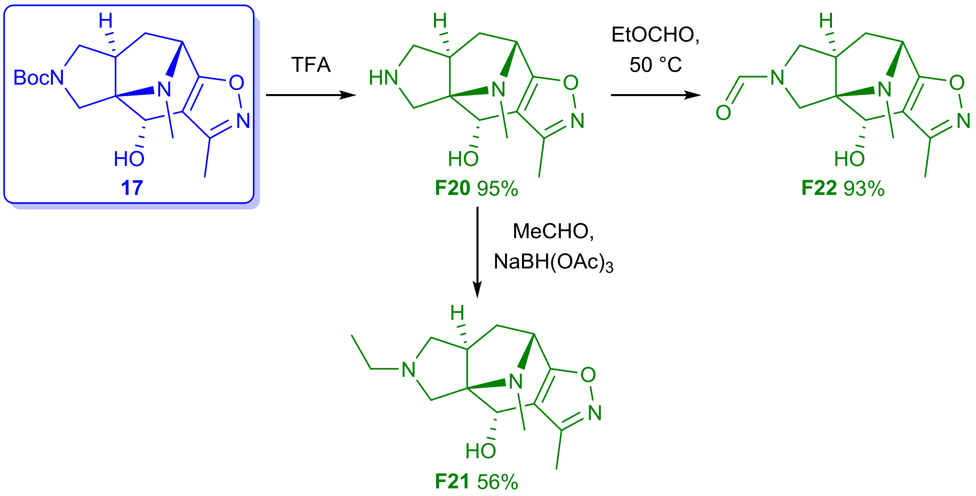

(1*R,2*S**,8*R**,10*R**)-4,14-Dimethyl-6-oxa-5,12,14-triazatetracyclo[6.5.1.0^{1,10}.0^{3,7}]tetradeca-3(7),4-dien-2-ol F20**

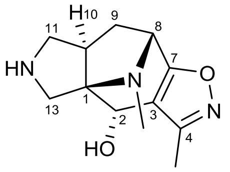

F20

Deprotection of Boc-protected compound **17** (431 mg, 1.28 mmol) was carried out by following general procedure **I**. Purification by SCX SPE, following eluting with MeOH, then sat. NH_3/MeOH, gave the *title compound* **F20** (288 mg, 1.22 mmol, 95%) as a yellow oil. **^{1}H NMR** (400 MHz, MeOD-d$_4$): δ 4.95 (1H, s, 2-H), 4.14 (1H, d, *J* 5.4, 8-H), 2.97 (1H, dd, *J* 11.1, 8.2, 11-H$_A$), 2.95 (1H, d, *J* 12.4, 13-H$_A$), 2.93-2.79 (2H, m, 10-H and 11-H$_B$), 2.84 (1H, d, *J* 12.4, 13-H$_B$), 2.36 (3H, s, C$_q$CH$_3$ or NCH$_3$), 2.33 (3H, s, C$_q$CH$_3$ or NCH$_3$), 2.16 (1H, dd, *J* 11.9, 8.6, 9-H$_A$), 1.90 (1H, app. dt, *J* 11.9, 5.8, 9-H$_B$). **^{13}C NMR** (100 MHz, MeOD-d$_4$): δ 171.1, 158.3, 112.3, 77.3, 62.4, 61.2, 53.4, 48.3, 41.9, 38.2, 30.4, 9.0. **IR** ν_{max}(film)/cm^{-1} 3316, 1633, 1451. **HRMS** (ESI): $C_{12}H_{18}N_3O_2$ [M+H]$^+$; calculated 236.1394, found 236.1392.

(1*R,2*S**,8*R**,10*R**)-12-Ethyl-4,14-dimethyl-6-oxa-5,12,14-triazatetracyclo[6.5.1.0^{1,10}.0^{3,7}]tetradeca-3(7),4-dien-12-ol F21**

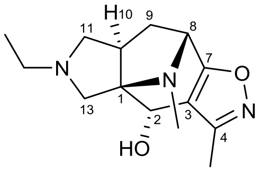

F21

Reductive amination was carried out by following general procedure **C**, using compound **F20** (89 mg, 0.38 mmol) and acetaldehyde (5.0 M in THF, 2.0 eq.). The reaction was quenched with H_2O (5 mL) and the phases separated. The aqueous phase was extracted with CH_2Cl_2 (2 x 10 mL) and the combined organic phases were dried, filtered and concentrated *in vacuo* to give a crude oil. The crude material

was purified by SCX SPE eluting with MeOH, then sat. NH_3/MeOH, to give the *title compound* **F21** (56 mg, 0.21 mmol, 56%) as a yellow oil. **^{1}H NMR** (500 MHz, MeOD-d_4, 333 K): δ 4.90 (1H, s, 2-H), 4.09 (1H, d, *J* 5.4, 8-H), 3.53 (1H, d, *J* 11.9, 13-H_A), 3.35 (1H, dd, *J* 11.6, 3.7, 11-H_A), 3.25 (1H, dd, *J* 11.6, 9.6, 11-H_B), 3.22-3.09 (4H, m, 10-H, 13-H_B and NC*H_2*CH_3), 2.28 (3H, s, NCH_3), 2.22 (3H, s, C_qCH_3), 2.09 (1H, dd, *J* 12.1, 9.1, 9-H_A), 1.96 (1H, app. dt, 12.1, 6.0, 9-H_B), 1.26 (3H, t, *J* 7.3, NCH_2C*H_3*). **^{13}C NMR** (125 MHz, MeOD-d_4, 333 K): δ 171.4, 159.8, 112.7, 76.0, 62.9, 62.0, 60.7, 55.6, 51.0, 40.4, 39.0, 31.5, 10.8, 10.2. **IR** ν_{max}(film)/cm^{-1} 3373, 2932, 1681, 1453, 1404, 1365, 1166, 1124. **HRMS** (ESI): $C_{14}H_{22}N_3O_2$ [M+H]$^+$; calculated 264.1712, found 264.1718.

(1*R,2*S**,8*R**,10*R**)-2-Hydroxy-4,14-dimethyl-6-oxa-5,12,14-triazatetracyclo[6.5.1.0^{1,10}.0^{3,7}]tetradeca-3(7),4-diene-12-carbaldehyde F22**

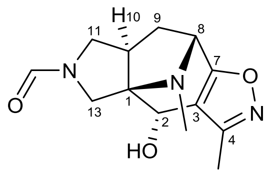

F22

To a solution of compound **F20** (95 mg, 0.40 mmol) in MeOH (1.0 mL) was added ethyl formate (1.0 mL) and the resulting solution was heated to 50 °C for 24 h. The solution was concentrated *in vacuo* to give the *title compound* **F22** (99 mg, 0.38 mmol, 93%) as a yellow oil. **^{1}H NMR** (500 MHz, MeOD-d_4, 333 K, two stable conformations observed at the pyrrolidine ring [50:50 mixture]): δ 8.01 (0.5H, s, NCHO), 7.99 (0.5H, s, NCHO), 4.85 (0.5H, s, 2-H), 4.83 (0.5H, s, 2-H), 4.06 (0.5H, d, *J* 5.8, 8-H), 4.04 (0.5H, d, *J* 5.7, 8-H), 3.77 (0.5H, dd, *J* 11.0, 9.4, 11-$H_{B\text{-}conf1}$), 3.63 (0.5H, d, *J* 11.9, 13-$H_{A\text{-}conf1}$), 3.62 (0.5H, d, *J* 12.8, 13-$H_{A\text{-}conf2}$), 3.55 (0.5H, d, *J* 11.9, 13-$H_{B\text{-}conf1}$), 3.49 (0.5H, dd, *J* 12.2, 9.6, 11-$H_{B\text{-}conf2}$), 3.42 (0.5H, dd, *J* 12.2, 5.2, 11-$H_{A\text{-}conf2}$), 3.40 (0.5H, d, *J* 12.8, 13-$H_{B\text{-}conf2}$), 3.35 (0.5H, dd, *J* 11.0, 6.3, 11-$H_{A\text{-}conf1}$), 3.03-2.91 (1H, m, 10-H), 2.27 (1.5H, s, NCH_3), 2.26 (1.5H, s, NCH_3), 2.21 (1.5H, s, C_qCH_3), 2.21 (1.5H, s, C_qCH_3), 2.06 (0.5H, dd, *J* 12.1, 8.9, 9-H_A), 2.01 (0.5H, dd, *J* 12.2, 8.8, 9-H_A), 1.88 (0.5H, dd, *J* 12.2, 5.7, 9-H_B), 1.84 (0.5H, dd, *J* 12.1, 5.8, 9-H_B). **^{13}C NMR** (125 MHz, MeOD-d_4, 333 K): δ 172.3, 163.2, 163.1, 159.8, 112.7, 76.4, 76.1, 64.3, 64.0, 62.7, 62.5, 54.7, 51.3, 49.6, 46.8, 40.9, 40.8, 39.8, 38.7, 31.8, 31.7, 10.2 [22 of 26 expected peaks observed]. **IR** ν_{max}(film)/cm^{-1} 3355, 1641, 1450. **HRMS** (ESI): $C_{13}H_{18}N_3O_3$ [M+H]$^+$; calculated 264.1343, found 264.1343.

5.4.3.11 Preparation of fragment derived from scaffold 18

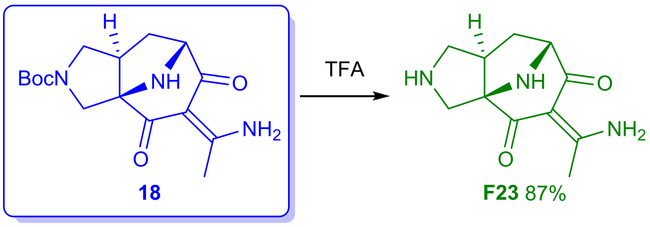

(1*R,5*R**,7*R**,9E)-9-(1-Aminoethylidene)-3,11-diazatricyclo[5.3.1.0^{1,5}]undecane-8,10-dione F23**

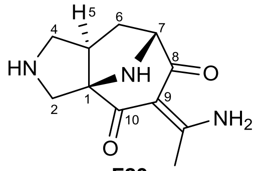
F23

Deprotection of Boc-protected compound **18** (100 mg, 0.31 mmol) was carried out by following general procedure **I**. Purification by SCX SPE eluting with MeOH then sat. NH_3/MeOH gave the *title compound* **F23** (60 mg, 0.27 mmol, 87%) as a yellow oil. **^{1}H NMR** (400 MHz, MeOD-d$_4$): δ 3.88 (1H, d, *J* 6.6, 7-H), 3.61 (1H, d, *J* 12.5, 2-H$_A$), 3.37 (1H, s, NH), 3.16 (1H, dd, *J* 11.9, 8.4, 4-H$_B$), 2.80 (1H, d, *J* 12.5, 2-H$_B$), 2.78 (1H, dd, *J* 11.9, 6.3, 4-H$_A$), 2.58 (1H, app. tt, *J* 8.5, 5.9, 5-H), 2.50 (3H, s, CH_3), 2.14 (1H, dd, *J* 12.8, 8.8, 6-H$_A$), 1.92 (1H, ddd, *J* 12.8, 6.6, 6.4, 6-H$_B$). **^{13}C NMR** (100 MHz, MeOD-d$_4$): δ 200.5, 197.3, 175.7, 103.2, 79.7, 68.7, 53.1, 50.1, 49.1, 48.5, 34.6. **HRMS** (ESI): $C_{11}H_{16}N_3O_2$ [M+H]$^+$; calculated 222.1237, found 222.1235.

5.4.3.12 Preparation of a fragment derived from scaffold 19

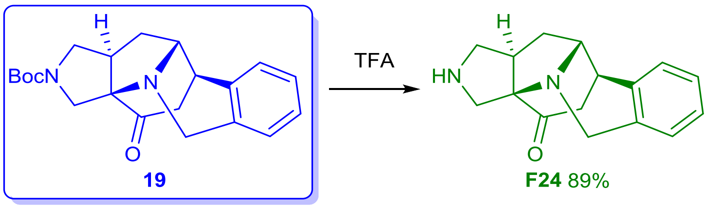

(1*R,10*R**,13*R**,17*R**)-2,15-diazapentacyclo[8.8.0.0^{2,13}.0^{4,9}.0^{13,17}]octadeca-4(9),5,7-triene-12-one F24**

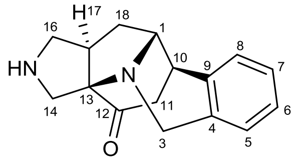
F24

Deprotection of Boc-protected compound **19** (12 mg, 34 μmol) was carried out by following general procedure **I**. Purification by SCX SPE eluting with MeOH then sat. NH_3/MeOH gave the *title compound* **F24** (8 mg, 31 μmol, 93%) as a yellow oil. **^{1}H NMR** (500 MHz, $CDCl_3$): δ 7.18 (1H, app. td, *J* 7.4, 1.3, 6-H or 7-H), 7.14 (1H, app. t, *J* 7.5, 6-H or 7-H), 7.01-6.96 (2H, m, 5-H and 8-H), 4.34 (1H, d, *J* 18.4, 3-H$_A$), 3.98 (1H, d, *J* 18.4, 3-H$_B$), 3.75 (1H, d, *J* 6.3, 1-H), 3.38-3.25 (3H, m, 10-H, 14-H$_A$ and

16-H_A), 2.98-2.90 (3H, m, 11-H_B, 14-H_B and 16-H_B), 2.72-2.59 (1H, m, 17-H), 2.34 (1H, dd, *J* 13.1, 9.2, 18-H_A), 2.26 (1H, d, *J* 15.0, 11-H_A), 2.17 (1H, ddd, *J* 13.1, 6.3, 4.3, 18-H_B). **^{13}C NMR** (125 MHz, $CDCl_3$): δ 209.1, 139.5, 132.0, 127.7, 127.2, 126.7, 125.8, 83.9, 60.0, 55.0, 48.7, 48.5, 46.6, 45.0, 44.5, 33.8. **HRMS** (ESI): $C_{16}H_{19}N_2O$ $[M+H]^+$; calculated 255.1492, found 255.1484.

5.4.3.13 Preparation of fragments derived from scaffold 20

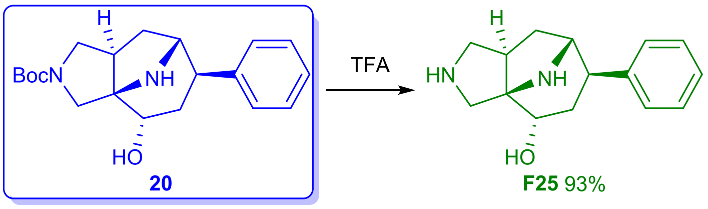

(1*R**,5*R**,7*R**,8*R**,10*S**)-8-Phenyl-3,11-diazatricyclo[5.3.1.0^{1,5}]undecan-10-ol F25

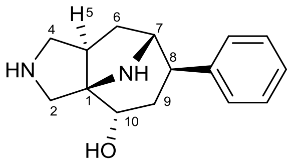

F25

Deprotection of Boc-protected compound **20** (40 mg, 0.12 mmol) was carried out by following general procedure **I**. Purification by SCX SPE eluting with MeOH then sat. NH_3/MeOH gave the *title compound* **F25** (26 mg, 0.11 mmol, 89%) as a brown oil. **^{1}H NMR** (500 MHz, MeOD-d_4, 333 K): δ 7.28-7.13 (4H, m, Ar-H), 7.10-7.04 (1H, m, Ar-H), 3.97 (1H, dd, *J* 10.6, 5.7, 10-H), 3.57 (1H, app. d, *J* 6.6, 7-H), 3.05 (1H, d, *J* 12.1, 2-H_A), 3.04 (1H, d, *J* 11.1, 4-H_A), 2.91 (1H, app. d, *J* 7.3, 8-H), 2.69-2.61 (1H, m, 5-H), 2.58 (1H, d, *J* 12.1, 2-H_B), 2.58 (1H, dd, *J* 11.1, 6.3, 4-H_B), 2.21 (1H, ddt, *J* 14.3, 5.8, 1.2, 9-H_A), 2.02 (1H, dd, *J* 13.1, 8.8, 6-H_A), 1.79 (1H, ddd, *J* 13.1, 6.6, 4.7, 6-H_B), 1.74 (1H, ddd, *J* 14.3, 10.7, 7.4, 9-H_B). **^{13}C NMR** (125 MHz, MeOD-d_4, 333 K): δ 145.5, 129.5, 128.9, 127.0, 78.4, 68.6, 65.0, 55.5, 52.8, 47.7, 42.3, 38.9, 33.9. **IR** ν_{max}(film)/cm^{-1} 3386, 1480, 1447. **HRMS** (ESI): $C_{15}H_{20}N_2O$ $[M+H]^+$; calculated 245.1654, found 245.1650.

5.4.3.14 Preparation of fragments derived from scaffold S16

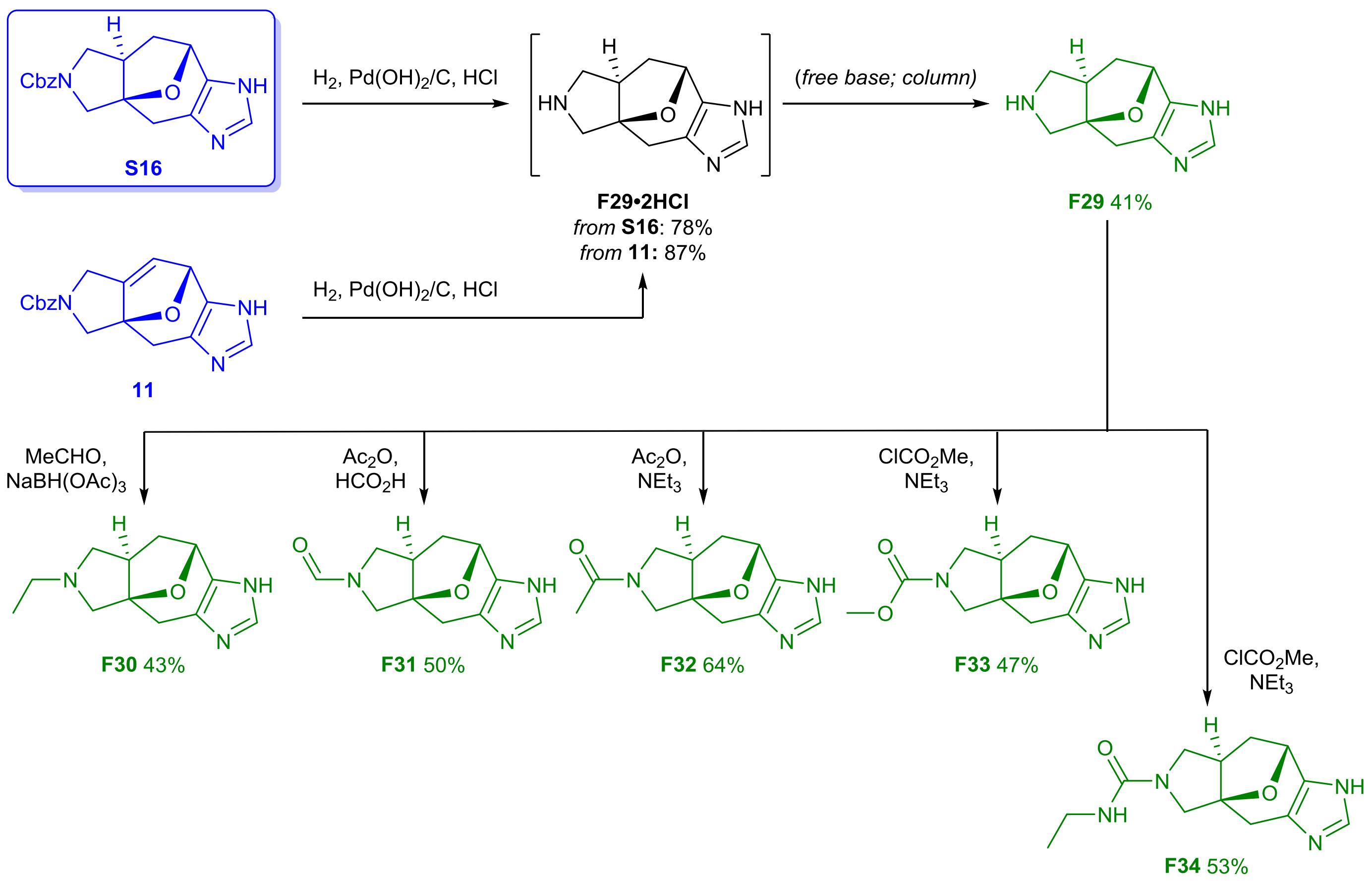

(*1R*,*8R*,*10R*)-14-oxa-4,6,12-triazatetracyclo[6.5.1.0^{1,10}.0^{3,7}]tetradeca-3(7),4-diene F29

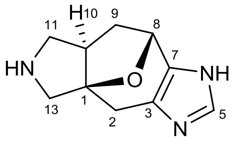
F29

From compound S16: Hydrogenation was carried out following general procedure **B** using compound **S16** (323 mg, 1.00 mmol), $Pd(OH)_2$/C (60 mg, 20% w/w) and conc. HCl (0.1 mL) in MeOH (10 mL) over 15 h. Filtration gave the *title compound* **F29** as the dihydrochloride salt (207 mg, 0.78 mmol, 78%), a colourless foam which was carried on to subsequent steps without further purification {^{1}**H NMR** (500 MHz, MeOD-d$_4$, 333 K, 2 × N^+H_2 not observed): δ 9.06 (1H, s, 5-H), 5.78 (1H, br. s, 8-H), 4.18-4.07 (1H, m, 13-H$_A$), 4.07-3.94 (1H, m, 11-H$_A$), 3.89-3.76 (1H, m, 13-H$_B$), 3.74-3.64 (2H, m, 2-H$_A$ and 11-H$_B$), 3.48-3.31 (2H, m, 2-H$_B$ and 10-H), 3.14-2.98 (1H, m, 9-H$_A$), 2.71-2.58 (1H, m, 9-H$_B$). ^{13}**C NMR** (125 MHz, MeOD-d$_4$, 333 K): δ 134.3 (5-C), 132.5 (7-C), 125.9 (3-C), 90.9 (1-C), 74.5 (8-C), 54.5 (13-C), 53.6 (11-C), 46.6 (10-C), 45.7 (9-C), 30.9 (2-C)}.

From compound 11: Hydrogenation was carried out following general procedure **B** using compound **11** (1.42 g, 4.36 mmol), $Pd(OH)_2$/C (300 mg, 20% w/w) and conc. HCl (0.5 mL) in

MeOH (25 mL) over 15 h. Filtration gave the *title compound* **F29** as the dihydrochloride salt (1.00 g, 3.78 mmol, 87%), a colourless foam.

Procedure to prepare the free amine: A sample of the amine hydrochloride salt **F29•2HCl** (625 mg, 2.37 mmol) was purified by SCX SPE following general procedure **A**, eluting with MeOH, then sat. NH_3/MeOH, followed by flash chromatography eluting with 50:8:1 CH_2Cl_2–EtOH–NH_3/MeOH to give the *title compound* **F29** (184 mg, 0.96 mmol, 41%) as a colourless foam. **^{1}H NMR** (400 MHz, $CDCl_3$, imidazole NH not observed): δ 7.44 (1H, s, 5-H), 5.18 (1H, d, *J* 5.6. 8-H), 3.28 (1H, d, *J* 12.6, 13-H_A), 3.15 (1H, d, *J* 15.0, 2-H_A), 3.02 (1H, dd, *J* 12.3, 7.5, 11-H_B), 2.83 (1H, d, *J* 12.3, 11-H_A), 2.63 (1H, d, *J* 12.6, 13-H_B), 2.58-2.49 (2H, m, includes: 1H, 9-H_A; and at δ 2.52: d, *J* 15.0, 2-H_B), 2.49-2.40 (1H, m, 10-H), 1.94-1.83 (1H, m, 9-H_B). **^{13}C NMR** (100 MHz, $CDCl_3$, 2 × imidazole C_q not observed): δ 133.4 (5-C), 93.3 (1-C), 75.8 (8-C), 56.6 (13-C), 54.1 (11-C), 48.2 (10-C), 45.6 (9-C), 31.5 (2-C). **IR** ν_{max}(film)/cm^{-1} 2959 2918, 2860 1443, 1225, 993, 909, 729. **HRMS** (ESI): $C_{10}H_{14}N_3O$ [M+H]$^+$; calculated 192.1131, found 192.1129.

(*1R,*8R**,*10R**)-12-Ethyl-14-oxa-4,6,12-triazatetracyclo[6.5.1.0^{1,10}.0^{3,7}]tetradeca-3(7),4-diene F30**

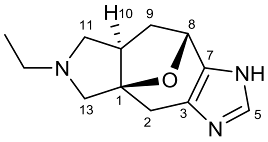

F30

Reductive amination was carried out by following general procedure **C**, using amine hydrochloride **F29•2HCl** (120 mg, 0.45 mmol) and acetaldehyde (5.0 M in THF, 3.0 eq.). Flash chromatography eluting with 93:7:1 CH_2Cl_2–EtOH–NH_3/MeOH gave the *title compound* **F30** (42 mg, 0.19 mmol, 43%, {estimate 90% purity*}) as a yellow oil. **R_f** 0.29 (93:7:1 CH_2Cl_2–EtOH–NH_3/MeOH). **^{1}H NMR** (400 MHz, $CDCl_3$, imidazole NH not observed): δ 7.41 (1H, s, 5-H), 5.22 (1H, d, *J* 5.6, 8-H), 3.23 (1H, d, *J* 10.5, 13-H_A), 3.15 (1H, d, *J* 15.2, 2-H_A), 2.79 (1H, app. d, *J* 7.2, 11-H_B), 2.64-2.36 (6H, m, includes: 1H, 2-H_B; 1H, 9-H_A; 1H, 10-H; 1H, 11-H_A; 2H, NCH_2CH_3), 2.28 (1H, d, *J* 10.5, 13-H_B), 2.09-1.98 (1H, m, 9-H_B), 1.14 (3H, t, *J* 7.3, NCH_2CH_3). **^{13}C NMR** (100 MHz, $CDCl_3$): δ 136.6 (7-C), 133.4 (5-C), 124.7 (3-C), 90.4 (1-C), 75.7 (8-C), 62.8 (13-C), 59.5 (11-C), 49.9 (NCH_2CH_3), 46.2 (10-C), 44.6 (9-C), 32.9 (2-C), 13.3 (NCH_2CH_3). **IR** ν_{max}(film)/cm^{-1} 2971, 2805, 2688, 1590, 1449, 1375, 1343. **HRMS** (ESI): $C_{12}H_{18}N_3O$ [M+H]$^+$; calculated 220.1444, found 220.1440.

---

* As judged by analysis of the product by NMR spectroscopy at 400 MHz.

(*1R,*8R**,*10R**)-14-Oxa-4,6,12-triazatetracyclo[6.5.1.0^{1,10}.0^{3,7}]tetradeca-3(7),4-diene-12-carbaldehyde F31**

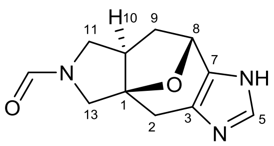

F31 ≡ 23

N-Formylation was carried out by following general procedure **D**, using amine **F29** (80 mg, 0.42 mmol) and Ac_2O (10 eq.). Flash chromatography eluting with 93:7:1 CH_2Cl_2–EtOH–NH_3/MeOH gave the *title compound* **F31** (45 mg, 0.21 mmol, 50%) a colourless oil. **R*f*** 0.27 (93:7:1 CH_2Cl_2–EtOH–NH_3/MeOH). **^{1}H NMR** (400 MHz, MeOD-d$_4$, two stable conformations observed at the pyrrolidine ring [50:50 mixture], imidazole NH not observed): δ 8.16 (0.5H, s, NCHO), 8.14 (0.5H, s, NHCO), 7.49 (1H, s, 5-H), 5.12 (1H, d, *J* 5.6, 8-H), 4.14 (0.5H, d, *J* 13.3, 13-$H_{A\text{-conf1}}$), 4.00 (0.5H, d, *J* 12.5, 13-$H_{A\text{-conf2}}$), 3.84 (0.5H, dd, *J* 11.6, 8.7, 11-$H_{B\text{-conf1}}$), 3.74 (0.5H, dd, *J* 12.7, 2.7, 11-$H_{A\text{-conf2}}$), 3.57-3.44 (1.5H, m, includes: 0.5H, 11-$H_{A\text{-conf1}}$; 0.5H, 11-$H_{B\text{-conf2}}$; and at δ 3.53: 0.5H, d, *J* 12.5, 13-$H_{B\text{-conf2}}$), 3.27 (0.5H, d, *J* 13.3, 13-$H_{B\text{-conf1}}$), 3.17-3.07 (1H, m, 2-H_A, includes at δ 3.13: 0.5H, *J* 15.3; and at δ 3.11: 0.5H, *J* 15.3), 2.73-2.64 (2H, m, includes: 1H, 10-H; and at δ 2.70: 0.5H, *J* 15.3, 2-H_B, and at δ 2.68: 0.5H, *J* 15.3, 2-H_B), 2.57-2.44 (1H, m, 9-H_A), 2.08-1.90 (1H, m, 9-H_B). **^{13}C NMR** (100 MHz, MeOD-d$_4$, 2 × Ar-C_q not observed): δ 163.3 (NCHO), 163.0 (NCHO), 134.9 (5-C), 91.6 (1-C), 91.0 (1-C), 77.0 (8-C), 76.9 (8-C), 55.9 (13-C_{conf2}), 53.8 (11-C_{conf1}), 52.3 (13-C_{conf1}), 50.3 (11-C_{conf2}), 47.2 (10-C), 47.0 (10-C), 46.9 (9-C), 45.9 (9-C), 32.9 (2-C), 32.5 (2-C) [17 of 22 expected peaks observed]. **IR** ν_{max}(film)/cm^{-1} 2963, 2865, 1645 (CO), 1437, 1389, 1231, 992. **HRMS** (ESI): $C_{11}H_{13}N_3NaO_2$ $[M+H]^+$; calculated 242.0900, found 242.0897.

1-[(*1R,*8R**,*10R**)-14-Oxa-4,6,12-triazatetracyclo[6.5.1.0^{1,10}.0^{3,7}]tetradeca-3(7),4-dien-12-yl]ethan-1-one F32**

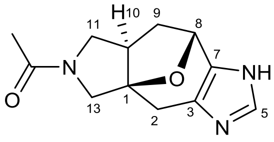

F32 ≡ 28

Ac_2O (20 µL, 0.22 mmol, 0.5 eq.) was added to a stirred solution of amine **F29** (83 mg, 0.43 mmol, 1.00 eq.) and NEt_3 (90 µL, 0.65 mmol, 1.5 eq.) in $CDCl_3$ (10 mL) at 0 °C. The reaction mixture was stirred for 10 minutes, then concentrated *in vacuo*. Flash chromatography eluting with 93:7:1 CH_2Cl_2–EtOH–NH_3/MeOH gave the *title compound* **F32** (64 mg, 0.27 mmol, 64%) as a colourless oil. **R*f*** 0.12 (50:8:1 CH_2Cl_2–EtOH–NH_3/MeOH). **^{1}H NMR** (400 MHz, $CDCl_3$, two stable conformations observed at the pyrrolidine ring [50:50 mixture], imidazole NH not observed): δ 7.43 (0.5H, s, 5-H), 7.42 (0.5H, s, 5-H), 5.25 (0.5H, d, *J* 5.8, 8-H), 5.22 (0.5H, d, *J* 5.8, 8-H), 4.15 (0.5H, d, *J* 13.6, 13-$H_{A\text{-conf1}}$), 3.98 (0.5H, d, *J* 12.2, 13-$H_{A\text{-conf2}}$), 3.89 (0.5H, dd, *J* 10.8, 9.5, 11-$H_{B\text{-conf1}}$), 3.71 (0.5H, dd, *J* 12.5, 9.0, 11-$H_{B\text{-conf2}}$), 3.63 (0.5H, dd, *J* 12.5, 4.5, 11-$H_{A\text{-conf2}}$), 3.49 (0.5H, d, *J* 12.2, 13-$H_{B\text{-conf2}}$), 3.44 (0.5H, d, *J* 13.6, 13-$H_{B\text{-conf1}}$), 3.40 (0.5H, dd, *J* 10.8, 5.4, 11-$H_{A\text{-conf1}}$), 3.23-3.15 (1H, m, 2-H_A, includes at δ 3.20: 0.5H, *J* 15.3; and at δ 3.18: 0.5H, *J* 15.3), 2.80-2.71 (0.5H, m, 10-H_{conf1}), 2.69-2.57 (1.5H, m,

includes: 0.5H, 10-H_{conf2}; at δ 2.62: 0.5H, d, *J* 15.3, 2-H_B, and at δ 2.60: 0.5H, d, *J* 15.3, 2-H_B), 2.51 (0.5H, d, *J* 11.9, 9-H_A), 2.49 (0.5H, d, *J* 11.9, 9-H_A), 2.14-2.02 (4H, m, includes: 1H, 9-H_B; and at δ 2.09: 1.5H, s, $COCH_3$ and at δ 2.05: 1.5H, s, $COCH_3$). **^{13}C NMR** (100 MHz, $CDCl_3$, mixture of two rotamers): δ 169.6 (*C*OCH_3), 169.5 (*C*OCH_3), 137.6 (7-C), 136.4 (7-C), 133.7 (5-C), 123.9 (3-C), 122.8 (3-C), 91.1 (1-C), 89.7 (1-C), 76.5 (8-C), 76.2 (8-C), 56.5 (13-C_{conf2}), 55.2 (13-C_{conf1}), 54.5 (11-C_{conf1}), 52.7 (11-C_{conf2}), 47.2 (10-C), 46.1 (9-C), 45.7 (9-C), 45.5 (10-C), 32.8 (2-C), 22.5 (CO*C*H_3), 22.2 (CO*C*H_3) [22 of 24 expected peaks observed]. **IR** ν_{max}(film)/cm^{-1} 2967, 2872, 1624 (CO), 1448, 1239, 1224, 730. **HRMS** (ESI): $C_{12}H_{16}N_3O_2$ $[M+H]^+$; calculated 234.1237, found 234.1238.

Methyl (*1R,*8R**,*10R**)-14-oxa-4,6,12-triazatetracyclo[6.5.1.0^{1,10}.0^{3,7}]tetradeca-3(7),4-diene-12-carboxylate F33**

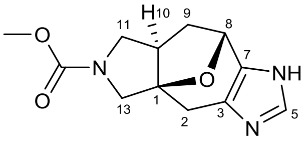

F33

N-Carbamoylation was carried out by following general procedure **F**, using amine **F29** (80 mg, 0.42 mmol). Purification by SCX SPE, eluting with MeOH, then sat. NH_3/MeOH, gave the *title compound* **F33** (49 mg, 0.20 mmol, 47%) as a colourless oil. **^{1}H NMR** (400 MHz, $CDCl_3$): δ 9.74 (1H, br. s, imidazole-NH), 7.43 (1H, s, 5-H), 5.24 (1H, d, *J* 5.9, 8-H), 4.02 (1H, d, *J* 11.9, 13-H_A), 3.77-3.67 (4H, m, includes: 1H, 11-H_A; and at δ 3.70: 3H, s, NCO_2CH_3), 3.54-3.31 (2H, m, 11-H_B and 13-H_B), 3.17 (1H, d, *J* 15.2, 2-H_A), 2.69-2.55 (2H, m, includes: 1H, 10-H; and at δ 2.58: 1H, d, *J* 15.2, 2-H_B), 2.48 (1H, dd, *J* 11.3, 9.0, 9-H_A), 2.12-2.03 (1H, m, 9-H_B). **^{13}C NMR** (100 MHz, $CDCl_3$, 2 × Ar-C_q not observed): δ 155.6 (N*C*O_2CH_3), 133.6 (5-C), 91.2 (1-C), 90.2 (1-C), 76.4 (8-C), 55.3 (13-C), 54.9 (13-C), 53.4 (11-C), 52.7 (NCO_2*C*H_3), 47.0 (10-C), 46.0 (10-C), 45.8 (9-C), 32.7 (2-C) [13 of 24 expected peaks observed]. **IR** ν_{max}(film)/cm^{-1} 2957, 2868, 1688 (CO), 1451, 1393, 1239, 1221, 729. **HRMS** (ESI): $C_{12}H_{16}N_3O_3$ $[M+H]^+$; calculated 250.1186, found 250.1185.

(*1R,*8R**,*10R**)-*N*-Ethyl-14-oxa-4,6,12-triazatetracyclo[6.5.1.0^{1,10}.0^{3,7}]tetradeca-3(7),4-diene-12-carboxamide F34**

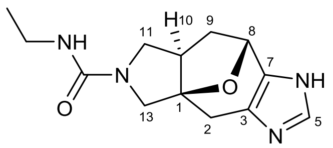

F34

Ethyl isocyanate (32.5 µL, 0.41 mmol, 0.90 eq.) was added to a stirred solution of amine **F29** (87 mg, 0.45 mmol, 1.00 eq.) in $CHCl_3$ (10 mL) at 0 °C. The reaction mixture was warmed to rt, stirred for 10 minutes, then concentrated *in vacuo*. Flash chromatography eluting with 93:7:1 CH_2Cl_2–EtOH–NH_3/MeOH gave the *title compound* **F34** (62 mg, 0.24 mmol, 53%) as a colourless oil. **R_f** 0.18 (50:8:1 CH_2Cl_2–EtOH–NH_3/MeOH). **^{1}H NMR** (400 MHz, $CDCl_3$, imidazole NH not observed): δ 7.44 (1H, s, 5-H), 5.26 (1H, d, *J* 5.9, 8-H), 4.25-4.16 (1H, m, NH), 3.90 (1H, d, *J* 11.6, 13-H_A),

3.72 (1H, app. t, *J* 9.8, 11-H_A), 3.40 (1H, d, *J* 11.6, 13-H_B), 3.36-3.23 (3H, m, 11-H_B and NHCH_2CH$_3$), 3.19 (1H, d, *J* 15.3, 2-H_A), 2.73-2.64 (1H, m, 10-H), 2.61 (1H, d, *J* 15.3, 2-H_B), 2.49 (1H, dd, *J* 11.7, 8.6, 9-H_A), 2.14-2.06 (1H, m, 9-H_B), 1.15 (3H, t, *J* 7.2, NHCH$_2$CH_3). **^{13}C NMR** (100 MHz, $CDCl_3$, 2 × Ar-C_q not observed): δ 157.3 (N(CO)NH), 133.7 (5-C), 90.7 (1-C), 76.5 (8-C), 55.2 (13-C), 53.5 (11-C), 46.6 (10-C), 45.9 (9-C), 35.7 (NH*C*H$_2$), 33.0 (2-C), 15.8 (NHCH$_2$*C*H$_3$). **IR** ν_{max}(film)/cm^{-1} 2969, 2929, 2866, 1619 (CO), 1537, 1396, 1374, 727. **HRMS** (ESI): $C_{13}H_{19}N_4O_2$ [M+H]$^+$; calculated 263.1503, found 263.1504.

5.4.3.15 Preparation of a fragment derived from scaffold S19

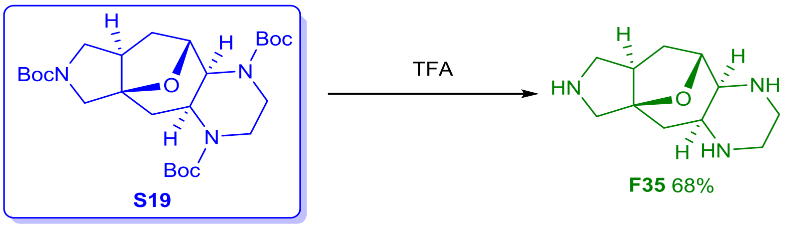

(*1R,*3S**,*8S**,*9R**,*11R**)-15-Oxa-4,7,13-triazatetracyclo[7.5.1.0^{1,11}.0^{3,8}]pentadecane F35**

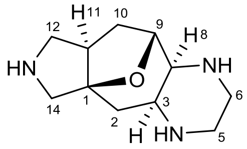

F35

TFA (5 mL) was added to a stirred solution of compound **S19** (36 mg, 71 μmol, 1.00 eq.) in CH_2Cl_2 (10 mL) at 0 °C. The reaction mixture was stirred for 1 h, then concentrated *in vacuo*. The residue was purified by SCX SPE following general procedure **A**, eluting with MeOH, then sat. NH_3/MeOH to give the *title compound* **F35** (10 mg, 48 μmol, 68%) as a colourless oil. **^{1}H NMR** (500 MHz, MeOD-d_4, 3 × NH not observed): δ 4.33 (1H, d, *J* 7.6, 9-H), 3.35-3.28 (2H, m, 5-H_A and 6-H_A), 3.23-3.17 (1H, m, 3-H), 3.01 (1H, dd, *J* 12.9, 1.6, 12-H_A), 2.97-2.78 (4H, m, 5-H_B, 6-H_B, 12-H_B and 14-H_A), 2.74 (1H, dd, *J* 4.0, 1.9, 8-H), 2.71-2.63 (2H, m, 11-H and 14-H_B), 2.47 (1H, app. t, *J* 12.5, 2-H_B), 2.21 (1H, dd, *J* 13.2, 9.1, 10-H_A), 1.97 (1H, ddd, *J* 13.2, 7.6, 4.0, 10-H_B), 1.58 (1H, dd, *J* 13.0, 5.8, 2-H_A). **^{13}C NMR** (100 MHz, MeOD-d_4): δ 92.7 (1-C), 82.0 (9-C), 56.8 (8-C), 55.5 (6-C), 55.0 (5-C), 47.6 (3-C), 46.3 (12-C), 45.6 (11-C), 39.7 (14-C), 36.9 (10-C), 31.3 (2-C). **IR** ν_{max}(film)/cm^{-1} 3387 (br. NH), 3252, 2931, 2856, 1452, 1108, 884, 836. **HRMS** (ESI): $C_{11}H_{20}N_3O$ [M+H]$^+$; calculated 210.1600, found 210.1596.

F35 NOESY correlations:

2-H_A: 2-H_B, 3-H, 11-H

3-H: 2-H_A, 8-H, 10-H_A, 11-H

8-H: 3-H, 9-H, 10-H_A

9-H: 8-H and 10-H_B

10-H_A: 3-H, 8-H, 10-H_B, 11-H

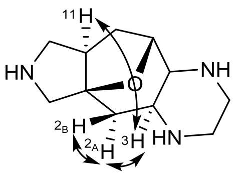

2-H_A

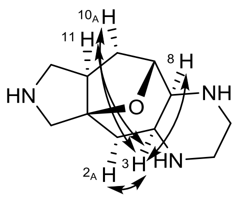

3-H

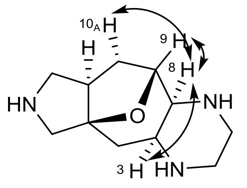

8-H

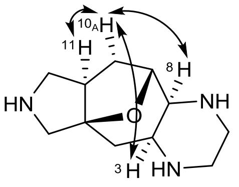

10-H_A

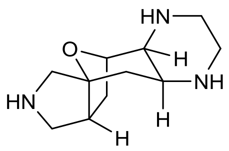

conformation

5.4.3.16 Preparation of a fragment derived from scaffold S20

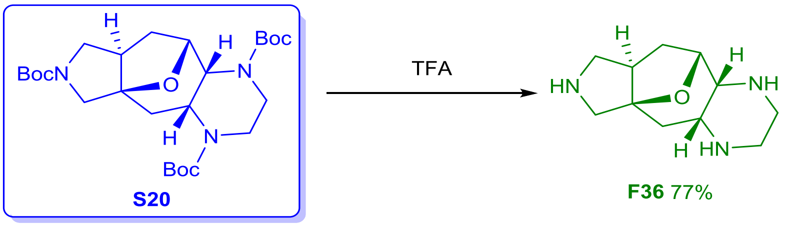

($1R^*$,$3R^*$,$8R^*$,$9R^*$,$11R^*$)-15-Oxa-4,7,13-triazatetracyclo[7.5.1.$0^{1,11}$.$0^{3,8}$]pentadecane F36

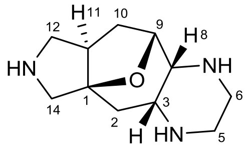

F36

TFA (5 mL) was added to a stirred solution of **S20** (210 mg, 0.41 mmol, 1.00 eq.) in CH_2Cl_2 (5 mL) at 0 °C. The reaction mixture was stirred for 1 h, then concentrated *in vacuo*. The residue was purified by SCX SPE following general procedure **A**, eluting with MeOH, then sat. NH_3/MeOH, followed by flash chromatography eluting with 50:8:1 CH_2Cl_2–EtOH–NH_3/MeOH to give the *title compound* **F36** (66 mg, 0.32 mmol, 77%) as a colourless oil. **R_f** 0.15 (50:8:1 CH_2Cl_2–EtOH–NH_3/MeOH). **^{1}H NMR** (500 MHz, MeOD-d_4, 3 × NH not observed): δ 4.32 (1H, dd, *J* 7.1, 4.5, 9-H), 3.21 (1H, t, *J* 5.2, 3-H), 3.16-3.09 (1H, m, 6-H_A), 3.09-3.06 (2H, m, 11-H and 12-H_A), 3.03 (1H, d, *J* 12.4, 14-H_B), 3.00-2.94 (3H, m, 5-H_A, 8-H and 10-H_A,), 2.79-2.71 (2H, m, 5-H_B and 12-H_B), 2.68 (1H, ddd, *J* 12.6, 3.2, 2.5, 6-H_B), 2.61 (1H, d, *J* 12.4, 14-H_A), 2.23 (1H, dd, *J* 14.1, 5.8, 2-H_B), 1.88-1.81 (1H, m, 10-H_B), 1.62 (1H, dd, *J* 14.1, 1.1, 2-H_A). **^{13}C NMR** (100 MHz, MeOD-d_4): δ 92.5 (1-C), 82.4 (9-C), 56.6 (14-C), 56.1 (12-C), 54.8 (8-C), 53.4 (3-C), 47.8 (5-C), 47.5 (11-C), 42.3 (6-C), 39.3 (10-C), 38.5 (2-C). **IR** ν_{max}(film)/cm^{-1} 3297 (br. NH), 2928, 2861, 1638, 1546, 1451, 1400, 1141, 1013. **HRMS** (ESI): $C_{11}H_{20}N_3O$ [M+H]$^+$; calculated 210.1600, found 210.1600.

F36 NOESY correlations:

2-H_A: 2-H_B, 3-H, 11-H, 14-H_A

2-H_B: 2-H_A, 3-H, 8-H, 14-H_A, 14-H_B

3-H: 2-H_A, 5-H_B, 8-H

9-H: 8-H and 10-H_B

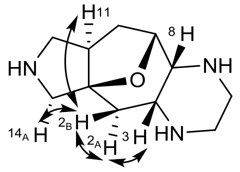

2-H_A

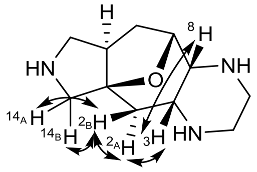

2-H_B

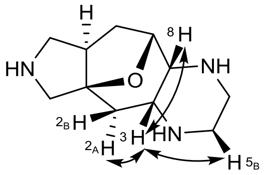

3-H

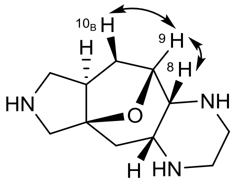

9-H

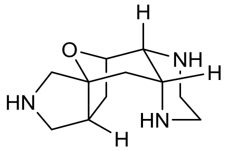

conformational diagram

5.4.3.17 Preparation of a fragment derived from scaffold S21

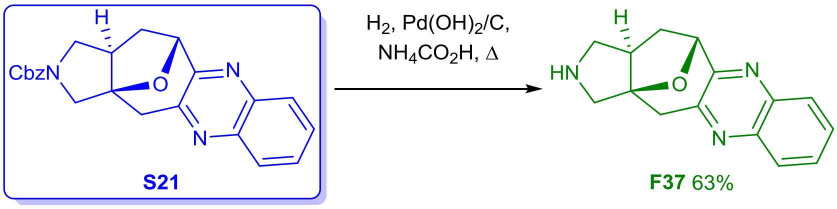

(*1R,*13R**,*15R**)-19-Oxa-4,11,17-triazapentacyclo[11.5.1.0^{1,15}.0^{3,12}.0^{5,10}]nonadeca-3,5(10),6,8,11-pentaene F37**

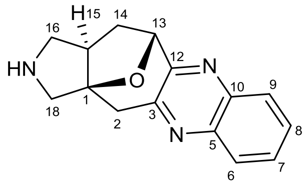

F37

To a stirred solution of quinoxaline **S21** (400 mg, 1.03 mmol, 1.00 eq.) in EtOH (10 mL) was added 20 wt% $Pd(OH)_2/C$ (40 mg, 10% w/w) and NH_4CO_2H (325 mg, 5.15 mmol, 5.00 eq.). The reaction mixture was heated at reflux for 15 h, then additional 20 wt% $Pd(OH)_2/C$ (40 mg, 10% w/w) and NH_4CO_2H (325 mg, 5.15 mmol, 5.00 eq.) were added. The reaction mixture was heated for a further 6 h, then cooled to rt. The reaction mixture was filtered through a pad of Celite eluting with EtOH, then concentrated *in vacuo*. The residue was partitioned between CH_2Cl_2 (25 mL) and washed with sat. $NaHCO_3$ solution (25 mL). The phases were separated and the aqeuous layer was extracted with CH_2Cl_2 (3 × 10 mL). The combined organics were dried over $MgSO_4$, filtered, and concentrated *in vacuo*. Flash chromatography eluting with 50:8:1 CH_2Cl_2–EtOH–NH_3/MeOH gave the *title compound* **F37** (165 mg, 0.65 mmol, 63%) as a colourless oil. **R_f** 0.10 (50:8:1 CH_2Cl_2–EtOH–NH_3/MeOH). **^{1}H NMR** (500 MHz, $CDCl_3$, NH not observed): δ 8.04-7.97 (2H, m, 6-H and 9-H), 7.75-7.70 (2H, m, 7-H and 8-H), 5.41 (1H, d, *J* 6.5, 13-H), 3.62 (1H, d, *J* 17.3, 2-H_A), 3.40 (1H, d, *J* 12.8, 18-H_A), 3.14 (1H, dd, *J* 12.4, 7.8, 16-H_B), 3.09 (1H, d, *J* 17.3, 2-H_B), 2.90 (1H, d, *J* 12.4, 16-H_A), 2.79 (1H, d, *J* 12.8, 18-H_B), 2.58 (1H, dd, *J* 12.6, 9.2, 14-H_A), 2.54-2.47 (1H, m, 15-H), 2.13 (1H, app. dt, *J* 13.6, 6.5, 14-H_B). **^{13}C NMR** (125 MHz, $CDCl_3$): δ 155.4 (Ar-C_q), 151.3 (Ar-C_q), 142.1 (Ar-C_q), 140.7 (Ar-C_q), 129.8 (Ar-C), 129.7 (Ar-C), 128.9 (Ar-C), 128.7 (Ar-C), 93.6 (1-C), 81.0 (13-C), 56.8 (18-C), 55.2 (16-C), 47.5 (15-C), 44.0 (14-C), 40.0 (2-C). **IR** ν_{max}(film)/cm^{-1} 2962, 2934, 1489, 1177, 1016, 958, 764. **HRMS** (ESI): $C_{15}H_{16}N_3O$ $[M+H]^+$; calculated 254.1288, found 254.1289.

5.4.3.18 Preparation of fragments derived from scaffold S24

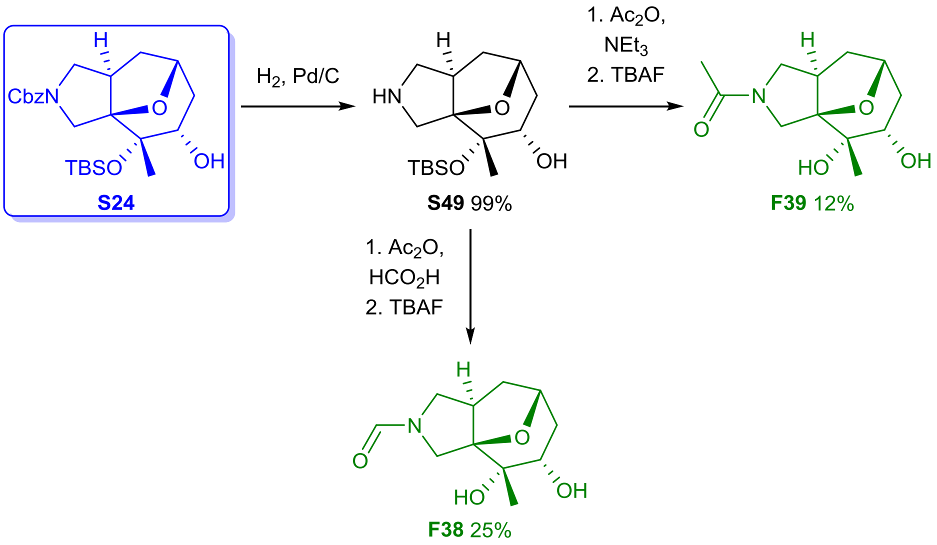

(*1R**,*5R**,*7R**,*9S**,*10S**)-10-[(*tert*-butyldimethylsilyl)oxy]-10-methyl-11-oxa-3-azatricyclo[5.3.1.0^{1,5}]undecan-9-ol S49

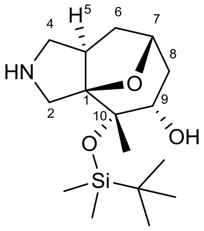

S49

Hydrogenation was carried out following general procedure **B**, using compound **S24** (142 mg, 0.32 mmol) and Pd/C (14 mg, 10% w/w) in MeOH (10 mL) over 15 h. The reaction mixture was filtered through Celite then concentrated *in vacuo* to give the *title compound* **S49** (99 mg, 0.32 mmol, 99%) as a colourless oil which was carried on to the subsequent steps without further purification. **^{1}H NMR** (400 MHz, $CHCl_3$, 50:50 mixture of rotamers, characteristic peaks): δ 4.41 (1H, dd, *J* 6.5, 5.0, 7-H), 3.67 (1H, d, *J* 3.8), 3.20-2.94 (4H, m), 2.75 (1H, dd, *J* 11.6, 4.3), 2.36 (1H, dd, *J* 12.2, 8.9), 2.18-2.10 (3H, m), 1.76-1.69 (1H, m, 6-H_B), 1.69-1.63 (1H, m, 8-H_A), 1.29 (3H, s, C_qCH_3), 0.94 (9H, s, $SiC_q(CH_3)_3$), 0.12 (3H, s, $SiCH_3$), 0.11 (3H, s, $SiCH_3$).

(*1R*,5R*,7R*,9S*,10S**)-9,10-dihydroxy-10-methyl-11-oxa-3-azatricyclo[5.3.1.0^{1,5}]undecane-3-carbaldehyde F38

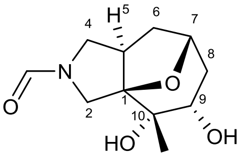

F38

N-Formylation was carried out by following general procedure **D**, using compound **S49** (50 mg, 0.16 mmol) and Ac_2O (30 eq.). *O*-Silyl deprotection was carried out by following general procedure **H**, using the crude residue. Purification by SCX SPE following general procedure **A**, eluting with MeOH, followed by flash chromatography eluting with 97:3:1 CH_2Cl_2–EtOH–NH_3/MeOH gave the *title compound* **F38** (9 mg, 40 μmol, 25%, 2 steps) as a colourless oil. **R_f** 0.09 (97:3:1 CH_2Cl_2–EtOH–NH_3/MeOH). **^{1}H NMR** (400 MHz, $CHCl_3$, 50:50 mixture of rotamers, 2 × OH not observed): δ 8.11 (1H, 2 × s, NCHO), 4.55-4.45 (1H, m, 7-H), 4.00 (0.5H, dd, *J* 10.5, 9.3, 4-H_B), 3.90 (0.5H, d, *J* 13.2, 2-H_A), 3.85 (0.5H, d, *J* 13.2, 2-H_A), 3.79-3.64 (2H, m, includes: 0.5H, 2-H_B; 0.5H, 4-H_B; and 1H, 9-H), 3.53-3.37 (1.5H, m, includes: 1H, 5-H; and at δ 3.49: 0.5H, d, *J* 13.2, 2-H_B), 3.33-3.26 (1H, m, 4-H_A), 2.66-2.61-2.51 (1H, m, 6-H_A, includes at δ 2.61: 0.5H, dd, *J* 12.2, 8.6; and at δ 2.55: 0.5H, dd, *J* 12.2, 8.6), 2.14 (1H, dt, *J* 14.8, 4.5, 8-H_B), 1.84-1.74 (2H, m, 6-H_B and 8-H_A), 1.31 (1.5H, C_qCH_3), 1.30 (1.5H, s, C_qCH_3). **^{13}C NMR** (100 MHz, MeOD-d_4, mixture of two rotamers): δ 163.3 (NCHO), 162.9 (NCHO), 95.3 (1-C), 94.9 (1-C), 78.8 (7-C), 78.4 (7-C), 73.8 (9-C), 73.7 (9-C), 71.3 (10-C), 71.1 (10-C), 55.8 (4-C), 52.8 (2-C), 51.8 (2-C), 41.4 (5-C), 40.9 (5-C), 39.6 (6-C), 37.6 (6-C), 37.6 (8-C), 37.5 (8-C), 23.1 (C_qCH_3) [20 of 22 expected peaks observed]. **IR** ν_{max}(film)/cm^{-1} 3359 (br. OH), 2937, 2880, 1644 (CO), 1432, 1386, 1163, 1009. **HRMS** (ESI): $C_{11}H_{18}NO_4$ [M+H]$^+$; calculated 228.1230, found 228.1230.

1-[(*1R*,5R*,7R*,9S*,10S**)-9,10-Dihydroxy-10-methyl-11-oxa-3-azatricyclo[5.3.1.0^{1,5}]undecan-3-yl]ethan-1-one F39 (≡ 24)

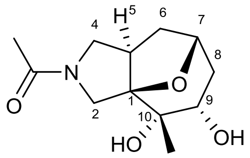

F39 ≡ 24

N-Acylation was carried out by following general procedure **E**, using **S49** (49 mg, 0.16 mmol). The reaction mixture was stirred at rt for 10 min, then sat. aq. NH_4Cl (10 mL) was added. The phases were separated and the aqueous layer was extracted with CH_2Cl_2 (2 × 10 mL). The combined organic phases were dried over $MgSO_4$, filtered, and concentrated *in vacuo*. *O*-Silyl deprotection was carried out by following general procedure **H**, using the crude residue. Flash chromatography eluting with 29:1:0.1 CH_2Cl_2–EtOH–NH_3/MeOH gave the *title compound* **F39** (≡ **24**: 4.5 mg, 18.5 μmol, 12%, 2 steps) as a colourless oil. **R_f** 0.10 (29:1:0.1 CH_2Cl_2–EtOH–NH_3/MeOH). **^{1}H NMR** (400 MHz, $CHCl_3$, 40:60 mixture of rotamers, 2 × OH not observed): δ 4.54-4.48 (1H, m, 7-H), 4.04-3.91 (1.4H, m, includes: 1H, 4-H_B; and 0.4H, 2-H_A), 3.84 (0.6H, d, *J* 13.3, 2-H_A), 3.68 (1H, d, *J* 4.2, 9-H), 3.64 (0.4H, d, *J* 13.3, 2-H_B), 3.59 (0.6H, d, *J* 13.3, 2-H_B), 3.49 (0.6H, app. ddd, *J* 17.9,

8.4, 3.1, 5-H), 3.44-3.26 (1H, m, includes: 0.6H, 4-H_A; and 0.4H, 5-H), 3.17 (0.4H, dd, *J* 12.2, 7.8, 4-H_A), 2.61 (1H, dd, *J* 11.6, 8.9, 6-H_A), 2.17-2.09 (1H, m, 8-H_B), 2.07 (1.2H, s, NH(CO)CH_3), 2.05 (1.8H, s, NH(CO)CH_3), 1.89-1.74 (2H, m, 6-H_B and 8-H_A), 1.31 (1.2H, s, C_qCH_3), 1.30 (1.8H, s, C_qCH_3). **^{13}C NMR** (100 MHz, MeOD-d_4, mixture of two rotamers): δ 171.7 (N(*C*O)CH_3), 96.3 (1-C), 94.9 (1-C), 78.6 (2 peaks, 7-C), 73.8 (2 peaks, 9-C), 71.1 (2 peaks, 10-C), 57.6 (4-C), 55.7 (4-C), 53.5 (2-C), 51.6 (2-C), 42.1 (5-C), 40.7 (5-C), 38.9 (6-C), 38.4 (6-C), 37.6 (8-C), 37.5 (8-C), 23.1 (2 peaks, C_q*C*H_3), 22.0 (N(CO)*C*H_3), 21.8 (N(CO)*C*H_3) [23 of 24 expected peaks observed]. **IR** ν_{max}(film)/cm^{-1} 3367 (br. OH), 2942, 2881, 1620 (CO), 1455, 1426, 1040, 1009. **HRMS** (ESI): $C_{12}H_{20}NO_4$ [M+H]$^+$; calculated 242.1387, found 242.1387.

5.4.3.19 Preparation of fragments derived from scaffold S25

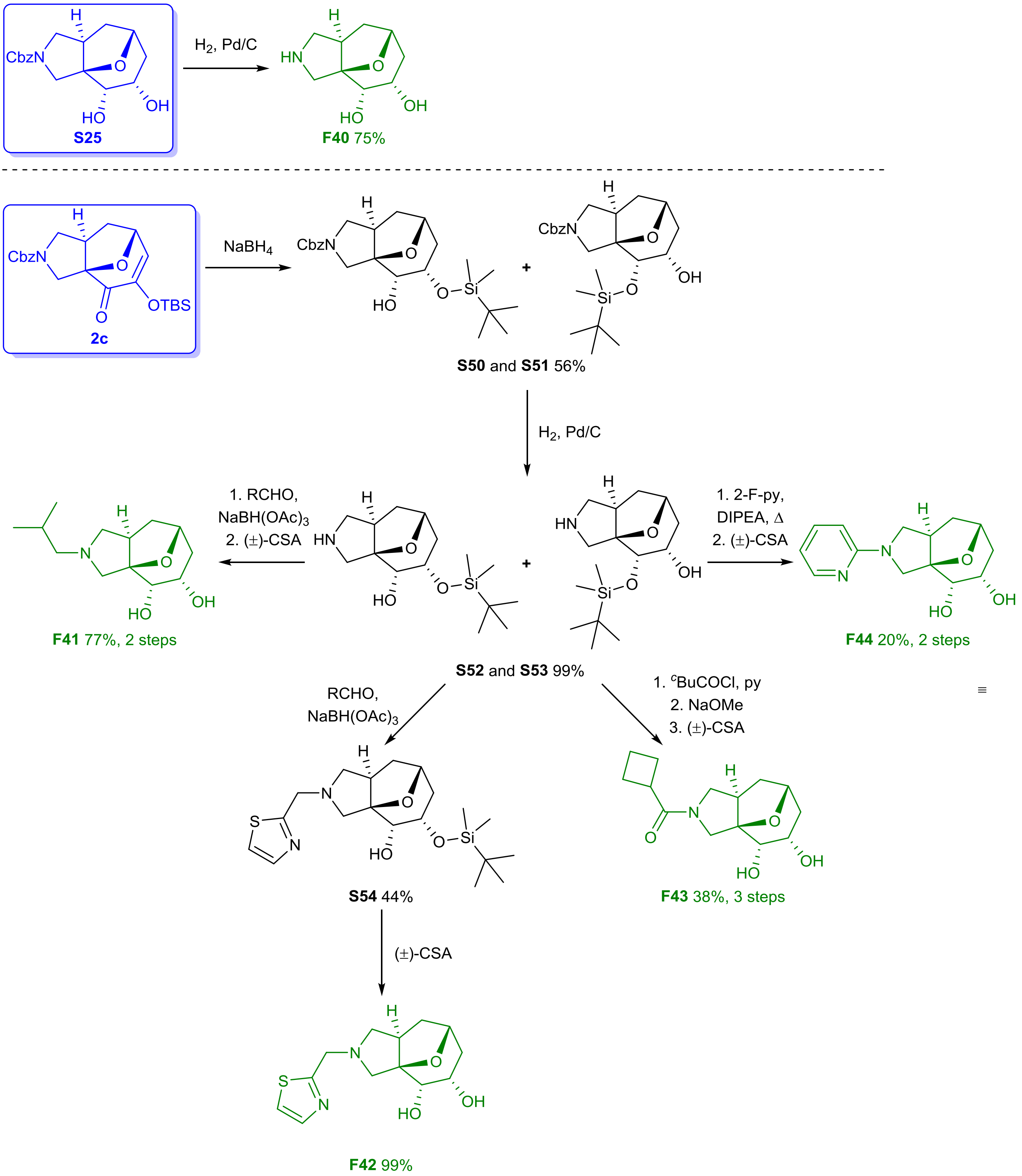

(*1R,*5R**,*7R**,*9S**,*10S**)-11-oxa-3-azatricyclo[5.3.1.0^{1,5}]undecane-9,10-diol F40**

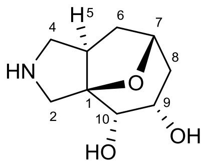

F40

Hydrogenation was carried out following general procedure **B**, using compound **S25** (168 mg, 0.53 mmol) and Pd/C (20 mg, 10% w/w) in MeOH (10 mL) over 15 h. Flash chromatography eluting with 50:8:1 CH_2Cl_2–EtOH–NH_3/MeOH, gave the *title compound* **F40** (74 mg, 0.40 mmol, 75%) as a colourless oil. **^{1}H NMR** (500 MHz, MeOD-d_4, NH and 2 × OH not observed): δ 4.41-4.37 (1H, m, 7-H), 4.04 (1H, td, *J* 4.7, 1.1, 9-H), 3.73 (1H, d, *J* 4.7, 10-H), 3.22-3.16 (1H, m, 5-H), 3.09-3.02 (2H, m, includes at δ 3.07: 1H, d, *J* 12.4, 2-H_B; and at δ 3.04: 1H, dd, *J* 11.8, 8.8, 4-H_B), 2.85 (1H, d, *J* 12.4, 2-H_A), 2.66 (1H, dd, *J* 11.8, 5.3, 4-H_A), 2.46 (1H, dd, *J* 12.2, 9.0, 6-H_A), 2.02 (1H, dtd, *J* 14.7, 4.7, 1.1, 8-H_B), 1.81 (1H, dt, *J* 14.7, 1.1, 8-H_A), 1.76-1.70 (1H, m, 6-H_B). **^{13}C NMR** (125 MHz, MeOD-d_4): δ 95.0 (1-C), 78.0 (7-C), 69.6 (10-C), 68.3 (9-C), 56.2 (4-C), 53.3 (2-C), 43.0 (5-C), 39.0 (8-C), 38.8 (6-C). **IR** ν_{max}(film)/cm^{-1} 3359 (br. OH), 2952, 2515, 1635, 1428, 1100, 1067, 1035. **HRMS** (ESI): $C_9H_{15}NO_3$ [M+H]$^+$; calculated 186.1125, found 186.1124.

Benzyl (*1R*,*5R**,*7R**,*9S**,*10S**)-9-[(*tert*-butyldimethylsilyl)oxy]-10-hydroxy-11-oxa-3-azatricyclo[5.3.1.0^{1,5}]undecane-3-carboxylate S50 and benzyl (*1R**,*5R**,*7R**,*9S**,*10S**)-10-[(*tert*-butyldimethylsilyl)oxy]-9-hydroxy-11-oxa-3-azatricyclo[5.3.1.0^{1,5}]undecane-3-carboxylate S51

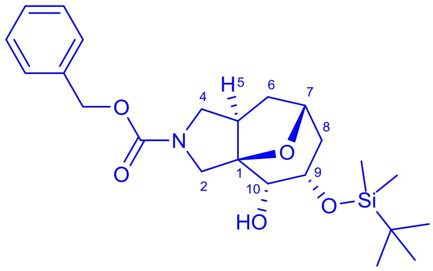

S50

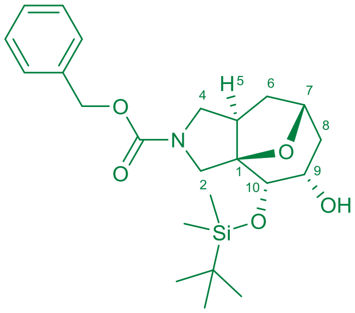

S51

$NaBH_4$ (680 mg, 17.9 mmol, 2.20 eq.) was added to a stirred solution of cycloadduct **2c** (3.50 g, 8.10 mmol, 1.00 eq.) in MeOH (45 mL) at 0 °C. The reaction mixture was stirred at 0 °C for 1 h, then concentrated *in vacuo*. The resulting residue was diluted in EtOAc (50 mL) and washed with brine (50 mL). The aqueous layer was extracted with EtOAc (2 × 25 mL). The combined organics were dried over $MgSO_4$ and concentrated *in vacuo*. Flash chromatography eluting with 4:1 pentane–EtOAc gave the *title compounds* **S50** and **S51** (1.98 g, 4.57 mmol, 56%, 2:1 mixture of regioisomers) as a colourless oil. $\mathbf{R_f}$ 0.15 (4:1 pentane–EtOAc). **^{1}H NMR** (500 MHz, $CDCl_3$, 2:1 mixture regiosomers, **characteristic peaks**): δ 7.40-7.27 (5H, m, major and minor Cbz Ar-H), 5.16-5.07 (2H, m, major and minor OCH_2Ph), 4.53-4.43 (1H, m, major and minor 7-H), 4.19-4.12 (0.66H, m, major 9-H), 4.03-3.98 (0.33H, m, minor 9-H), 3.97-3.78 (2H, m), 3.74-3.61 (1H, m), 3.60-3.47 (1H, m), 3.22-3.09 (1.33H, m, includes 0.33H, minor 5-H), 3.09-2.98 (0.66H, m, major 5-H), 2.73-2.63 (1H, m), 2.52 (0.33H, dd, *J* 12.0, 8.4), 2.43-2.33 (0.66H, m), 2.12-1.96 (1H, m), 1.92 (0.33H, d, *J* 14.4), 1.89-1.74 (1.66H, includes at δ 1.78: 0.66H, d, *J* 14.4), 0.98-0.86 (9H, m, $SiC_q(CH_3)_3$), 0.19-0.04 (6H, m, $SiCH_3$). **IR** ν_{max}(film)/cm^{-1} 2952, 2929, 2885, 2856, 1687 (CO), 1423, 1116, 1087. **HRMS** (ESI): $C_{23}H_{36}NO_5Si$ $[M+H]^+$; calculated 434.2363, found 434.2364.

(*1R,*5R**,*7R**,*9S**,*10S**)-9-[(*tert*-Butyldimethylsilyl)oxy]-11-oxa-3-azatricyclo[5.3.1.0^{1,5}]undecan-10-ol S52 and (*1R**,*5R**,*7R**,*9S**,*10S**)-10-[(*tert*-butyldimethylsilyl)oxy]-11-oxa-3-azatricyclo[5.3.1.0^{1,5}]undecan-9-ol S53**

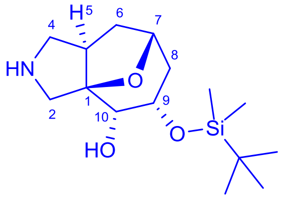

S52

+

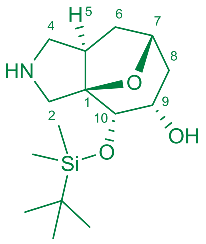

S53

Hydrogenation was carried out following general procedure **B**, using compounds **S50** and **S51** (1.98 g, 4.61 mmol) and Pd/C (200 mg, 10% w/w) in EtOH (40 mL) over 15 h Filtration followed by concentration gave the *title compounds* **S52** and **S53** (1.36 g, 4.54 mmol, 99%) as a colourless foam which was not purified further. **^{1}H NMR** (500 MHz, $CDCl_3$, 2:1 mixture of regioisomers, NH not observed): δ 4.55-4.47 (1H, m, major and minor 7-H), 4.14 (0.66H, t, *J* 4.0, major 9-H), 3.99 (0.33H, t, *J* 4.1, minor 9-H), 3.90 (0.33H, d, *J* 4.1, minor 10-H), 3.74 (0.66H, t, *J* 4.0, major 10-H), 3.65 (0.66H, d, *J* 12.4, major 2-H_A), 3.56 (0.66H, dd, *J* 11.6, 9.4, major 4-H_B), 3.47-3.36 (1.33H, m, includes: 0.33H, minor 4-H_B and at δ 3.41: 1H, d, *J* 12.4, major 2-H_B), 3.34-3.25 (1H, m, major and minor 5-H), 3.25-3.18 (0.66H, m, includes: 0.33H, minor 4-H_A; and at δ 3.22: 0.33H, d, *J* 12.4, minor 2-H_A), 3.14 (0.66H, dd, *J* 11.6, 6.8, major 4-H_A), 2.87 (0.66H, s, major OH), 2.60-2.53 (0.66H, m, includes at δ 2.56: 0.33H, s, minor OH; and at δ 2.57: 0.33H, dd, *J* 12.4, 8.8, minor 6-H_A), 2.42 (0.66H, dd, *J* 12.4, 8.8, major 6-H_A), 2.07 (0.66H, app. dt, *J* 14.8, 4.4, major 8-H_B), 2.11-1.87 (1.66H, m, includes: 0.33H, minor 8-H_A; 0.33H, minor 8-H_B; and, 1H, minor and major 6-H_B), 1.76 (0.66H, d, *J* 14.8, major 8-H_A), 0.93 (3H, s, minor $SiC_q(CH_3)_3$), 0.93 (6H, s, major, $SiC_q(CH_3)_3$), 0.16 (1H, s, minor $SiCH_3$), 0.13 (1H, s, minor $SiCH_3$), 0.12 (2H, s, major $SiCH_3$), 0.11 (2H, s, major $SiCH_3$). **^{13}C NMR** (125 MHz, $CDCl_3$, 2:1 mixture of regioisomers): δ 91.9 (major, 1-C), 91.1 (minor, 1-C), 76.6 (7-C, major), 76.2 (minor, 7-C), 69.0 (minor, 10-C), 68.1 (major, 9-C), 67.6 (major, 10-C), 67.3 (minor, 9-C), 53.2 (minor, 4-C), 52.8 (major, 4-C), 50.1 (major, 2-C), 50.1 (minor, 2-C), 40.0 (major and minor, 5-C), 38.2 (minor, 8-C), 38.1 (major, 8-C), 37.0 (major, 6-C), 36.1 (minor, 6-C), 25.2 (major and minor, $SiC_q(CH_3)_3$), 18.0 (major and minor, 2 peaks, SiC_q), −4.5 (minor, $SiCH_3$), −4.6 (major, $SiCH_3$), −4.7 (minor, $SiCH_3$), −5.2 (major, $SiCH_3$). **IR** ν_{max}(film)/cm^{-1} 3352 (br. s, OH), 2951, 2928, 2891, 2856, 1252, 1109, 1084. **HRMS** (ESI): $C_{15}H_{30}NO_3Si$ $[M+H]^+$; calculated 300.1995, found 300.1993.

(*1R**,*5R**,*7R**,*9S**,*10S**)-3-(2-Methylpropyl)-11-oxa-3-azatricyclo[5.3.1.0^{1,5}]undecane-9,10-diol F41

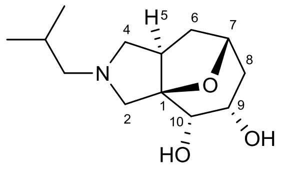

F41

Reductive amination was carried out by following general procedure **C**, using amines **S52** and **S53** (300 mg, 1.00 mmol) and isobutyraldehyde (2.5 eq.). The reaction mixture was partitioned between EtOAc (25 mL) and brine (25 mL). The phases were separated and the aqueous phase was extracted with EtOAc (2 × 25 mL). The combined organic phases were dried over $MgSO_4$, filtered and concetrated *in vacuo* to give a colourless oil (290 mg). *O*-Silyl deprotection was carried out by following general procedure **G**, using the crude residue and (±)-camphorsulfonic acid (4.0 eq.). Flash chromatography eluting with 93:7:1 CH_2Cl_2–EtOH–NH_3/MeOH gave the *title compound* **F41** (185 mg, 0.77 mmol, 77%) as a colourless oil. **R*f*** 0.18 (50:8:1 CH_2Cl_2–EtOH–NH_3/MeOH). **^{1}H NMR** (500 MHz, $CDCl_3$, one OH not observed): δ 4.50-4.45 (1H, m, 7-H), 4.13 (1H, app. t, *J* 4.4, 9-H), 3.80 (1H, d, *J* 4.8, 10-H), 3.25-3.17 (2H, m, includes: 1H, 5-H; and at δ 3.23: 1H, d, *J* 10.6, $NCH_AH_BCH(CH_3)_2$), 3.02 (1H, app. t, *J* 8.1, 4-H_A), 2.60 (1H, s, OH), 2.43 (1H, d, *J* 10.6, $NCH_AH_BCH(CH_3)_2$), 2.35-2.19 (4H, m, 2-H, 4-H_B, 6-H_A), 2.06 (1H, dt, *J* 14.8, 4.8, 8-H_B), 1.85 (1H, app. d, *J* 14.7, 8-H_A), 1.81-1.69 (2H, m, 6-H_B and $CH(CH_3)_2$), 0.93 (3H, d, *J* 6.6, $CH(CH_3)_A$), 0.91 (3H, d, *J* 6.6, $CH(CH_3)_B$). **^{13}C NMR** (125 MHz, $CDCl_3$): δ 91.7 (1-C), 77.4 (7-C), 70.1 (10-C), 67.5 (9-C), 64.8 (2-C), 62.3 (4-C), 60.9 ($NCH_2CH(CH_3)_3$), 41.4 (5-C), 37.8 (8-C), 34.8 (6-C), 27.3 ($CH(CH_3)_2$), 21.3 ($CH(CH_3)_A$), 21.2 ($CH(CH_3)_B$). **IR** ν_{max}(film)/cm^{-1} 3398 (br. OH), 2951, 2807, 1467, 1074, 1047, 1034. **HRMS** (ESI): $C_{13}H_{24}NO_3$ $[M+H]^+$; calculated 242.1756, found 242.1754.

(*1R**,*5R**,*7R**,*9S**,*10S**)-9-[(*tert*-Butyldimethylsilyl)oxy]-3-(1,3-thiazol-2-ylmethyl)-11-oxa-3-azatricyclo[5.3.1.0^{1,5}]undecan-10-ol S54

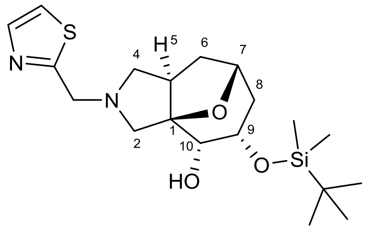

S54

Reductive amination was carried out by following general procedure **C**, using amines **S52** and **S53** (304 mg, 1.02 mmol) and 1,3-thiazolecarbaldehyde (2.5 eq.). The reaction mixture was partitioned between EtOAc (25 mL) and brine (25 mL). The phases were separated and the aqueous phase was extracted with EtOAc (2 × 25 mL). The combined organic phases were dried over $MgSO_4$, filtered and concentrated *in vacuo*.[*] Flash chromatography eluting with 1:1 pentane–EtOAc gave the *title compound* **S54** (177 mg, 0.45 mmol, 44%, single regioisomer) as a colourless oil. **R*f*** 0.28 (1:1 pentane–EtOAc). **^{1}H NMR** (500 MHz, $CDCl_3$): δ 7.70 (1H, d, *J* 3.4, thiazole 4-H), 7.26 (1H, d, *J* 3.4, thiazole 5-H), 4.47-4.43 (1H, m, 7-H),

[*] The crude product was a 2:1 mixture of regioisomers (as judged by analysis of the crude residue by NMR spectroscopy at 300 Hz).

4.13 (1H, app. t, *J* 4.2, 9-H), 4.08 (1H, d, *J* 14.7, 2-H_A), 4.01 (1H, d, *J* 14.7, 2-H_B), 3.74-3.64 (1H, m, 10-H), 3.29 (1H, d, *J* 10.4, NC*H*$_A$H$_B$Ar), 3.12-3.01 (2H, m, 5-H and 4-H_A), 2.72-2.60 (2H, m, includes: 1H, OH; and at δ 2.69: 1H, d, *J* 10.4, NCH$_A$*H*$_B$Ar), 2.52 (1H, app. t, *J* 7.5, 4-H_B), 2.23 (1H, dd, *J* 12.2, 8.3, 6-H_A), 2.11-2.04 (1H, m, 8-H_B), 1.83-1.70 (2H, m, 6-H_B and at δ 1.75: 1H, d, *J* 14.7, 8-H_A), 0.94 (9H, s, SiC(CH_3)$_3$), 0.12 (3H, s, SiCH_3), 0.11 (3H, s, SiCH_3). **^{13}C NMR** (125 MHz, $CDCl_3$): δ 169.6 (thiazole 2-C), 142.3 (thiazole 4-C), 119.5 (thiazole 5-C), 92.7 (1-C), 76.6 (7-C), 69.1 (9-C or 10-C), 68.9 (9-C or 10-C), 61.5 (4-C), 59.8 (N*C*H_2Ar), 56.6 (2-C), 41.0 (5-C), 38.8 (8-C), 35.5 (6-C), 26.0 (SiC$_q$(*C*H_3)$_3$), 18.1 (Si*C*$_q$), −4.4 (Si*C*H_3), −5.1 (Si*C*H_3). **IR** ν_{max}(film)/cm^{-1} 2950, 2929, 2855, 1253, 1097, 1061, 934, 836. **HRMS** (ESI): $C_{19}H_{33}N_2O_3SSi$ [M+H]$^+$; calculated 397.1981, found 397.1986.

(*1R*,5R*,7R*,9S*,10S**)-3-(1,3-thiazol-2-ylmethyl)-11-oxa-3-azatricyclo[5.3.1.0^{1,5}]undecane-9,10-diol F42

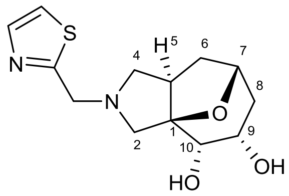

F42

O-Silyl deprotection was carried out by following general procedure **G**, using compound **S54** (177 mg, 0.44 mmol) and (±)-camphorsulfonic acid (10.0 eq.) over 2.5 days. Flash chromatography eluting with 93:7:1 CH_2Cl_2–EtOH–NH_3/MeOH gave the *title compound* **F42** (126 mg, 0.44 mmol, 99%) as a pale yellow foam. **R*f*** 0.13 (93:7:1 CH_2Cl_2–EtOH–NH_3/MeOH). **^{1}H NMR** (500 MHz, $CDCl_3$, one OH not observed): δ 7.72 (1H, d, *J* 3.2, thiazole 4-H), 7.28 (1H, d, *J* 3.2, thiazole 5-H), 4.52-4.45 (1H, m, 7-H), 4.14 (1H, app. t, *J* 4.3, 9-H), 4.09 (1H, d, *J* 14.7, 2-H_A), 3.98 (1H, d, *J* 14.7, 2-H_B), 3.80 (1H, d, *J* 4.5, 10-H), 3.28 (1H, d, *J* 10.6, NC*H*$_A$H$_B$Ar), 3.26-3.20 (1H, m, 5-H), 3.10 (1H, app. t, *J* 8.3, 4-H_A), 2.88 (1H, s, OH), 2.65 (1H, d, *J* 10.6, NCH$_A$*H*$_B$Ar), 2.46 (1H, app. t, *J* 8.3, 4-H_B), 2.29 (1H, dd, *J* 12.2, 8.8, 6-H_A), 2.07 (1H, dt, *J* 14.7, 4.5, 8-H_B), 1.85 (1H, d, *J* 14.7, 8-H_A), 1.82-1.76 (1H, m, 6-H_B). **^{13}C NMR** (125 MHz, $CDCl_3$): δ 169.4 (thiazole 2-C), 142.5 (thiazole 4-C), 119.5 (thiazole 5-C), 92.1 (1-C), 77.3 (7-C), 69.9 (10-C), 67.6 (9-C), 61.6 (4-C), 60.2 (N*C*H_2Ar), 56.5 (2-C), 41.6 (5-C), 37.8 (8-C), 35.1 (6-C). **IR** ν_{max}(film)/cm^{-1} 3375 (br. OH), 2942, 2821, 1505, 1075, 1051, 1034. **HRMS** (ESI): $C_{13}H_{19}N_2O_3S$ [M+H]$^+$; calculated 283.1116, found 283.1111.

(*1R*,5R*,7R*,9S*,10S**)-3-Cyclobutanecarbonyl-11-oxa-3-azatricyclo[5.3.1.0^{1,5}]undecane-9,10-diol F43

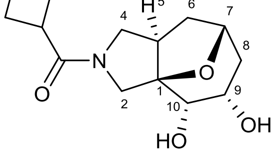

F43 ≡ 22

Cyclobutanecarbonyl chloride (0.57 mL, 5.00 mmol, 5.00 eq.) was added to a stirred solution of amines **S52** and **S53** (300 mg, 1.00 mmol, 1.00 eq., 2:1 mixture of regioisomers) in pyridine (10 mL) at 0 °C. The reaction mixture was warmed to rt then stirred for 15 h. Further cyclobutanecarbonyl chloride

(0.21 mL, 1.83 mmol, 2.50 eq.) was added and the reaction mixture was stirred for 1 h. The reaction mixture was concentrated *in vacuo*, then partitioned between EtOAc (25 mL) and sat. aq. $NaHCO_3$ solution (25 mL). The phases were separated and the aqueous phase was extracted with EtOAc (2 × 25 mL). The combined organic phases were dried over $MgSO_4$, filtered and concetrated *in vacuo*. Flash chromatography eluting with 1:9 pentane–EtOAc was used to isolate fractions with R_f= 0.48, giving a colourless oil (240 mg) {**LRMS** (HPLC-MS): $C_{25}H_{41}NO_5Si$; found 463.9 [M^+], consistent with the diacetylated product}. The residue (240 mg, [estimate 0.52 mmol], 1.00 eq) was diluted in MeOH (10 mL) and NaOMe (25 wt% in MeOH, 0.60 mL, 2.50 mmol, 5.00 eq.) was added. The reaction mixture was stirred for 1 h at rt, then concentrated *in vacuo*. The residue was diluted in CH_2Cl_2 (25 mL) and washed with H_2O (25 mL). The phases were separated and the aqueous phase was extracted with CH_2Cl_2 (2 × 10 mL). The combined organic phases were dried over $MgSO_4$, filtered and concetrated *in vacuo* to give a colourless oil (190 mg). The residue was diluted in MeOH (10 mL) and (±)-camphorsulfonic acid (465 mg, 2.00 mmol, 4.00 eq.) was added. The reaction mixture heated at 45 °C for 15 h, then concentrated *in* vacuo. Flash chromatography eluting with 93:7:1 CH_2Cl_2–EtOH–NH_3/MeOH gave the *title compound* **F43** (102 mg, 0.38 mmol, 38% [three steps]) as a colourless oil. **R_f** 0.38 (50:8:1 CH_2Cl_2–EtOH–NH_3/MeOH). **^{1}H NMR** (500 MHz, $CDCl_3$, 60:40 mixture of rotamers): δ 4.52-4.47 (1H, m, 7-H), 4.34-4.24 (0.6H, s, OH), 4.22-4.18 (0.4H, m, 9-H), 4.16 (0.6H, app. t, *J* 4.3, 9-H), 4.04 (0.4H, dd, *J* 11.9, 9.6, 4-H_B), 3.99 (0.6H, d, *J* 13.5, 2-H_A), 3.88-3.74 (2H, m, includes: 0.4H, 2-H_A; 0.6H, 4-H_B; and 1H, 10-H), 3.56 (0.6H, d, *J* 12.6, 2-H_B), 3.46 (0.4H, d, *J* 12.6, 2-H_B), 3.27-3.01 (3.4H, m, includes: 1H, 4-H_A; 1H, 5-H; 1H, cyclobutyl 1-H; and 0.4H, OH), 2.76 (0.6H, s, OH), 2.51 (1H, td, *J* 12.1, 8.4, 6-H_A), 2.39 (0.4H, s, OH), 2.37-2.25 (2H, m, cylobutyl 2-H), 2.19-2.02 (3H, m, includes: 1H, 8-H_A; and 2H, cyclobutyl 2-H), 2.00-1.76 (4H, m, includes: 1H, 6-H_B; 1H, 8-H_B; and 2H, cyclobutyl 3-H). **^{13}C NMR** (125 MHz, $CDCl_3$): δ 173.3 (major and minor, N(*C*O)CH), 92.6 (minor, 1-C), 90.7 (major, 1-C), 76.6 (major, 7-C), 76.4 (minor, 7-C), 68.3 (minor, 10-C), 67.5 (major, 10-C), 67.4 (major, 9-C), 67.2 (minor, 9-C), 54.7 (major, 4-C), 54.4 (minor, 4-C), 51.7 (major, 2-C), 51.4 (minor, 2-C), 41.1 (major and minor, 5-C), 39.0 (minor, cyclobutyl 1-C), 38.2 (minor, 8-C), 38.1 (major, cyclobutyl 1-C), 37.2 (2 peaks: minor, 6-C and major, 8-C), 36.4 (major, 6-C), 24.9 (minor, cyclobutyl 2-C), 24.7 (major, cyclobutyl 2-C), 24.6 (major, cyclobutyl 2-C'), 24.5 (minor, cyclobutyl 2-C'), 18.0 (major and minor, cyclobutyl 3-C). **IR** ν_{max}(film)/cm^{-1} 3359 (br. OH), 2945, 2878, 1615 (CO), 1451, 1220, 1078, 1052. **HRMS** (ESI): $C_{14}H_{22}NO_4$ [M+H]$^+$; calculated 268.1549, found 268.1543.

(*1R,*5R**,*7R**,*9S**,*10S**)-3-(pyridin-2-yl)-11-oxa-3-azatricyclo[5.3.1.0^{1,5}]undecane-9,10-diol F44**

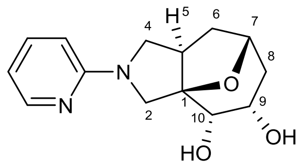

F44

2-Fluoropyridine (74 µL, 0.84 mmol, 1.0 eq.) was added to a stirred solution of amines **S52** and **S53** (256 mg, 0.84 mmol, 1.00 eq., 2:1 mixture of regioisomers) and DIPEA (18 µL, 1.20 mmol, 1.20 eq.) in DMA (5.0 mL). The reaction mixture was heated at 120 °C for 15 h. The reaction mixture was concentrated *in vacuo*, then partitioned between EtOAc (25 mL) and sat. aq. $NaHCO_3$ solution (25 mL). The phases were separated and the aqueous phase was extracted with EtOAc (2 × 25 mL). The combined organic phases were dried over $MgSO_4$, filtered and concetrated *in vacuo*. *O*-Silyl deprotection was carried out by following general procedure **G**, using the the crude residue (130 mg) and (±)-camphorsulfonic acid (2.7 eq.). Flash chromatography eluting with 90:9:1 CH_2Cl_2–EtOH–NH_3/MeOH gave the *title compound* **F44** (43 mg, 0.16 mmol, 20% [two steps]) as a colourless amorphous solid. **R_f** 0.17 (2:1 CH_2Cl_2–"Mixture-A"). **^{1}H NMR** (500 MHz, $CDCl_3$, 2 × OH not observed): δ 8.02 (1H, dd, *J* 5.3, 1.5, pyridine 6-H), 7.44 (1H, ddd, *J* 8.8, 6.9, 1.5, pyridine 4-H), 6.53 (1H, dd, *J* 6.9, 5.3, pyridine 5-H), 6.36 (1H, d, *J* 8.8, pyridine 3-H), 4.52 (1H, dd, *J* 7.4, 4.4, 7-H), 4.14 (1H, app. t, *J* 4.4, 9-H), 4.08 (1H, d, *J* 11.8, 2-H_A), 3.96 (1H, d, *J* 4.7, 10-H), 3.88 (1H, t, *J* 9.5, 4-H_A), 3.69 (1H, d, *J* 11.8, 2-H_B), 3.40-3.32 (1H, m, 5-H), 3.20 (1H, dd, *J* 9.5, 8.4, 4-H_B), 2.62 (1H, dd, *J* 12.2, 8.5, 8-H_B), 2.06 (1H, dt, *J* 14.5, 4.4, 6-H_A), 1.99-1.92 (2H, m, 6-H_B and 8-H_A). **^{13}C NMR** (125 MHz, $CDCl_3$): δ 156.9 (pyridine 2-C), 147.2 (pyridine 6-C), 137.7 (pyridine 4-C), 111.7 (pyridine 5-C), 107.6 (pyridine 3-C), 91.8 (1-C), 76.6 (7-C), 67.7 (9-C or 10-C), 67.5 (9-C or 10-C), 56.4 (4-C), 53.1 (2-C), 40.7 (5-C), 37.5 (8-C), 37.2 (6-C). **IR** ν_{max}(film)/cm^{-1} 3391 (br. OH), 2944, 2870, 1601, 1556, 1499, 1474, 1445. **HRMS** (ESI): $C_{13}H_{19}N_2O_3S$ $[M+H]^+$; calculated 263.1396, found 263.1395.

5.4.3.20 Preparation of fragments derived from scaffold S27

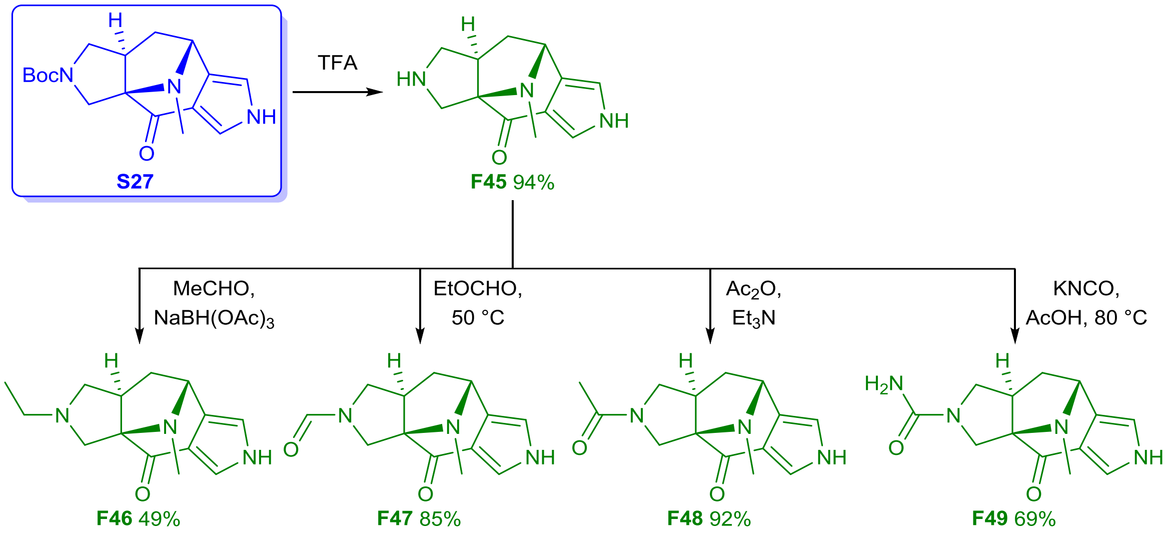

(1*R,8*R**,10*R**)-14-methyl-5,12,14-triazatetracyclo[6.5.1.0^{1,10}.0^{3,7}]tetradeca-3,6-dien-2-one F45**

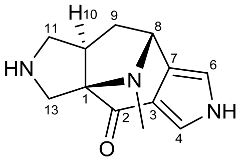

F45

Deprotection of Boc-protected compound **S27** (1.71 g, 5.39 mmol) was carried out by following general procedure **I**. Purification by SCX SPE eluting with MeOH, then sat. NH_3/MeOH, gave the *title compound* **F45** (1.10 g, 5.06 mmol, 94%) as a yellow solid. **Mp:** Decomposition observed above 180 °C. **^{1}H NMR** (500 MHz, MeOD-d_4, 333 K): δ 7.34 (1H, s, 4-H), 6.62 (1H, s, 6-H), 4.32 (1H, d, *J* 4.5, 8-H), 3.43 (1H, d, *J* 12.4, 13-H_A), 3.31 (1H, br. s, NH), 3.08 (1H, dd, *J* 11.1, 8.7, 11-H_B), 2.84 (1H, dd, *J* 11.1, 4.6, 11-H_A), 2.75 (1H, d, *J* 12.4, 13-H_B), 2.64-2.49 (1H, m, 10-H), 2.22 (3H, s, NCH_3), 2.08-2.00 (1H, m, 9-H_A), 1.97 (1H, app. t, *J* 10.0, 9-H_B). **^{13}C NMR** (125 MHz, MeOD-d_4, 333 K): δ 196.0, 128.4, 121.0, 119.5, 115.4, 83.4, 63.4, 54.2, 50.2, 48.3, 39.2, 33.0. **IR** ν_{max}(film)/cm^{-1} 2860, 1654, 1520, 1480, 1446, 1350, 1179, 902. **HRMS** (ESI): $C_{12}H_{16}N_3O$ [M+H]$^+$; calculated 218.1288, found 218.1291.

(1*R,8*R**,10*R**)-12-Ethyl-14-methyl-5,12,14-triazatetracyclo[6.5.1.0^{1,10}.0^{3,7}]tetradeca-3,6-dien-2-one F46**

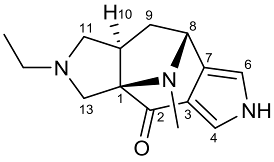

F46

Reductive amination was carried out by following general procedure **C**, using compound **F45** (151 mg, 0.70 mmol) and acetaldehyde (5.0 M in THF, 2.0 eq.). The reaction was quenched with H_2O (5 mL) and the layers separated. The aqueous layer was extracted with CH_2Cl_2 (2 x 10 mL) and the combined organic layers were dried, filtered and concentrated *in vacuo* to give a crude oil. The crude material was purified by SCX SPE eluting with MeOH, then sat. NH_3/MeOH, to give the *title compound* **F46** (83 mg, 0.34 mmol, 49%) as a yellow oil. **^{1}H NMR** (500 MHz, MeOD-d_4, 333 K, NH not observed):

δ 7.36 (1H, app. t, *J* 1.6, 4-H), 6.63 (1H, d, *J* 1.5, 6-H), 4.33 (1H, dd, *J* 5.5, 8-H), 3.60 (1H, d, *J* 10.0, 13-H_A), 3.01 (1H, dd, *J* 8.6, 7.8, 11-H_B), 2.67-2.47 (4H, m, 10-H, 11-H_A and NCH_2CH_3,), 2.30 (1H, d, *J* 10.0, 13-H_B), 2.21 (3H, s, NCH_3), 2.10 (1H, app. td, *J* 11.3, 5.5, 9-H_A), 1.89 (1H, dd, *J* 11.5, 8.7, 9-H_B), 1.15 (3H, t, *J* 7.3, NCH_2CH_3). **^{13}C NMR** (125 MHz, MeOD-d_4, 333 K): δ 194.9, 126.9, 119.9, 117.7, 113.8, 80.2, 62.5, 60.0, 54.5, 49.9, 47.6, 36.2, 31.7, 12.0. **IR** ν_{max}(film)/cm^{-1} 3197, 2967, 2937, 1667, 1516, 1451. **HRMS** (ESI): $C_{14}H_{20}N_3O$ [M+H]$^+$; calculated 246.1601, found 246.1602.

(1*R,8*R**,10*R**)-14-Methyl-2-oxo-5,12,14-triazatetracyclo[6.5.1.0^{1,10}.0^{3,7}]tetradeca-3,6-diene-12-carbaldehyde F47**

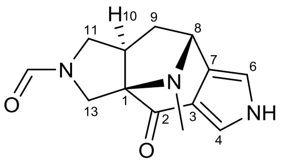

F47

To a solution of compound **F45** (100 mg, 0.46 mmol) in MeOH (0.5 mL) was added ethyl formate (3.0 mL) and the resulting solution was heated to 50 °C for 24 h, then concentrated *in vacuo* to give formamide **F47** (96 mg, 0.39 mmol, 85%) as a yellow oil. **^{1}H NMR** (500 MHz, MeOD-d_4, 333 K, two stable conformations observed at the pyrrolidine ring [50:50 mixture], NH not observed): δ 8.04-8.00 (1H, m, CHO), 7.31-7.26 (1H, m, 4-H), 6.57-6.53 (1H, m, 6-H), 4.28 (0.5H, d, *J* 9.1, 8-H), 4.27 (0.5H, d, *J* 9.1, 8-H), 4.23 (0.5H, d, *J* 12.1, 13-$H_{A\text{-conf1}}$), 4.21 (0.5H, d, *J* 13.1, 13-$H_{A\text{-conf2}}$), 3.93 (0.5H, dd, *J* 10.9, 9.0, 11-$H_{B\text{-conf1}}$), 3.74 (0.5H, *J* 12.1, 9.4, 11-$H_{B\text{-conf2}}$), 3.47 (0.5H, *J* 12.1, 13-$H_{B\text{-conf1}}$), 3.42 (0.5H, dd, *J* 10.9, 7.7, 11-$H_{A\text{-conf1}}$), 3.37 (1H, dd, *J* 12.1, 6.5, 11-$H_{A\text{-conf2}}$), 3.31 (0.5H, d, *J* 13.2, 13-$H_{B\text{-conf2}}$), 2.66-2.57 (1H, m, 10-H), 2.15 (1.5H, s, NCH_3), 2.14 (1.5H, s, NCH_3), 2.08-1.99 (1H, m, 9-H_A), 1.93 (0.5H, dd, *J* 11.9, 9.0, 9-$H_{B\text{-conf(1 or 2)}}$), 1.88 (0.5H, dd, *J* 12.1, 8.7, 9-$H_{B\text{-conf(1 or 2)}}$). **^{13}C NMR** (125 MHz, MeOD-d_4, 333 K,): δ 194.4, 194.2, 163.3, 163.0, 128.7, 128.6, 121.8, 121.7, 118.7, 118.6, 115.3, 115.2, 81.6, 81.3, 63.3, 63.1, 54.6, 51.1, 47.8, 47.6, 47.4, 44.8, 39.9, 38.7, 33.1, 33.0 [26 of 26 expected peaks observed]. **HRMS** (ESI): $C_{13}H_{15}N_3NaO_2$ [M+H]$^+$; calculated 268.1056, found 268.1054.

(1*R,8*R**,10*R**)-12-Acetyl-14-methyl-5,12,14-triazatetracyclo[6.5.1.0^{1,10}.0^{3,7}]tetradeca-3,6-dien-2-one F48**

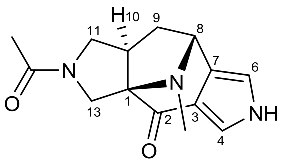

F48 ≡ 25

N-Acylation was carried out by following general procedure **E**, using compound **F45** (155 mg, 0.71 mmol). CH_2Cl_2 (10 mL) and H_2O (10 mL) were added and the phases separated. The aqueous layer was extracted with CH_2Cl_2 (2 x 10 mL) and the combined organic layers were dried over $MgSO_4$, filtered and concentrated *in vacuo* to give a crude brown oil. Flash chromatography eluting with 80:10:1 CH_2Cl_2–EtOH–NH_4OH gave the *title compound* **F48** (172 mg, 0.66 mmol, 93%) as a brown oil. **^{1}H NMR** (400 MHz, MeOD-d_4, 333 K, two stable conformations observed at the pyrrolidine ring

[50:50 mixture], NH not observed): δ 7.44-7.37 (1H, m, 4-H), 6.70-6.60 (1H, m, 6-H), 4.45 (0.5H, d, *J* 12.0, 13-$H_{A\text{-}conf1}$), 4.41 (0.5H, app. s, 8-H), 4.39 (0.5H, app. s, 8-H), 4.32 (0.5H, d, *J* 13.4, 13-$H_{A\text{-}conf2}$), 4.11-4.00 (1H, m, 11-H_B), 3.57-3.47 (1.5H, m, 11-H_A and 13-H_B), 3.41 (0.5H, dd, *J* 12.0, 7.7, 11-H_A), 2.83 (0.5H, ddd, *J* 17.0, 9.0, 4.6, 10-H), 2.72 (0.5H, ddd, *J* 16.9, 8.9, 4.7, 10-H), 2.28 (1.5H, s, NCH_3), 2.26 (1.5H, s, NCH_3), 2.26-2.13 (1H, m, 9-H_A), 2.12 (1.5H, s, $N(CO)CH_3$), 2.07 (1.5H, s, $N(CO)CH_3$), 2.01 (1H, dd, *J* 12.1, 8.8, 9-H_B). **^{13}C NMR** (100 MHz, MeOD-d_4, 333 K,): δ 192.6, 192.5, 175.0, 170.5, 127.4, 120.6 (2 peaks), 116.9, 113.8 (2 peaks), 81.3, 79.8, 61.7, 61.6, 55.0, 54.5, 47.5, 46.4, 45.7, 45.1, 38.3, 38.2, 31.7 (2 peaks), 20.7, 20.6 [26 of 28 expected peaks observed]. **HRMS** (ESI): $C_{14}H_{18}N_3O_2$ [M+H]$^+$; calculated 260.1394, found 260.1394.

(1*R,8*R**,10*R**)-14-Methyl-2-oxo-5,12,14-triazatetracyclo[6.5.1.0^{1,10}.0^{3,7}]tetradeca-3,6-diene-12-carboxamide F49**

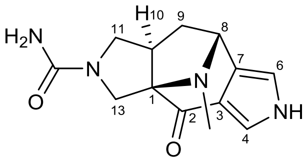

F49

To a solution of compound **F45** (128 mg, 0.59 mmol, 1.00 eq.) in 1:1 dioxane–H_2O (5.0 mL) was added AcOH (0.10 mL, 1.77 mmol, 3.00 eq.) followed by KNCO (72 mg, 0.88 mmol, 1.50 eq.) and the resulting solution was stirred at 80 °C for 24 h. H_2O (5 mL) and EtOAc (5 mL) were added and the phases separated. The aqueous phase was extracted with EtOAc (2 x 5 mL) and the combined organic layers were dried over $MgSO_4$, filtered and concentrated *in vacuo* to give the *title compound* **F49** (106 mg, 0.41 mmol, 69%) as a yellow oil. **^{1}H NMR** (500 MHz, MeOD-d_4, 333 K, pyrrole NH and NH_2 not observed): δ 7.42 (1H, d, *J* 1.3, 4-H), 6.68 (1H, d, *J* 1.3, 6-H), 4.41 (1H, d, *J* 5.9, 8-H), 4.30 (1H, d, *J* 11.7, 13-H_A), 3.94 (1H, app. t, *J* 9.8, 11-H_A), 3.43-3.31 (2H, m, 11-H_B and 13-H_B), 2.83-2.71 (1H, m, 10-H), 2.27 (3H, s, NCH_3), 2.25-2.15 (1H, m, 9-H_A), 2.01 (1H, dd, *J* 12.0, 8.8, 9-H_B). **^{13}C NMR** (125 MHz, MeOD-d_4, 333 K): δ 192.9, 159.1, 127.4, 121.5, 117.0, 113.8, 81.0, 61.7, 53.5, 46.1, 38.1, 31.7, 21.4. **IR** ν_{max}(film)/cm^{-1} 3357, 2922, 2852, 1631, 1588, 1458.

5.4.3.21 Preparation of fragments derived from scaffold S32

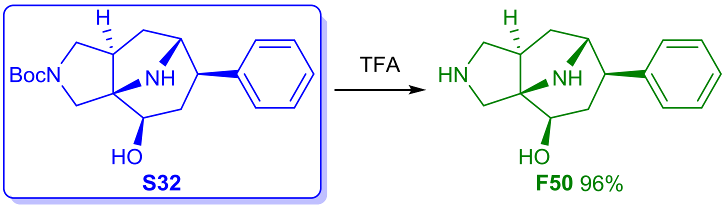

(1*R**,5*R**,7*R**,8*R**,10*R**)-8-Phenyl-3,11-diazatricyclo[5.3.1.$0^{1,5}$]undecan-10-ol F50

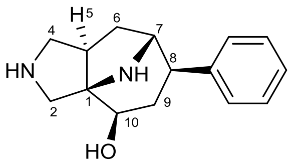

F50

Deprotection of Boc-protected compound **S32** (40 mg, 0.12 mmol) was carried out by following general procedure **I**. Purification by SCX SPE eluting with MeOH then sat. NH_3/MeOH gave the *title compound* **F50** (27 mg, 0.11 mmol, 96%) as a brown oil. **^{1}H NMR** (500 MHz, MeOD-d_4, 333 K, OH and 2 × NH not observed): δ 7.62 (2H, d, *J* 7.6, Ar-H), 7.29 (2H, t, 7.6, Ar-H), 7.16 (1H, t, *J* 7.6, Ar-H), 3.78 (1H, d, *J* 4.9, 10-H), 3.70 (1H, d, *J* 6.4, 7-H), 3.13 (1H, dd, *J* 11.5, 8.8, 4-H_B), 2.93 (1H, d, *J* 12.2, 2-H_A), 2.82 (1H, d, *J* 8.1, 8-H), 2.77 (1H, d, *J* 12.2, 2-H_B), 2.75 (1H, dd, *J* 11.5, 5.9, 4-H_A), 2.50-2.39 (1H, m, 5-H), 2.35 (1H, ddd, *J* 15.6, 8.3, 4.8, 9-H_A), 2.17 (1H, dd, *J* 12.9, 9.0, 6-H_A), 2.07 (1H, dd, *J* 15.6, 1.2, 9-H_B), 1.94-1.85 (1H, m, 6-H_B). **^{13}C NMR** (125 MHz, MeOD-d_4, 333 K): δ 146.8, 129.3, 129.3, 126.8, 77.4, 71.0, 65.1, 56.3, 55.7, 46.5, 44.7, 38.6, 32.3. **IR** ν_{max}(film)/cm^{-1} 3389, 1460, 1416. **HRMS** (ESI): $C_{15}H_{20}N_2O$ $[M+H]^+$; calculated 245.1654, found 245.1650.

5.4.3.22 Preparation of fragments derived from scaffold S33 and S34

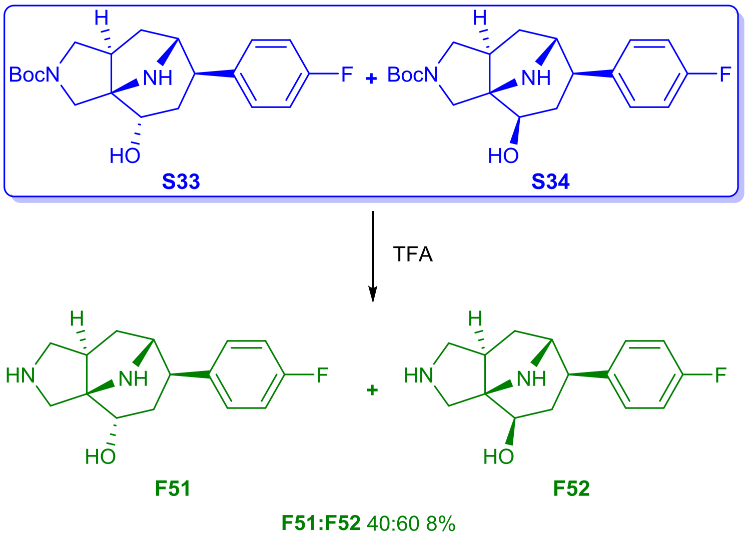

(1*R,5*R**,7*R**,8*R**,10*S**)-8-(4-Fluorophenyl)-3,11-diazatricyclo[5.3.1.0^{1,5}]undecan-10-ol F51 and (1*R**,5*R**,7*R**,8*R**,10*R**)-8-(4-fluorophenyl)-3,11-diazatricyclo[5.3.1.0^{1,5}]undecan-10-ol F52**

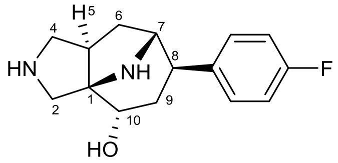

F51

+

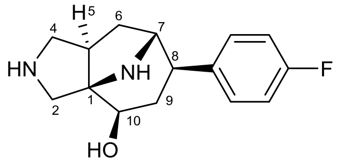

F52

Deprotection of Boc-protected compounds **S33** and **S34** (119 mg, 0.46 mmol) was carried out by following general procedure **I**. Purification by SCX eluting with MeOH then sat. NH_3/MeOH gave the *title compounds* **F51** and **F52** (10 mg, 38 μmol, 8%, 40:60 mixture of diastereomers) as a brown oil. **^{1}H NMR** (400 MHz, MeOD-d_4, major and minor assigned by analogy to compounds **F51** and **F52**): δ 7.61-7.55 (1.2H, m, major Ar 3-H), 7.31-7.23 (0.8H, m, minor Ar 3-H), 7.02-6.90 (2H, m, major and minor, Ar 2-H), 4.01 (0.4H, dd, *J* 10.8, 5.8, minor, 10-H), 3.86 (0.6H, d, *J* 3.7, major, 10-H), 3.73 (0.6H, d, *J* 6.9, major, 7-H), 3.65 (0.4H, d, *J* 6.7, minor 7-H), 3.52 (0.6H, dd, *J* 11.9, 9.5, major 4-H_B), 3.45 (0.4H, dd, *J* 11.8, 9.5, minor, 4-H_B), 3.41 (0.4H, d, *J* 11.9, minor 2-H_A), 3.29 (0.6H, d, *J* 12.4, major, 2-H_A), 3.13 (0.6H, dd, *J* 11.9, 6.6, major, 4-H_A), 3.10 (0.6H, d, *J* 12.4, major, 2-H_B), 3.08 (0.4H, d, *J* 11.9, minor, 2-H_B), 3.05 (0.4H, dd, *J* 11.8, 7.2, minor, 4-H_A), 2.98 (0.4H, d, *J* 7.0, minor, 8-H), 2.94-2.82 (0.4H, m, minor, 5-H), 2.86 (0.6H, d, *J* 8.0, major, 8-H), 2.79-2.65 (0.6H, m, major, 5-H), 2.29 (0.6H, ddd, *J* 13.4, 8.3, 4.7, major, 9-H_B), 2.27-2.21 (0.4H, m, minor, 9-H_B), 2.20 (0.6H, dd, *J* 13.6, 9.1, major, 6-H_B), 2.10 (0.4H, dd, *J* 13.4, 8.9, minor, 6-H_B), 2.04-1.97 (0.6H, m, major, 6-H_A), 2.03 (0.6H, ddd, *J* 13.4, 6.9, 4.7, major, 9-H_A), 1.96 (0.4H, ddd, *J* 13.3, 6.8, 4.4, minor, 6-H_A), 1.78 (0.4H, ddd, *J* 14.5, 10.9, 7.2, minor, 9-H_A). **^{13}C NMR** (100 MHz, MeOD-d_4): δ 161.5 (d, *J* 243), 161.4 (d, *J* 244), 139.7, 138.8, 130.0 (d, *J* 7.9), 129.3 (d, *J* 7.7), 114.8 (d, *J* 21), 114.4 (d, *J* 21), 74.8, 74.6, 72.2, 67.5, 65.2, 63.6, 62.9, 52.4, 52.3, 49.4, 45.2, 42.1, 41.7, 38.3, 36.4, 35.7, 31.5, 29.2 [26 of 26 expected peaks observed]. **IR** ν_{max}(film)/cm^{-1} 3389, 1482, 1447. **HRMS** (ESI): $C_{15}H_{19}FN_2NaO$ [M+Na]$^+$; calculated 285.1374, found 285.1370.

6.0 High-throughput protein crystallography

Proteins were expressed, purified, and crystallised as previously described for JMJD2D,[S11] and the ATAD2 and BRD1 bromodomains.[S12] Crystal soaking was performed using the XChem platform.[S13] X-ray diffraction data were collected at Diamond Light Source beamline I04-1 and processed through the Diamond autoprocessing pipeline. Electron density maps were generated in batch by *DIMPLE*[S14] using *XChemExplorer*.[S15] Ligand restraints were generated with *ACEDRG or Grade*[S16] and ligand binding was detected with *PanDDA,*[S17] with ligands built into *PanDDA* event maps. Fragments based on natural product paralogues were screened as racemates. Both enantiomers were fitted to the electron density in the *PanDDA* event maps using *COOT*[S18], then the enantiomer with the best fit was chosen. Iterative refinement and manual model correction was performed using *REFMAC*[S19] and *COOT*, respectively.

7.0 Processed NMR spectra

Compounds are listed in order of appearance within in the Supplementary Information. NOESY spectra are also included in this Section.

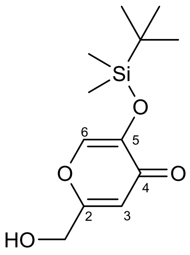

S1

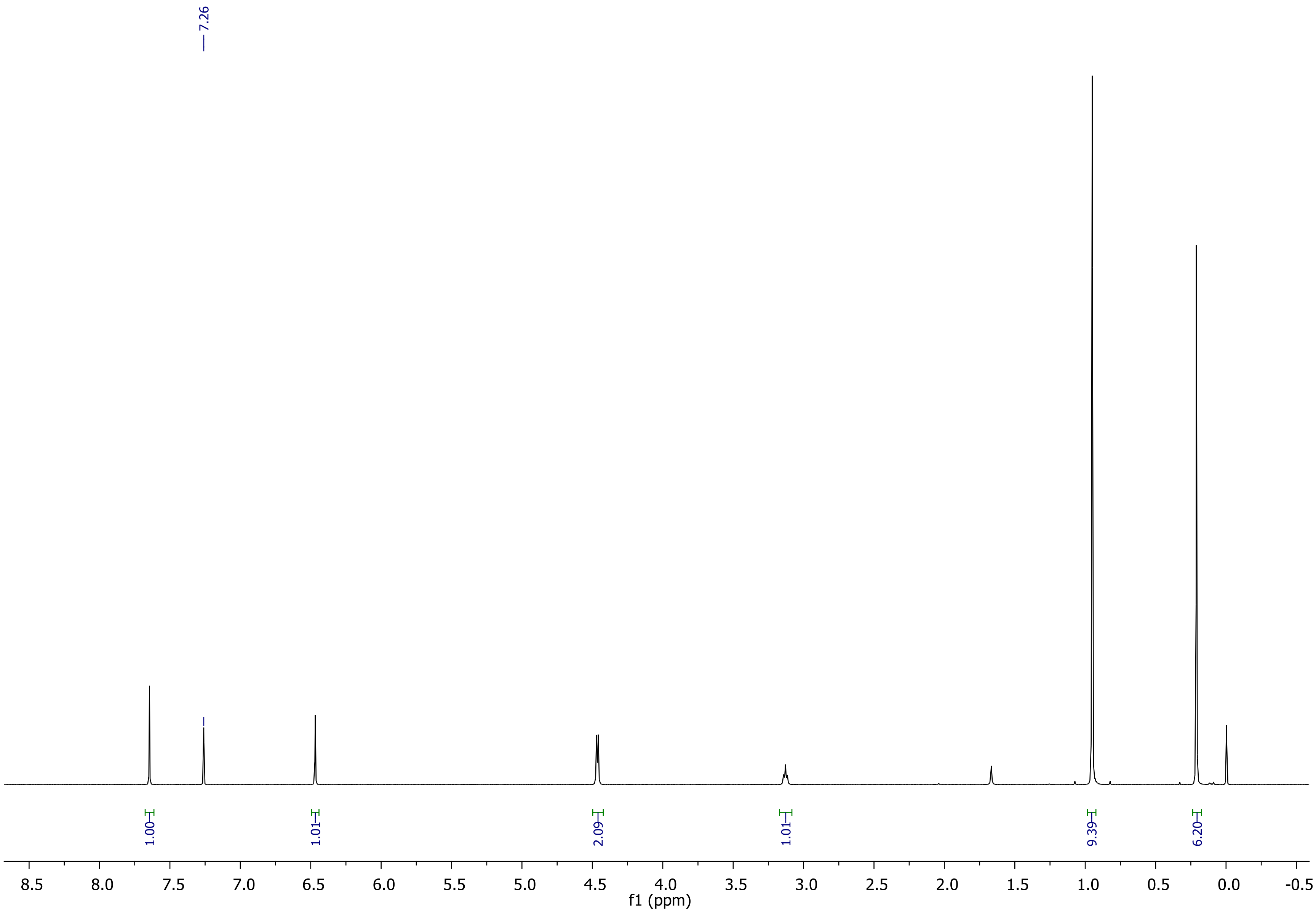

7.26
1.00
1.01
2.09
1.01
9.39
6.20
8.5
8.0
7.5
7.0
6.5
6.0
5.5
5.0
4.5
4.0
3.5
3.0
2.5
2.0
1.5
1.0
0.5
0.0
-0.5
f1 (ppm)

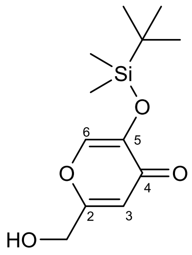
Si
O
6
5
O
O
4
2
3
HO

S1

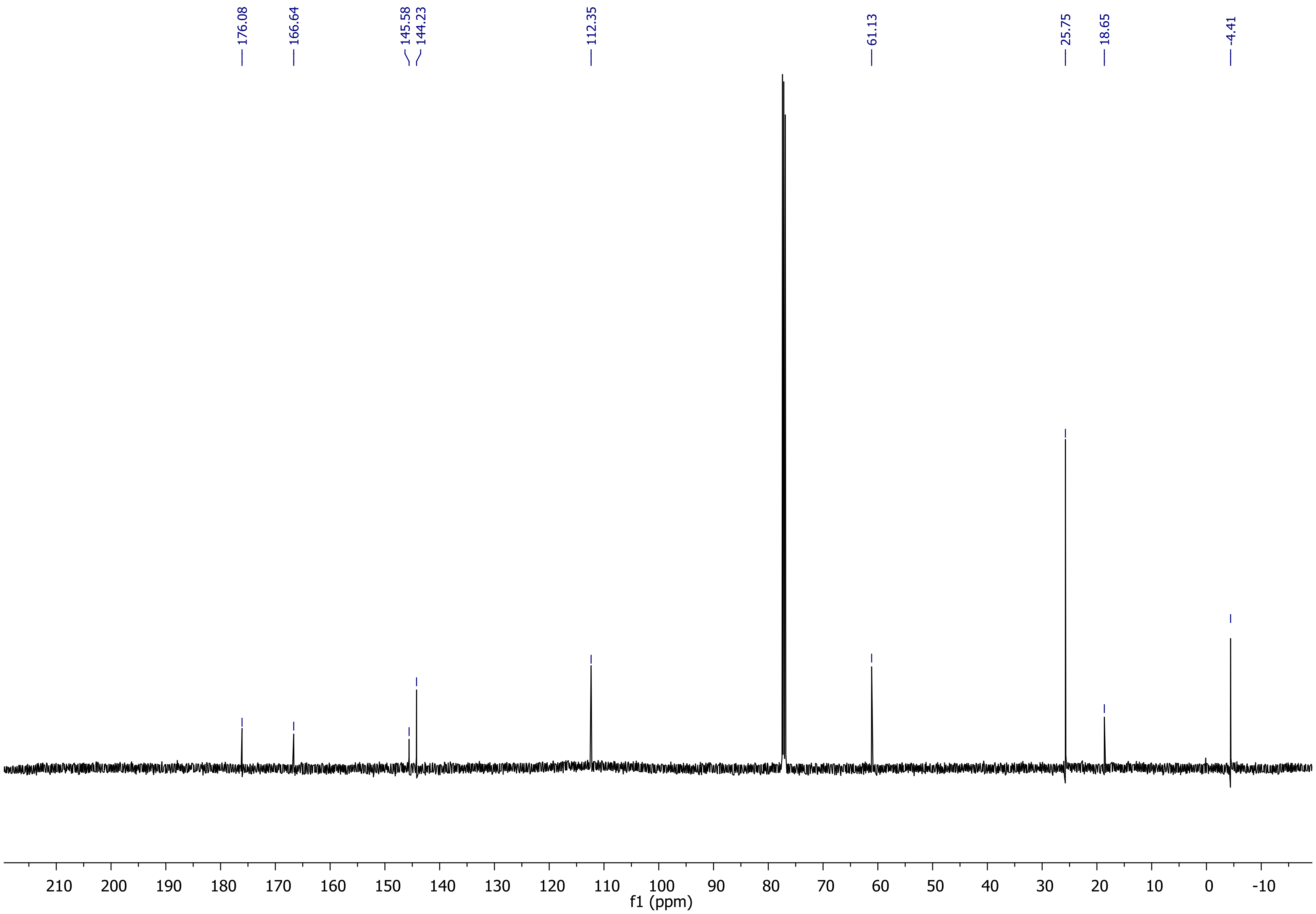
176.08
166.64
145.58
144.23
112.35
61.13
25.75
18.65
-4.41
210
200
190
180
170
160
150
140
130
120
110
100
90
80
70
60
50
40
30
20
10
0
-10
f1 (ppm)

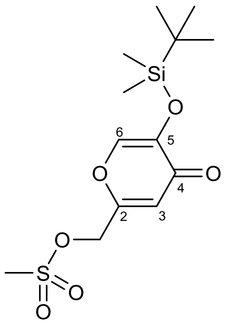

S2

carried forward crude

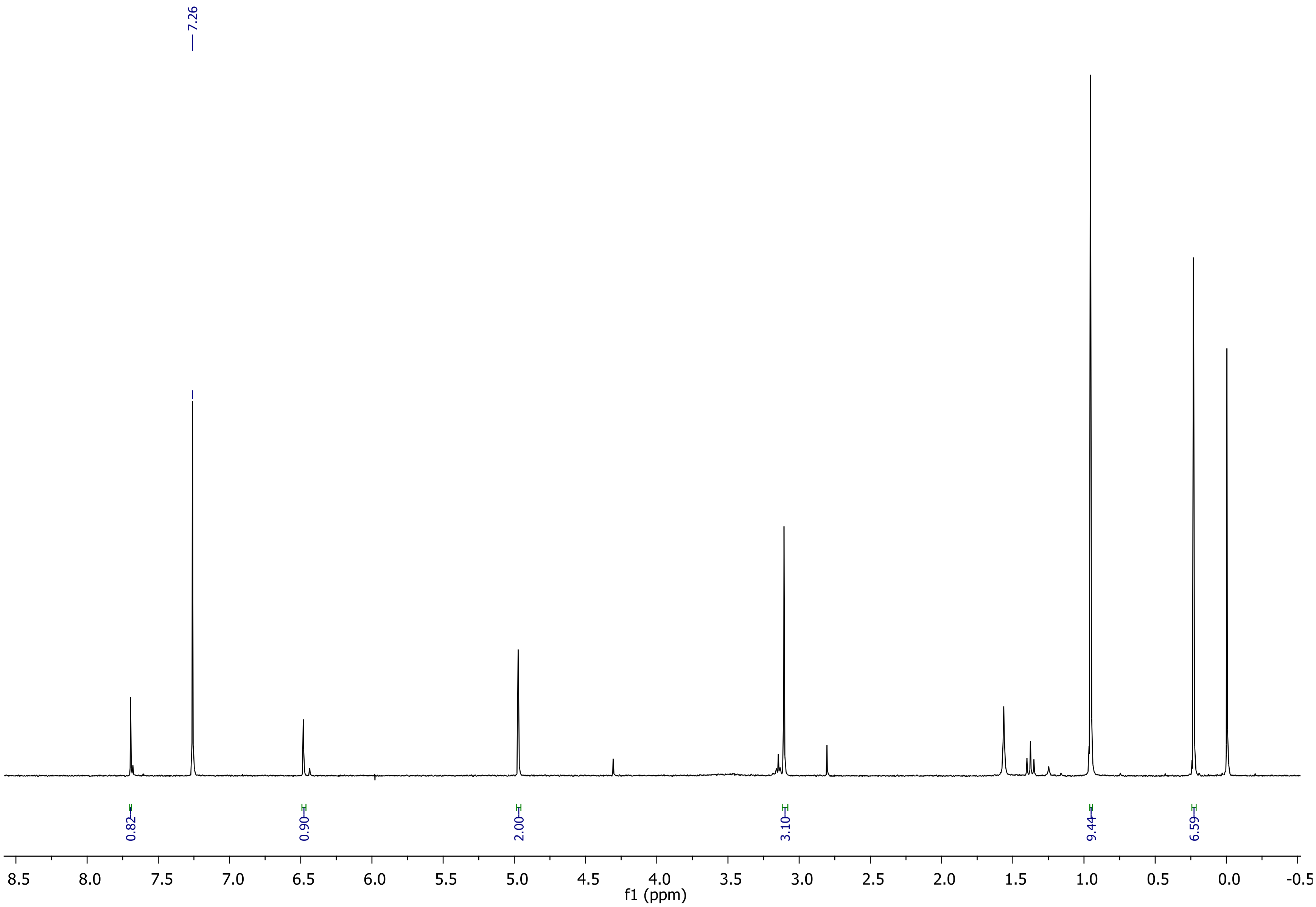

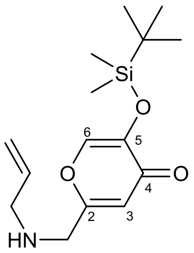
Si
O
O
O
HN
6
5
4
3
2

S3

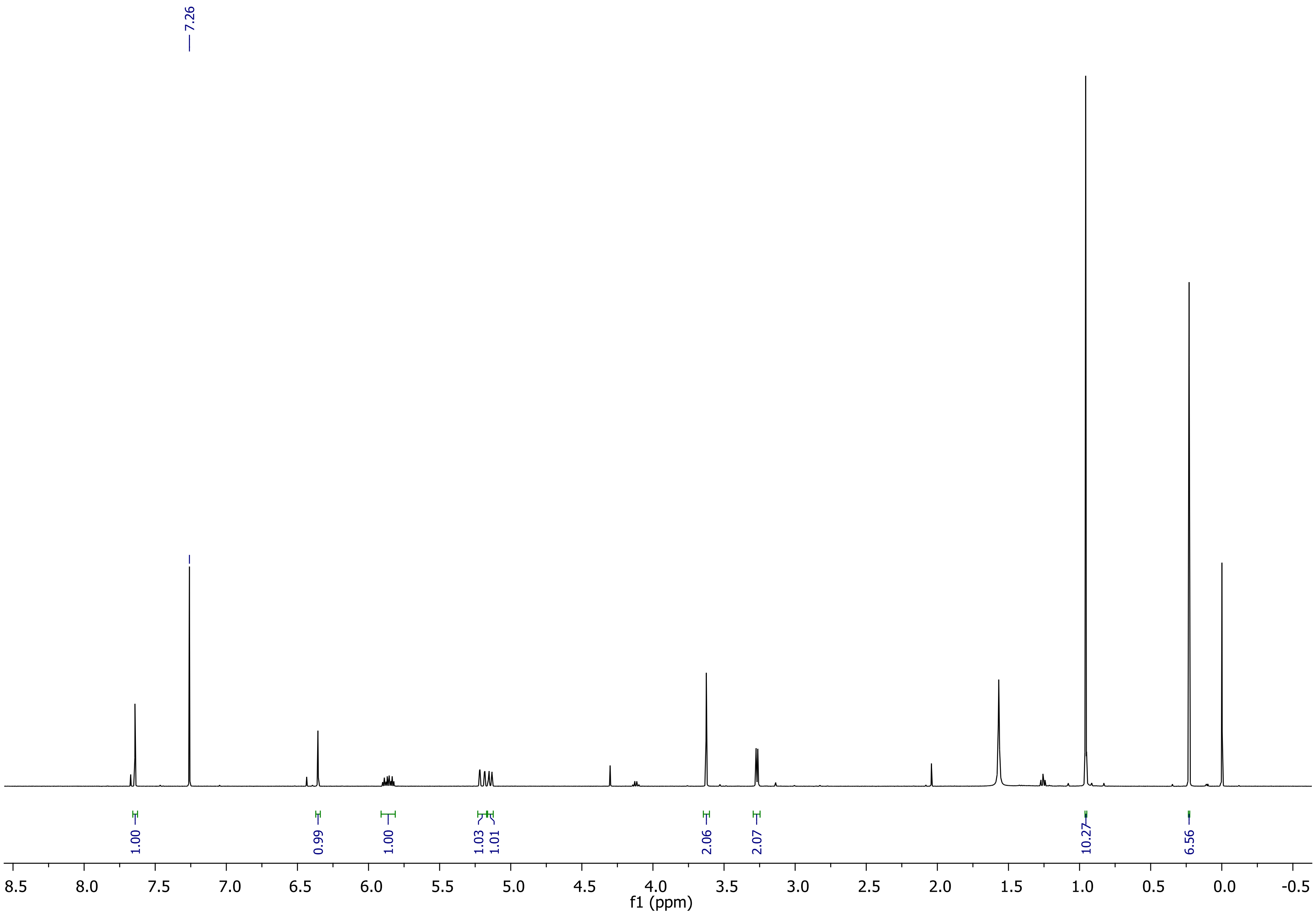
7.26
1.00
0.99
1.00
1.03
1.01
2.06
2.07
10.27
6.56
8.5 8.0 7.5 7.0 6.5 6.0 5.5 5.0 4.5 4.0 3.5 3.0 2.5 2.0 1.5 1.0 0.5 0.0 -0.5
f1 (ppm)

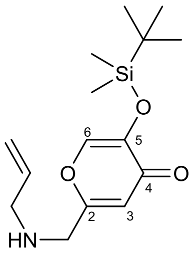

S3

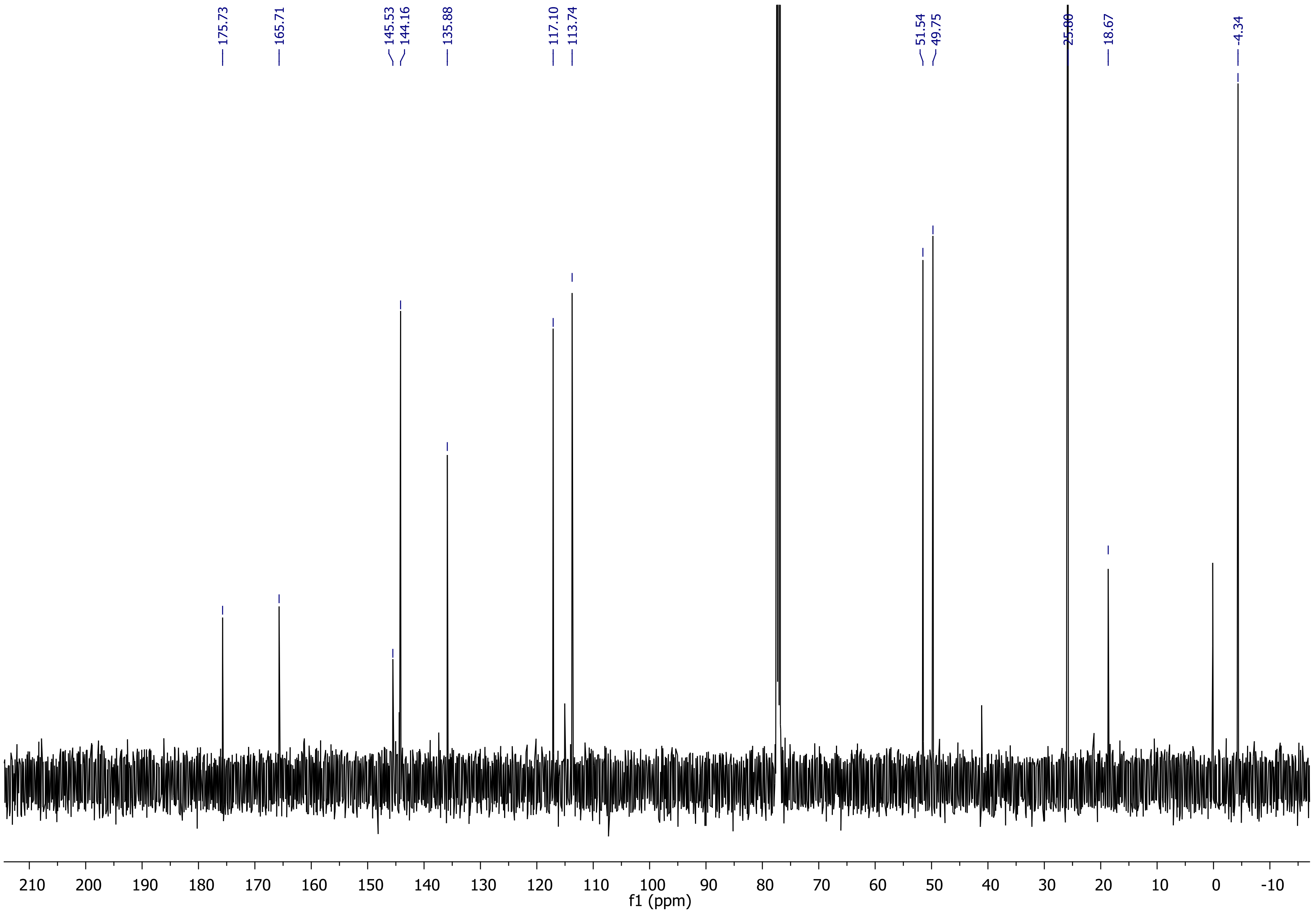
175.73
165.71
145.53
144.16
135.88
117.10
113.74
51.54
49.75
25.80
18.67
-4.34
210 200 190 180 170 160 150 140 130 120 110 100 90 80 70 60 50 40 30 20 10 0 -10
f1 (ppm)

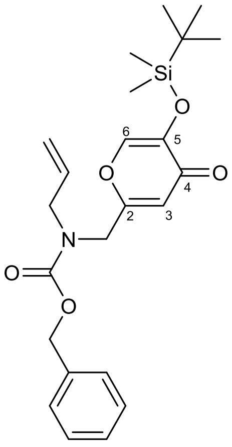
Si
O
O
O
N
O
O
6
5
4
3
2

S4

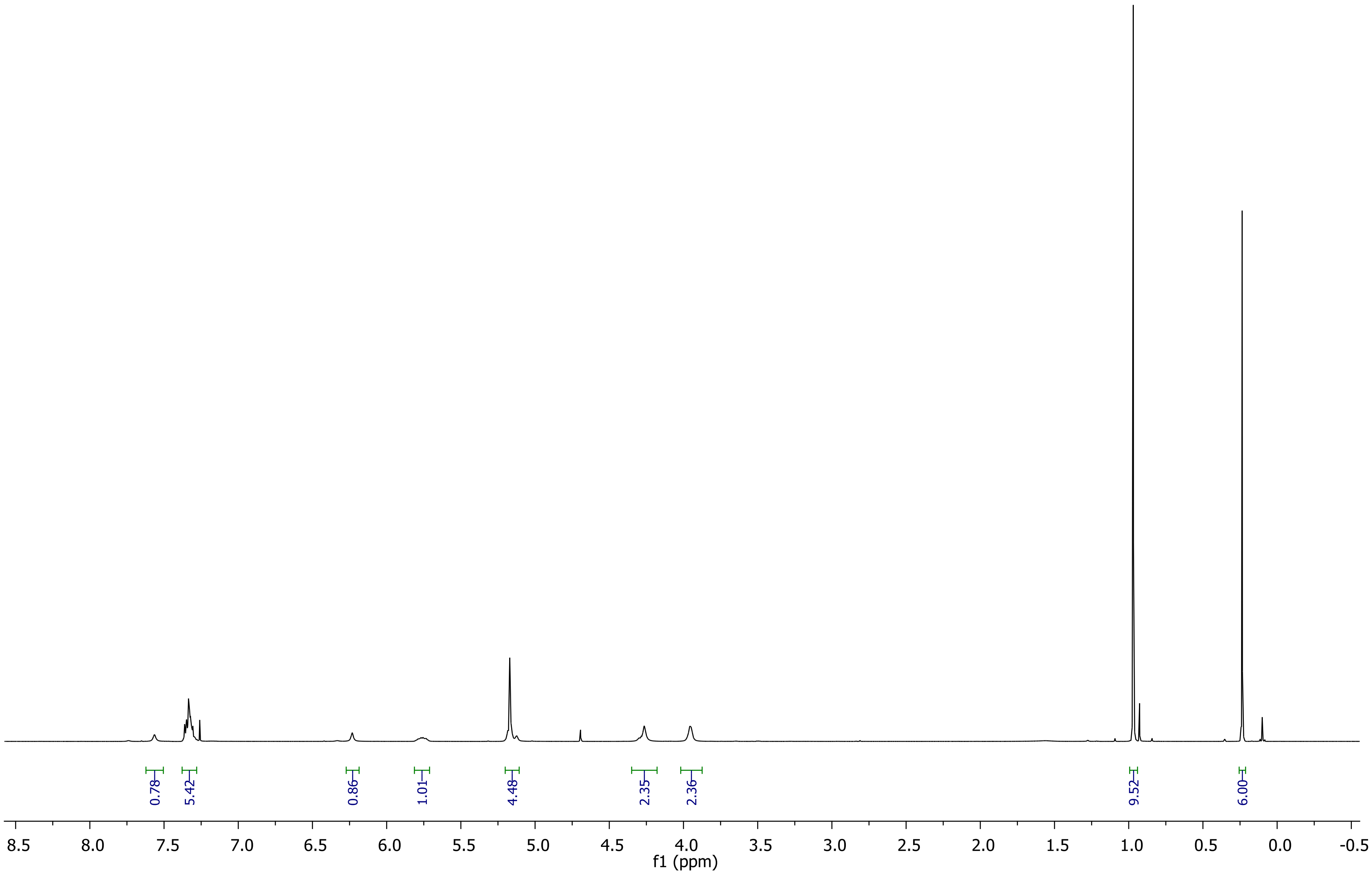
0.78
5.42
0.86
1.01
4.48
2.35
2.36
9.52
6.00
8.5
8.0
7.5
7.0
6.5
6.0
5.5
5.0
4.5
4.0
3.5
3.0
2.5
2.0
1.5
1.0
0.5
0.0
-0.5
f1 (ppm)

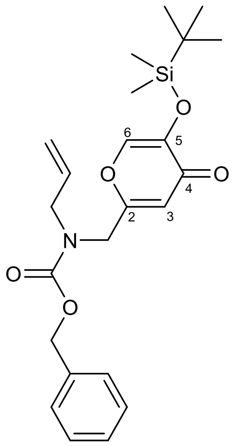

Si
O
O
O
6
5
4
3
2
N
O
O

S4

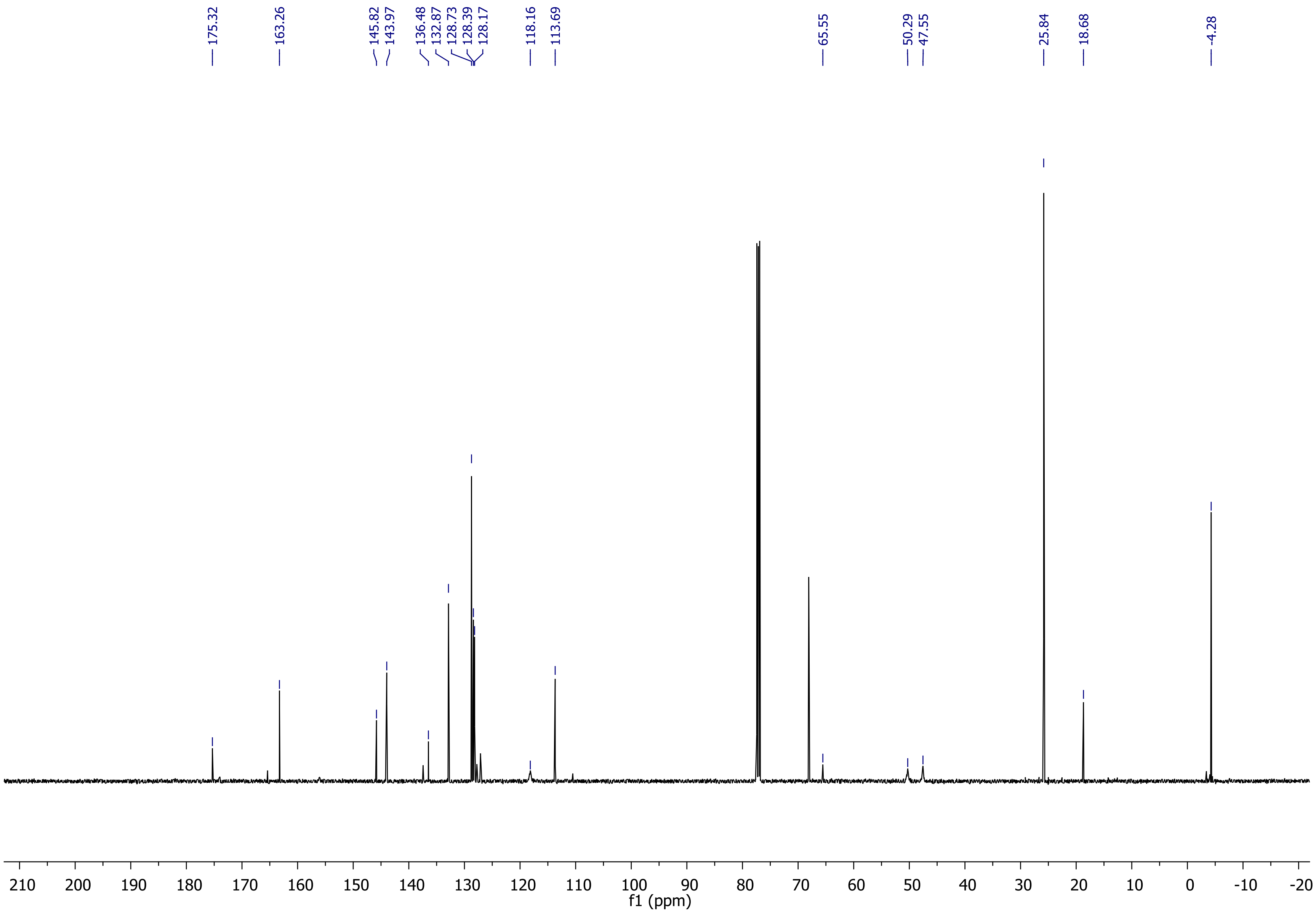

175.32
163.26
145.82
143.97
136.48
132.87
128.73
128.39
128.17
118.16
113.69
65.55
50.29
47.55
25.84
18.68
-4.28
210 200 190 180 170 160 150 140 130 120 110 100 90 80 70 60 50 40 30 20 10 0 -10 -20
f1 (ppm)

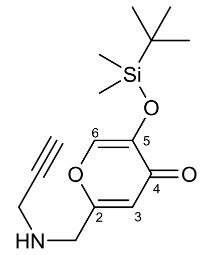

S5

carried forward crude

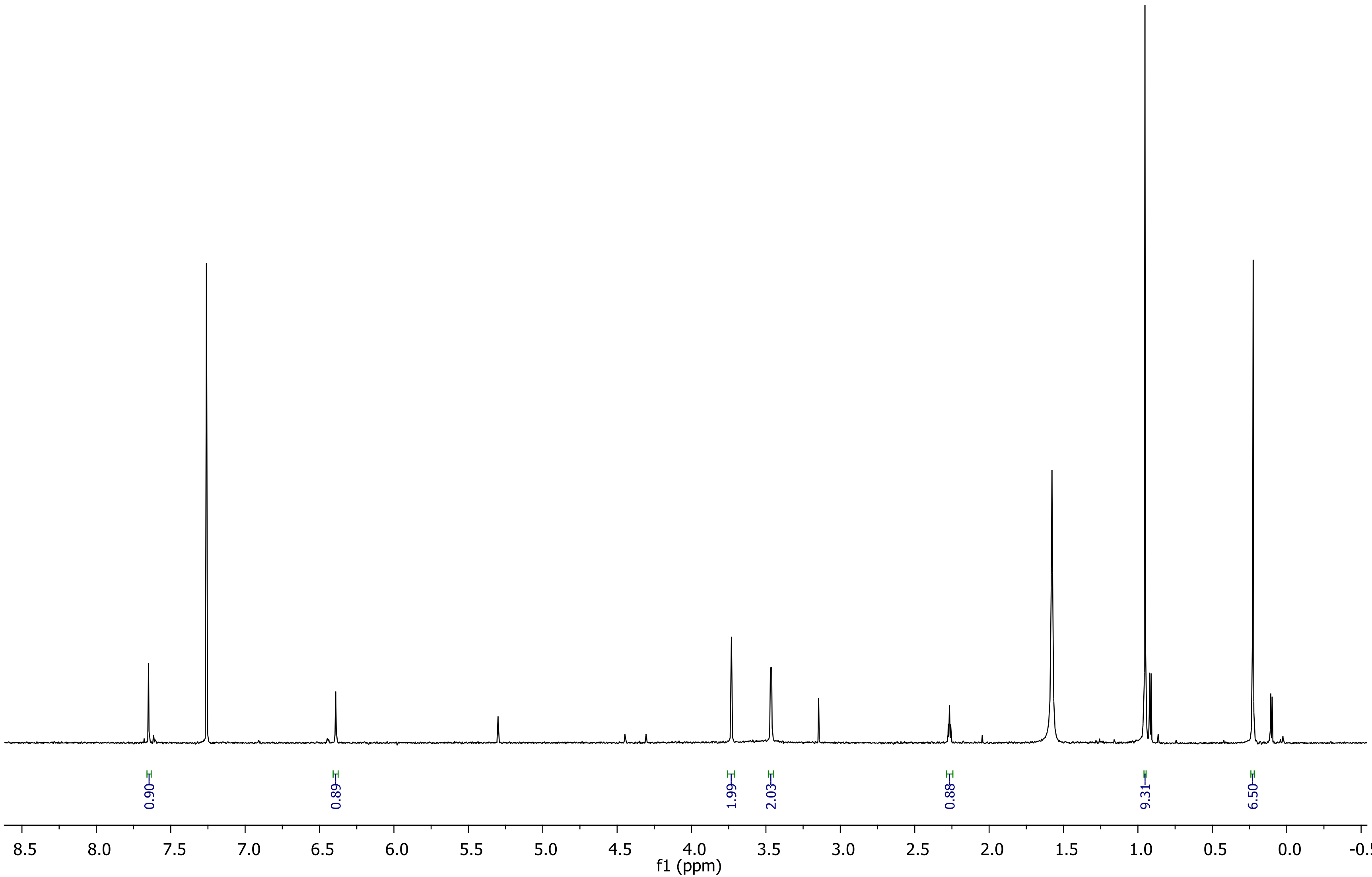

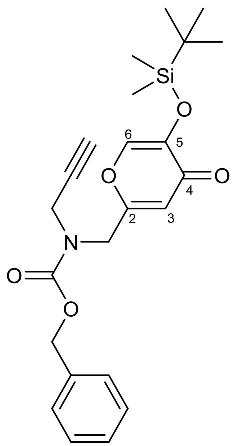

Si
O
O
6
5
4
3
2
O
N
O
O

S6

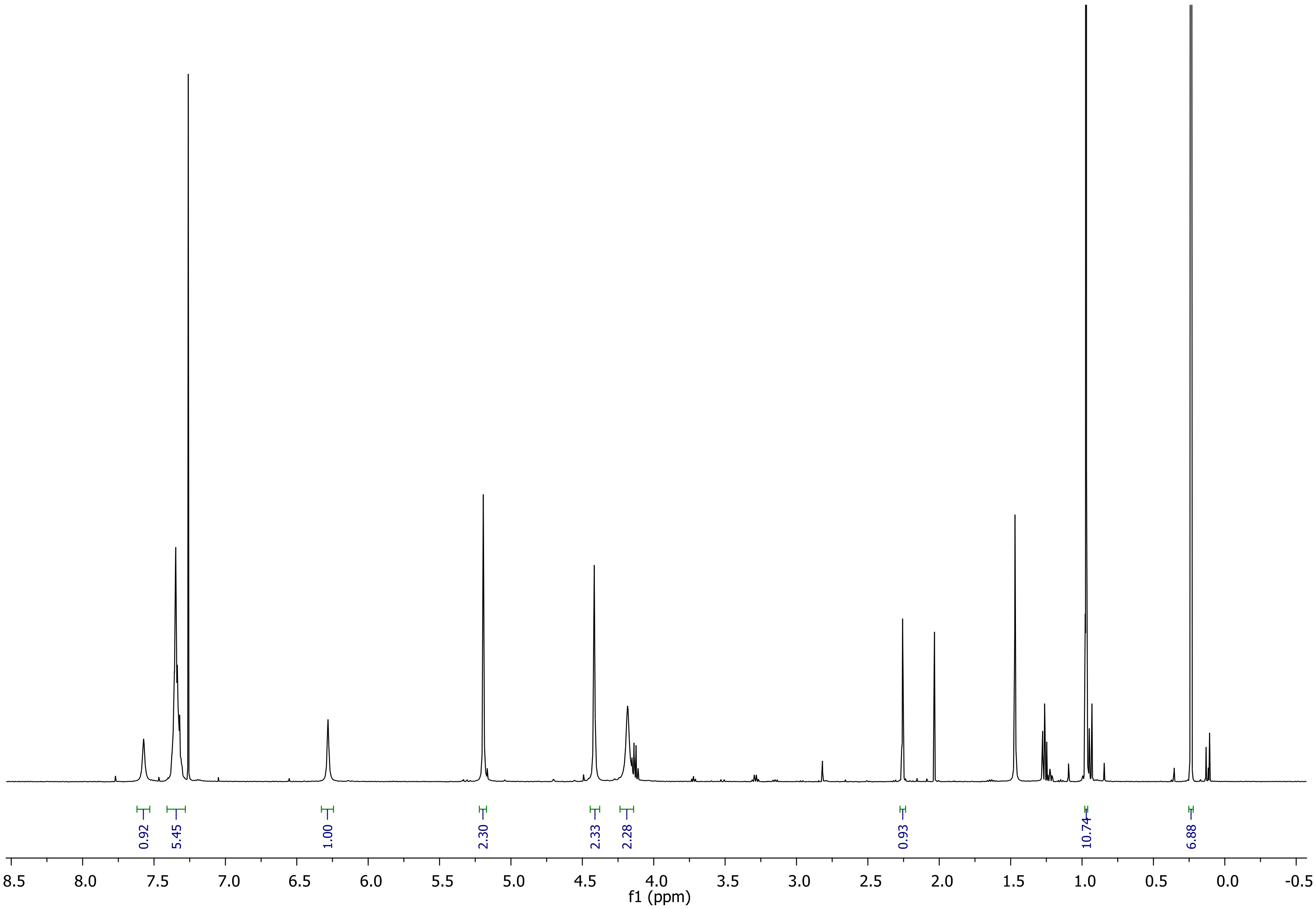

0.92
5.45
1.00
2.30
2.33
2.28
0.93
10.74
6.88
8.5
8.0
7.5
7.0
6.5
6.0
5.5
5.0
4.5
4.0
3.5
3.0
2.5
2.0
1.5
1.0
0.5
0.0
-0.5
f1 (ppm)

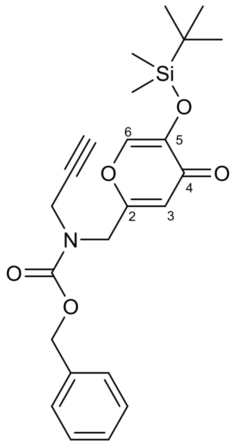

Si
O
O
O
6
5
4
3
2
N
O
O

S6

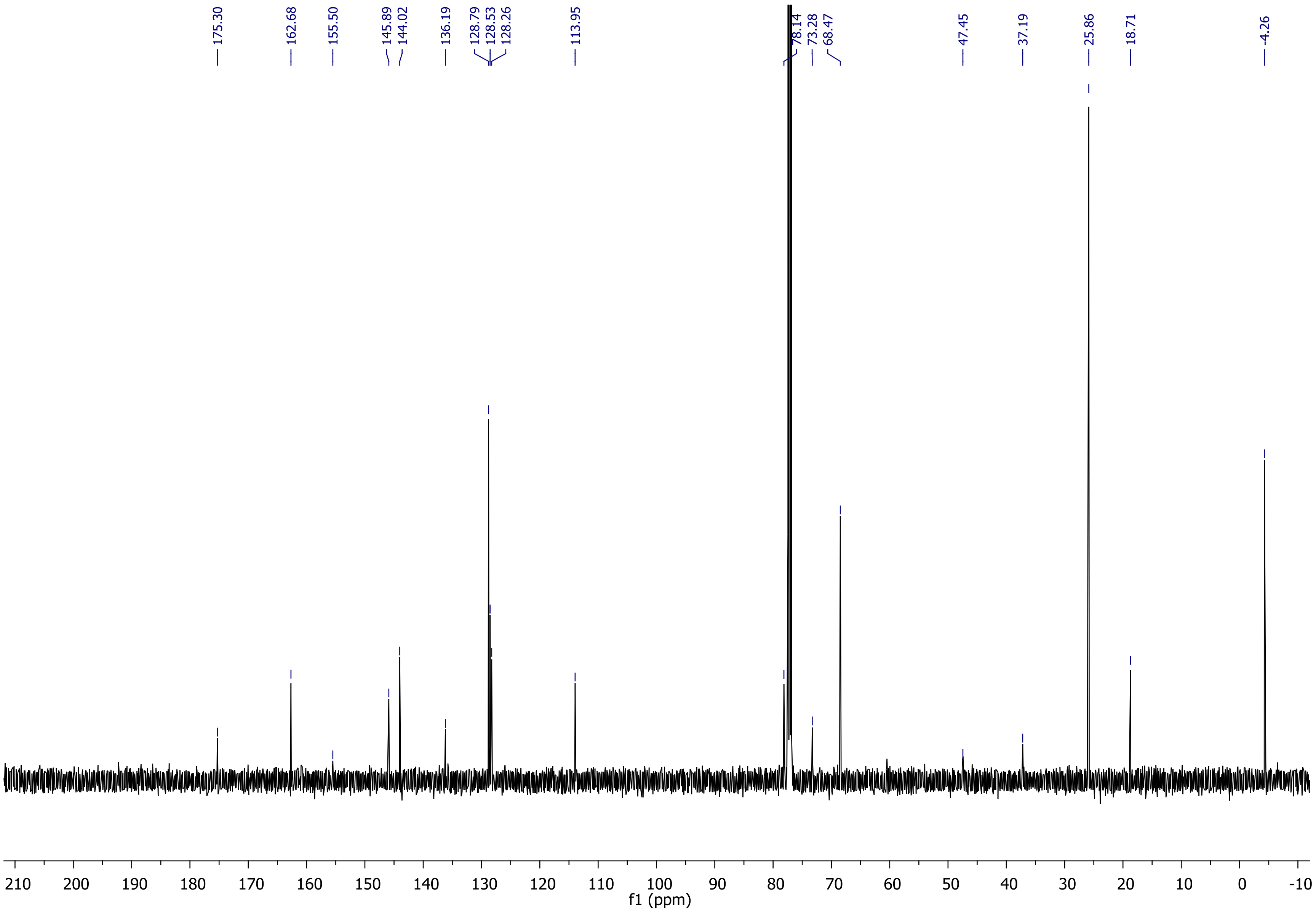

175.30
162.68
155.50
145.89
144.02
136.19
128.79
128.53
128.26
113.95
78.14
73.28
68.47
47.45
37.19
25.86
18.71
-4.26
210
200
190
180
170
160
150
140
130
120
110
100
90
80
70
60
50
40
30
20
10
0
-10
f1 (ppm)

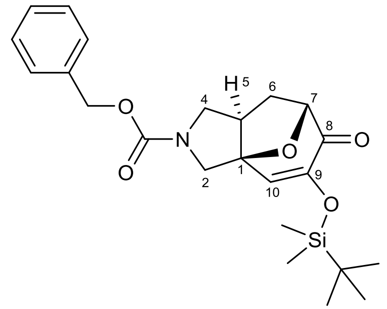

O
N
O
H 5
4
6
7
8
O
O
1
2
10
9
O
Si

2a

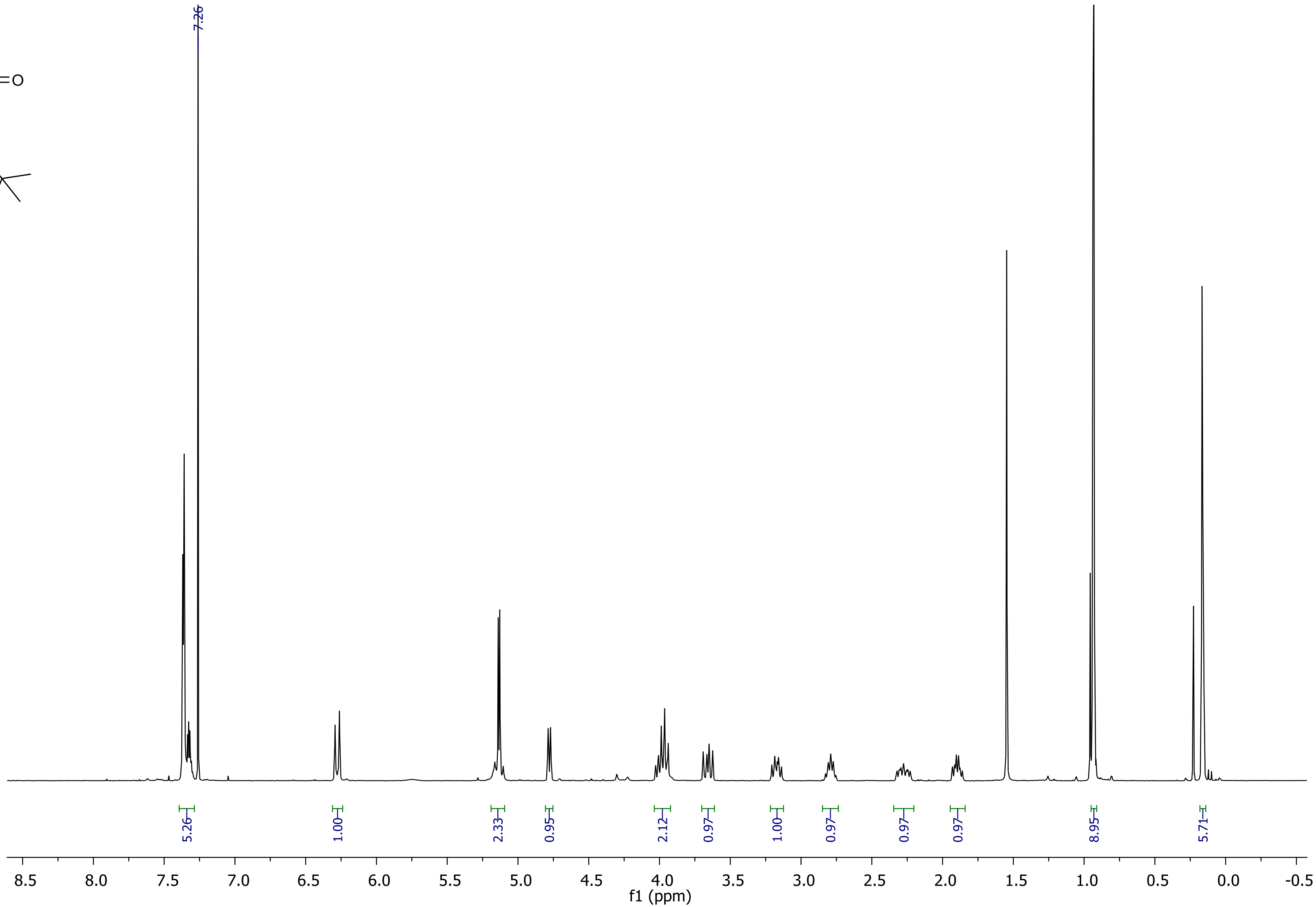

7.26
5.26
1.00
2.33
0.95
2.12
0.97
1.00
0.97
0.97
0.97
8.95
5.71
8.5
8.0
7.5
7.0
6.5
6.0
5.5
5.0
4.5
4.0
3.5
3.0
2.5
2.0
1.5
1.0
0.5
0.0
-0.5
f1 (ppm)

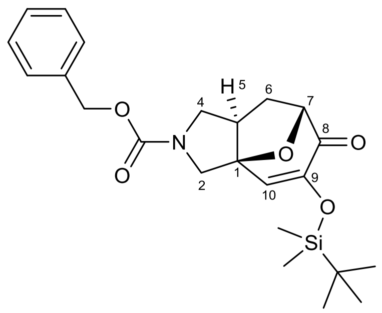

2a

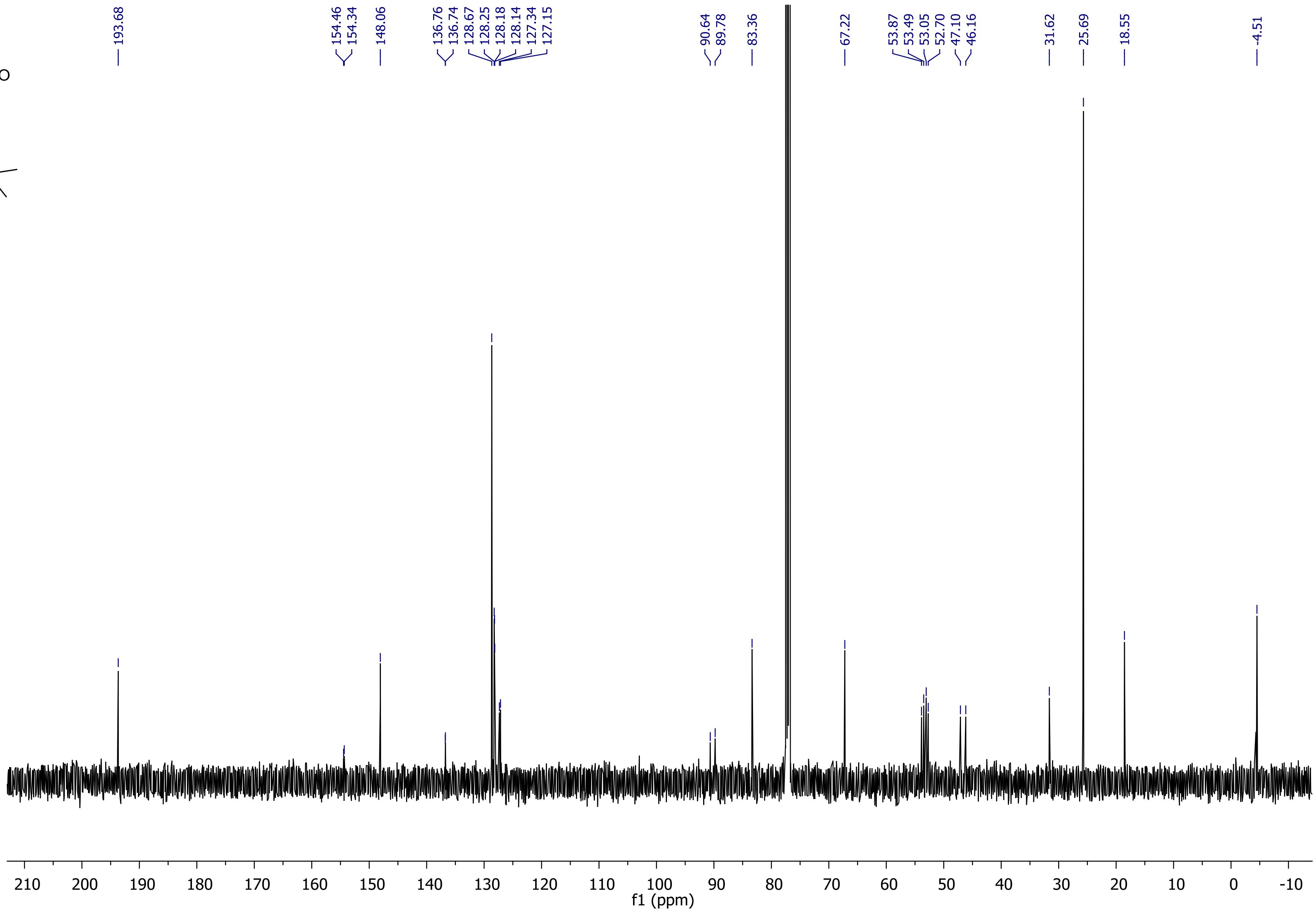
193.68
154.46
154.34
148.06
136.76
136.74
128.67
128.25
128.18
128.14
127.34
127.15
90.64
89.78
83.36
67.22
53.87
53.49
53.05
52.70
47.10
46.16
31.62
25.69
18.55
-4.51
210 200 190 180 170 160 150 140 130 120 110 100 90 80 70 60 50 40 30 20 10 0 -10
f1 (ppm)

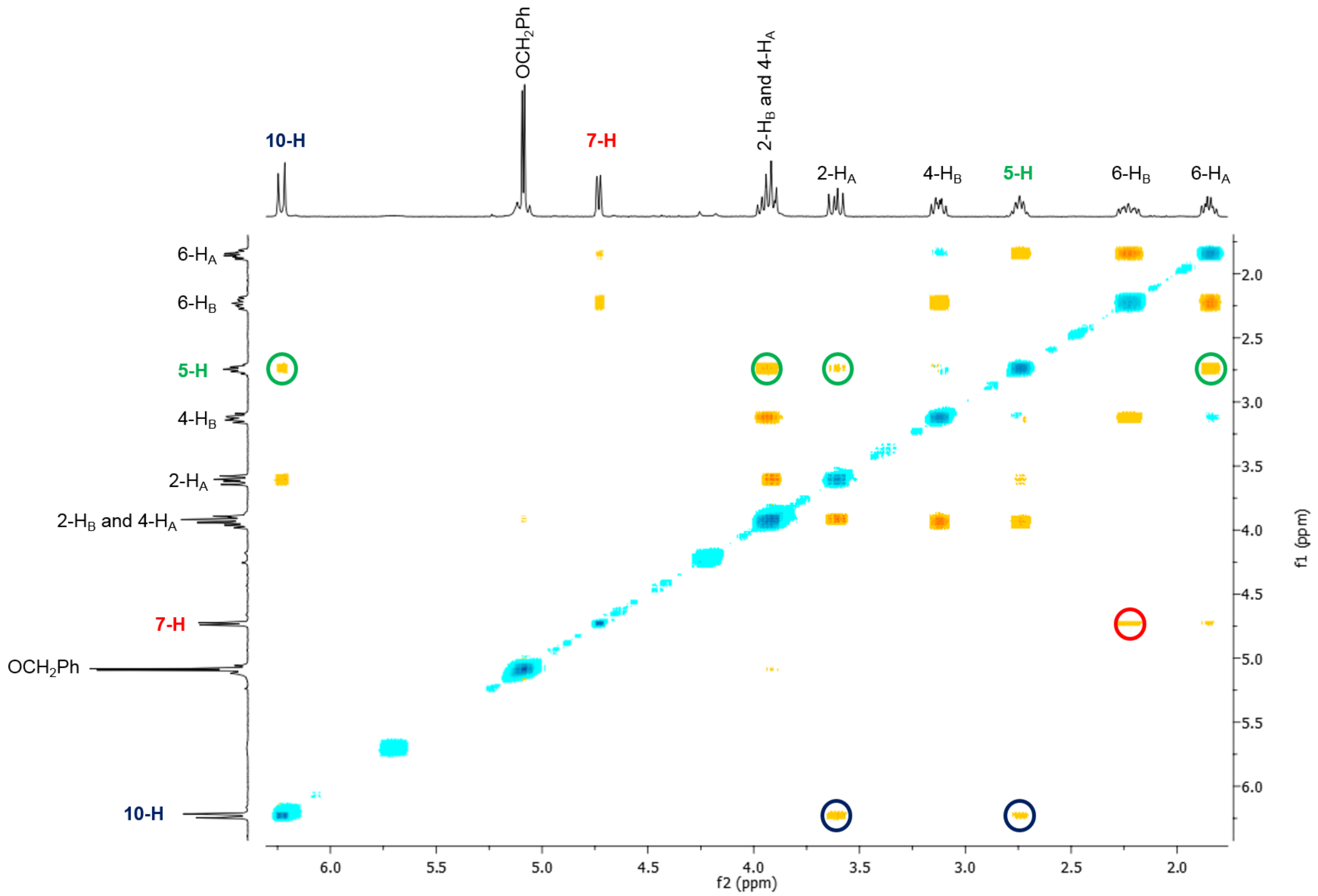

2a NOESY correlations:

5-H: 2-H_A; 4-H_A; 6-H_A; 10-H

7-H: 6-H_B

10-H: 2-H_A; 5-H

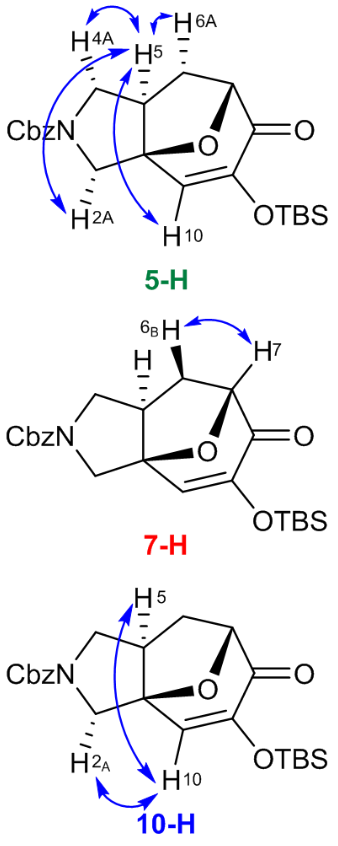

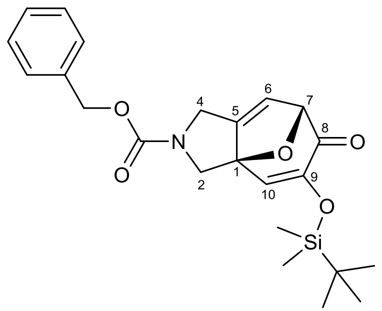

O
N
O
O
O
O
Si
1
2
4
5
6
7
8
9
10

2b

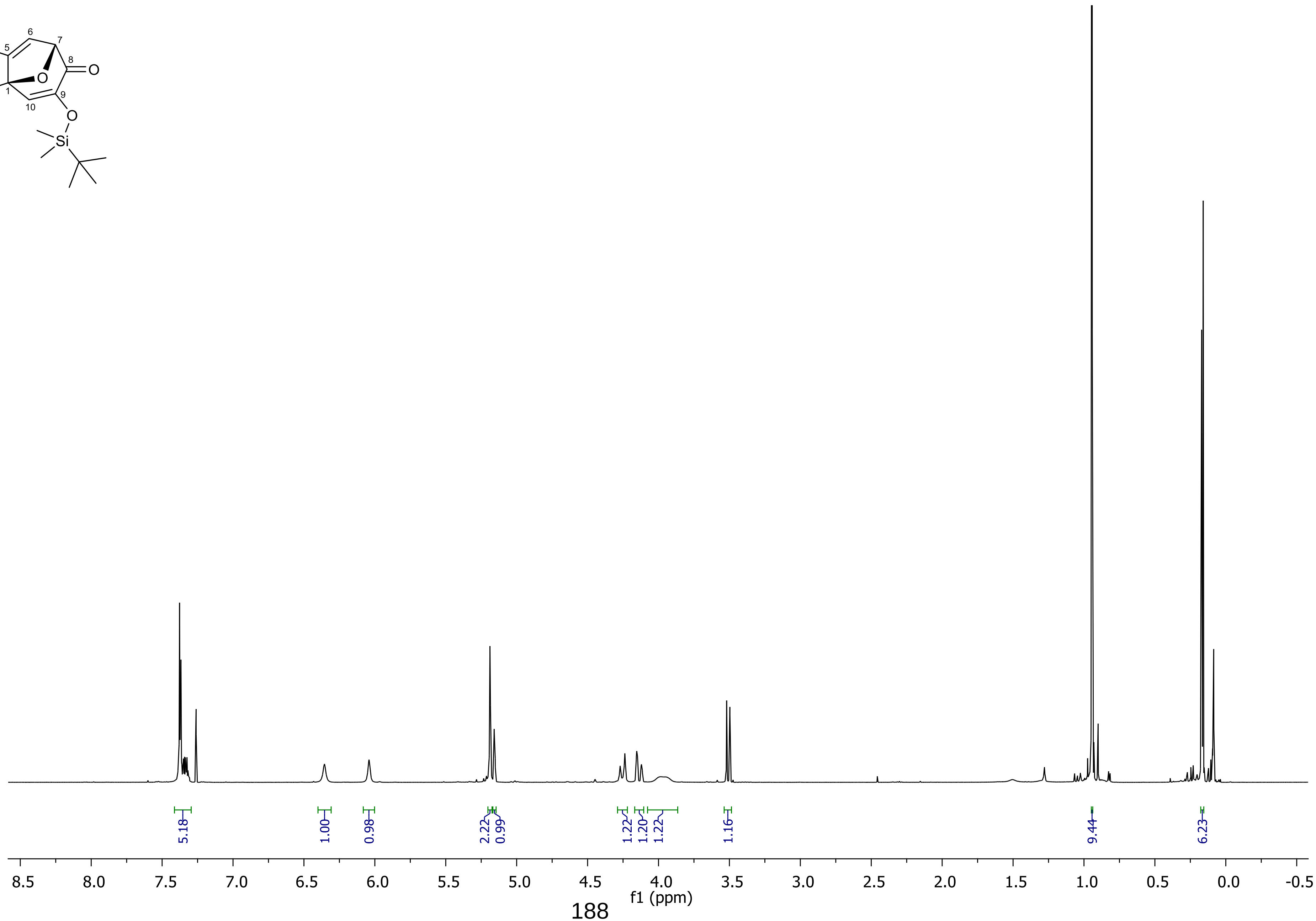

5.18
1.00
0.98
2.22
0.99
1.22
1.20
1.22
1.16
9.44
6.23
8.5
8.0
7.5
7.0
6.5
6.0
5.5
5.0
4.5
4.0
3.5
3.0
2.5
2.0
1.5
1.0
0.5
0.0
-0.5
f1 (ppm)

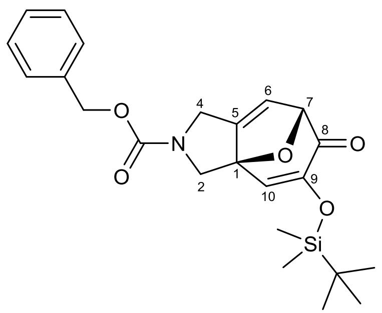

2b

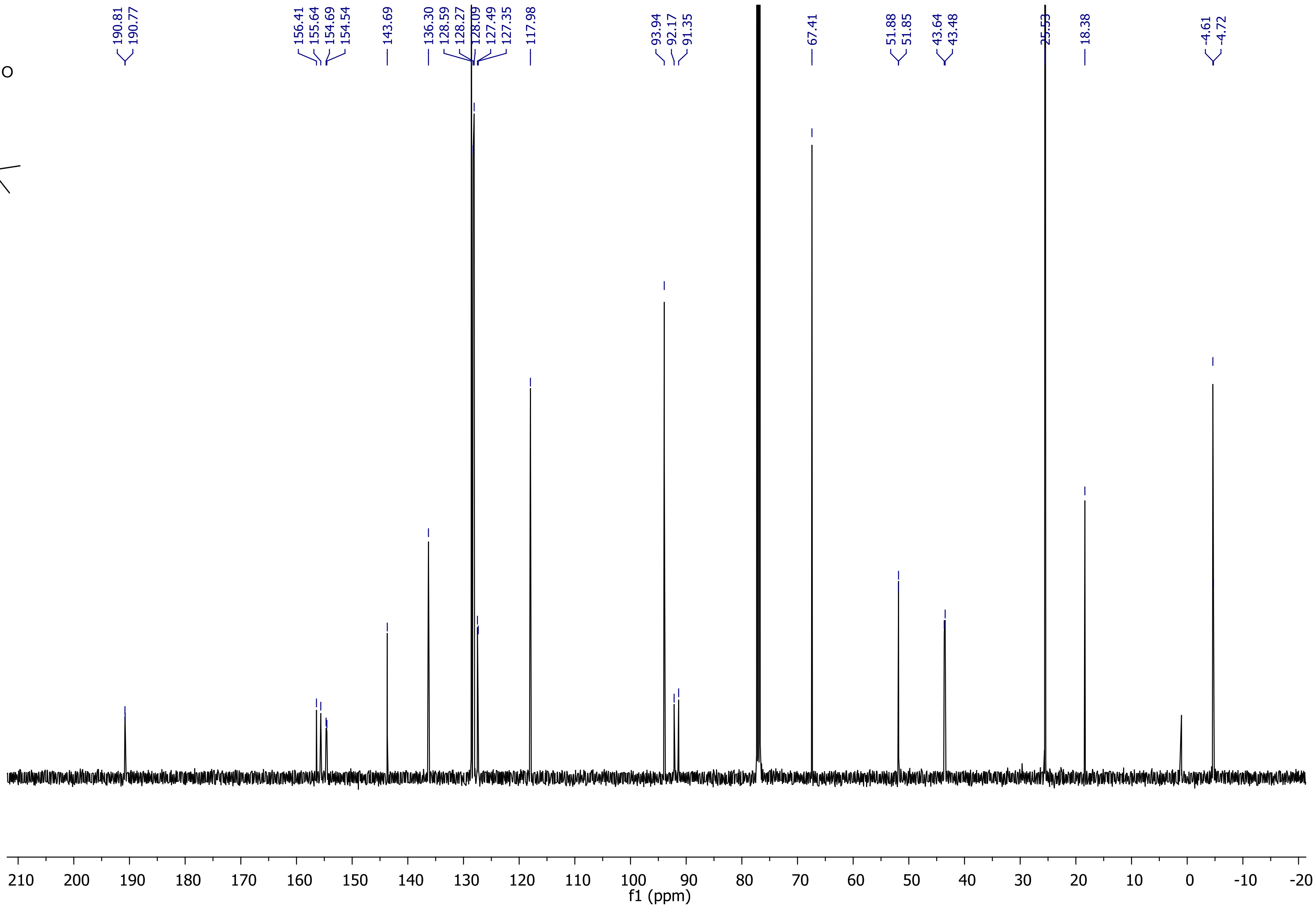
190.81
190.77
156.41
155.64
154.69
154.54
143.69
136.30
128.59
128.27
128.09
127.49
127.35
117.98
93.94
92.17
91.35
67.41
51.88
51.85
43.64
43.48
25.53
18.38
-4.61
-4.72
210 200 190 180 170 160 150 140 130 120 110 100 90 80 70 60 50 40 30 20 10 0 -10 -20
f1 (ppm)

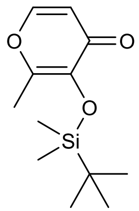

S7

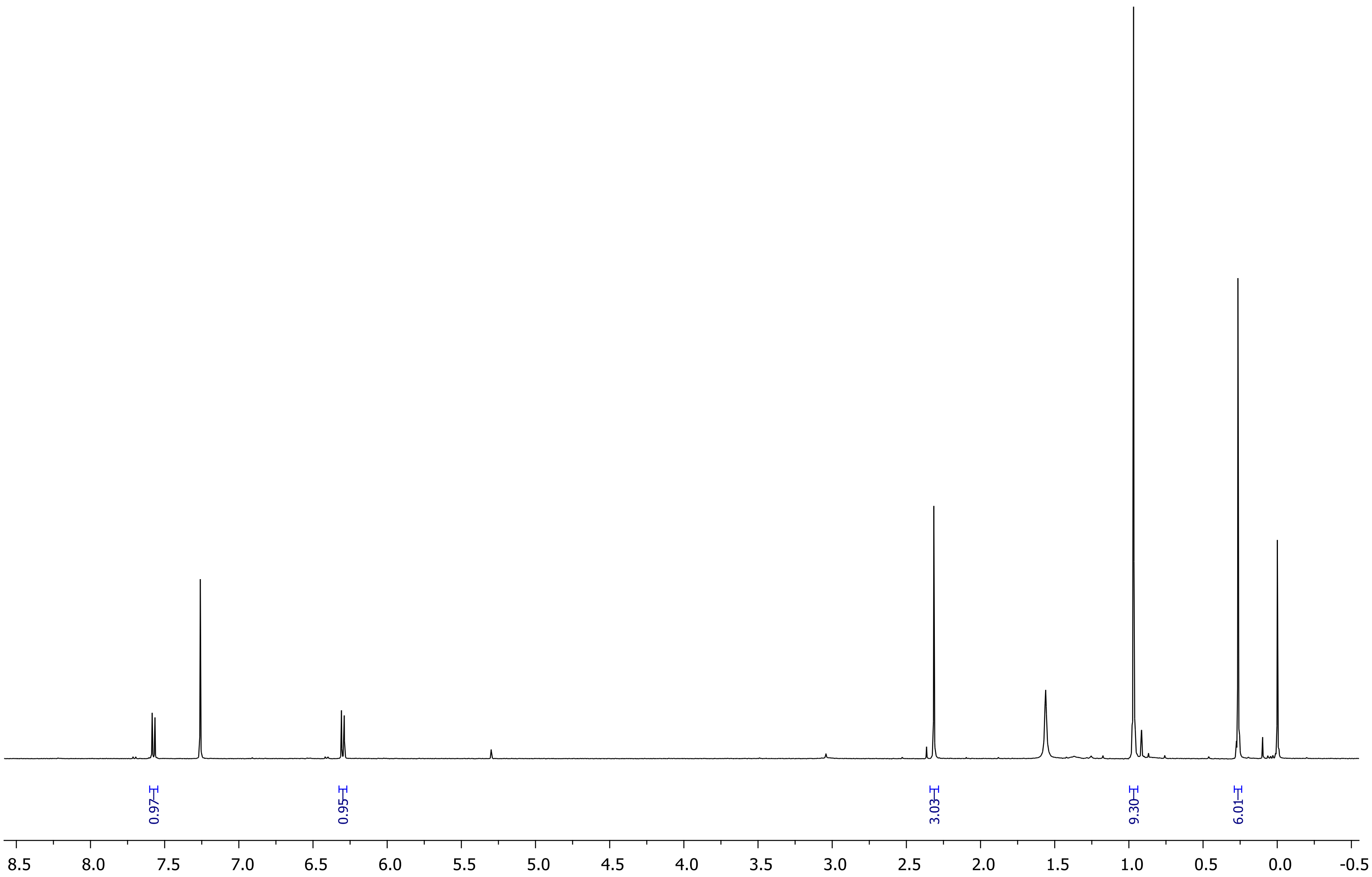

0.97
0.95
3.03
9.30
6.01
8.5 8.0 7.5 7.0 6.5 6.0 5.5 5.0 4.5 4.0 3.5 3.0 2.5 2.0 1.5 1.0 0.5 0.0 -0.5

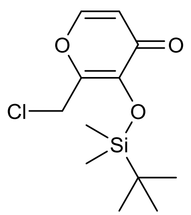

S8

carried forward crude

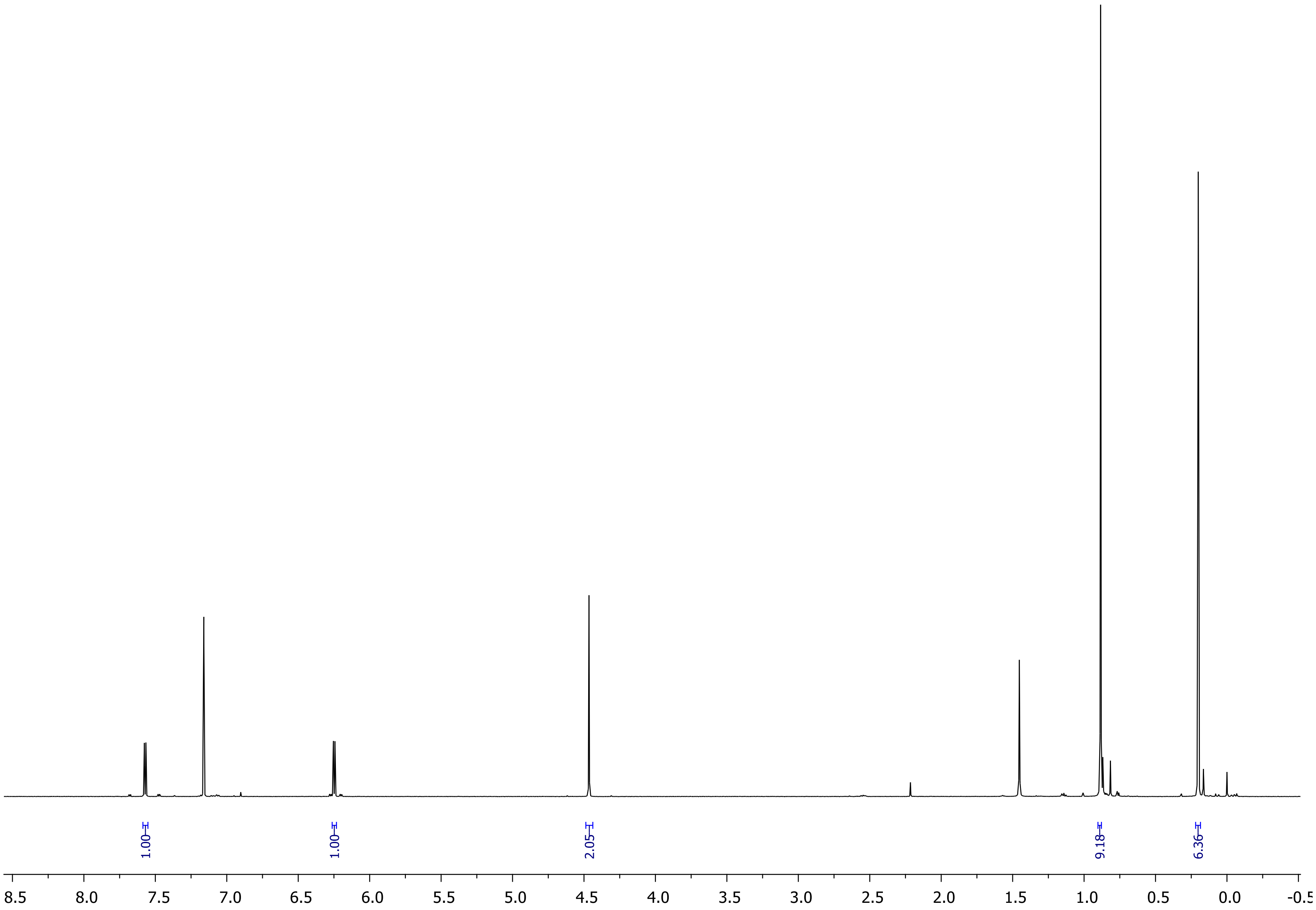

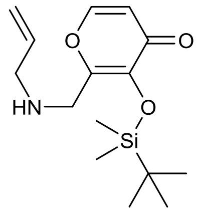

S9

carried forward crude

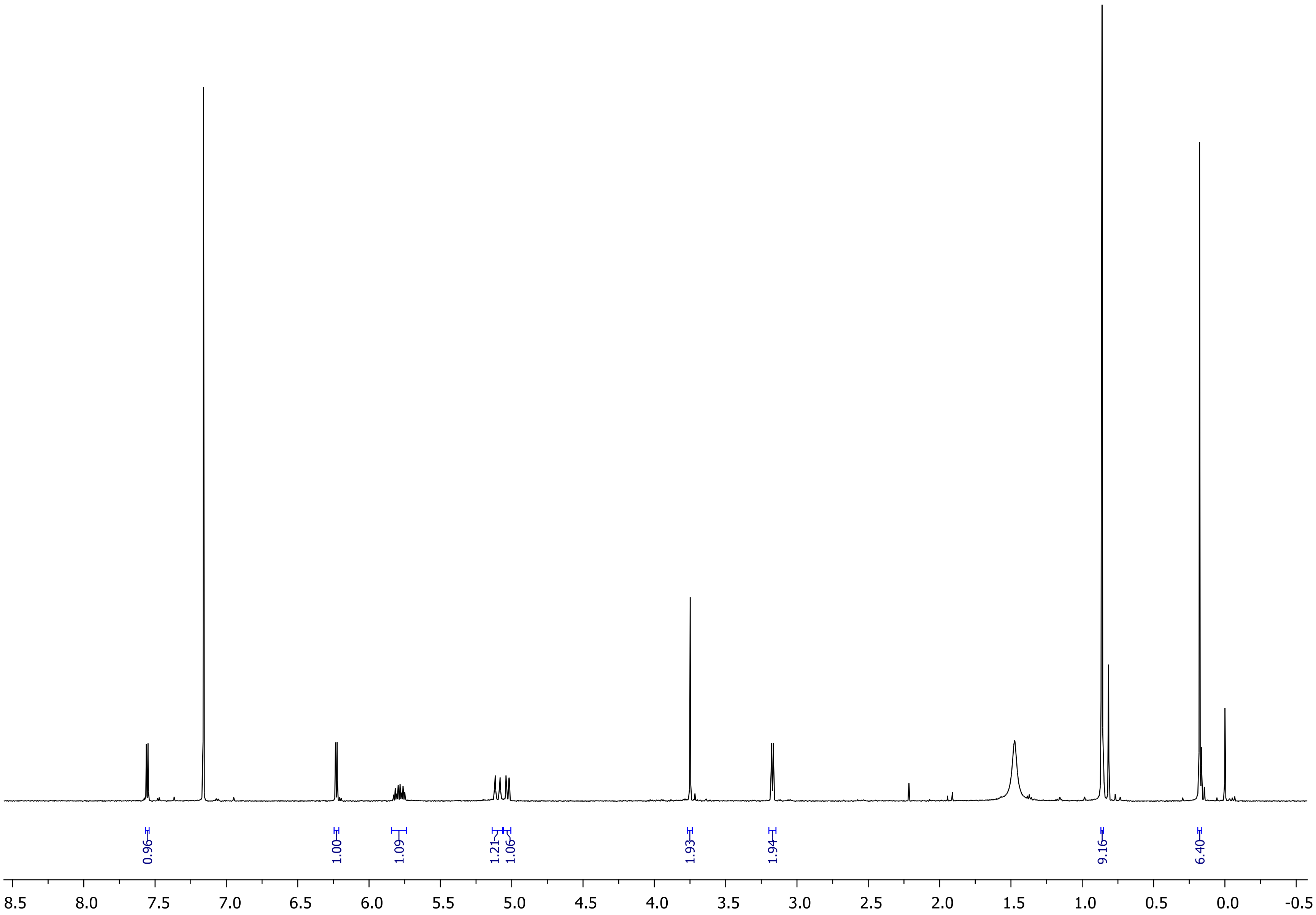

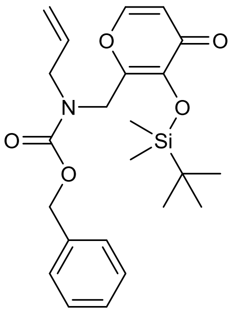

S10

carried forward crude

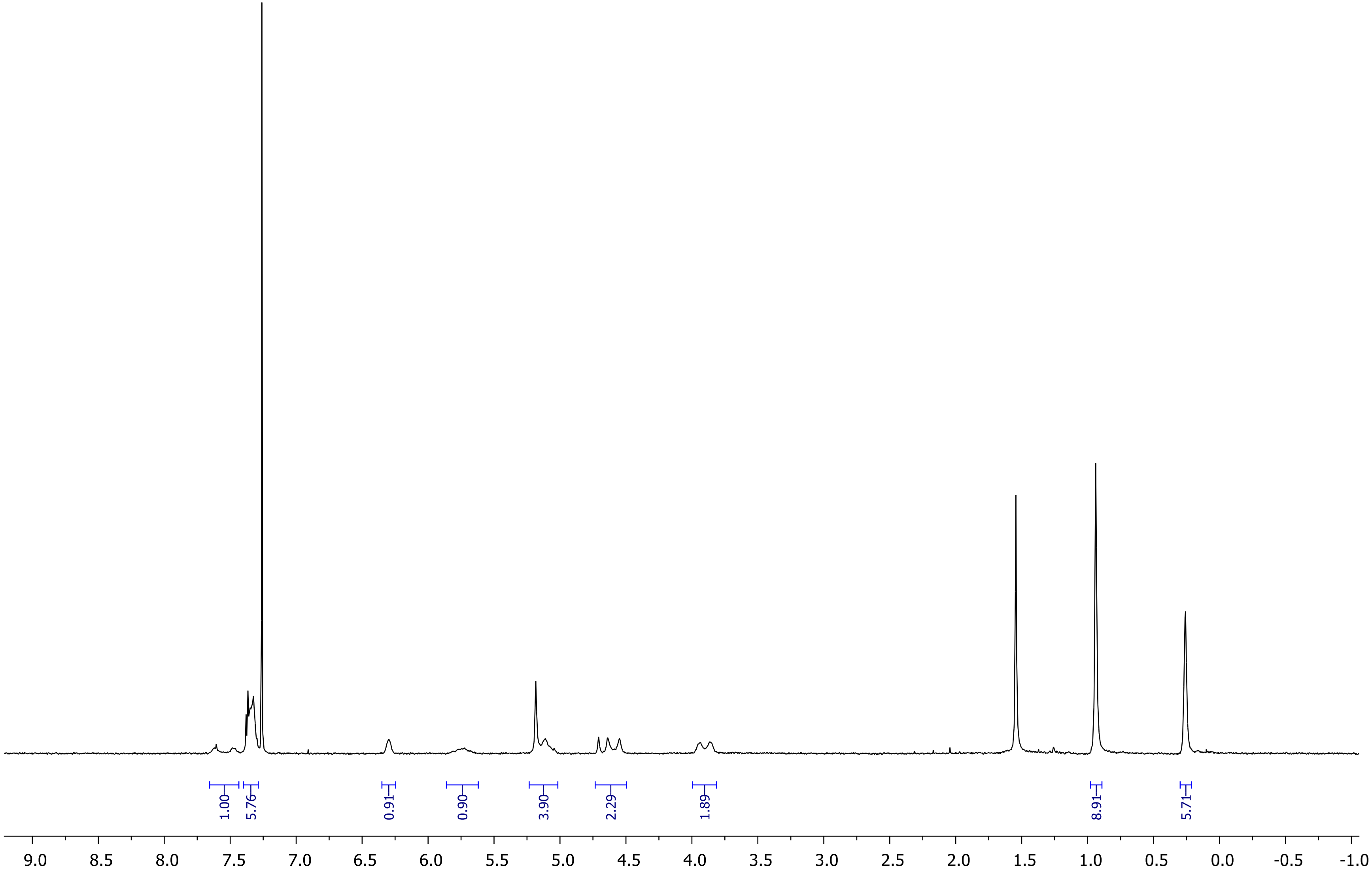

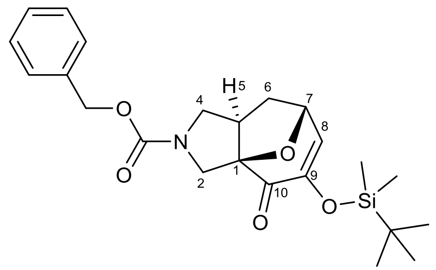

2c

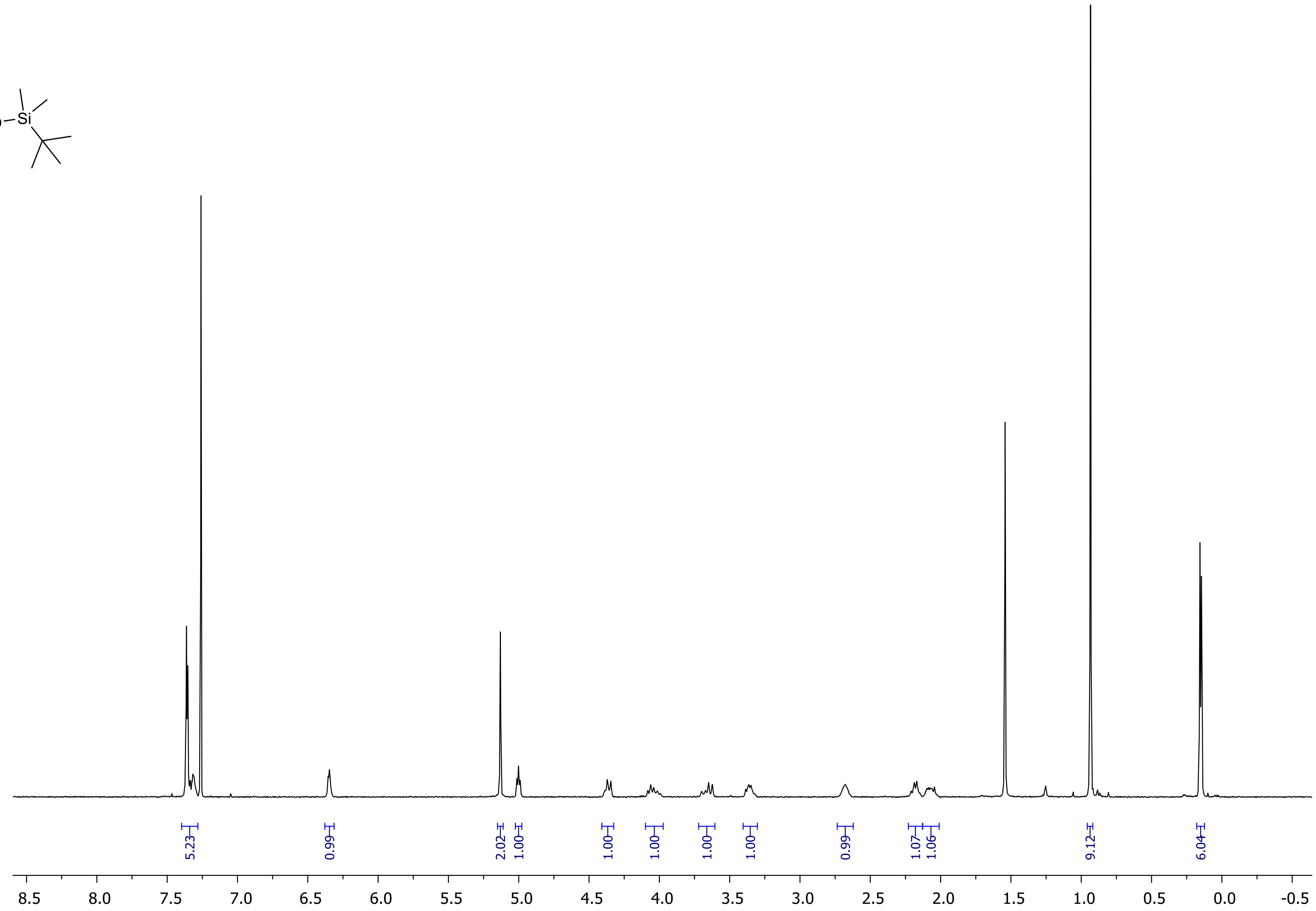

5.23
0.99
2.02
1.00
1.00
1.00
1.00
1.00
0.99
1.07
1.06
9.12
6.04
8.5
8.0
7.5
7.0
6.5
6.0
5.5
5.0
4.5
4.0
3.5
3.0
2.5
2.0
1.5
1.0
0.5
0.0
-0.5

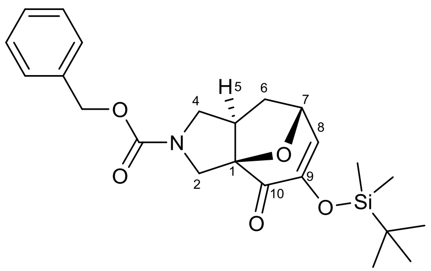

2c
$CDCl_3$ @ 329 K

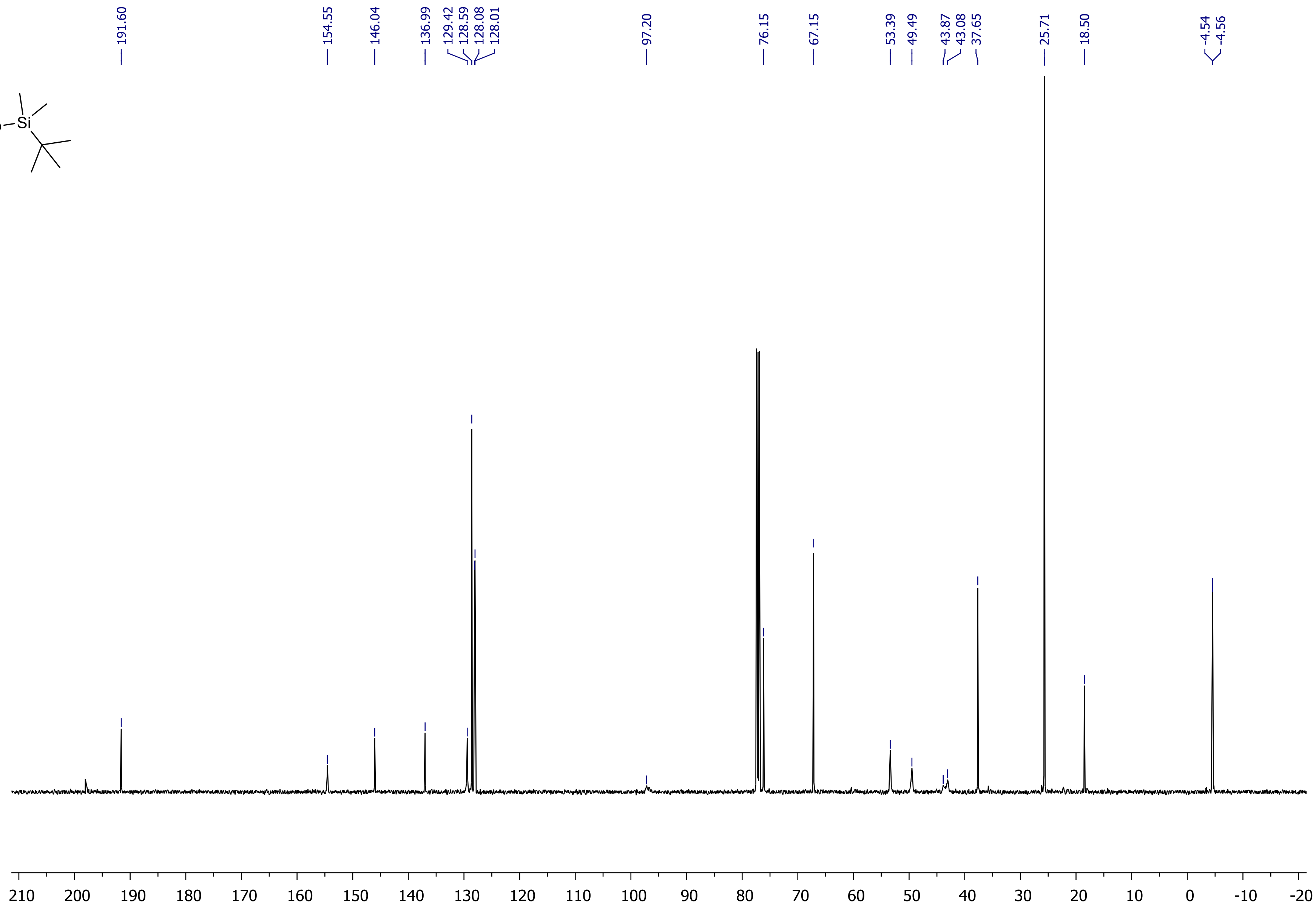

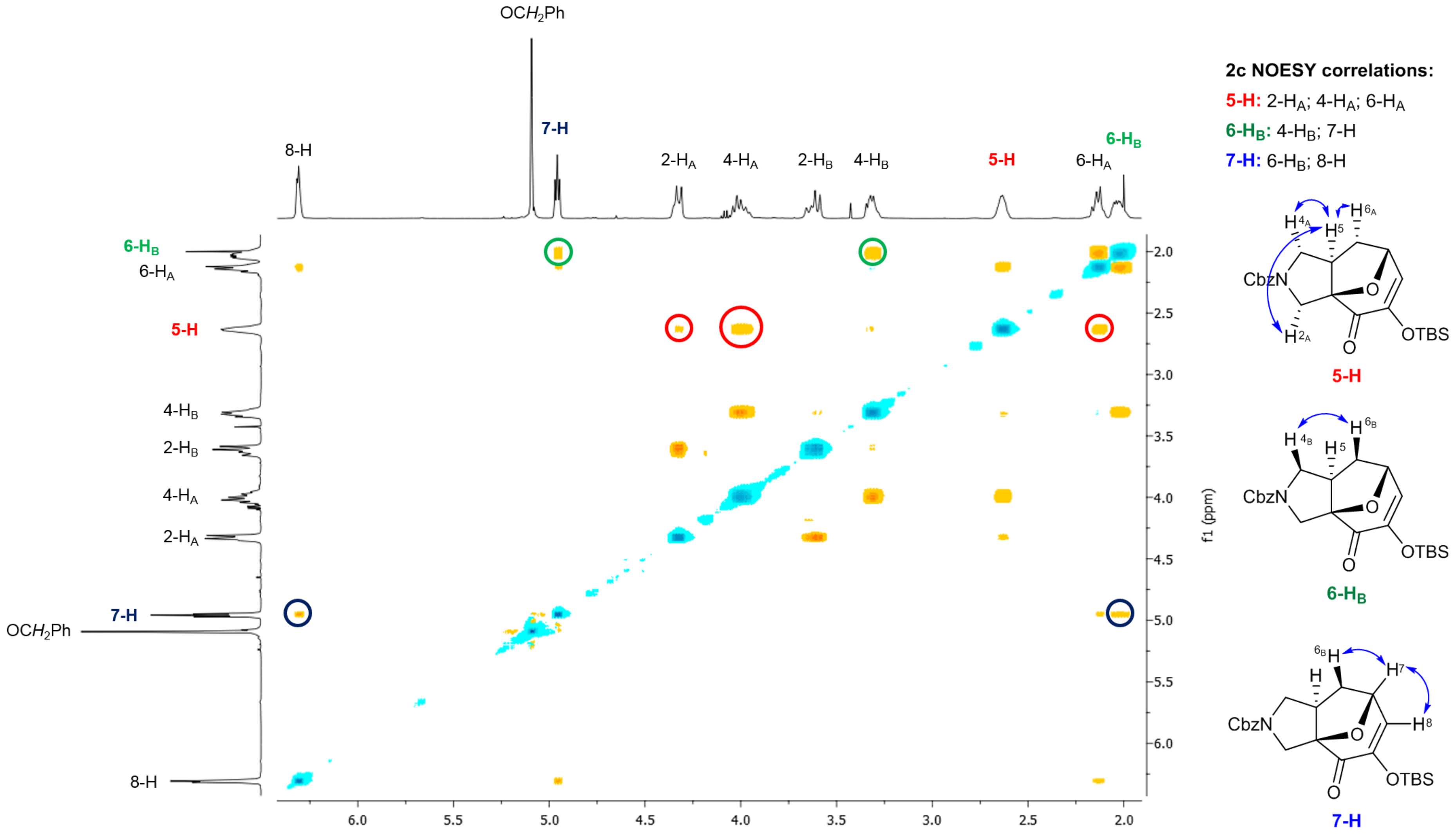

2c NOESY correlations:
5-H: 2-H_A; 4-H_A; 6-H_A
6-H_B: 4-H_B; 7-H
7-H: 6-H_B; 8-H
OCH_2Ph
8-H
7-H
2-H_A
4-H_A
2-H_B
4-H_B
5-H
6-H_A
6-H_B
f1 (ppm)
CbzN
OTBS
O

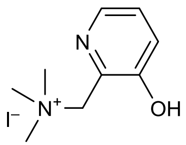
N
N+
I−
OH

S11

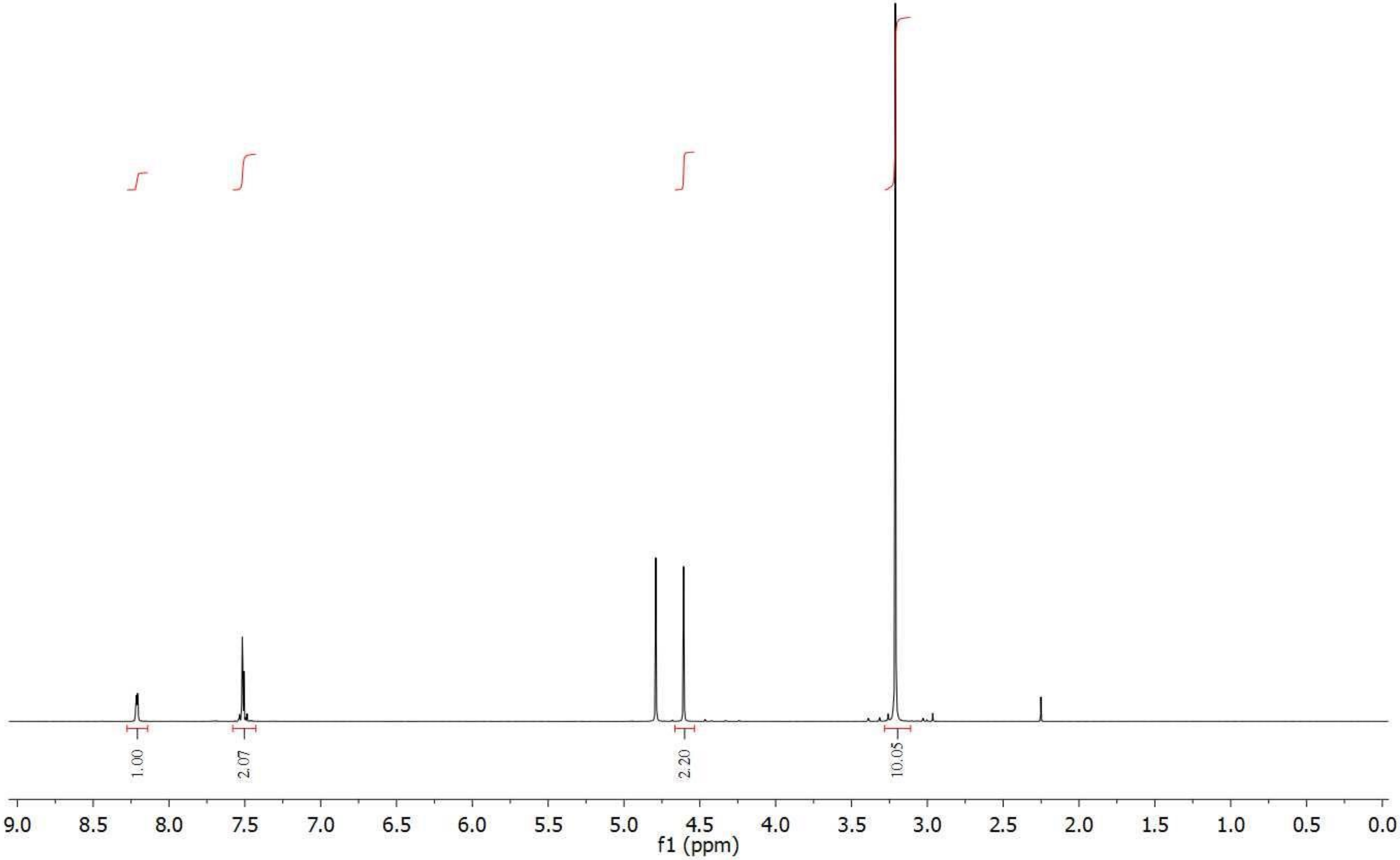
1.00
2.07
2.20
10.05
9.0
8.5
8.0
7.5
7.0
6.5
6.0
5.5
5.0
4.5
4.0
3.5
3.0
2.5
2.0
1.5
1.0
0.5
0.0
f1 (ppm)

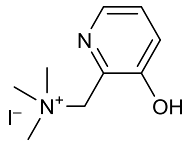
N
N+
I−
OH

S11

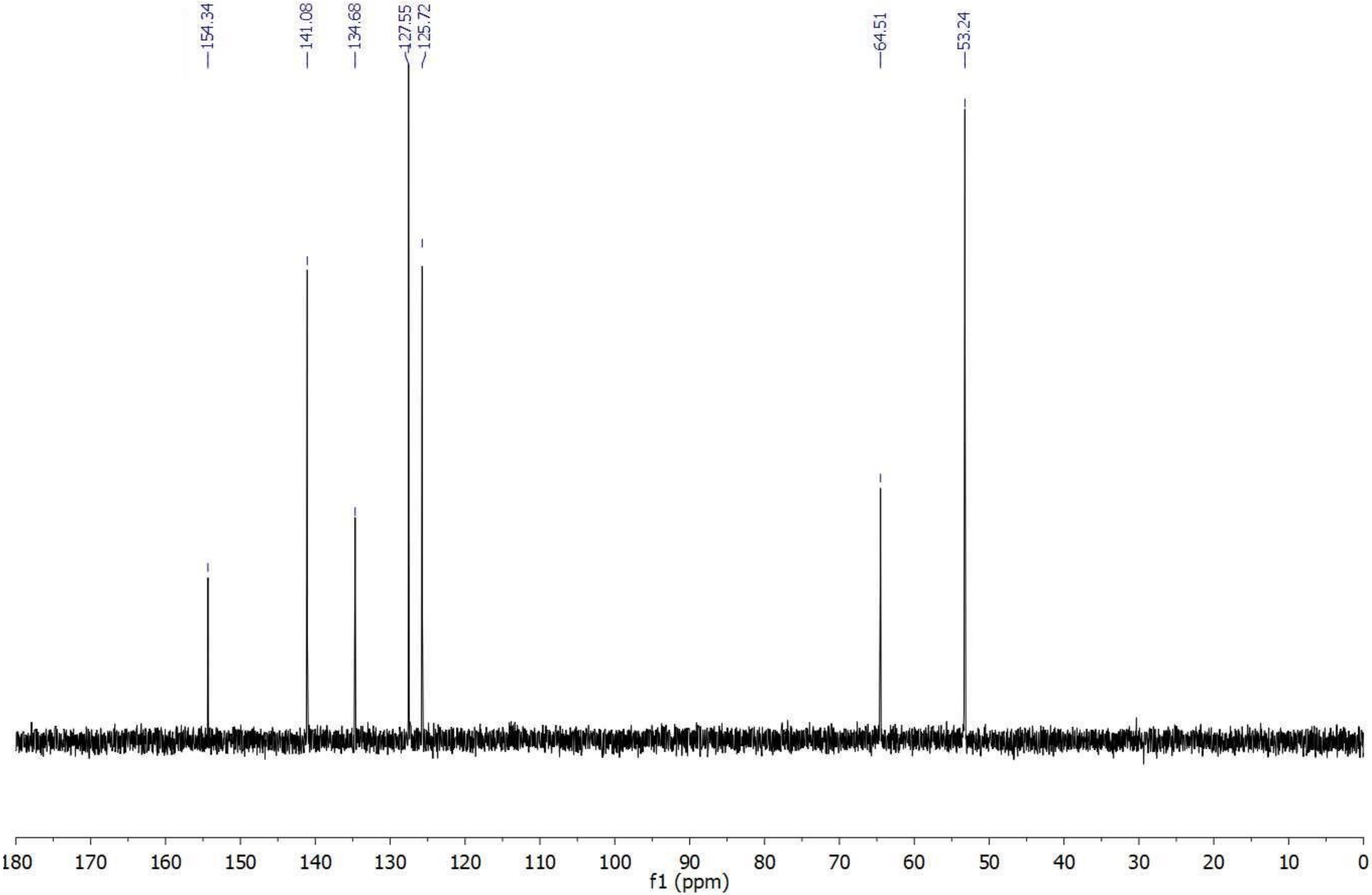
154.34
141.08
134.68
127.55
125.72
64.51
53.24
180
170
160
150
140
130
120
110
100
90
80
70
60
50
40
30
20
10
0
f1 (ppm)

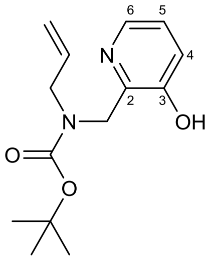
N
OH
O
O
2
3
4
5
6
S12

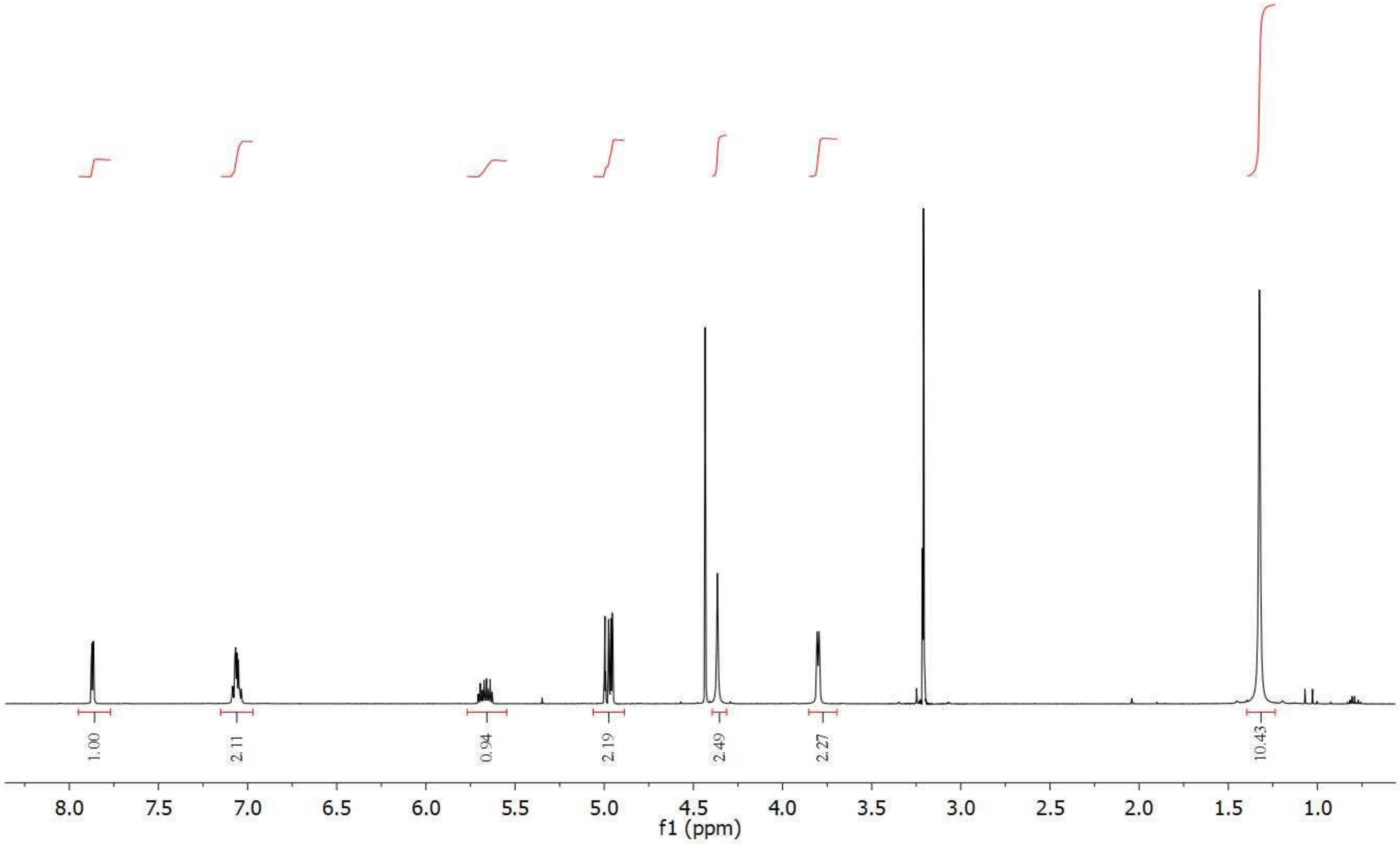
1.00
2.11
0.94
2.19
2.49
2.27
10.43
8.0
7.5
7.0
6.5
6.0
5.5
5.0
4.5
4.0
3.5
3.0
2.5
2.0
1.5
1.0
f1 (ppm)

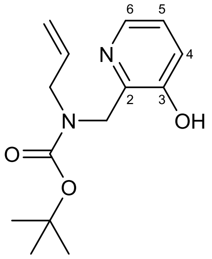

S12

(CD_3OD @ 333 K)

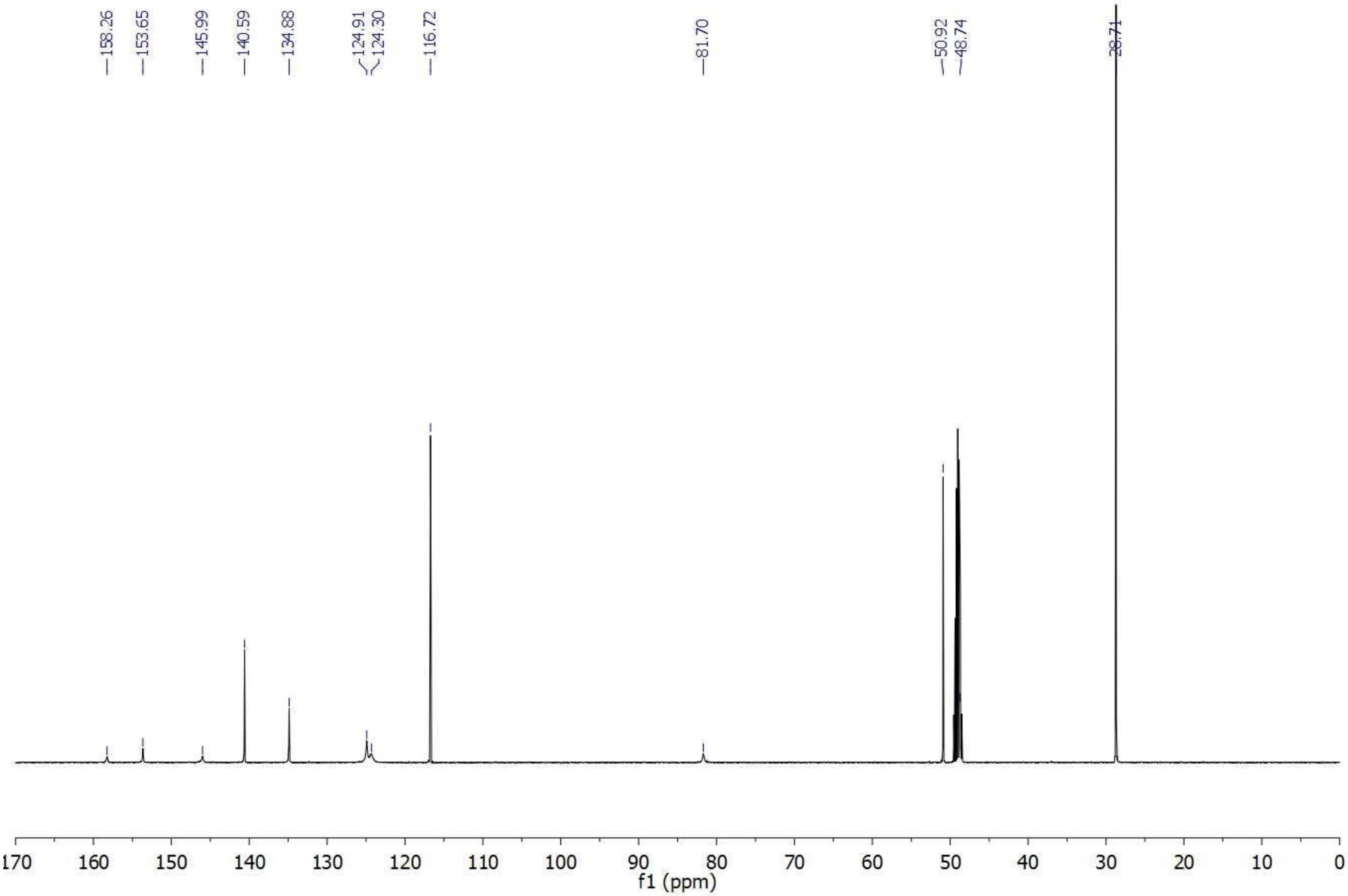

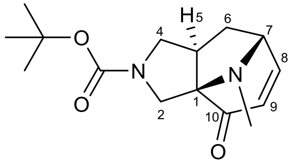

2d
(CD_3OD @ 333 K)

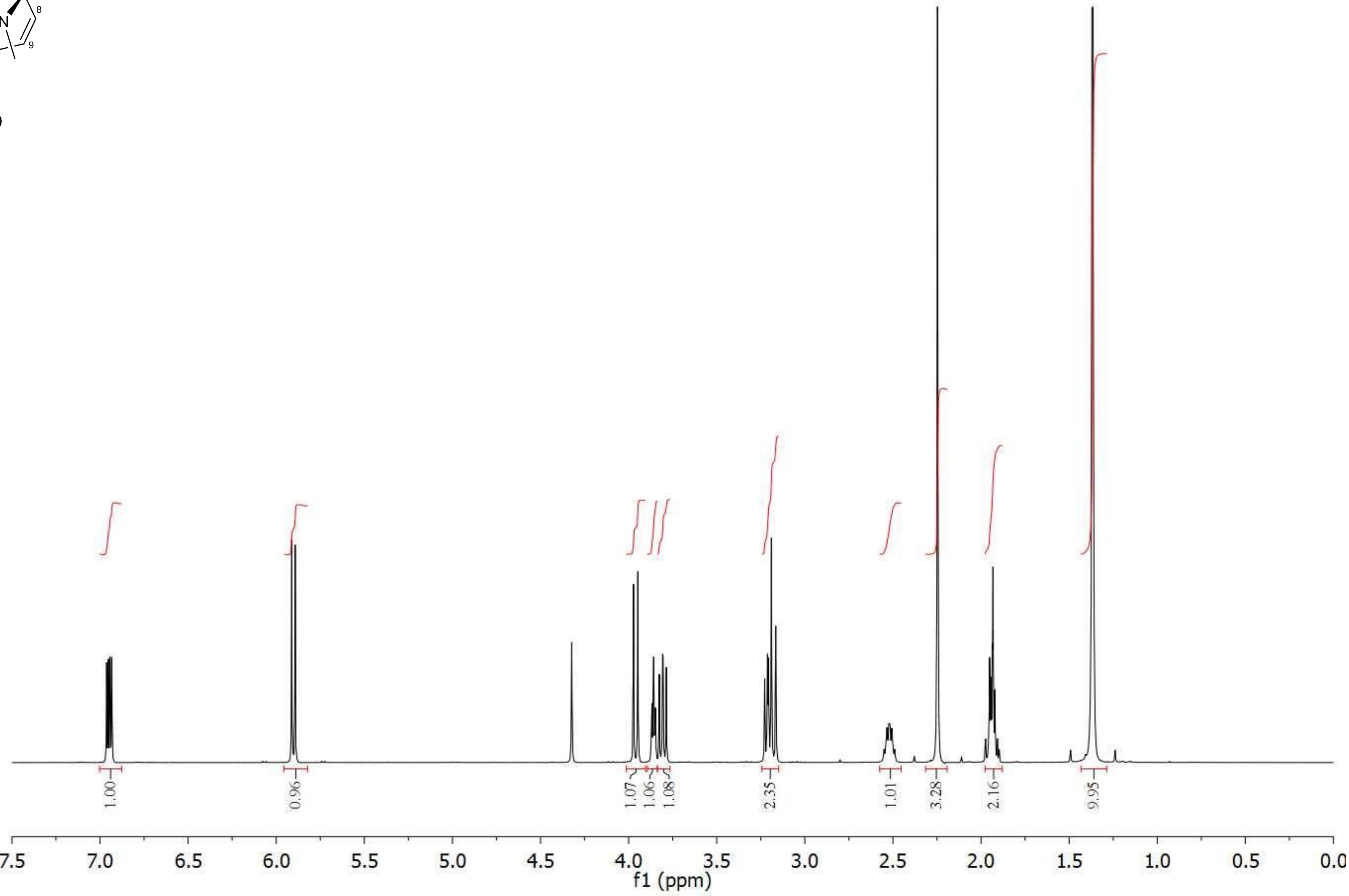

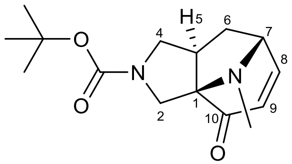

2d
(CD_3OD @ 333 K)

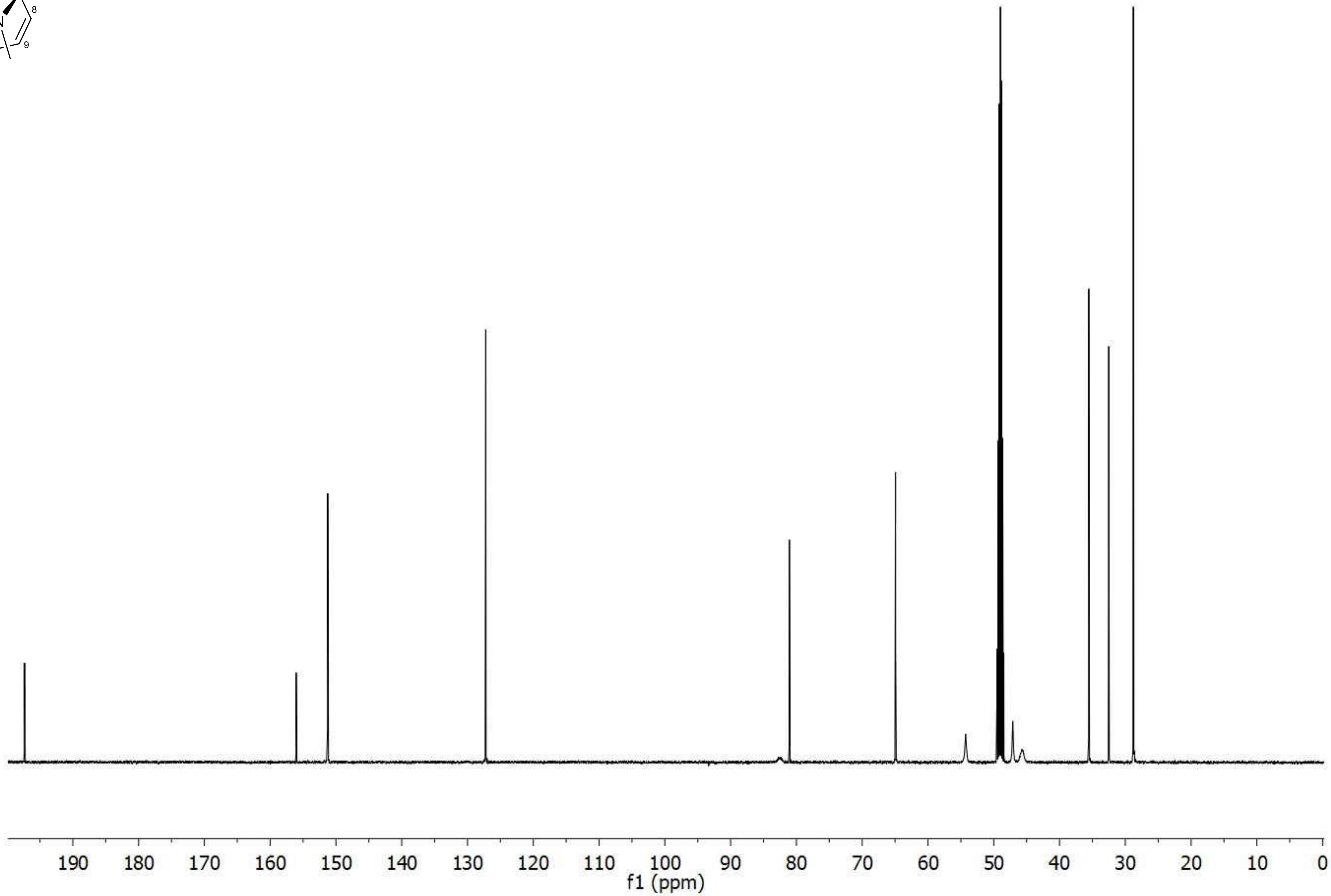

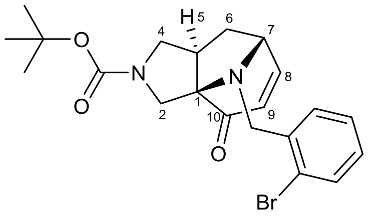

2e
(CD_3OD @ 333 K)

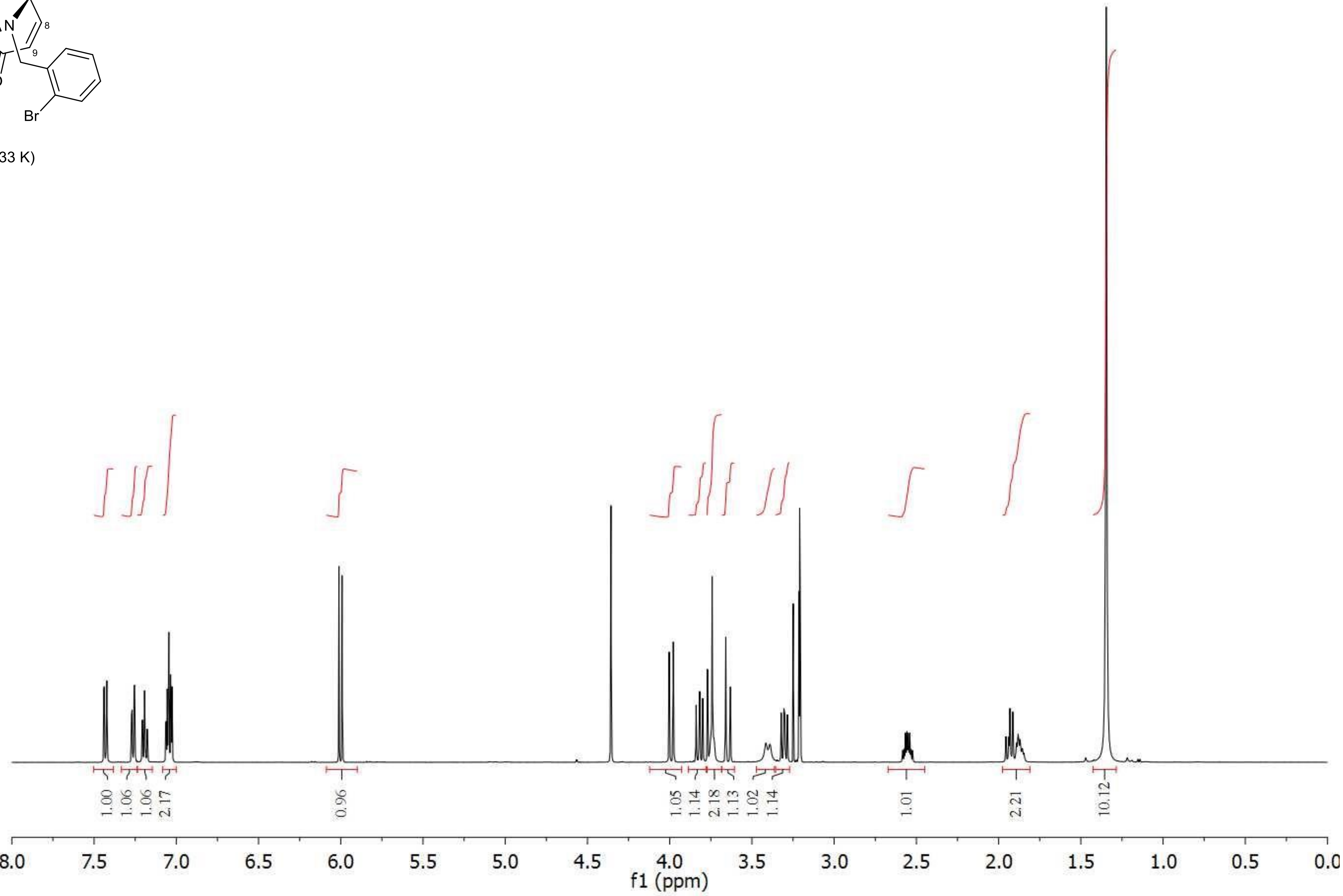

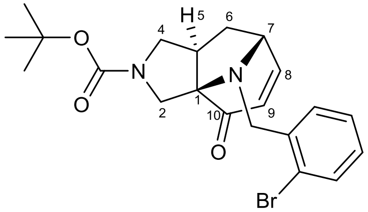

2e
(CD_3OD @ 333 K)

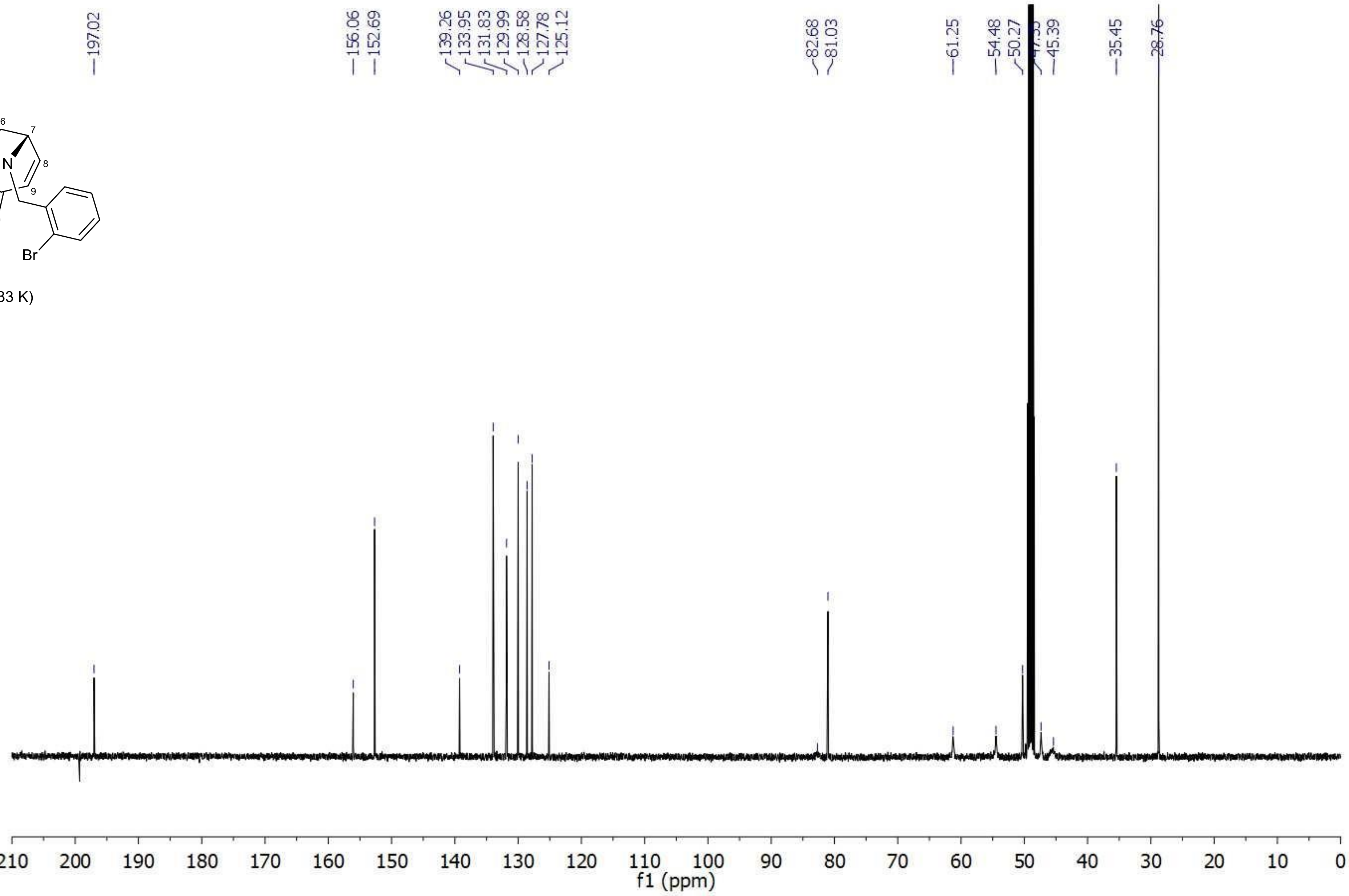

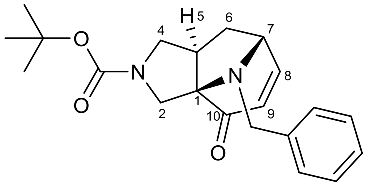

2f
(CD_3OD @ 333 K)

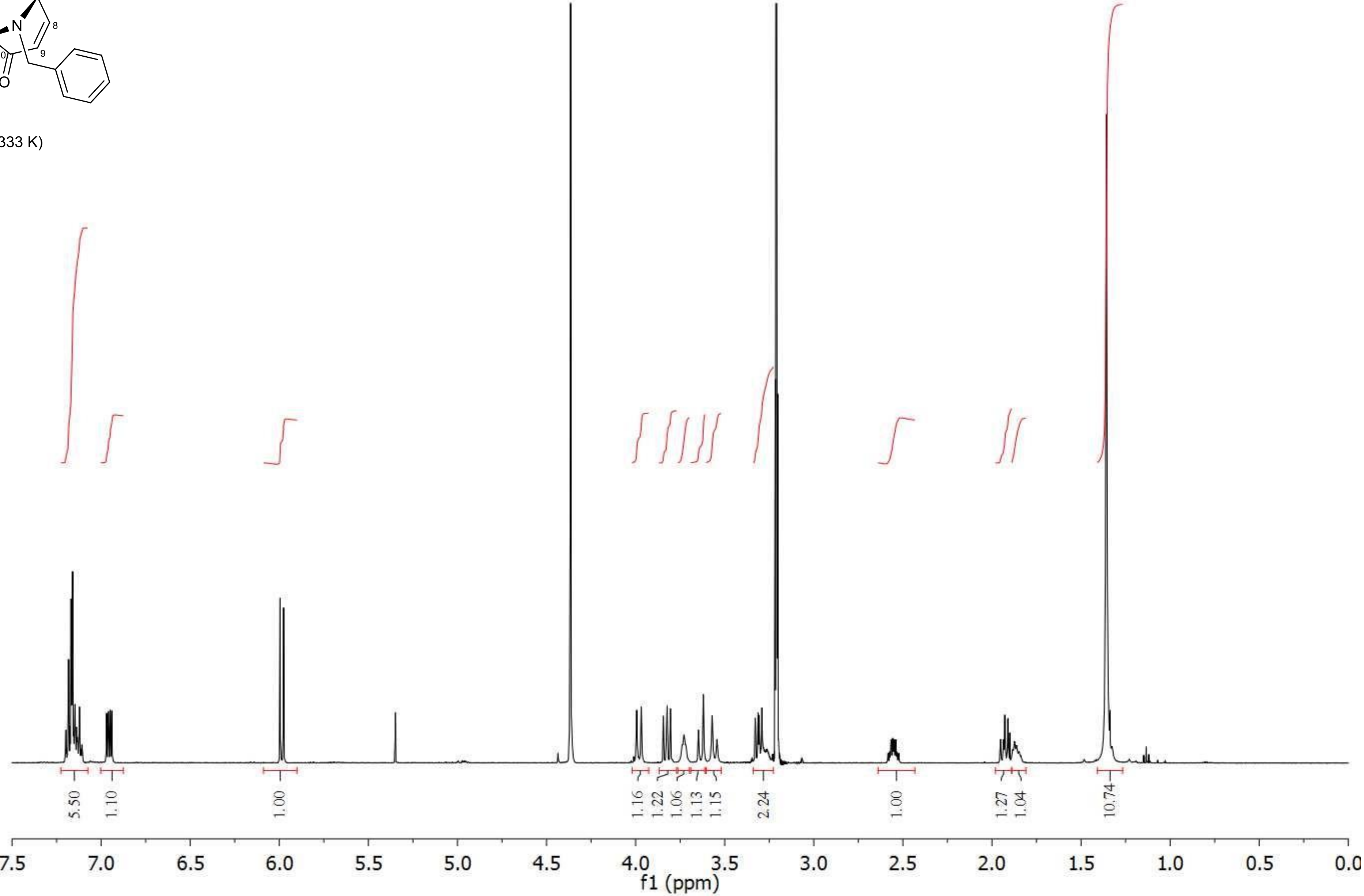

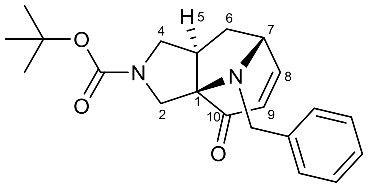

2f
(CD_3OD @ 333 K)

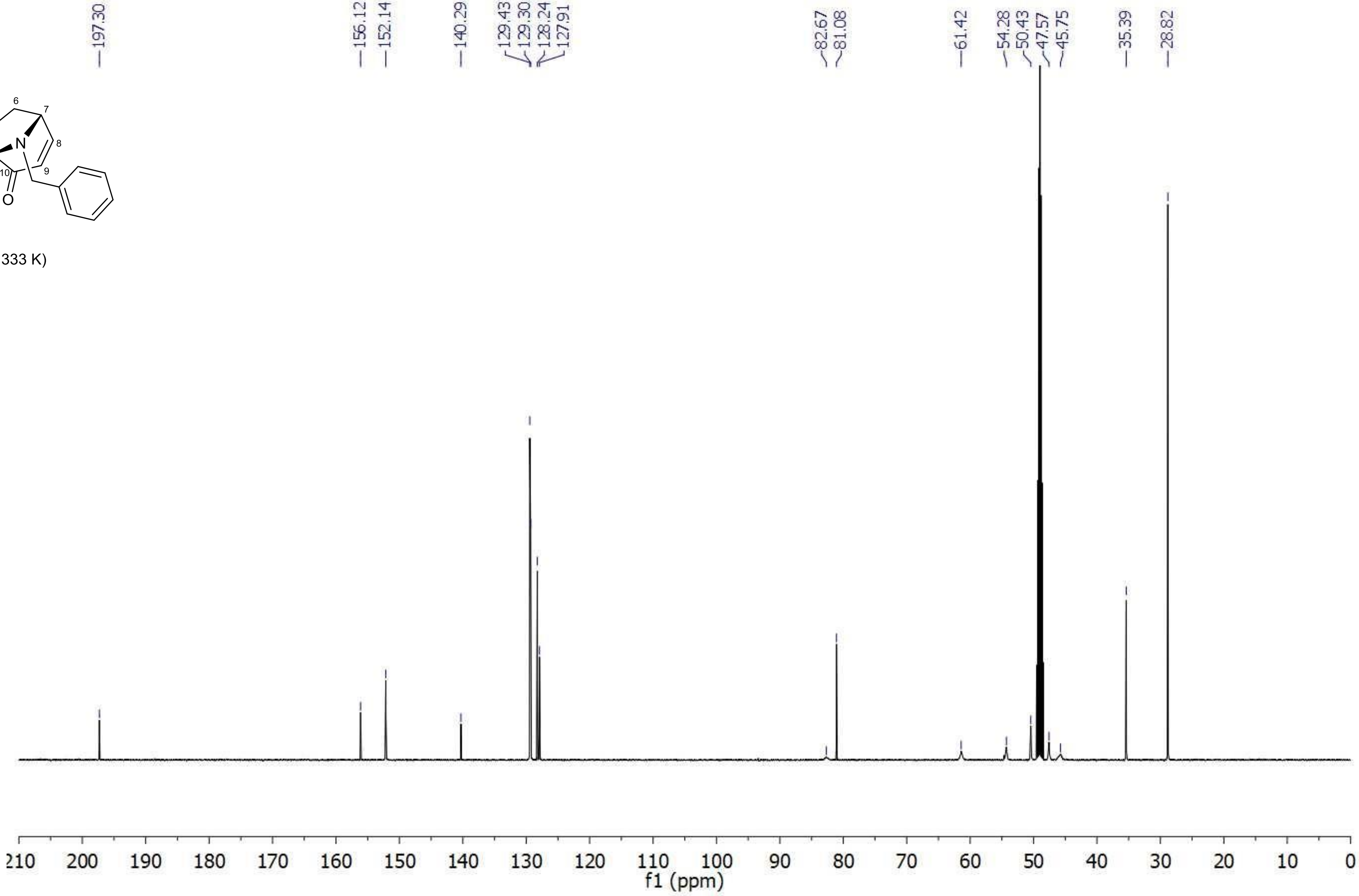

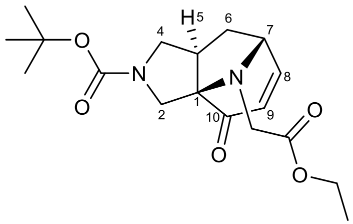

2g
(CD_3OD @ 333 K)

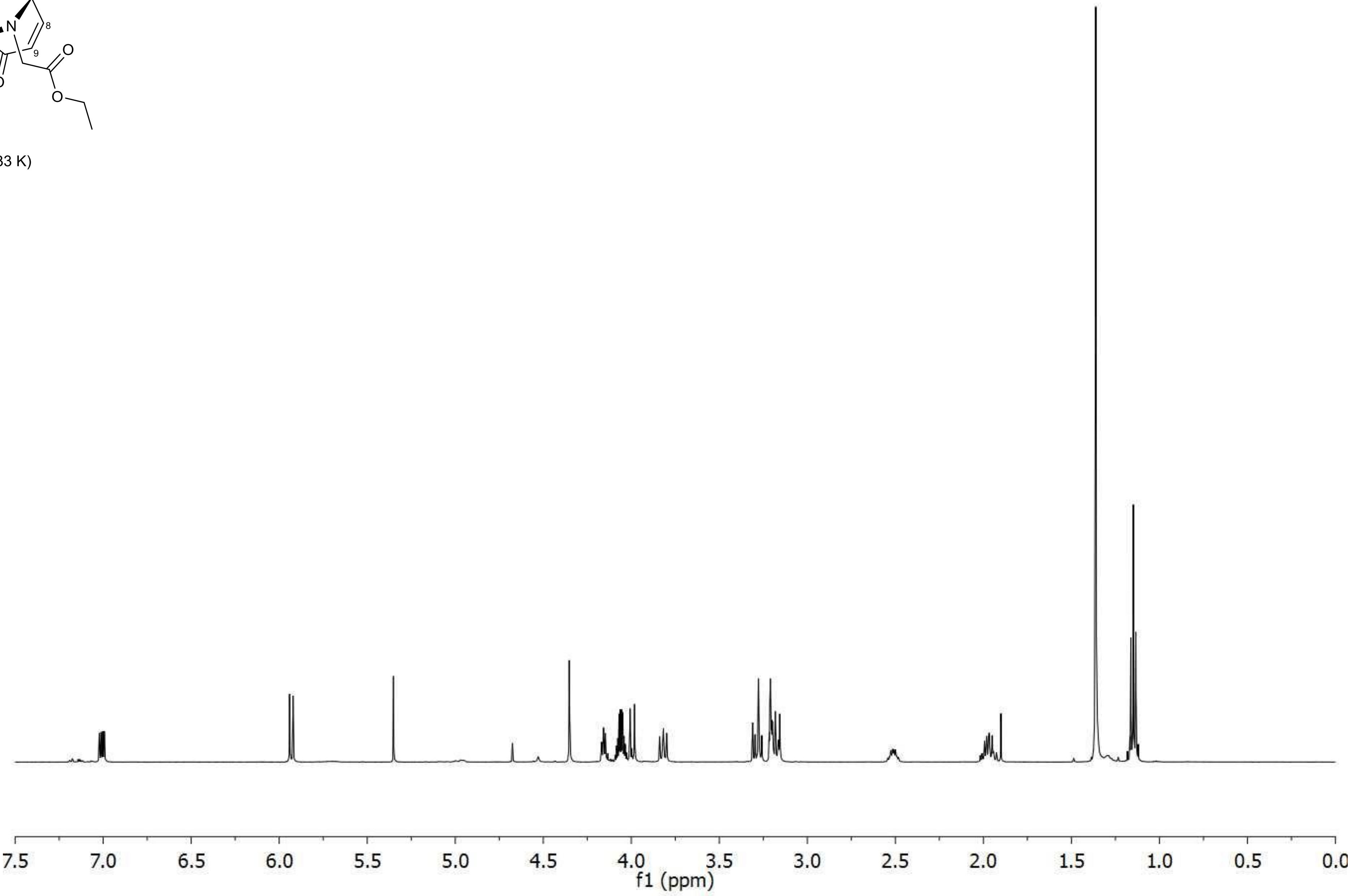

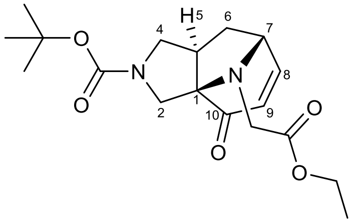

2g
(CD_3OD @ 333 K)

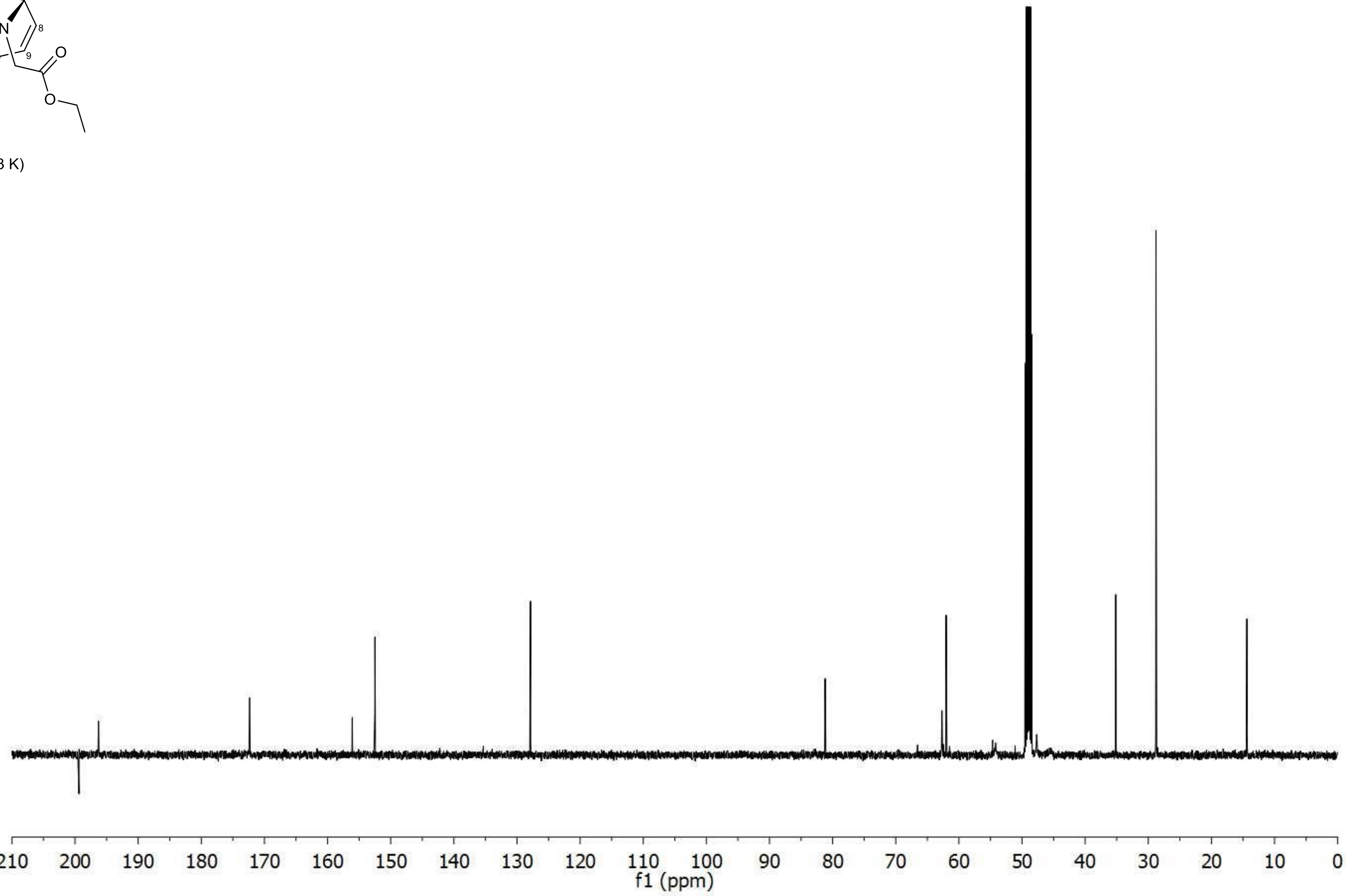

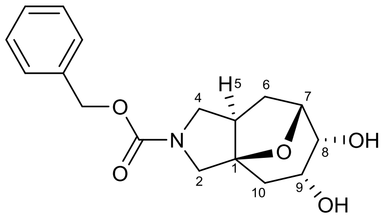
OH
OH
O
N
O
O
H
1
2
4
5
6
7
8
9
10

S13

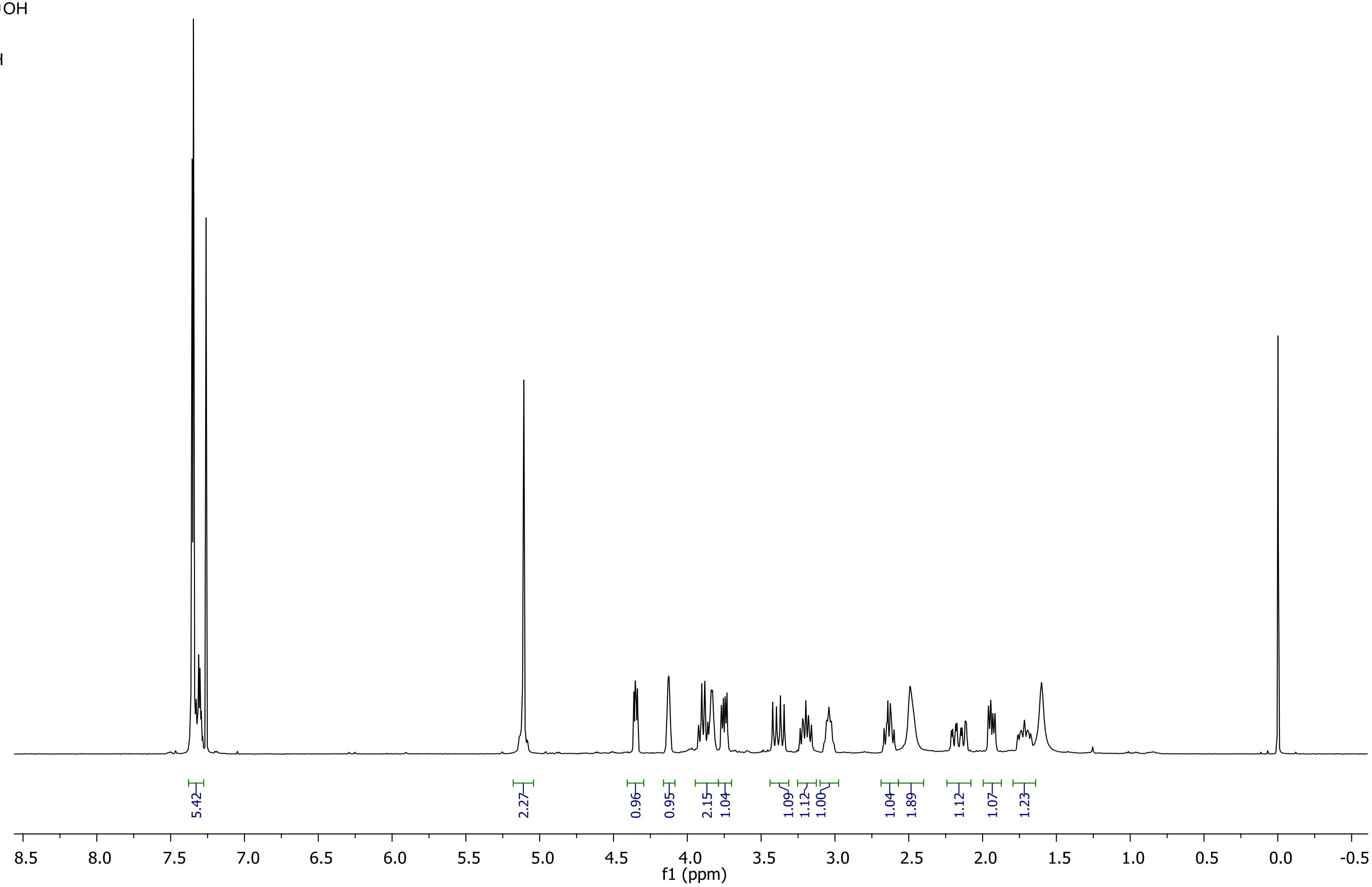
5.42
2.27
0.96
0.95
2.15
1.04
1.09
1.12
1.00
1.04
1.89
1.12
1.07
1.23
8.5
8.0
7.5
7.0
6.5
6.0
5.5
5.0
4.5
4.0
3.5
3.0
2.5
2.0
1.5
1.0
0.5
0.0
-0.5
f1 (ppm)

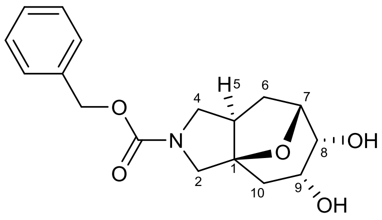
H
5
6
7
4
8
OH
O
N
O
O
1
2
10
9
OH

S13

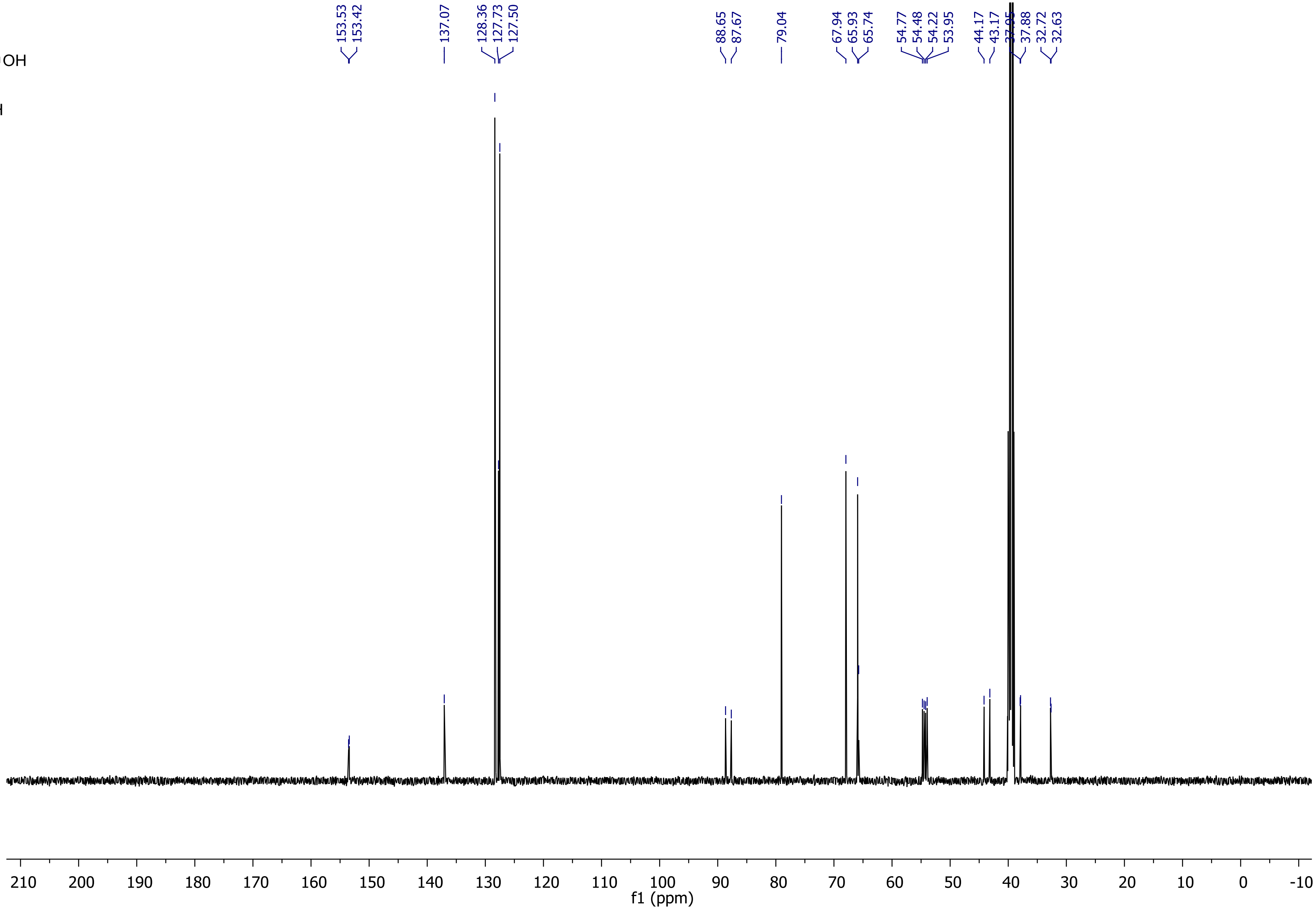
153.53
153.42
137.07
128.36
127.73
127.50
88.65
87.67
79.04
67.94
65.93
65.74
54.77
54.48
54.22
53.95
44.17
43.17
37.95
37.88
32.72
32.63
210 200 190 180 170 160 150 140 130 120 110 100 90 80 70 60 50 40 30 20 10 0 -10
f1 (ppm)

S13 NOESY correlations:

5-H: OH; 4-H_A; 6-H_A; 10-H_A

7-H: 6-H_B; 8-H

8-H: OH; 7-H; 9-H

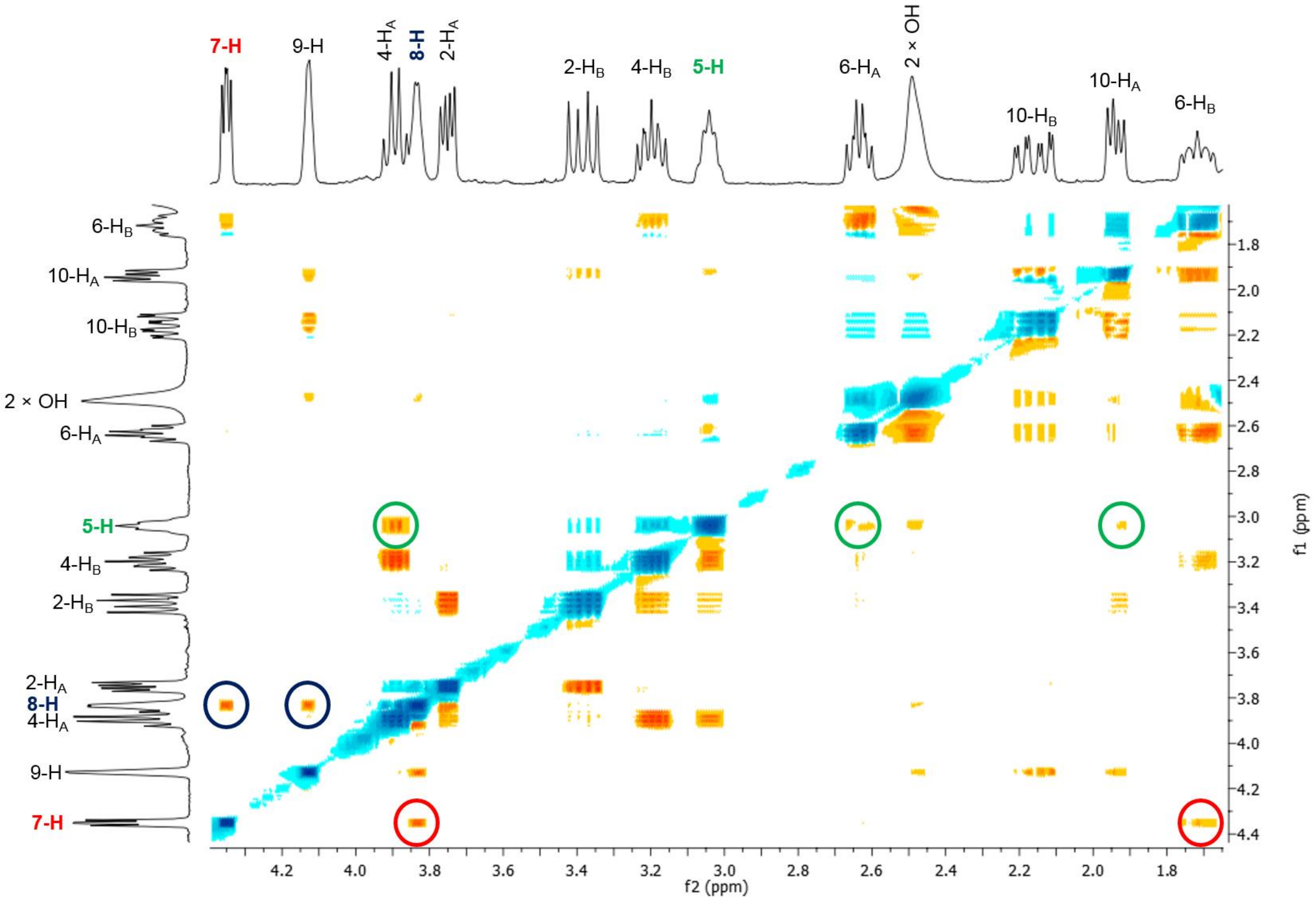

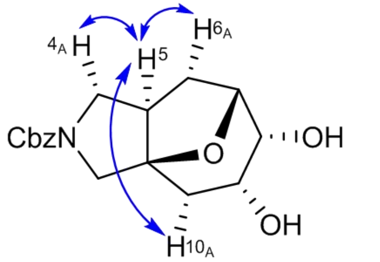

5-H

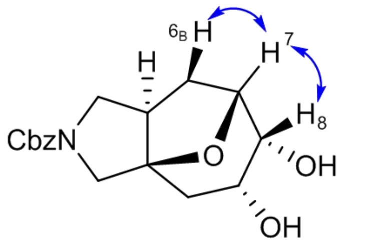

7-H

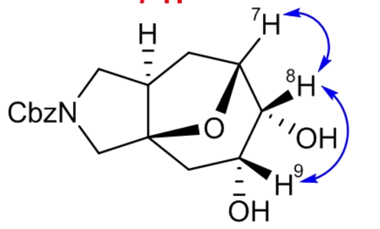

8-H

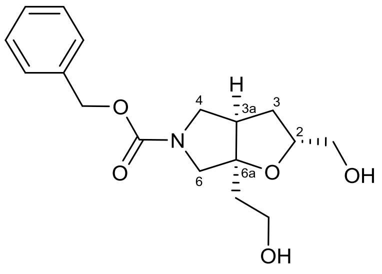

H
4
3a
3
2
N
O
O
O
6
6a
O
OH
OH

7

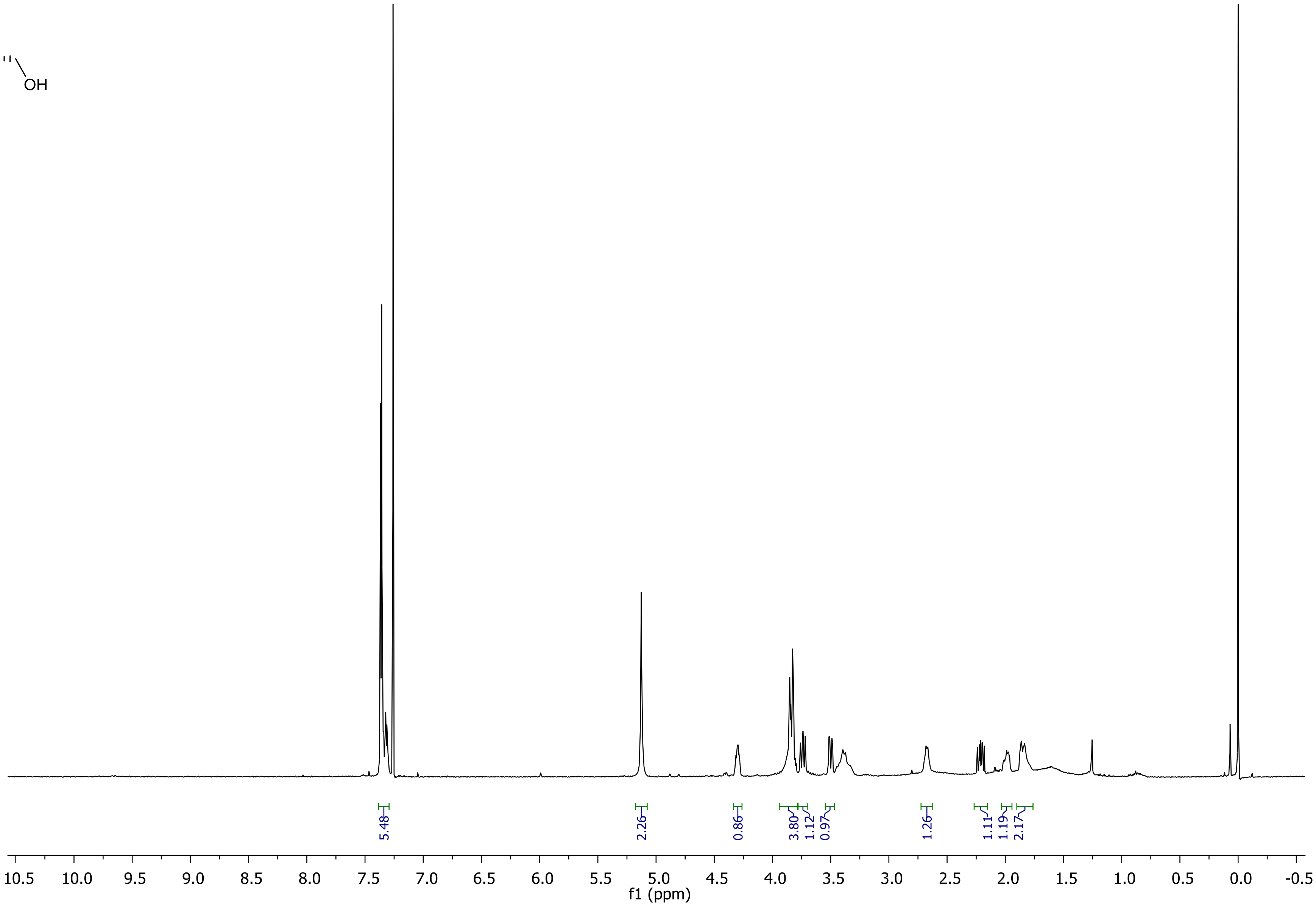

5.48
2.26
0.86
3.80
1.12
0.97
1.26
1.11
1.19
2.17
10.5 10.0 9.5 9.0 8.5 8.0 7.5 7.0 6.5 6.0 5.5 5.0 4.5 4.0 3.5 3.0 2.5 2.0 1.5 1.0 0.5 0.0 -0.5
f1 (ppm)

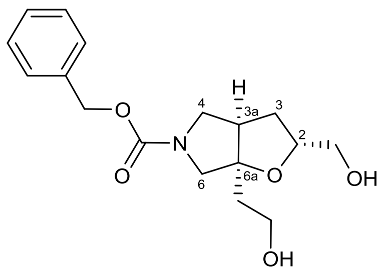

H
3a
3
4
2
N
O
O
O
OH
6
6a
OH

7

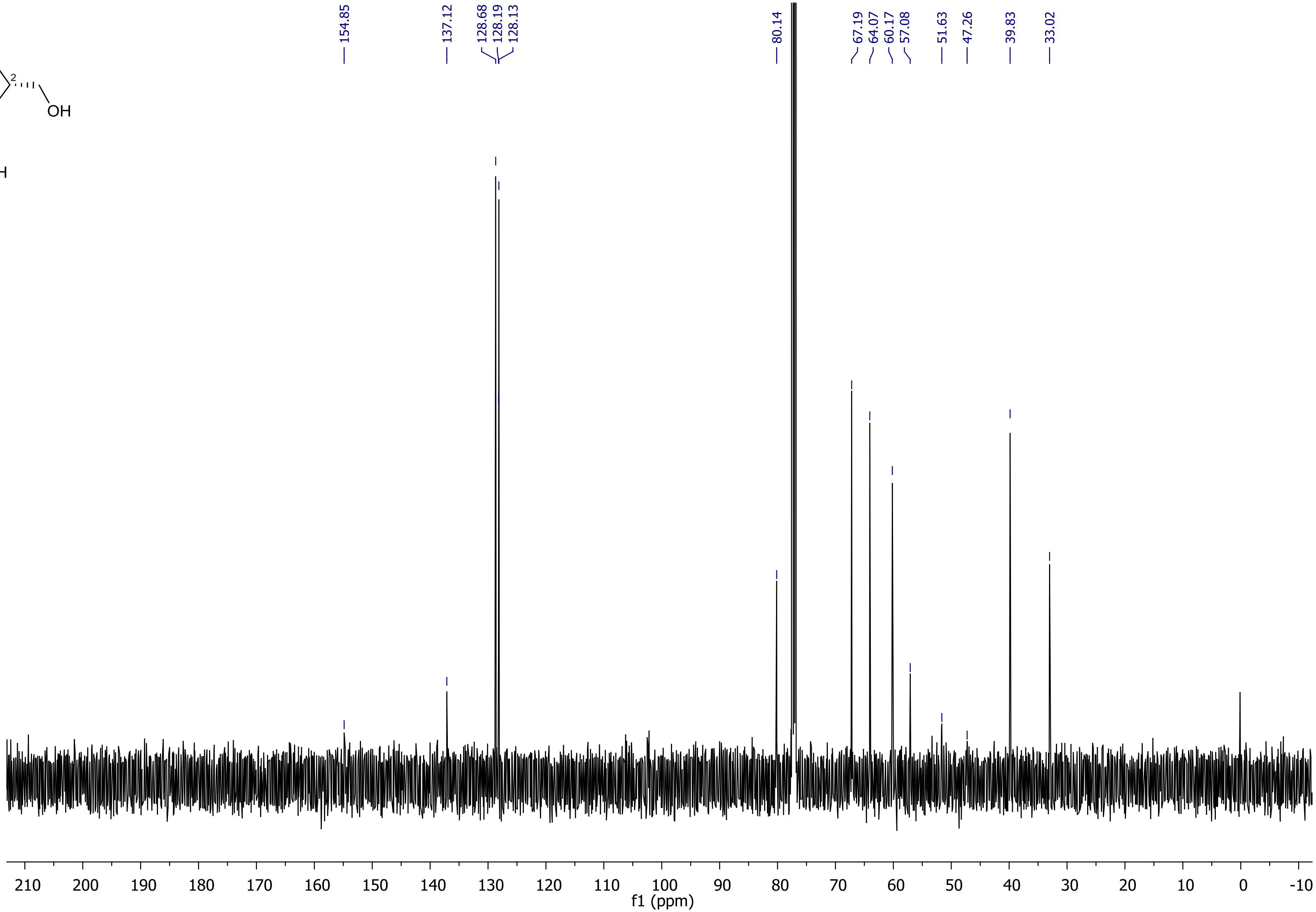

154.85
137.12
128.68
128.19
128.13
80.14
67.19
64.07
60.17
57.08
51.63
47.26
39.83
33.02
210
200
190
180
170
160
150
140
130
120
110
100
90
80
70
60
50
40
30
20
10
0
-10
f1 (ppm)

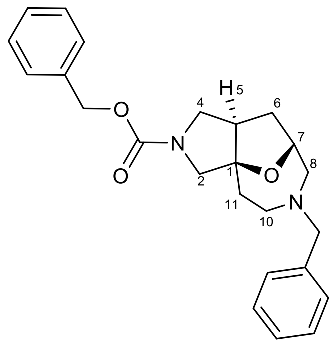

4

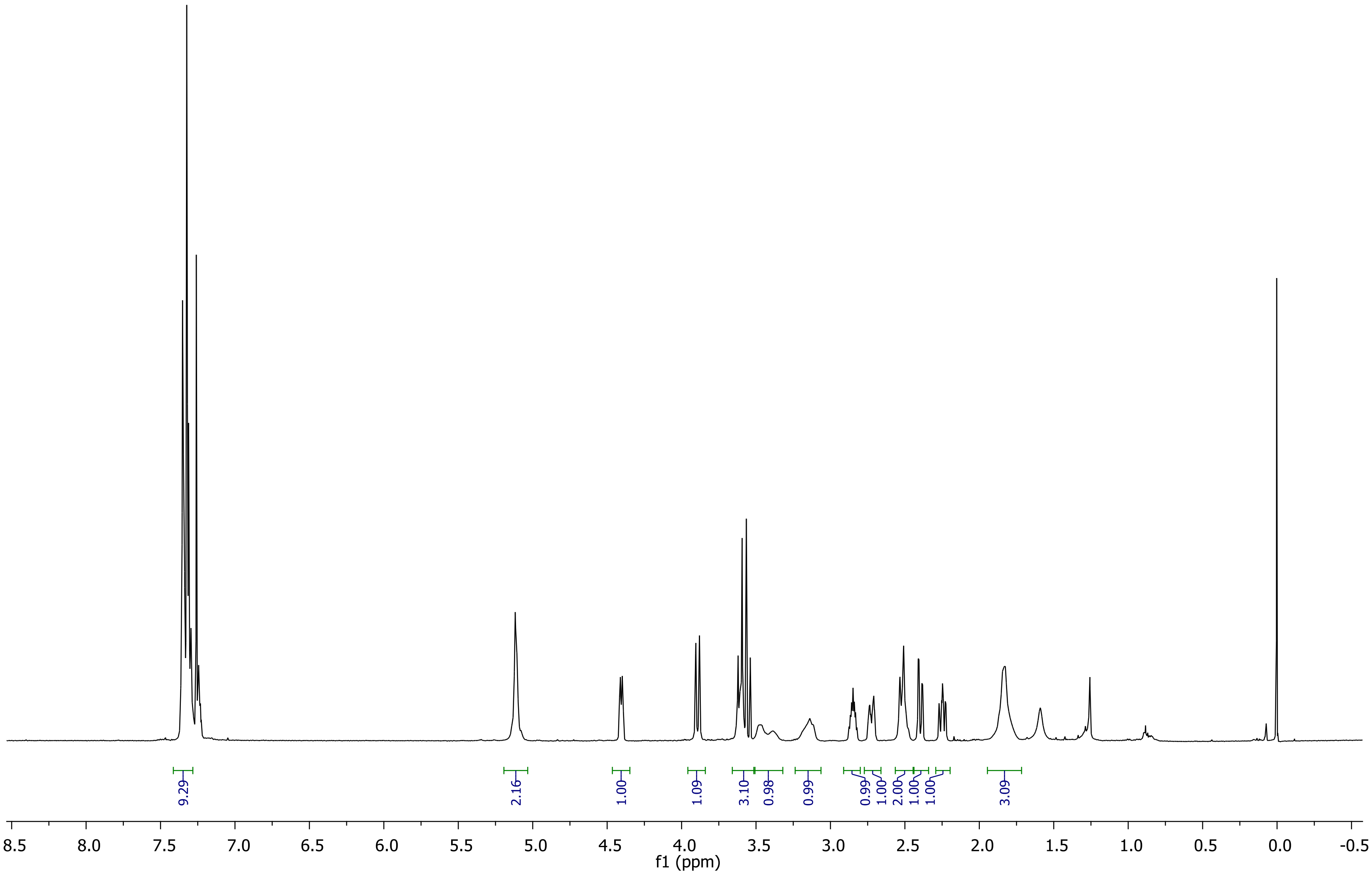

9.29
2.16
1.00
1.09
3.10
0.98
0.99
0.99
1.00
2.00
1.00
1.00
3.09
8.5 8.0 7.5 7.0 6.5 6.0 5.5 5.0 4.5 4.0 3.5 3.0 2.5 2.0 1.5 1.0 0.5 0.0 -0.5
f1 (ppm)

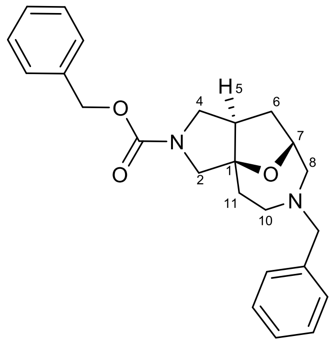

4

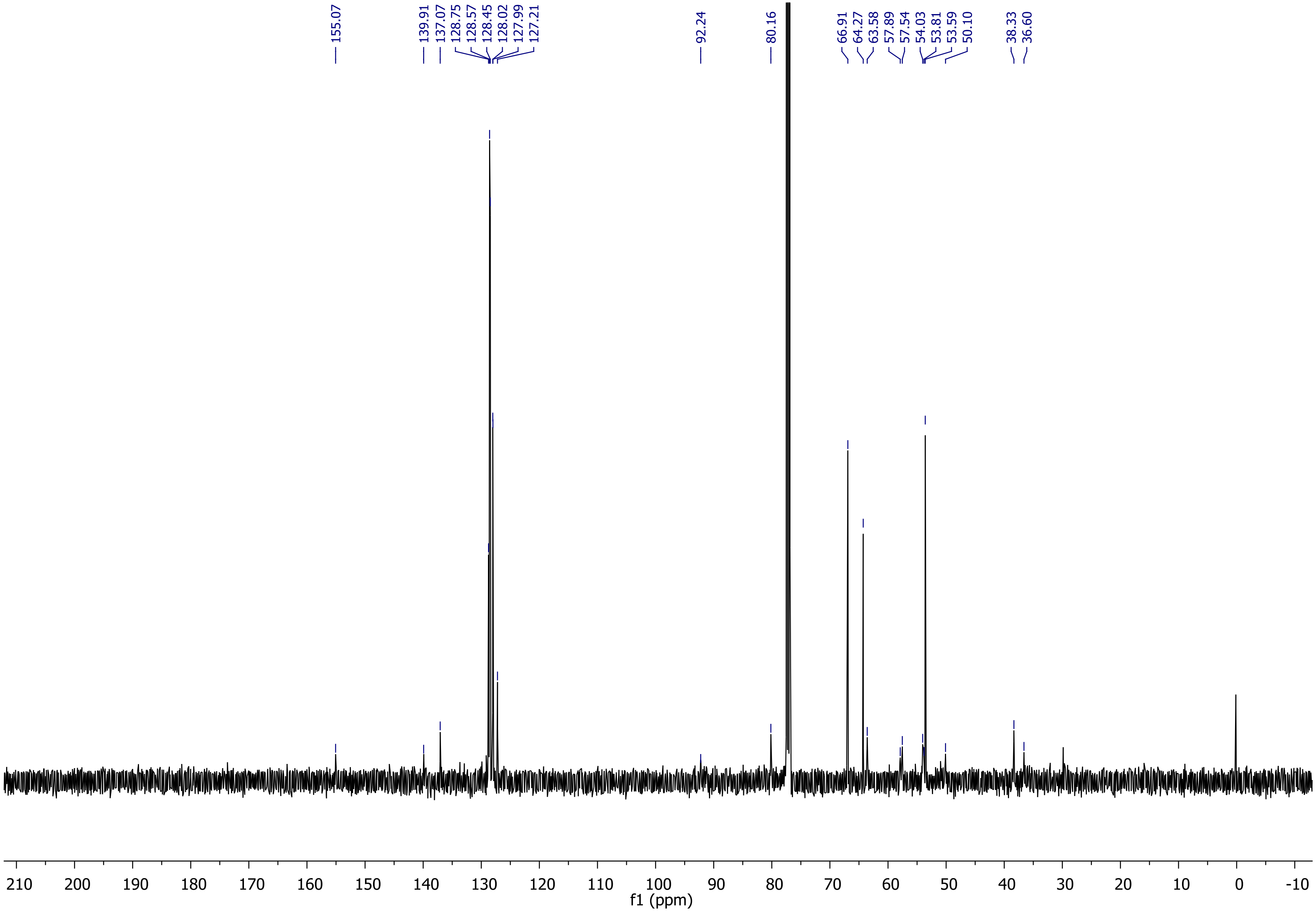

155.07
139.91
137.07
128.75
128.57
128.45
128.02
127.99
127.21
92.24
80.16
66.91
64.27
63.58
57.89
57.54
54.03
53.81
53.59
50.10
38.33
36.60
210 200 190 180 170 160 150 140 130 120 110 100 90 80 70 60 50 40 30 20 10 0 -10
f1 (ppm)

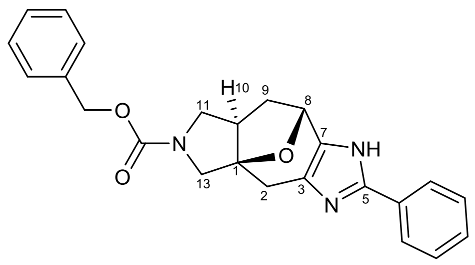

H 10
9
8
11
7
NH
N
O
O
13
1
2
3
N
5
O

5

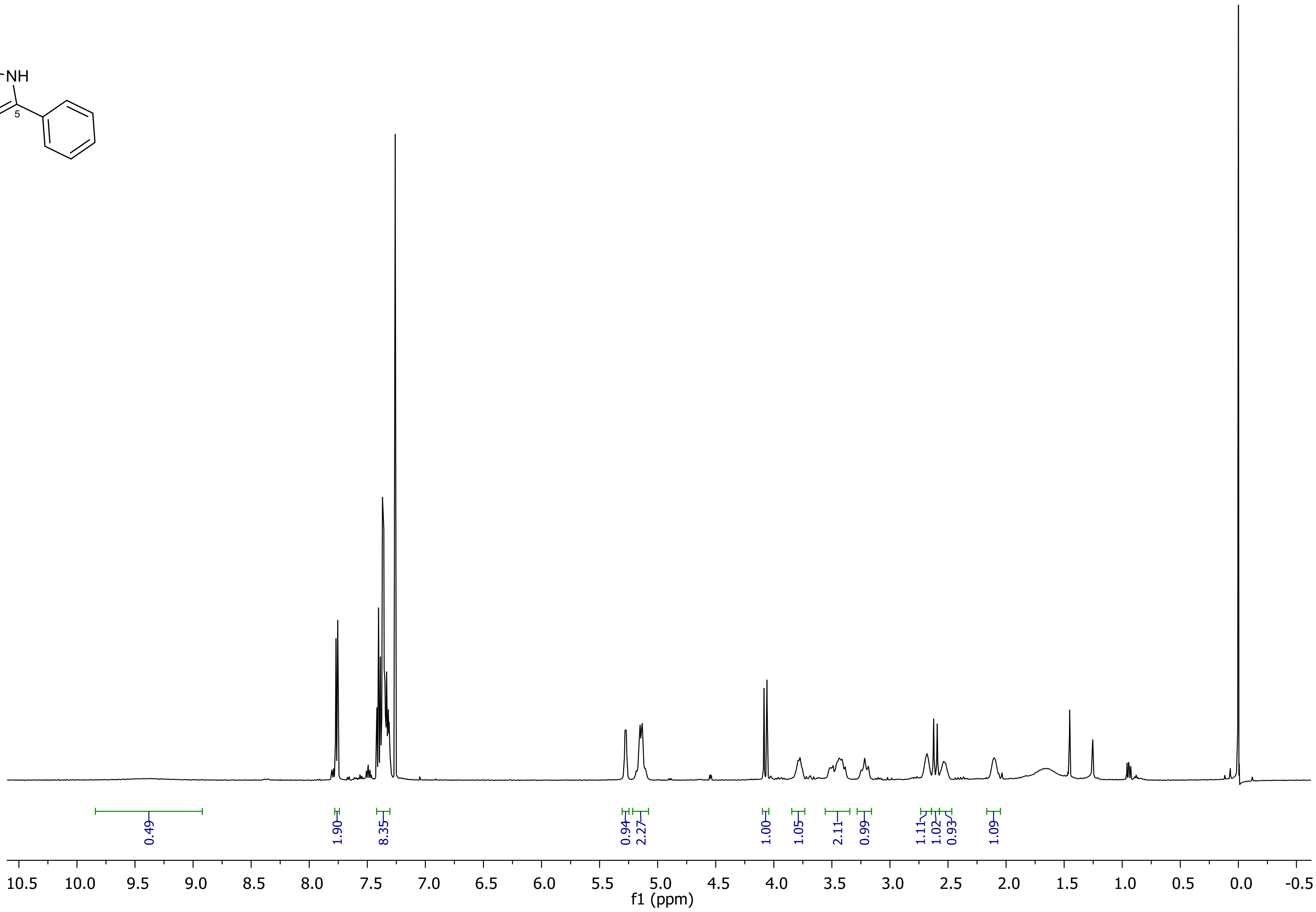

0.49
1.90
8.35
0.94
2.27
1.00
1.05
2.11
0.99
1.11
1.02
0.93
1.09
10.5 10.0 9.5 9.0 8.5 8.0 7.5 7.0 6.5 6.0 5.5 5.0 4.5 4.0 3.5 3.0 2.5 2.0 1.5 1.0 0.5 0.0 -0.5
f1 (ppm)

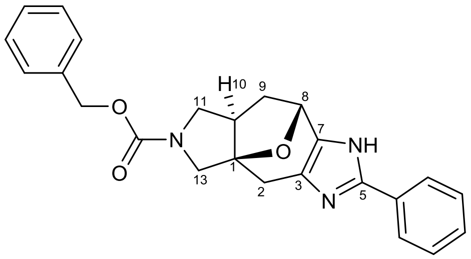
H
10
9
8
11
O
N
O
NH
7
1
13
2
3
N
5

5

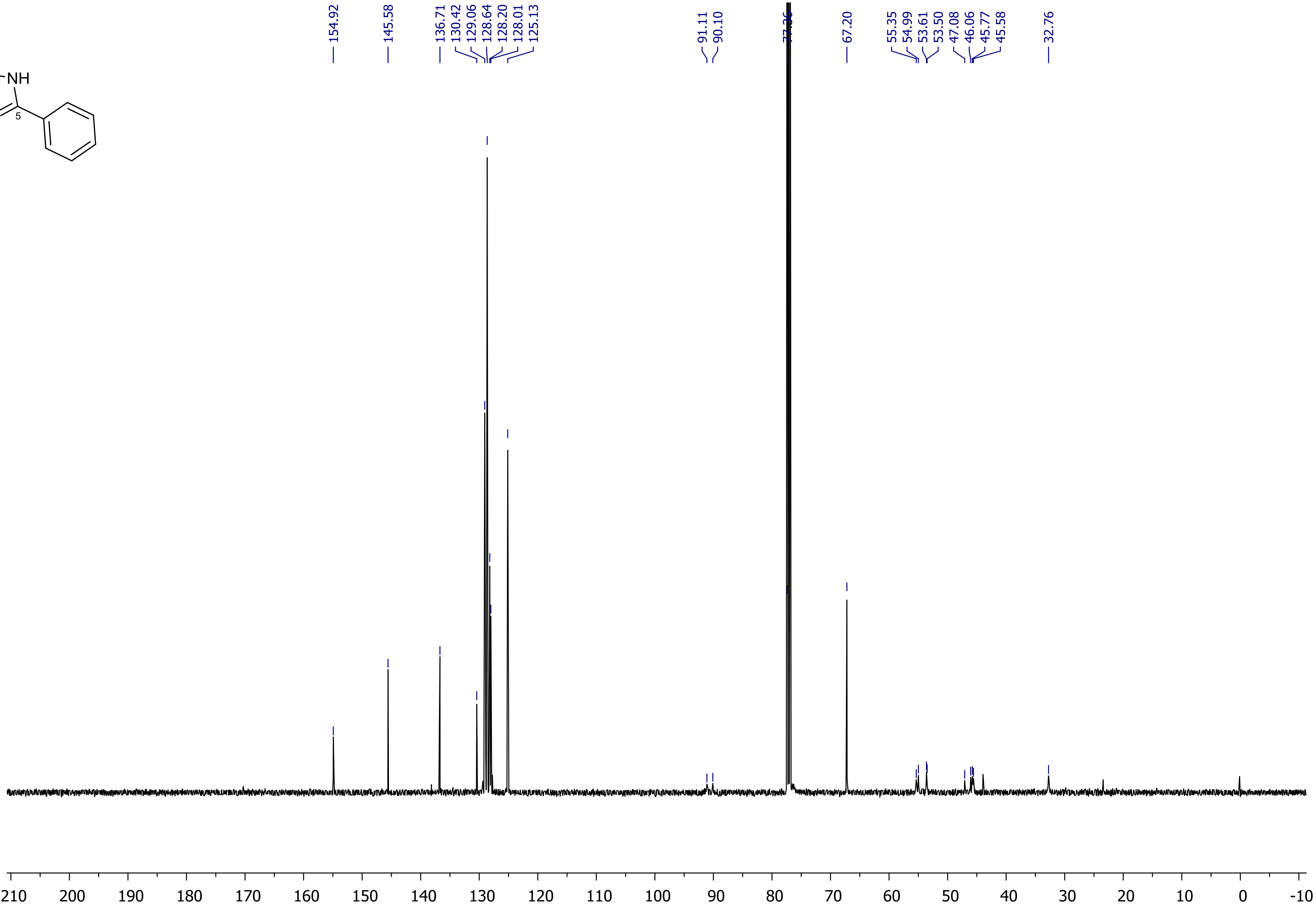
154.92
145.58
136.71
130.42
129.06
128.64
128.20
128.01
125.13
91.11
90.10
67.20
55.35
54.99
53.61
53.50
47.08
46.06
45.77
45.58
32.76
210 200 190 180 170 160 150 140 130 120 110 100 90 80 70 60 50 40 30 20 10 0 -10

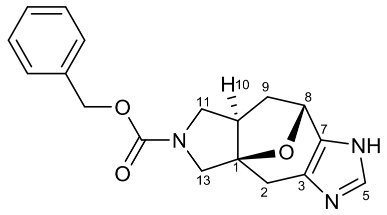
O
N
O
H
10
9
8
11
7
NH
O
13
1
2
3
N
5

S16

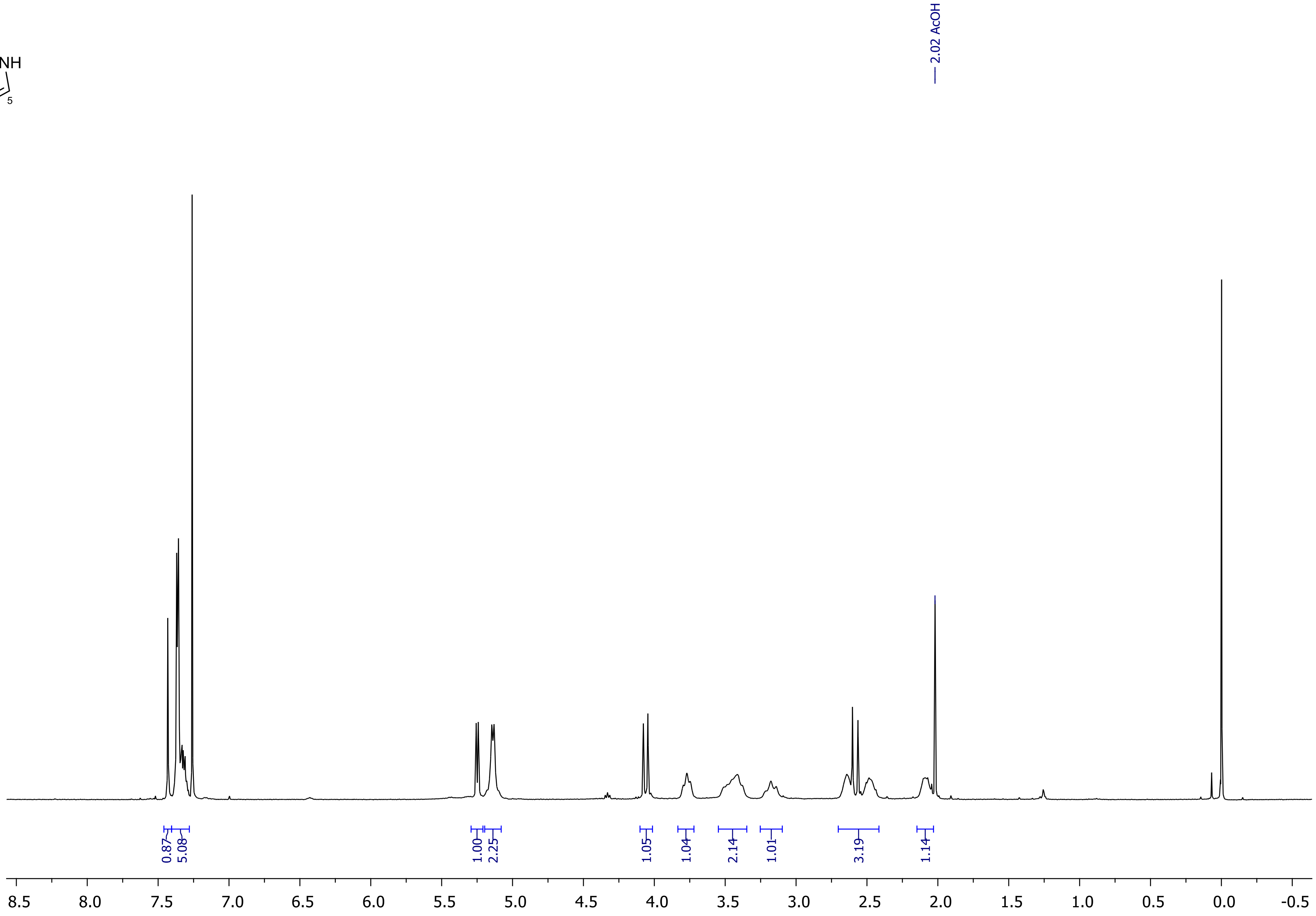
2.02 AcOH
0.87
5.08
1.00
2.25
1.05
1.04
2.14
1.01
3.19
1.14
8.5
8.0
7.5
7.0
6.5
6.0
5.5
5.0
4.5
4.0
3.5
3.0
2.5
2.0
1.5
1.0
0.5
0.0
-0.5

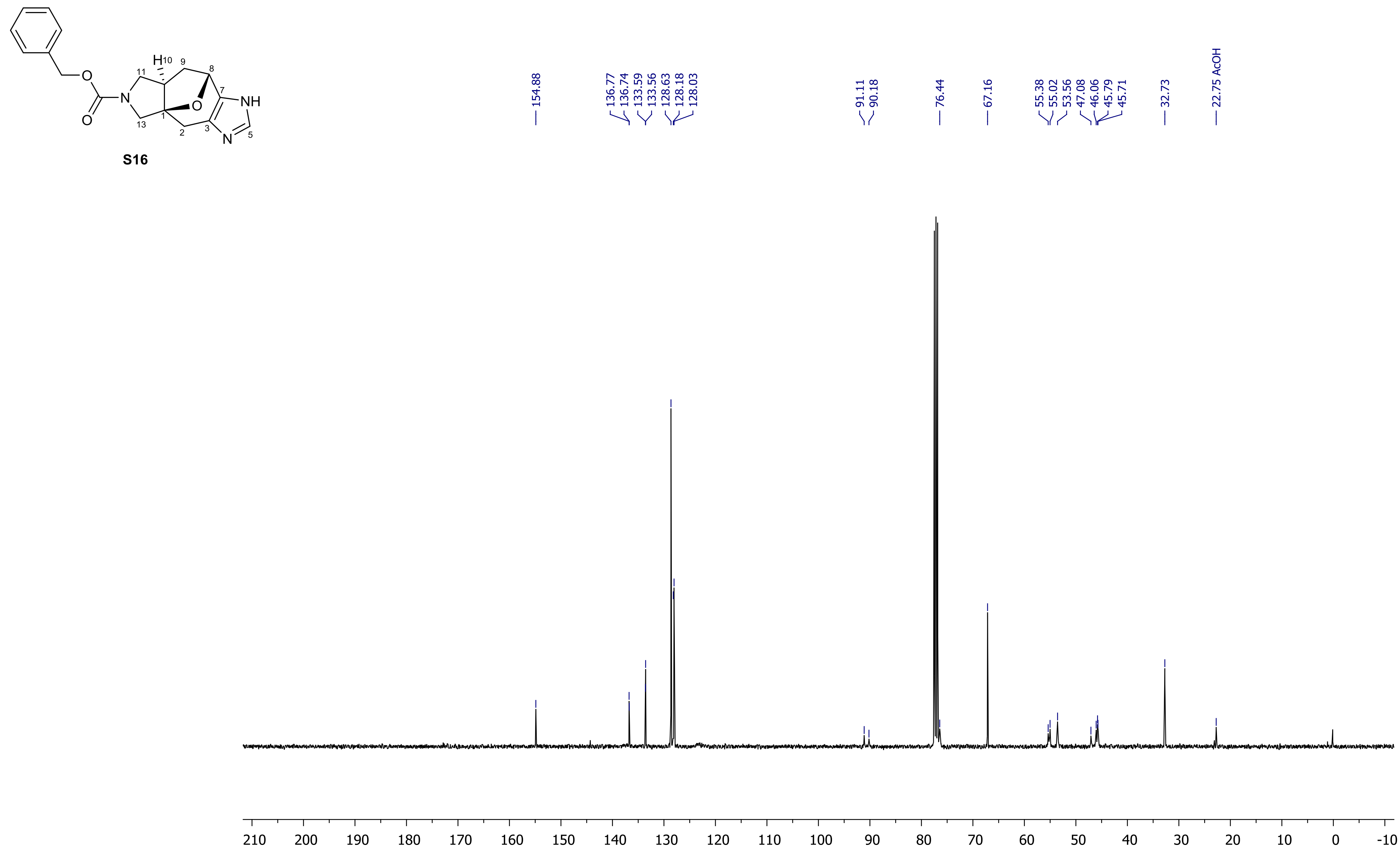

H
10
9
8
7
NH
11
N
O
O
O
13
1
2
3
N
5
154.88
136.77
136.74
133.59
133.56
128.63
128.18
128.03
91.11
90.18
76.44
67.16
55.38
55.02
53.56
47.08
46.06
45.79
45.71
32.73
22.75 AcOH
210
200
190
180
170
160
150
140
130
120
110
100
90
80
70
60
50
40
30
20
10
0
-10

S16

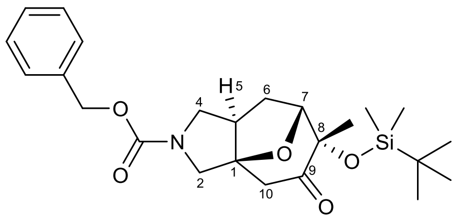

H
5
4
6
7
8
O
N
O
O
Si
O
9
O
2
1
10

S17

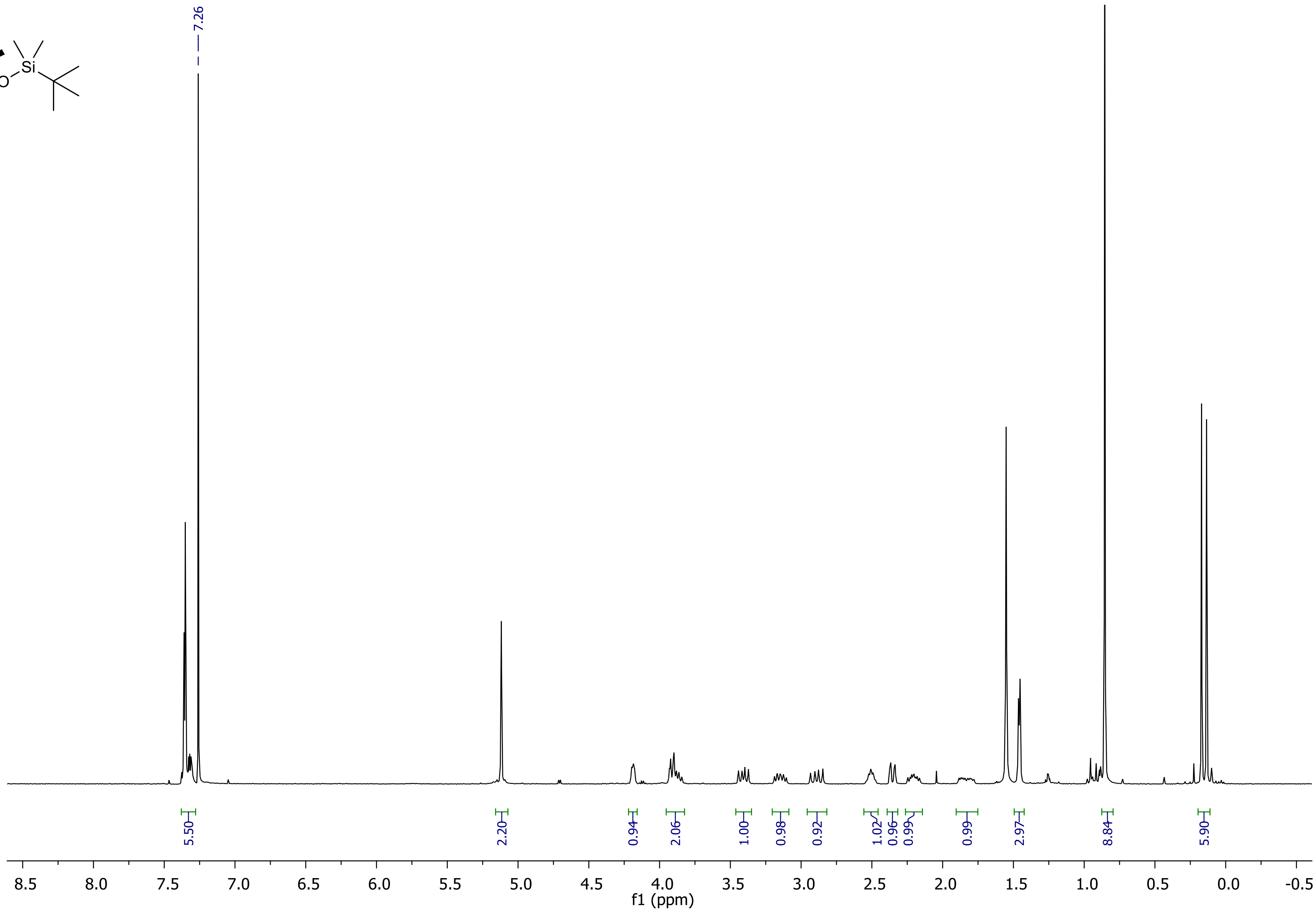

7.26
5.50
2.20
0.94
2.06
1.00
0.98
0.92
1.02
0.96
0.99
0.99
2.97
8.84
5.90
8.5 8.0 7.5 7.0 6.5 6.0 5.5 5.0 4.5 4.0 3.5 3.0 2.5 2.0 1.5 1.0 0.5 0.0 -0.5
f1 (ppm)

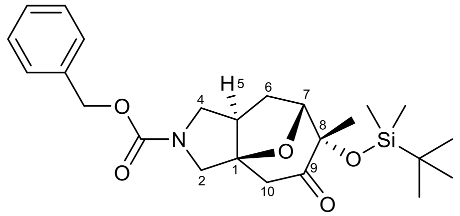

S17

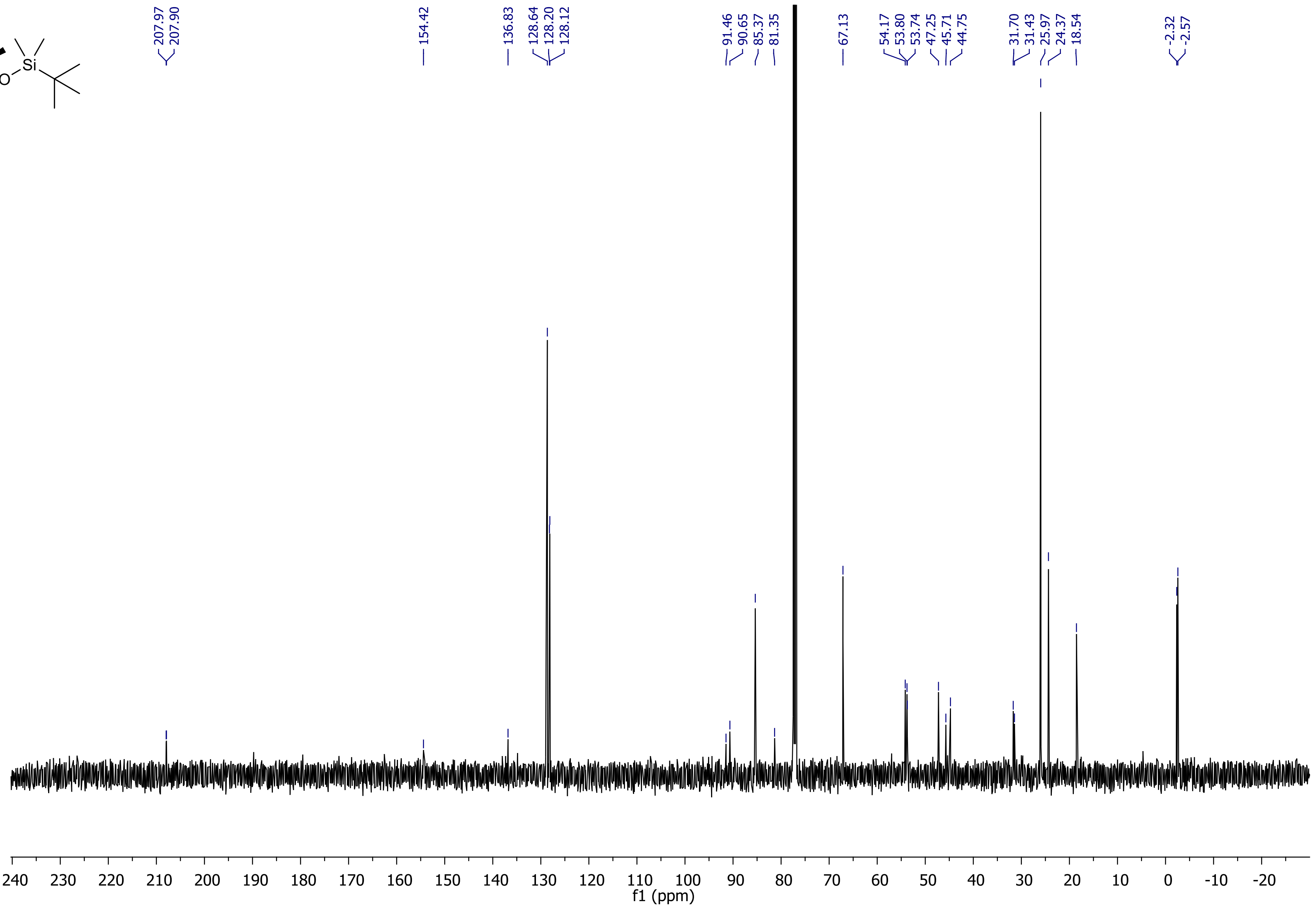
207.97
207.90
154.42
136.83
128.64
128.20
128.12
91.46
90.65
85.37
81.35
67.13
54.17
53.80
53.74
47.25
45.71
44.75
31.70
31.43
25.97
24.37
18.54
-2.32
-2.57
240 230 220 210 200 190 180 170 160 150 140 130 120 110 100 90 80 70 60 50 40 30 20 10 0 -10 -20
f1 (ppm)

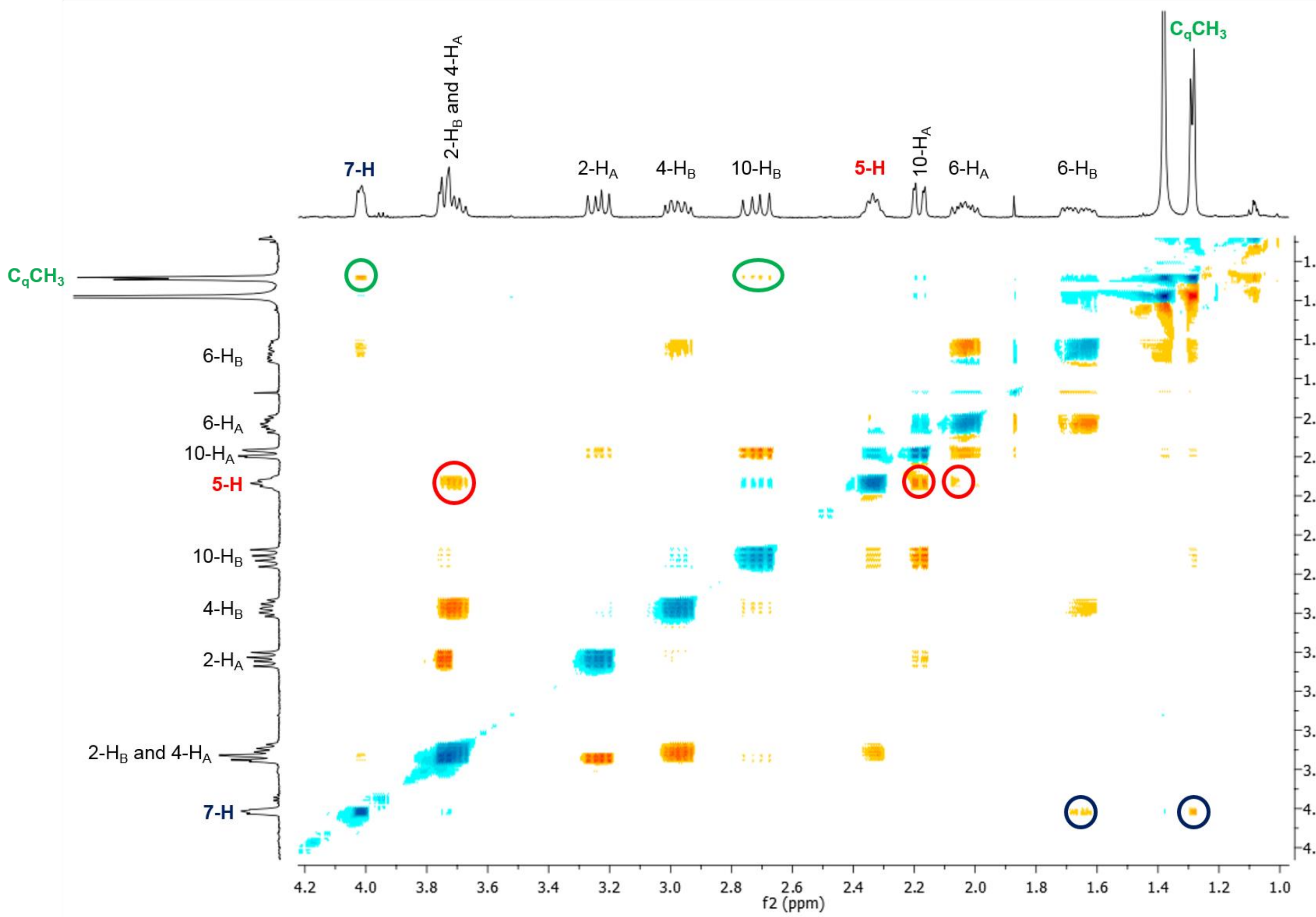

S17 NOESY correlations:

C_qCH_3: 7-H; 10-H$_B$

5-H: 4-H$_A$; 6-H$_A$; 10-H$_A$

7-H: C_qCH_3; 6-H$_B$

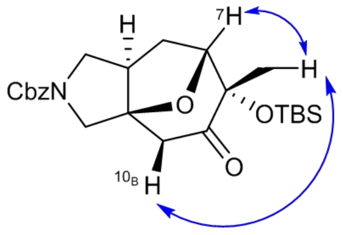

C_qCH_3

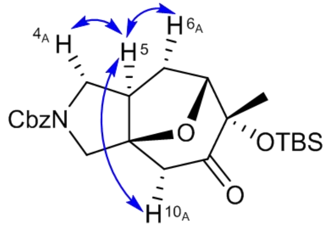

5-H

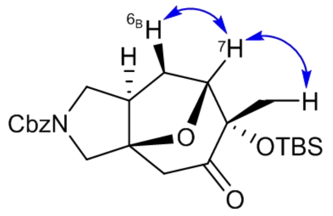

7-H

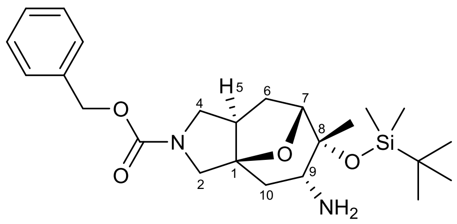

6

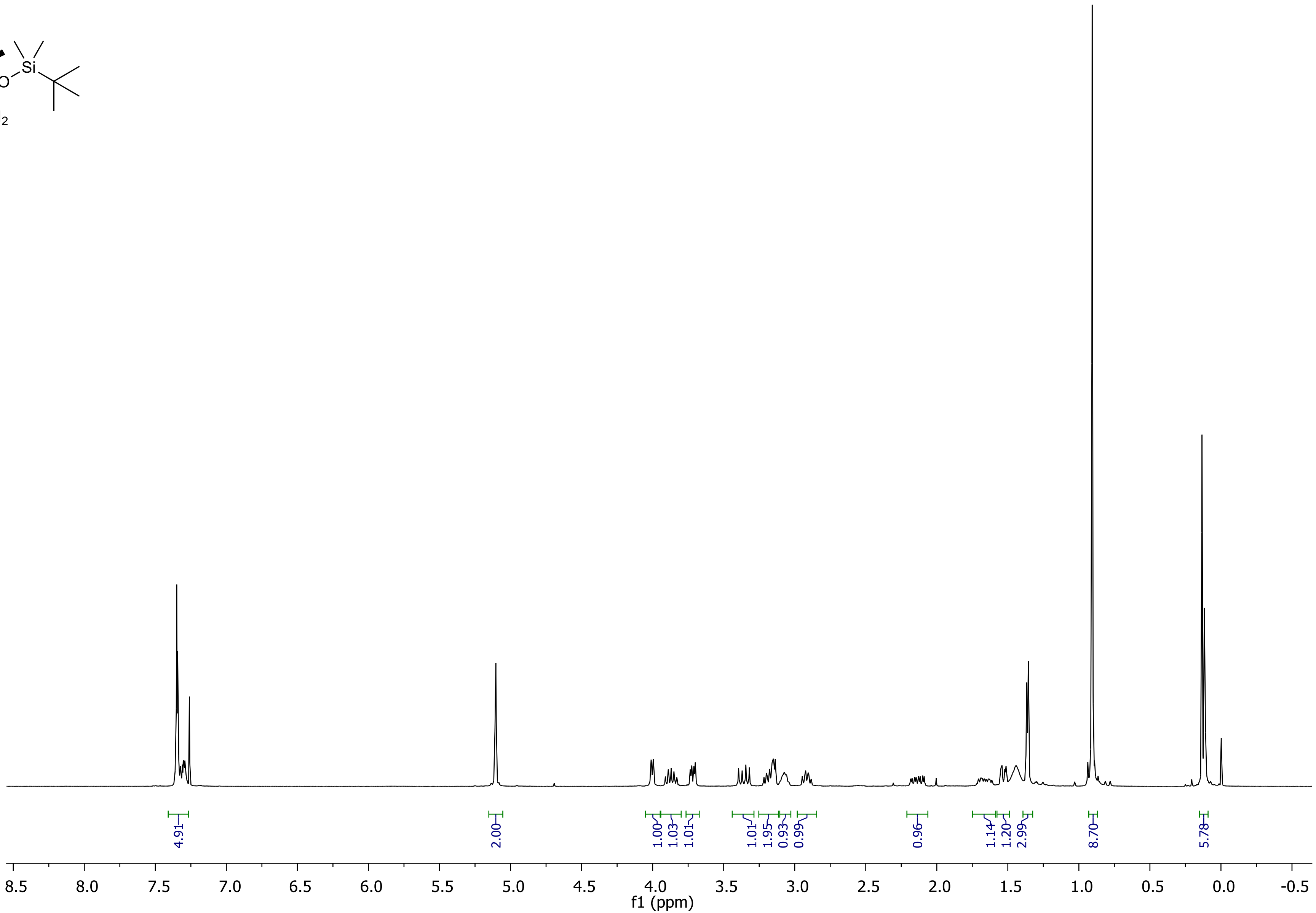

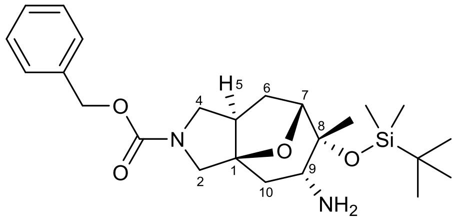

6

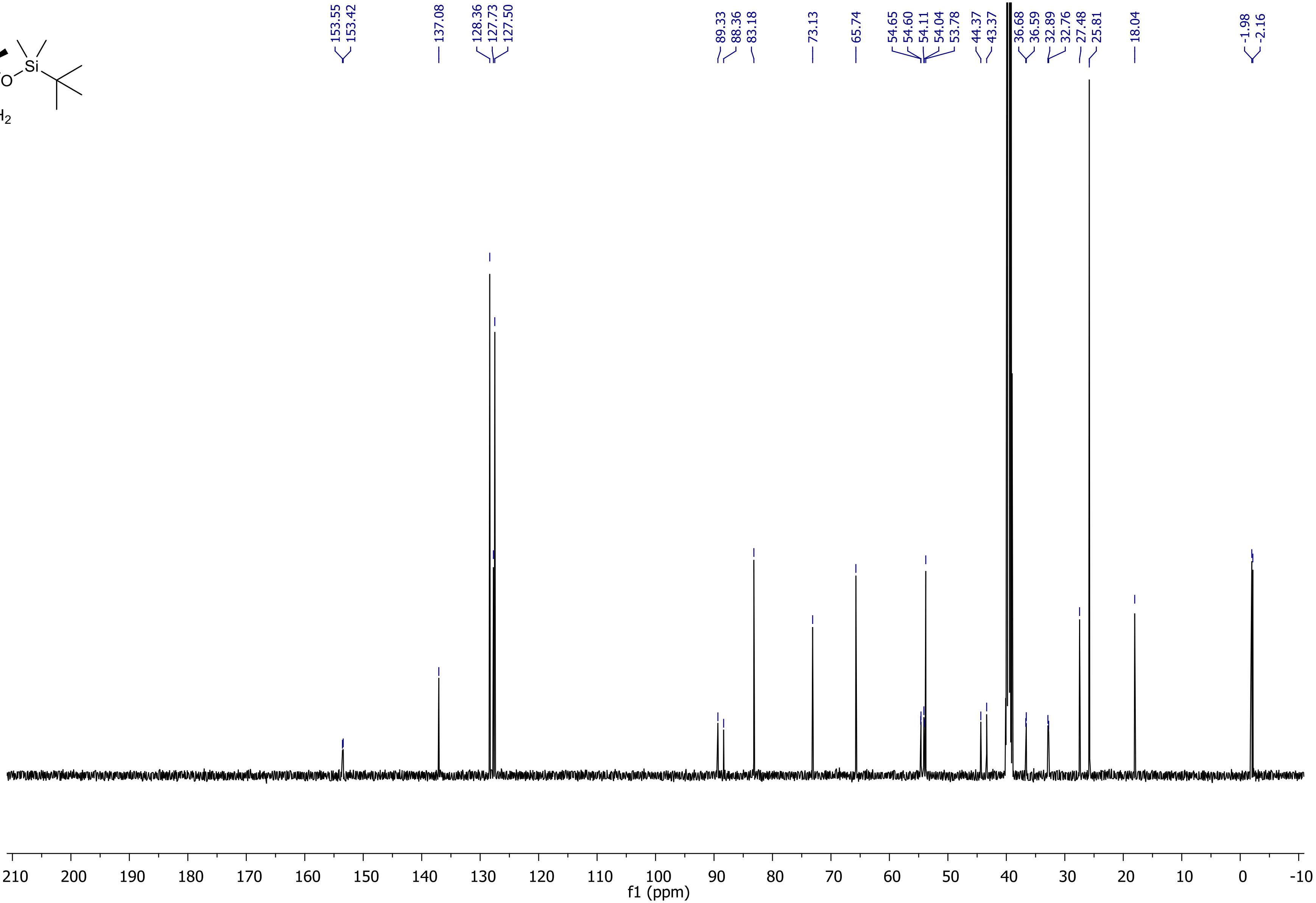

153.55
153.42
137.08
128.36
127.73
127.50
89.33
88.36
83.18
73.13
65.74
54.65
54.60
54.11
54.04
53.78
44.37
43.37
36.68
36.59
32.89
32.76
27.48
25.81
18.04
-1.98
-2.16
f1 (ppm)

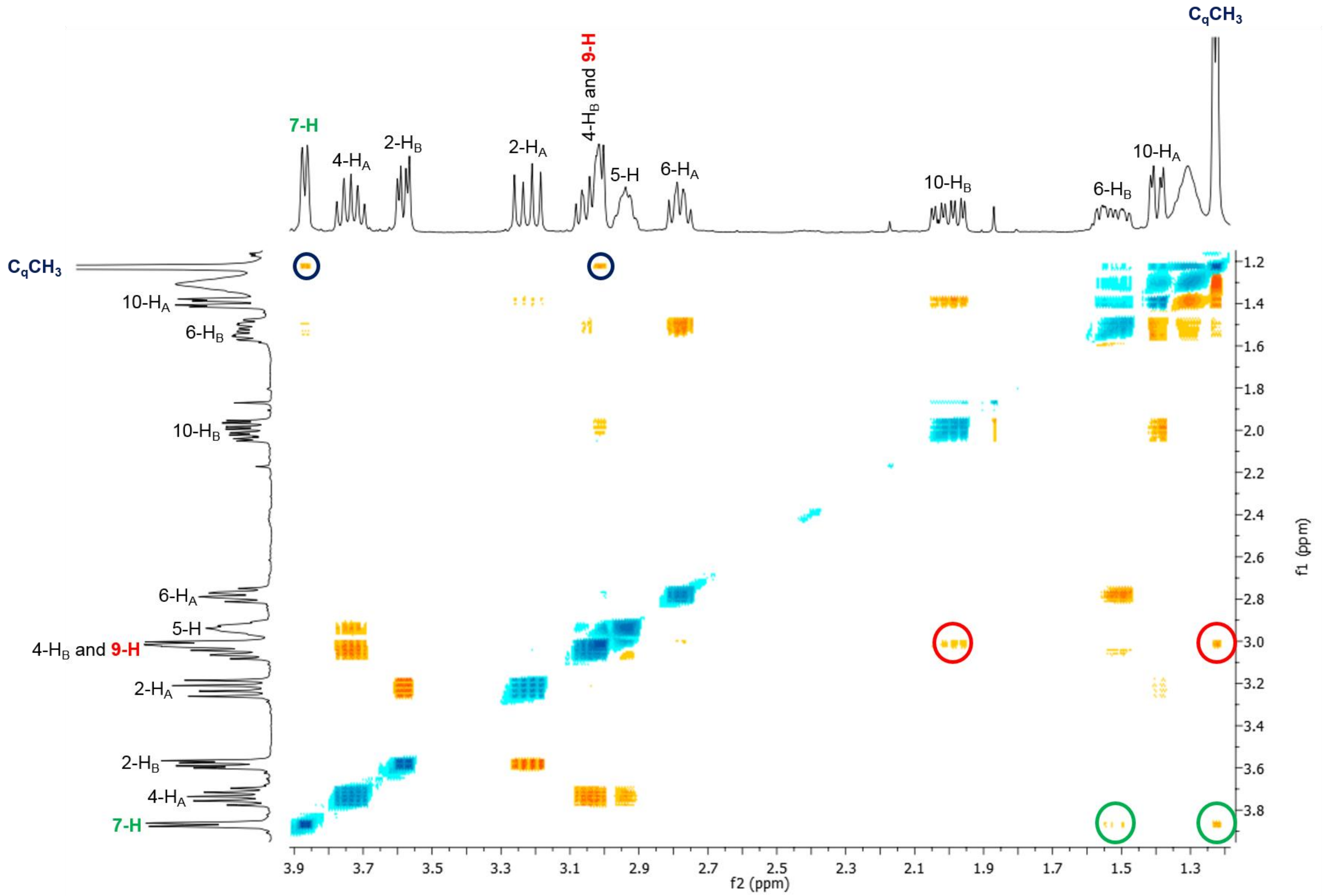

6 NOESY correlations:

Me: 7-H; 9-H

7-H: Me; $6\text{-}H_B$

9-H: Me; $10\text{-}H_B$

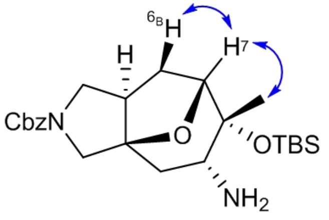

7-H

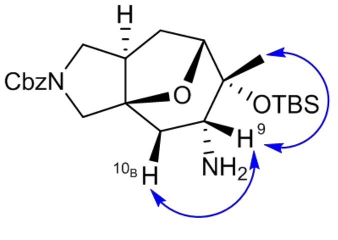

9-H

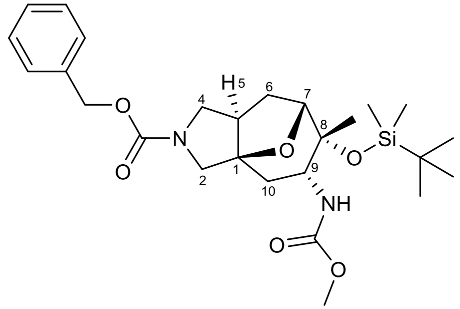

O
N
O
H 5
4
6
7
8
O
Si
O
1
2
10
9
NH
O
O

S18

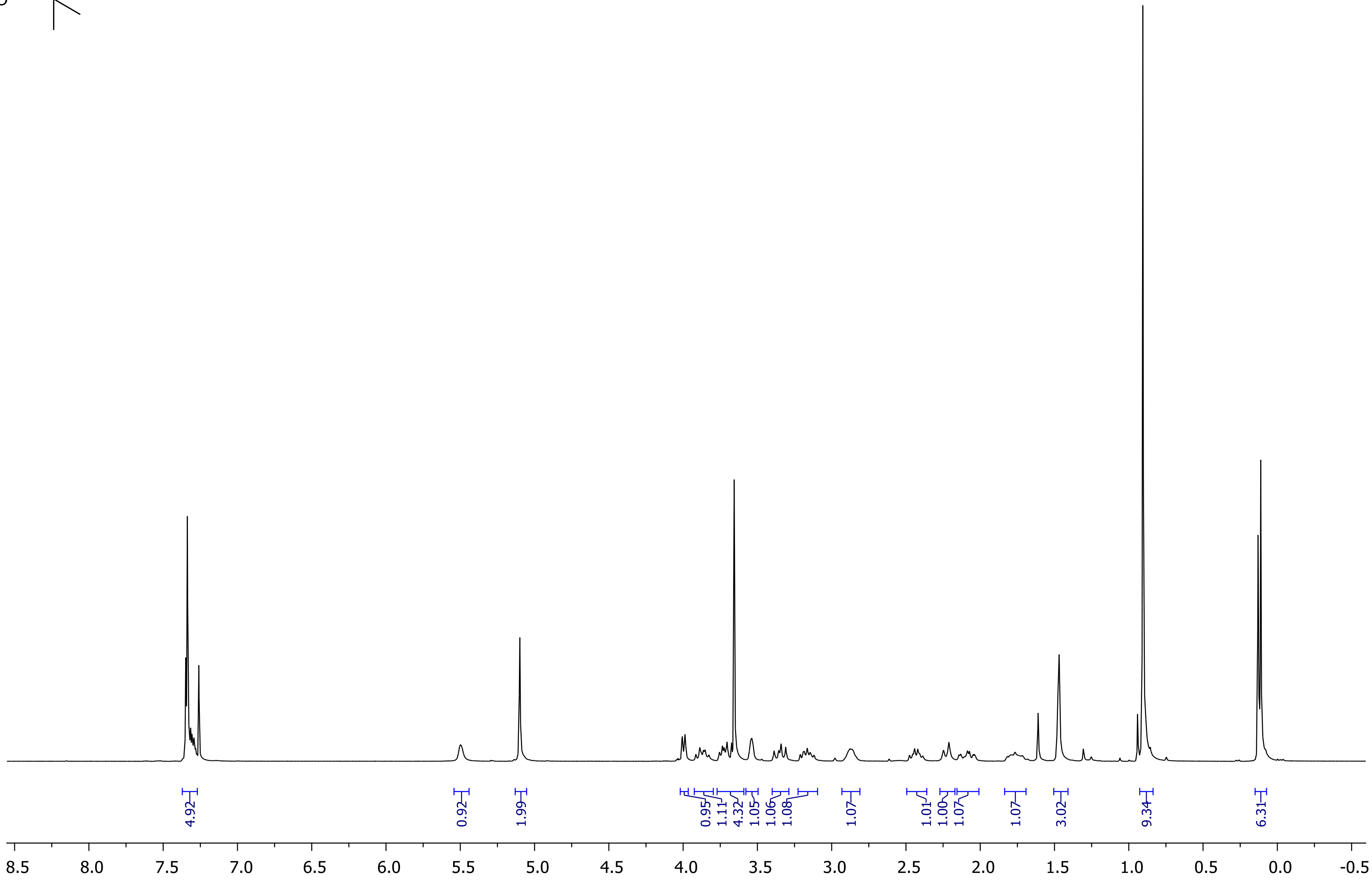

4.92
0.92
1.99
0.95
1.11
4.32
1.05
1.06
1.08
1.07
1.01
1.00
1.07
1.07
3.02
9.34
6.31
8.5
8.0
7.5
7.0
6.5
6.0
5.5
5.0
4.5
4.0
3.5
3.0
2.5
2.0
1.5
1.0
0.5
0.0
-0.5

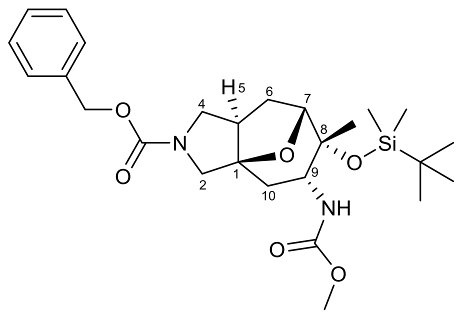

S18

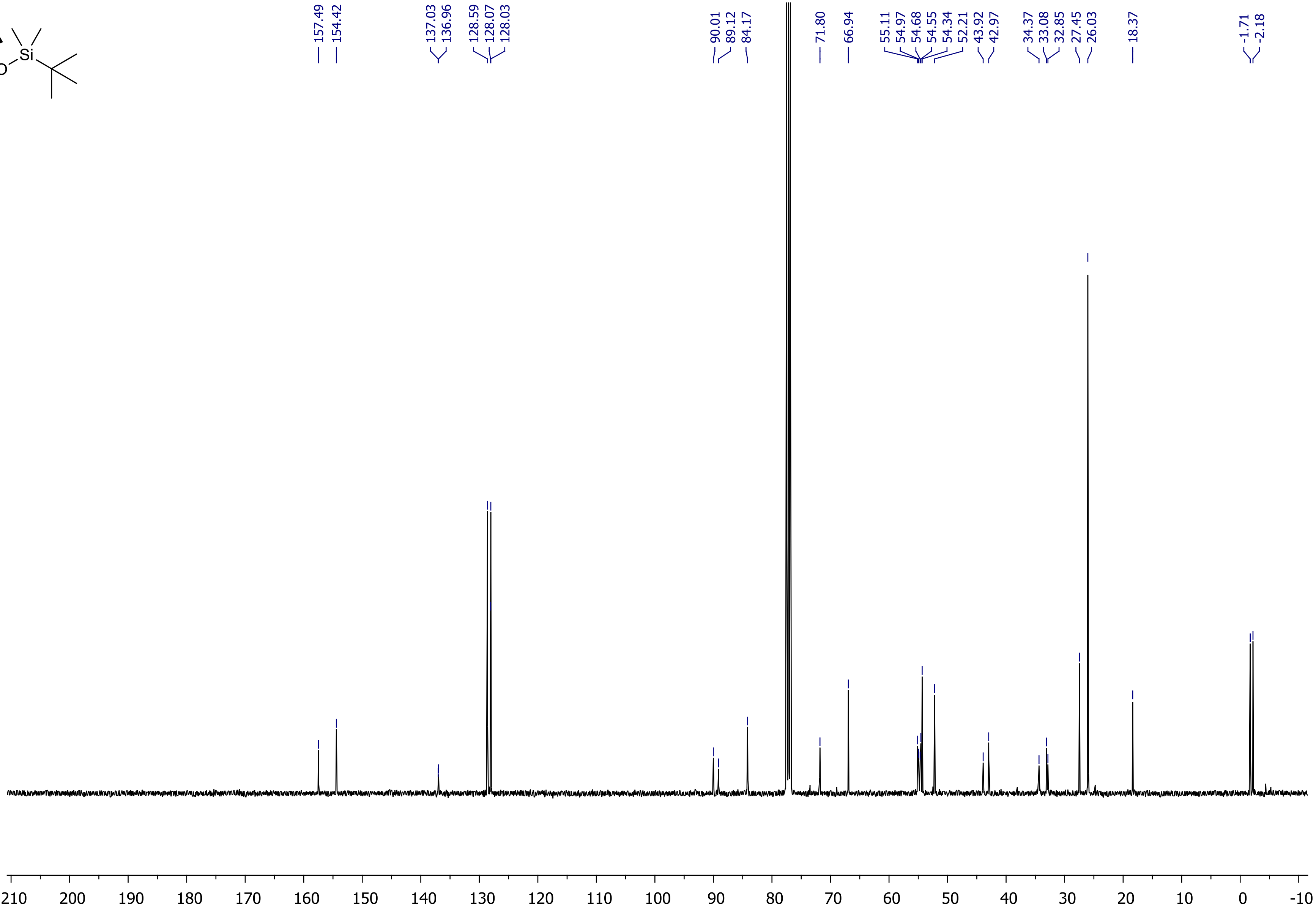
157.49
154.42
137.03
136.96
128.59
128.07
128.03
90.01
89.12
84.17
71.80
66.94
55.11
54.97
54.68
54.55
54.34
52.21
43.92
42.97
34.37
33.08
32.85
27.45
26.03
18.37
-1.71
-2.18
210 200 190 180 170 160 150 140 130 120 110 100 90 80 70 60 50 40 30 20 10 0 -10

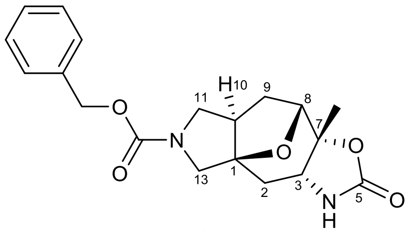
H
10
9
8
11
7
O
O
O
N
O
13
1
2
3
N
H
5
O

8

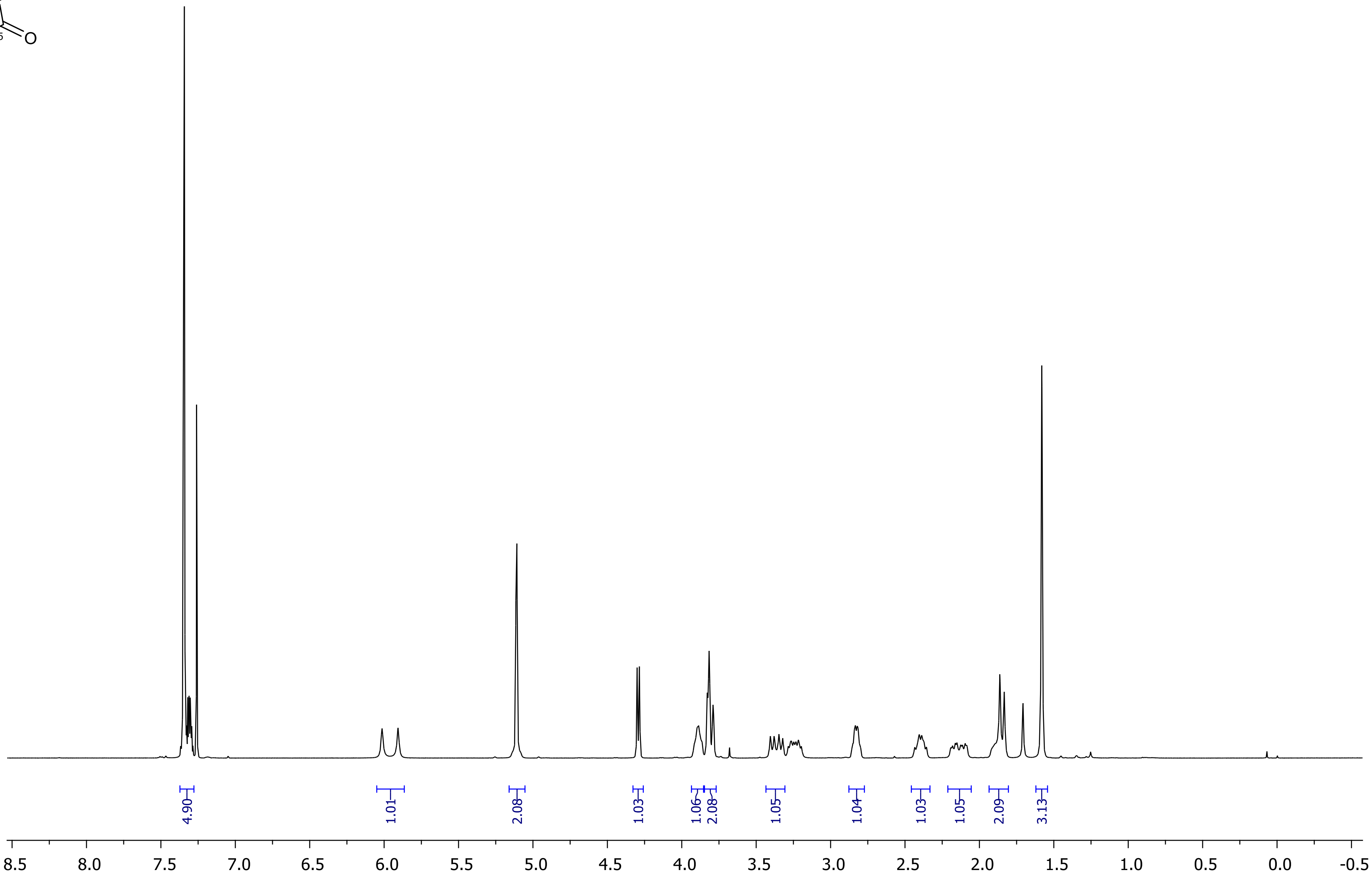
4.90
1.01
2.08
1.03
1.06
2.08
1.05
1.04
1.03
1.05
2.09
3.13
8.5
8.0
7.5
7.0
6.5
6.0
5.5
5.0
4.5
4.0
3.5
3.0
2.5
2.0
1.5
1.0
0.5
0.0
-0.5

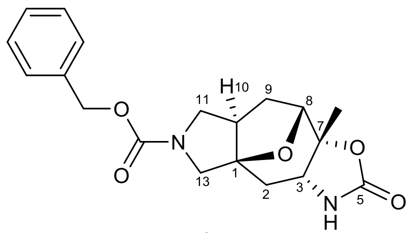

8

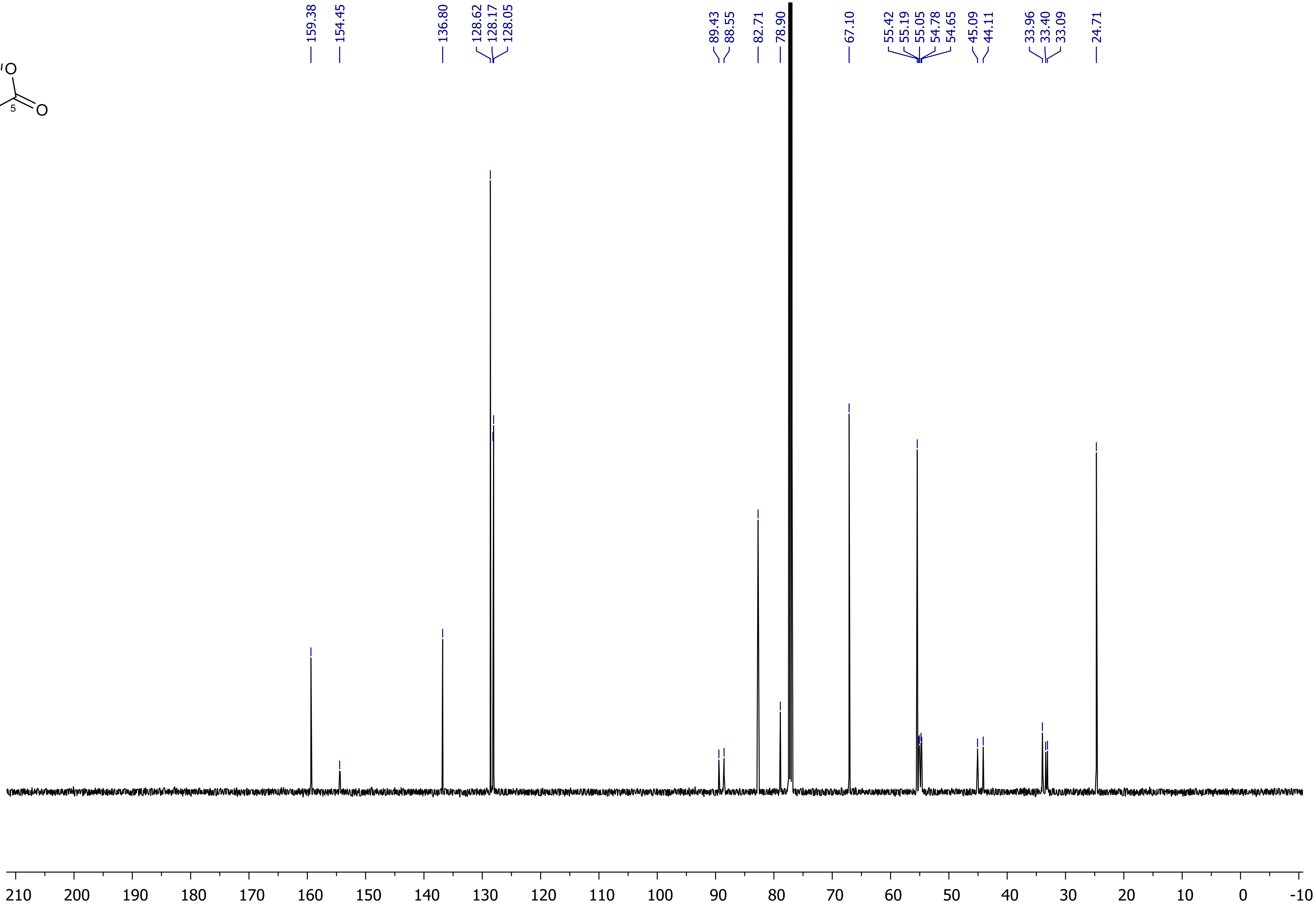

159.38
154.45
136.80
128.62
128.17
128.05
89.43
88.55
82.71
78.90
67.10
55.42
55.19
55.05
54.78
54.65
45.09
44.11
33.96
33.40
33.09
24.71
210 200 190 180 170 160 150 140 130 120 110 100 90 80 70 60 50 40 30 20 10 0 -10

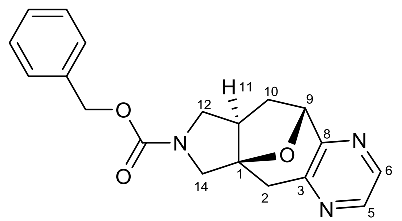
H
11
10
9
12
O
N
8
N
O
6
O
1
3
14
2
N
5

9

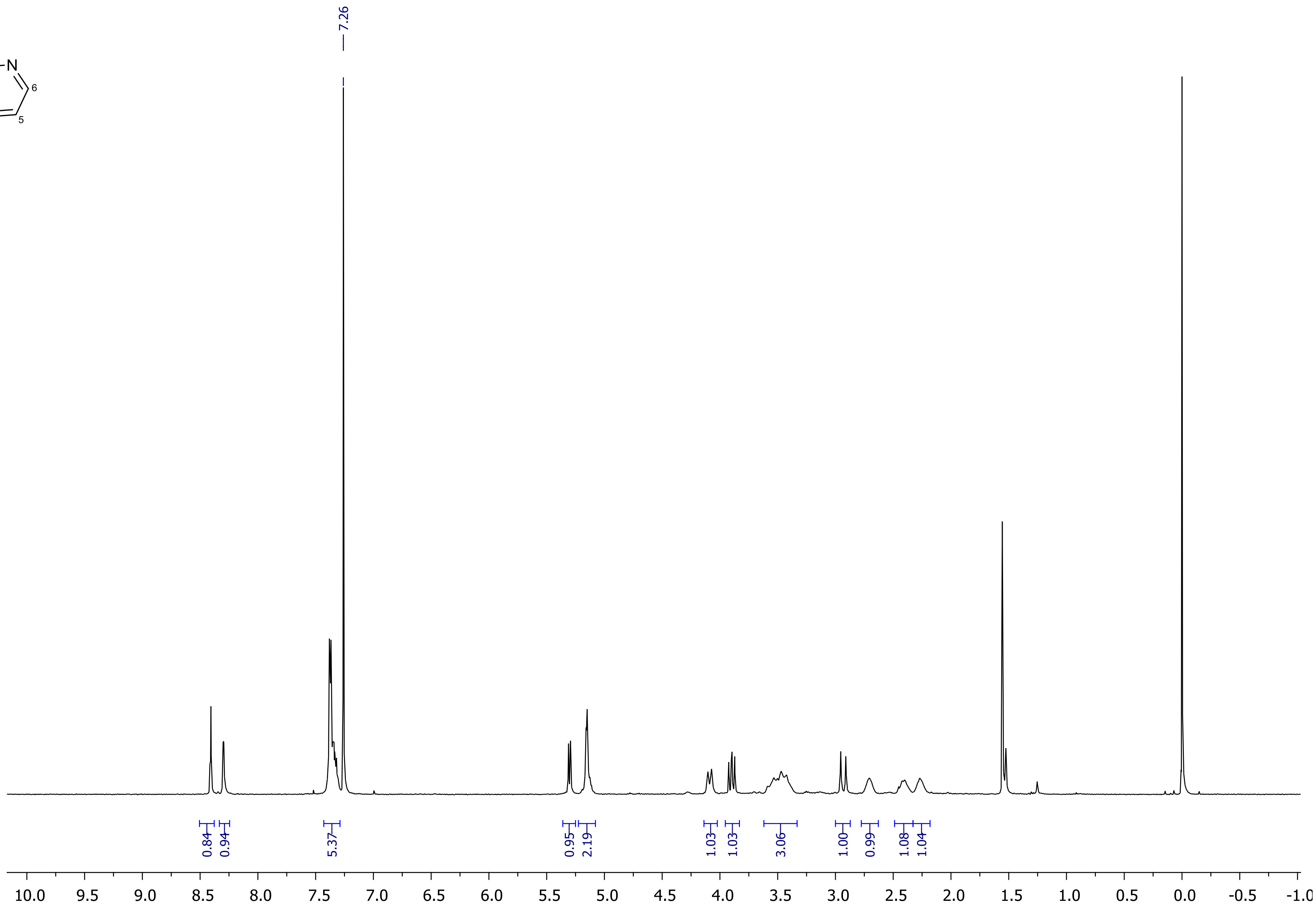
7.26
0.84
0.94
5.37
0.95
2.19
1.03
1.03
3.06
1.00
0.99
1.08
1.04
10.0
9.5
9.0
8.5
8.0
7.5
7.0
6.5
6.0
5.5
5.0
4.5
4.0
3.5
3.0
2.5
2.0
1.5
1.0
0.5
0.0
-0.5
-1.0

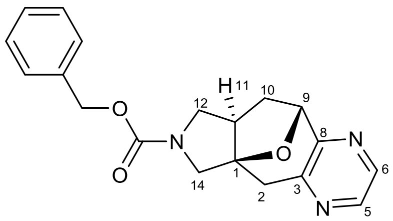

H
11
10
9
12
8
N
O
N
O
6
O
14
1
2
3
N
5

9

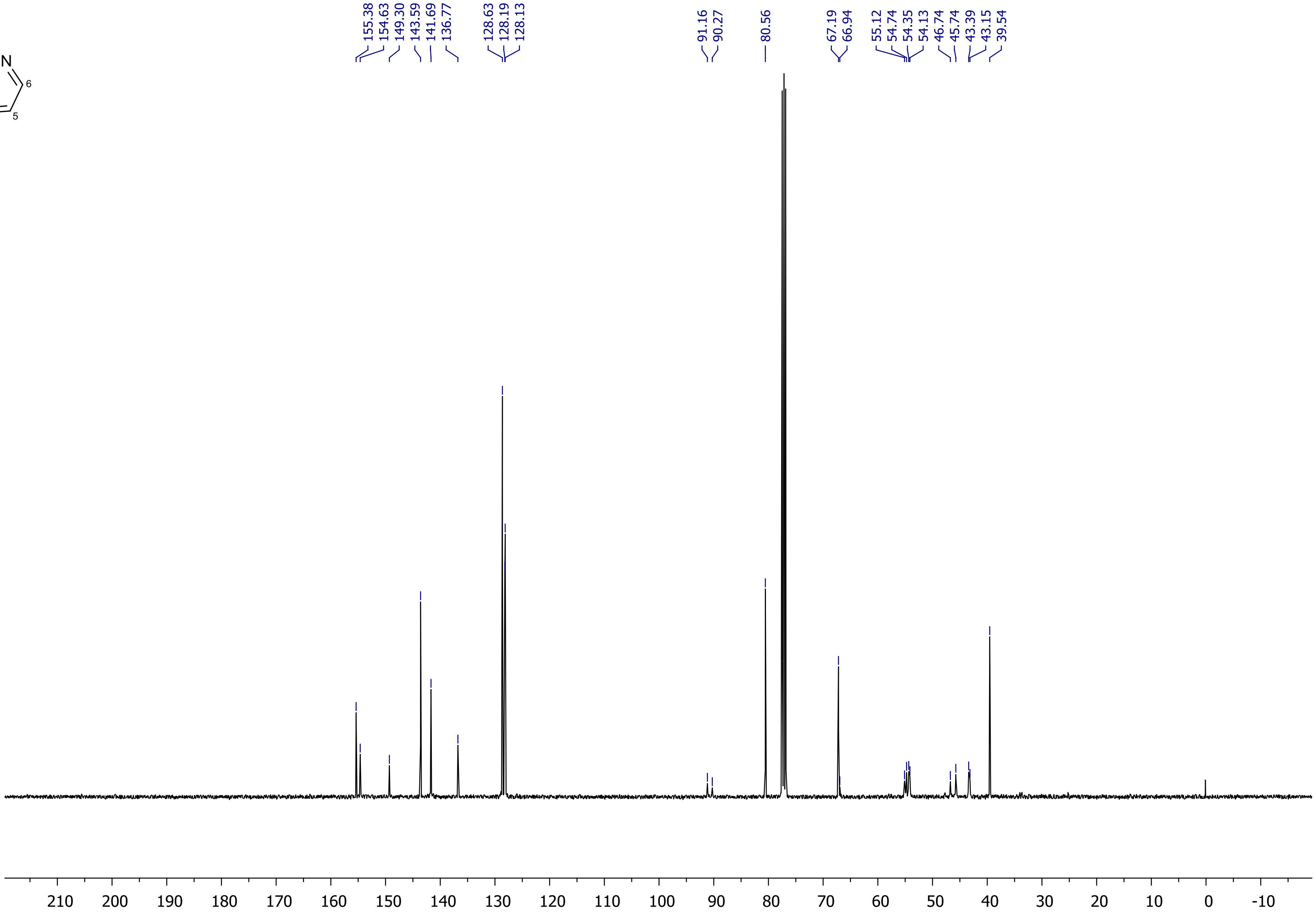

155.38
154.63
149.30
143.59
141.69
136.77
128.63
128.19
128.13
91.16
90.27
80.56
67.19
66.94
55.12
54.74
54.35
54.13
46.74
45.74
43.39
43.15
39.54
210
200
190
180
170
160
150
140
130
120
110
100
90
80
70
60
50
40
30
20
10
0
-10

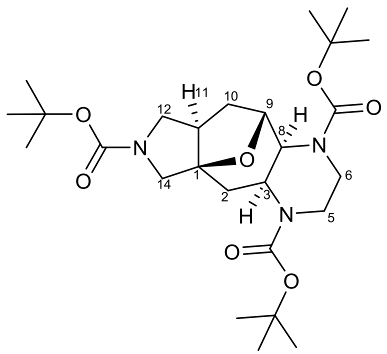

S19

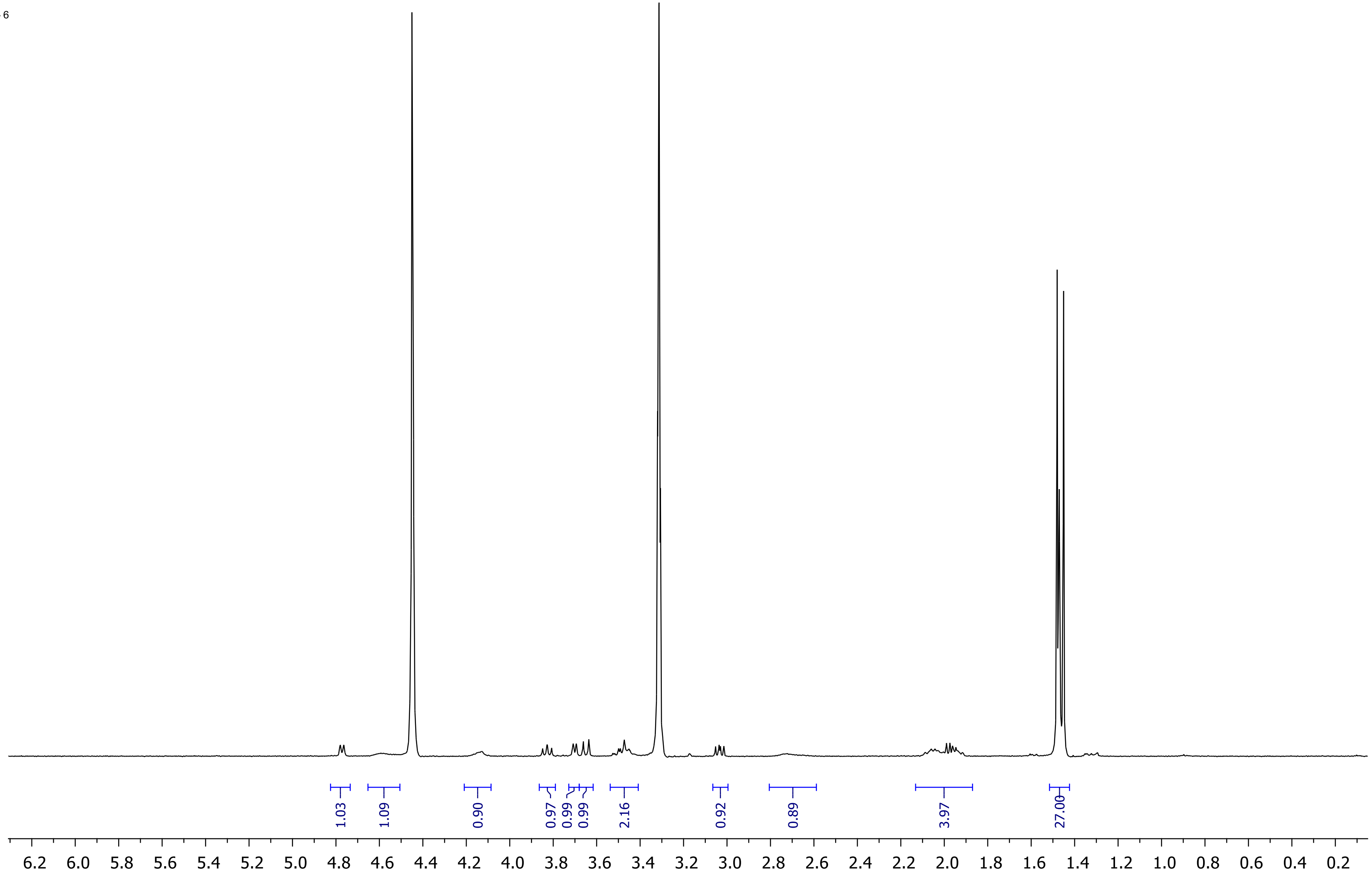

1.03
1.09
0.90
0.97
0.99
0.99
2.16
0.92
0.89
3.97
27.00
6.2 6.0 5.8 5.6 5.4 5.2 5.0 4.8 4.6 4.4 4.2 4.0 3.8 3.6 3.4 3.2 3.0 2.8 2.6 2.4 2.2 2.0 1.8 1.6 1.4 1.2 1.0 0.8 0.6 0.4 0.2

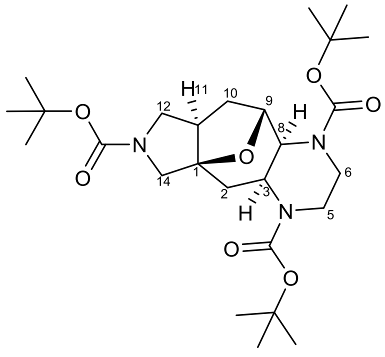

O
O
N
H
O
N
N
O
O
O
O
H
H
11
10
9
8
6
5
3
2
1
14
12

S19

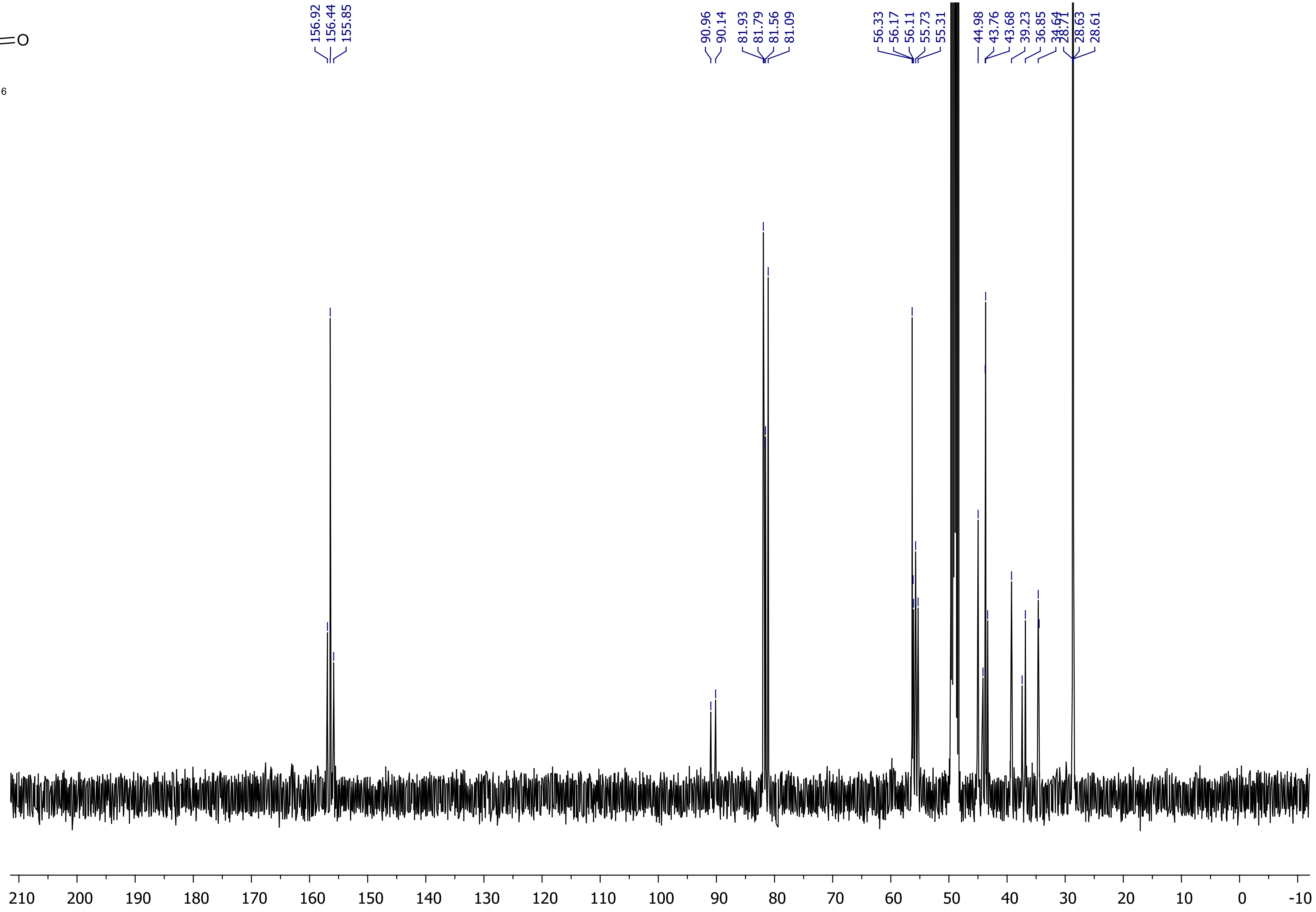

156.92
156.44
155.85
90.96
90.14
81.93
81.79
81.56
81.09
56.33
56.17
56.11
55.73
55.31
44.98
43.76
43.68
39.23
36.85
34.64
28.71
28.63
28.61
210
200
190
180
170
160
150
140
130
120
110
100
90
80
70
60
50
40
30
20
10
0
-10

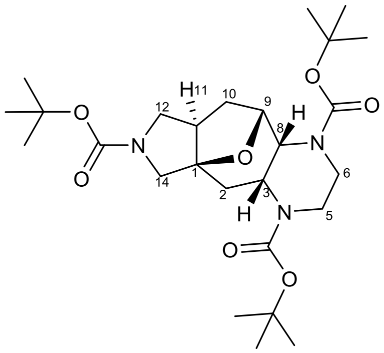

S20

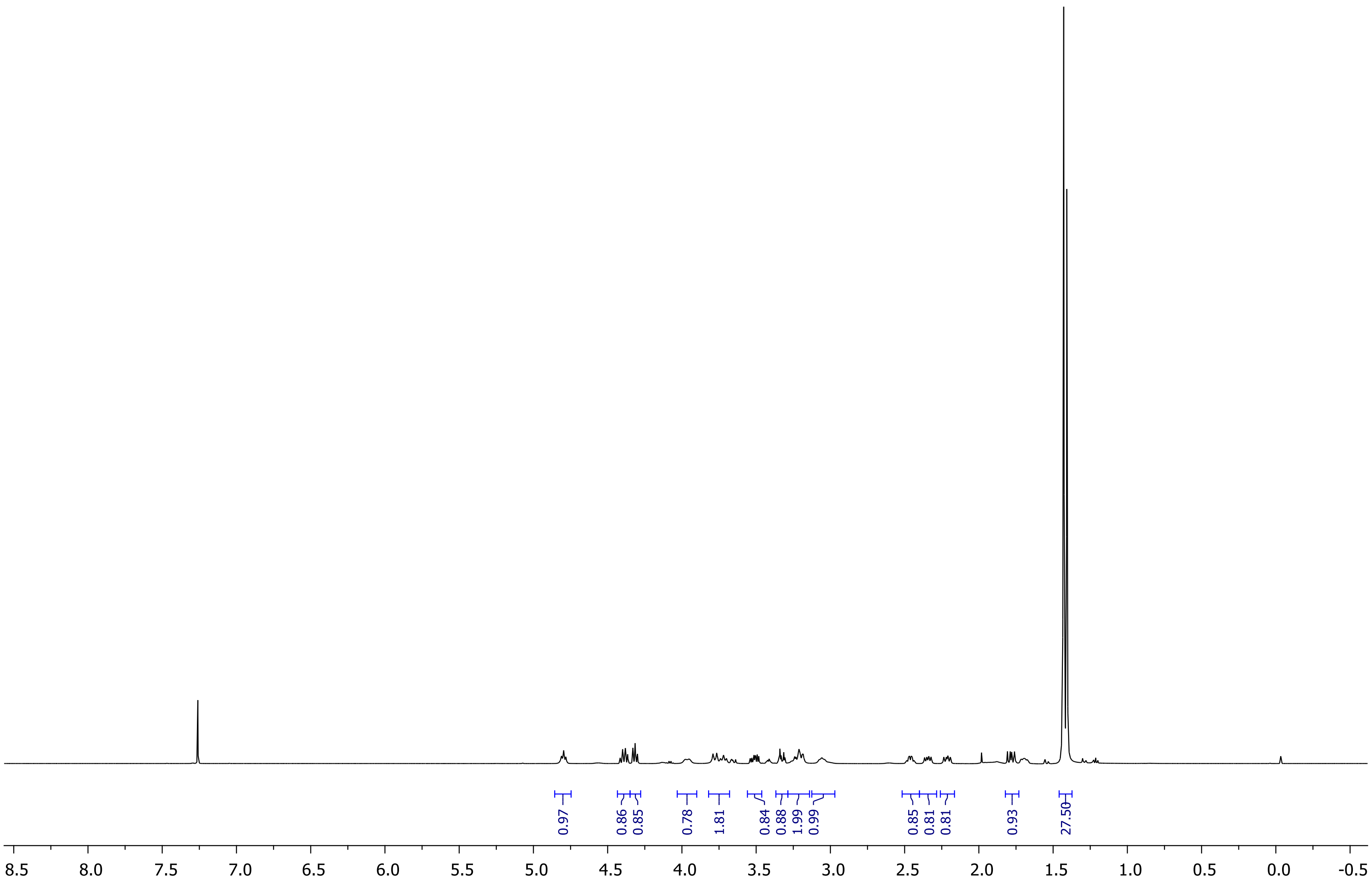
0.97
0.86
0.85
0.78
1.81
0.84
0.88
1.99
0.99
0.85
0.81
0.81
0.93
27.50
8.5 8.0 7.5 7.0 6.5 6.0 5.5 5.0 4.5 4.0 3.5 3.0 2.5 2.0 1.5 1.0 0.5 0.0 -0.5

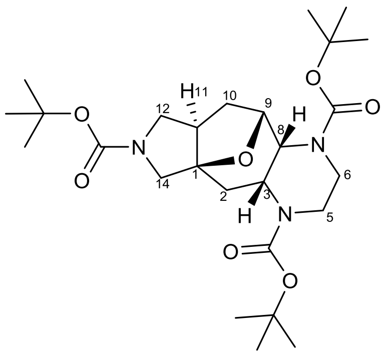

S20

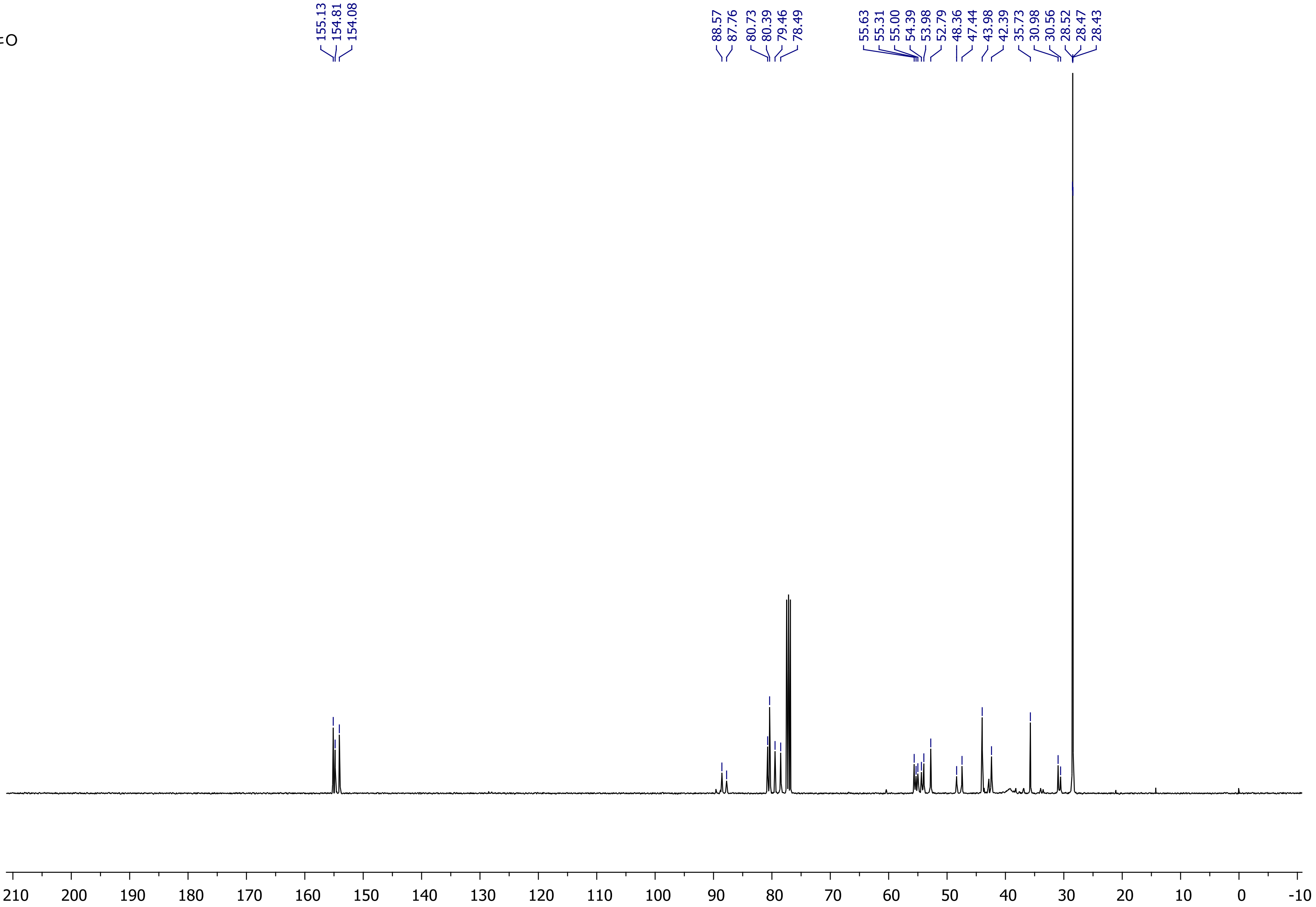
155.13
154.81
154.08
88.57
87.76
80.73
80.39
79.46
78.49
55.63
55.31
55.00
54.39
53.98
52.79
48.36
47.44
43.98
42.39
35.73
30.98
30.56
28.52
28.47
28.43
210
200
190
180
170
160
150
140
130
120
110
100
90
80
70
60
50
40
30
20
10
0
-10

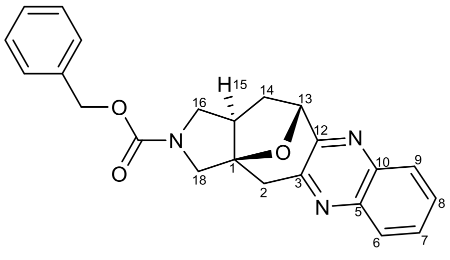
H
15
14
13
16
O
N
12
N
10
9
O
1
O
18
2
3
8
N
5
6
7

S21

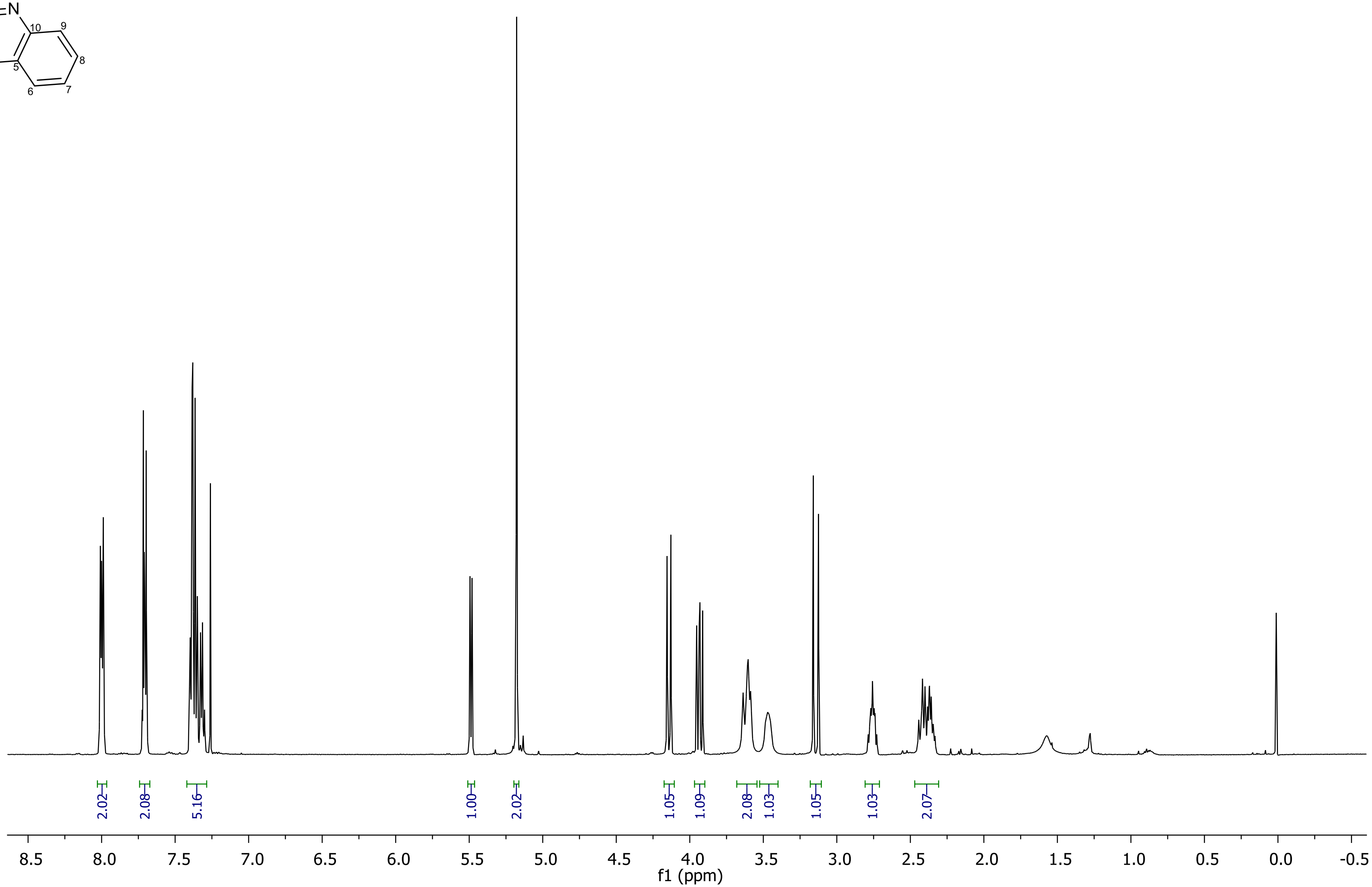
2.02
2.08
5.16
1.00
2.02
1.05
1.09
2.08
1.03
1.05
1.03
2.07
8.5
8.0
7.5
7.0
6.5
6.0
5.5
5.0
4.5
4.0
3.5
3.0
2.5
2.0
1.5
1.0
0.5
0.0
-0.5
f1 (ppm)

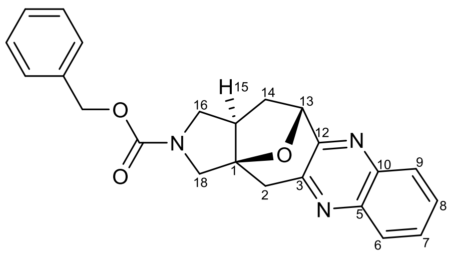
H
O
O
N
O
N
N
1
2
3
5
6
7
8
9
10
12
13
14
15
16
18

S21

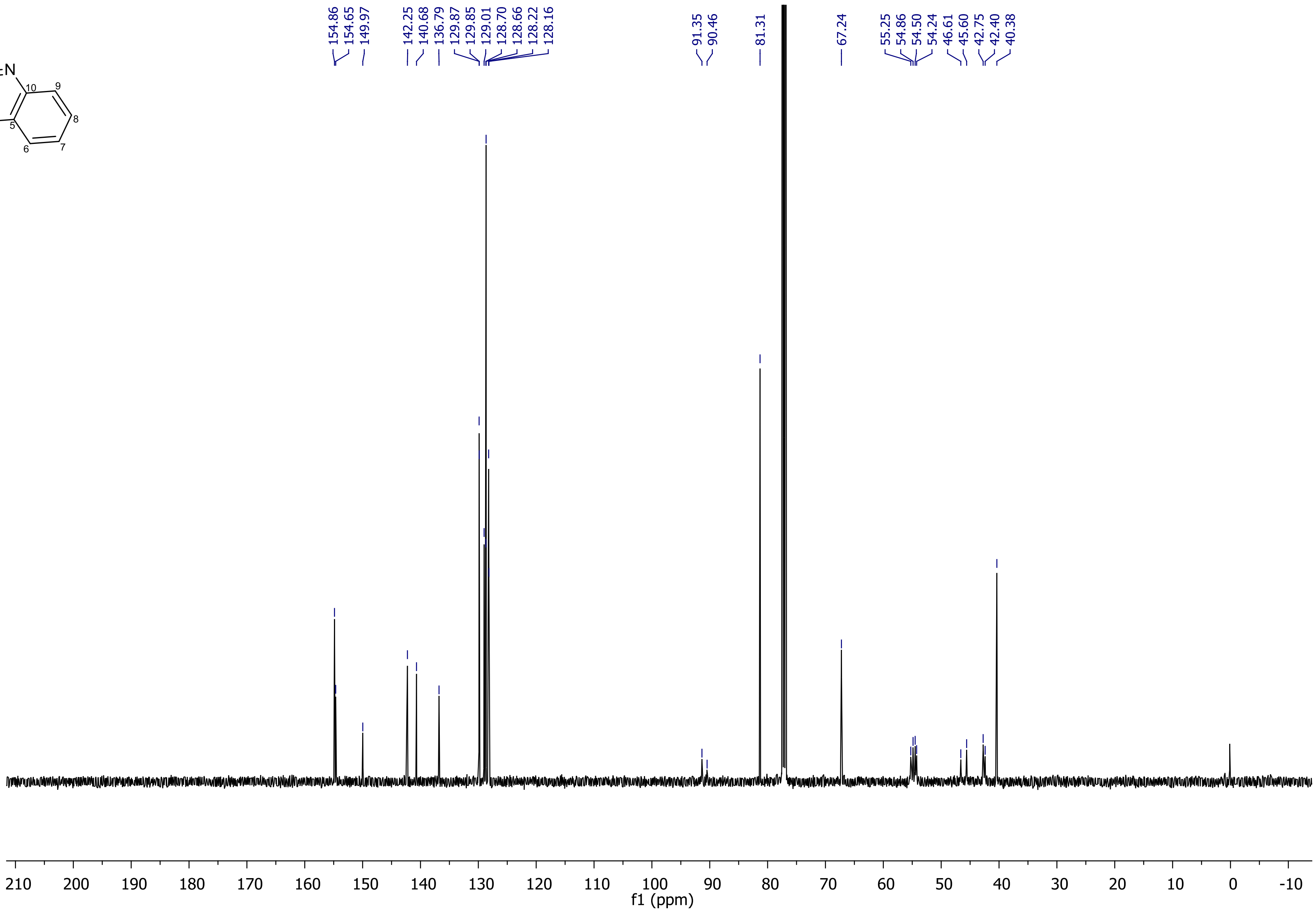
154.86
154.65
149.97
142.25
140.68
136.79
129.87
129.85
129.01
128.70
128.66
128.22
128.16
91.35
90.46
81.31
67.24
55.25
54.86
54.50
54.24
46.61
45.60
42.75
42.40
40.38
210 200 190 180 170 160 150 140 130 120 110 100 90 80 70 60 50 40 30 20 10 0 -10
f1 (ppm)

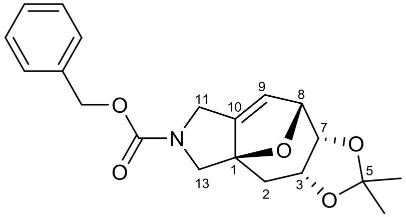

S22

(DMSO-d_6 @ 343 K)

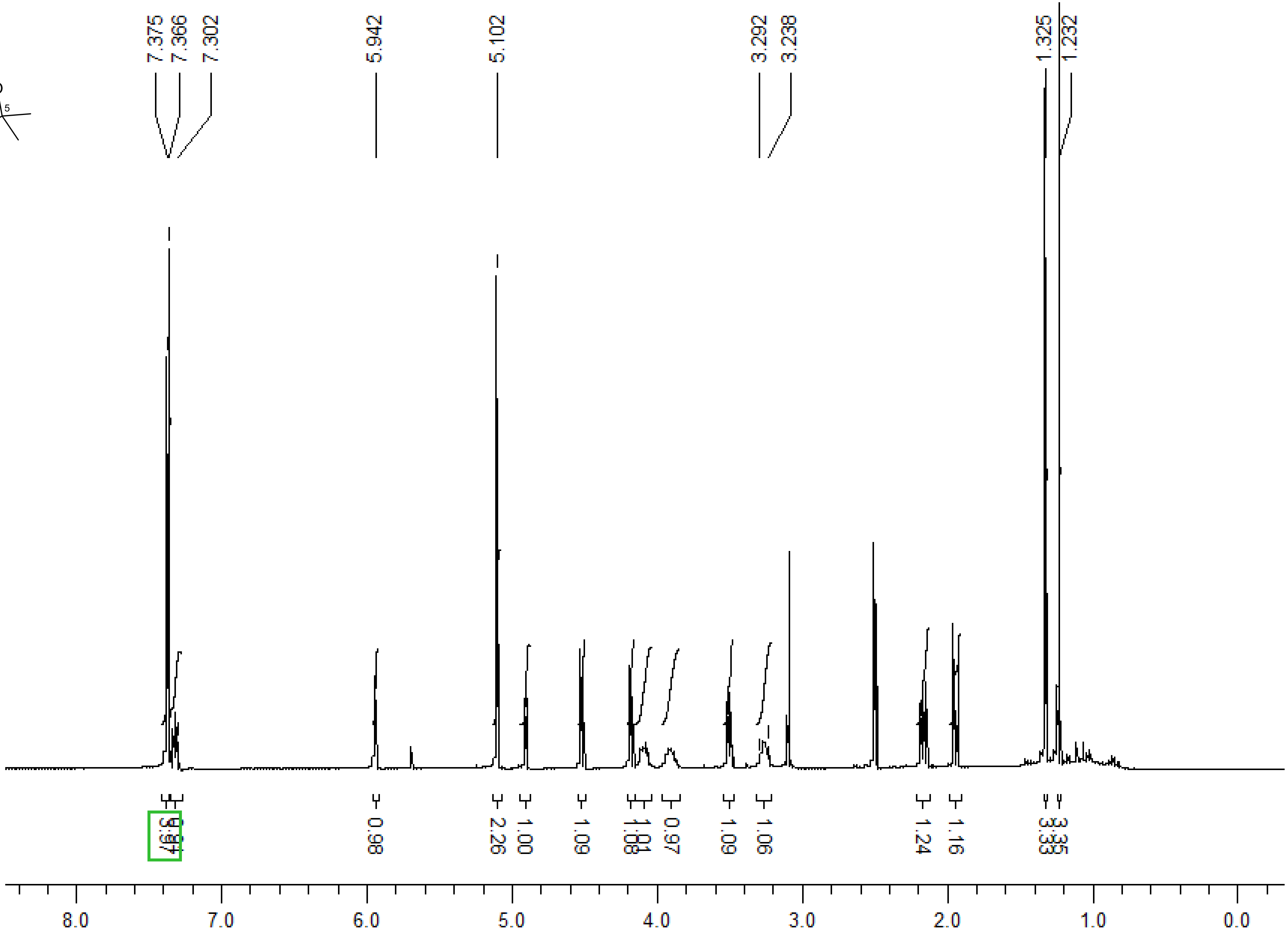

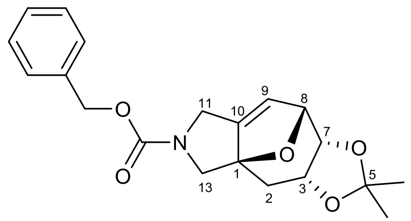

S22
(DMSO-d_6 @ 343 K)

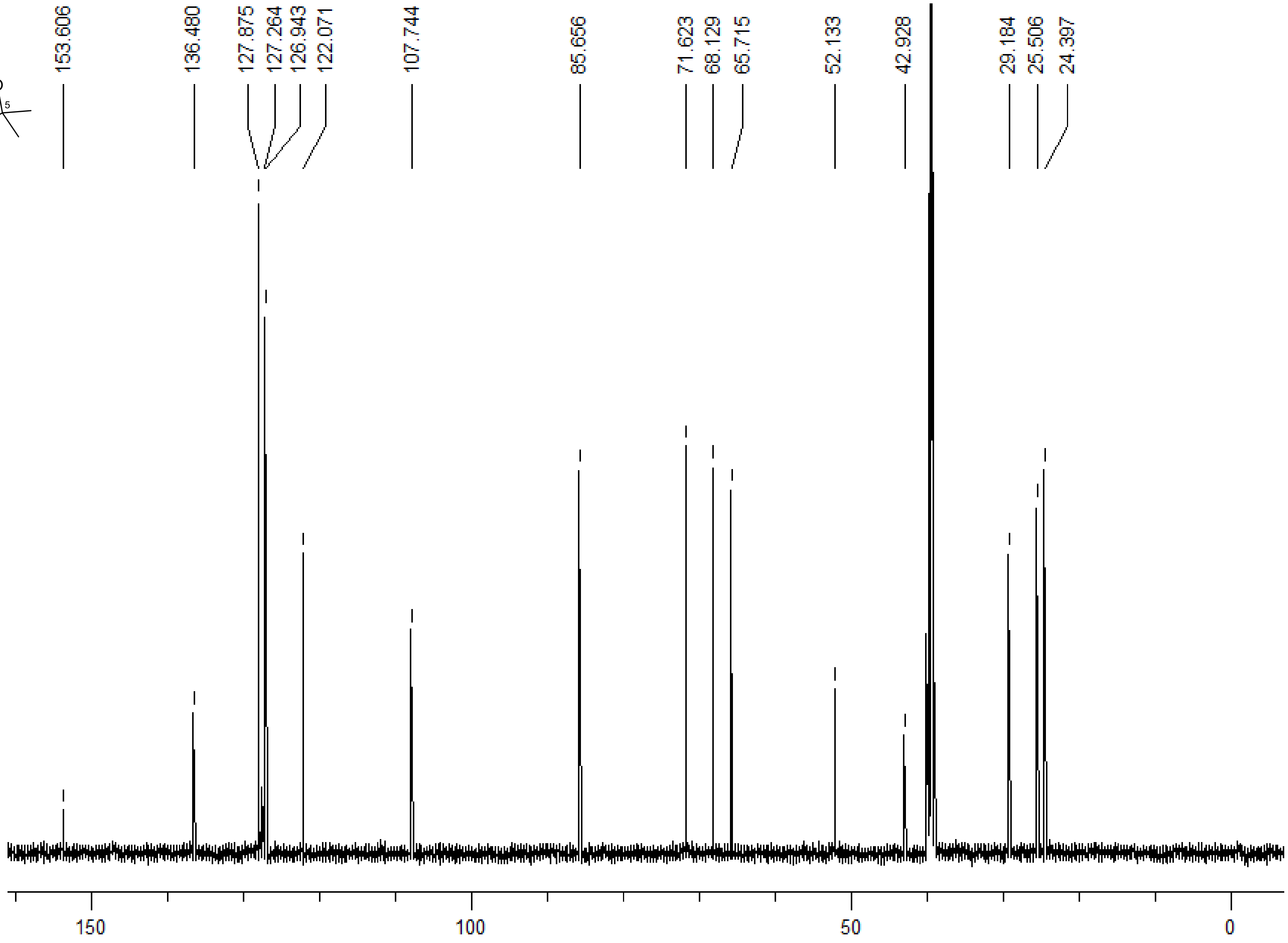

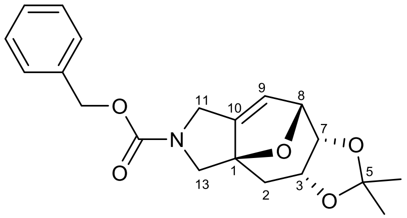

S22

($CDCl_3$ @ 300 K)

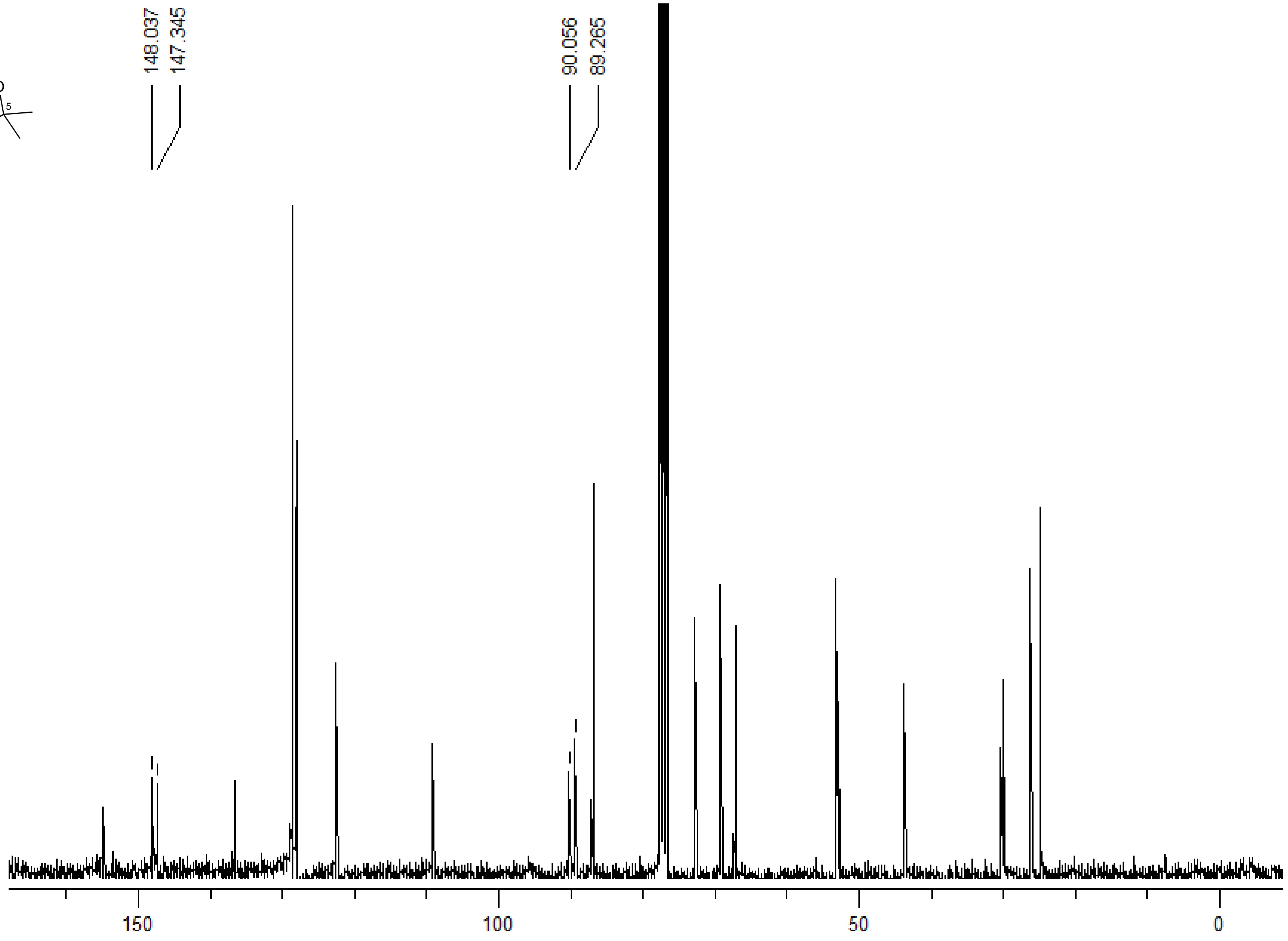

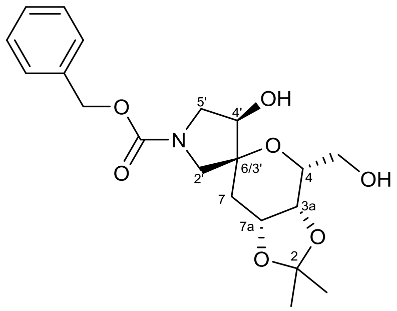

3

(CD_3OD @ 333 K)

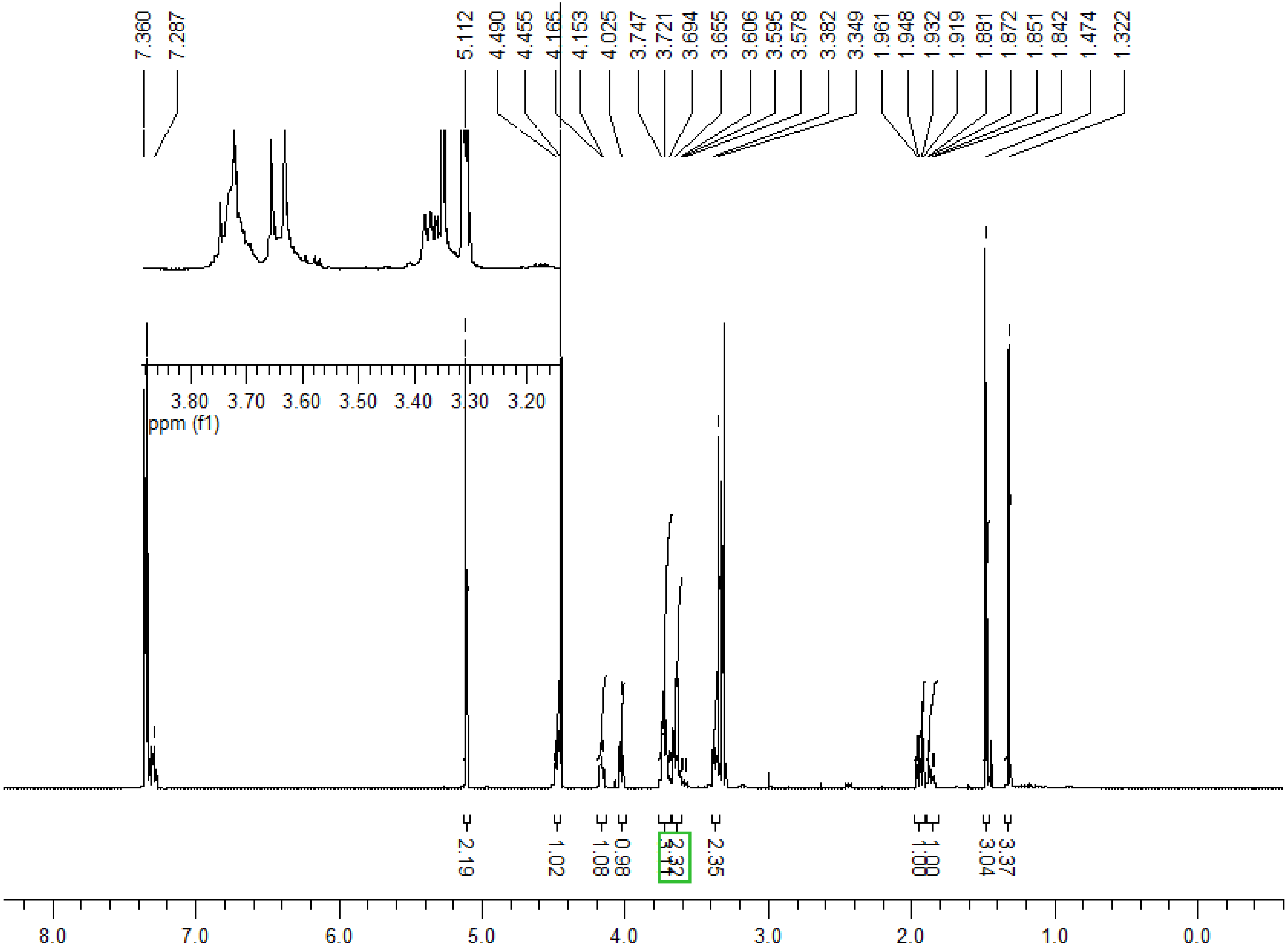

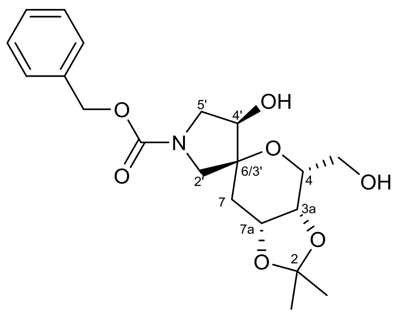
OH
OH
5'
4'
4
6/3'
2'
7
3a
7a
2
O
N

3
(CD_3OD @ 333 K)

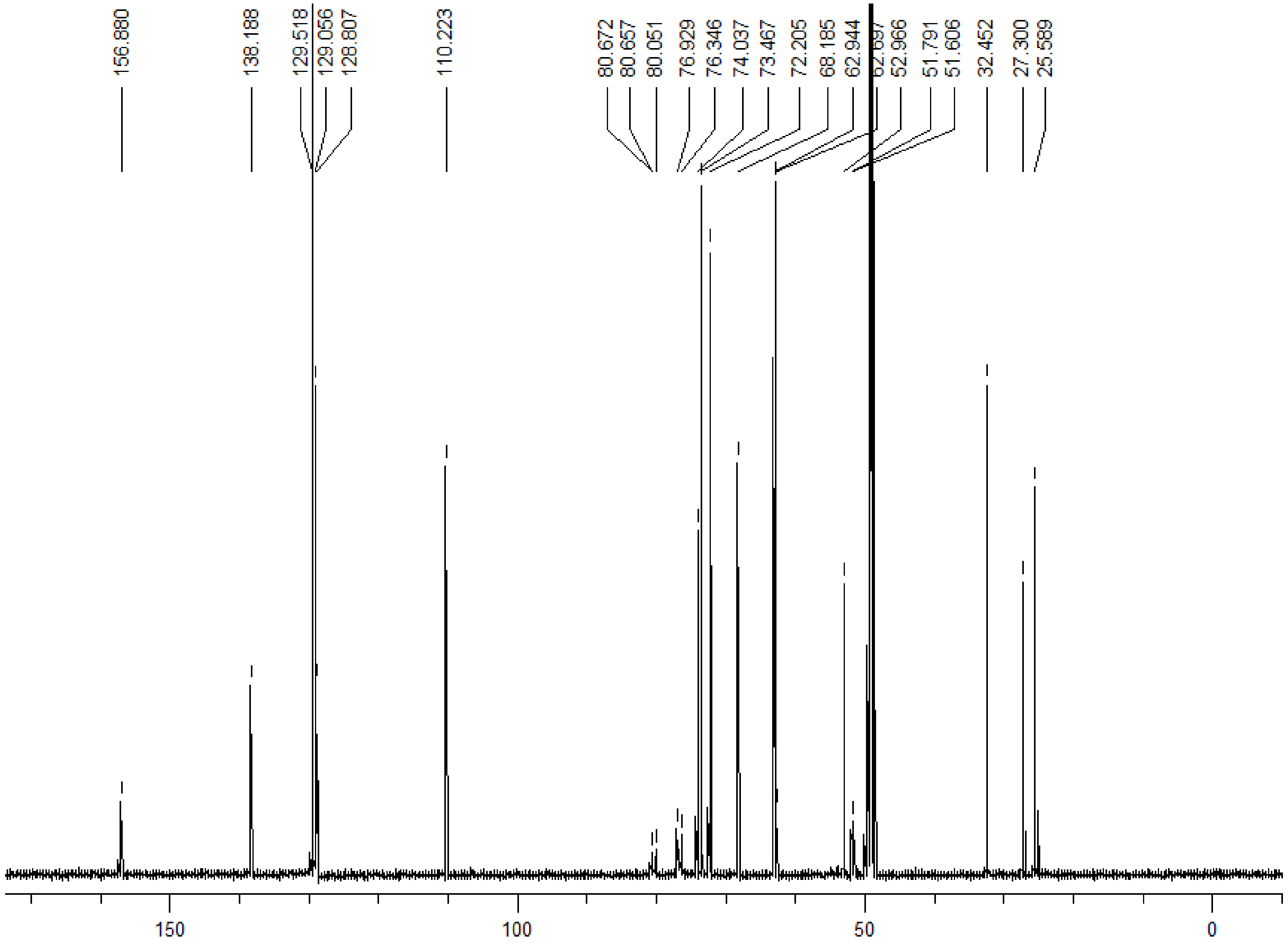
156.880
138.188
129.518
129.056
128.807
110.223
80.672
80.657
80.051
76.929
76.346
74.037
73.467
72.205
68.185
62.944
52.966
51.791
51.606
32.452
27.300
25.589
150
100
50
0

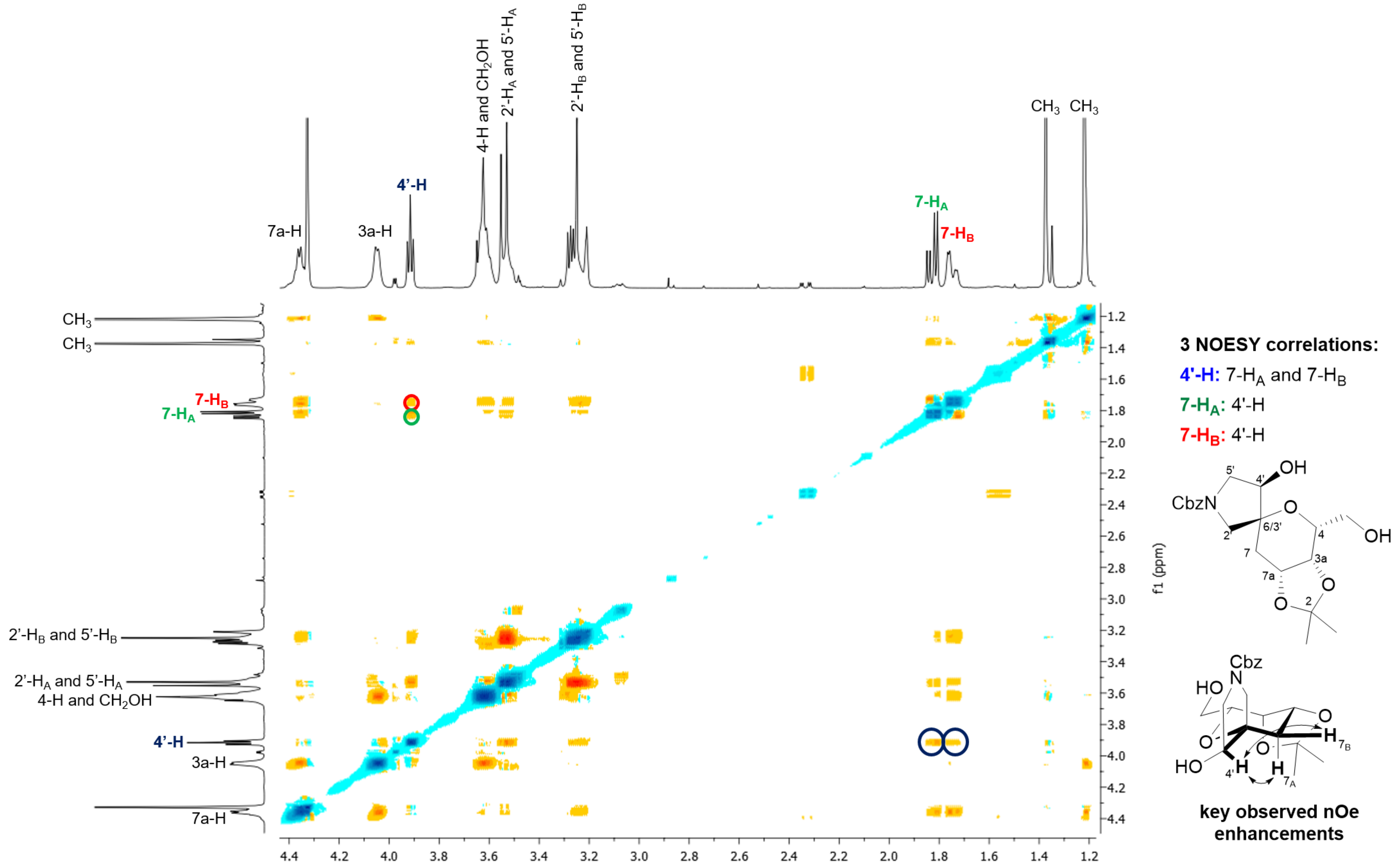

7a-H
3a-H
4'-H
4-H and CH2OH
2'-HA and 5'-HA
2'-HB and 5'-HB
7-HA
7-HB
CH3
CH3
f1 (ppm)
3 NOESY correlations:
4'-H: 7-HA and 7-HB
7-HA: 4'-H
7-HB: 4'-H
CbzN
OH
Cbz
HO
key observed nOe enhancements

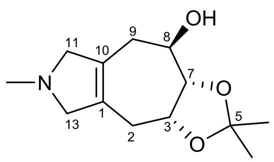

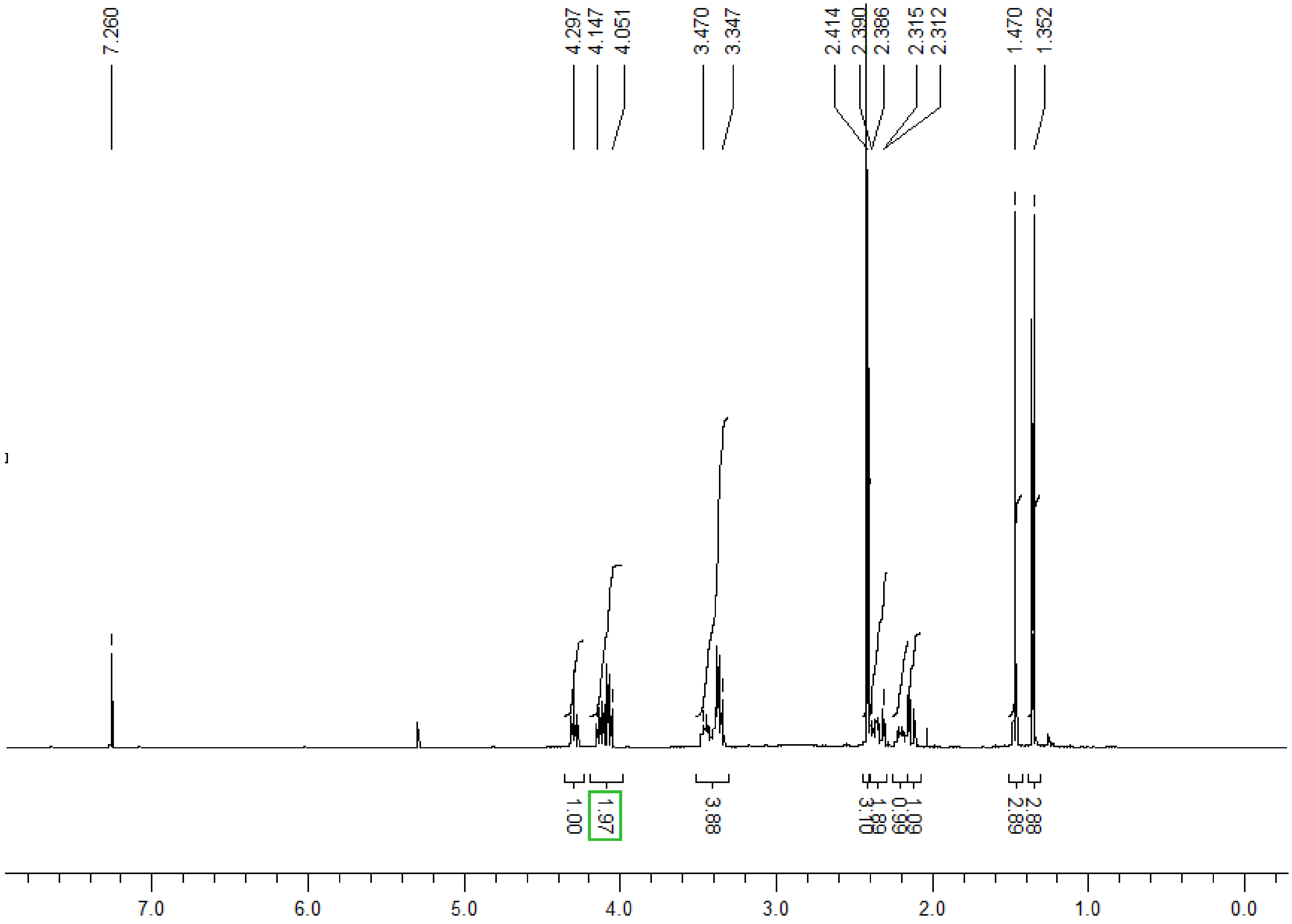
7.260
4.297
4.147
4.051
3.470
3.347
2.414
2.390
2.386
2.315
2.312
1.470
1.352
1.00
1.97
3.88
2.89
2.88
7.0
6.0
5.0
4.0
3.0
2.0
1.0
0.0

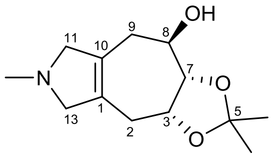

12 ≡ F12

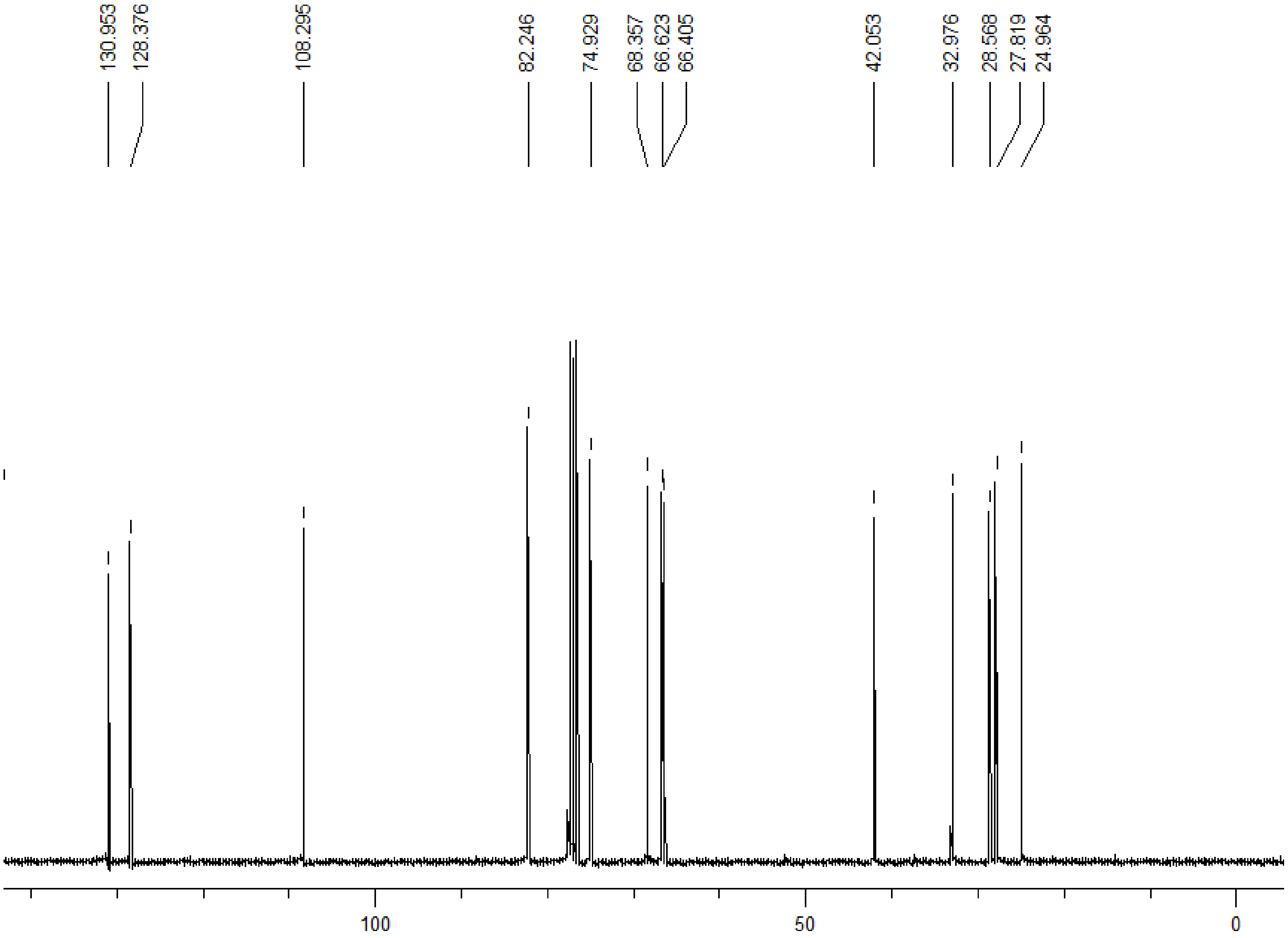

130.953
128.376
108.295
82.246
74.929
68.357
66.623
66.405
42.053
32.976
28.568
27.819
24.964
100
50
0

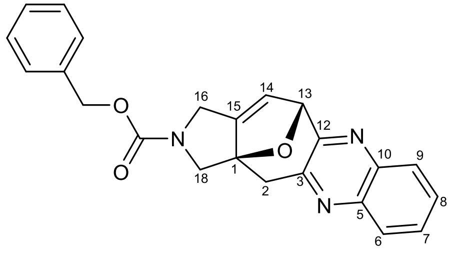

16
14
13
15
12
N
10
9
O
N
1
18
2
3
8
5
6
7
O
O
N

10

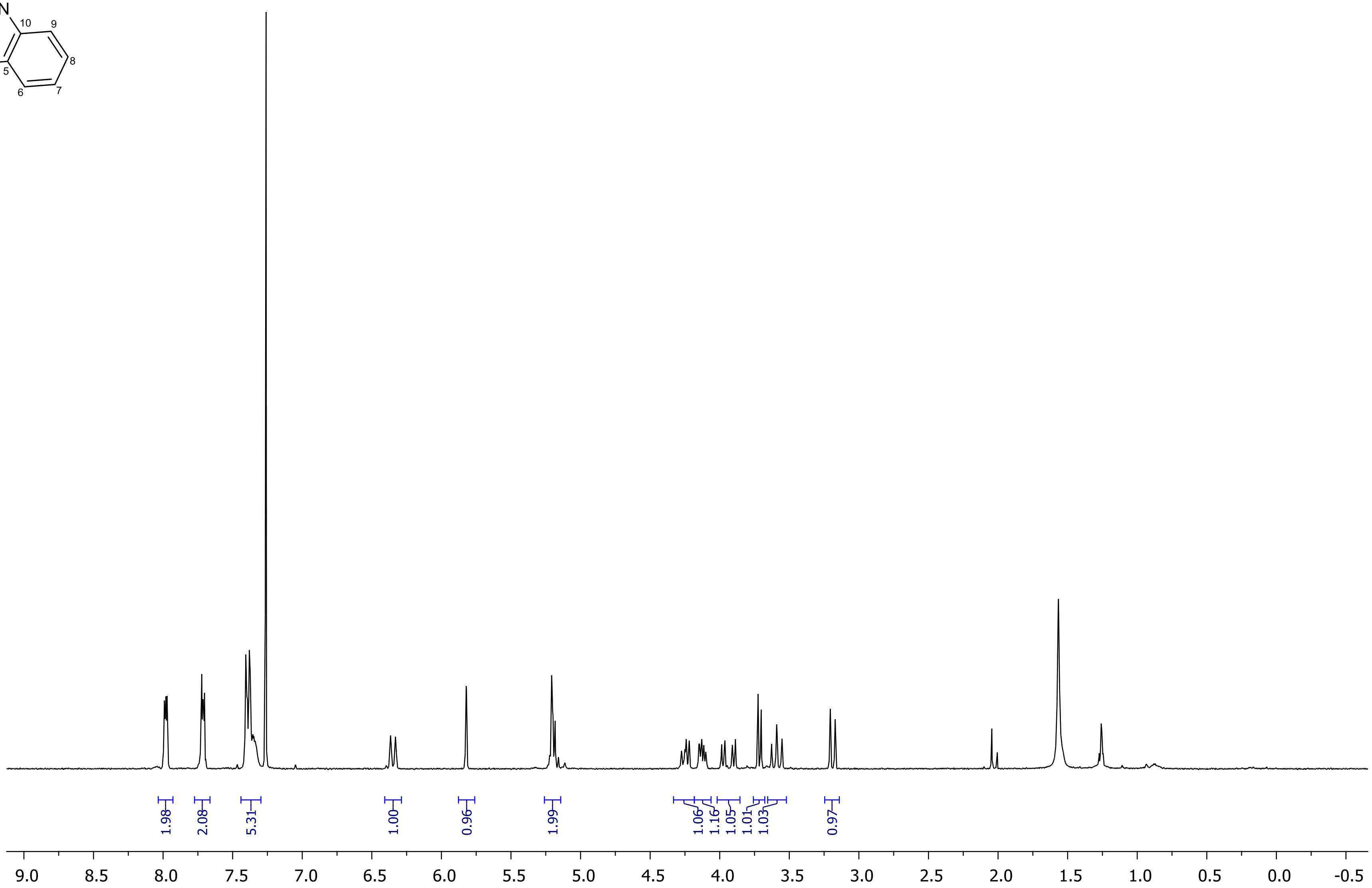

1.98
2.08
5.31
1.00
0.96
1.99
1.06
1.16
1.05
1.01
1.03
0.97
9.0
8.5
8.0
7.5
7.0
6.5
6.0
5.5
5.0
4.5
4.0
3.5
3.0
2.5
2.0
1.5
1.0
0.5
0.0
-0.5

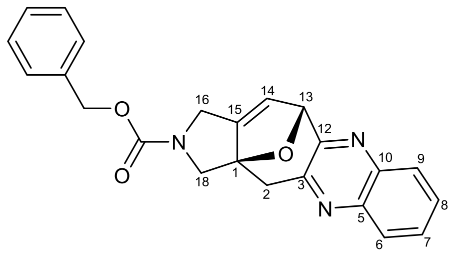

10

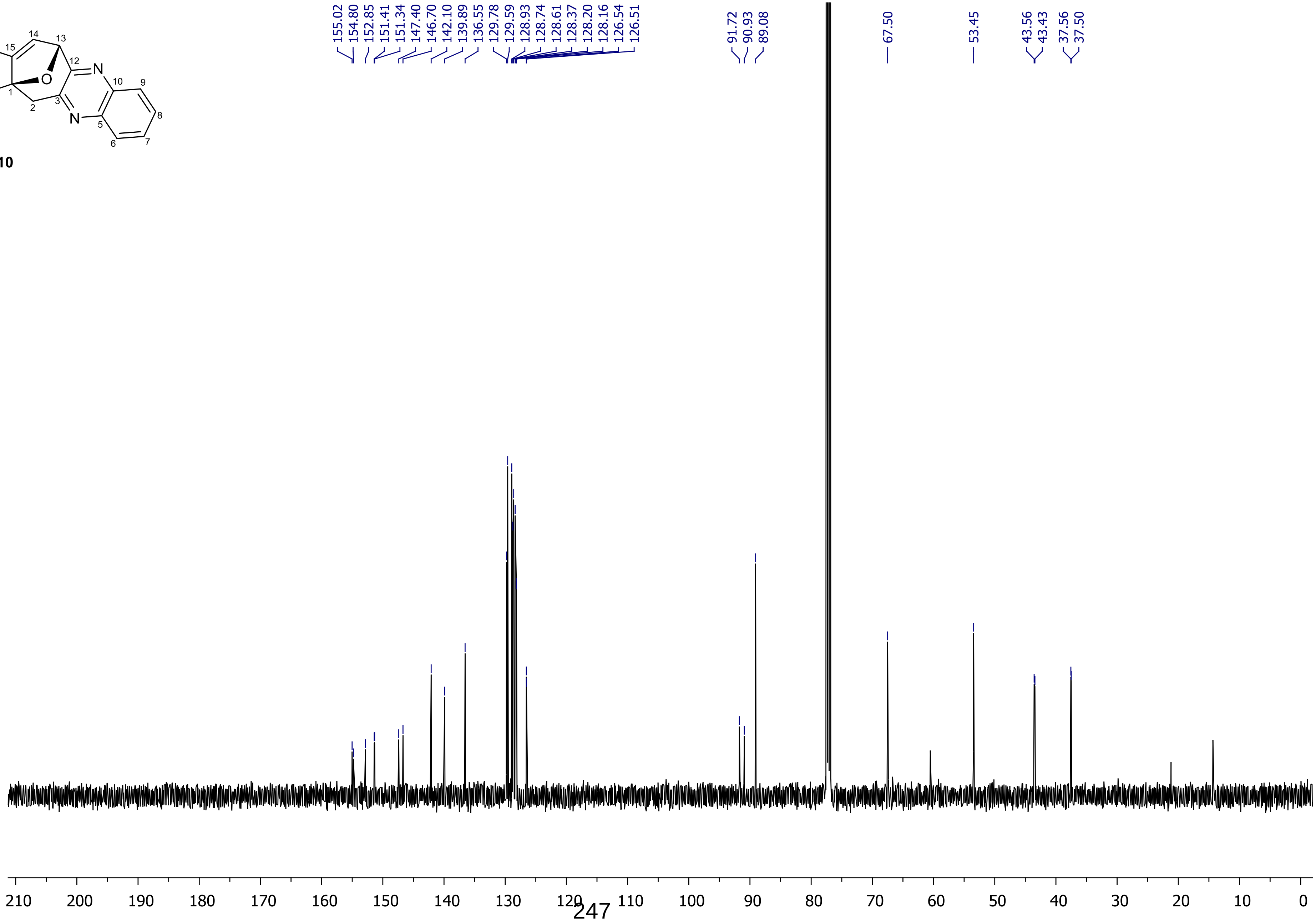

155.02
154.80
152.85
151.41
151.34
147.40
146.70
142.10
139.89
136.55
129.78
129.59
128.93
128.74
128.61
128.37
128.20
128.16
126.54
126.51
91.72
90.93
89.08
67.50
53.45
43.56
43.43
37.56
37.50
210 200 190 180 170 160 150 140 130 120 110 100 90 80 70 60 50 40 30 20 10 0

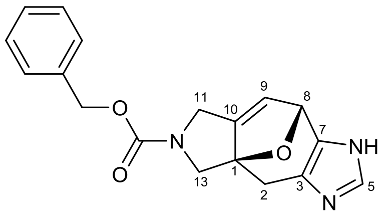

11

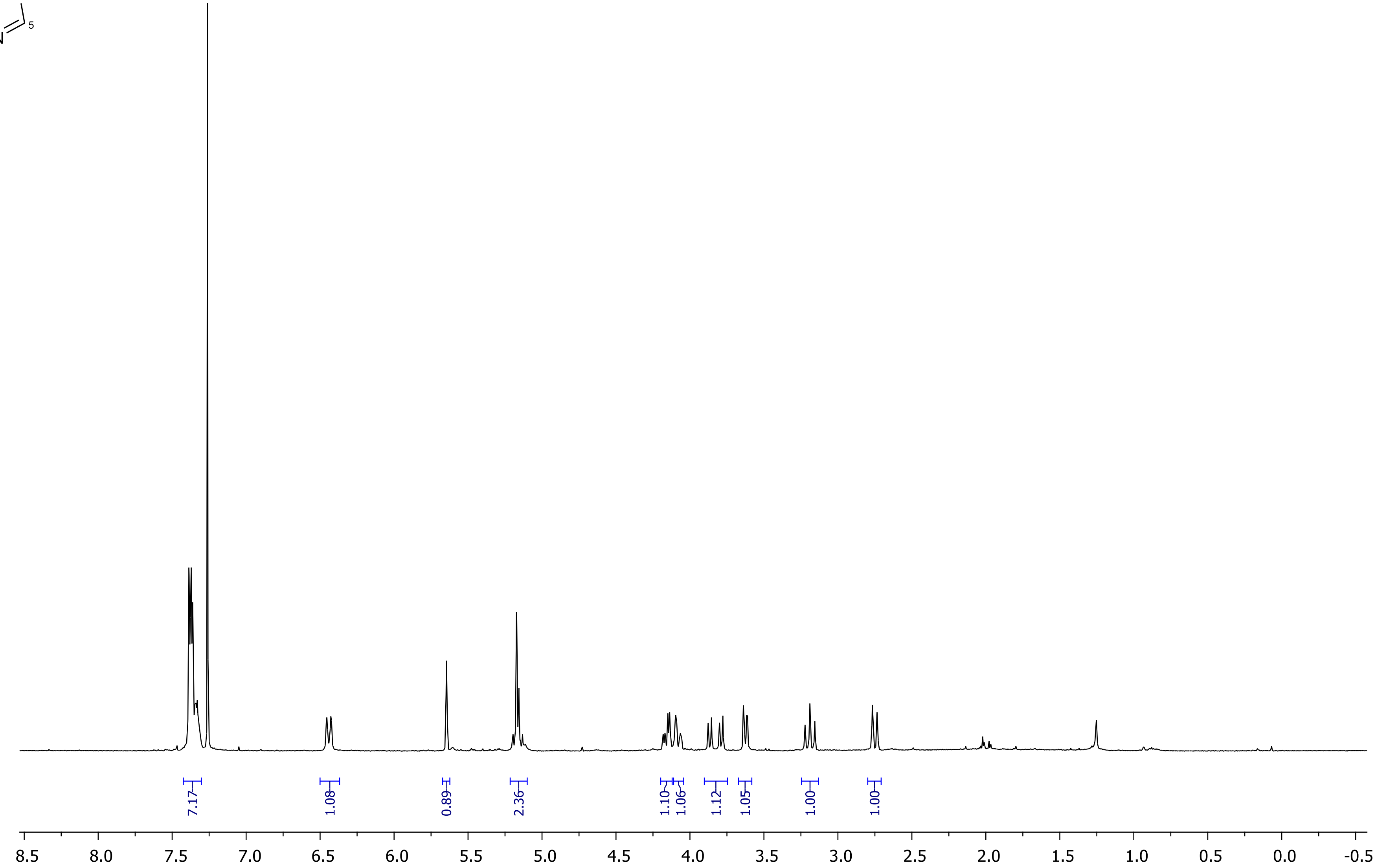

7.17
1.08
0.89
2.36
1.10
1.06
1.12
1.05
1.00
1.00
8.5 8.0 7.5 7.0 6.5 6.0 5.5 5.0 4.5 4.0 3.5 3.0 2.5 2.0 1.5 1.0 0.5 0.0 -0.5

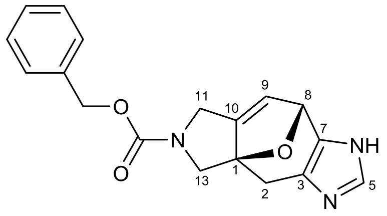

O
O
N
11
10
9
8
7
NH
O
13
1
2
3
N
5

11

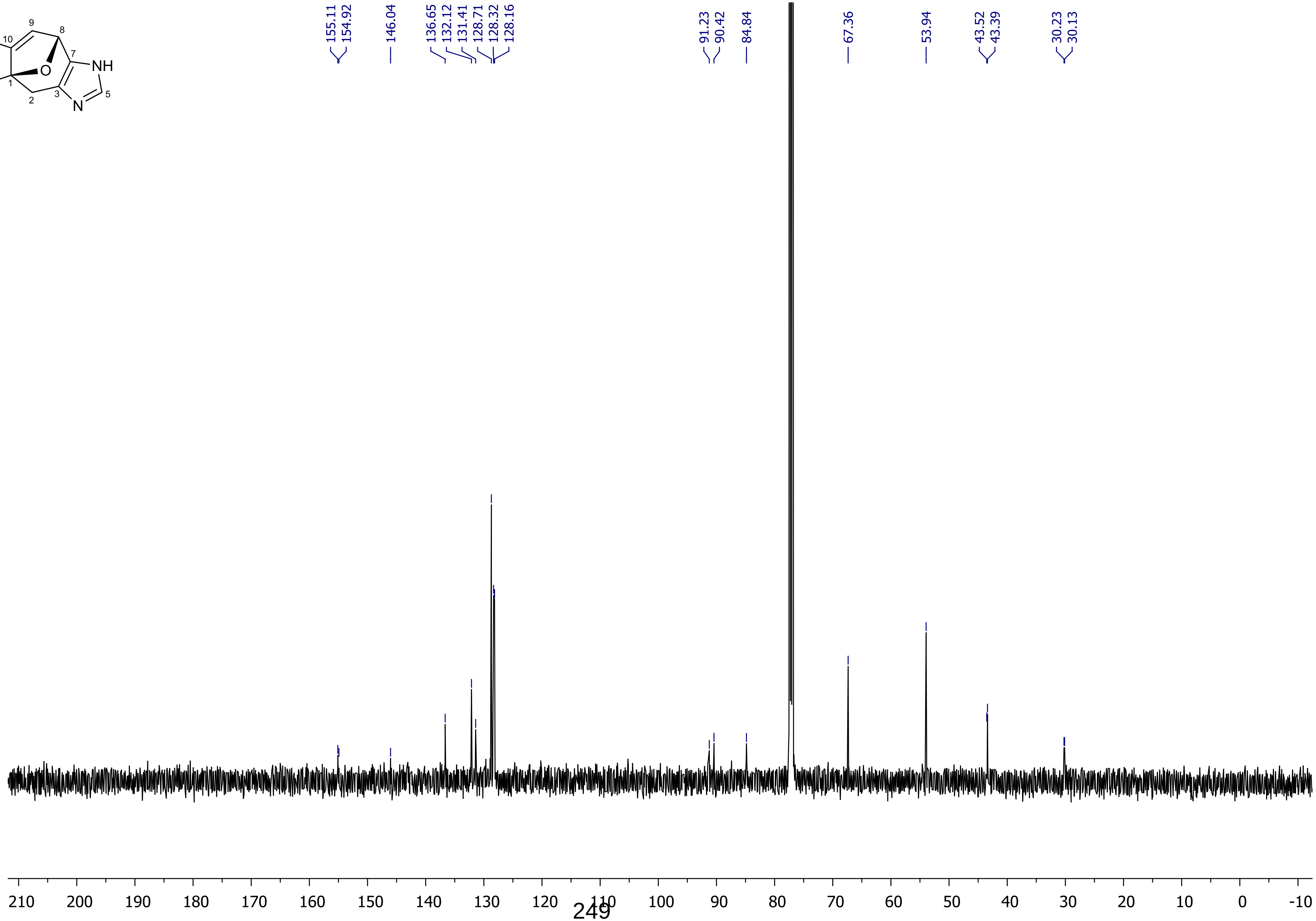

155.11
154.92
146.04
136.65
132.12
131.41
128.71
128.32
128.16
91.23
90.42
84.84
67.36
53.94
43.52
43.39
30.23
30.13
210 200 190 180 170 160 150 140 130 120 110 100 90 80 70 60 50 40 30 20 10 0 -10

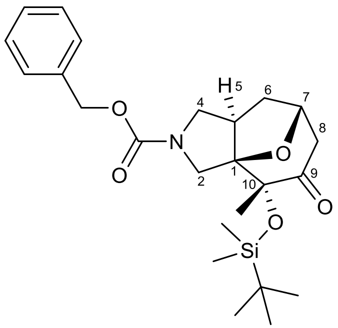

S23

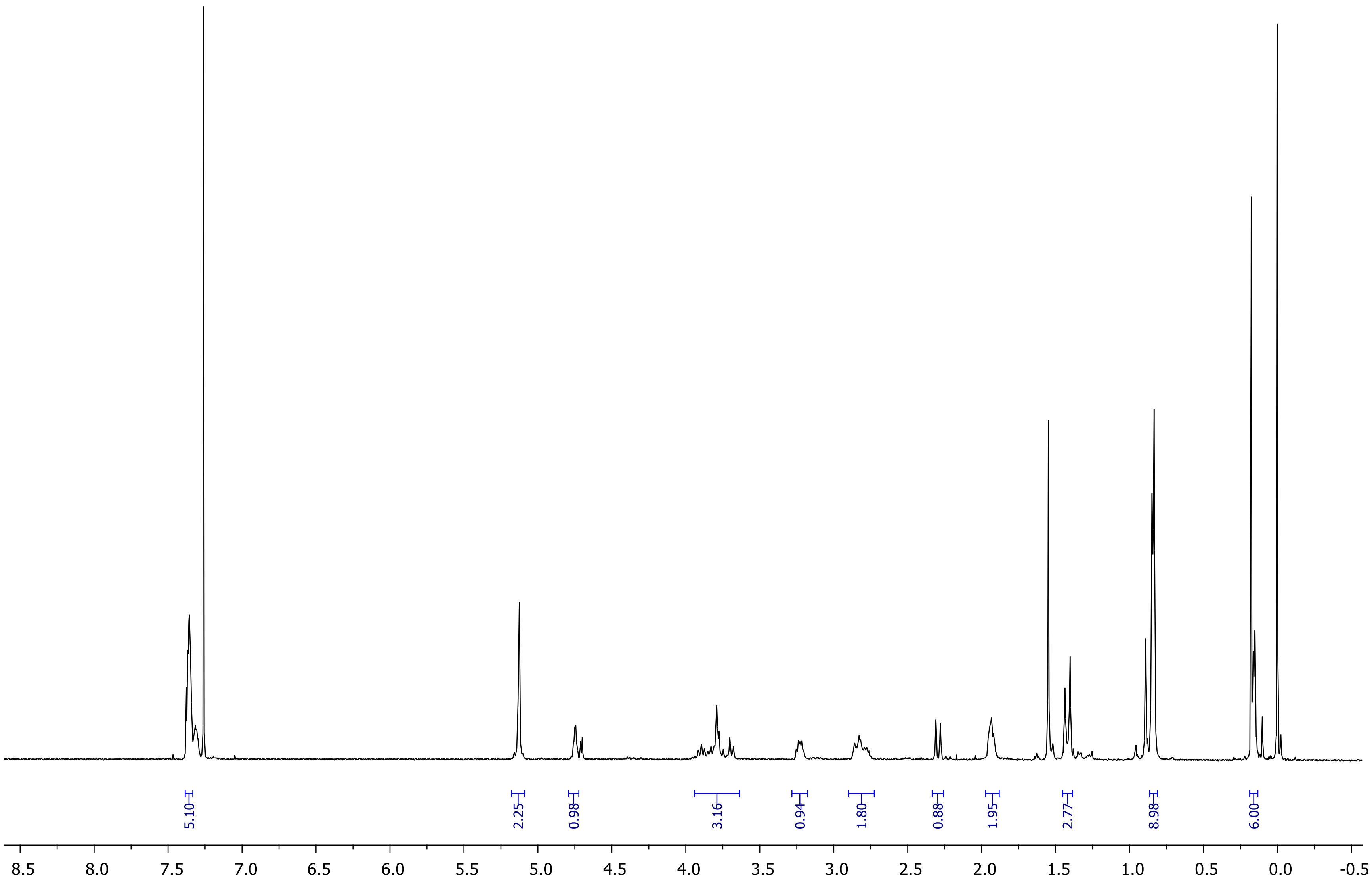
5.10
2.25
0.98
3.16
0.94
1.80
0.88
1.95
2.77
8.98
6.00
8.5
8.0
7.5
7.0
6.5
6.0
5.5
5.0
4.5
4.0
3.5
3.0
2.5
2.0
1.5
1.0
0.5
0.0
-0.5

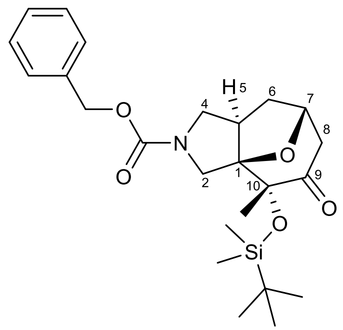
H
5
6
7
8
4
N
O
O
O
1
2
10
9
O
O
Si

S23

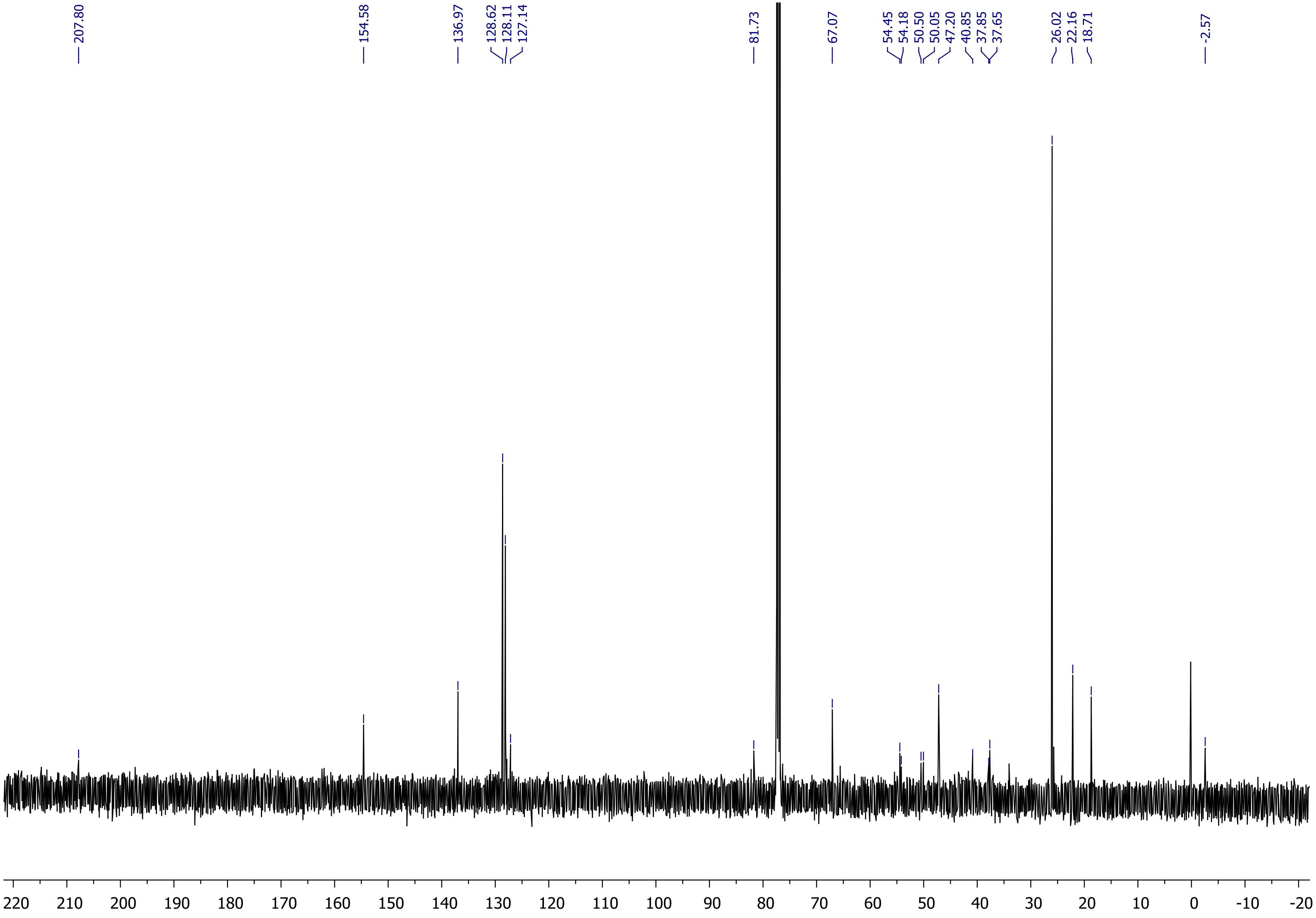
207.80
154.58
136.97
128.62
128.11
127.14
81.73
67.07
54.45
54.18
50.50
50.05
47.20
40.85
37.85
37.65
26.02
22.16
18.71
-2.57
220 210 200 190 180 170 160 150 140 130 120 110 100 90 80 70 60 50 40 30 20 10 0 -10 -20

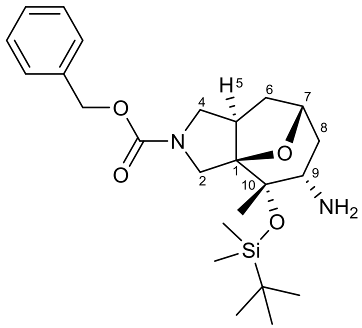

13

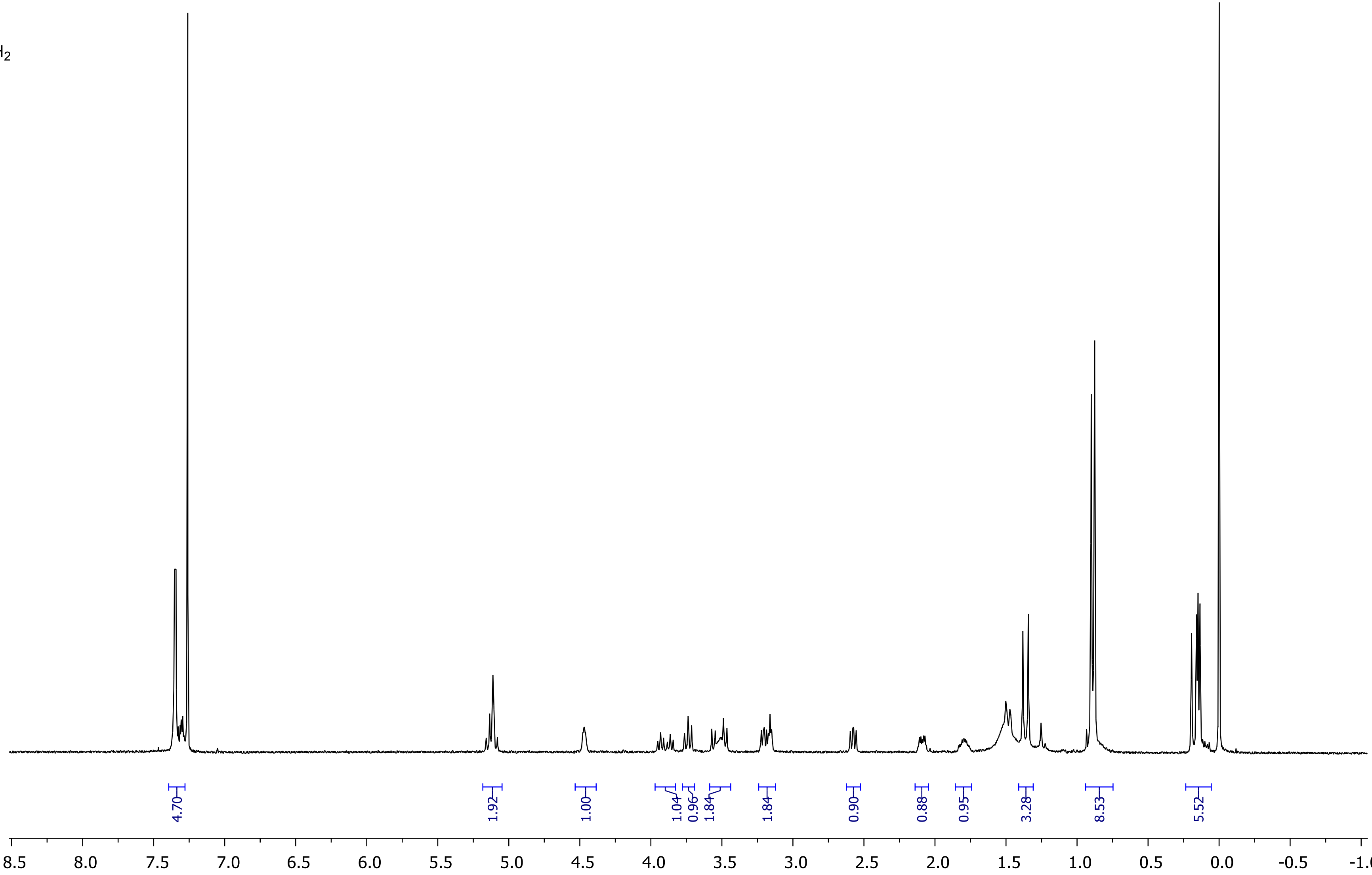

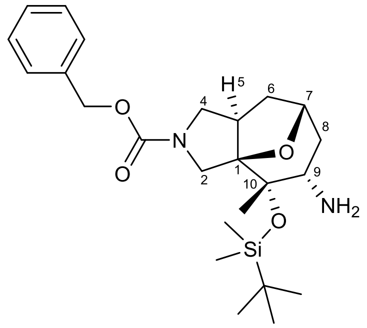
H 5
6
7
4
8
N
O
O
O
2
1
10
9
NH2
O
Si

13

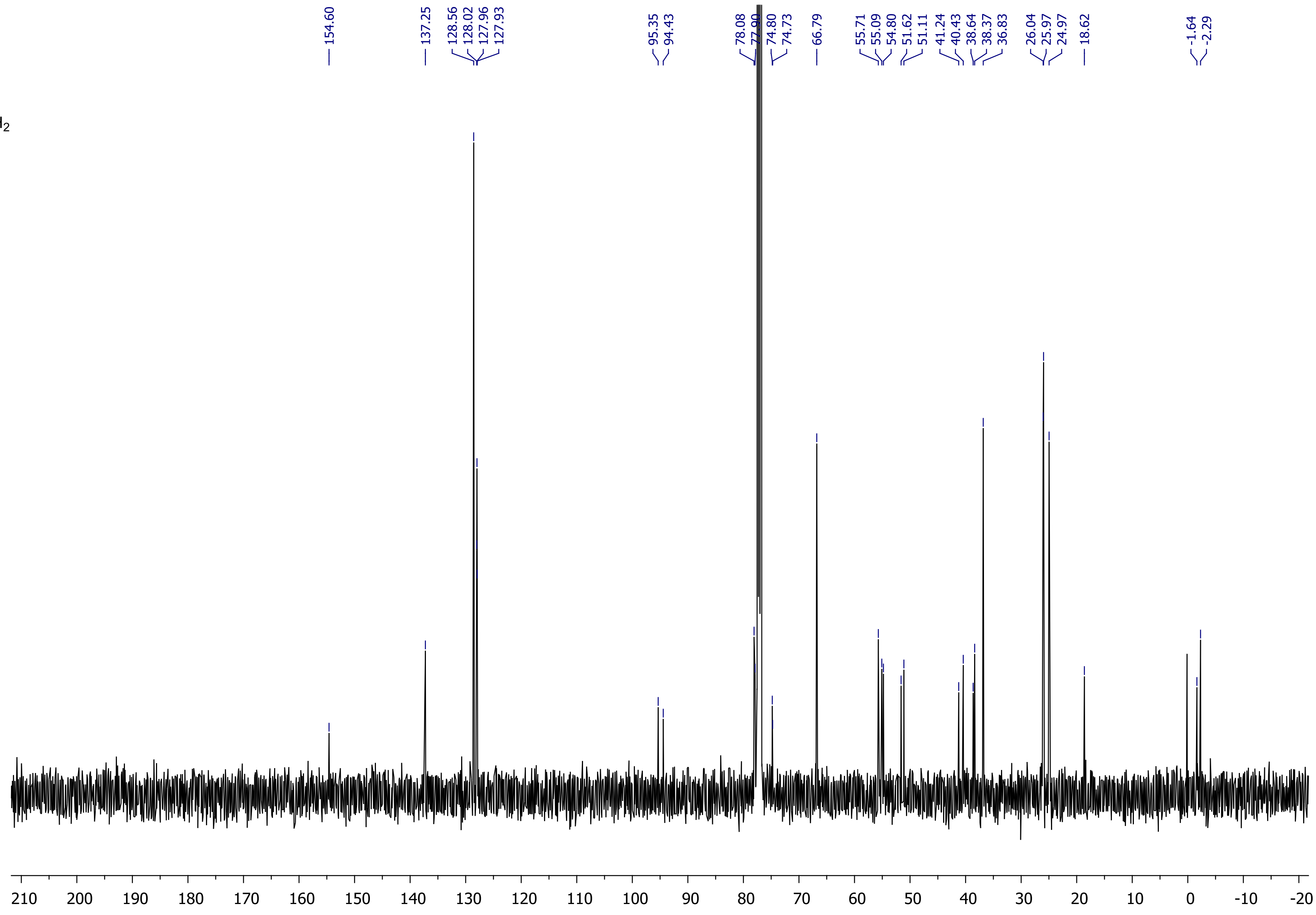
154.60
137.25
128.56
128.02
127.96
127.93
95.35
94.43
78.08
77.90
74.80
74.73
66.79
55.71
55.09
54.80
51.62
51.11
41.24
40.43
38.64
38.37
36.83
26.04
25.97
24.97
18.62
-1.64
-2.29
210
200
190
180
170
160
150
140
130
120
110
100
90
80
70
60
50
40
30
20
10
0
-10
-20

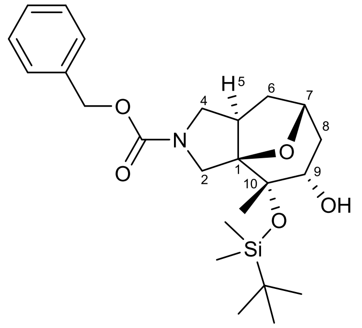

H5
4
6
7
8
N
O
O
O
1
2
10
9
OH
O
Si

S24

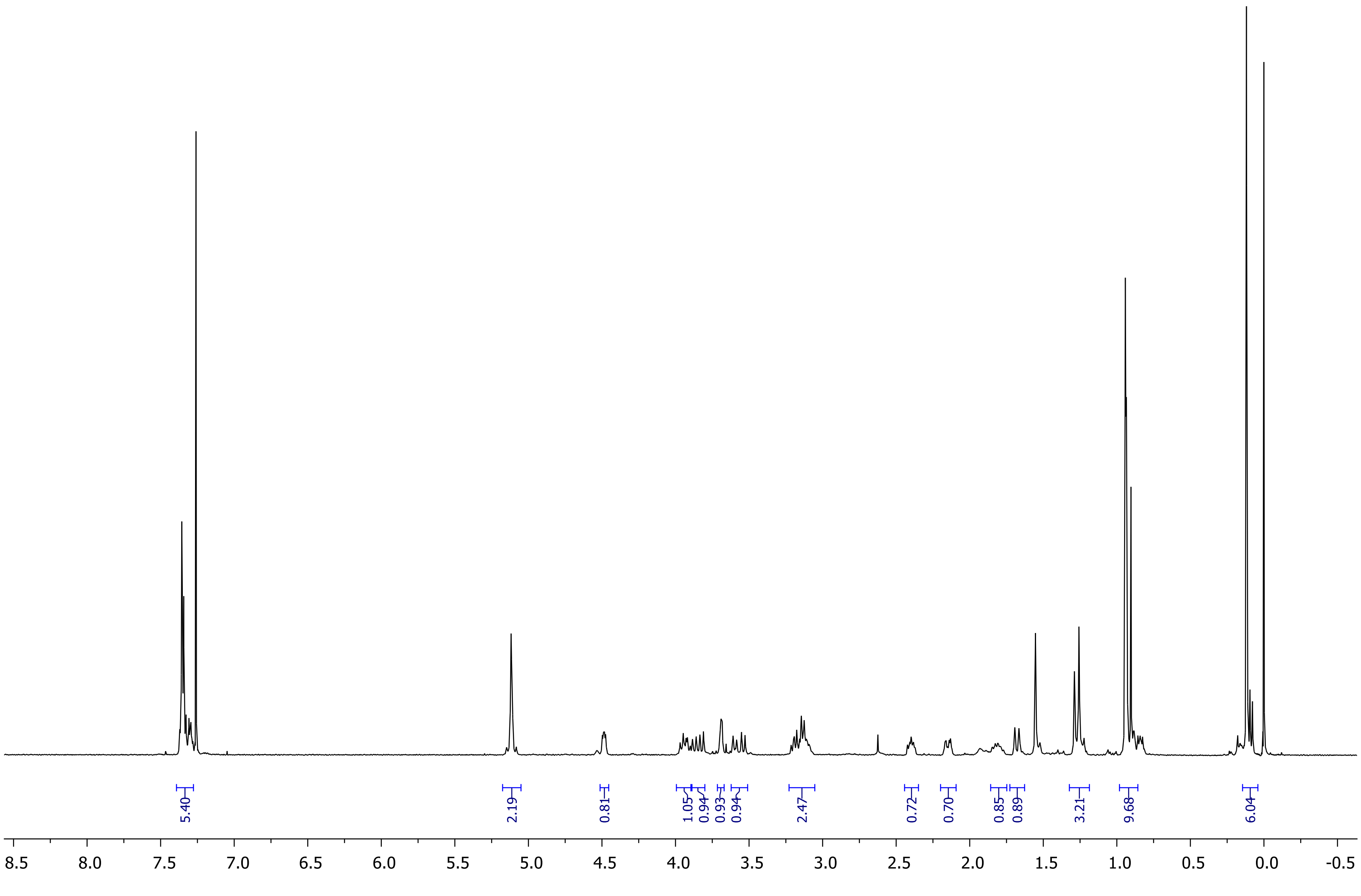

5.40
2.19
0.81
1.05
0.94
0.93
0.94
2.47
0.72
0.70
0.85
0.89
3.21
9.68
6.04
8.5 8.0 7.5 7.0 6.5 6.0 5.5 5.0 4.5 4.0 3.5 3.0 2.5 2.0 1.5 1.0 0.5 0.0 -0.5

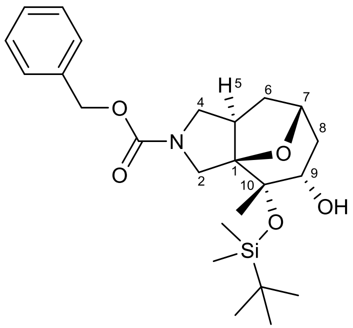

O
O
N
H5
4
6
7
8
O
1
2
10
9
OH
O
Si

S24

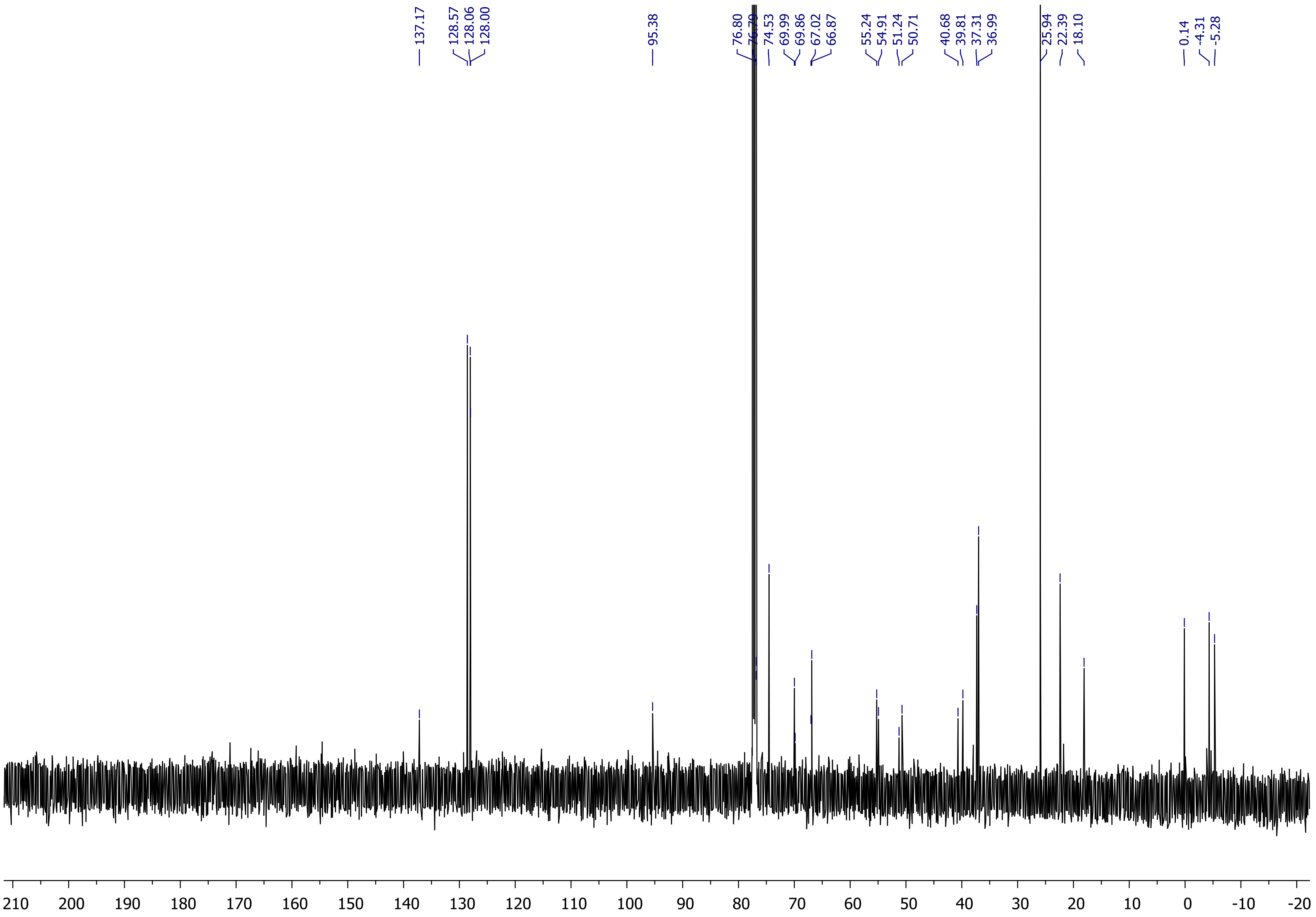

137.17
128.57
128.06
128.00
95.38
76.80
74.53
69.99
69.86
67.02
66.87
55.24
54.91
51.24
50.71
40.68
39.81
37.31
36.99
25.94
22.39
18.10
0.14
-4.31
-5.28
210 200 190 180 170 160 150 140 130 120 110 100 90 80 70 60 50 40 30 20 10 0 -10 -20

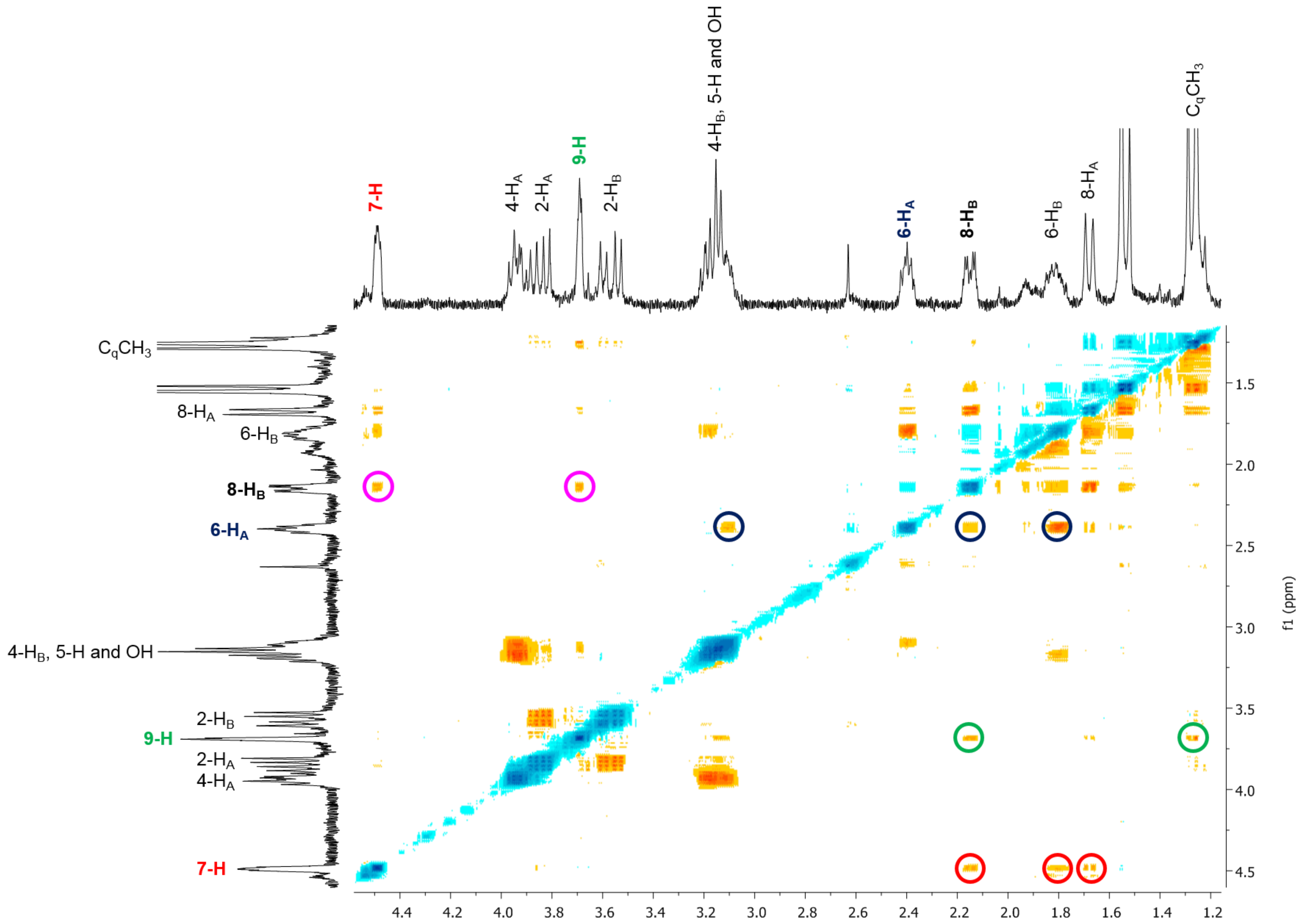

S24 NOESY correlations:

6-H$_A$: 5-H; 6-H$_B$; 8-H$_A$

7-H: 6-H$_B$; 8-H

8-H$_B$: 7-H; 9-H

9-H: 8-H$_B$; C$_q$CH$_3$

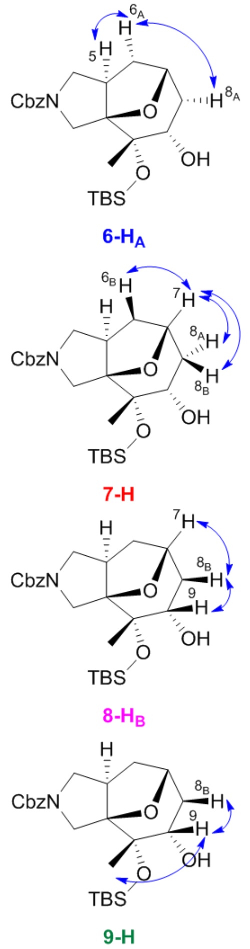

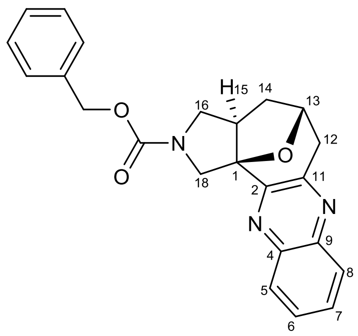

14

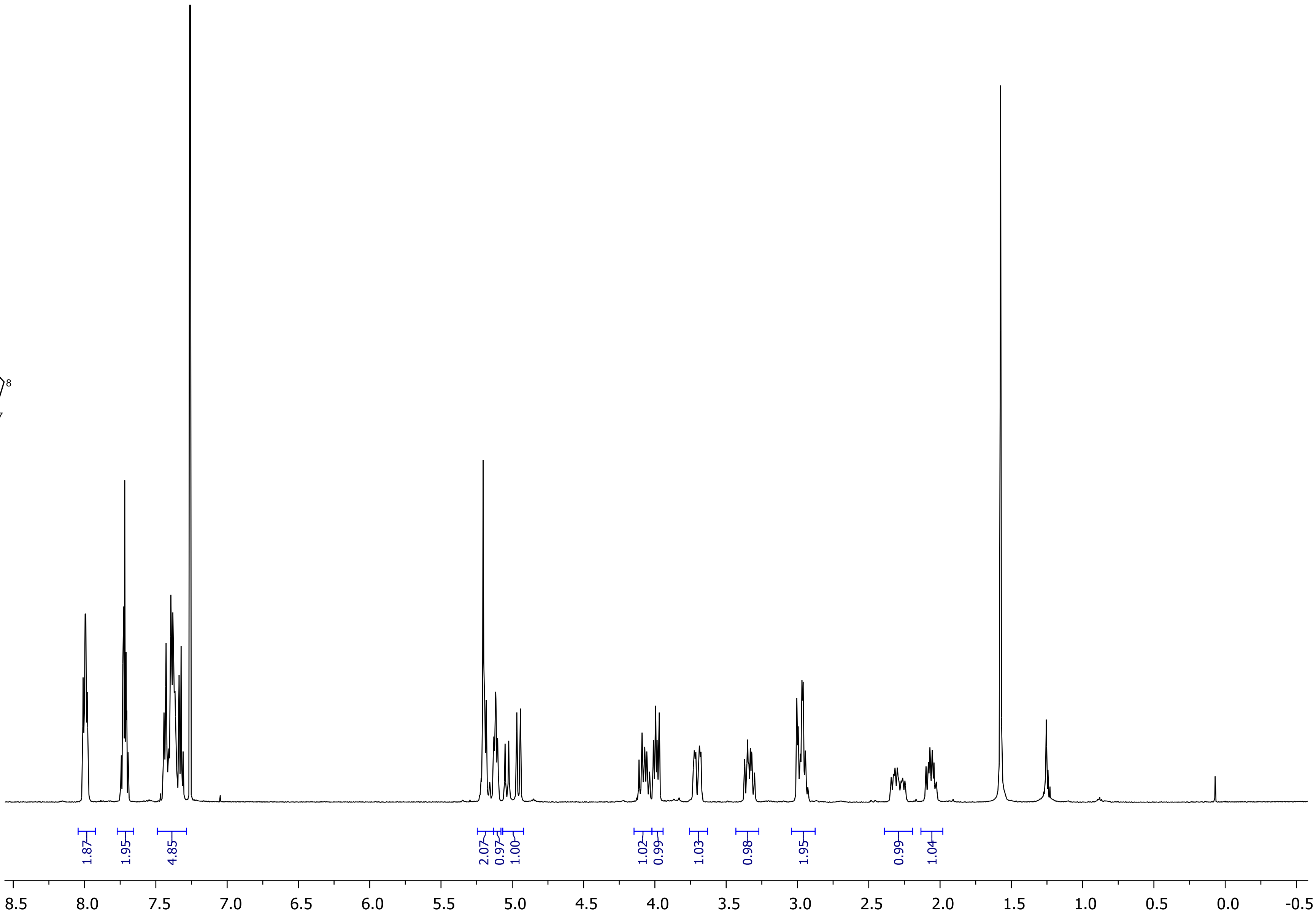
1.87
1.95
4.85
2.07
0.97
1.00
1.02
0.99
1.03
0.98
1.95
0.99
1.04
8.5 8.0 7.5 7.0 6.5 6.0 5.5 5.0 4.5 4.0 3.5 3.0 2.5 2.0 1.5 1.0 0.5 0.0 -0.5

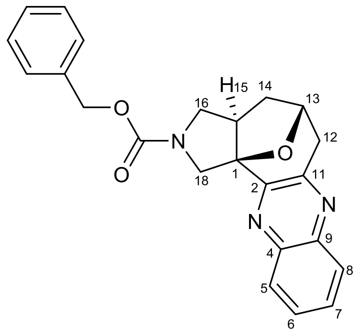
O
O
N
H
15
14
13
12
16
18
1
2
11
O
N
N
9
4
5
6
7
8

14

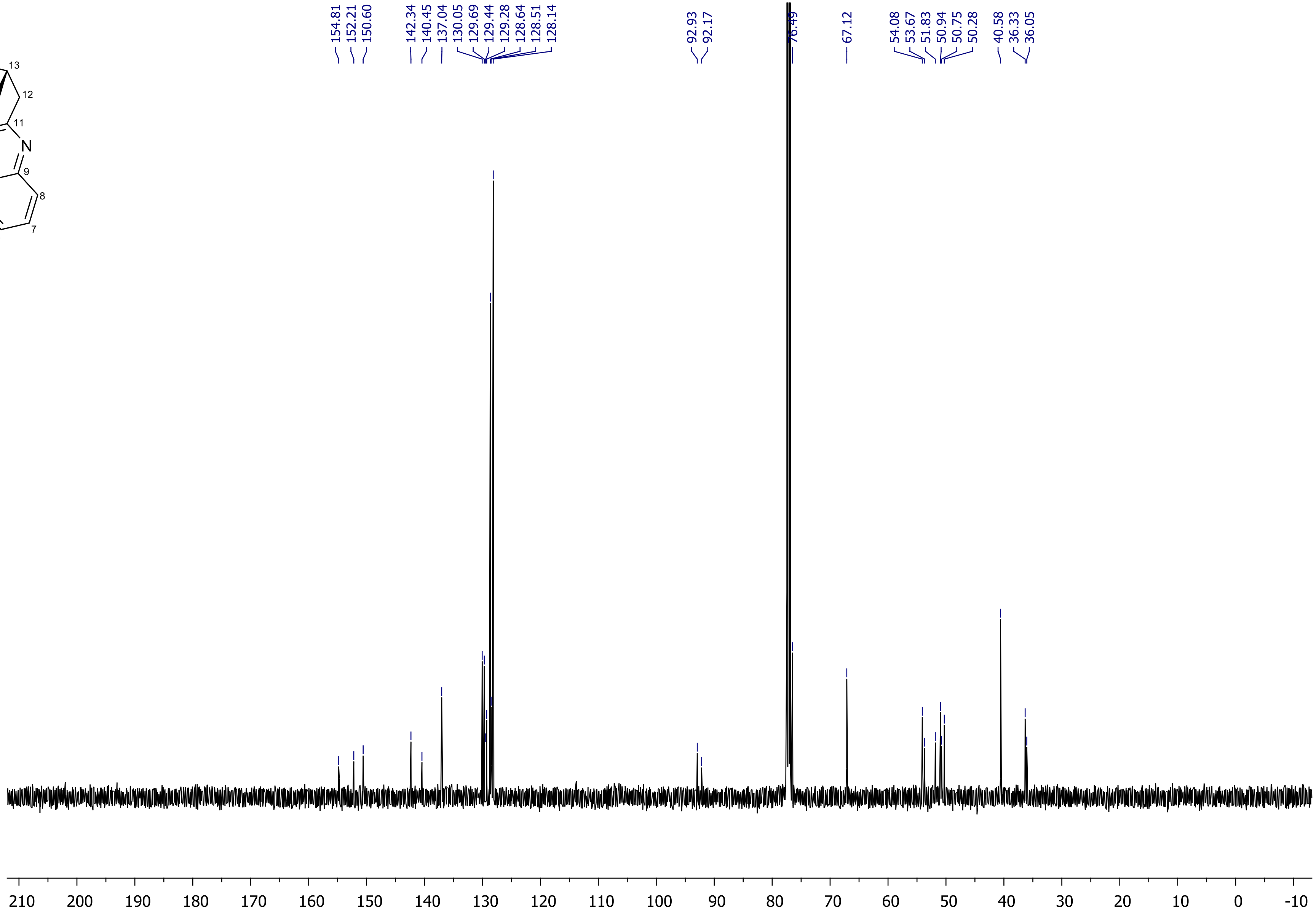
154.81
152.21
150.60
142.34
140.45
137.04
130.05
129.69
129.44
129.28
128.64
128.51
128.14
92.93
92.17
76.49
67.12
54.08
53.67
51.83
50.94
50.75
50.28
40.58
36.33
36.05
210 200 190 180 170 160 150 140 130 120 110 100 90 80 70 60 50 40 30 20 10 0 -10

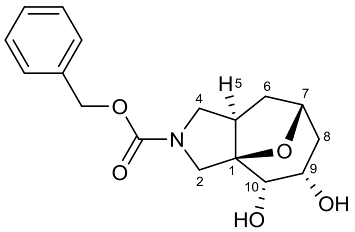
H
5
6
7
4
8
N
O
O
O
2
1
10
9
OH
HO

S25

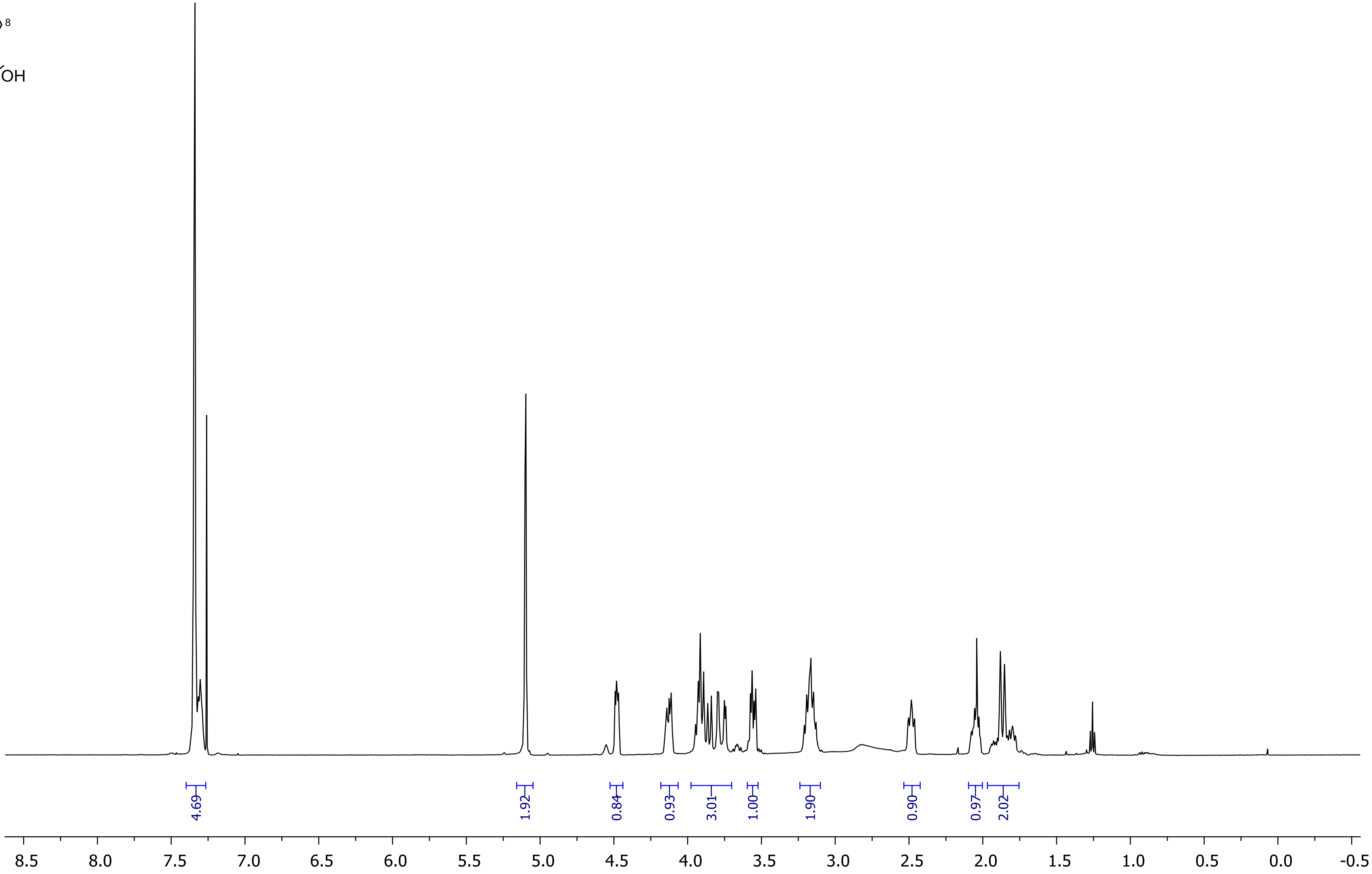
4.69
1.92
0.84
0.93
3.01
1.00
1.90
0.90
0.97
2.02
8.5
8.0
7.5
7.0
6.5
6.0
5.5
5.0
4.5
4.0
3.5
3.0
2.5
2.0
1.5
1.0
0.5
0.0
-0.5

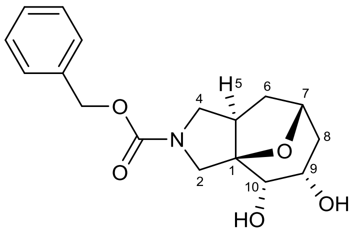
O
N
O
H 5
4
6
7
8
1
2
10
9
O
OH
HO

S25

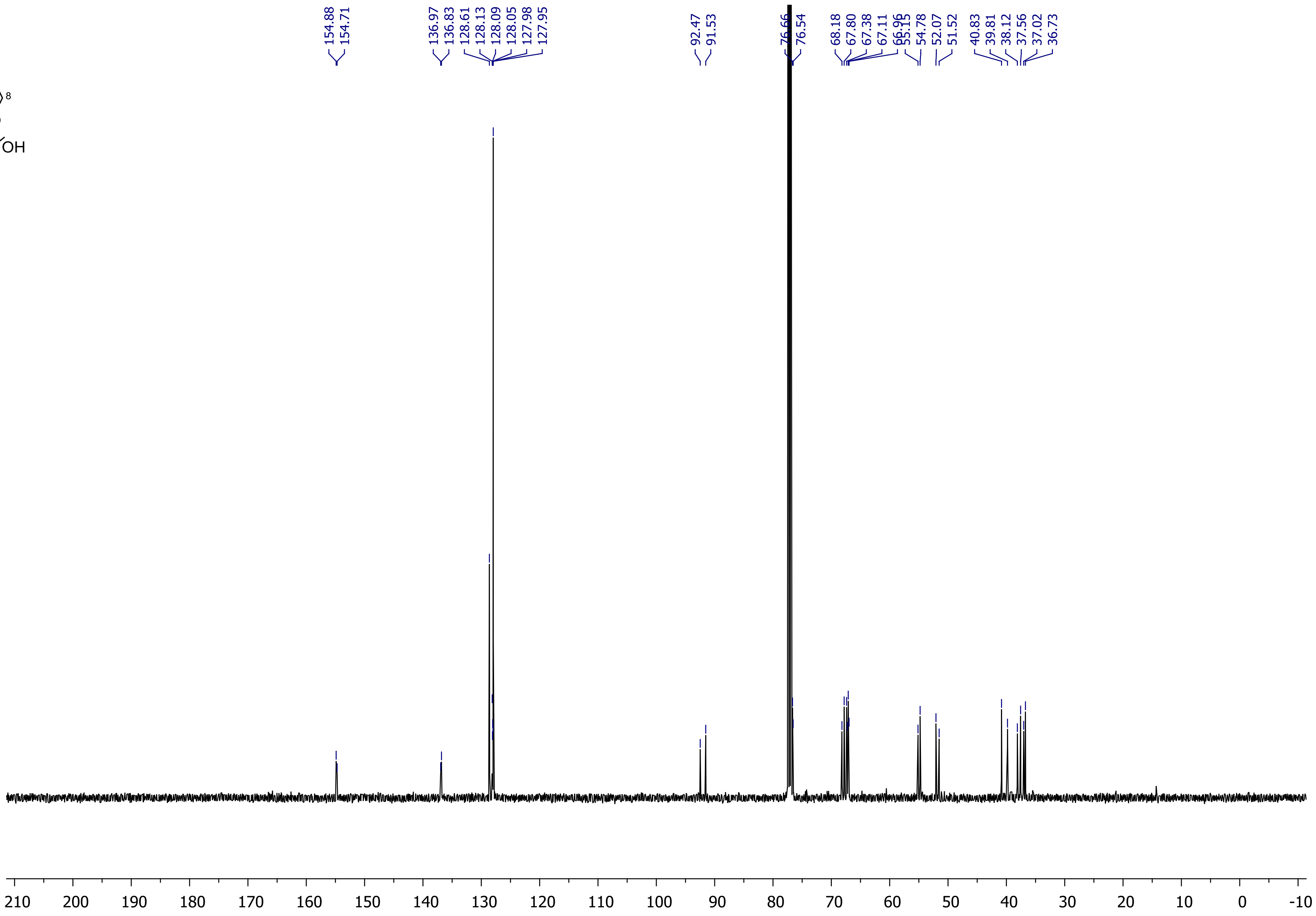
154.88
154.71
136.97
136.83
128.61
128.13
128.09
128.05
127.98
127.95
92.47
91.53
76.66
76.54
68.18
67.80
67.38
67.11
66.96
55.15
54.78
52.07
51.52
40.83
39.81
38.12
37.56
37.02
36.73
210 200 190 180 170 160 150 140 130 120 110 100 90 80 70 60 50 40 30 20 10 0 -10

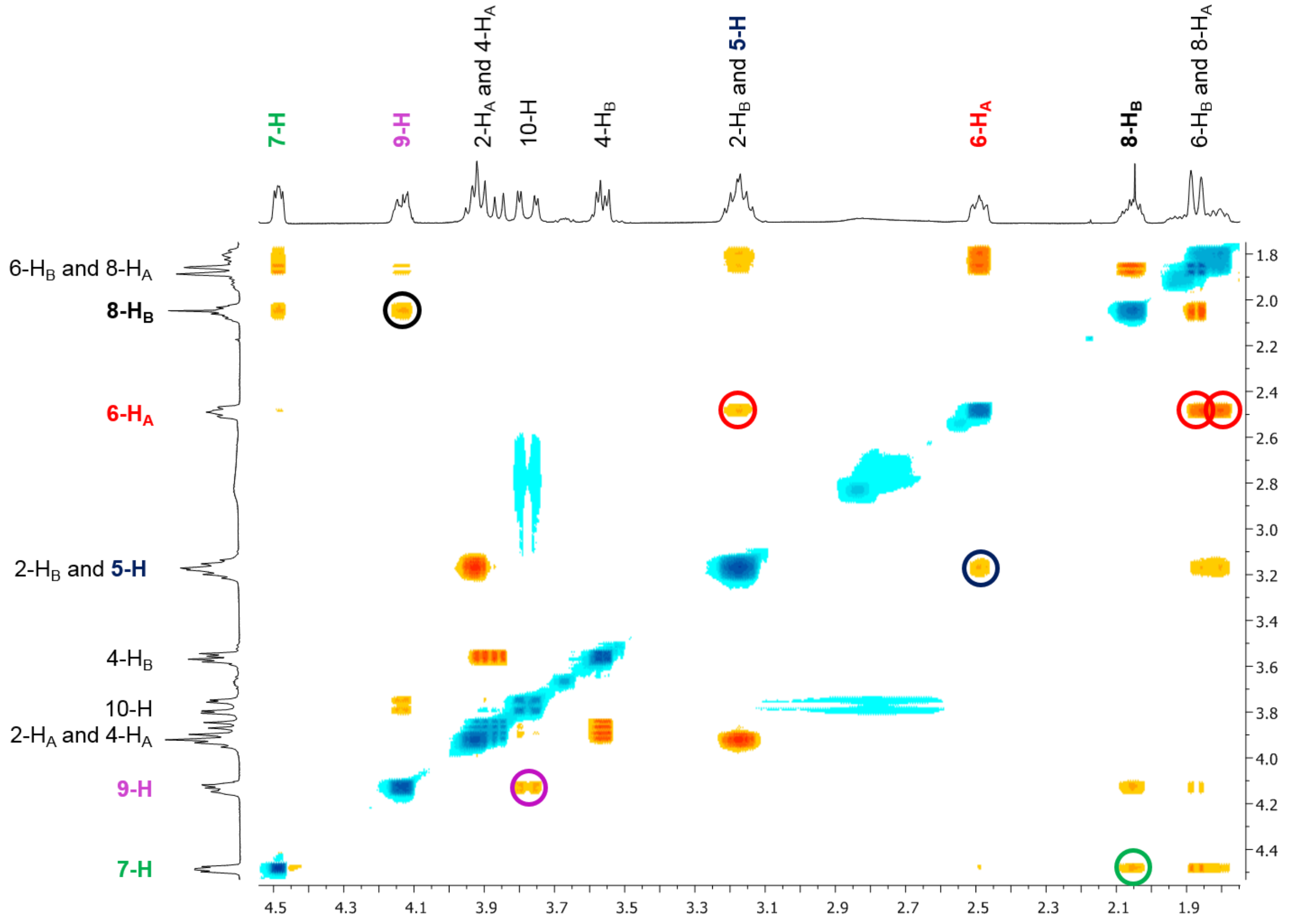

S25 NOESY correlations:

5-H: 6_A

6-H$_A$: 5-H; 8-H$_A$

7-H: 6-H$_B$; 8-H$_B$

8-H$_B$: 7-H; 9-H

9-H: 10-H

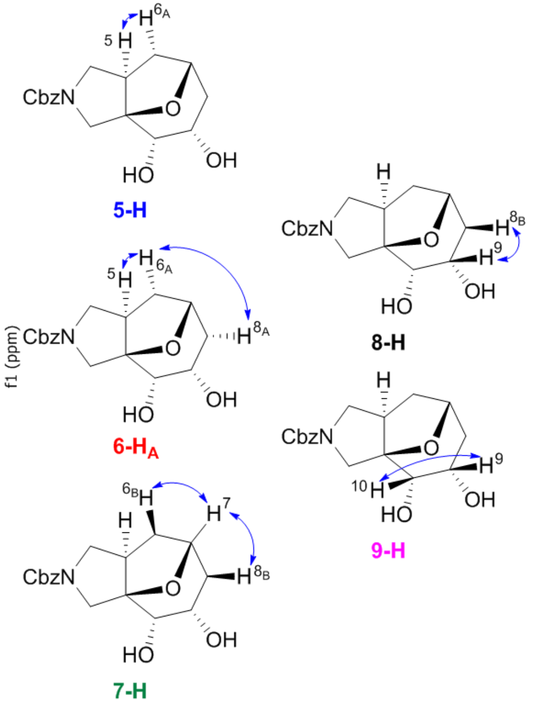

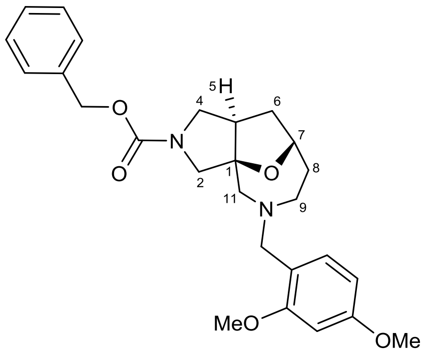

15

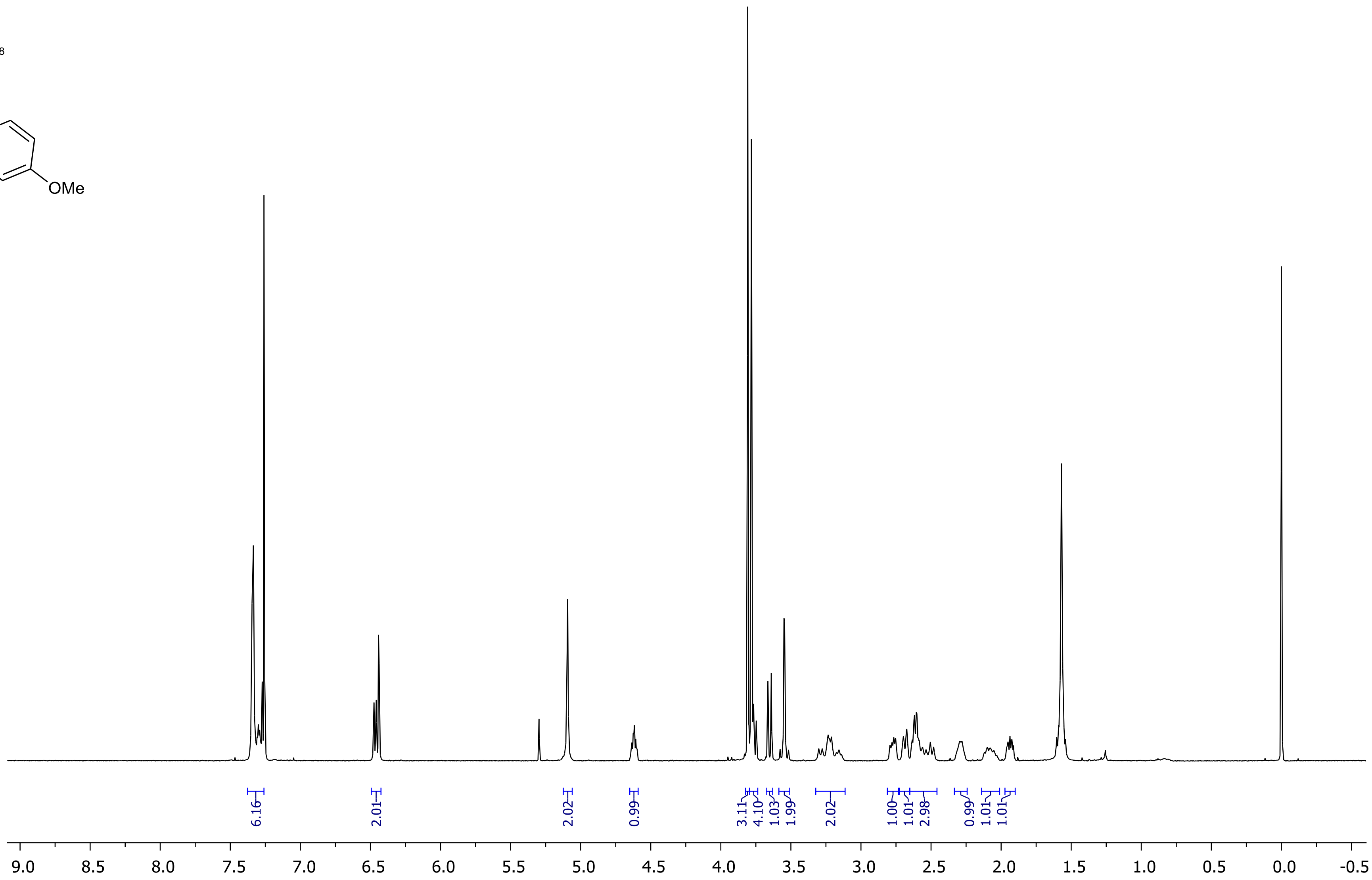
6.16
2.01
2.02
0.99
3.11
4.10
1.03
1.99
2.02
1.00
1.01
2.98
0.99
1.01
1.01
9.0 8.5 8.0 7.5 7.0 6.5 6.0 5.5 5.0 4.5 4.0 3.5 3.0 2.5 2.0 1.5 1.0 0.5 0.0 -0.5

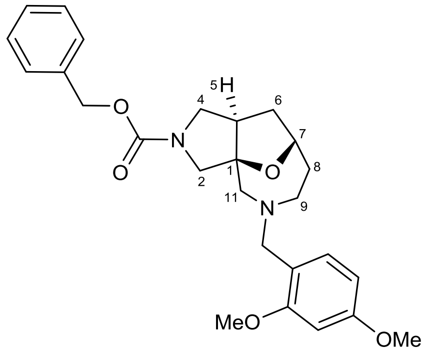

5H
4
6
7
8
9
11
2
1
N
O
O
O
N
MeO
OMe

15

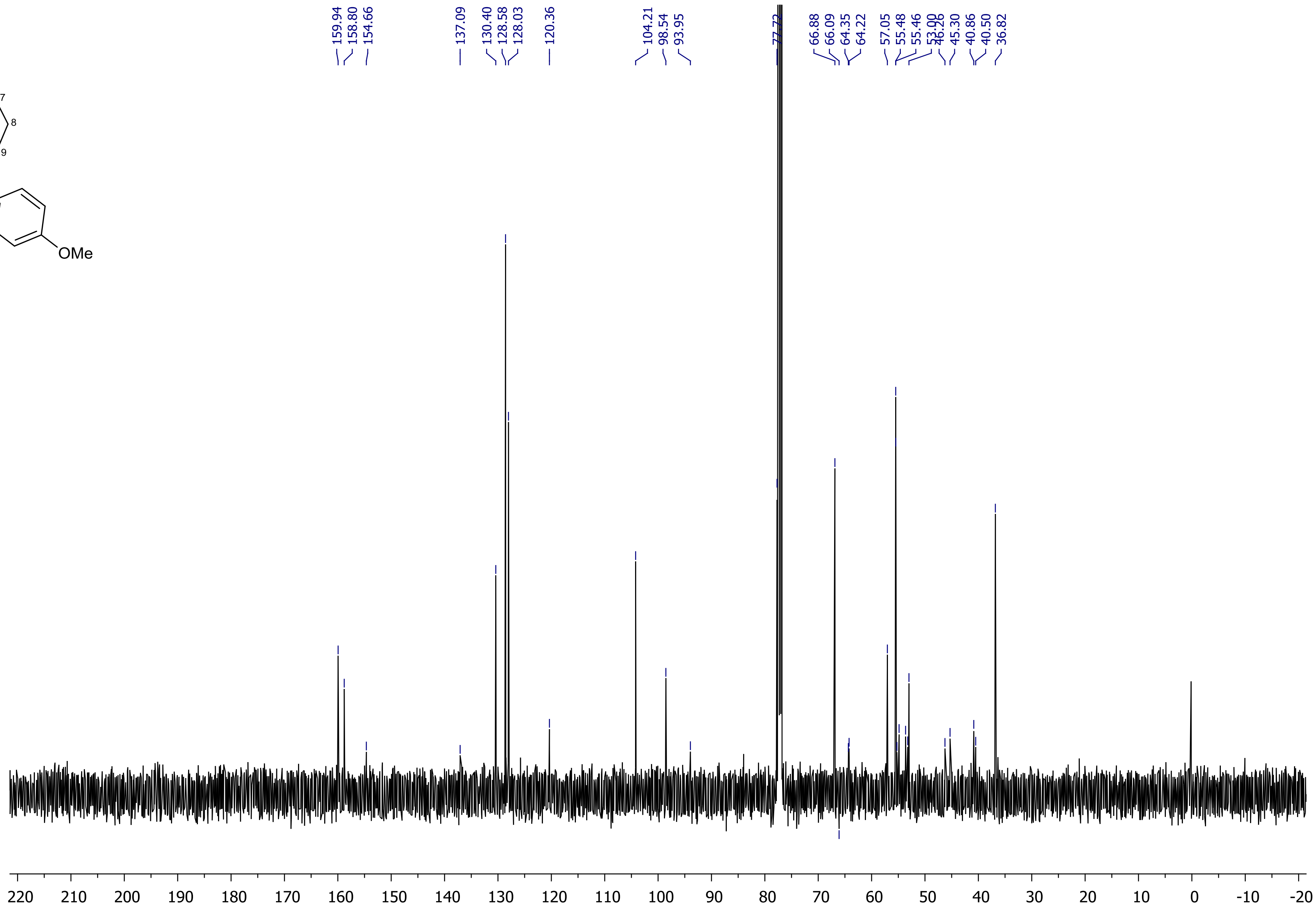

159.94
158.80
154.66
137.09
130.40
128.58
128.03
120.36
104.21
98.54
93.95
77.72
66.88
66.09
64.35
64.22
57.05
55.48
55.46
53.00
46.26
45.30
40.86
40.50
36.82
220 210 200 190 180 170 160 150 140 130 120 110 100 90 80 70 60 50 40 30 20 10 0 -10 -20

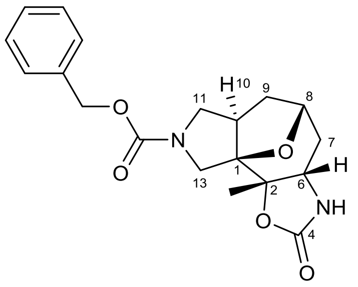

O
O
N
H
10
11
9
8
O
7
H
1
6
13
2
NH
O
4
O

16

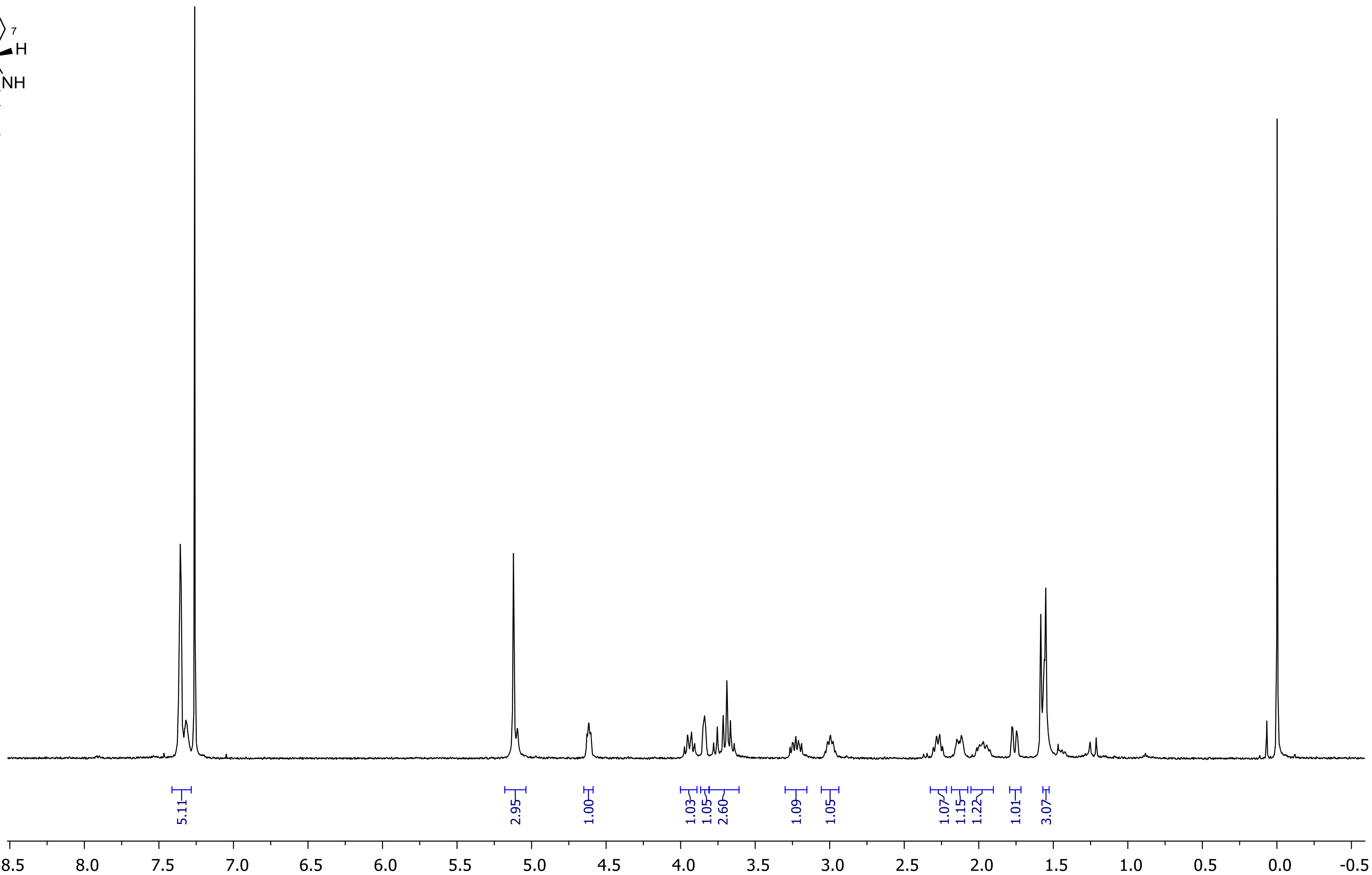

5.11
2.95
1.00
1.03
1.05
2.60
1.09
1.05
1.07
1.15
1.22
1.01
3.07
8.5
8.0
7.5
7.0
6.5
6.0
5.5
5.0
4.5
4.0
3.5
3.0
2.5
2.0
1.5
1.0
0.5
0.0
-0.5

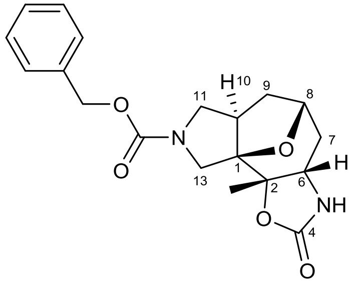
H
10
9
8
11
7
O
N
H
O
O
1
13
2
6
NH
O
4
O
O

16

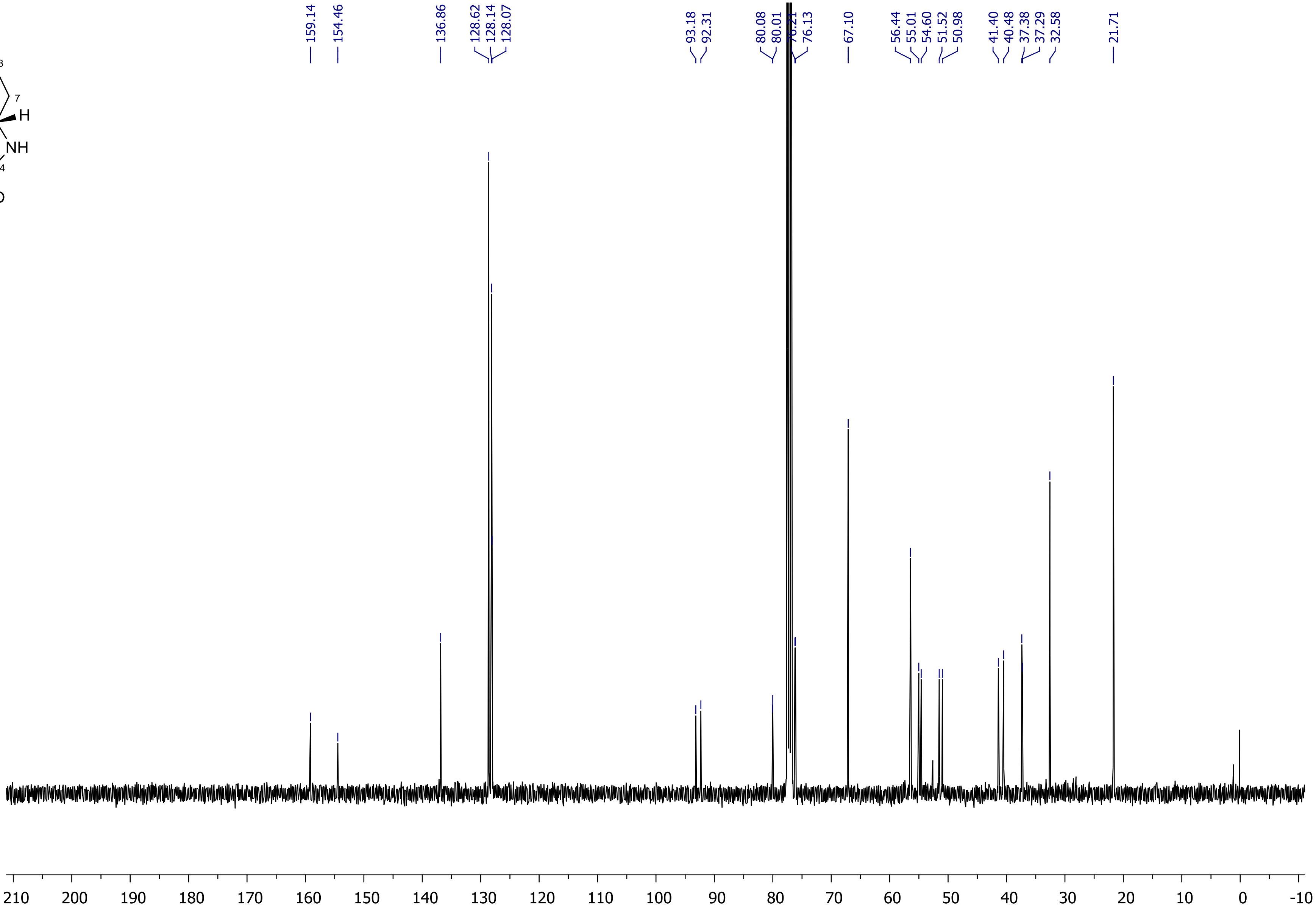
159.14
154.46
136.86
128.62
128.14
128.07
93.18
92.31
80.08
80.01
76.13
67.10
56.44
55.01
54.60
51.52
50.98
41.40
40.48
37.38
37.29
32.58
21.71
210 200 190 180 170 160 150 140 130 120 110 100 90 80 70 60 50 40 30 20 10 0 -10

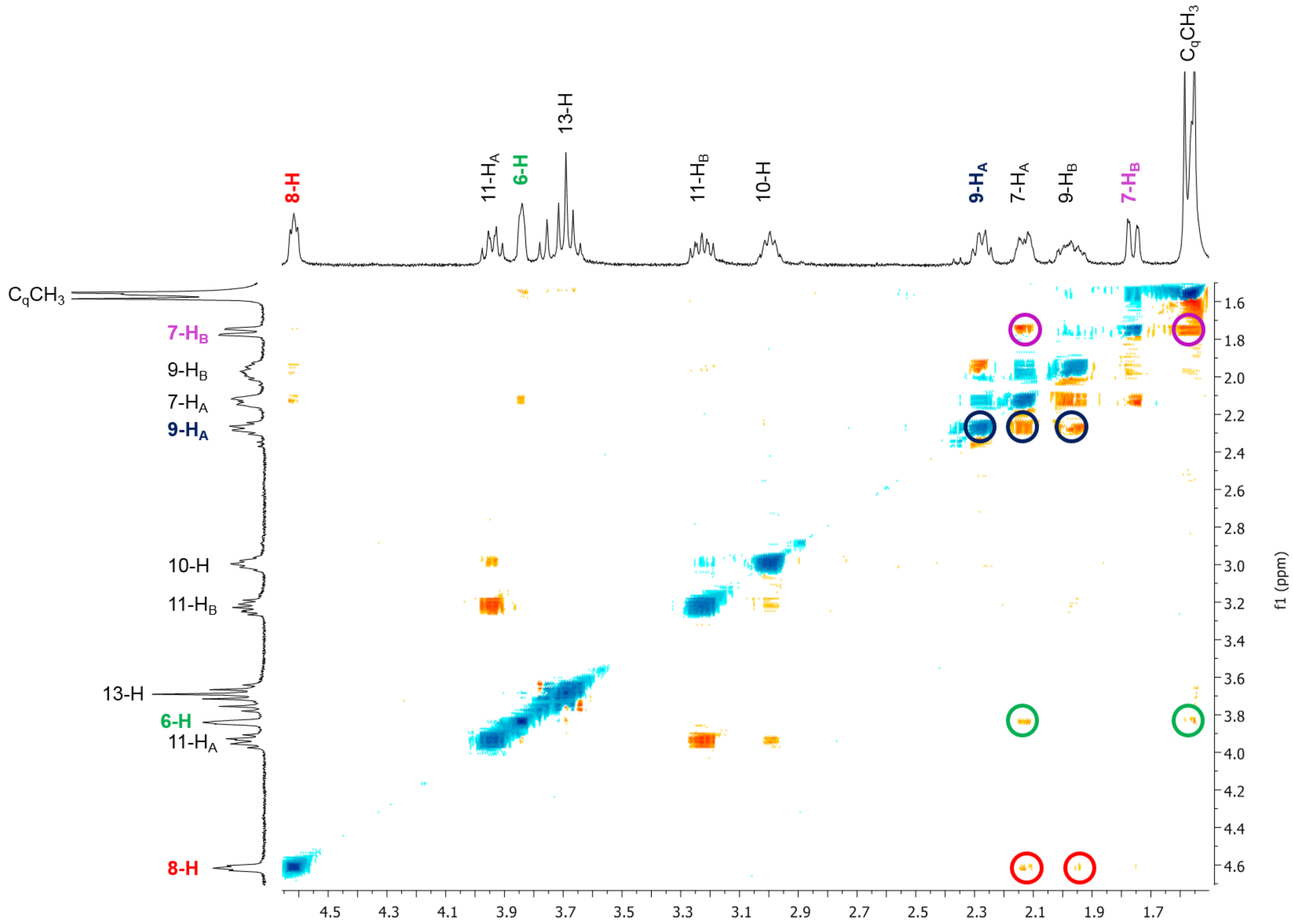

16 NOESY correlations:

6-H: 7-H$_A$; C$_q$CH$_3$

7-H$_B$: 7-H$_A$; C$_q$CH$_3$

8-H: 7-H$_B$; 9-H$_B$

9-H$_B$: 7-H$_A$; 9-H$_B$

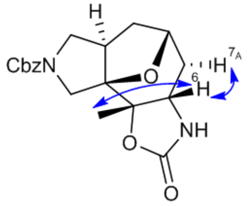

6-H

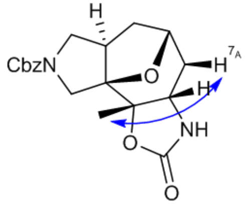

7-H$_B$

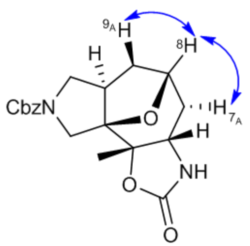

8-H

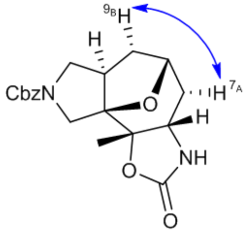

9-H$_B$

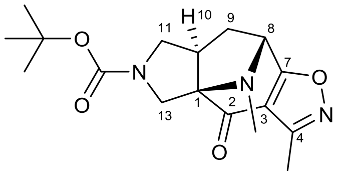
O
N
H 10
9
8
11
7
O
N
N
13
1
2
3
4
O
O

S26

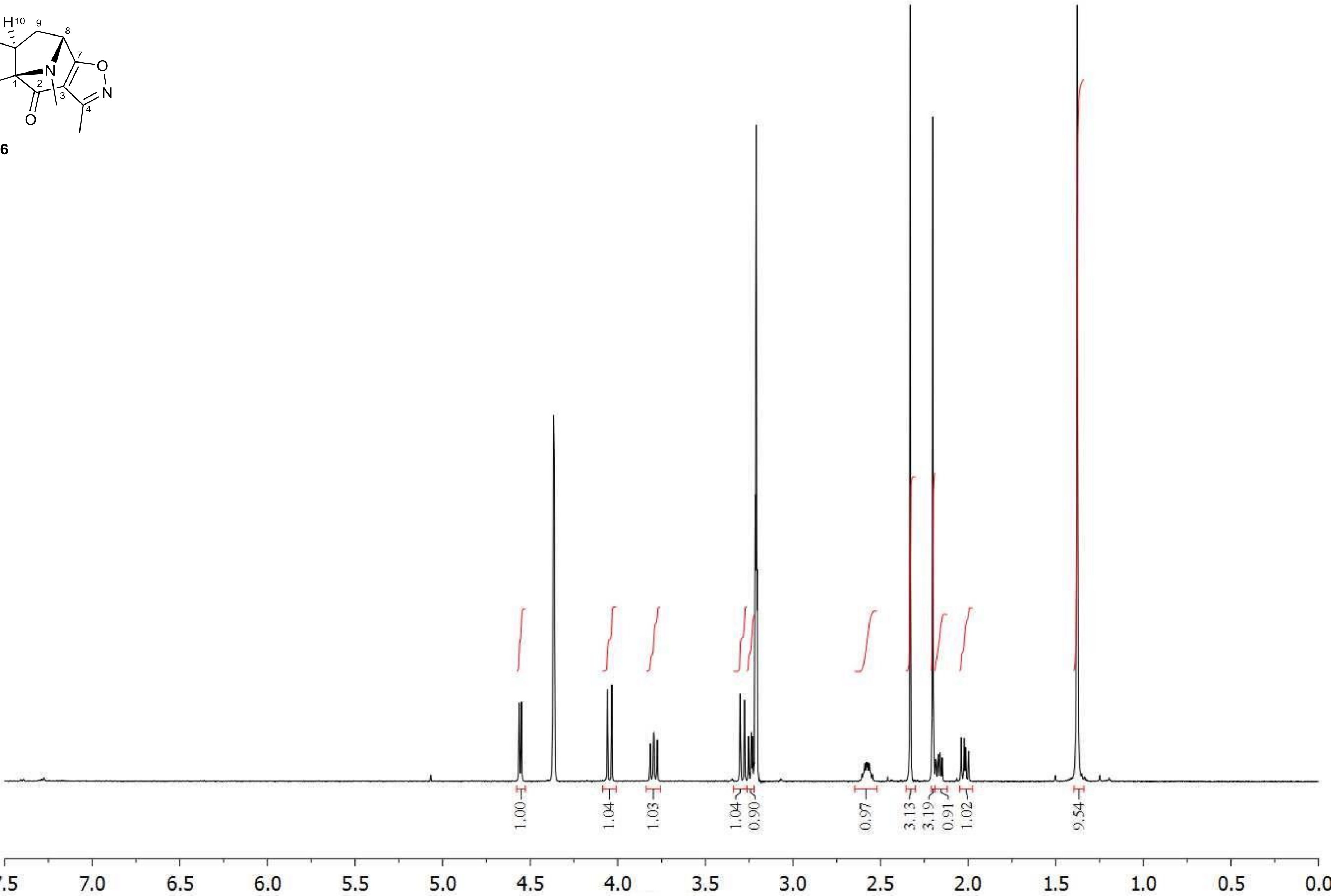
1.00
1.04
1.03
1.04
0.90
0.97
3.13
3.19
0.91
1.02
9.54
7.5
7.0
6.5
6.0
5.5
5.0
4.5
4.0
3.5
3.0
2.5
2.0
1.5
1.0
0.5
0.0

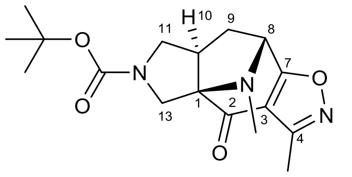
H
10
9
8
11
7
O
N
O
O
N
2
1
13
3
4
N
O

S26

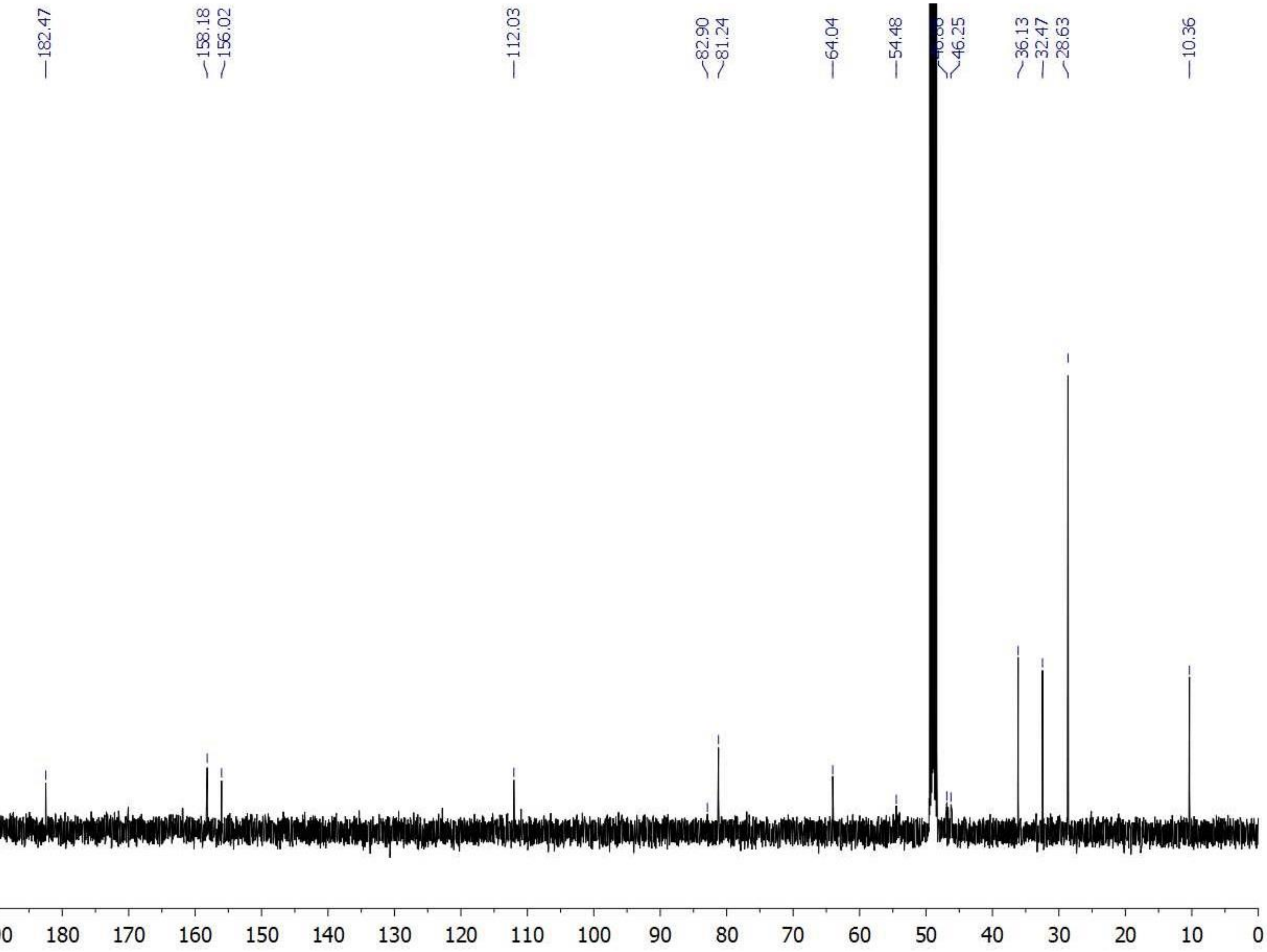
182.47
158.18
156.02
112.03
82.90
81.24
64.04
54.48
46.25
36.13
32.47
28.63
10.36
210
200
190
180
170
160
150
140
130
120
110
100
90
80
70
60
50
40
30
20
10
0

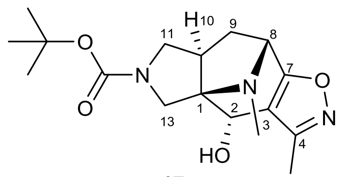
O
O
N
H10
9
8
7
O
N
11
1
2
3
4
13
HO

17

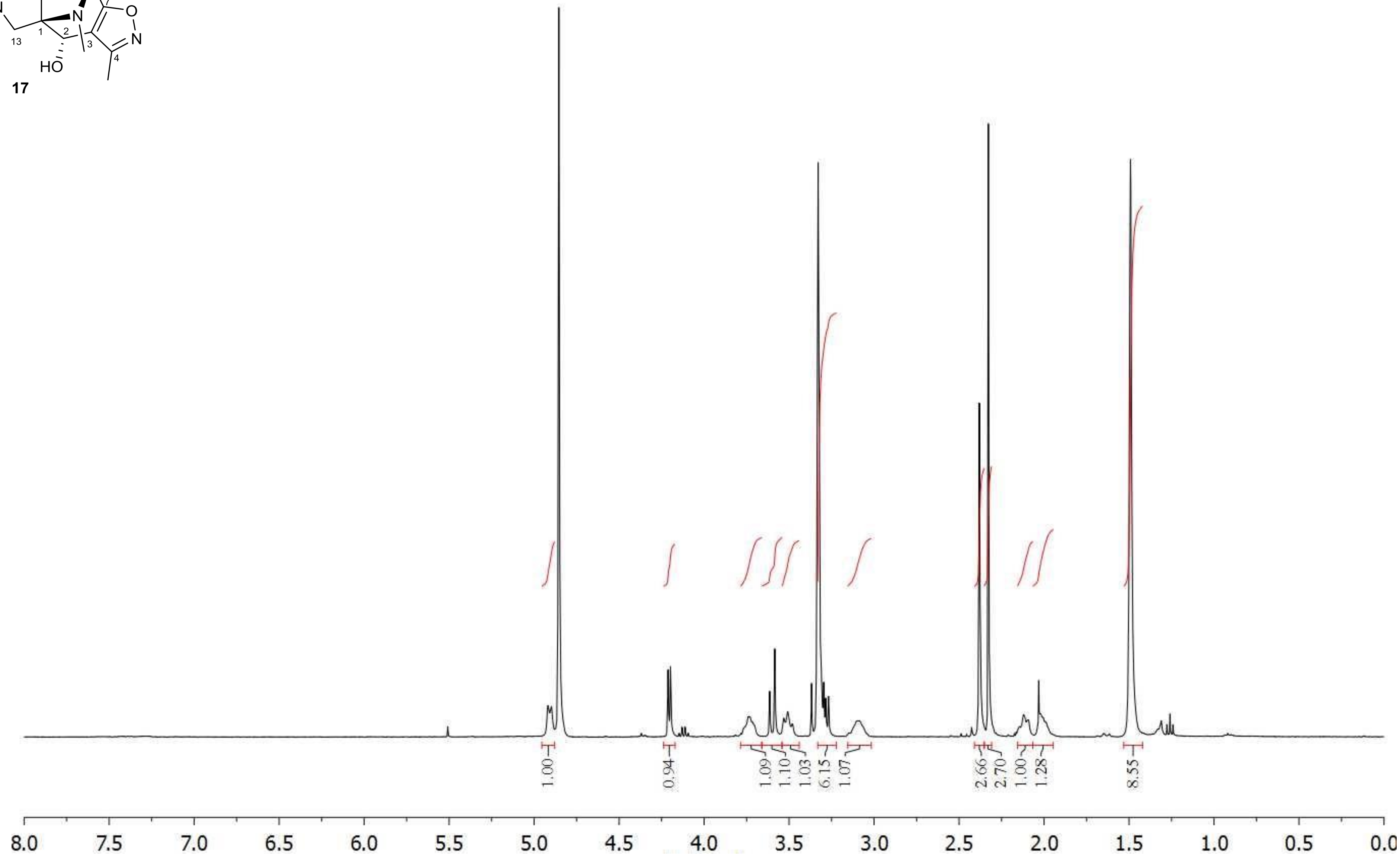
1.00
0.94
1.09
1.10
1.03
6.15
1.07
2.66
2.70
1.00
1.28
8.55
8.0
7.5
7.0
6.5
6.0
5.5
5.0
4.5
4.0
3.5
3.0
2.5
2.0
1.5
1.0
0.5
0.0

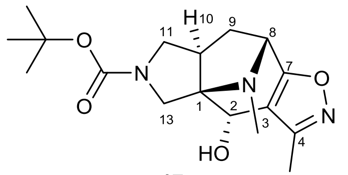

17

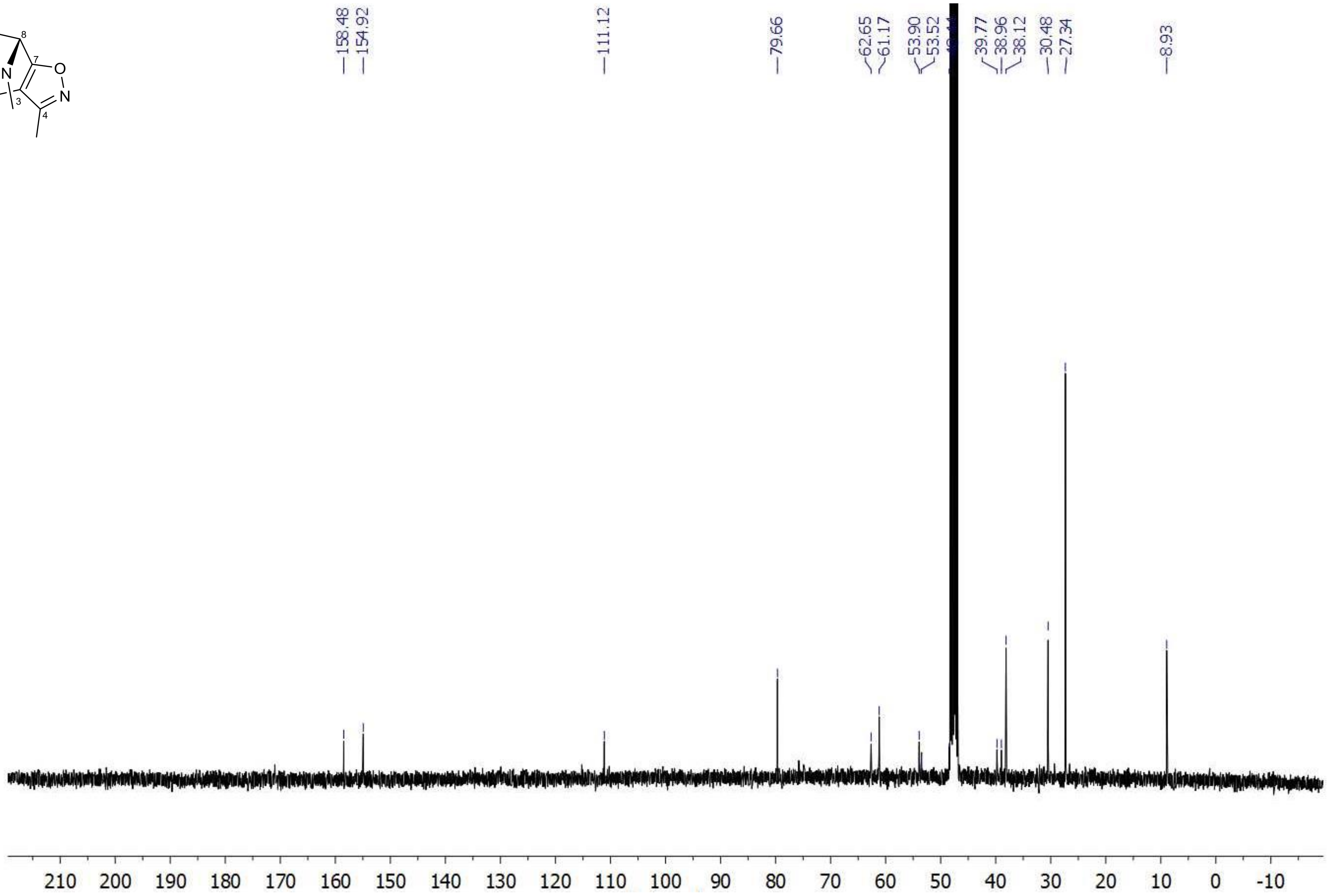

158.48
154.92
111.12
79.66
62.65
61.17
53.90
53.52
39.77
38.96
38.12
30.48
27.34
8.93
210 200 190 180 170 160 150 140 130 120 110 100 90 80 70 60 50 40 30 20 10 0 -10

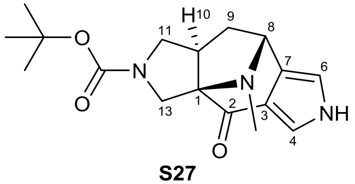
H
10
9
8
11
7
6
O
N
N
NH
1
2
3
13
4
O
O

S27

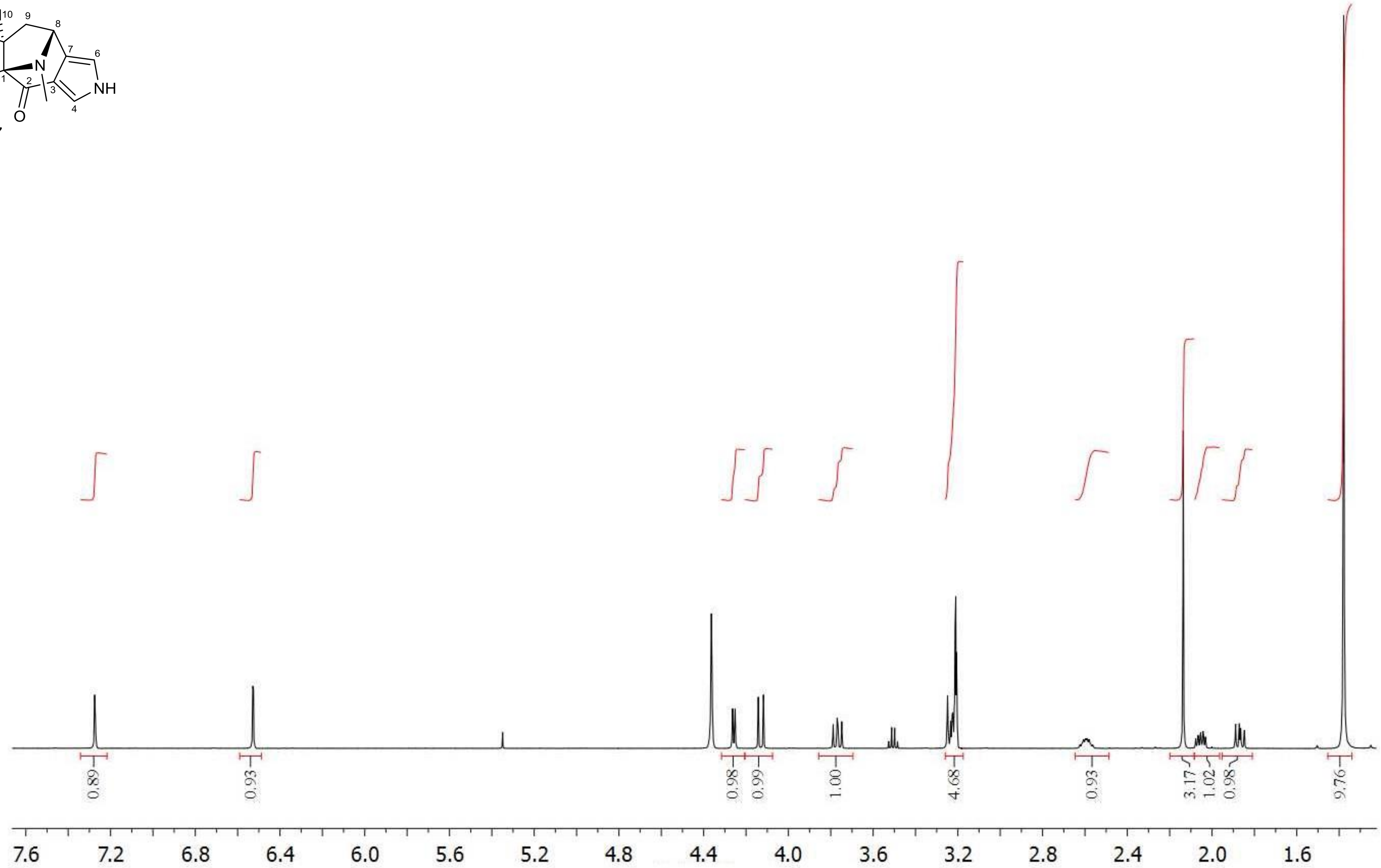
0.89
0.93
0.98
0.99
1.00
4.68
0.93
3.17
1.02
0.98
9.76
7.6
7.2
6.8
6.4
6.0
5.6
5.2
4.8
4.4
4.0
3.6
3.2
2.8
2.4
2.0
1.6

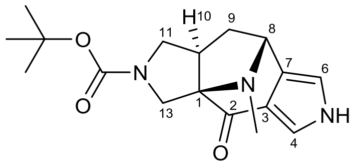

O
O
O
N
H10
9
8
11
7
6
N
NH
13
1
2
3
4
O

S27

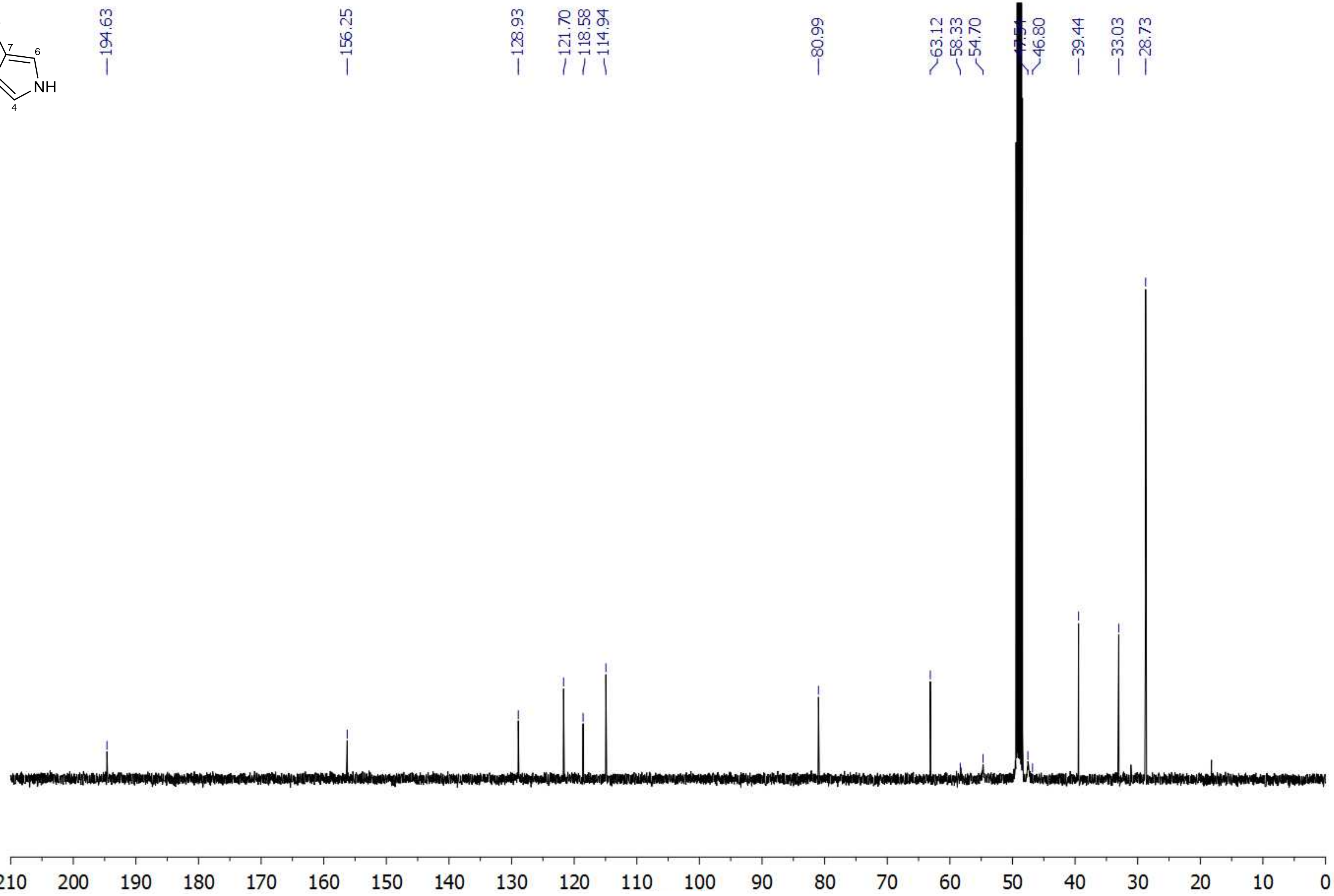

194.63
156.25
128.93
121.70
118.58
114.94
80.99
63.12
58.33
54.70
46.80
39.44
33.03
28.73
210 200 190 180 170 160 150 140 130 120 110 100 90 80 70 60 50 40 30 20 10 0

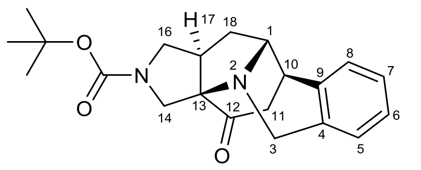

19

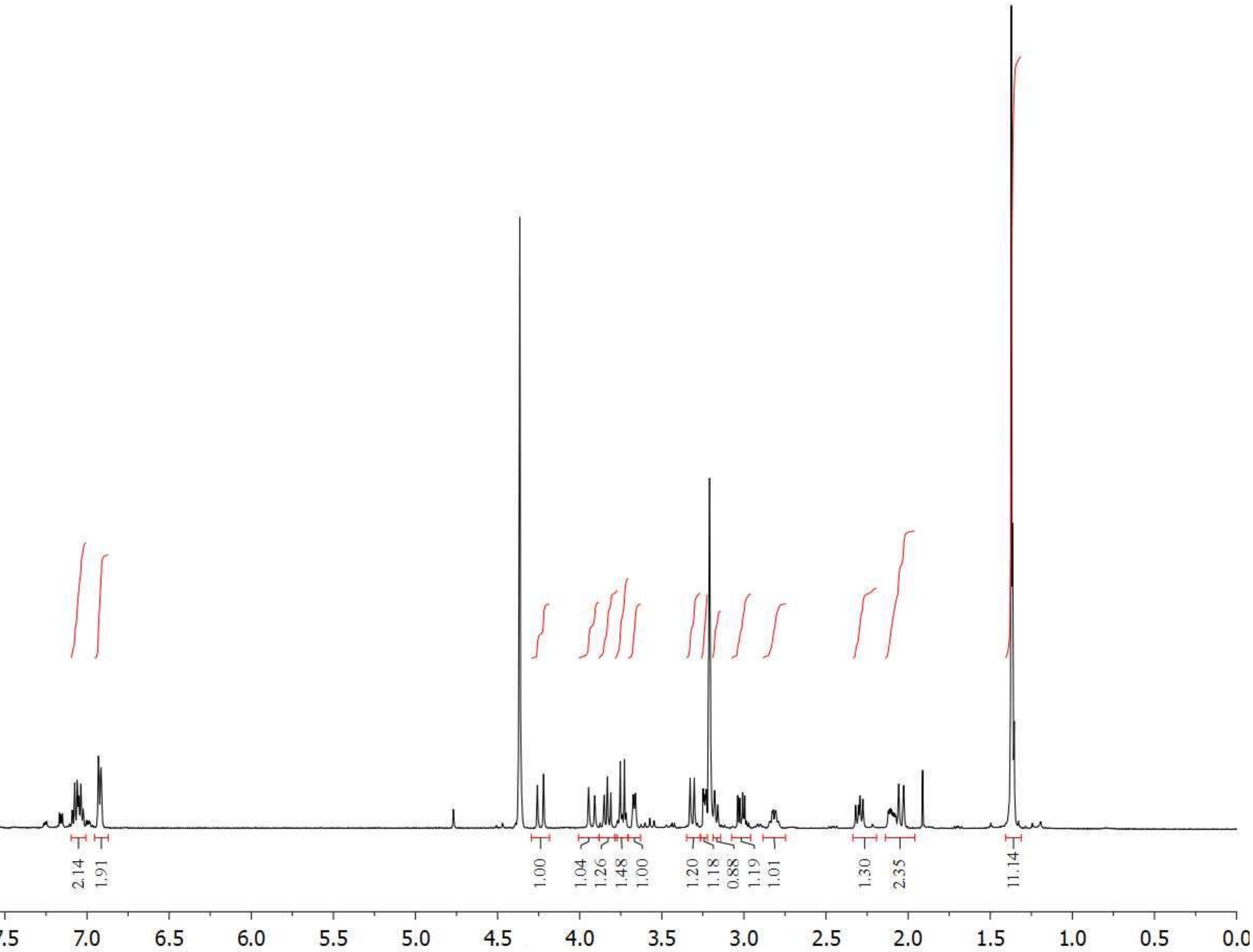
2.14
1.91
1.00
1.04
1.26
1.48
1.00
1.20
1.18
0.88
1.19
1.01
1.30
2.35
11.14
8.5 8.0 7.5 7.0 6.5 6.0 5.5 5.0 4.5 4.0 3.5 3.0 2.5 2.0 1.5 1.0 0.5 0.0

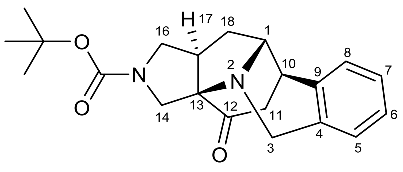

19

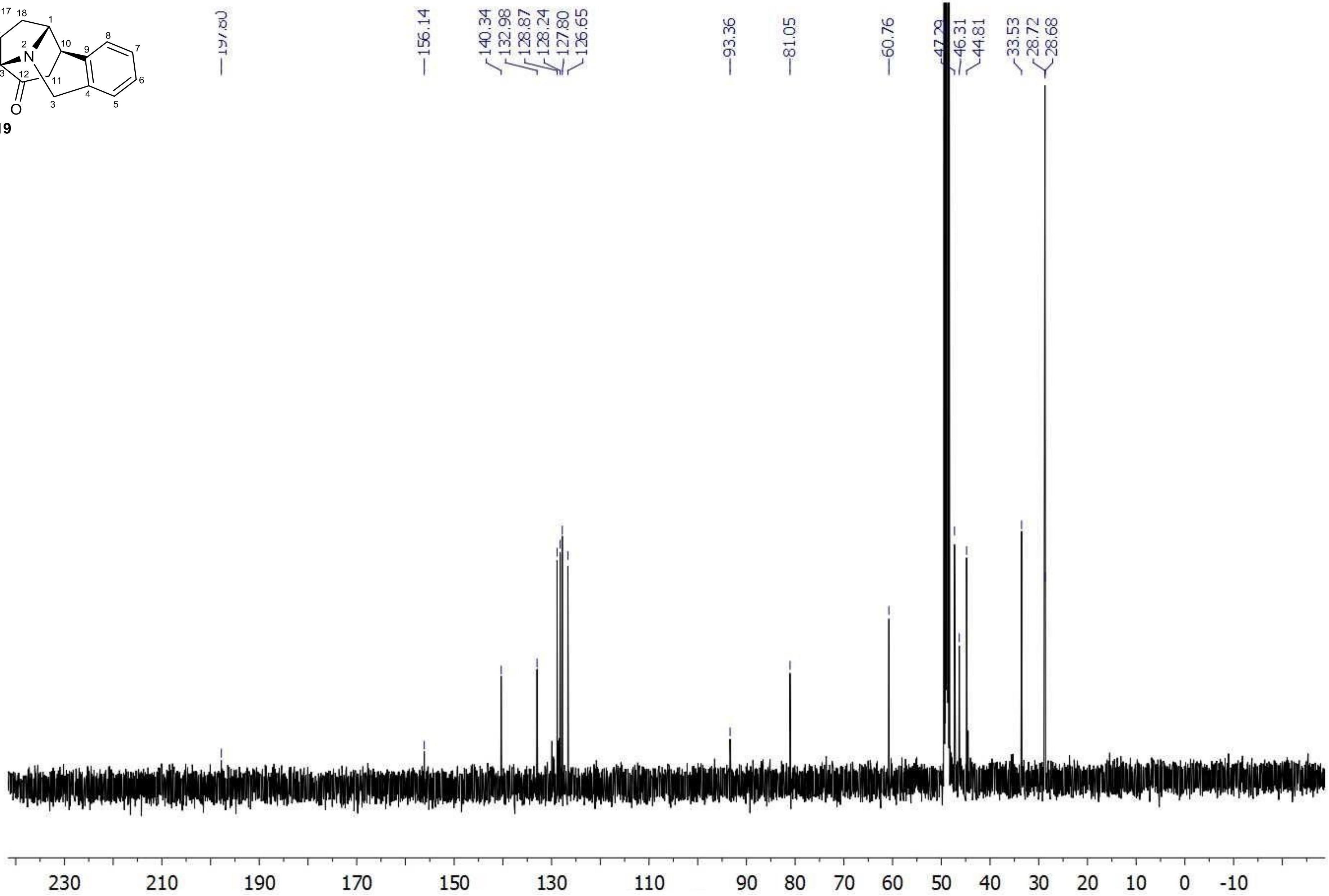
197.80
156.14
140.34
132.98
128.87
128.24
127.80
126.65
93.36
81.05
60.76
47.29
46.31
44.81
33.53
28.72
28.68
230 210 190 170 150 130 110 90 80 70 60 50 40 30 20 10 0 -10

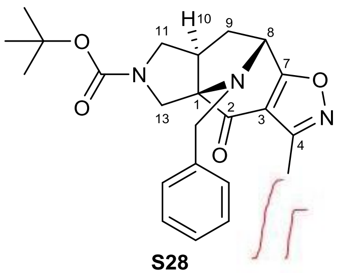

S28

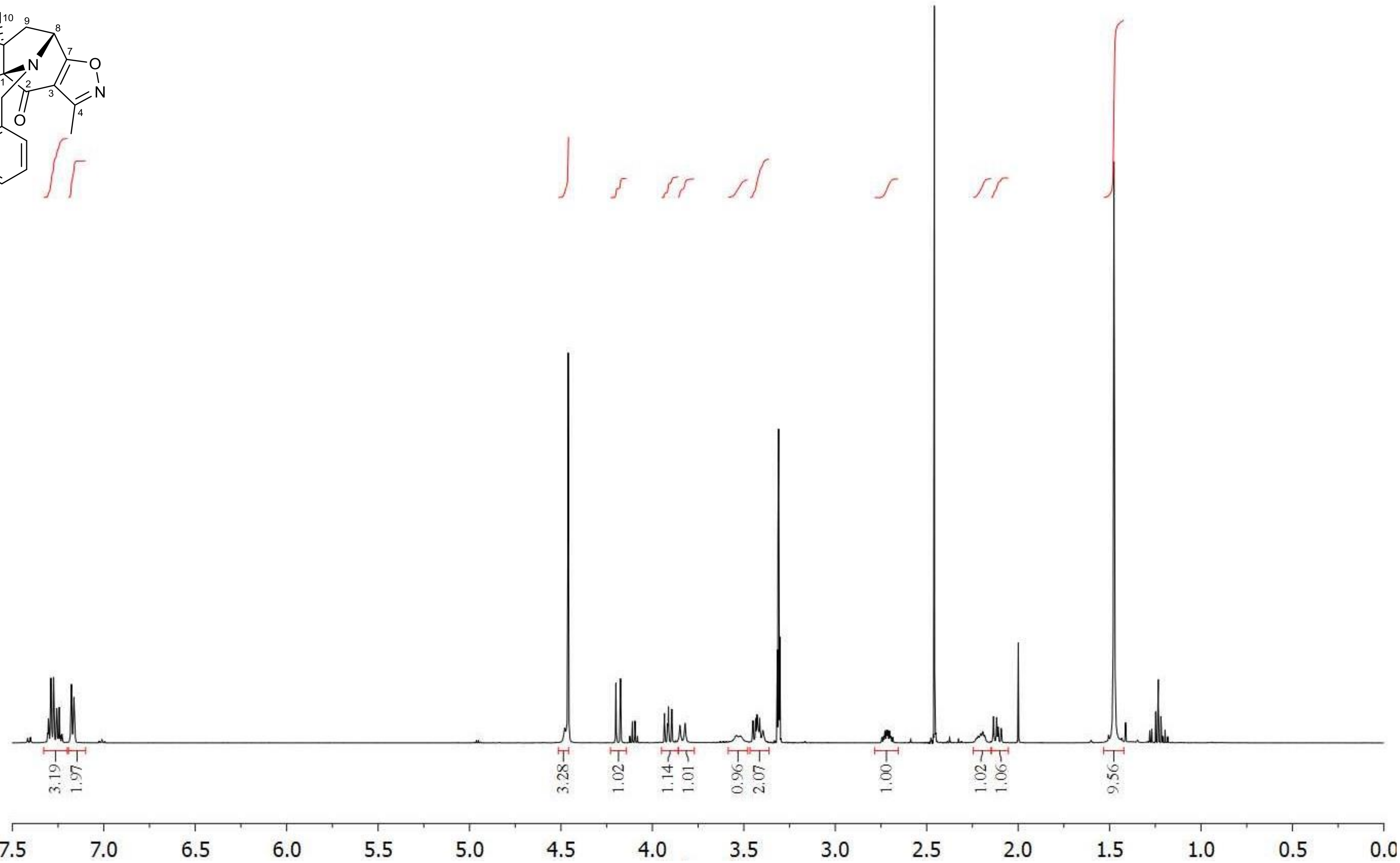

3.19
1.97
3.28
1.02
1.14
1.01
0.96
2.07
1.00
1.02
1.06
9.56
7.5
7.0
6.5
6.0
5.5
5.0
4.5
4.0
3.5
3.0
2.5
2.0
1.5
1.0
0.5
0.0

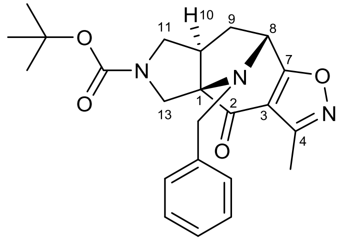

O
N
O
H
10
11
9
8
7
O
N
1
2
3
4
13
O

S28

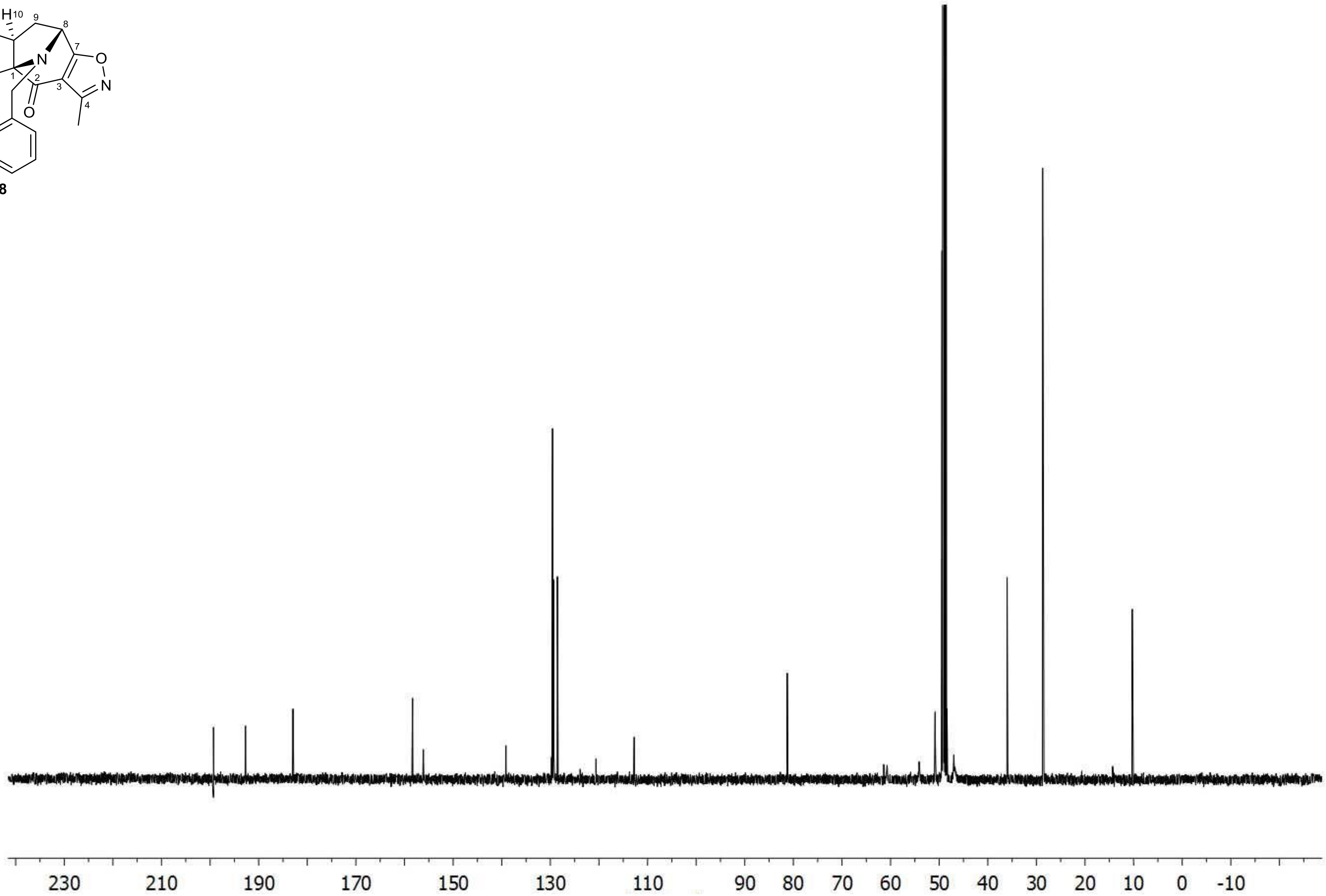

230
210
190
170
150
130
110
90
80
70
60
50
40
30
20
10
0
-10

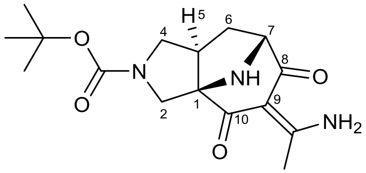

H
5
4
6
7
8
O
N
O
NH
O
2
1
10
9
NH2
O

18

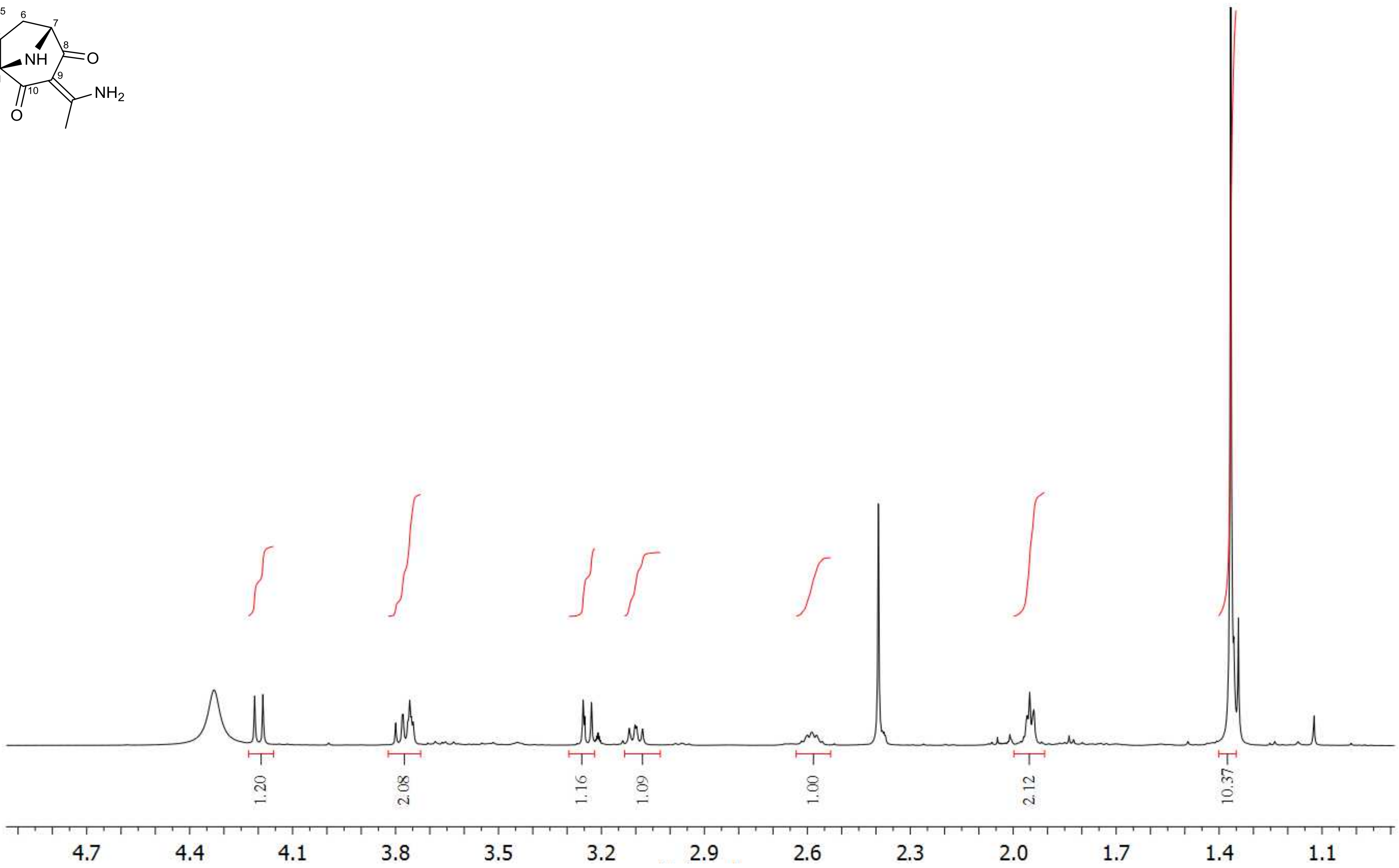

1.20
2.08
1.16
1.09
1.00
2.12
10.37
4.7
4.4
4.1
3.8
3.5
3.2
2.9
2.6
2.3
2.0
1.7
1.4
1.1

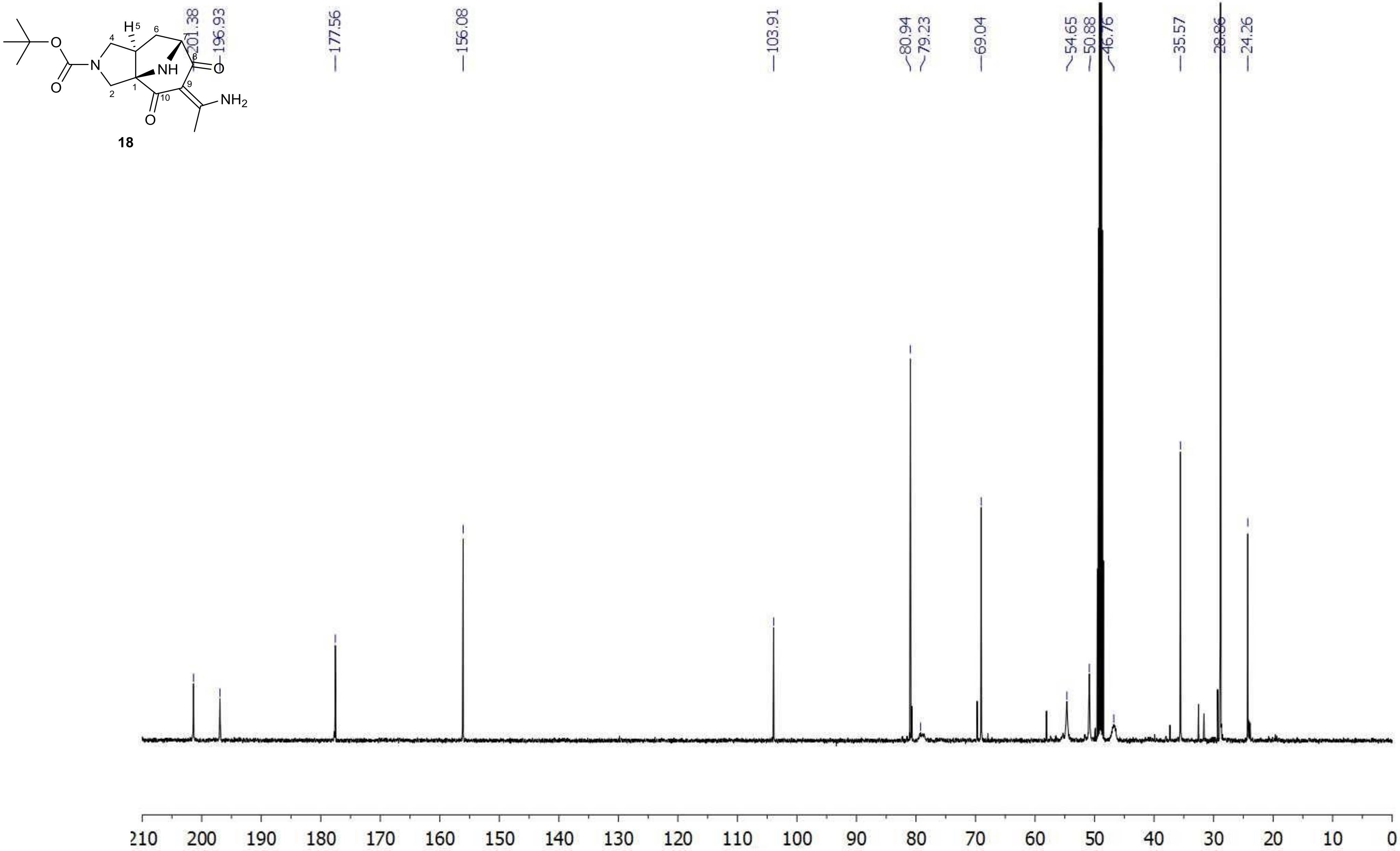

18
201.38
196.93
177.56
156.08
103.91
80.94
79.23
69.04
54.65
50.88
46.76
35.57
28.86
24.26
210 200 190 180 170 160 150 140 130 120 110 100 90 80 70 60 50 40 30 20 10 0

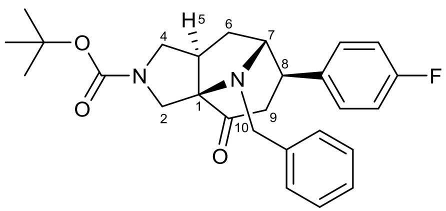

S29

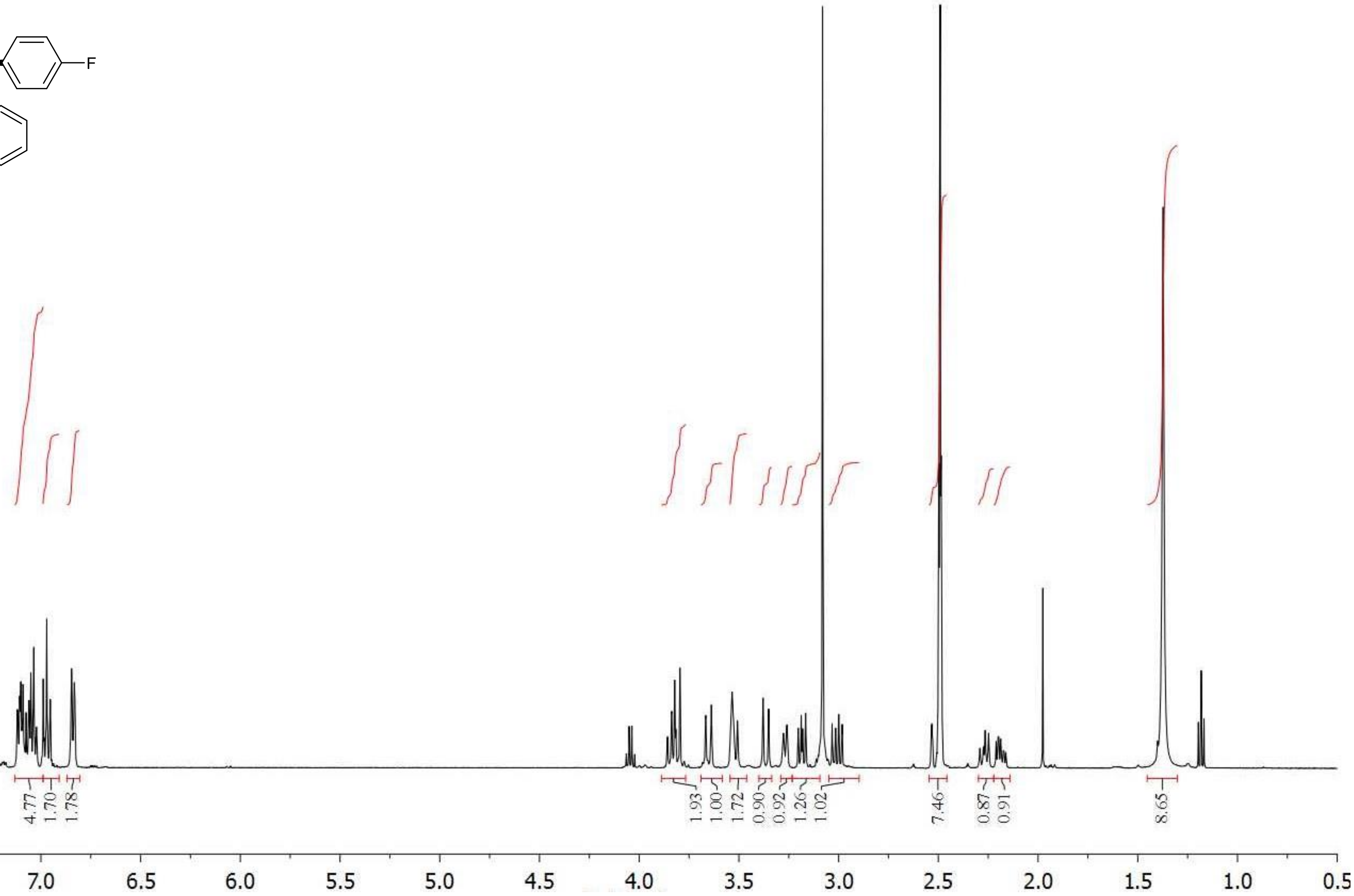
4.77
1.70
1.78
1.93
1.00
1.72
0.90
0.92
1.26
1.02
7.46
0.87
0.91
8.65
7.5 7.0 6.5 6.0 5.5 5.0 4.5 4.0 3.5 3.0 2.5 2.0 1.5 1.0 0.5

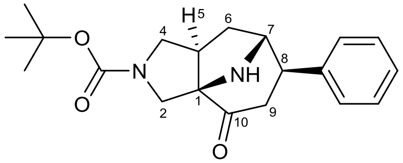

H5
NH
N
O
1
2
4
6
7
8
9
10

S30

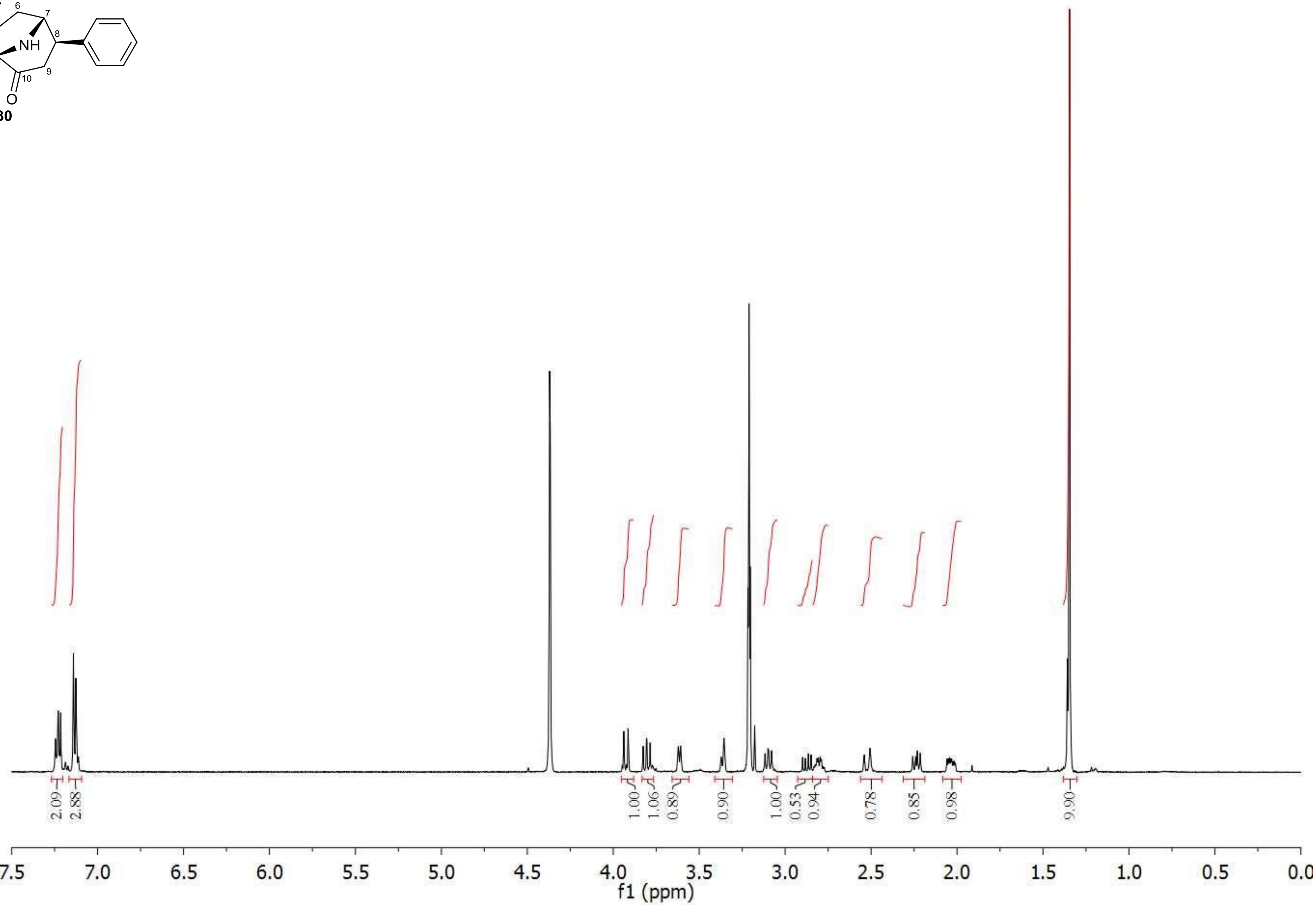

2.09
2.88
1.00
1.06
0.89
0.90
1.00
0.53
0.94
0.78
0.85
0.98
9.90
7.5
7.0
6.5
6.0
5.5
5.0
4.5
4.0
3.5
3.0
2.5
2.0
1.5
1.0
0.5
0.0
f1 (ppm)

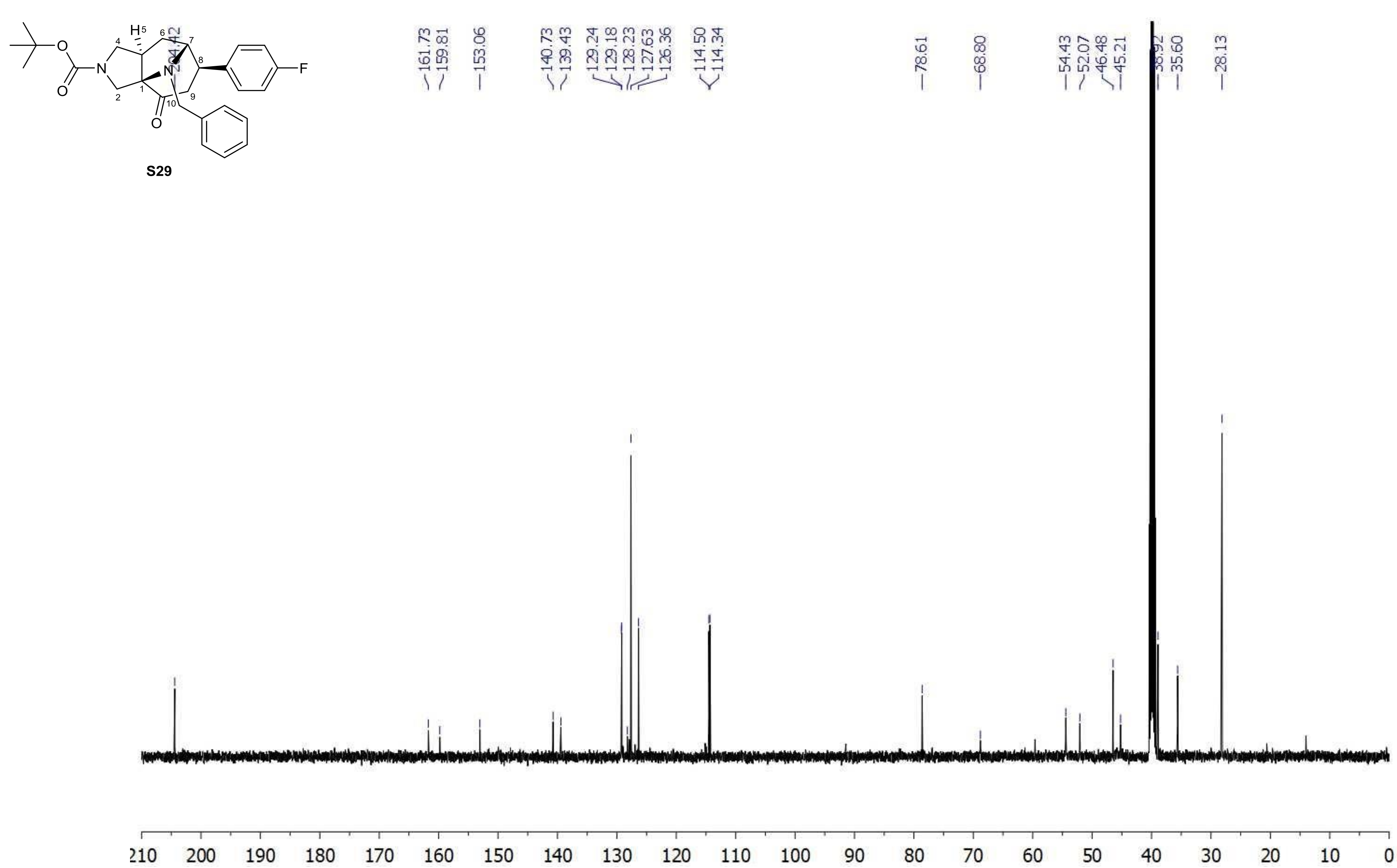

S29
161.73
159.81
153.06
140.73
139.43
129.24
129.18
128.23
127.63
126.36
114.50
114.34
78.61
68.80
54.43
52.07
46.48
45.21
35.60
28.13
210
200
190
180
170
160
150
140
130
120
110
100
90
80
70
60
50
40
30
20
10
0

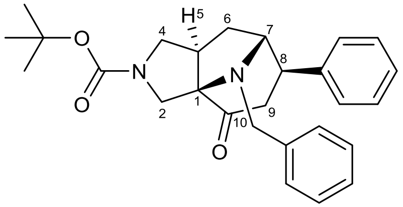

carried forward crude

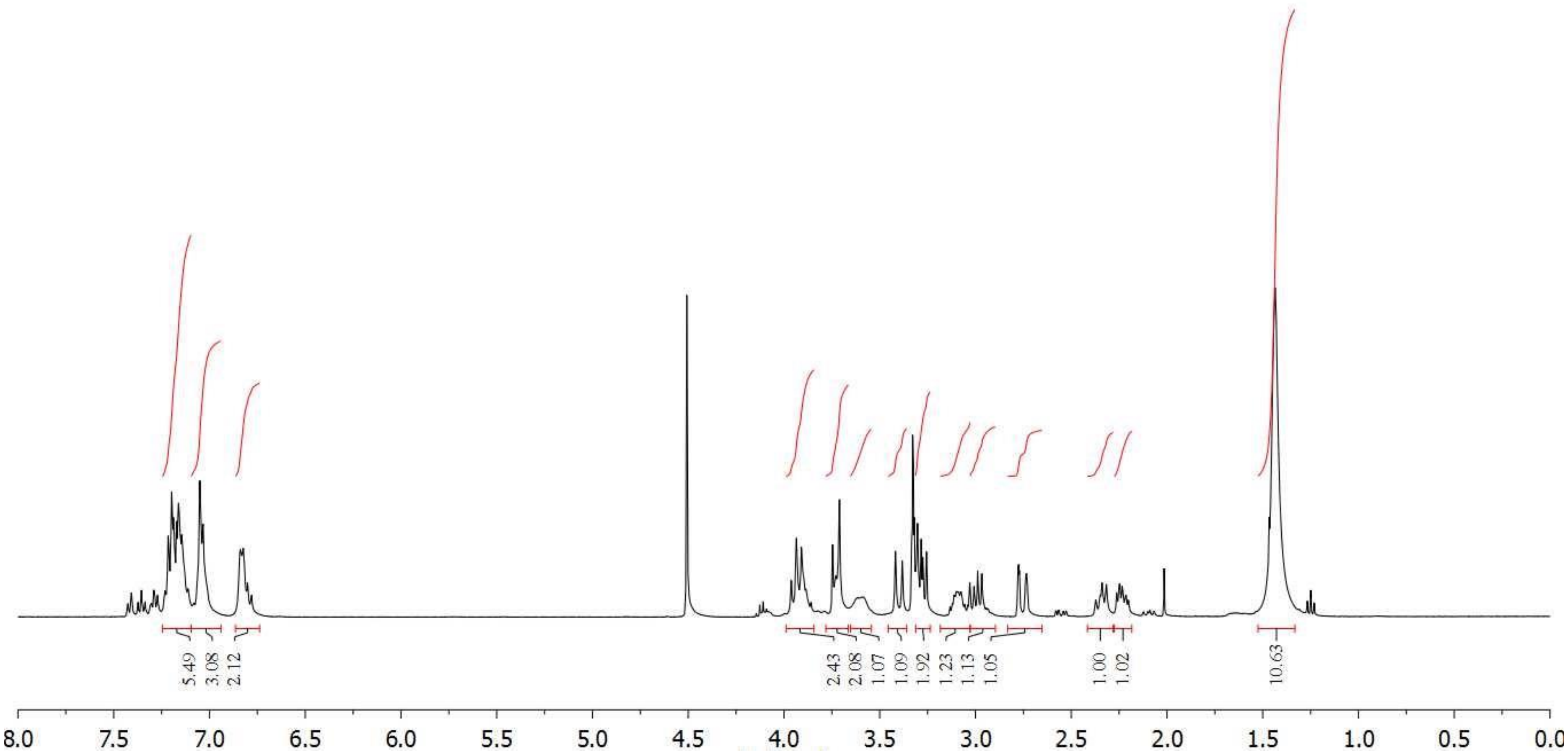

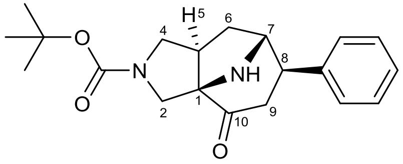

S30

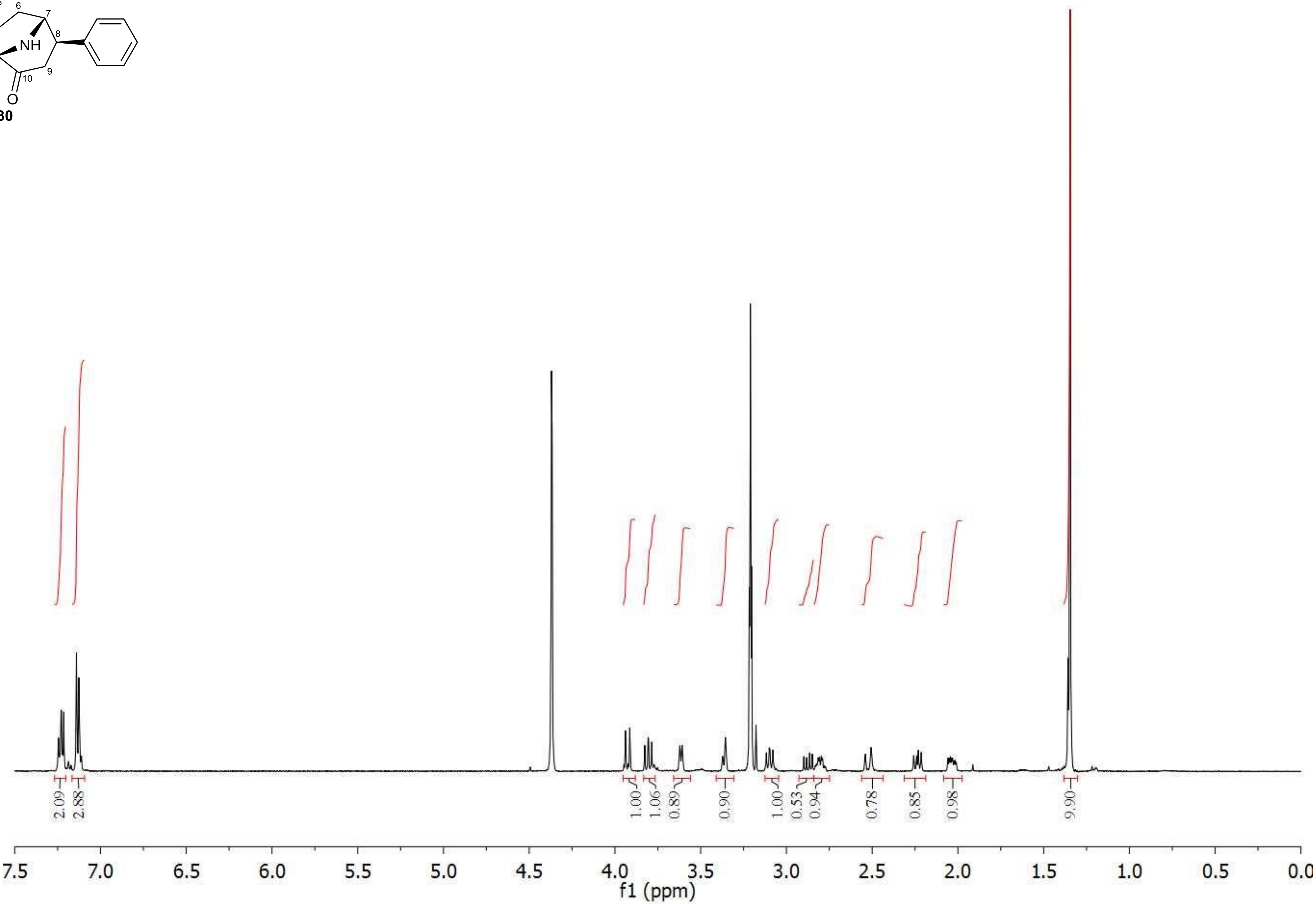
2.09
2.88
1.00
1.06
0.89
0.90
1.00
0.53
0.94
0.78
0.85
0.98
9.90
7.5
7.0
6.5
6.0
5.5
5.0
4.5
4.0
3.5
3.0
2.5
2.0
1.5
1.0
0.5
0.0
f1 (ppm)

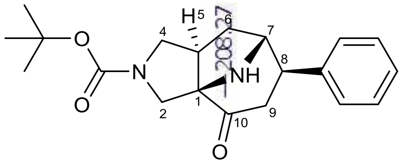

S30

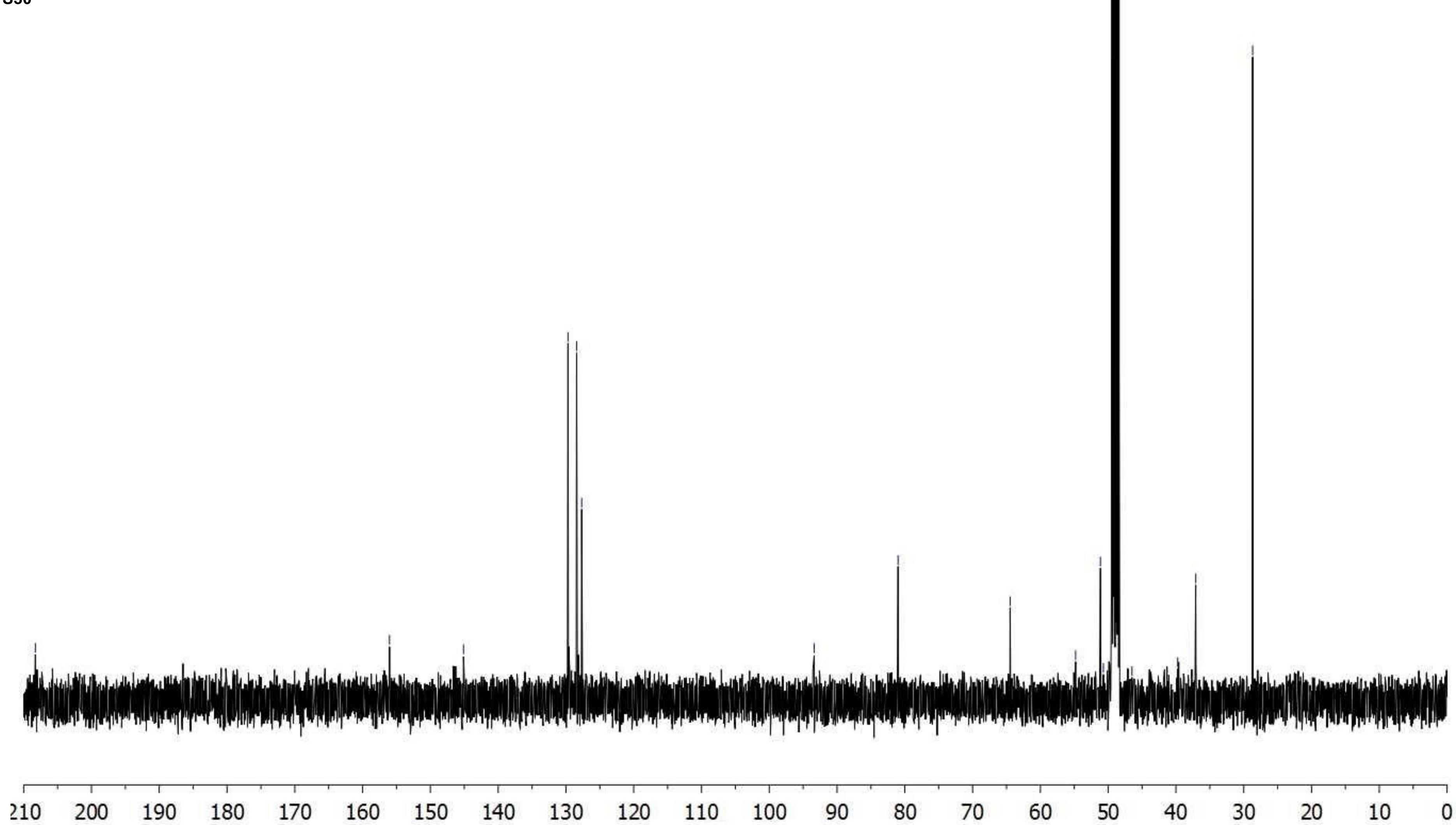

208.27
156.03
145.11
129.69
128.42
127.68
93.37
80.99
64.46
54.81
51.14
46.49
39.74
37.09
28.68
210
200
190
180
170
160
150
140
130
120
110
100
90
80
70
60
50
40
30
20
10
0

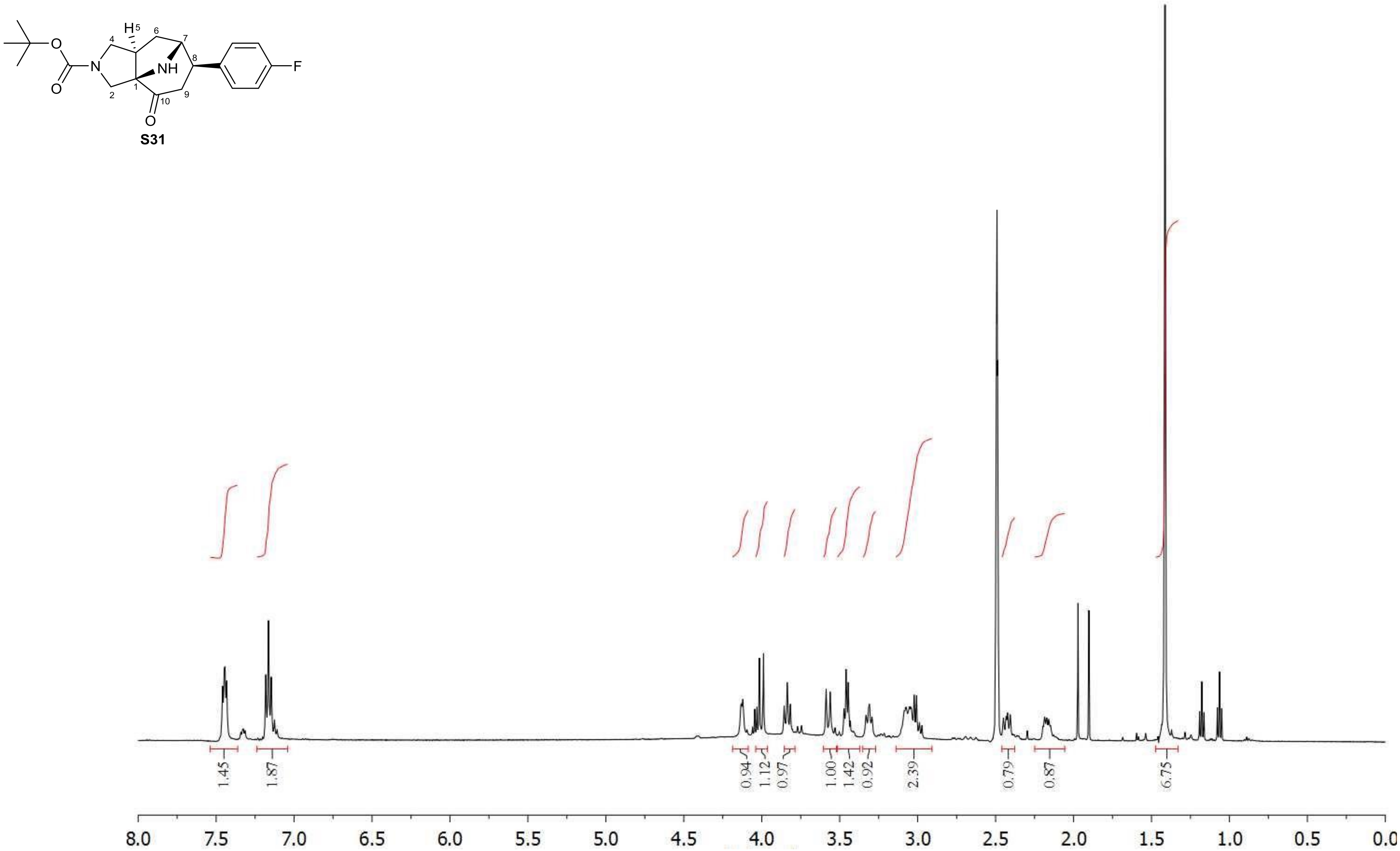

S31
1.45
1.87
0.94
1.12
0.97
1.00
1.42
0.92
2.39
0.79
0.87
6.75
8.0
7.5
7.0
6.5
6.0
5.5
5.0
4.5
4.0
3.5
3.0
2.5
2.0
1.5
1.0
0.5
0.0

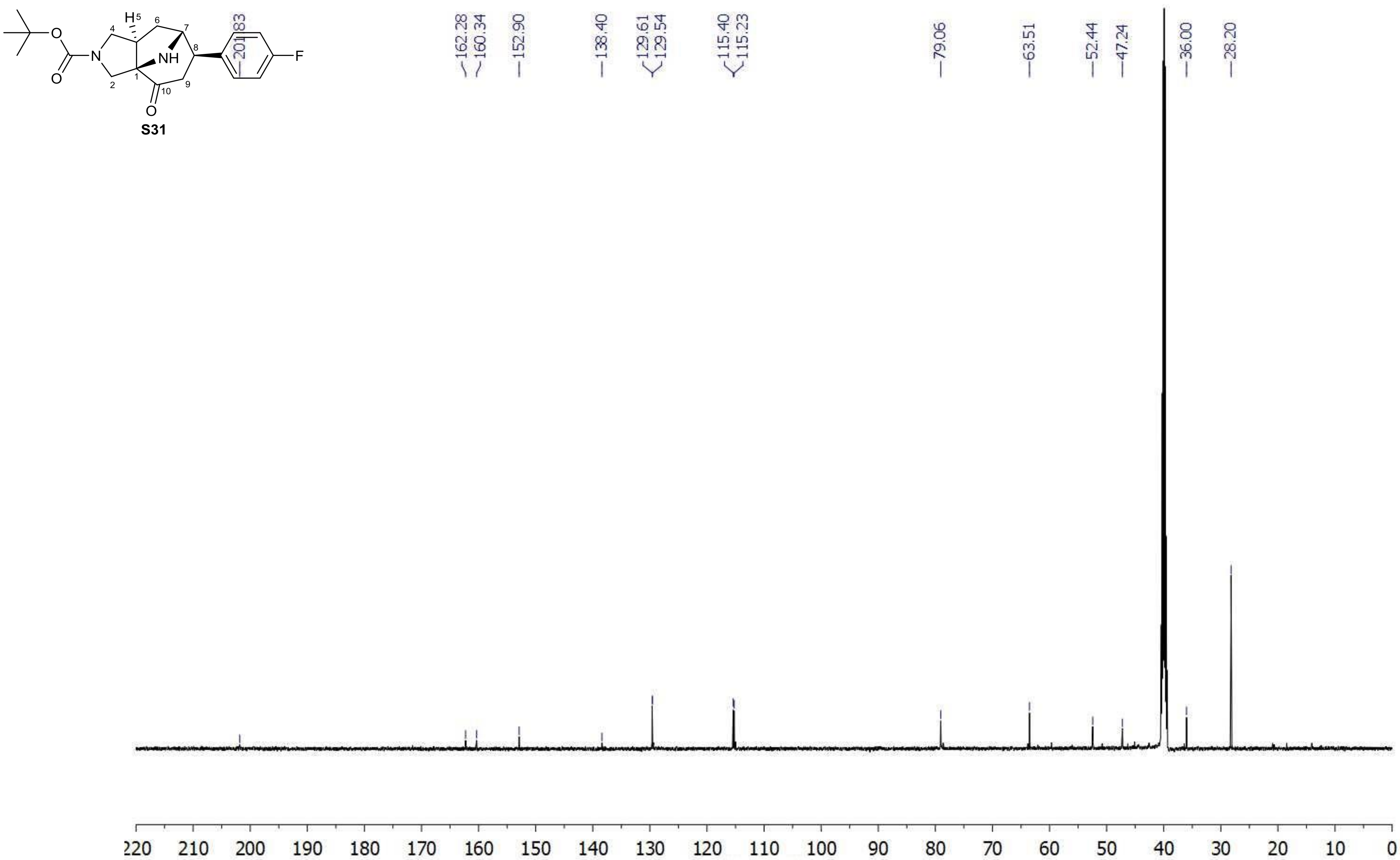
S31
201.83
162.28
160.34
152.90
138.40
129.61
129.54
115.40
115.23
79.06
63.51
52.44
47.24
36.00
28.20
220 210 200 190 180 170 160 150 140 130 120 110 100 90 80 70 60 50 40 30 20 10 0

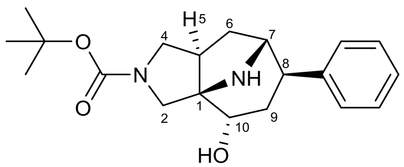

HO
NH
N
O
O

20

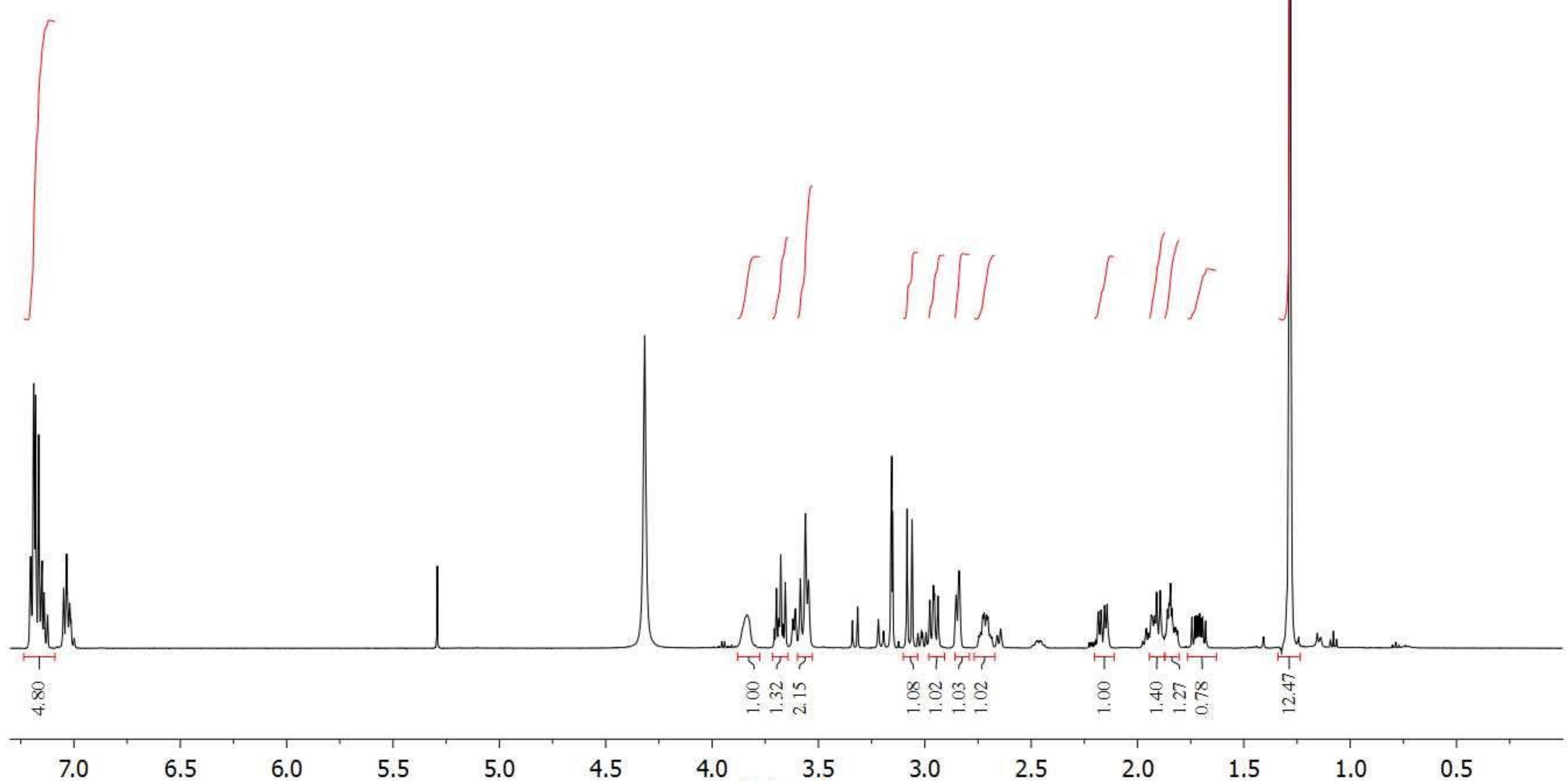

4.80
1.00
1.32
2.15
1.08
1.02
1.03
1.02
1.00
1.40
1.27
0.78
12.47
7.0
6.5
6.0
5.5
5.0
4.5
4.0
3.5
3.0
2.5
2.0
1.5
1.0
0.5

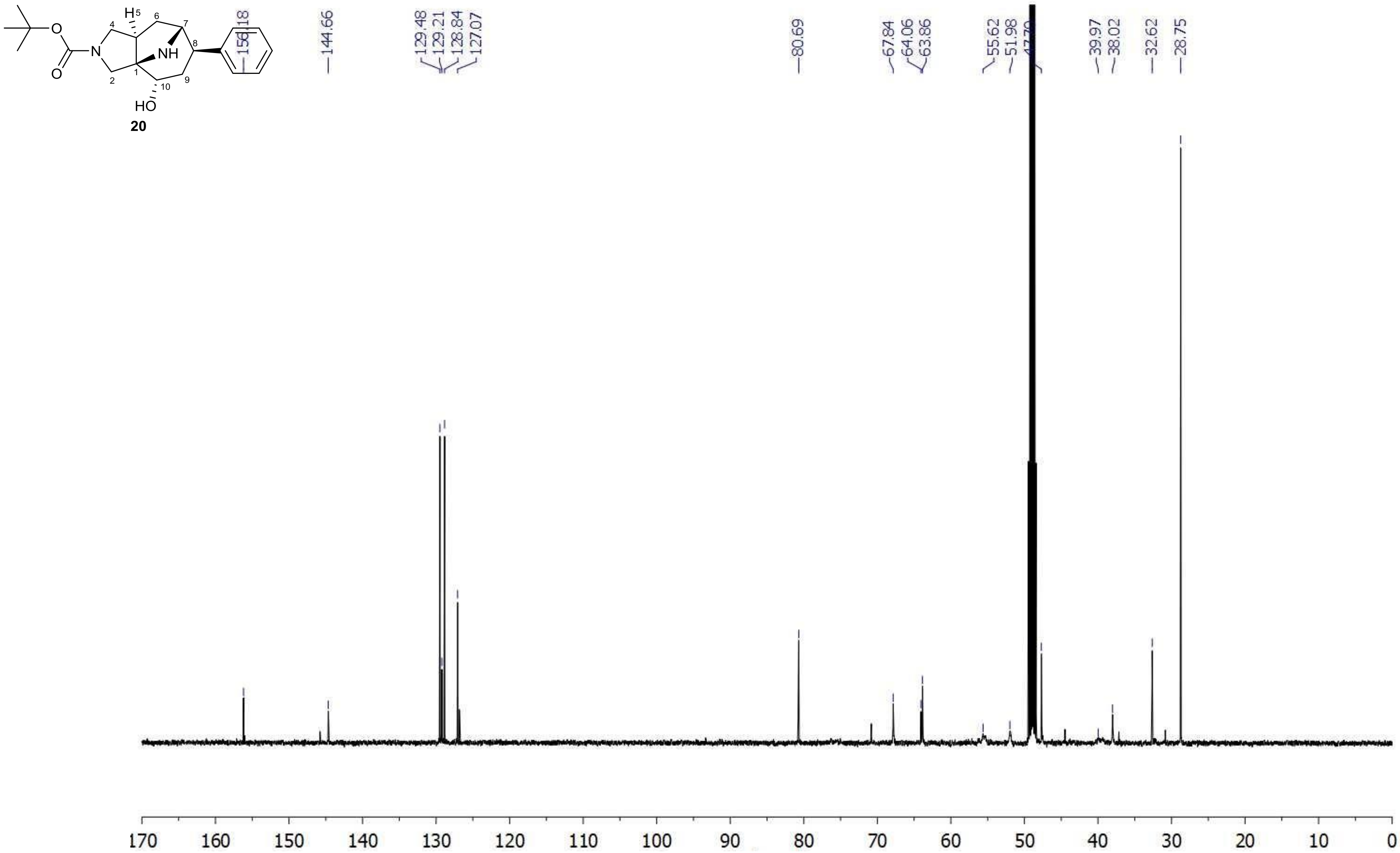
20
156.18
144.66
129.48
129.21
128.84
127.07
80.69
67.84
64.06
63.86
55.62
51.98
39.97
38.02
32.62
28.75
170 160 150 140 130 120 110 100 90 80 70 60 50 40 30 20 10 0

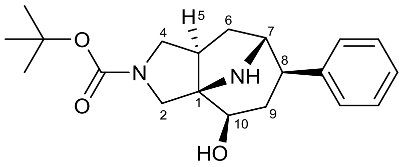

H
NH
HO
O
N
O
1
2
4
5
6
7
8
9
10

S32

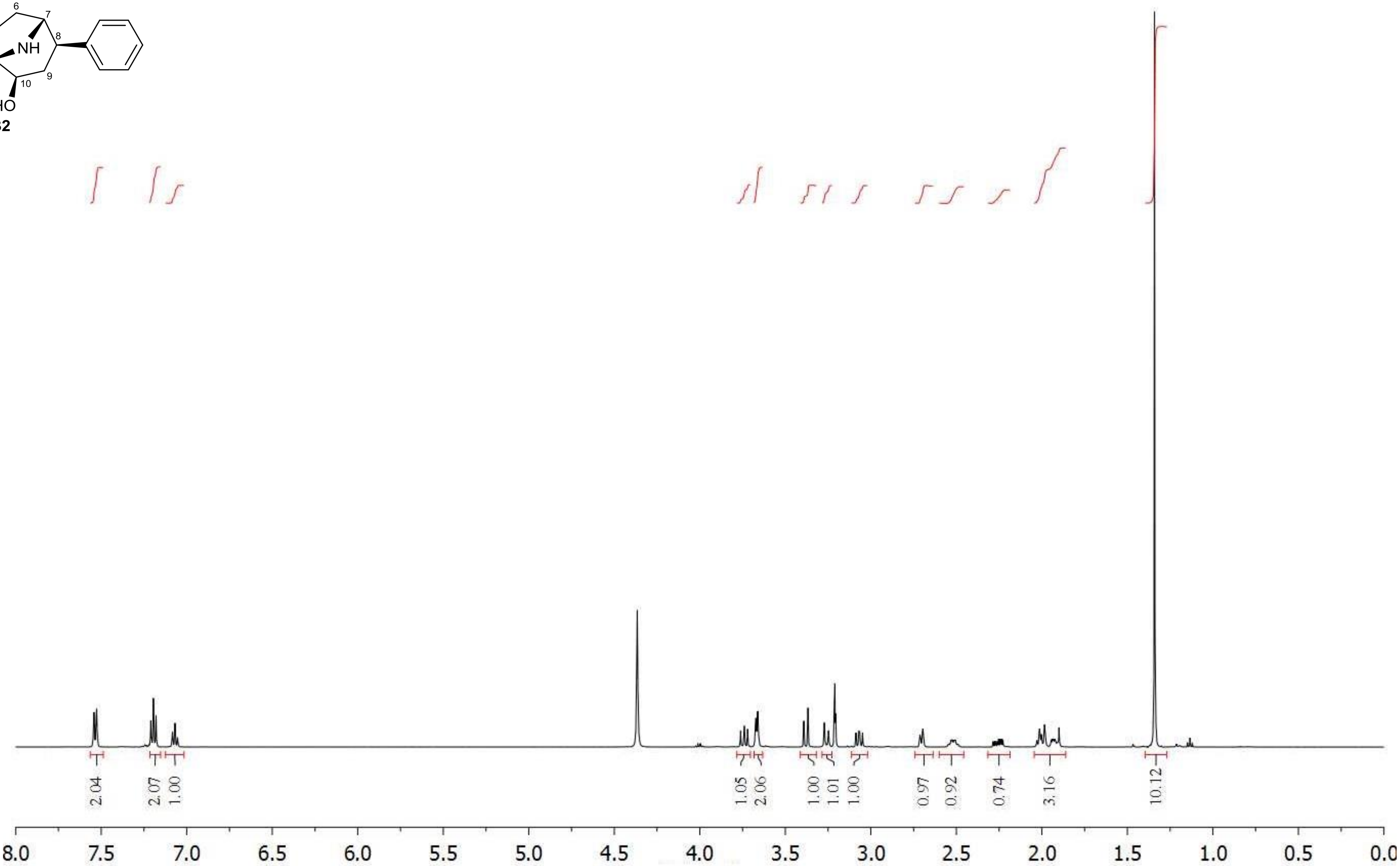

2.04
2.07
1.00
1.05
2.06
1.00
1.01
1.00
0.97
0.92
0.74
3.16
10.12
8.0
7.5
7.0
6.5
6.0
5.5
5.0
4.5
4.0
3.5
3.0
2.5
2.0
1.5
1.0
0.5
0.0

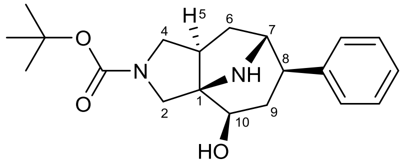
H5
6
7
8
4
O
N
NH
O
1
2
10
9
HO

S32

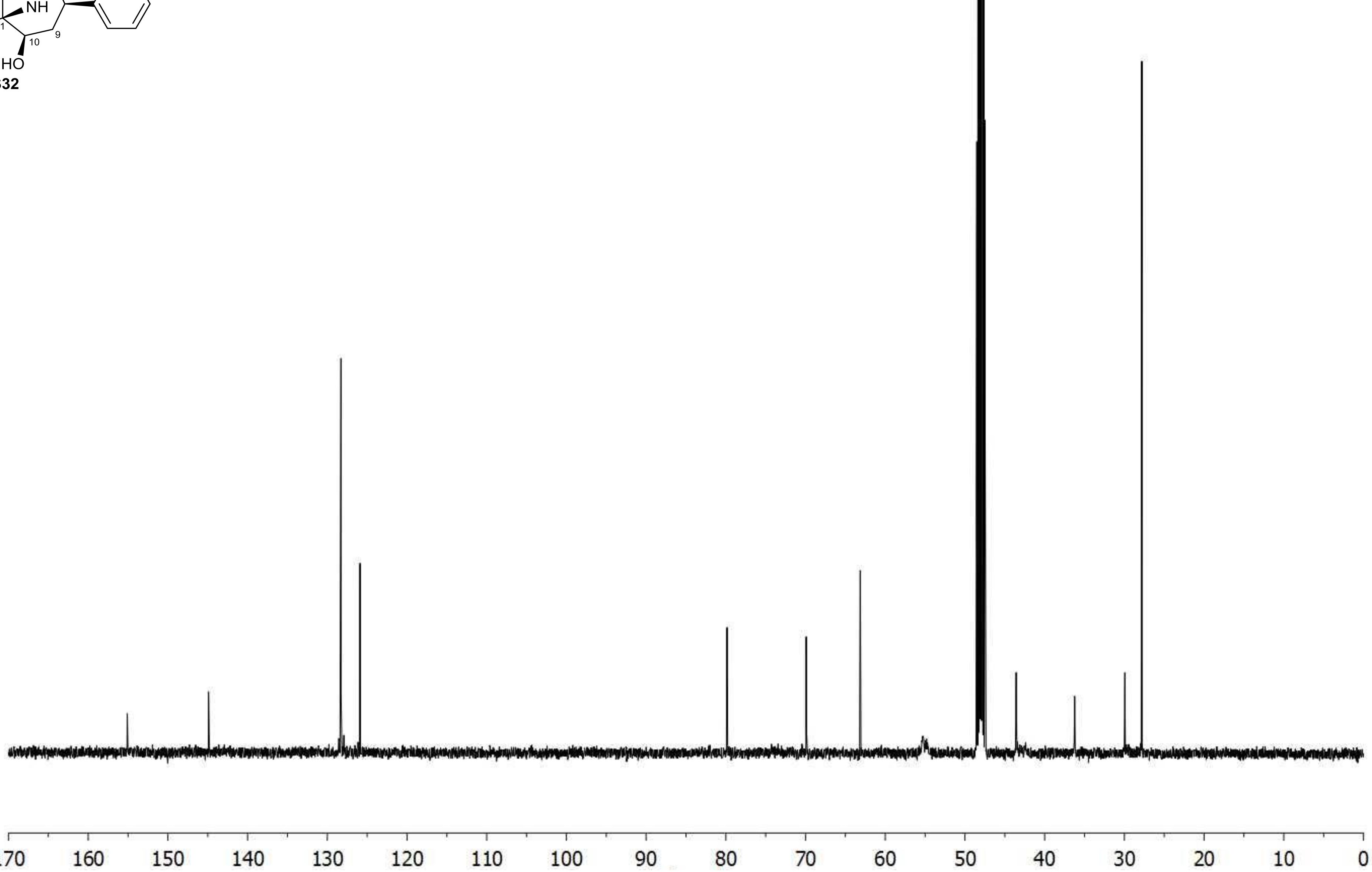
170 160 150 140 130 120 110 100 90 80 70 60 50 40 30 20 10 0

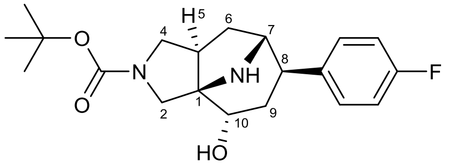

S33

+

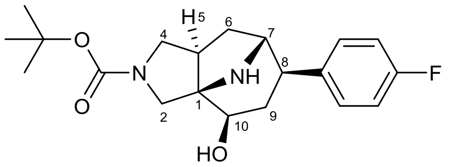

S34

S33:S34 40:60

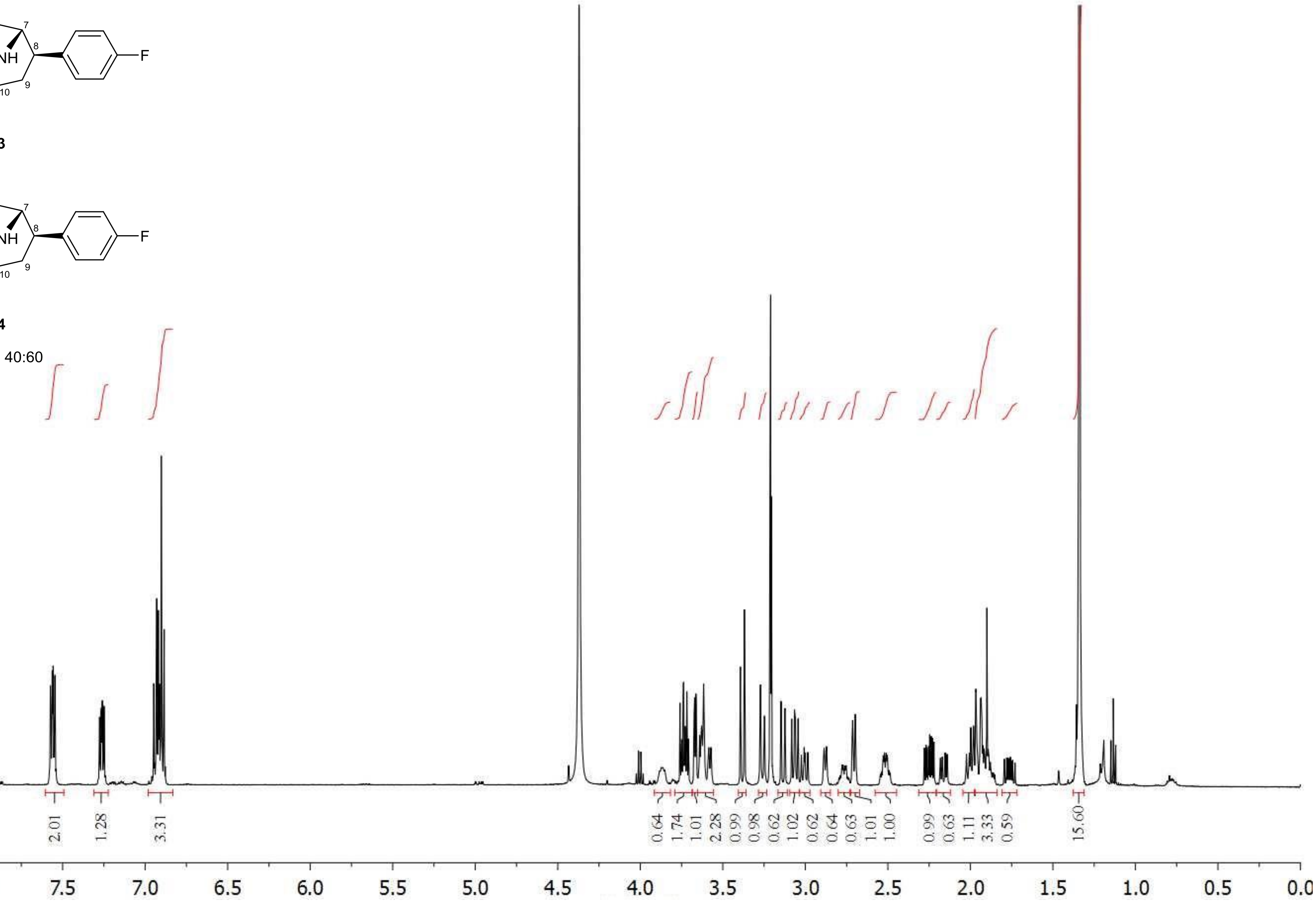

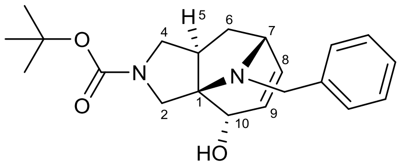
H
5
6
7
4
8
O
N
N
O
1
2
9
10
HO

S35

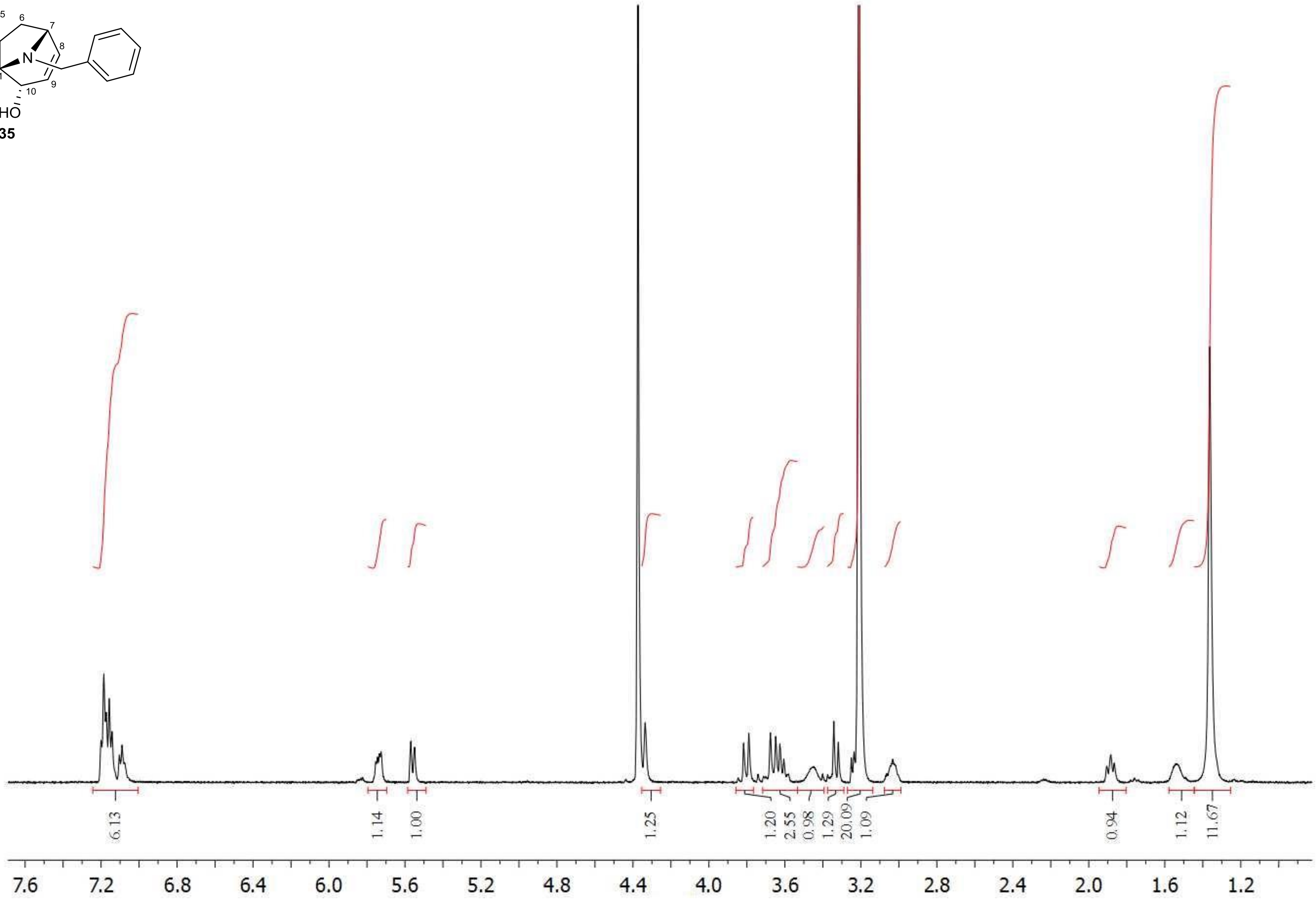
6.13
1.14
1.00
1.25
1.20
2.55
0.98
1.29
20.09
1.09
0.94
1.12
11.67
7.6
7.2
6.8
6.4
6.0
5.6
5.2
4.8
4.4
4.0
3.6
3.2
2.8
2.4
2.0
1.6
1.2

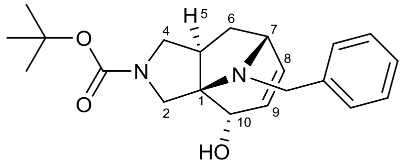

S35

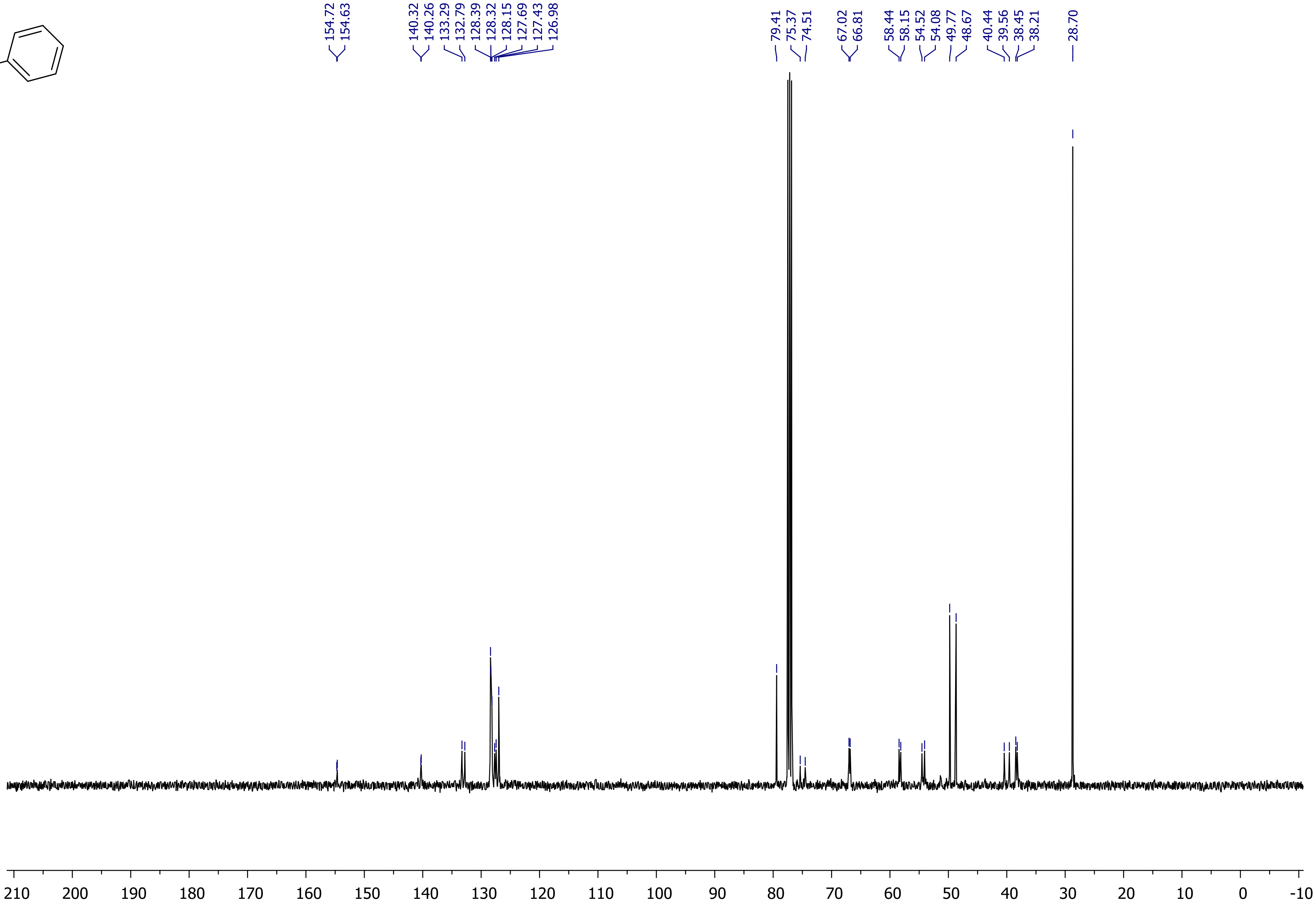
154.72
154.63
140.32
140.26
133.29
132.79
128.39
128.32
128.15
127.69
127.43
126.98
79.41
75.37
74.51
67.02
66.81
58.44
58.15
54.52
54.08
49.77
48.67
40.44
39.56
38.45
38.21
28.70
210 200 190 180 170 160 150 140 130 120 110 100 90 80 70 60 50 40 30 20 10 0 -10

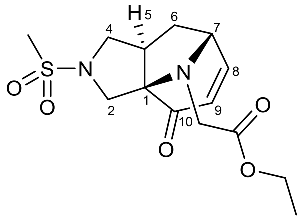

S36
(MeOD-d_4 @ 333 K)

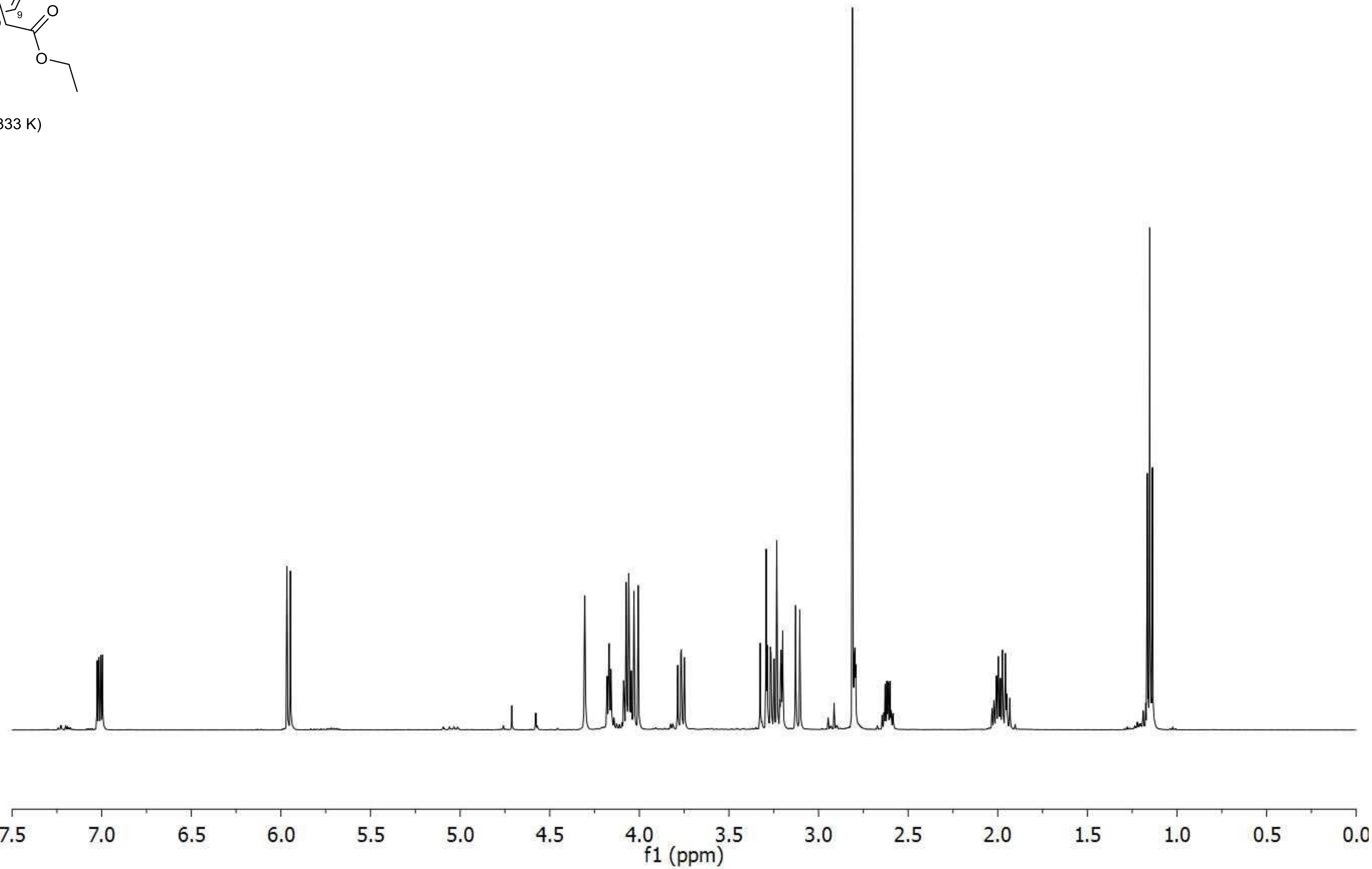

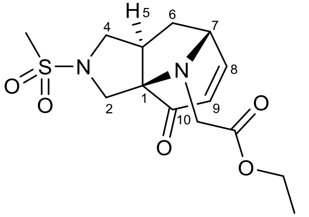

S36
(MeOD-d_4 @ 333 K)

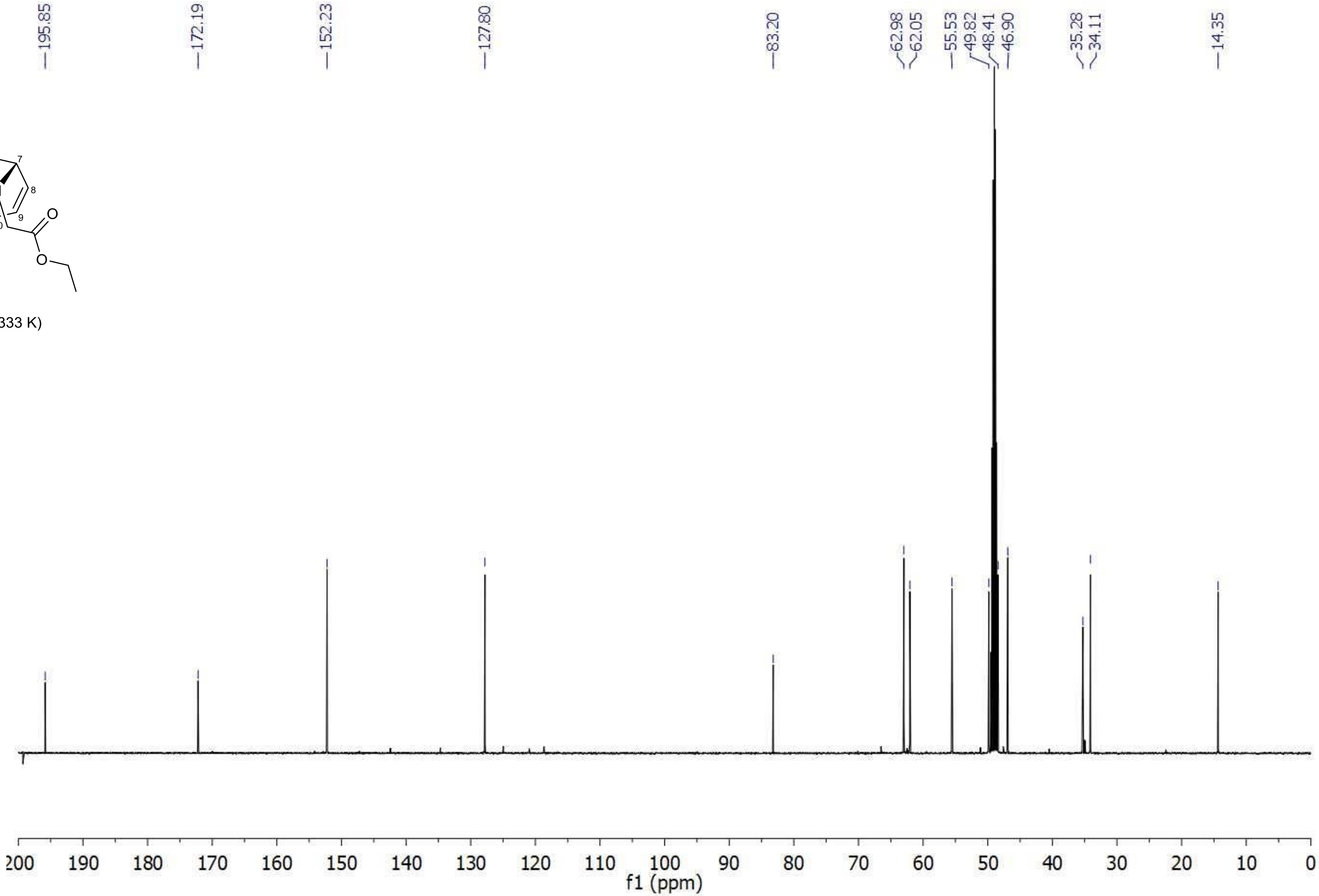

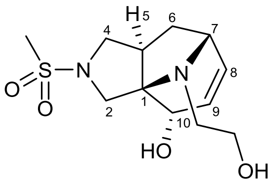

F1

+

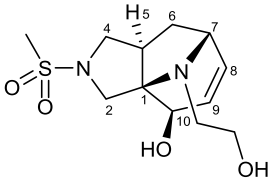

F2

(MeOD-d_4 @ 333 K)

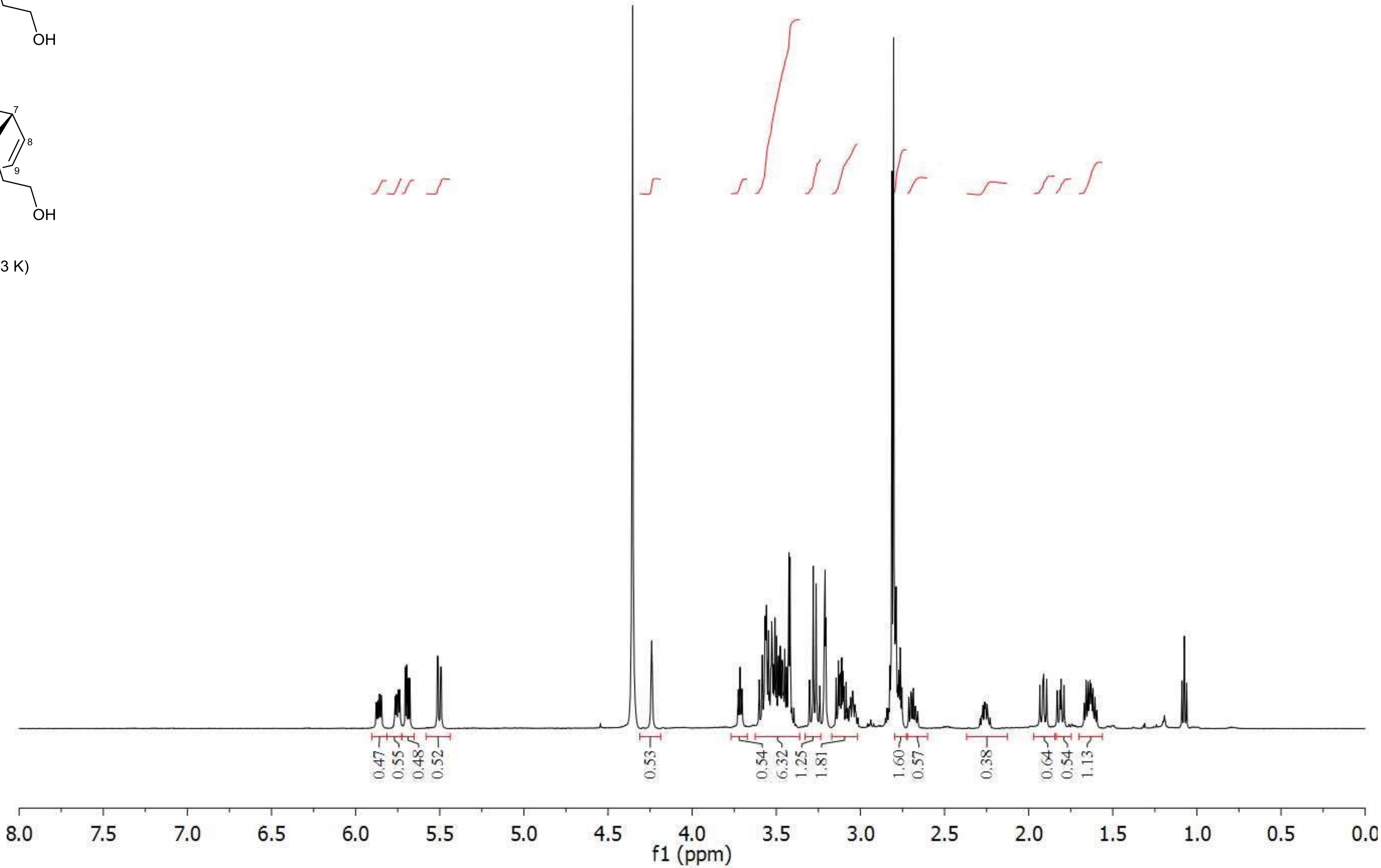

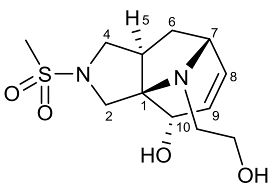

F1

+

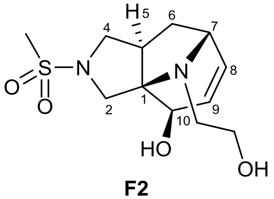

F2

(MeOD-d_4 @ 333 K)

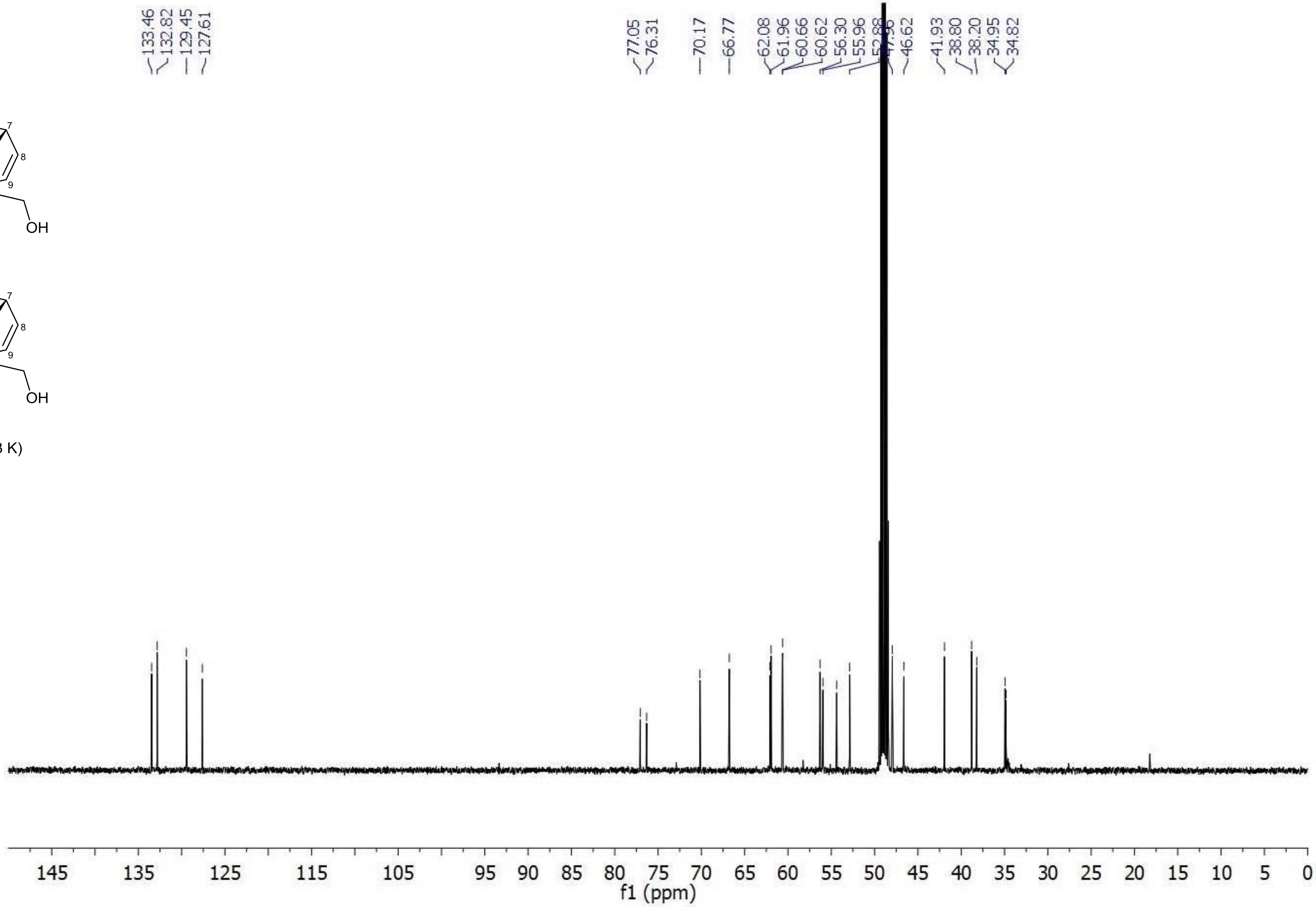

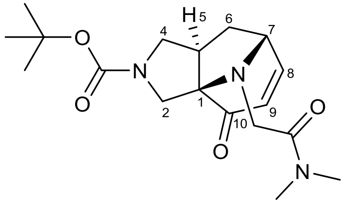

S37
(MeOD-d_4 @ 333 K)

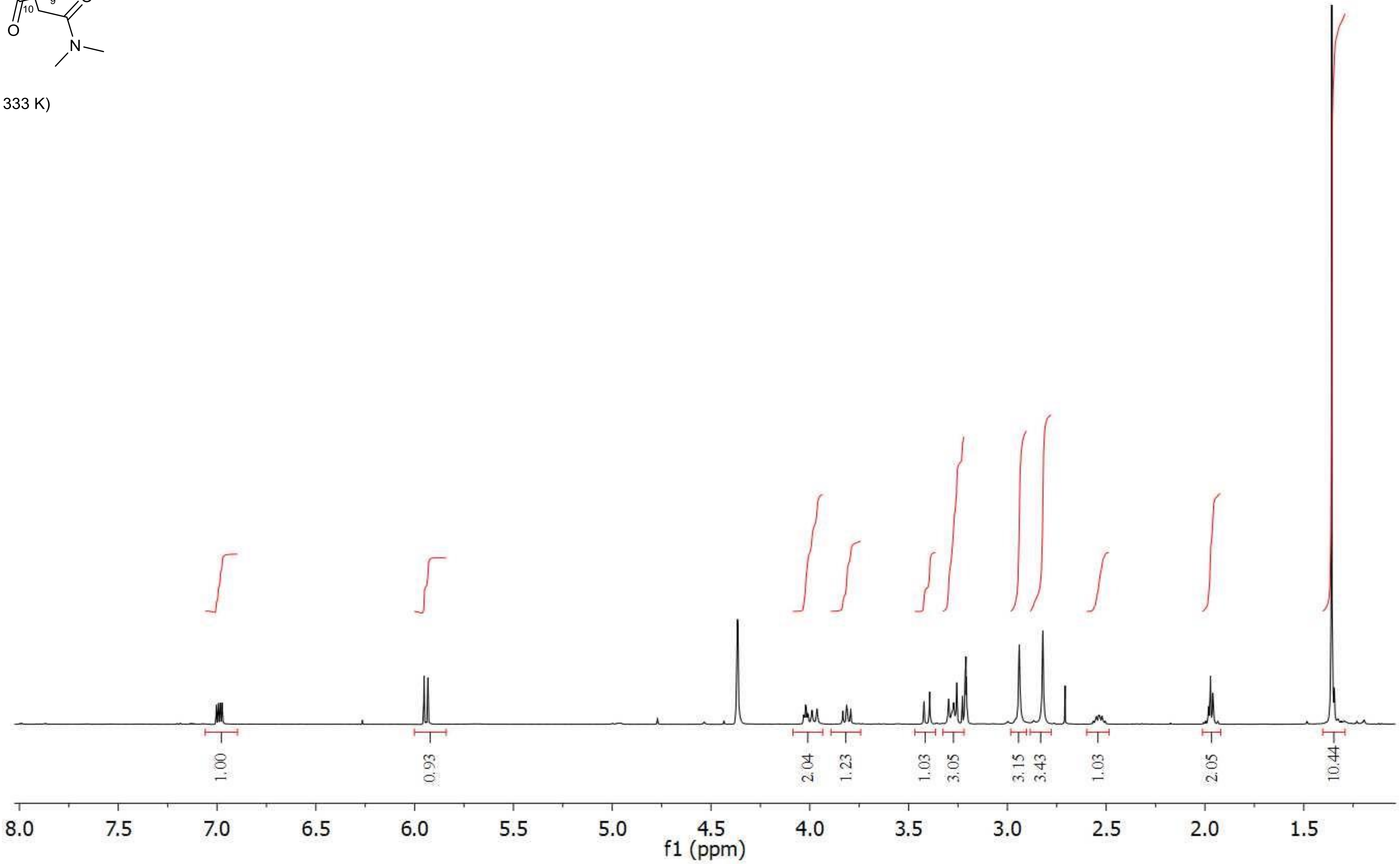

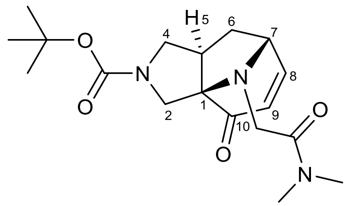

S37
(MeOD-d_4 @ 333 K)

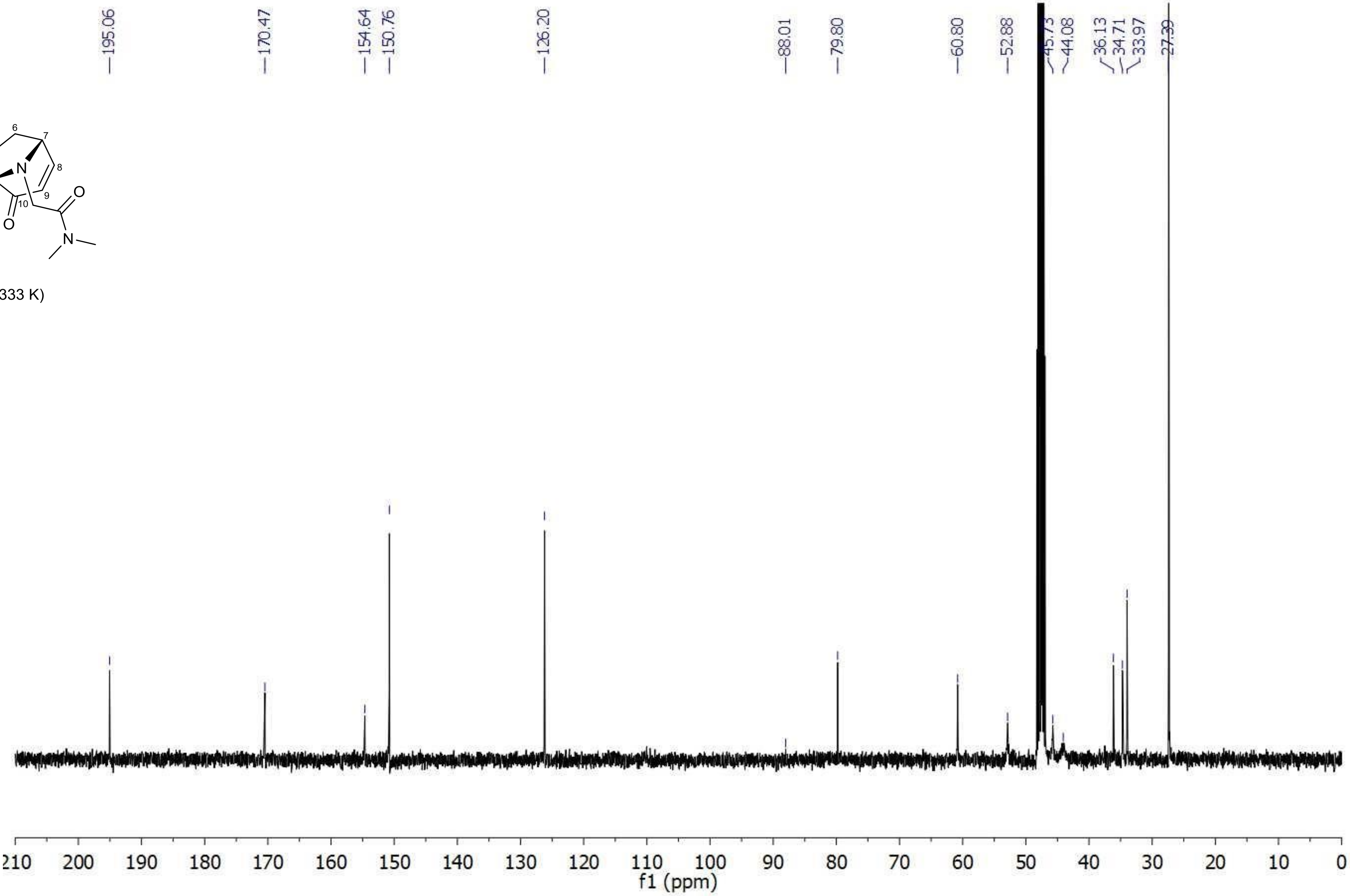

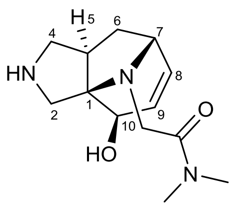

F3
(MeOD-d_4 @ 333 K)

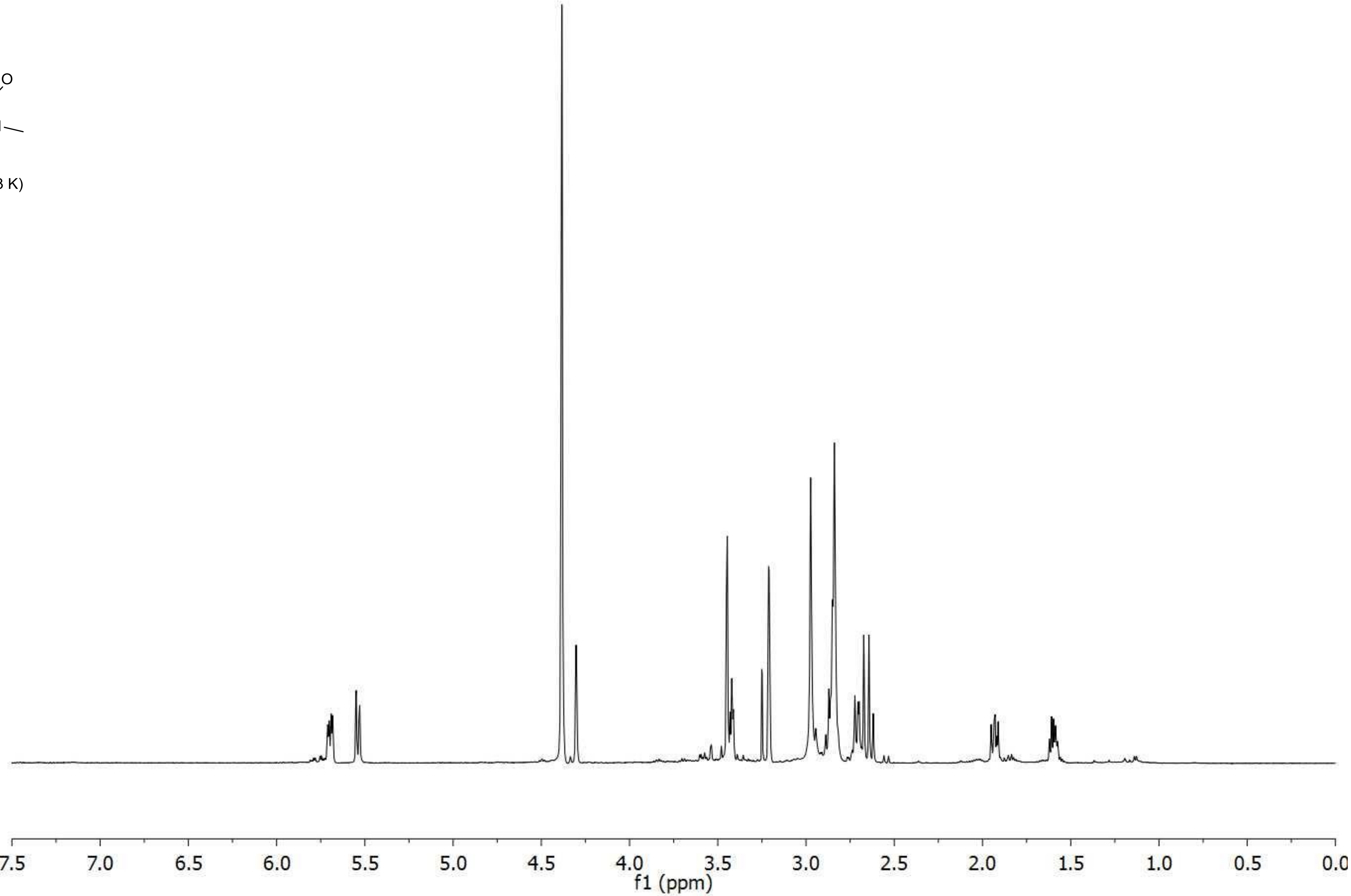

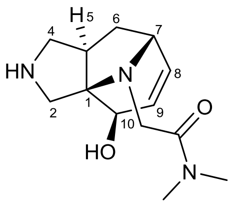

F3
(MeOD-d_4 @ 333 K)

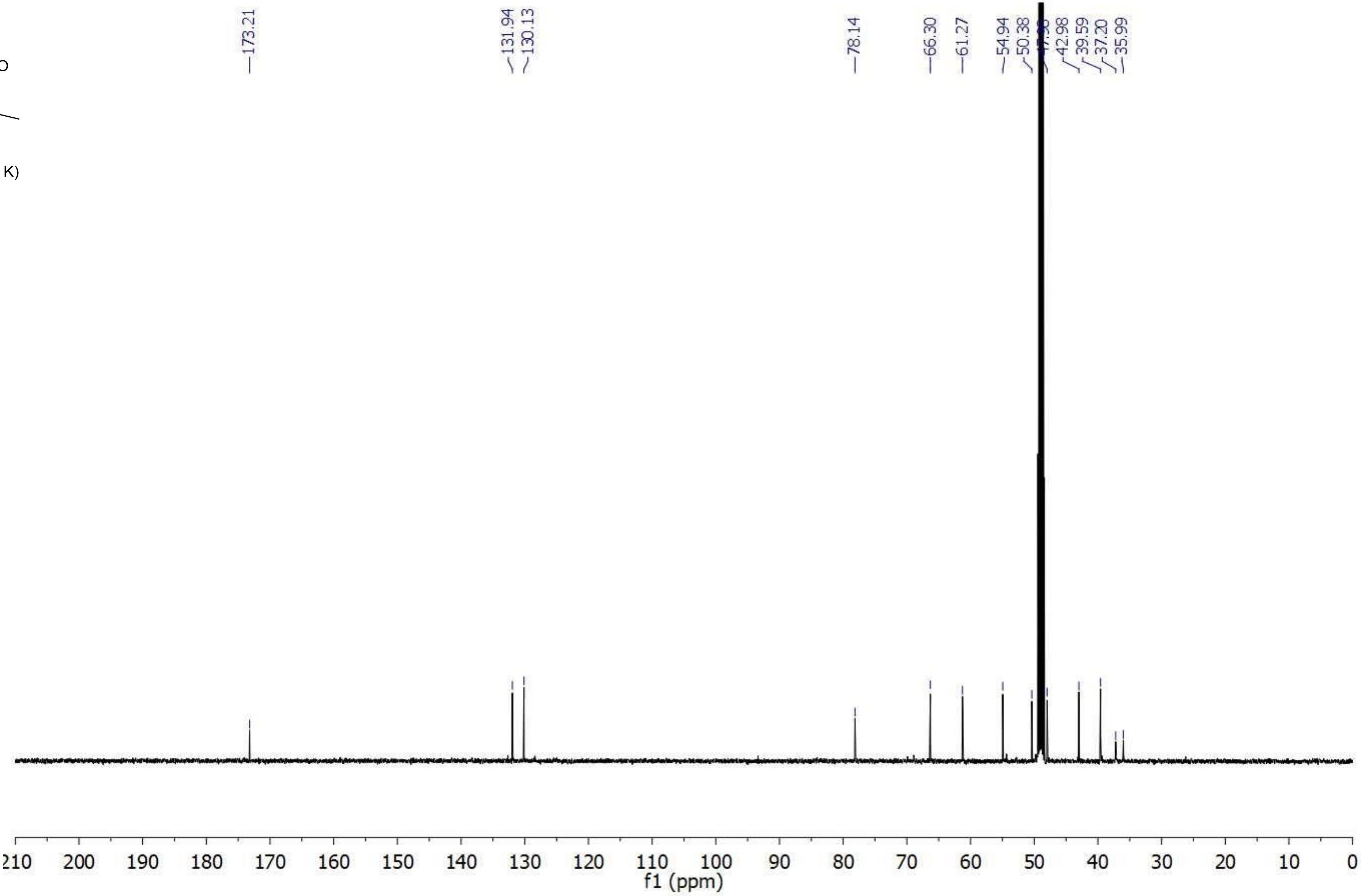

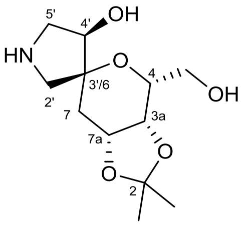

F4

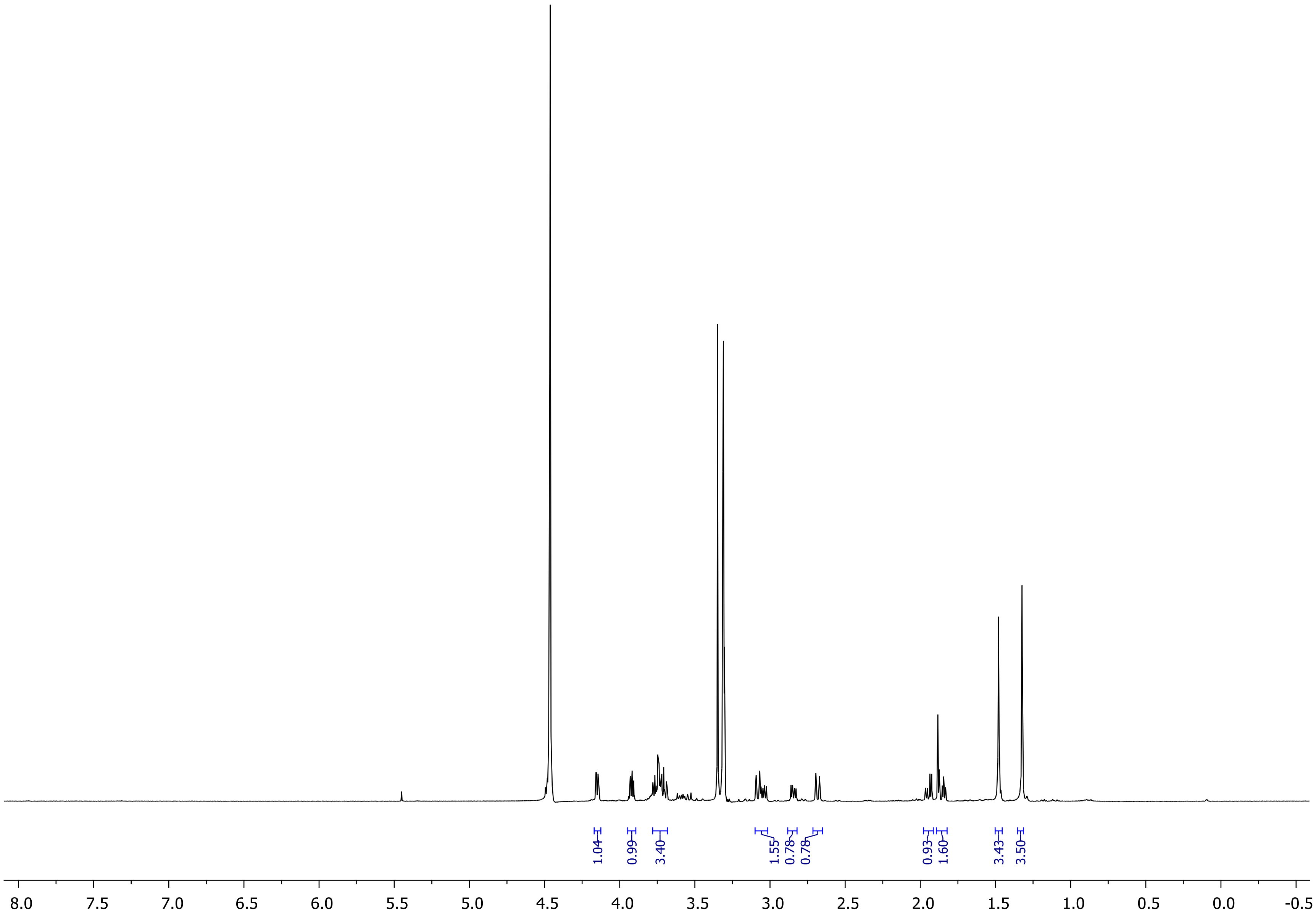

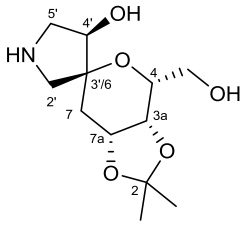
OH
5'
4'
HN
O
4
3'/6
OH
2'
7
3a
7a
O
O
2

F4

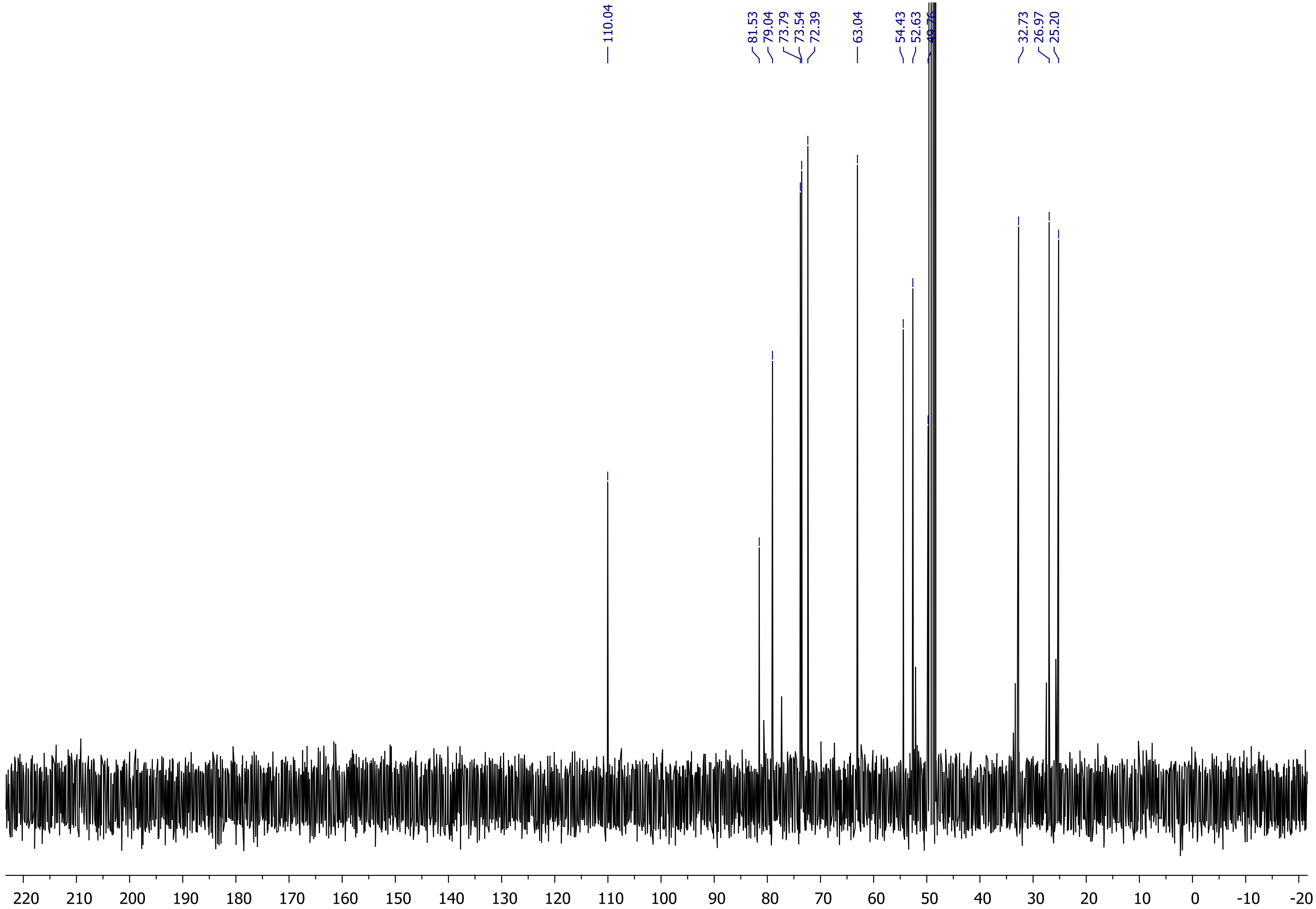
110.04
81.53
79.04
73.79
73.54
72.39
63.04
54.43
52.63
49.76
32.73
26.97
25.20
220 210 200 190 180 170 160 150 140 130 120 110 100 90 80 70 60 50 40 30 20 10 0 -10 -20

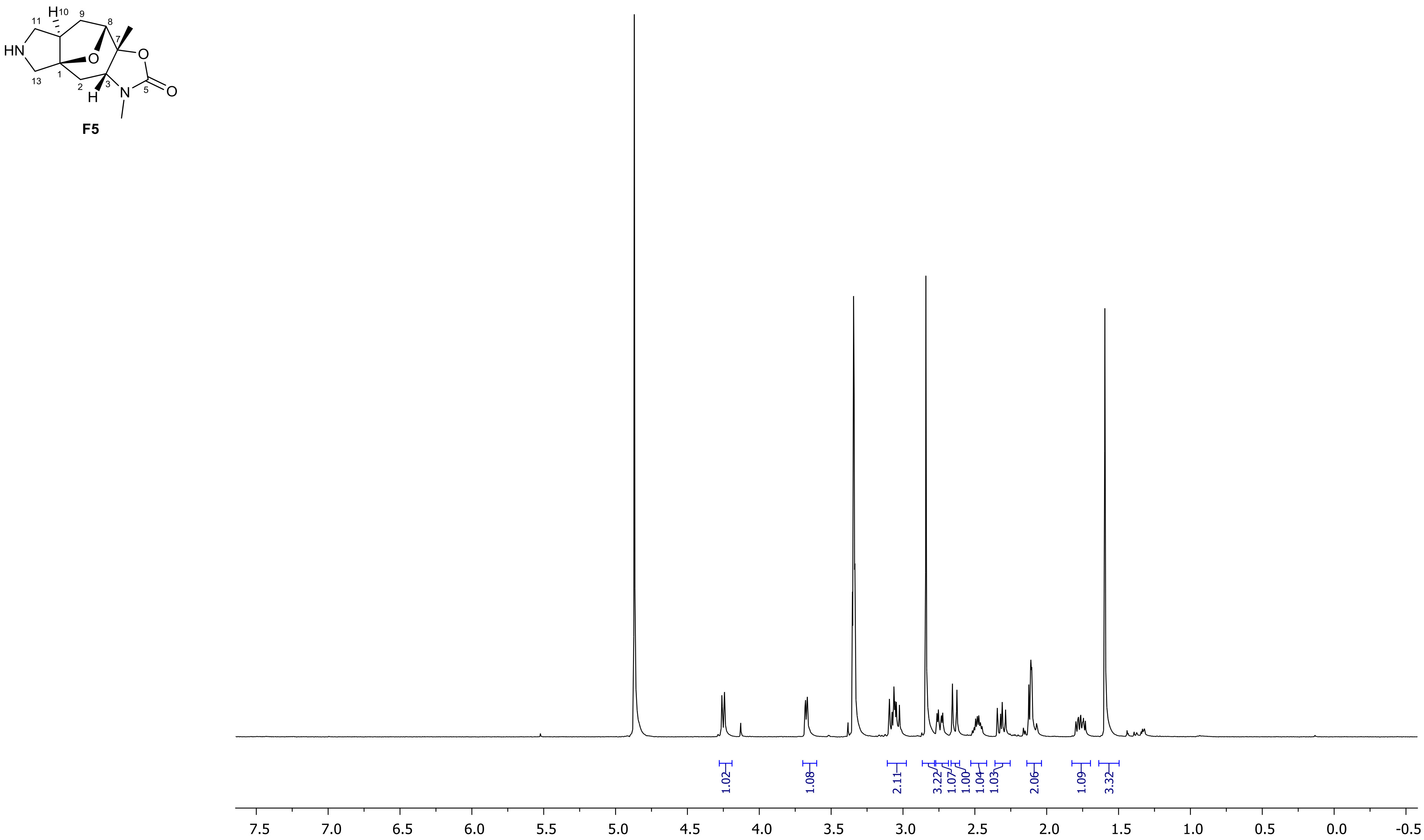
H
10
9
8
7
11
HN
O
O
13
1
2
3
5
O
N
H
1.02
1.08
2.11
3.22
1.07
1.00
1.04
1.03
2.06
1.09
3.32
7.5 7.0 6.5 6.0 5.5 5.0 4.5 4.0 3.5 3.0 2.5 2.0 1.5 1.0 0.5 0.0 -0.5

F5

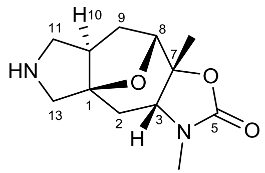
H
10
9
8
11
HN
O
7
O
13
1
2
3
5
O
H
N

F5

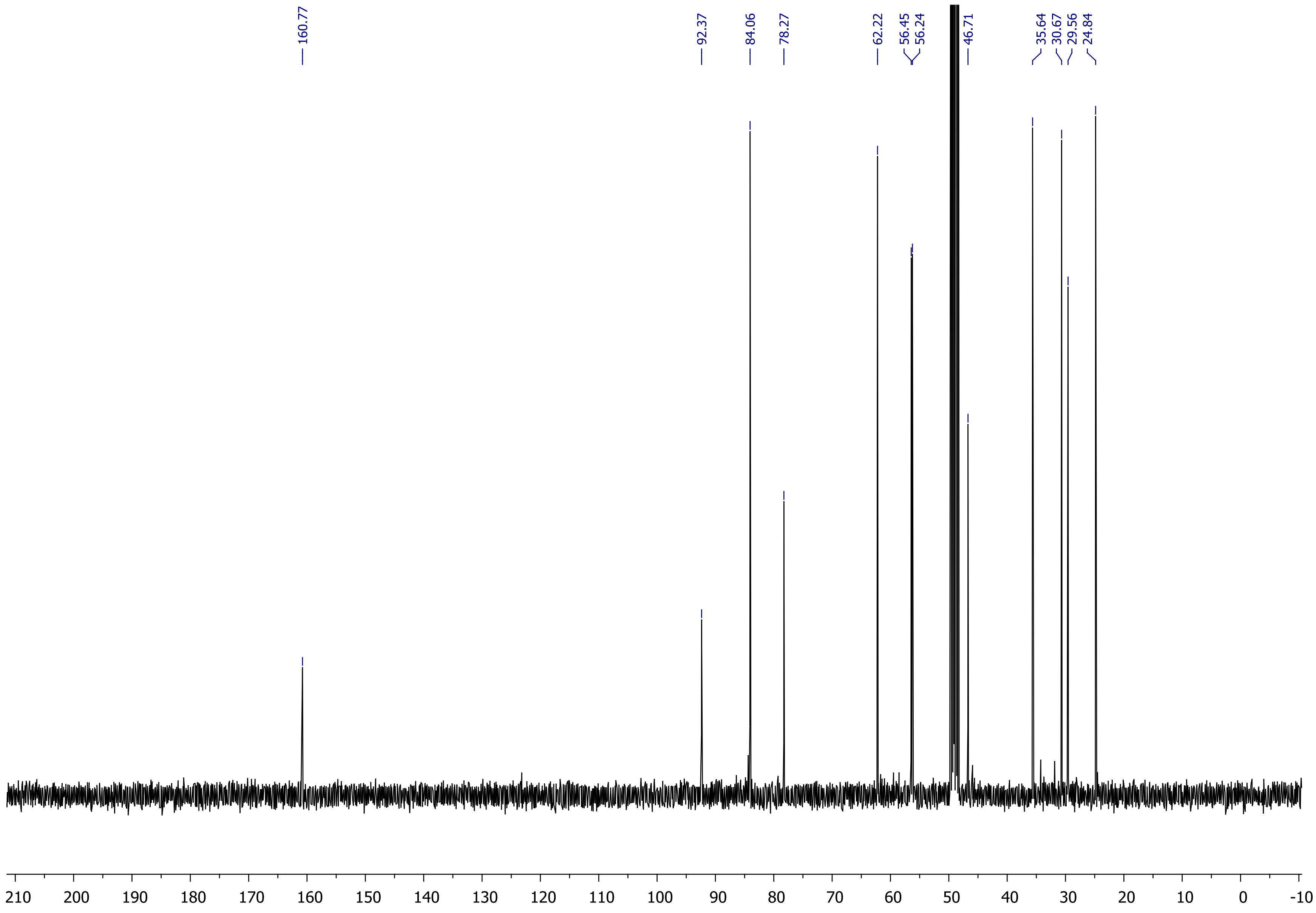
160.77
92.37
84.06
78.27
62.22
56.45
56.24
46.71
35.64
30.67
29.56
24.84
210
200
190
180
170
160
150
140
130
120
110
100
90
80
70
60
50
40
30
20
10
0
-10

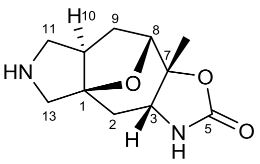

H
10
9
8
11
7
HN
O
O
1
3
13
2
H
N
H
5
O

F6

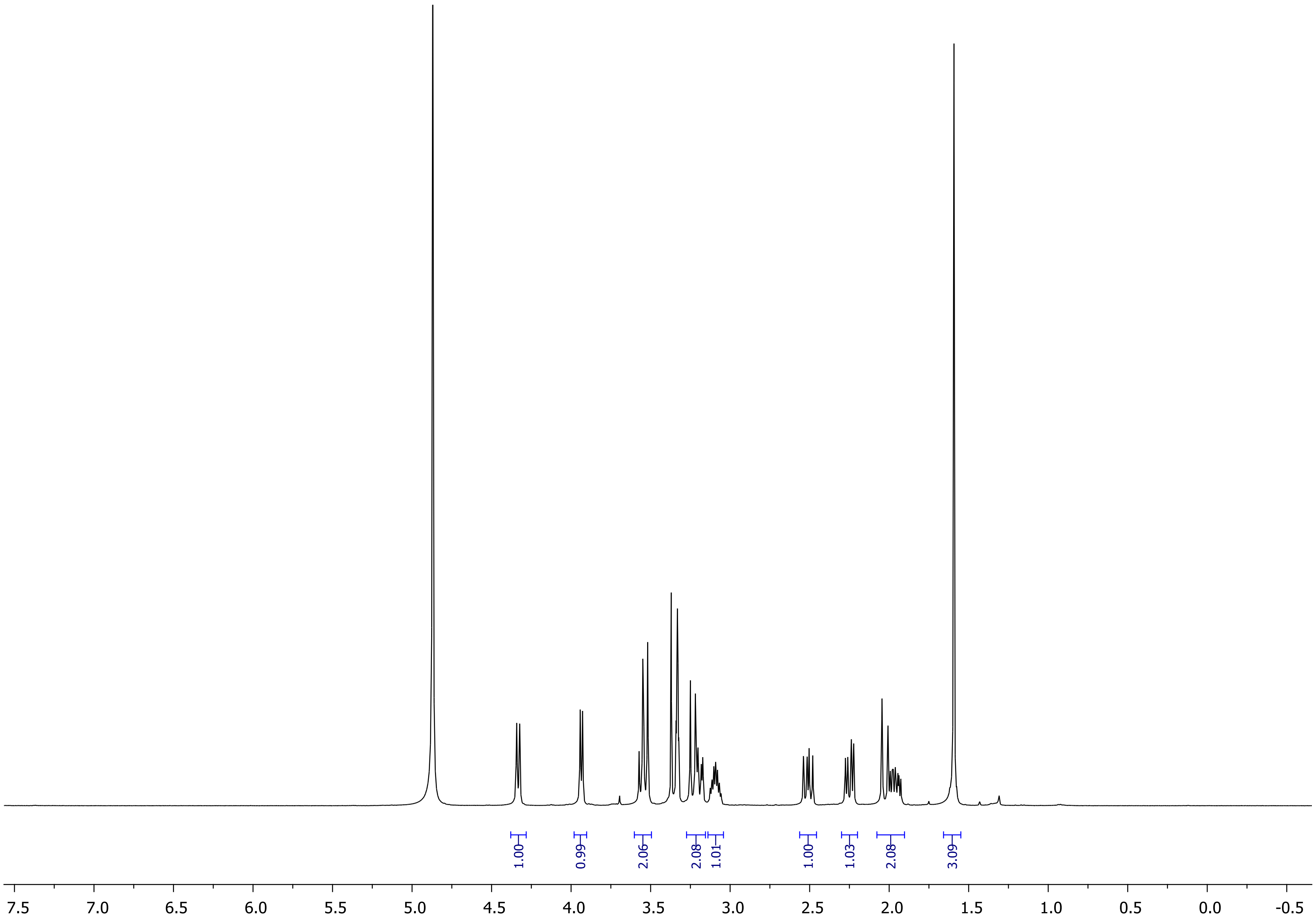

1.00
0.99
2.06
2.08
1.01
1.00
1.03
2.08
3.09
7.5
7.0
6.5
6.0
5.5
5.0
4.5
4.0
3.5
3.0
2.5
2.0
1.5
1.0
0.5
0.0
-0.5

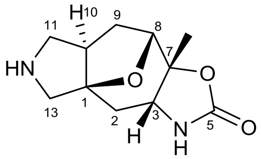
H
10
9
8
11
7
HN
O
O
1
13
2
3
5
O
H
N
H

F6

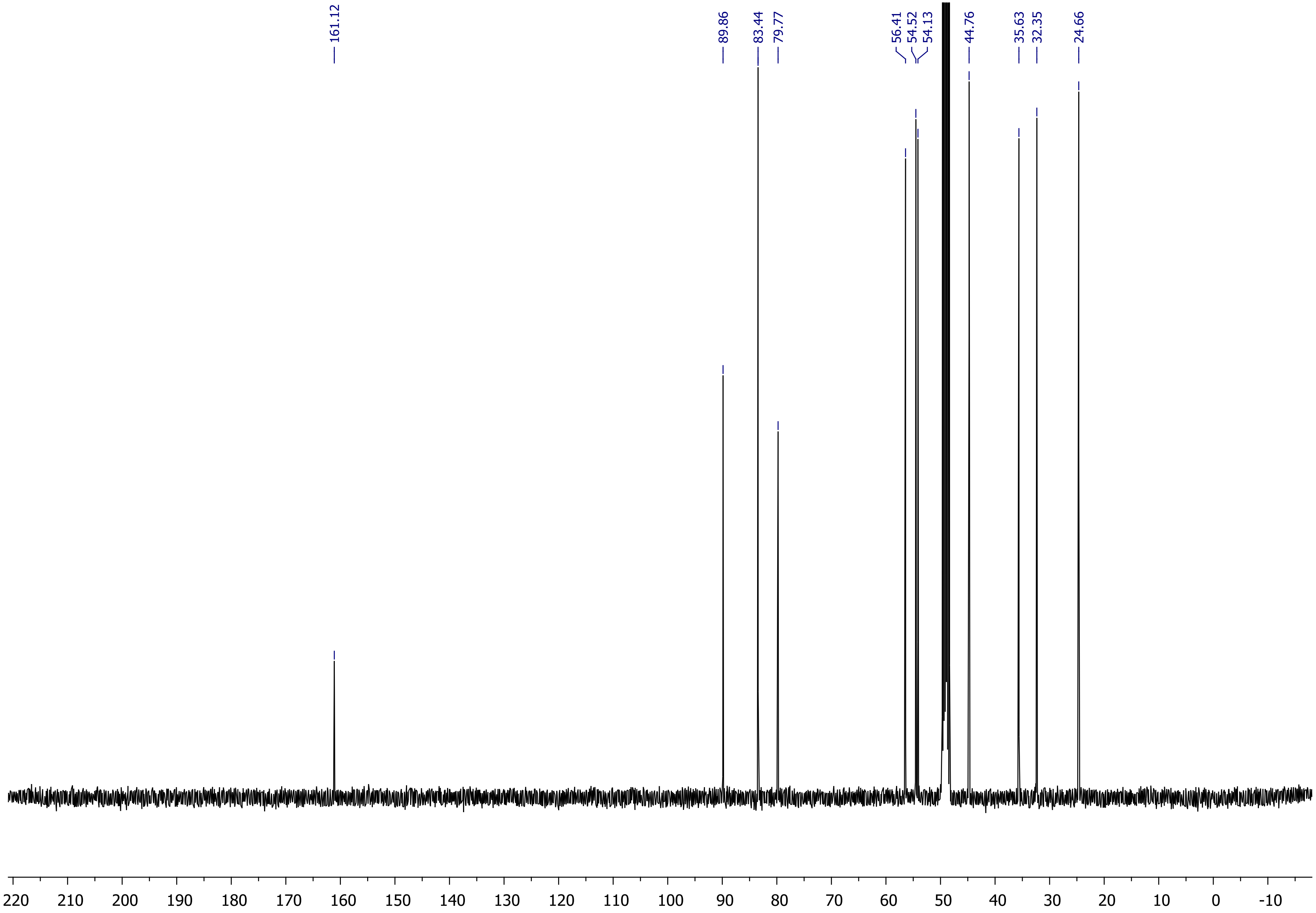
161.12
89.86
83.44
79.77
56.41
54.52
54.13
44.76
35.63
32.35
24.66
220 210 200 190 180 170 160 150 140 130 120 110 100 90 80 70 60 50 40 30 20 10 0 -10

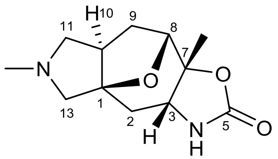

F7

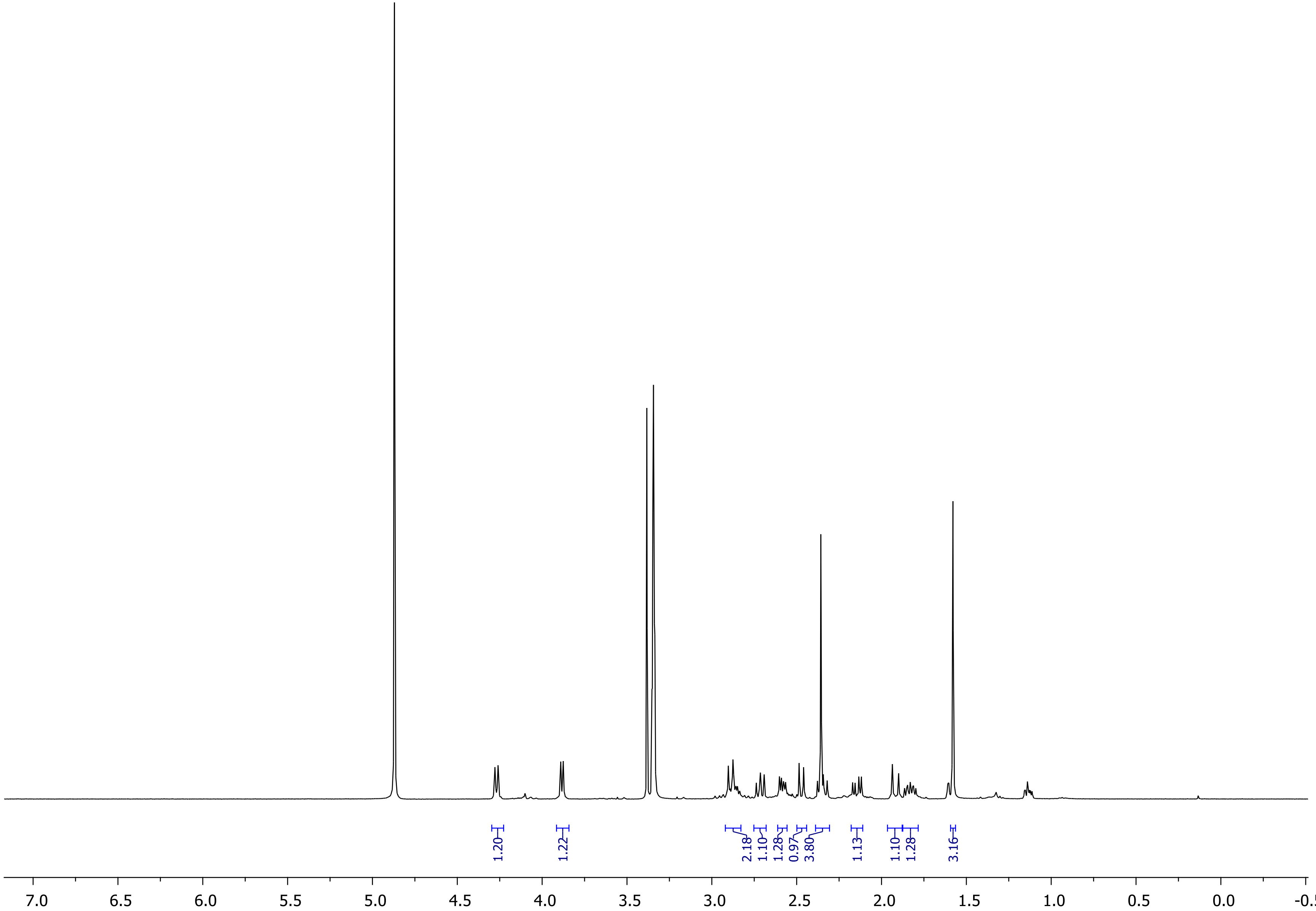
1.20
1.22
2.18
1.10
1.28
0.97
3.80
1.13
1.10
1.28
3.16
7.0 6.5 6.0 5.5 5.0 4.5 4.0 3.5 3.0 2.5 2.0 1.5 1.0 0.5 0.0 -0.5

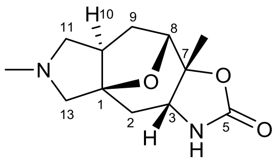

F7

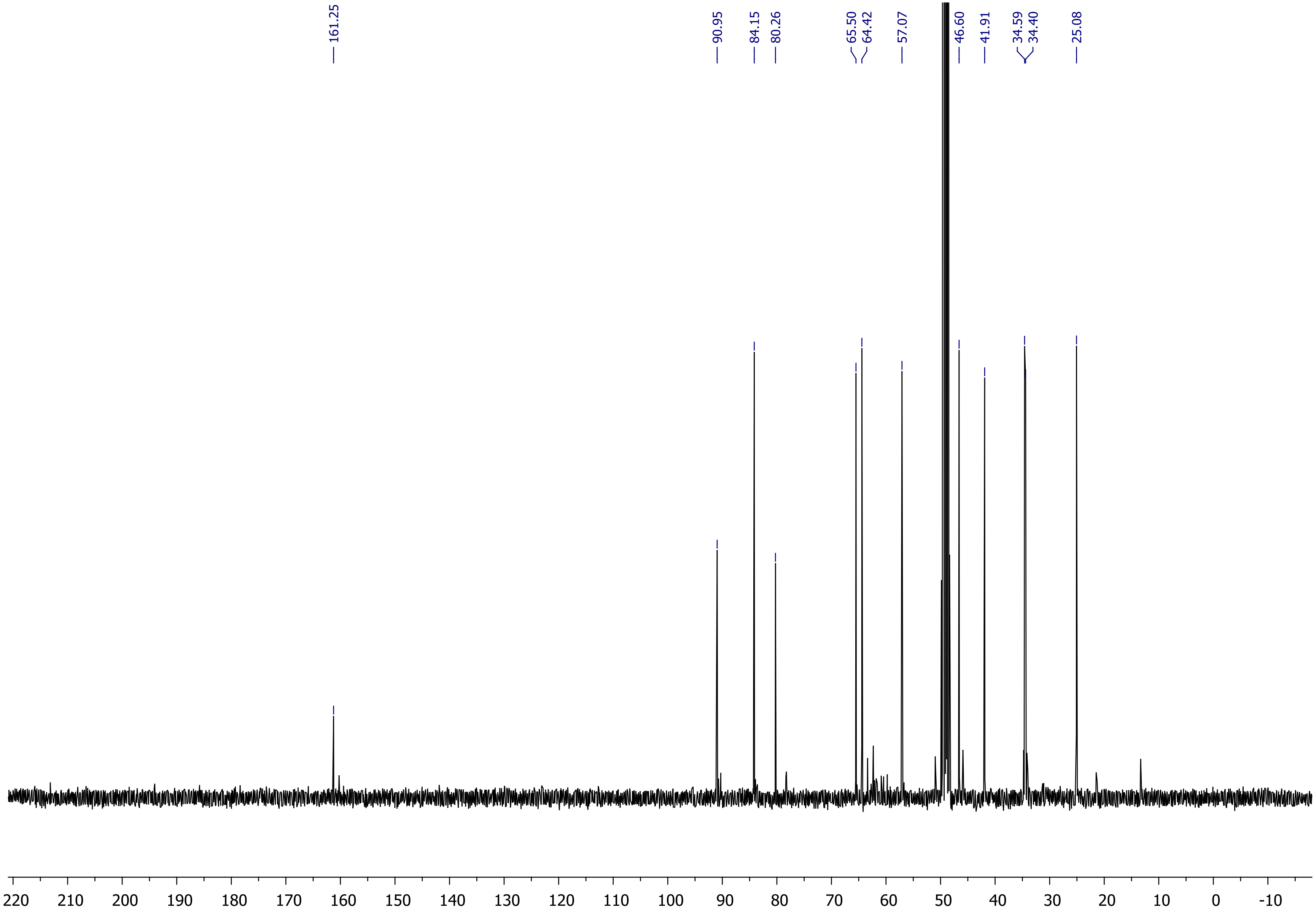
161.25
90.95
84.15
80.26
65.50
64.42
57.07
46.60
41.91
34.59
34.40
25.08
220 210 200 190 180 170 160 150 140 130 120 110 100 90 80 70 60 50 40 30 20 10 0 -10

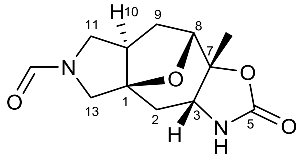

F8

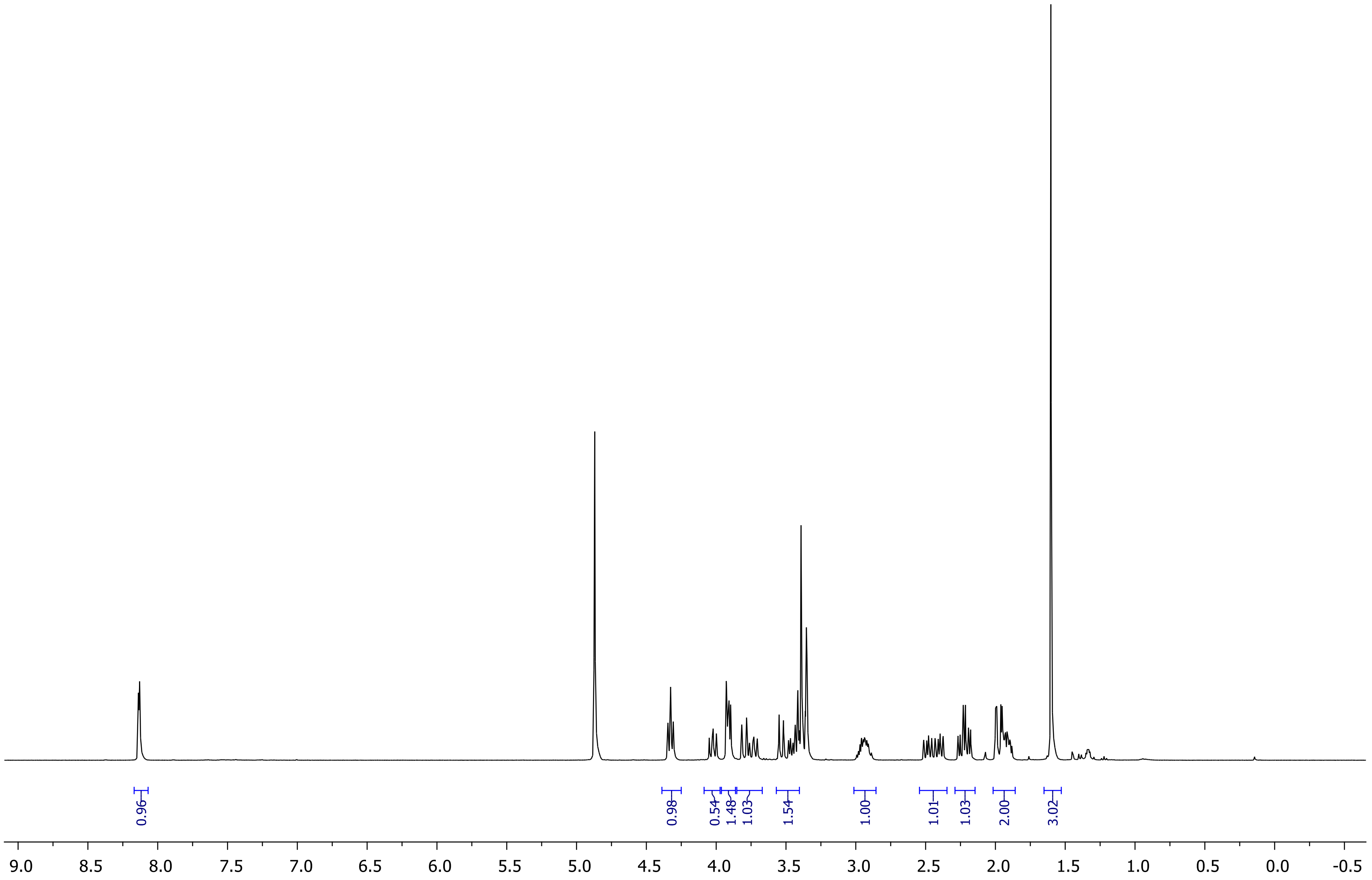

0.96
0.98
0.54
1.48
1.03
1.54
1.00
1.01
1.03
2.00
3.02
9.0
8.5
8.0
7.5
7.0
6.5
6.0
5.5
5.0
4.5
4.0
3.5
3.0
2.5
2.0
1.5
1.0
0.5
0.0
-0.5

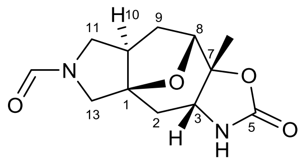
H
10
9
8
7
11
O
N
O
O
1
O
13
2
3
H
N
H
5
O

F8

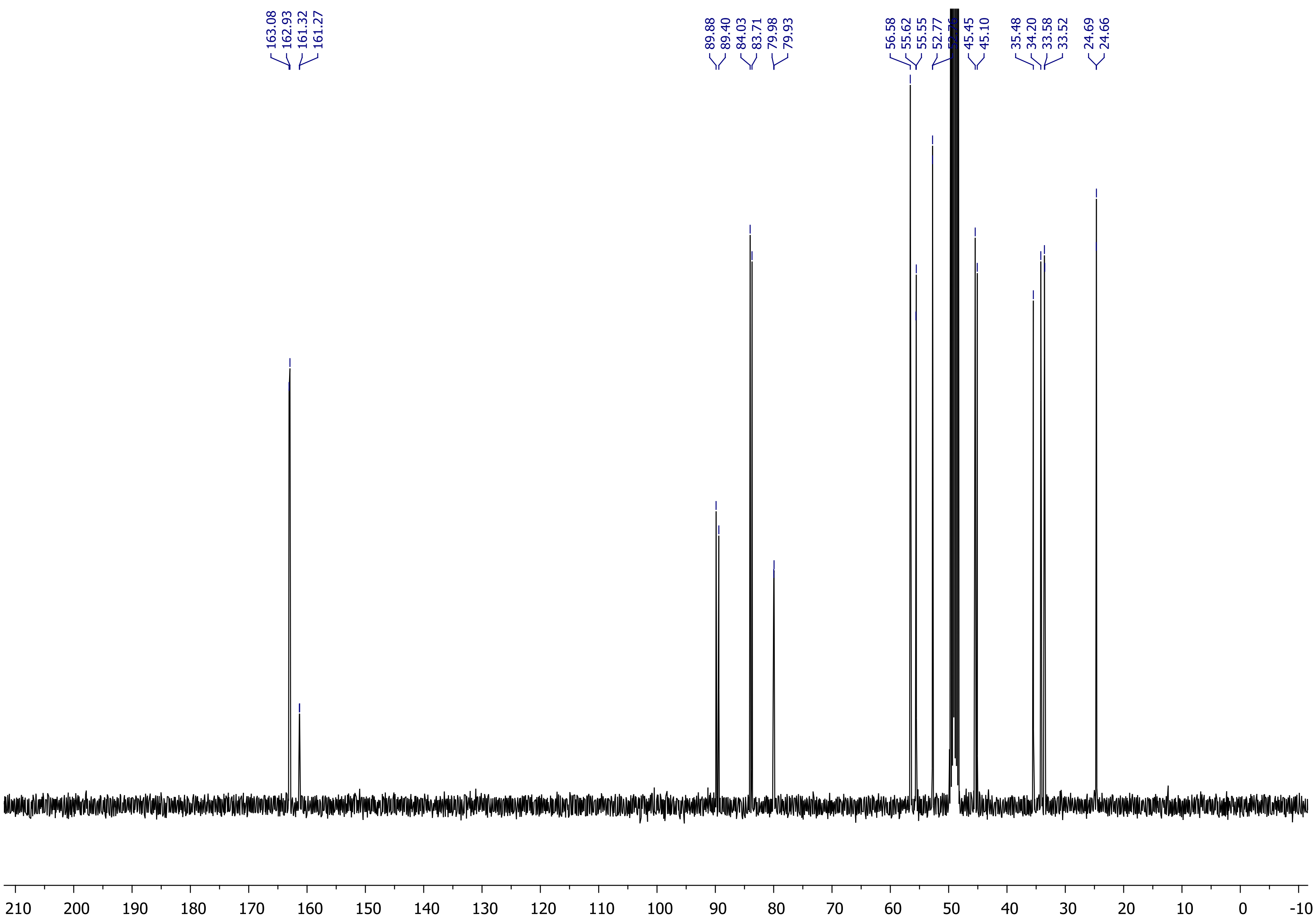
163.08
162.93
161.32
161.27
89.88
89.40
84.03
83.71
79.98
79.93
56.58
55.62
55.55
52.77
45.45
45.10
35.48
34.20
33.58
33.52
24.69
24.66
210
200
190
180
170
160
150
140
130
120
110
100
90
80
70
60
50
40
30
20
10
0
-10

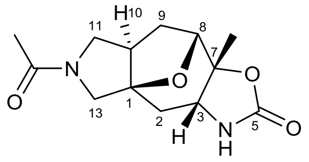
H
10
9
8
11
7
O
N
O
O
13
1
2
3
5
O
H
N
H

F9

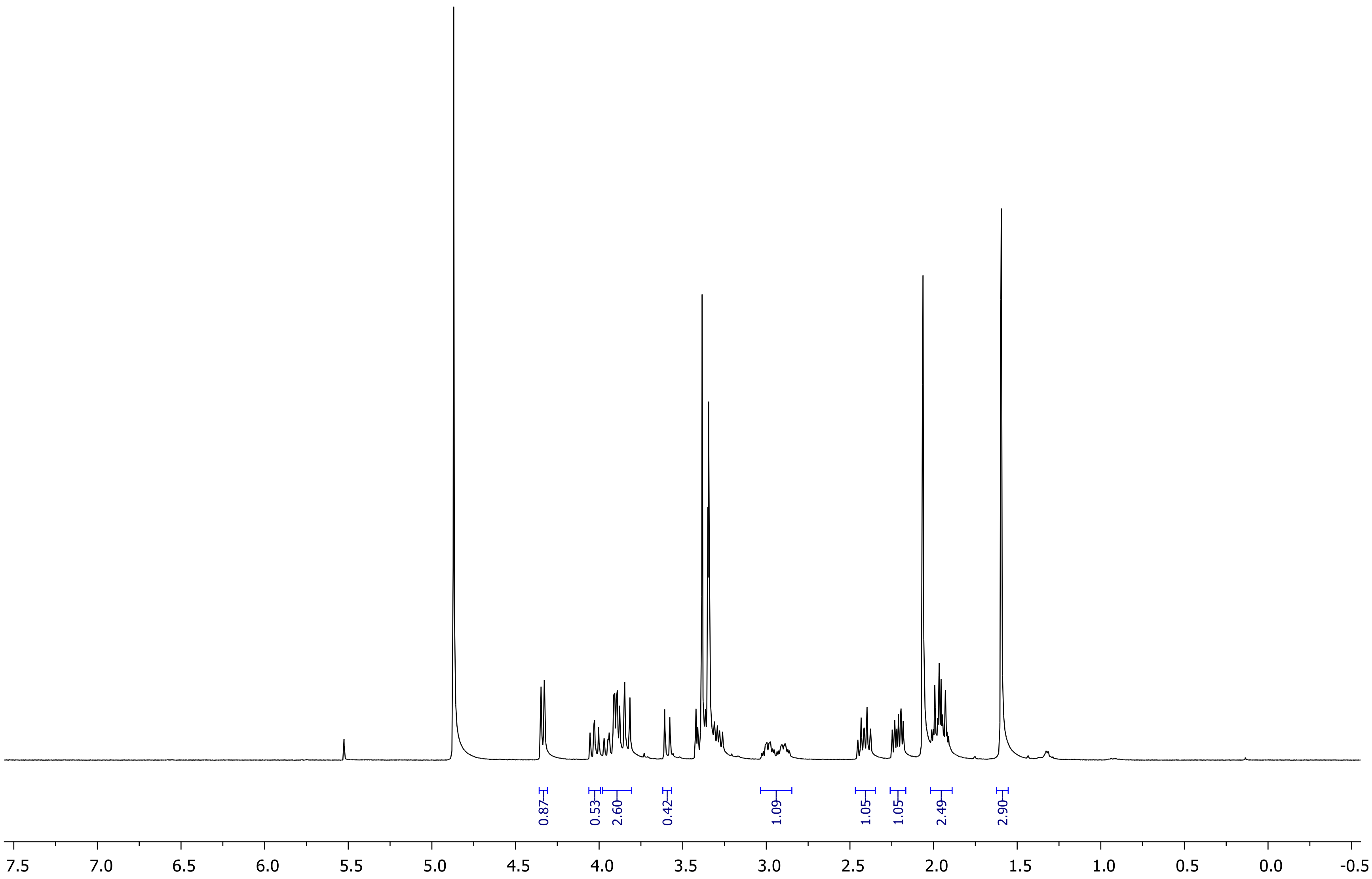
0.87
0.53
2.60
0.42
1.09
1.05
1.05
2.49
2.90
7.5
7.0
6.5
6.0
5.5
5.0
4.5
4.0
3.5
3.0
2.5
2.0
1.5
1.0
0.5
0.0
-0.5

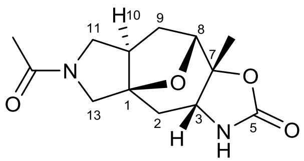

N
O
H 10
11
9
8
7
O
O
13
1
2
3
5
H
NH
H
O

F9

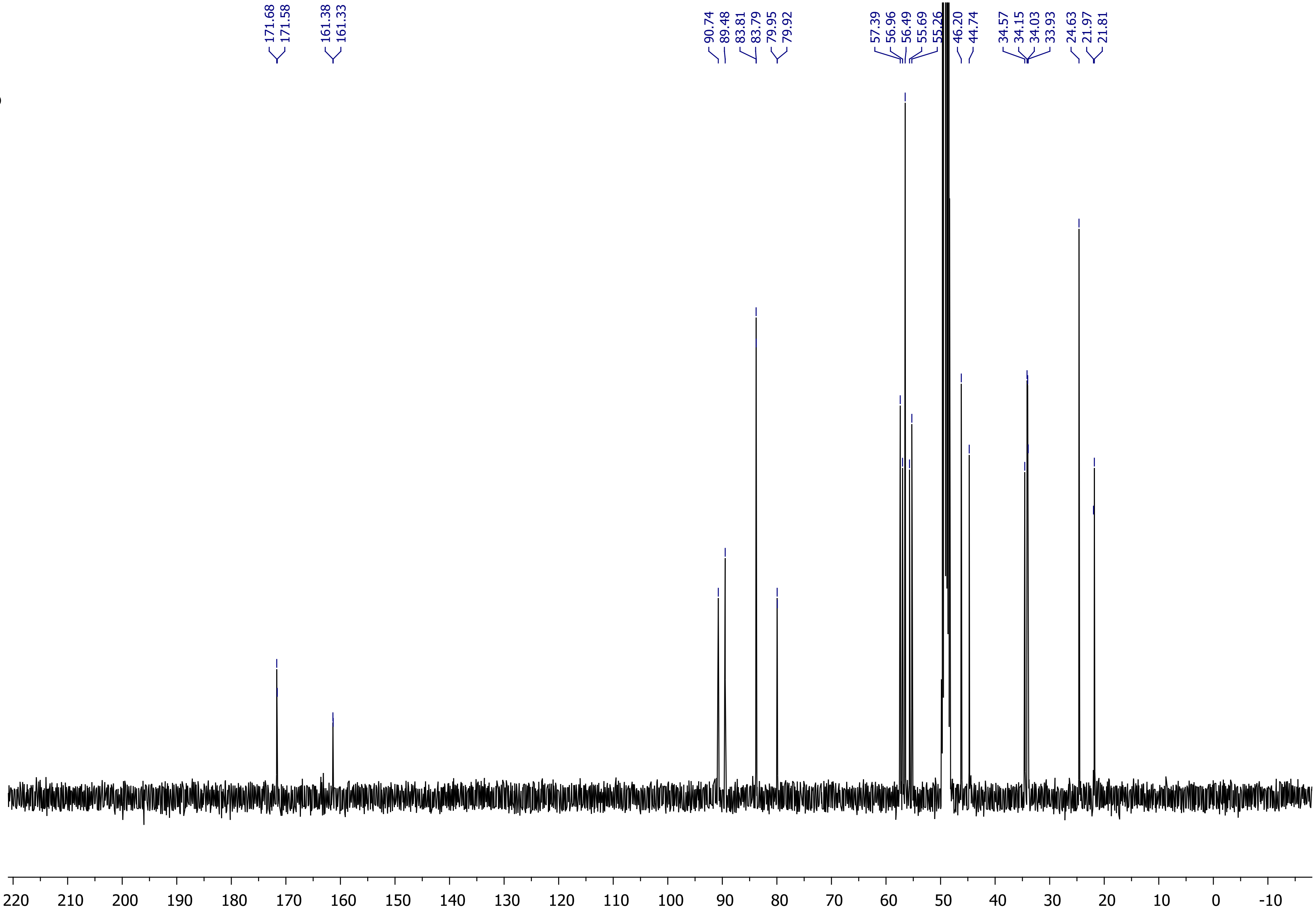

171.68
171.58
161.38
161.33
90.74
89.48
83.81
83.79
79.95
79.92
57.39
56.96
56.49
55.69
55.26
46.20
44.74
34.57
34.15
34.03
33.93
24.63
21.97
21.81
220 210 200 190 180 170 160 150 140 130 120 110 100 90 80 70 60 50 40 30 20 10 0 -10

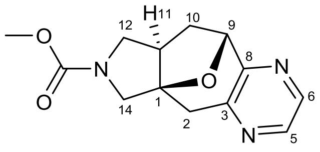

F10

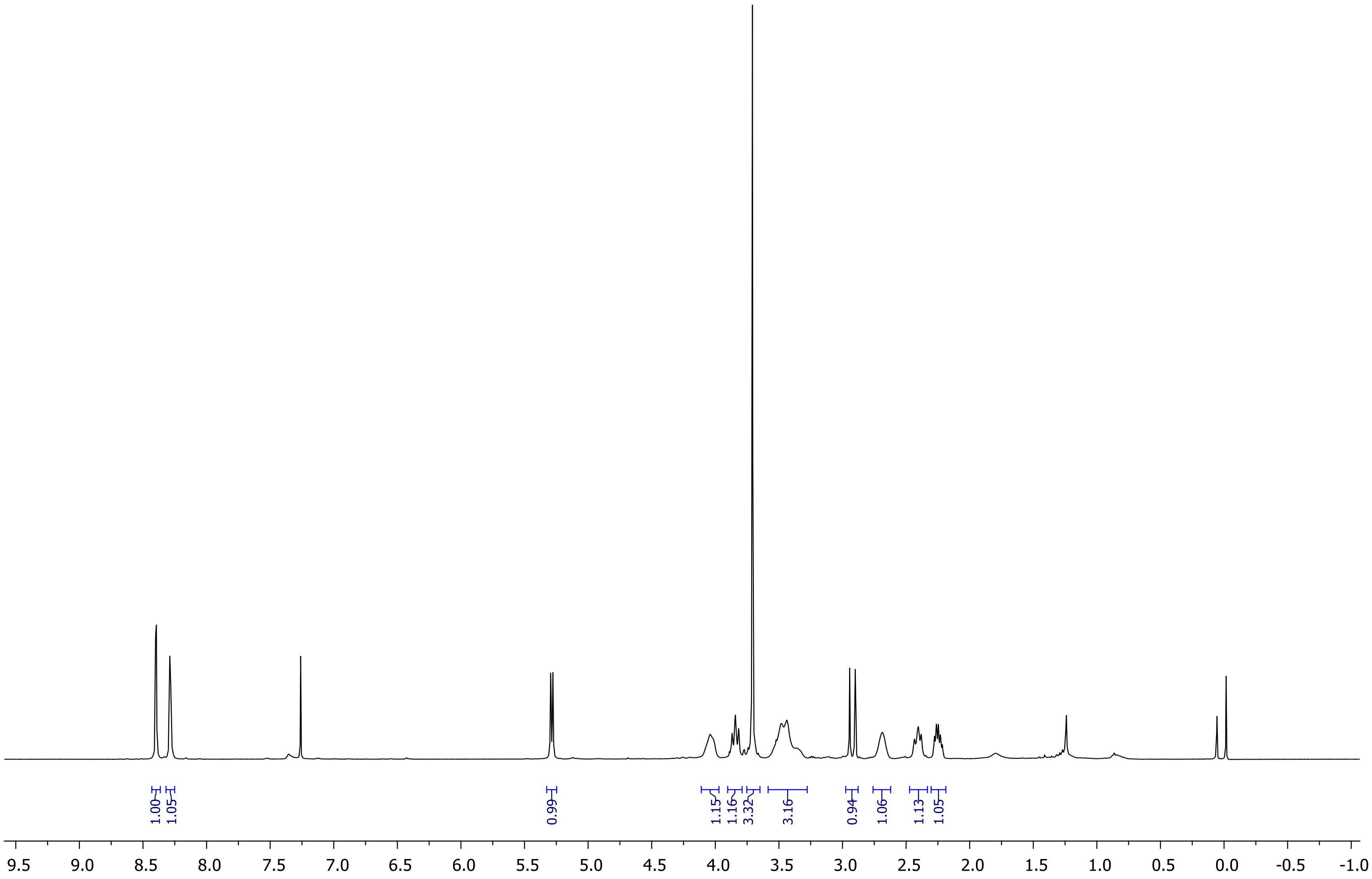

1.00
1.05
0.99
1.15
1.16
3.32
3.16
0.94
1.06
1.13
1.05
9.5
9.0
8.5
8.0
7.5
7.0
6.5
6.0
5.5
5.0
4.5
4.0
3.5
3.0
2.5
2.0
1.5
1.0
0.5
0.0
-0.5
-1.0

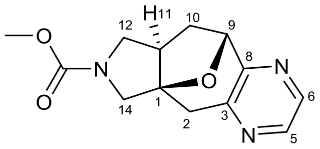

F10

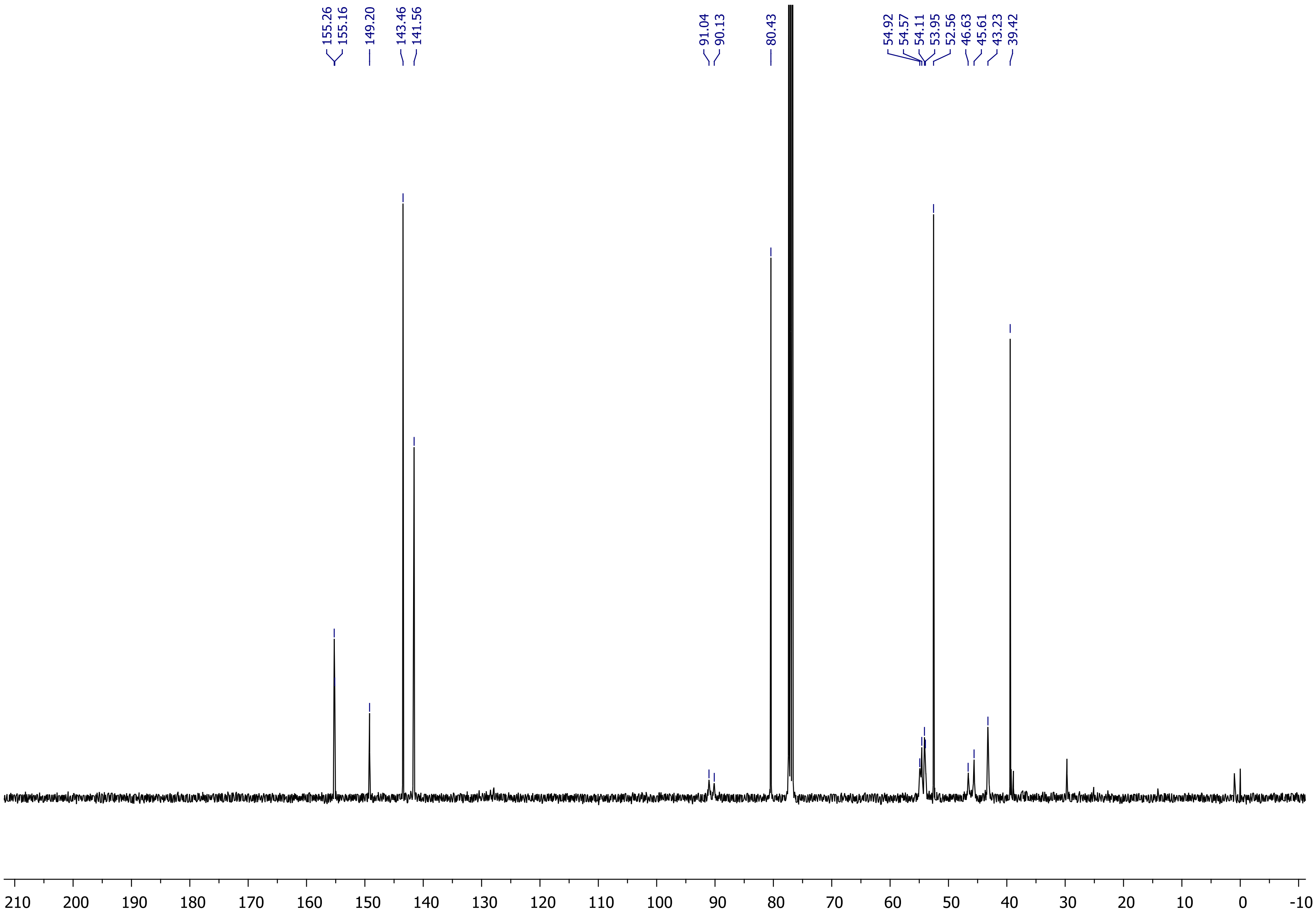

155.26
155.16
149.20
143.46
141.56
91.04
90.13
80.43
54.92
54.57
54.11
53.95
52.56
46.63
45.61
43.23
39.42
210 200 190 180 170 160 150 140 130 120 110 100 90 80 70 60 50 40 30 20 10 0 -10

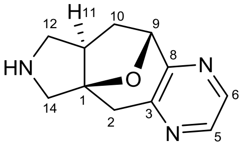
HN
O
N
N
H
1
2
3
5
6
8
9
10
11
12
14

F11

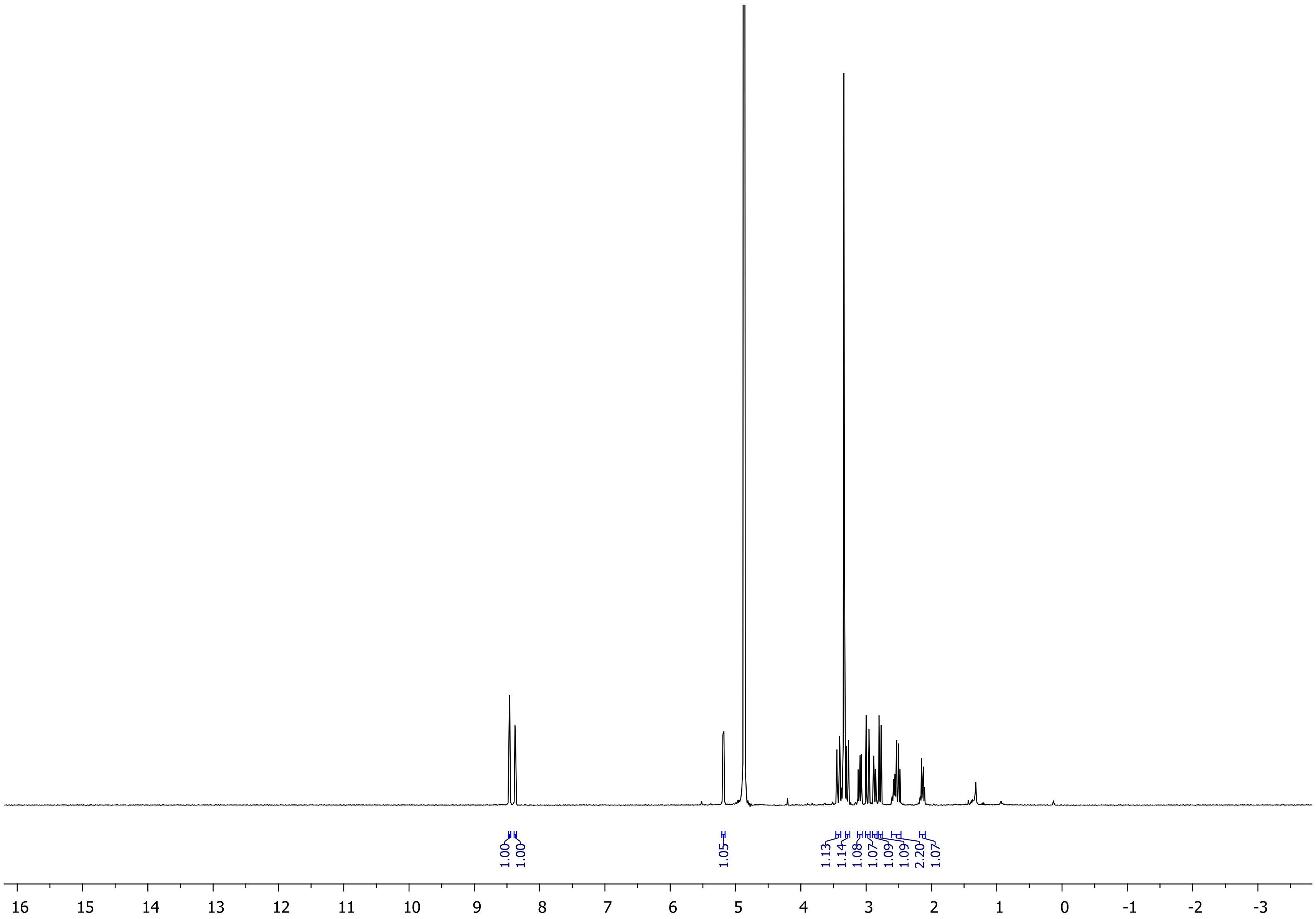
1.00
1.00
1.05
1.13
1.14
1.08
1.07
1.09
2.20
1.07
16
15
14
13
12
11
10
9
8
7
6
5
4
3
2
1
0
-1
-2
-3

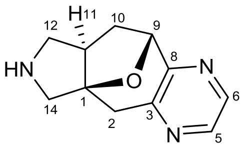
HN
O
N
N
H
1
2
3
5
6
8
9
10
11
12
14

F11

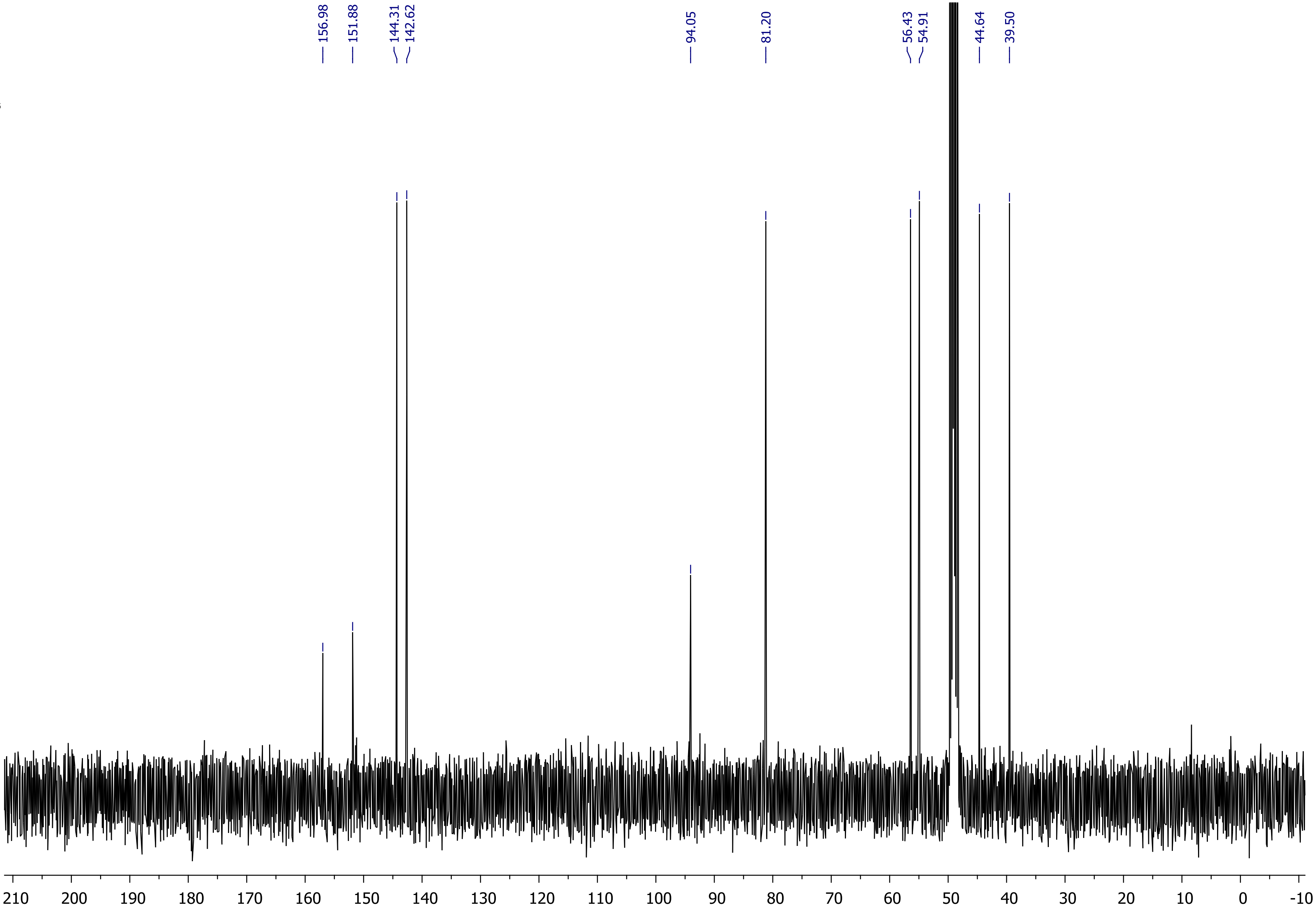
156.98
151.88
144.31
142.62
94.05
81.20
56.43
54.91
44.64
39.50
210 200 190 180 170 160 150 140 130 120 110 100 90 80 70 60 50 40 30 20 10 0 -10

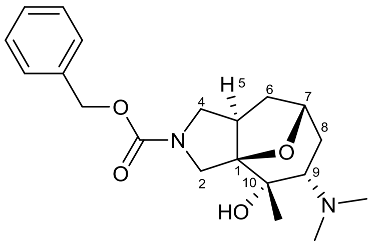
H
5
6
7
8
4
O
O
N
O
1
2
10
9
HO
N

S38

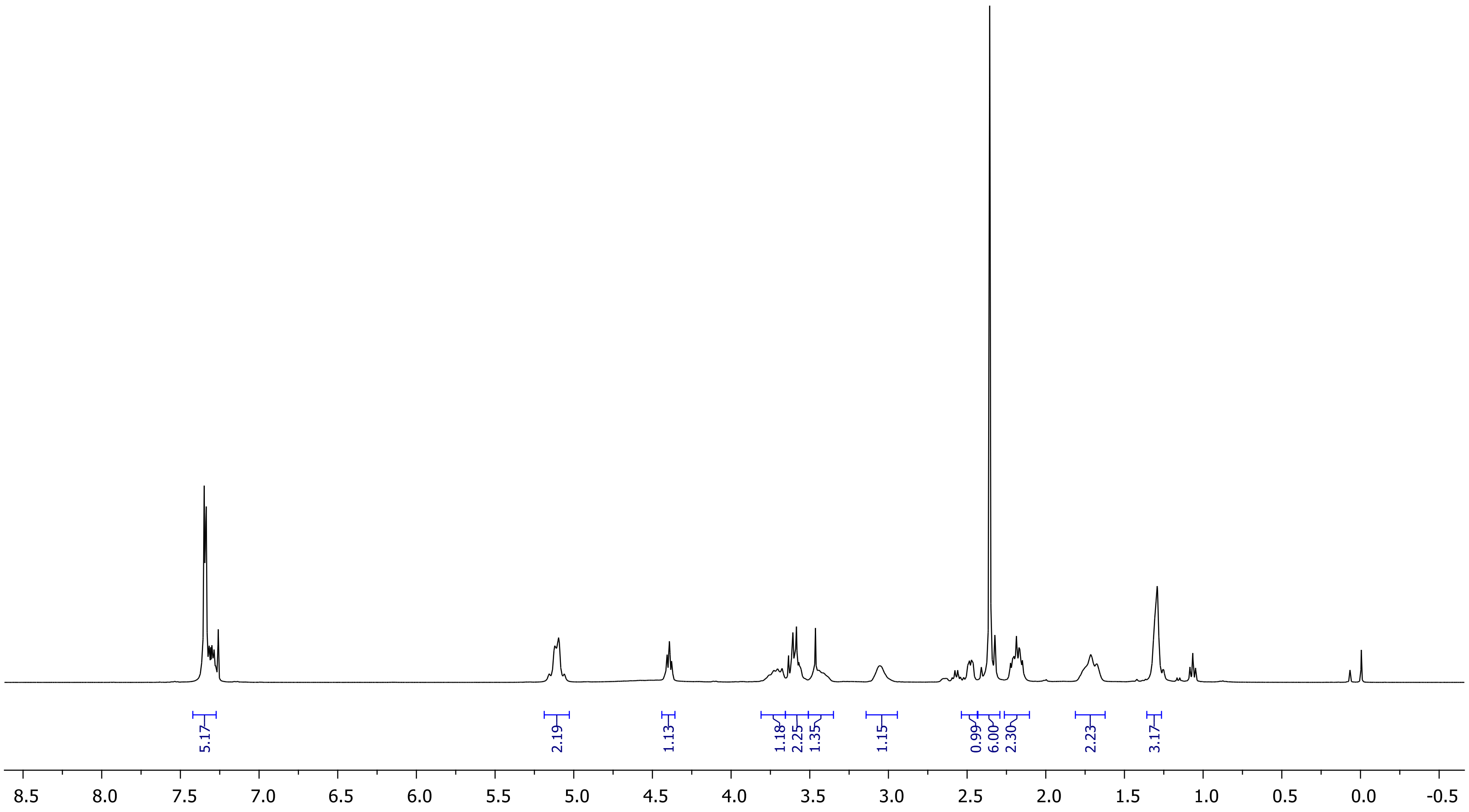
5.17
2.19
1.13
1.18
2.25
1.35
1.15
0.99
6.00
2.30
2.23
3.17
8.5
8.0
7.5
7.0
6.5
6.0
5.5
5.0
4.5
4.0
3.5
3.0
2.5
2.0
1.5
1.0
0.5
0.0
-0.5

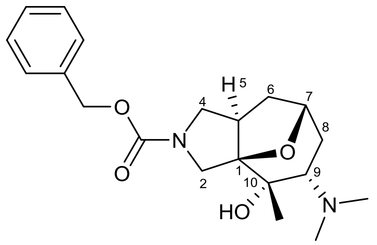
H
5
4
6
7
8
O
N
O
O
2
1
10
9
HO
N

S38

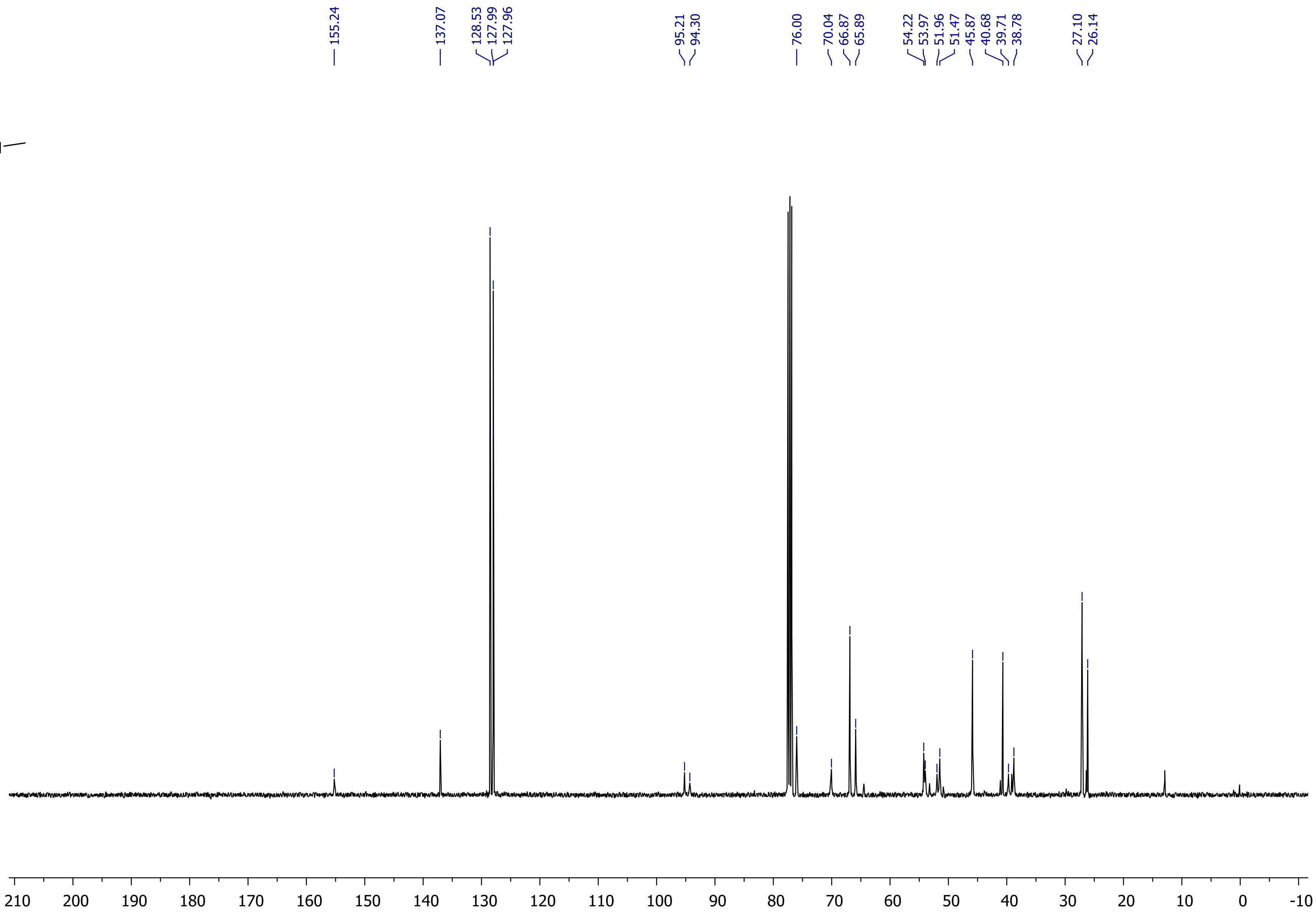
155.24
137.07
128.53
127.99
127.96
95.21
94.30
76.00
70.04
66.87
65.89
54.22
53.97
51.96
51.47
45.87
40.68
39.71
38.78
27.10
26.14
210
200
190
180
170
160
150
140
130
120
110
100
90
80
70
60
50
40
30
20
10
0
-10

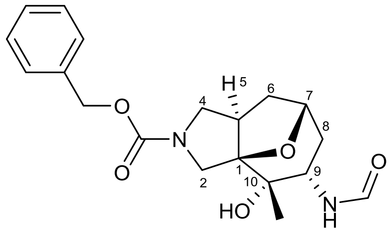
H
5
4
6
7
8
N
O
O
O
2
1
10
9
HO
N
H
O

S39

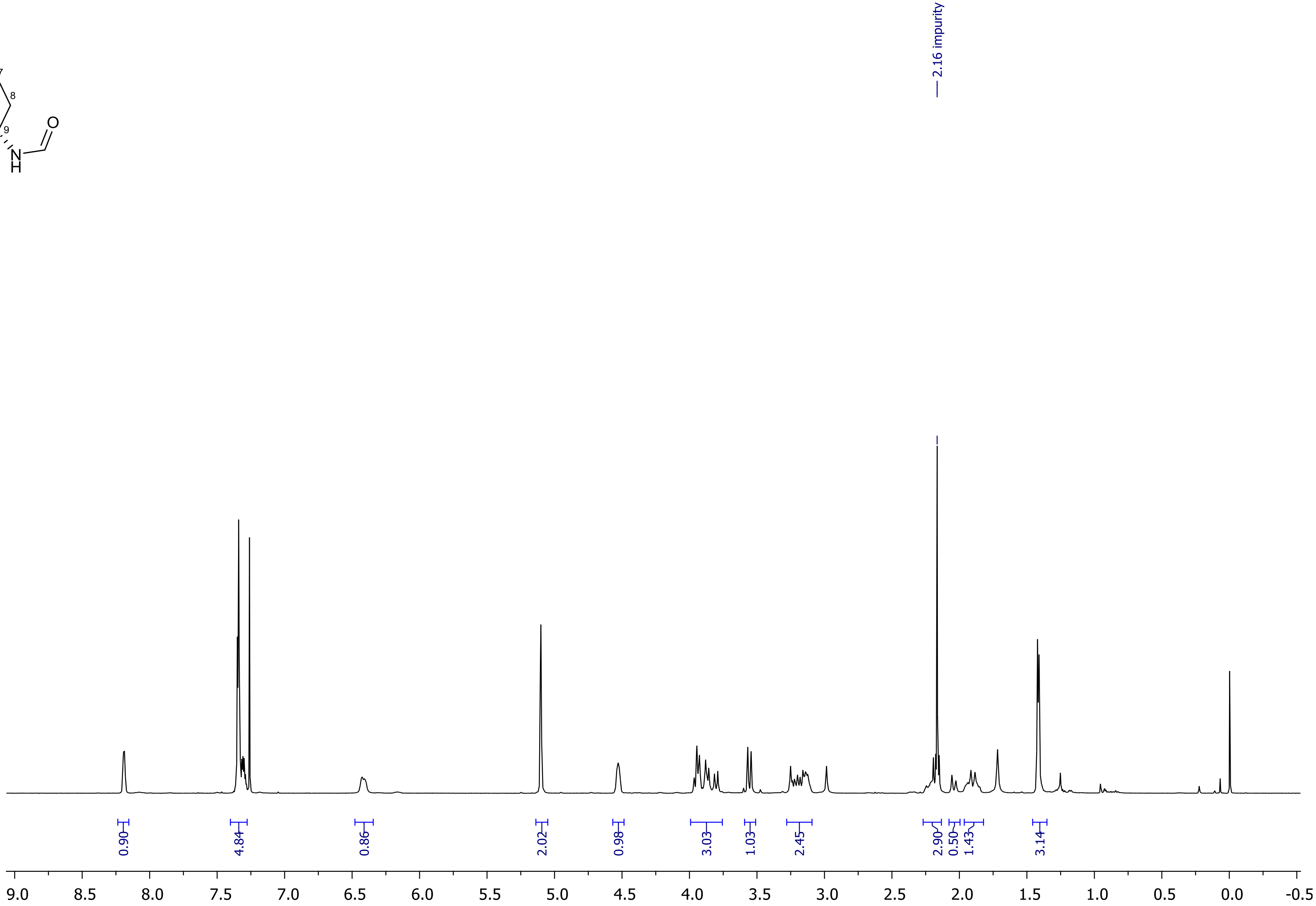
2.16 impurity
0.90
4.84
0.86
2.02
0.98
3.03
1.03
2.45
2.90
0.50
1.43
3.14
9.0
8.5
8.0
7.5
7.0
6.5
6.0
5.5
5.0
4.5
4.0
3.5
3.0
2.5
2.0
1.5
1.0
0.5
0.0
-0.5

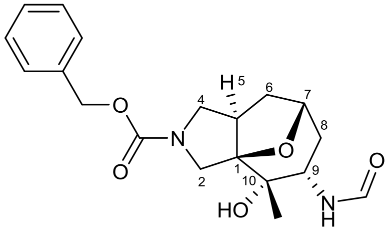
H 5
6
7
8
4
O
N
O
O
1
2
10
9
HO
N
H
O

S39

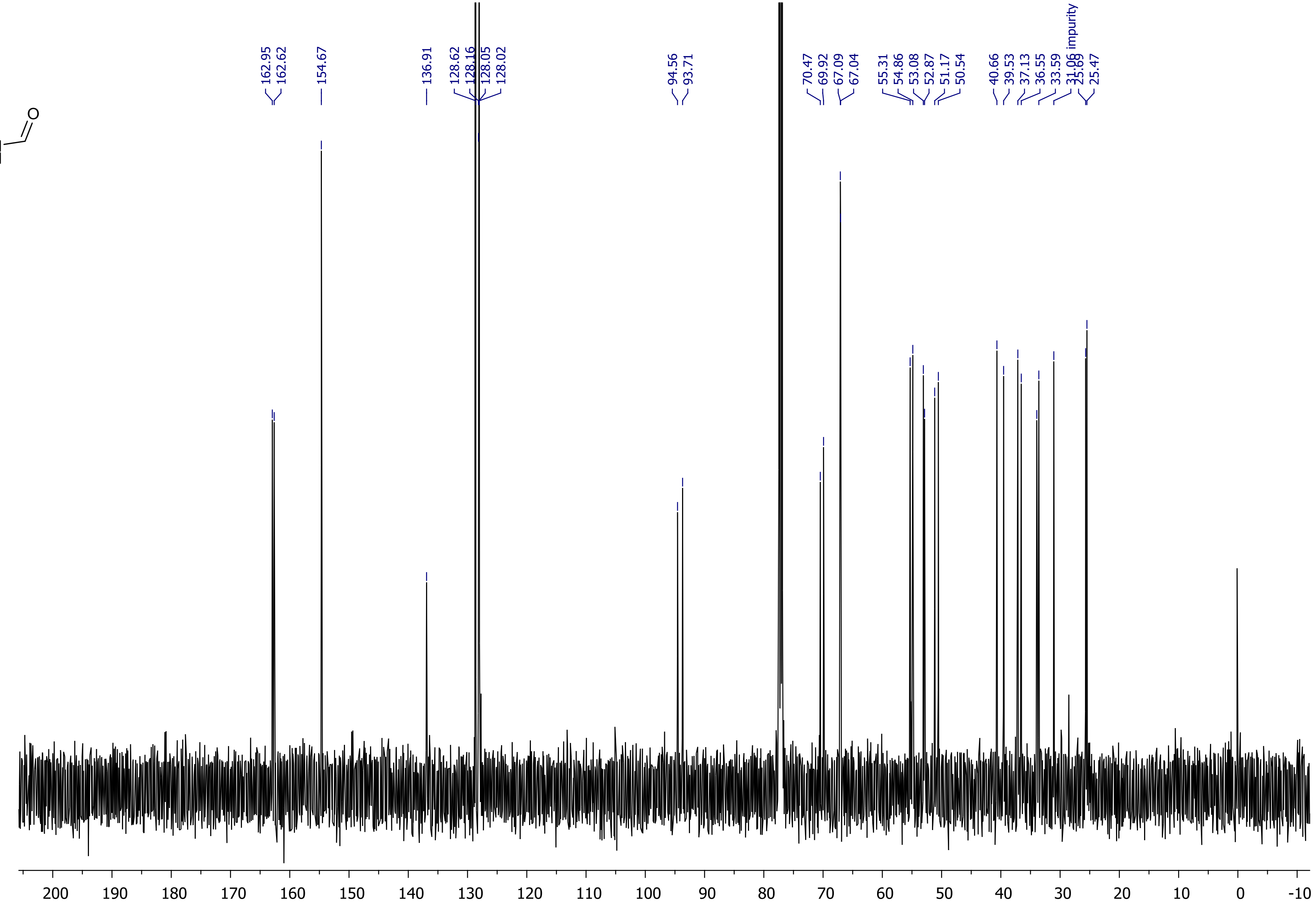
162.95
162.62
154.67
136.91
128.62
128.16
128.05
128.02
94.56
93.71
70.47
69.92
67.09
67.04
55.31
54.86
53.08
52.87
51.17
50.54
40.66
39.53
37.13
36.55
33.59
31.06 impurity
25.69
25.47
200 190 180 170 160 150 140 130 120 110 100 90 80 70 60 50 40 30 20 10 0 -10

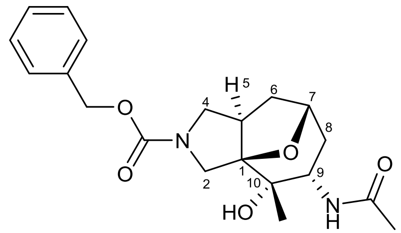

S40

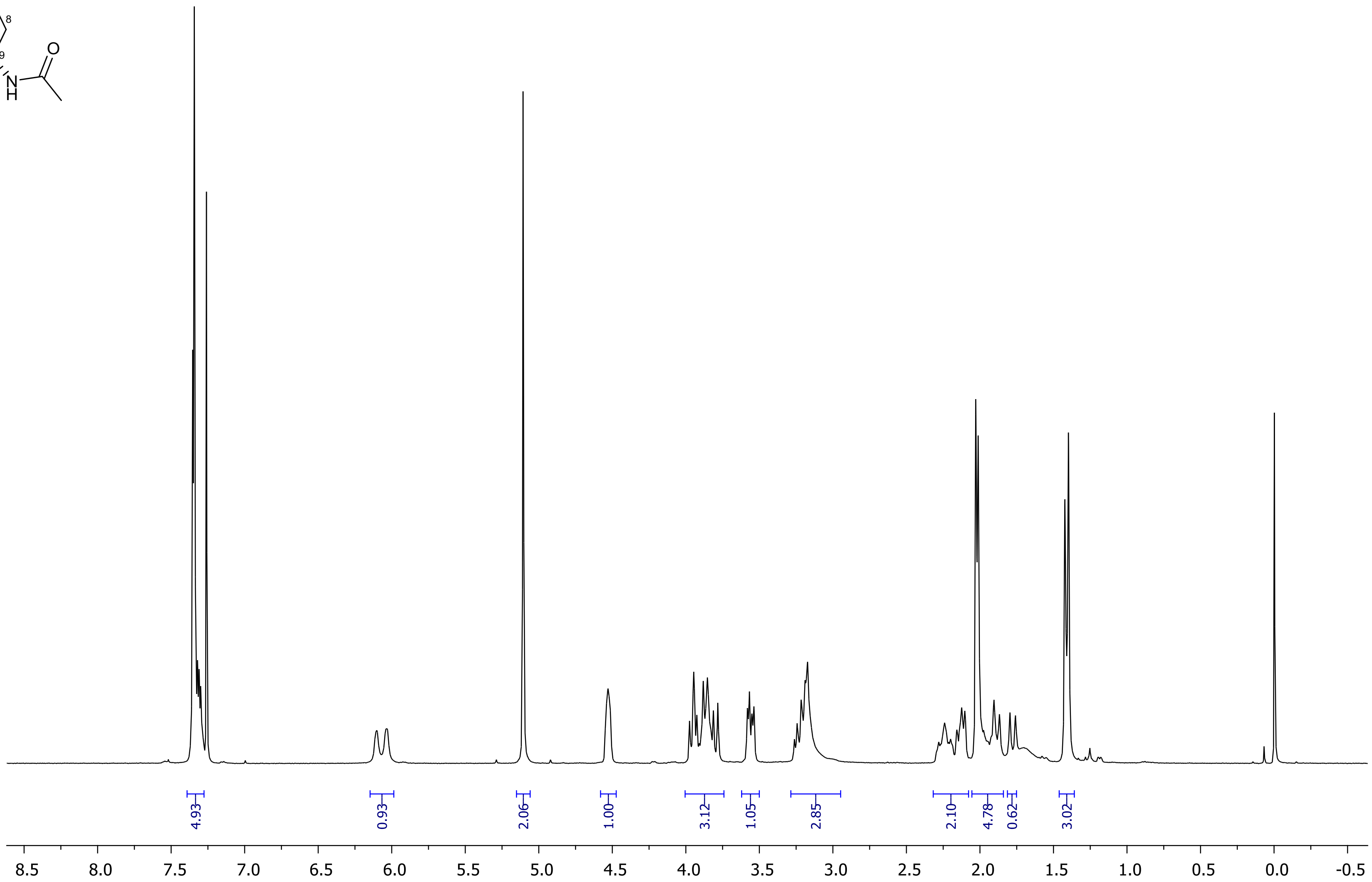

4.93
0.93
2.06
1.00
3.12
1.05
2.85
2.10
4.78
0.62
3.02
8.5
8.0
7.5
7.0
6.5
6.0
5.5
5.0
4.5
4.0
3.5
3.0
2.5
2.0
1.5
1.0
0.5
0.0
-0.5

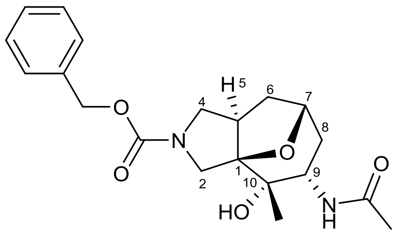
H
5
6
7
8
4
N
O
O
O
1
2
10
9
HO
N
H
O

S40

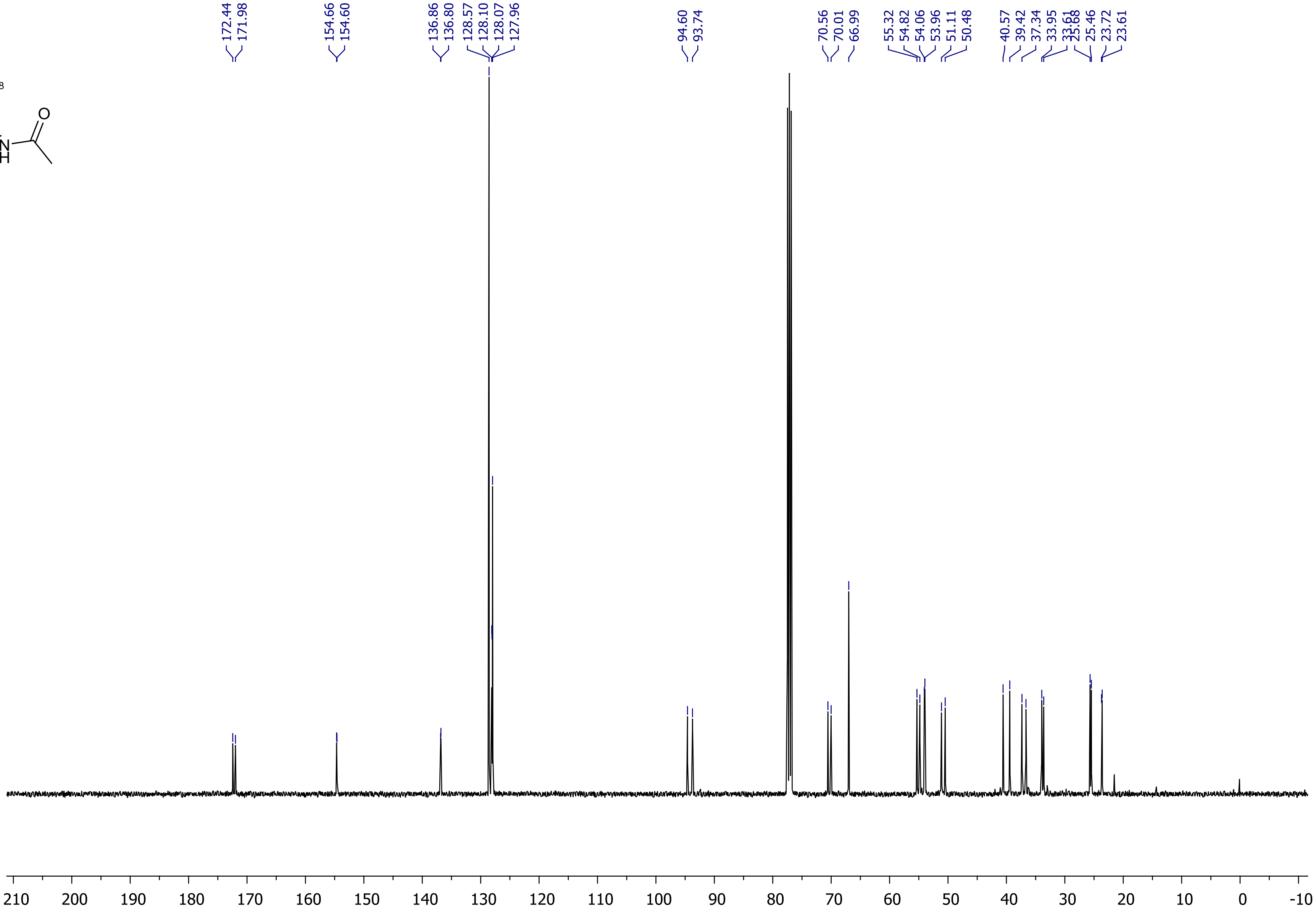
172.44
171.98
154.66
154.60
136.86
136.80
128.57
128.10
128.07
127.96
94.60
93.74
70.56
70.01
66.99
55.32
54.82
54.06
53.96
51.11
50.48
40.57
39.42
37.34
33.95
33.61
33.68
25.46
23.72
23.61
210
200
190
180
170
160
150
140
130
120
110
100
90
80
70
60
50
40
30
20
10
0
-10

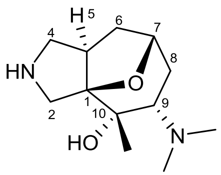

F13

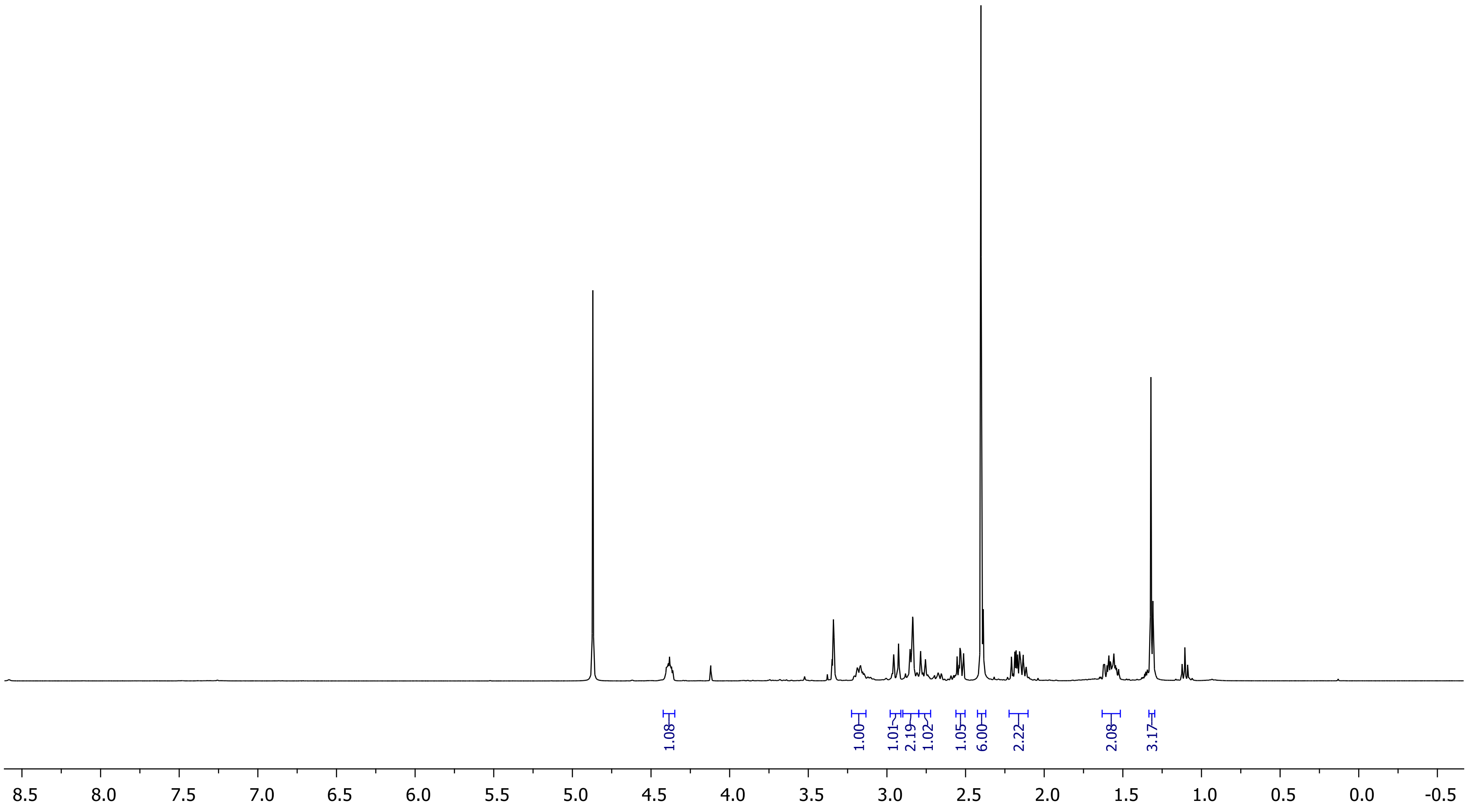
1.08
1.00
1.01
2.19
1.02
1.05
6.00
2.22
2.08
3.17
8.5
8.0
7.5
7.0
6.5
6.0
5.5
5.0
4.5
4.0
3.5
3.0
2.5
2.0
1.5
1.0
0.5
0.0
-0.5

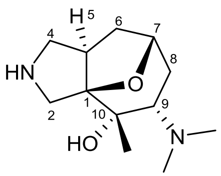
HN
O
HO
N
1
2
4
5
6
7
8
9
10

F13

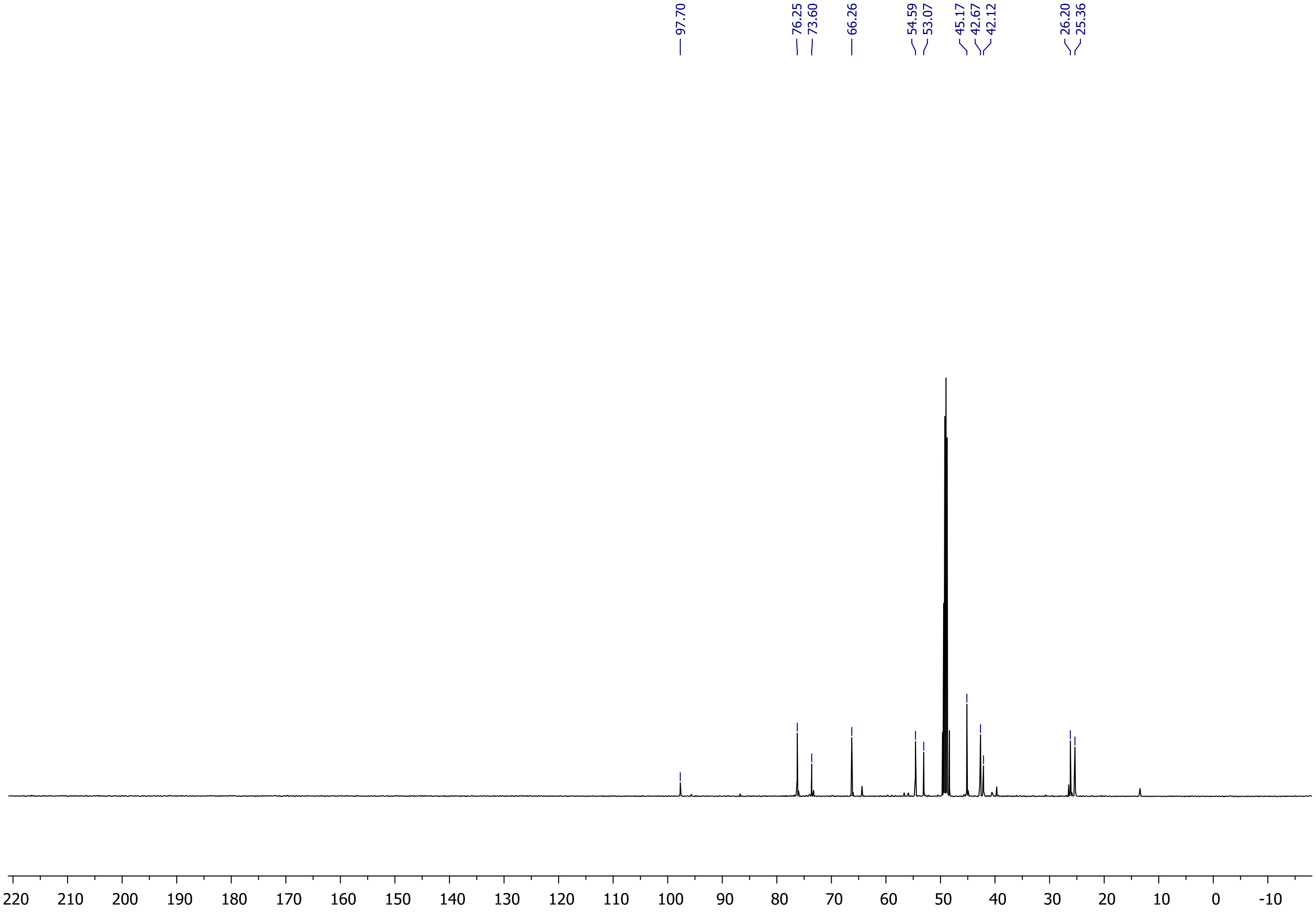
97.70
76.25
73.60
66.26
54.59
53.07
45.17
42.67
42.12
26.20
25.36
220 210 200 190 180 170 160 150 140 130 120 110 100 90 80 70 60 50 40 30 20 10 0 -10

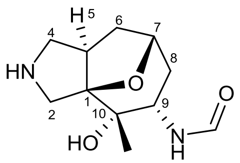

H
5
6
7
4
8
HN
O
O
2
1
10
9
HO
N
H

F14

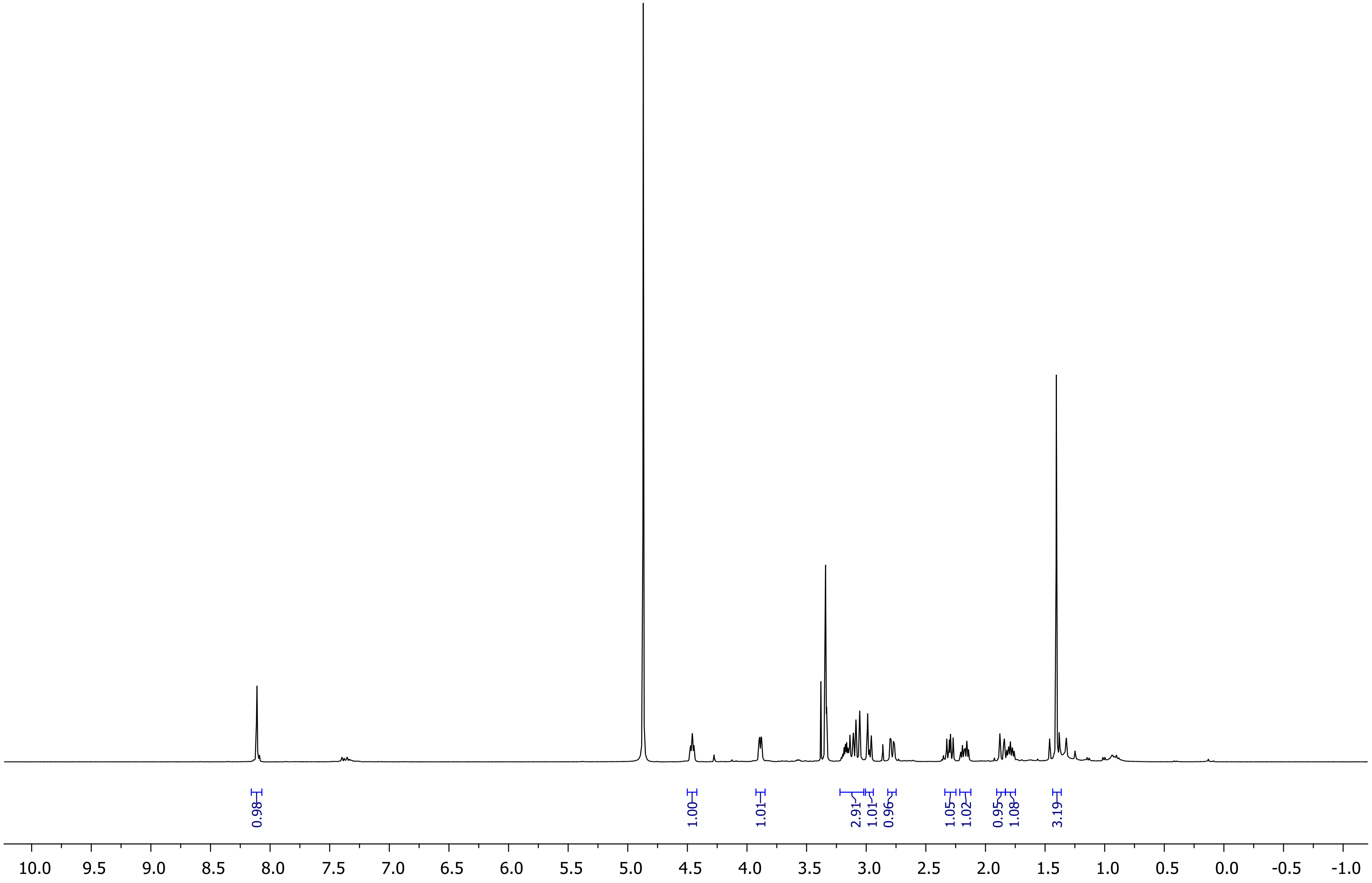

0.98
1.00
1.01
2.91
1.01
0.96
1.05
1.02
0.95
1.08
3.19
10.0
9.5
9.0
8.5
8.0
7.5
7.0
6.5
6.0
5.5
5.0
4.5
4.0
3.5
3.0
2.5
2.0
1.5
1.0
0.5
0.0
-0.5
-1.0

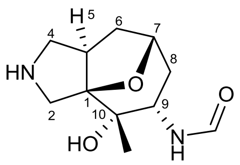
H
5
4
6
7
8
HN
O
O
1
9
2
10
HO
N
H

F14

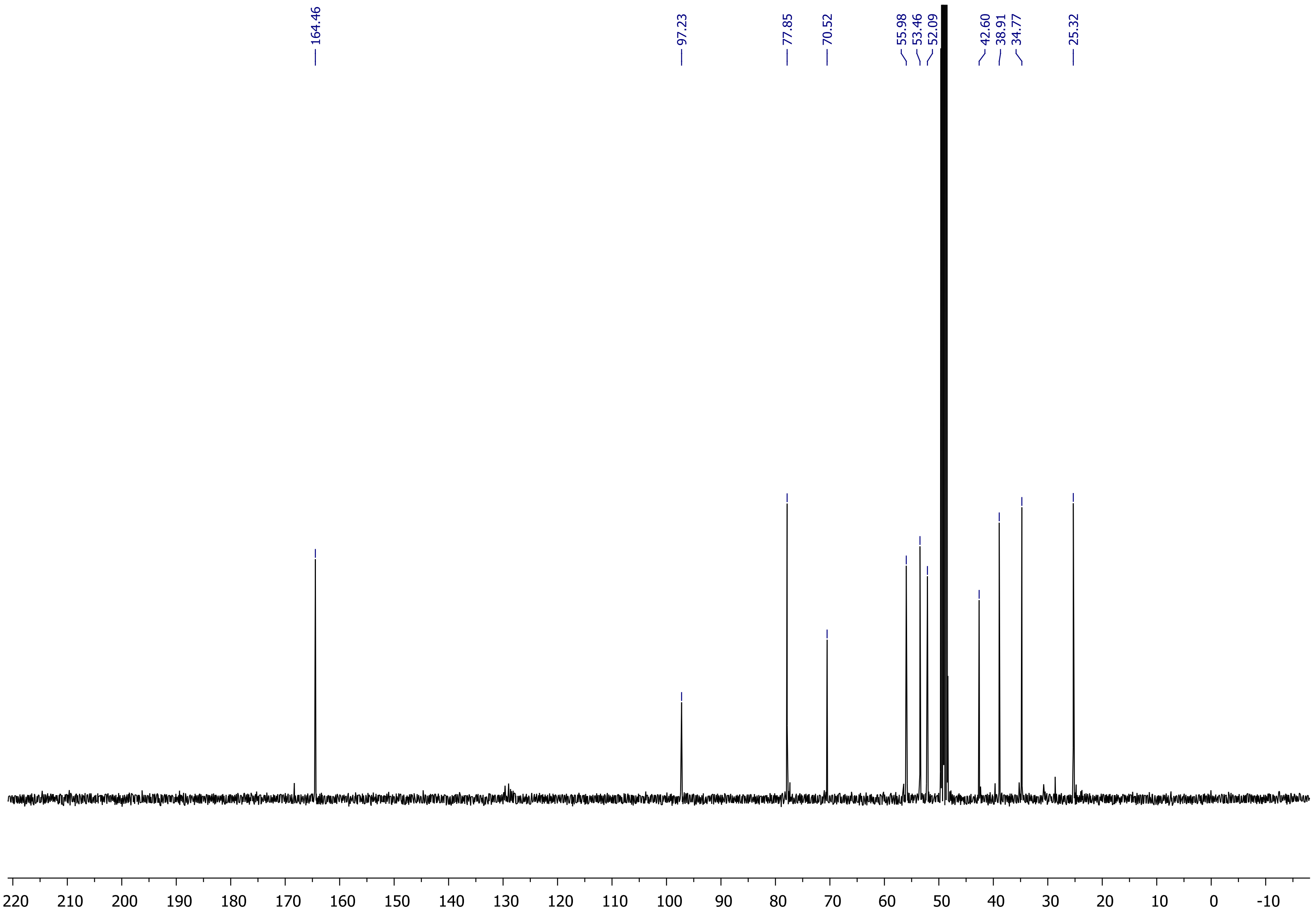
164.46
97.23
77.85
70.52
55.98
53.46
52.09
42.60
38.91
34.77
25.32
220
210
200
190
180
170
160
150
140
130
120
110
100
90
80
70
60
50
40
30
20
10
0
-10

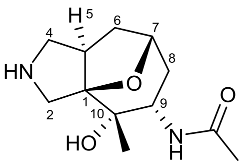
HN
O
HO
N
H
O
1
2
4
5
6
7
8
9
10

F15

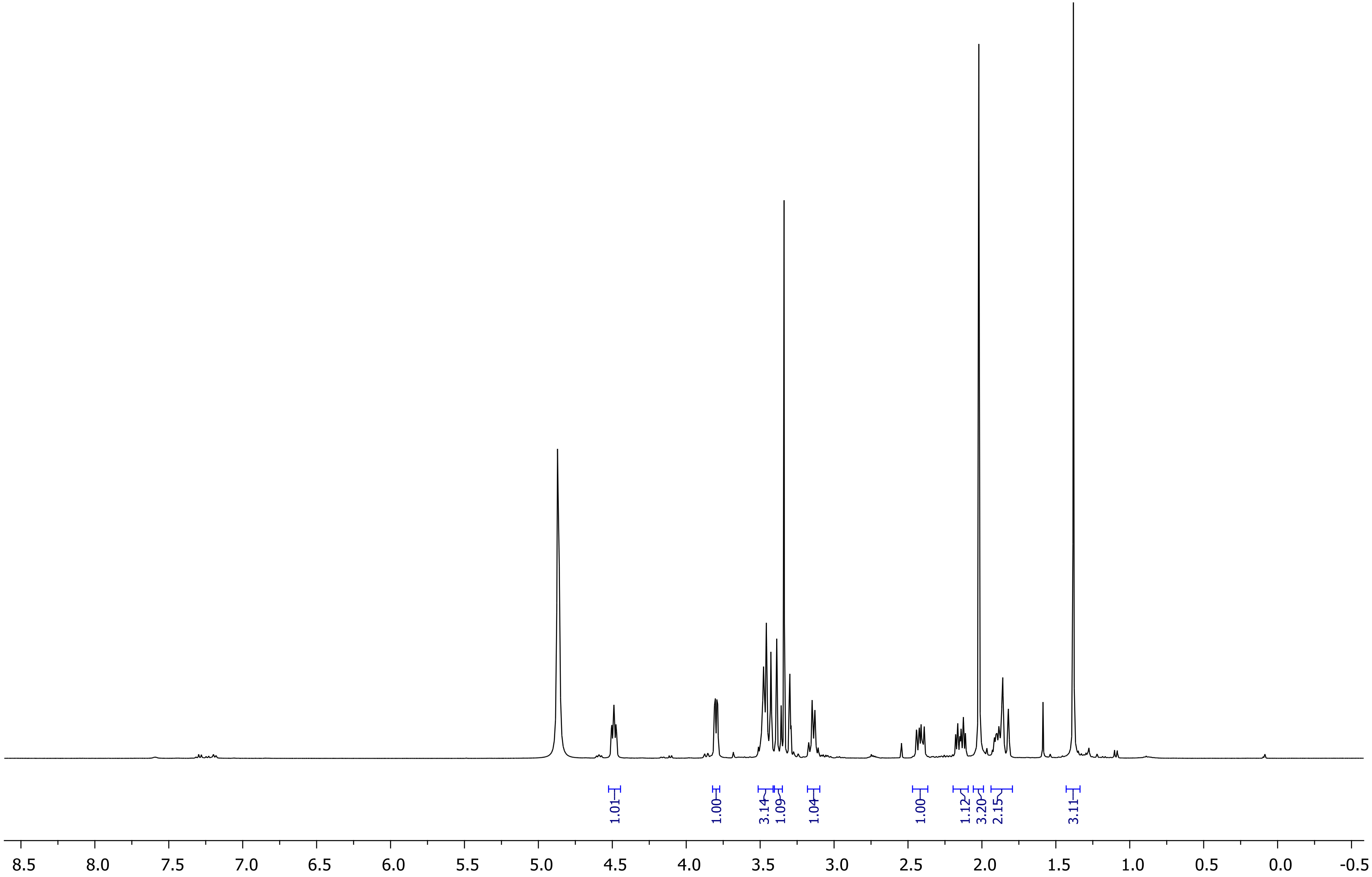
1.01
1.00
3.14
1.09
1.04
1.00
1.12
3.20
2.15
3.11
8.5
8.0
7.5
7.0
6.5
6.0
5.5
5.0
4.5
4.0
3.5
3.0
2.5
2.0
1.5
1.0
0.5
0.0
-0.5

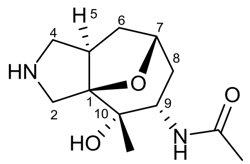
HN
H
O
HO
N
H
O
1
2
4
5
6
7
8
9
10

F15

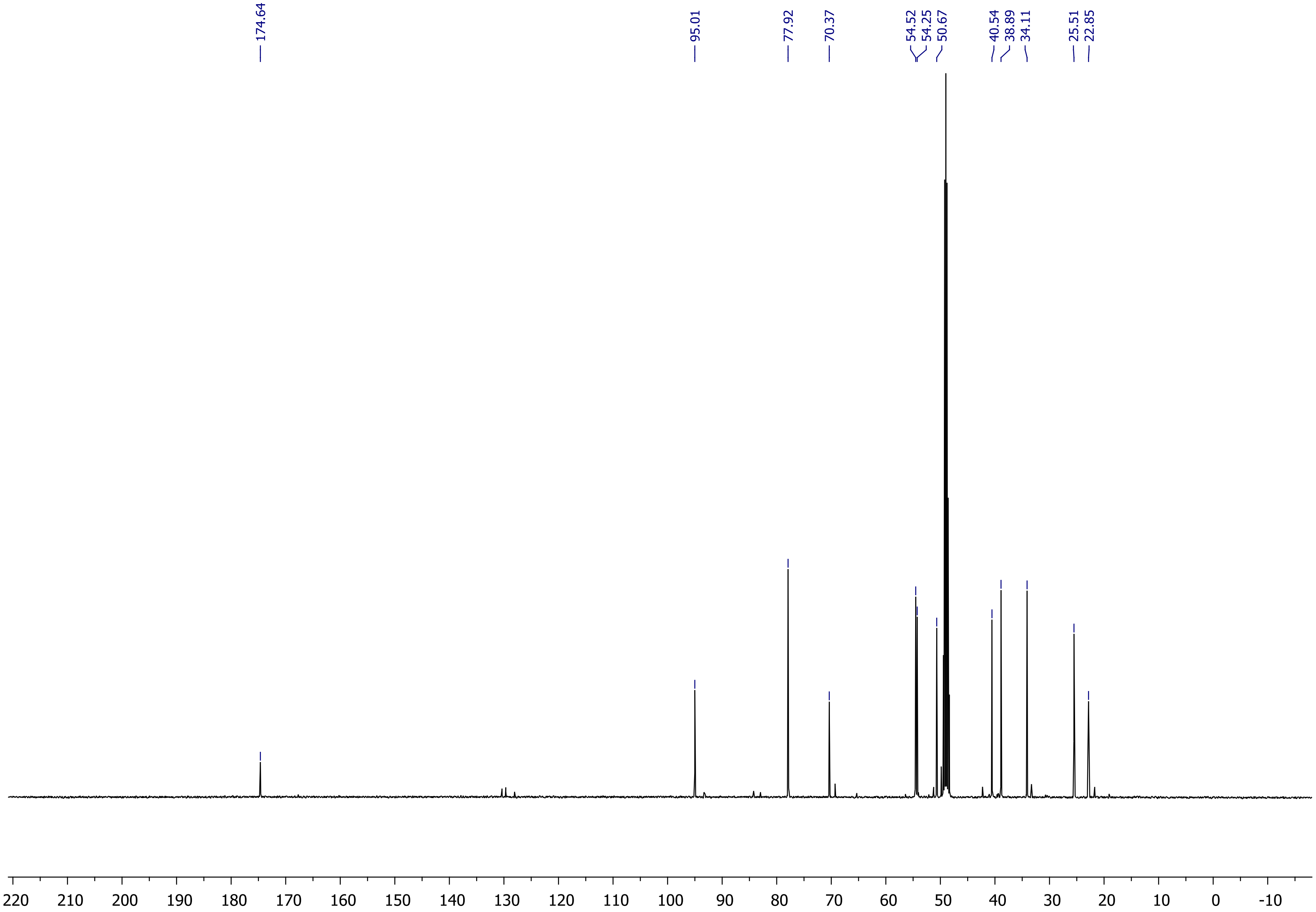
174.64
95.01
77.92
70.37
54.52
54.25
50.67
40.54
38.89
34.11
25.51
22.85
220 210 200 190 180 170 160 150 140 130 120 110 100 90 80 70 60 50 40 30 20 10 0 -10

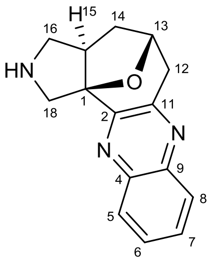
H
15
14
13
16
HN
O
12
18
1
2
11
N
N
4
9
8
5
6
7

F16

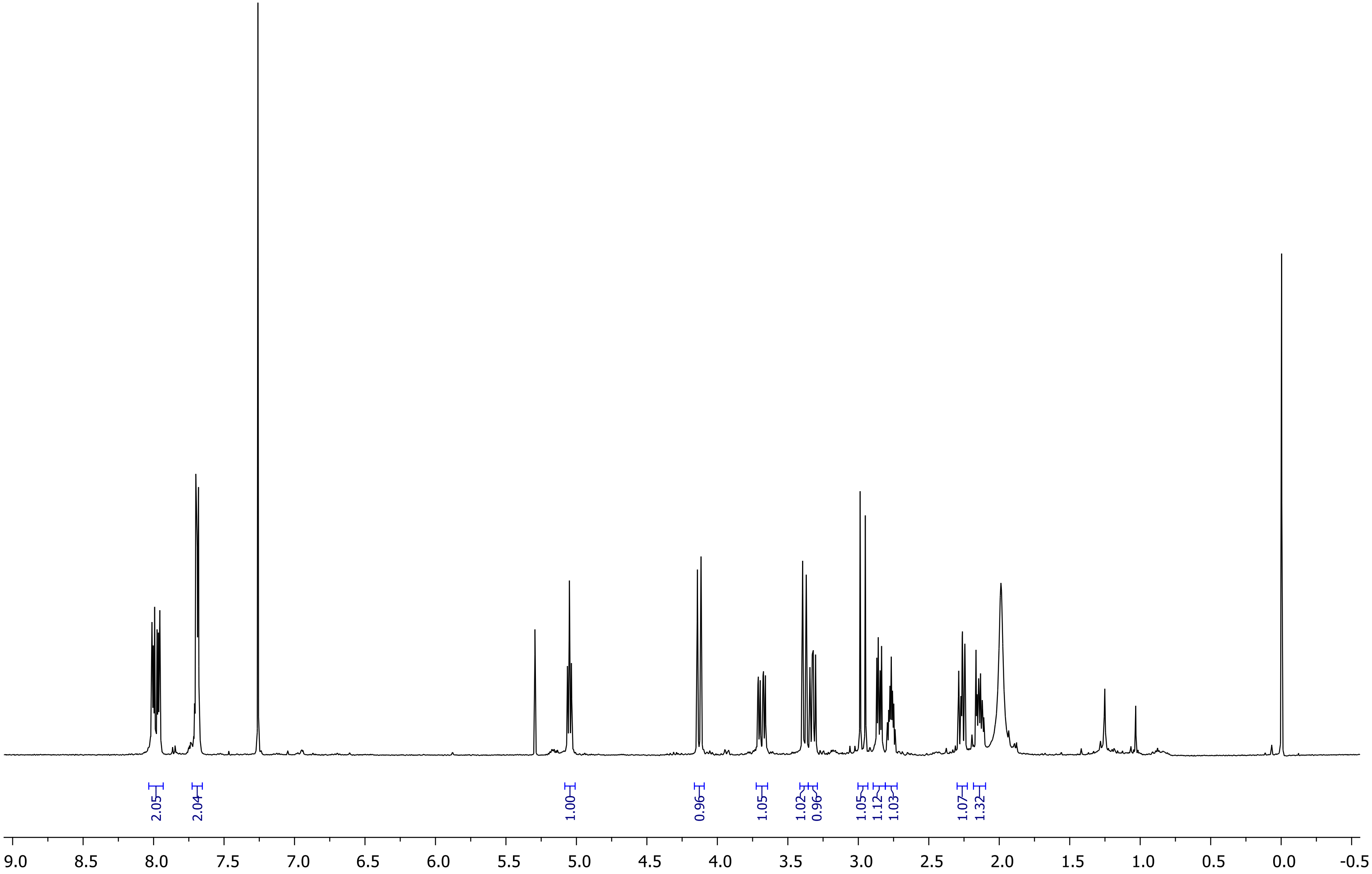
2.05
2.04
1.00
0.96
1.05
1.02
0.96
1.05
1.12
1.03
1.07
1.32
9.0
8.5
8.0
7.5
7.0
6.5
6.0
5.5
5.0
4.5
4.0
3.5
3.0
2.5
2.0
1.5
1.0
0.5
0.0
-0.5

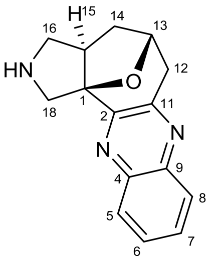
H
15
14
13
16
HN
O
12
18
1
11
2
N
N
4
9
8
5
6
7

F16

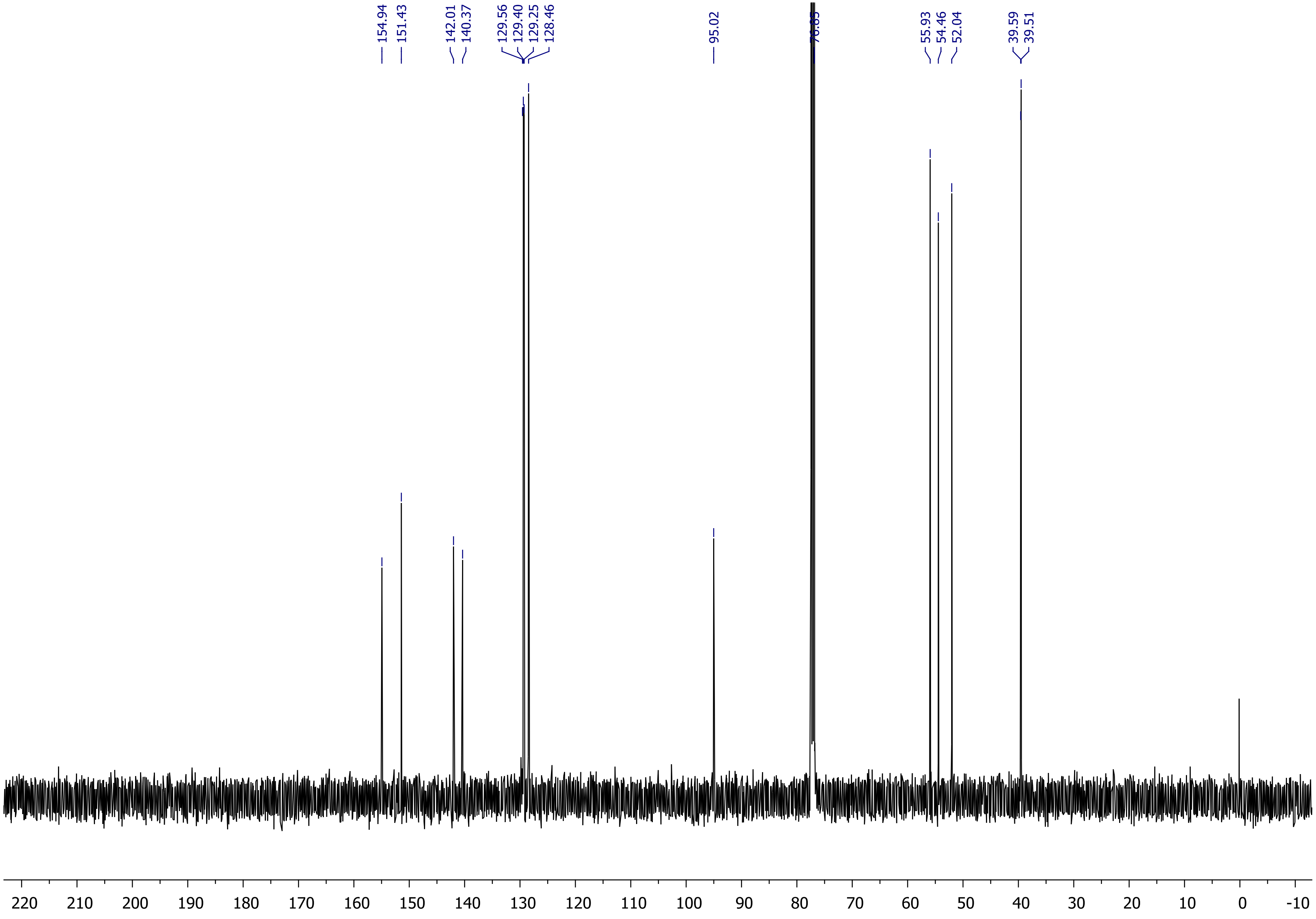
154.94
151.43
142.01
140.37
129.56
129.40
129.25
128.46
95.02
55.93
54.46
52.04
39.59
39.51
220 210 200 190 180 170 160 150 140 130 120 110 100 90 80 70 60 50 40 30 20 10 0 -10

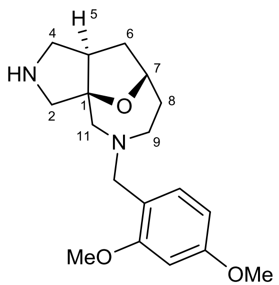

H
5
4
6
HN
7
O
1
8
2
11
N
9
MeO
OMe

S41

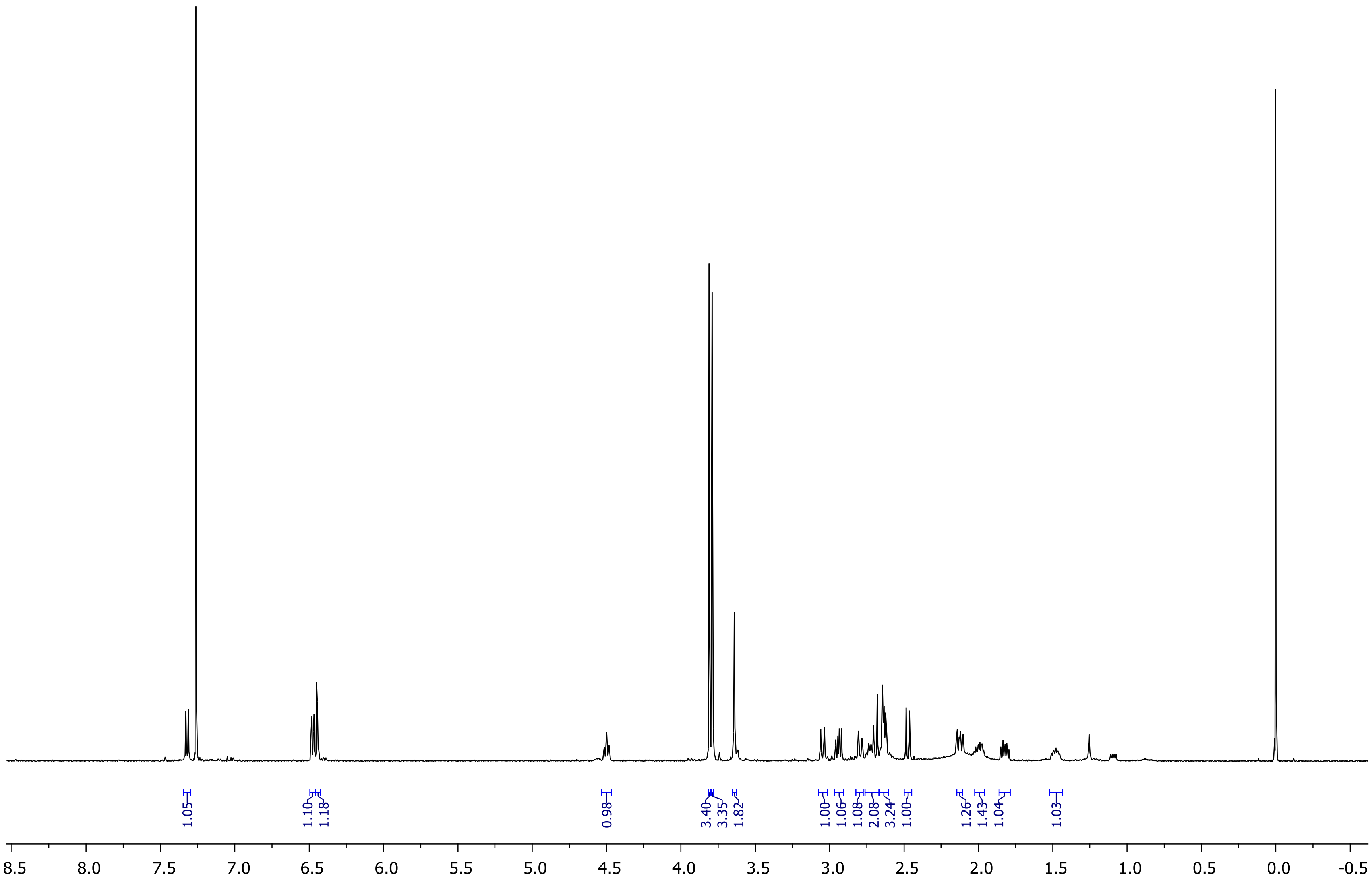

1.05
1.10
1.18
0.98
3.40
3.35
1.82
1.00
1.06
1.08
2.08
3.24
1.00
1.26
1.43
1.04
1.03
8.5
8.0
7.5
7.0
6.5
6.0
5.5
5.0
4.5
4.0
3.5
3.0
2.5
2.0
1.5
1.0
0.5
0.0
-0.5

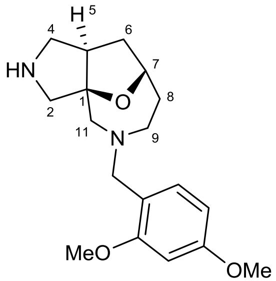
H
5
4
6
HN
7
O
8
2
1
11
N
9
MeO
OMe

S41

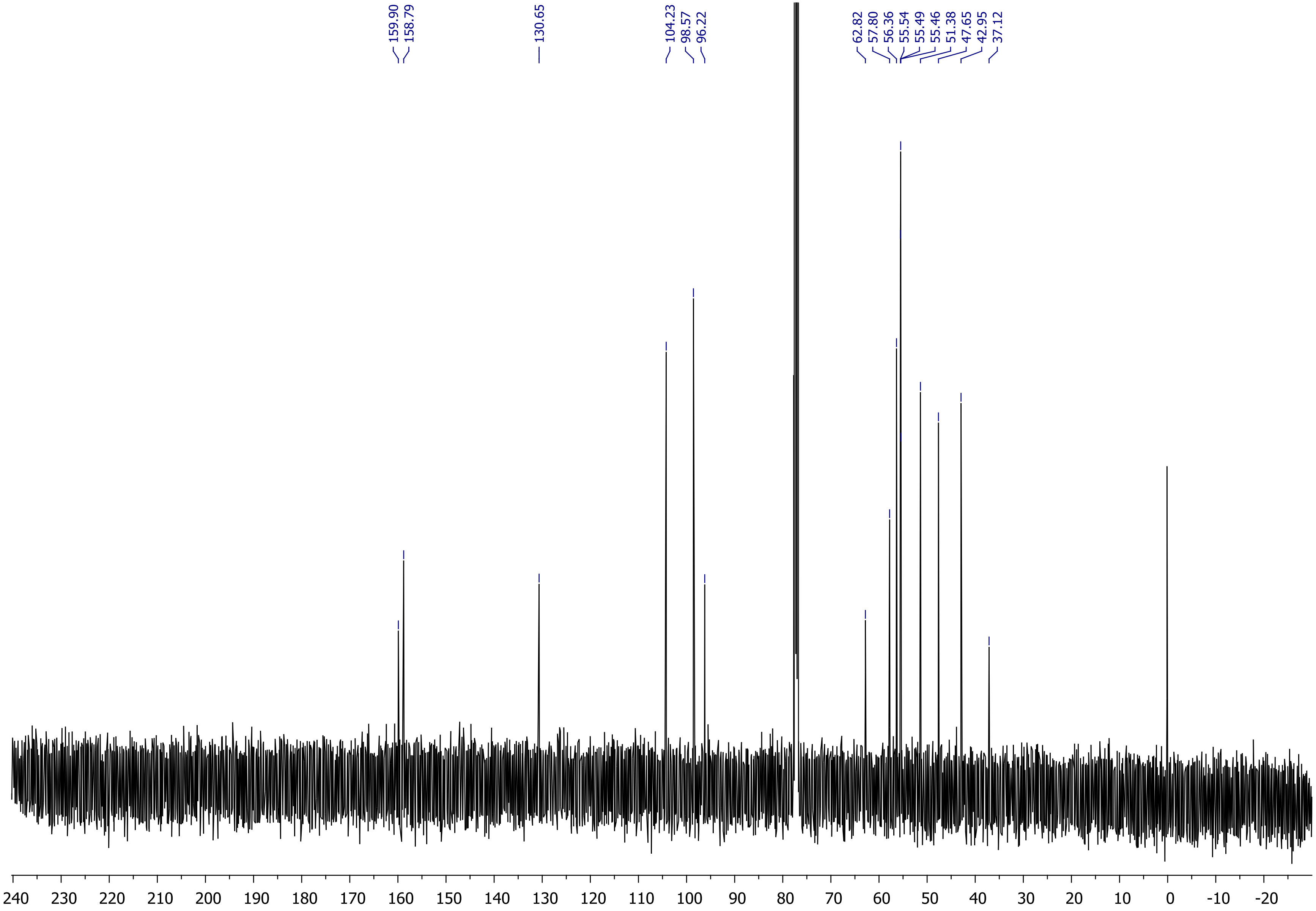
159.90
158.79
130.65
104.23
98.57
96.22
62.82
57.80
56.36
55.54
55.49
55.46
51.38
47.65
42.95
37.12
240 230 220 210 200 190 180 170 160 150 140 130 120 110 100 90 80 70 60 50 40 30 20 10 0 -10 -20

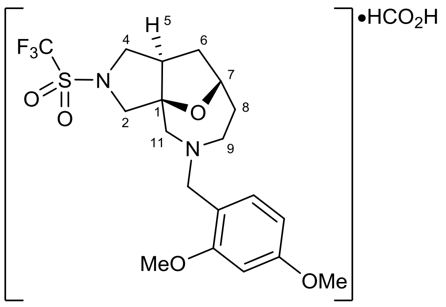
•HCO2H
F3C
O
O
S
N
H 5
4
6
7
8
O
1
2
11
N
9
MeO
OMe

S42

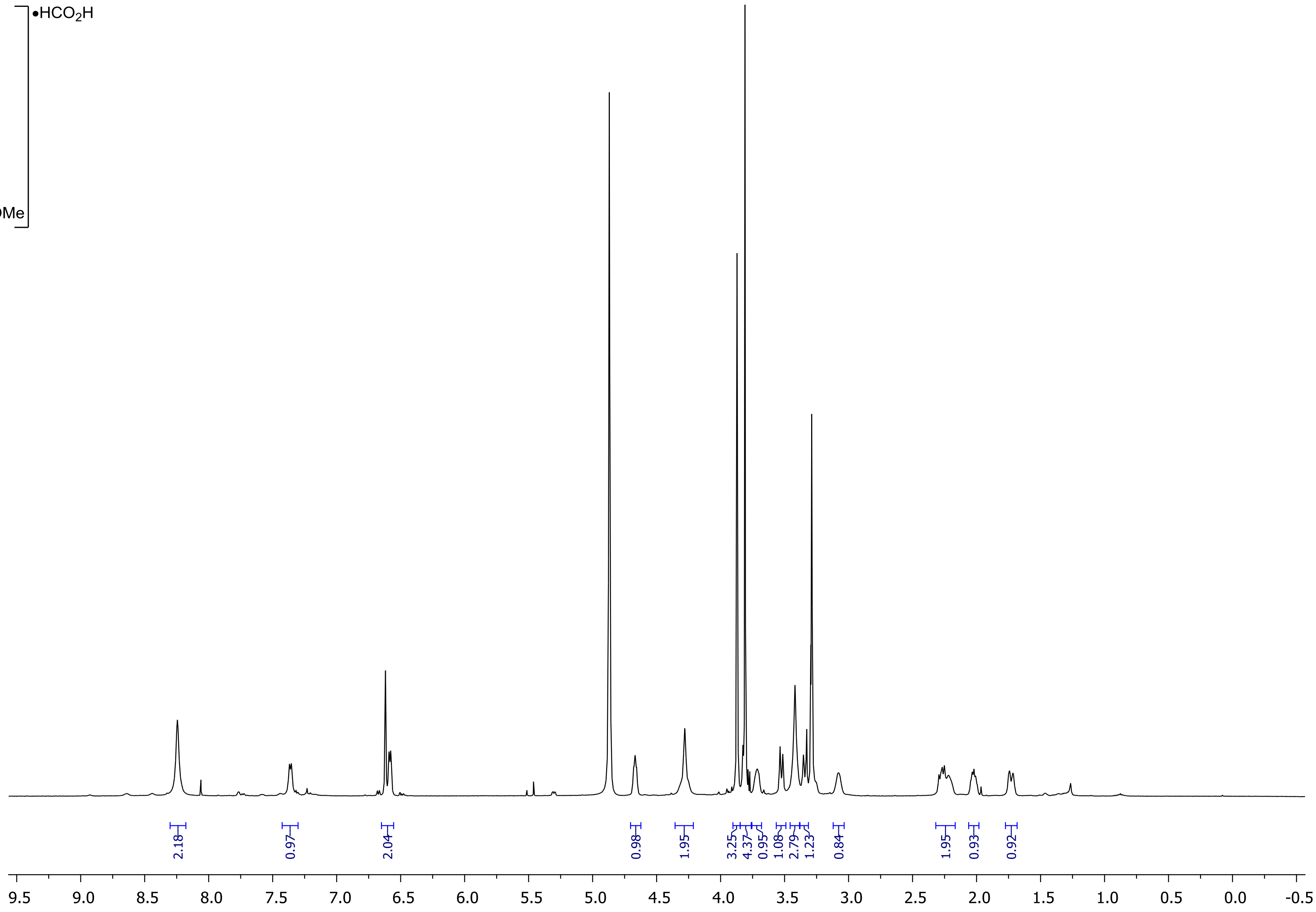
2.18
0.97
2.04
0.98
1.95
3.25
4.37
0.95
1.08
2.79
1.23
0.84
1.95
0.93
0.92
9.5
9.0
8.5
8.0
7.5
7.0
6.5
6.0
5.5
5.0
4.5
4.0
3.5
3.0
2.5
2.0
1.5
1.0
0.5
0.0
-0.5

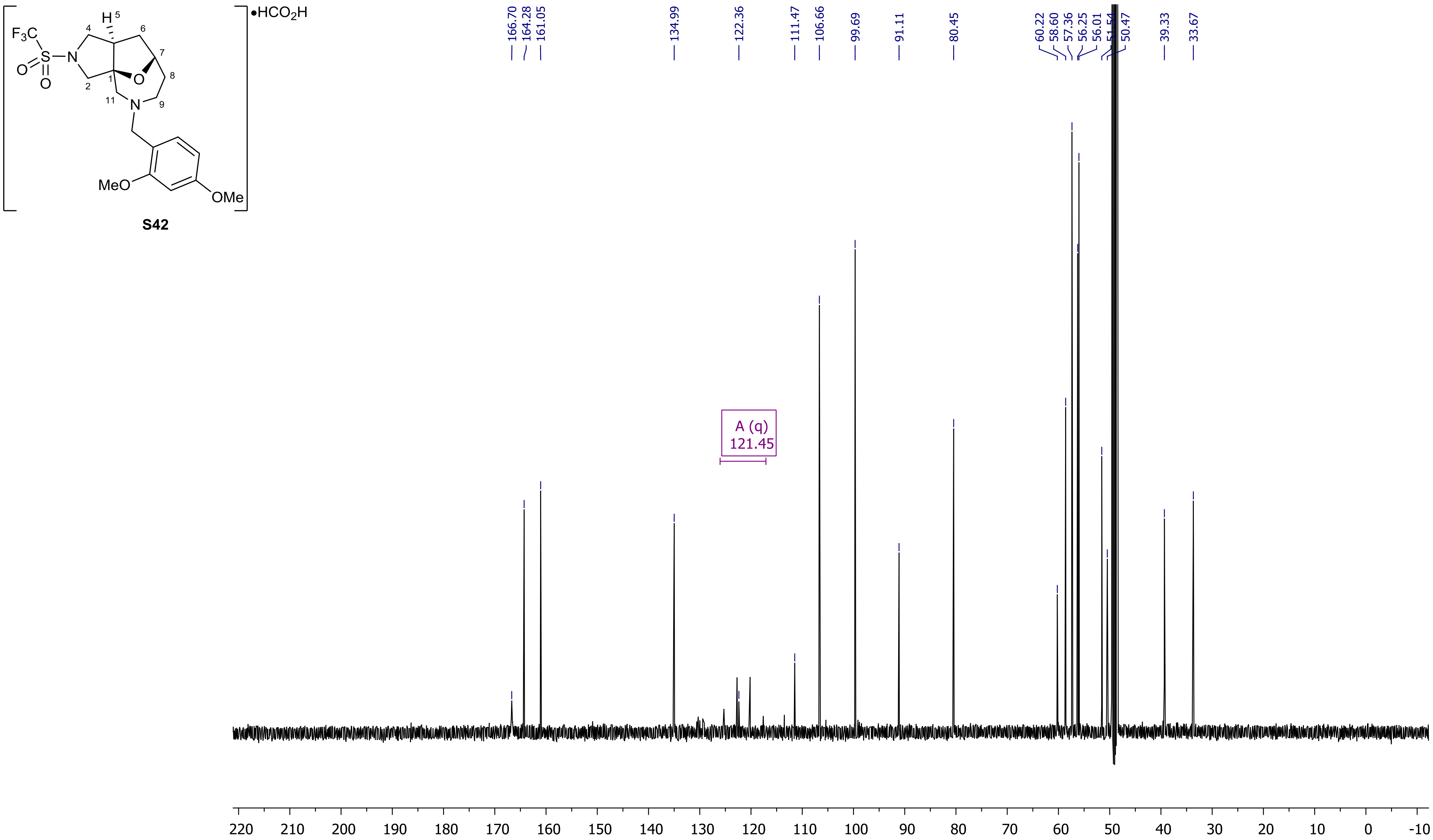
•HCO2H
S42
166.70
164.28
161.05
134.99
122.36
111.47
106.66
99.69
91.11
80.45
60.22
58.60
57.36
56.25
56.01
51.54
50.47
39.33
33.67
A (q)
121.45
220 210 200 190 180 170 160 150 140 130 120 110 100 90 80 70 60 50 40 30 20 10 0 -10

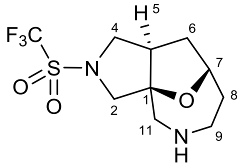
F3C
S
O
O
N
H 5
4
6
7
8
2
1
O
11
N
H
9

F17

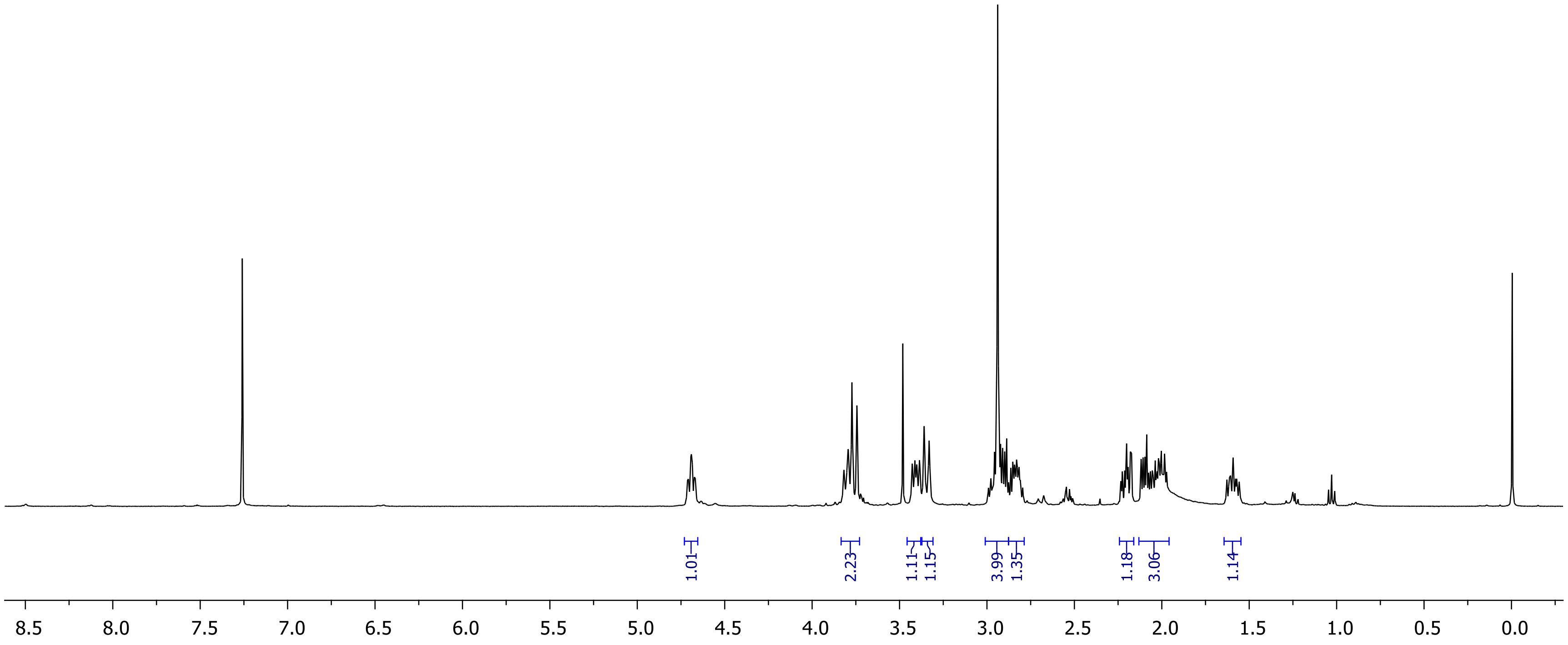
1.01
2.23
1.11
1.15
3.99
1.35
1.18
3.06
1.14
8.5
8.0
7.5
7.0
6.5
6.0
5.5
5.0
4.5
4.0
3.5
3.0
2.5
2.0
1.5
1.0
0.5
0.0

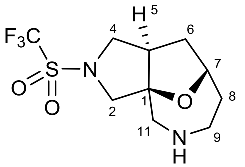

F17

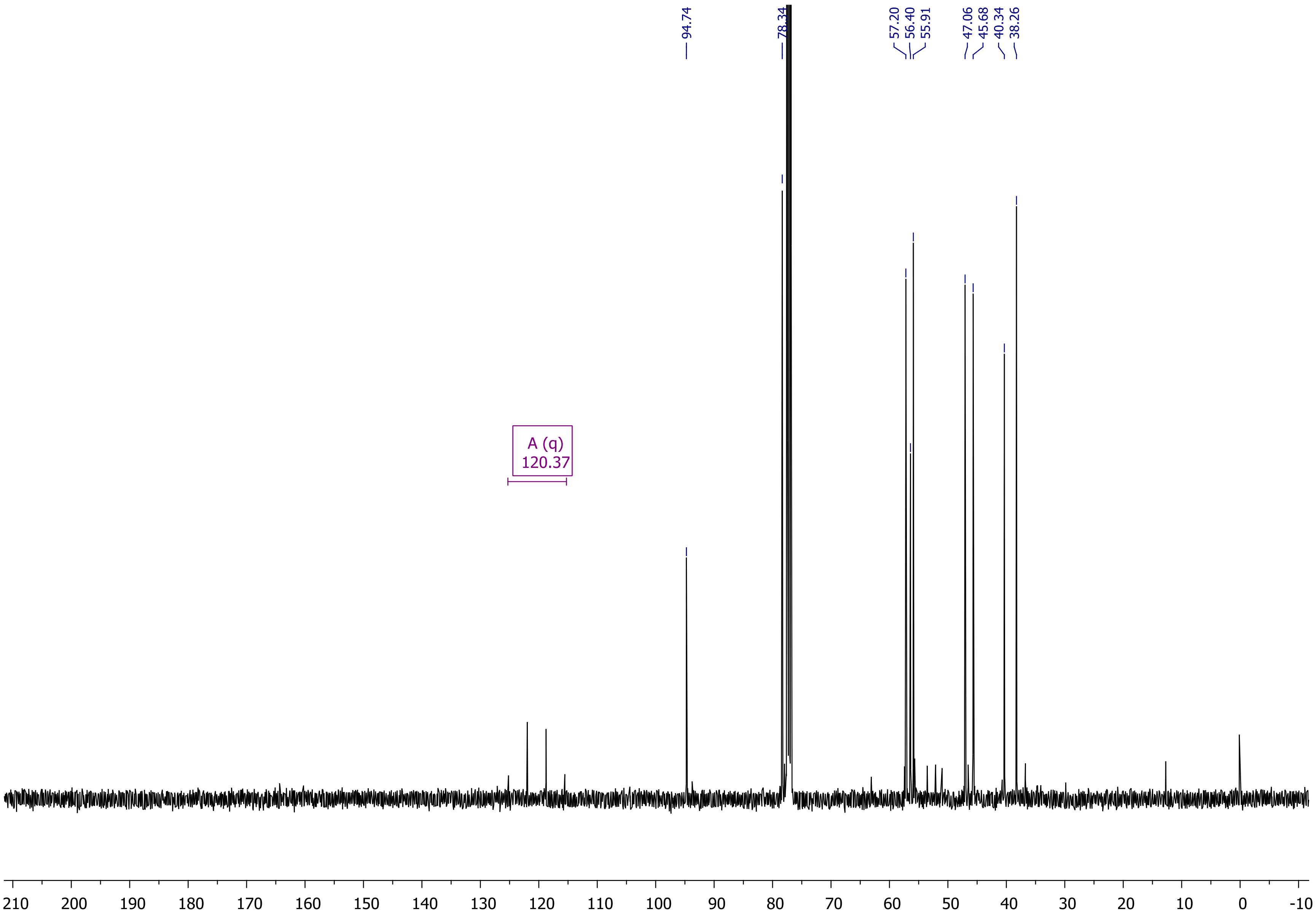

94.74
78.34
57.20
56.40
55.91
47.06
45.68
40.34
38.26
A (q)
120.37
210
200
190
180
170
160
150
140
130
120
110
100
90
80
70
60
50
40
30
20
10
0
-10

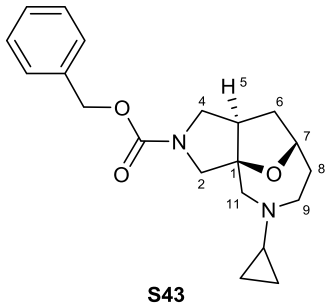

H 5
4
6
7
8
9
11
2
1
O
N
O
O
N

S43

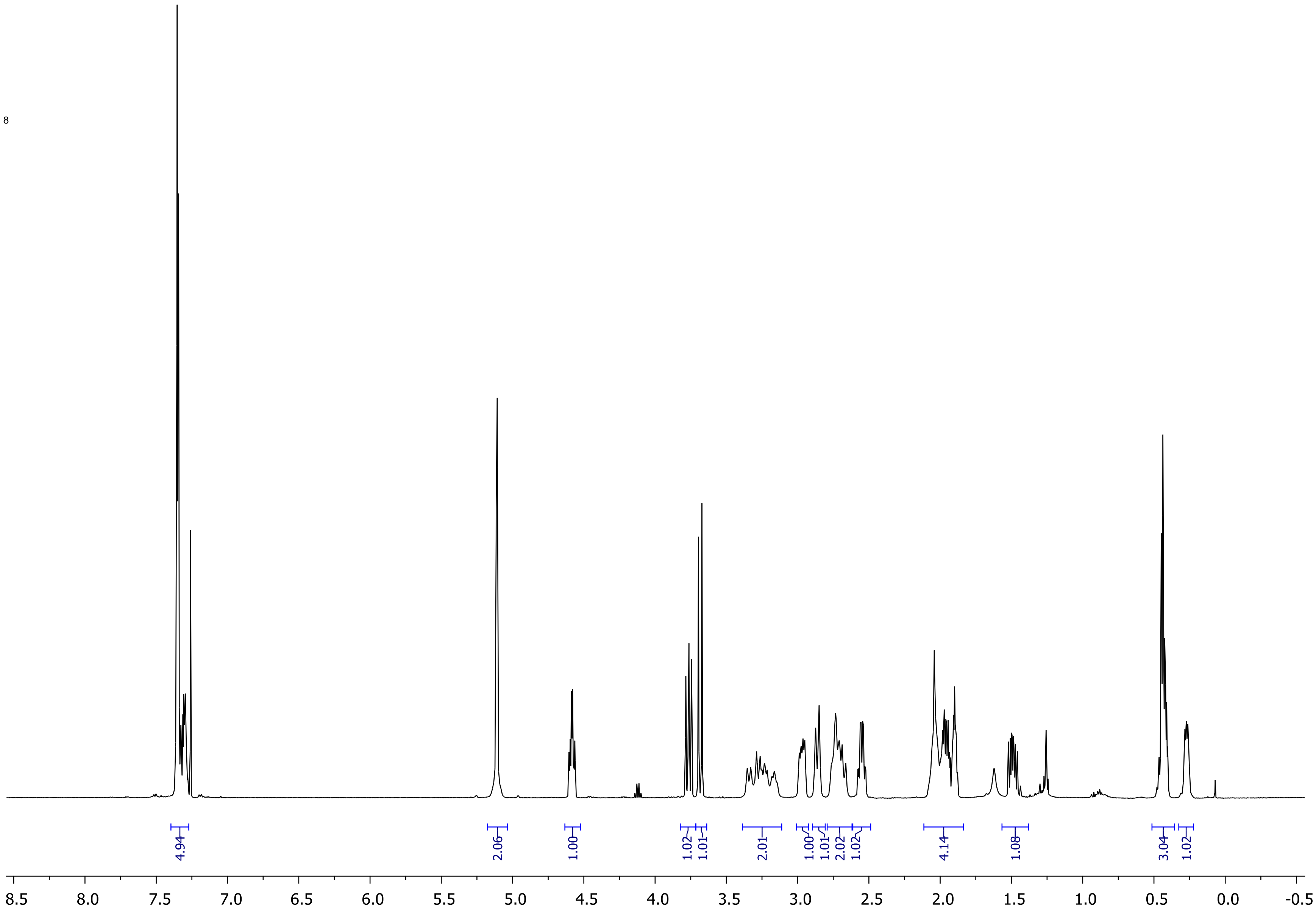

4.94
2.06
1.00
1.02
1.01
2.01
1.00
1.01
2.02
1.02
4.14
1.08
3.04
1.02
8.5
8.0
7.5
7.0
6.5
6.0
5.5
5.0
4.5
4.0
3.5
3.0
2.5
2.0
1.5
1.0
0.5
0.0
-0.5

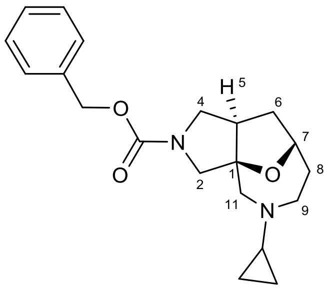

S43
($CDCl_3$ @ 329 K)

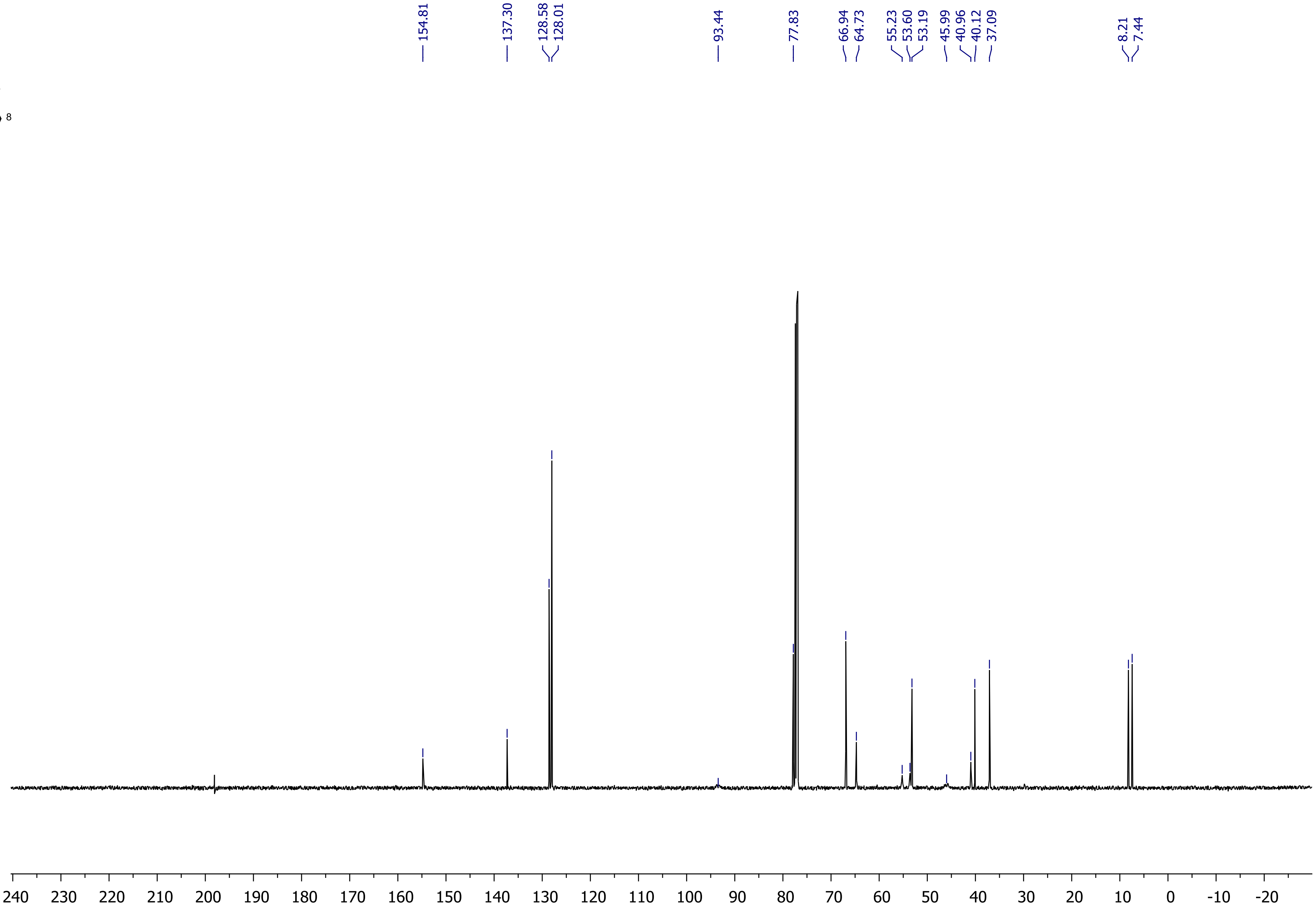

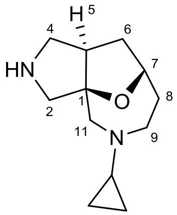

HN
H 5
4
6
7
8
O
1
2
11
N
9

F18

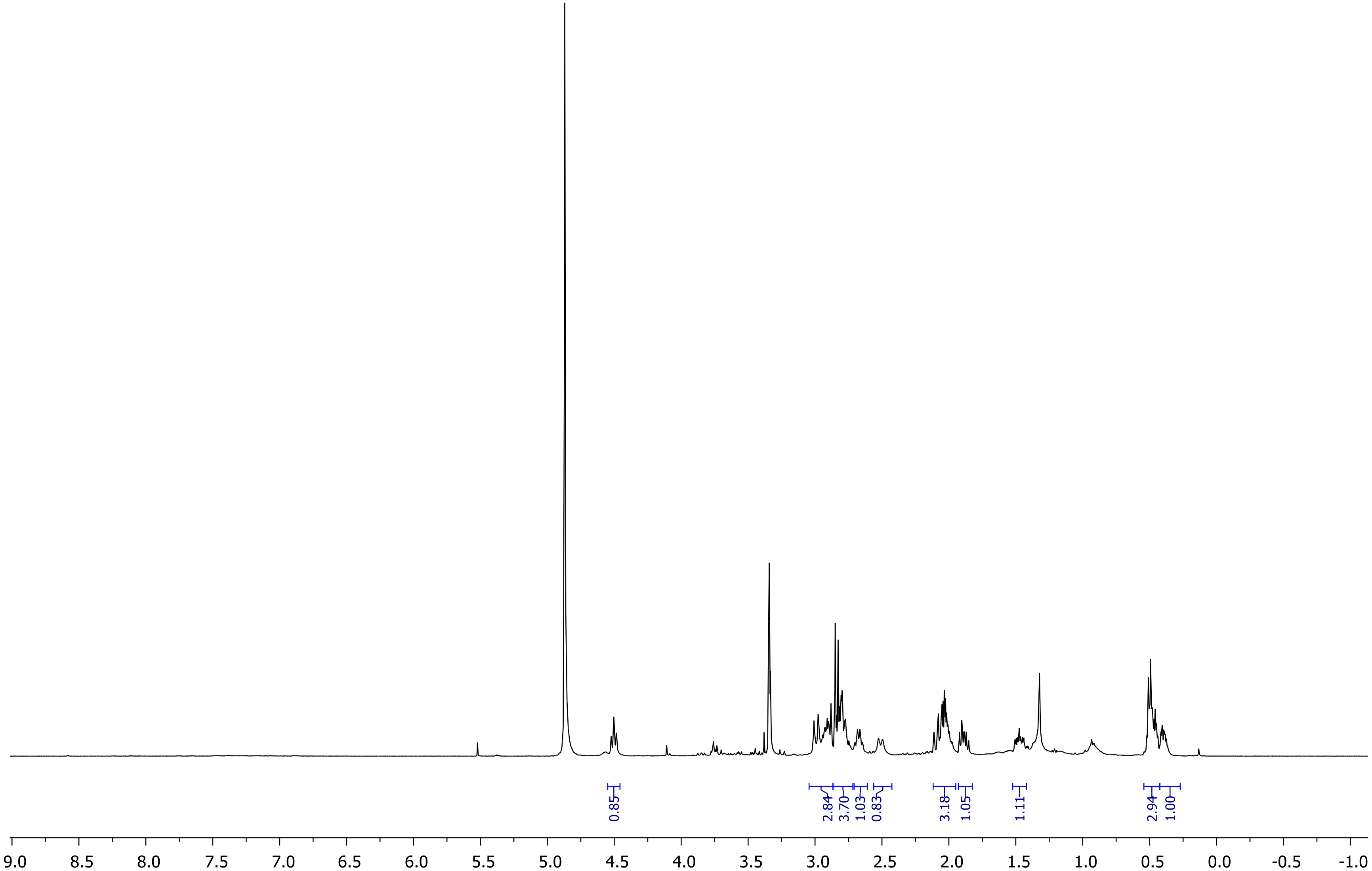

0.85
2.84
3.70
1.03
0.83
3.18
1.05
1.11
2.94
1.00
9.0 8.5 8.0 7.5 7.0 6.5 6.0 5.5 5.0 4.5 4.0 3.5 3.0 2.5 2.0 1.5 1.0 0.5 0.0 -0.5 -1.0

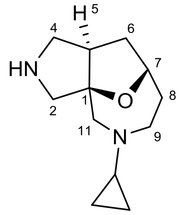
H
5
4
6
7
HN
O
8
1
2
11
N
9

F18

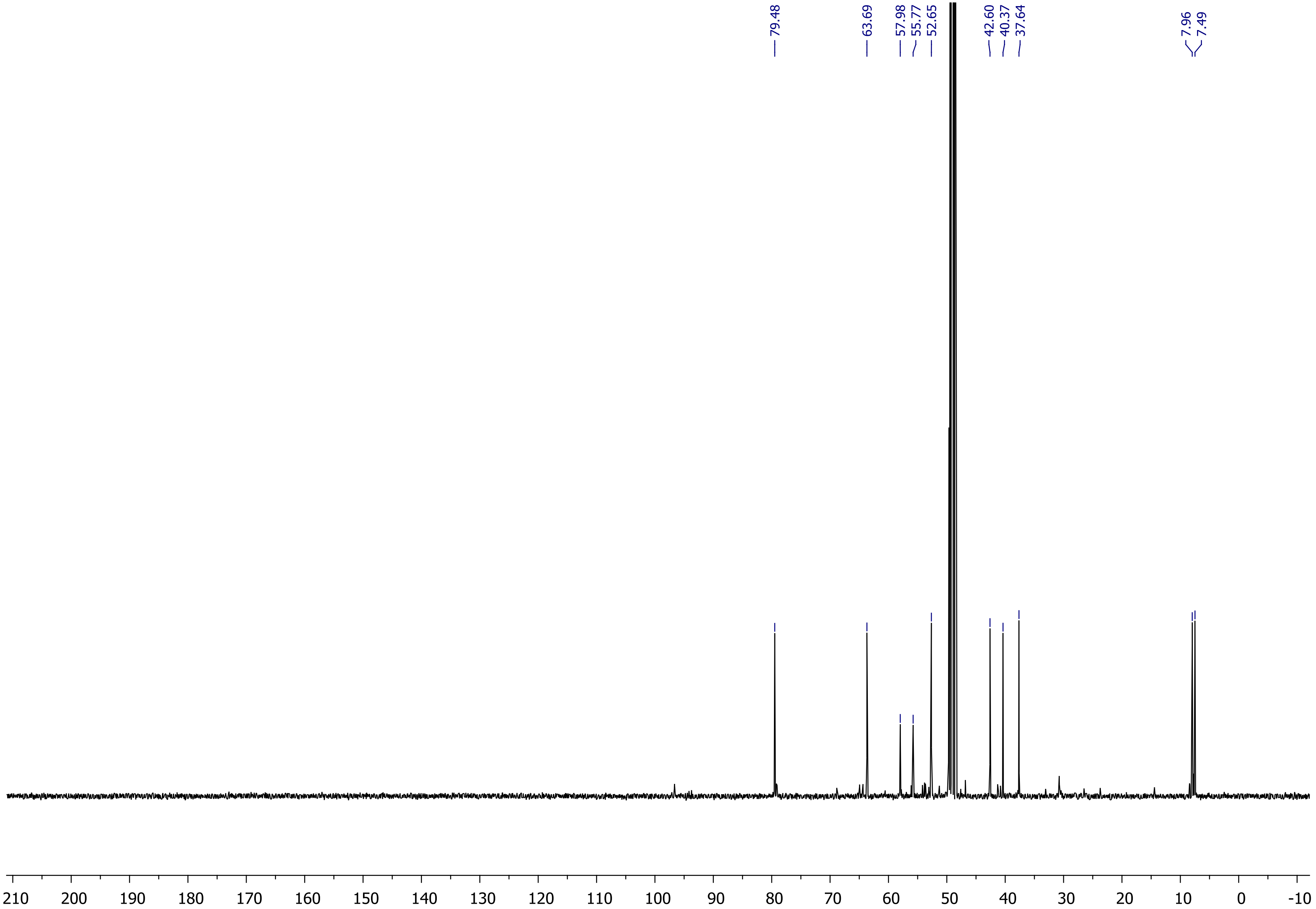
79.48
63.69
57.98
55.77
52.65
42.60
40.37
37.64
7.96
7.49
210
200
190
180
170
160
150
140
130
120
110
100
90
80
70
60
50
40
30
20
10
0
-10

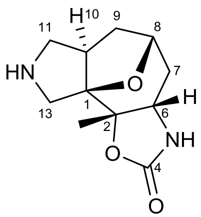

F19

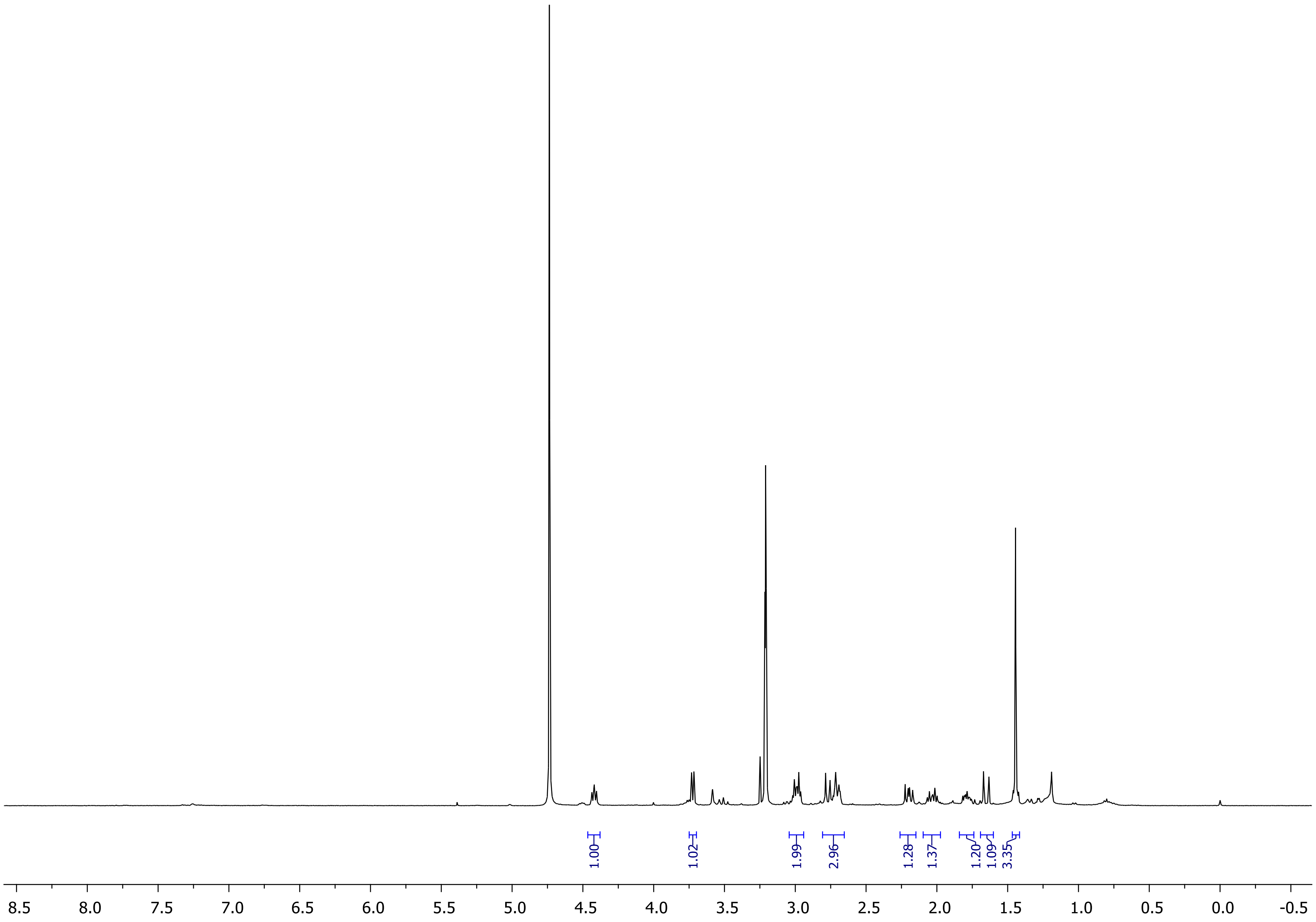

1.00
1.02
1.99
2.96
1.28
1.37
1.20
1.09
3.35
8.5
8.0
7.5
7.0
6.5
6.0
5.5
5.0
4.5
4.0
3.5
3.0
2.5
2.0
1.5
1.0
0.5
0.0
-0.5

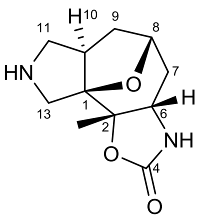

HN
H
O
H
NH
O
O
11
10
9
8
7
13
1
2
6
4

F19

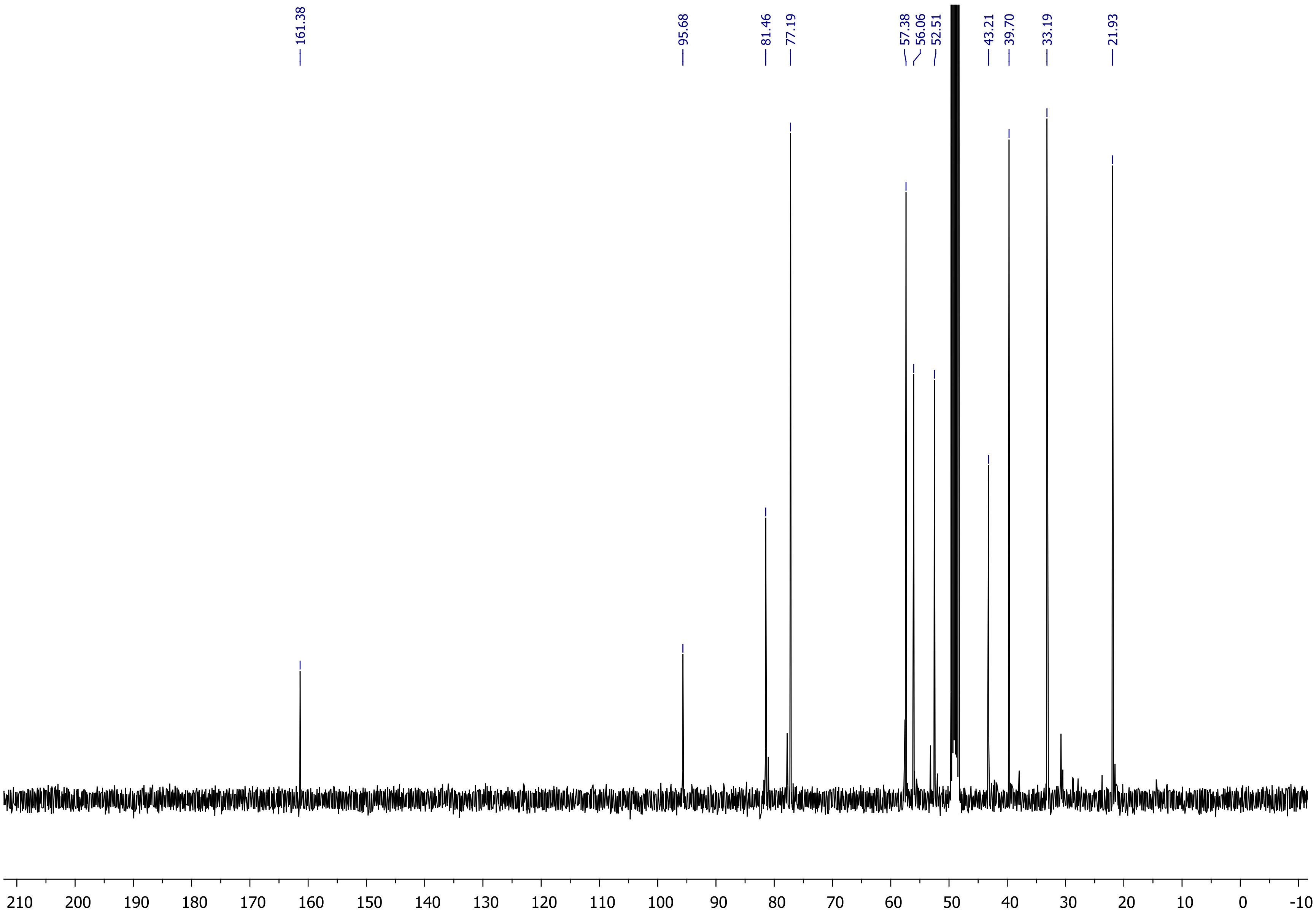

161.38
95.68
81.46
77.19
57.38
56.06
52.51
43.21
39.70
33.19
21.93
210 200 190 180 170 160 150 140 130 120 110 100 90 80 70 60 50 40 30 20 10 0 -10

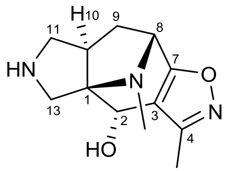

H10
9
8
11
7
O
HN
N
N
1
3
13
2
HO
4

F20

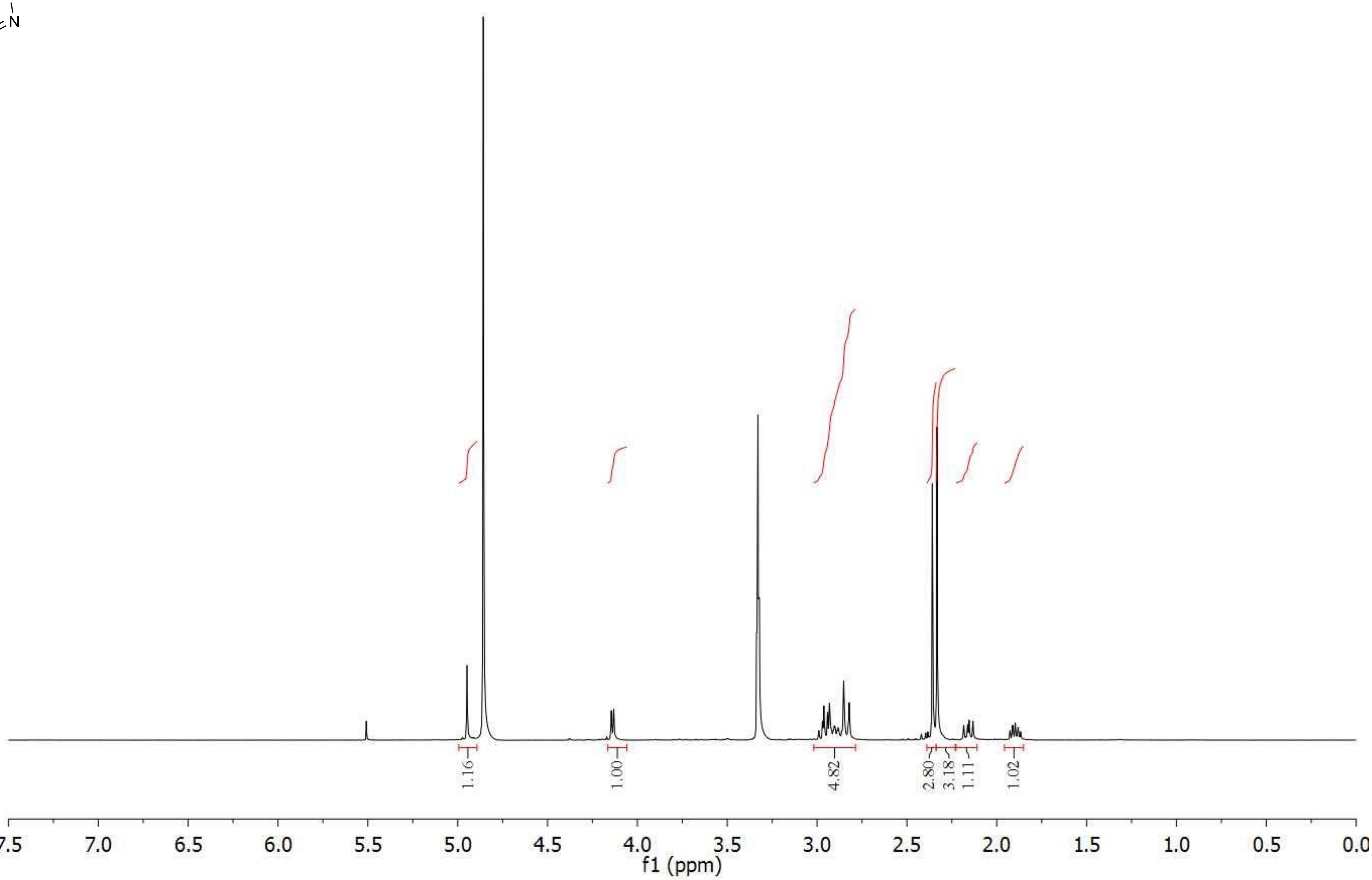

1.16
1.00
4.82
2.80
3.18
1.11
1.02
7.5
7.0
6.5
6.0
5.5
5.0
4.5
4.0
3.5
3.0
2.5
2.0
1.5
1.0
0.5
0.0
f1 (ppm)

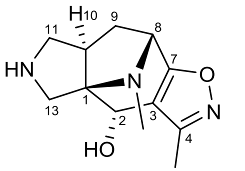
H10
9
8
11
HN
N
7
O
N
13
1
2
3
4
HO

F20

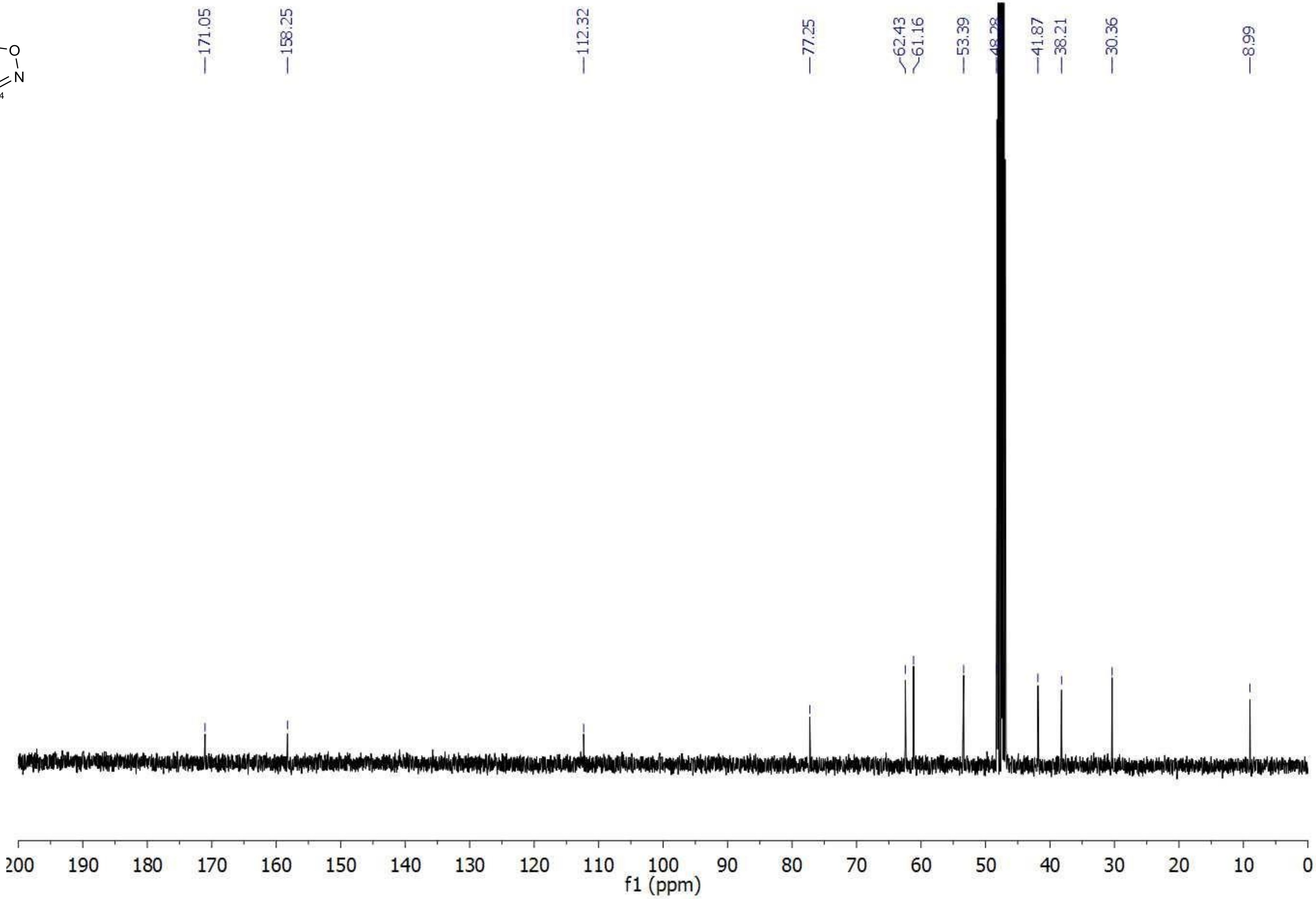
171.05
158.25
112.32
77.25
62.43
61.16
53.39
48.28
41.87
38.21
30.36
8.99
200 190 180 170 160 150 140 130 120 110 100 90 80 70 60 50 40 30 20 10 0
f1 (ppm)

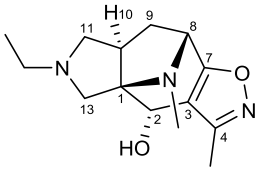

F21

(MeOD-d_4 @ 333 K)

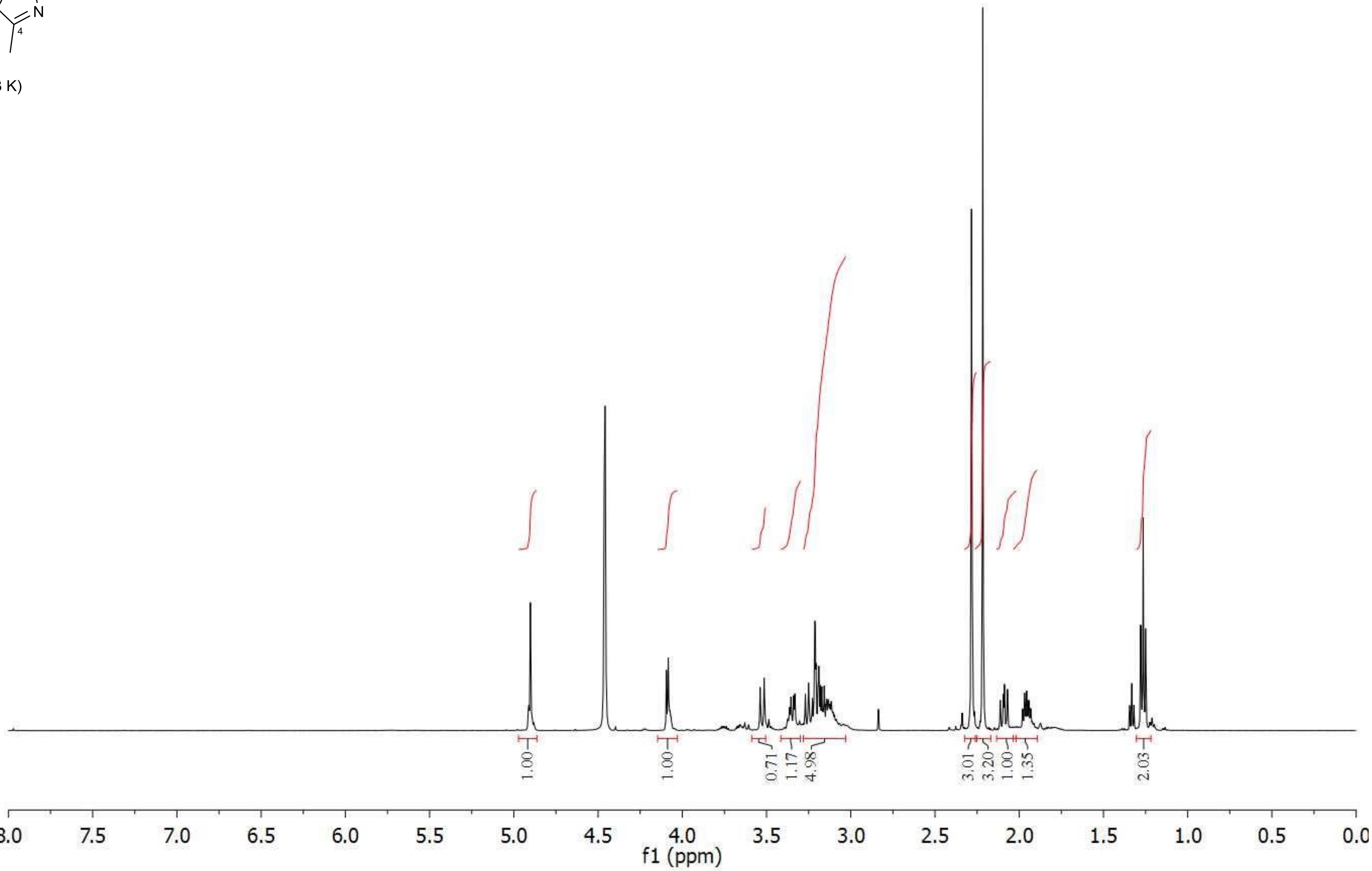

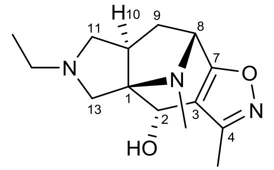

F21
(MeOD-d_4 @ 333 K)

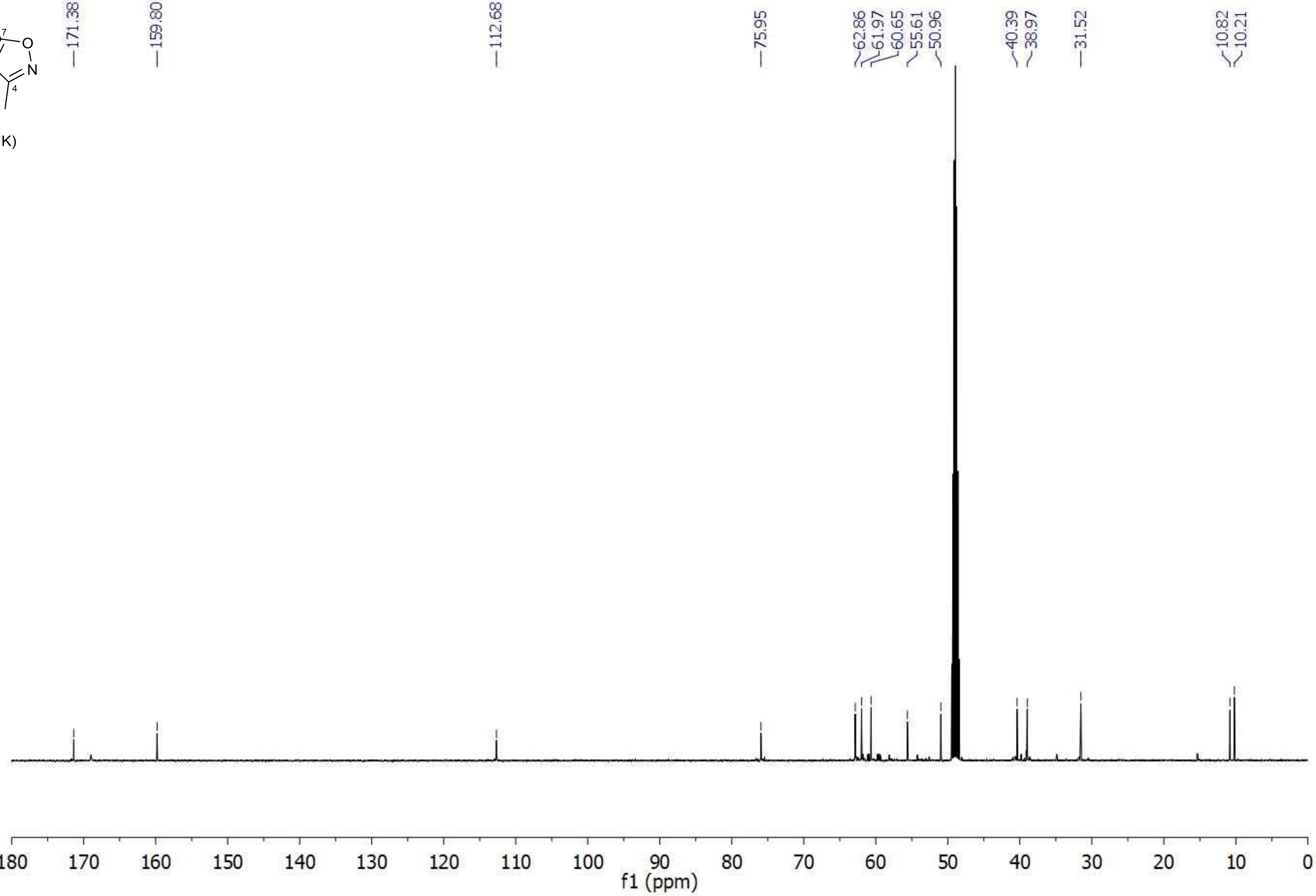

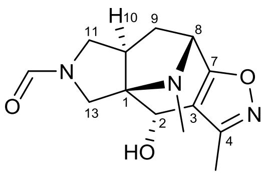

F22

(MeOD-d_4 @ 333 K)

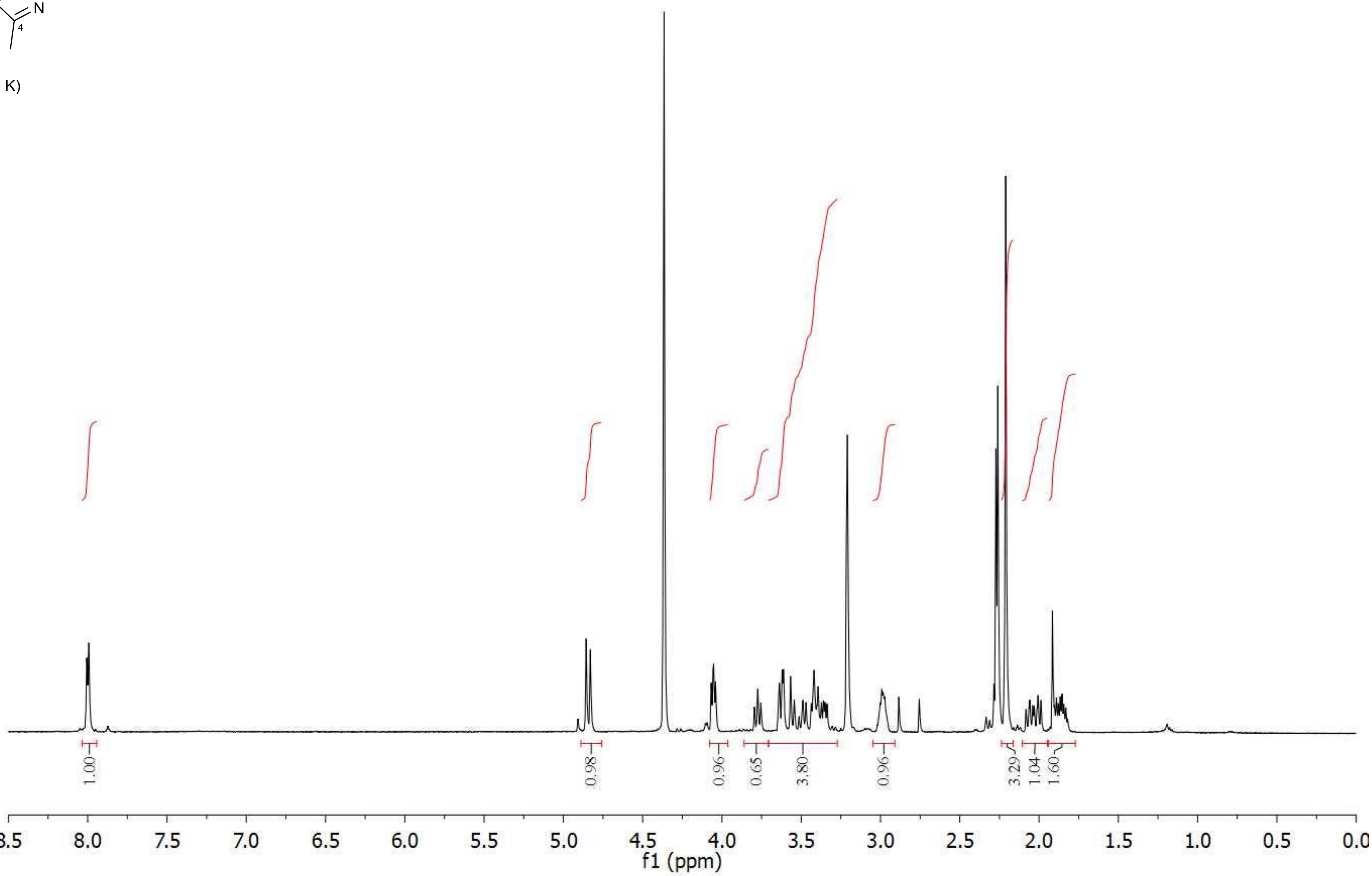

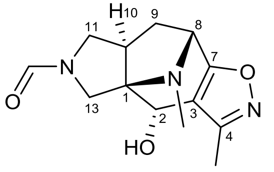

F22
(MeOD-d_4 @ 333 K)

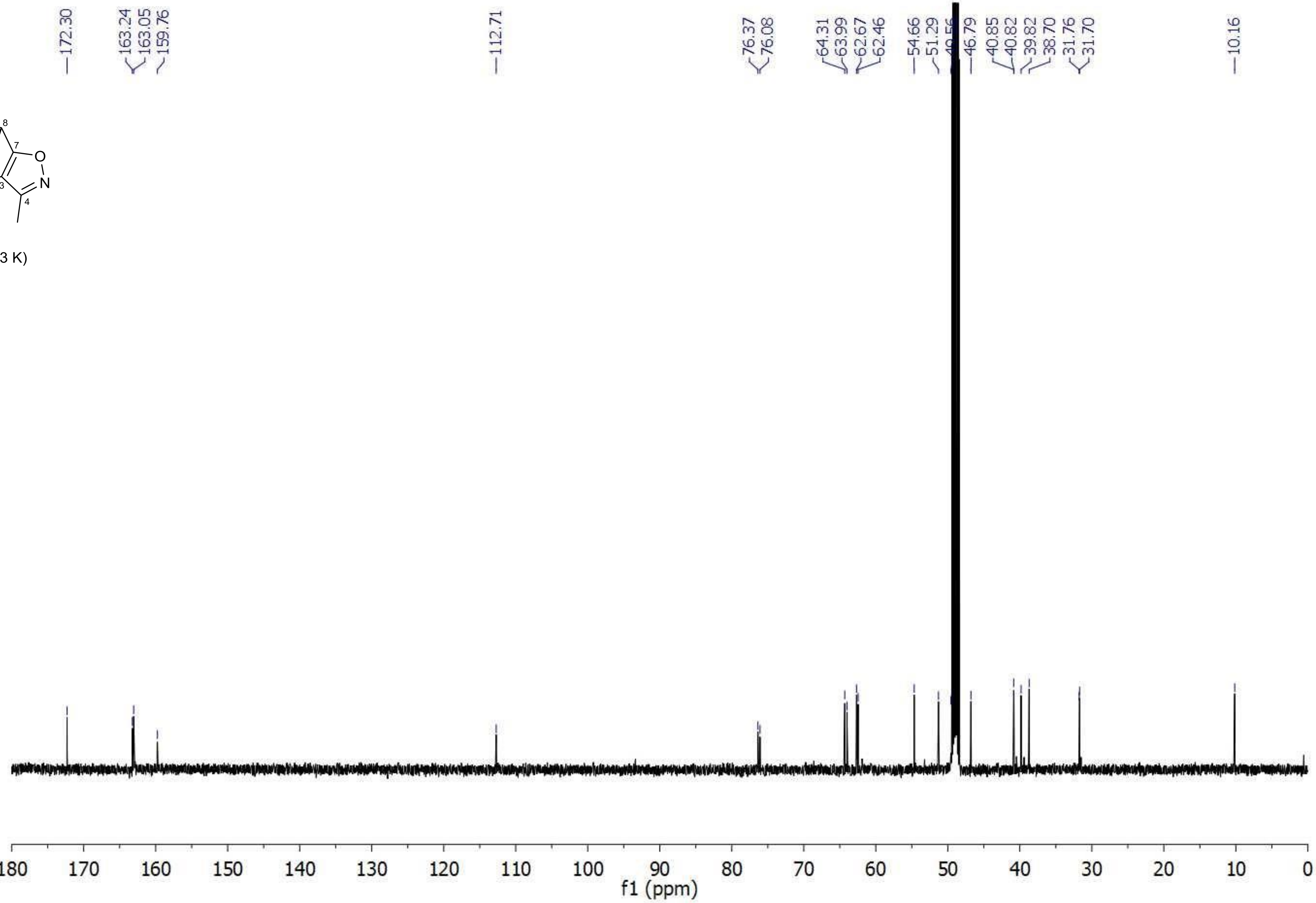

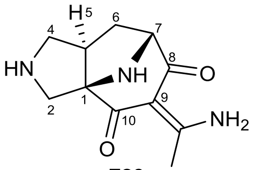
HN
NH
NH2
O
O
H5
1
2
4
6
7
8
9
10

F23

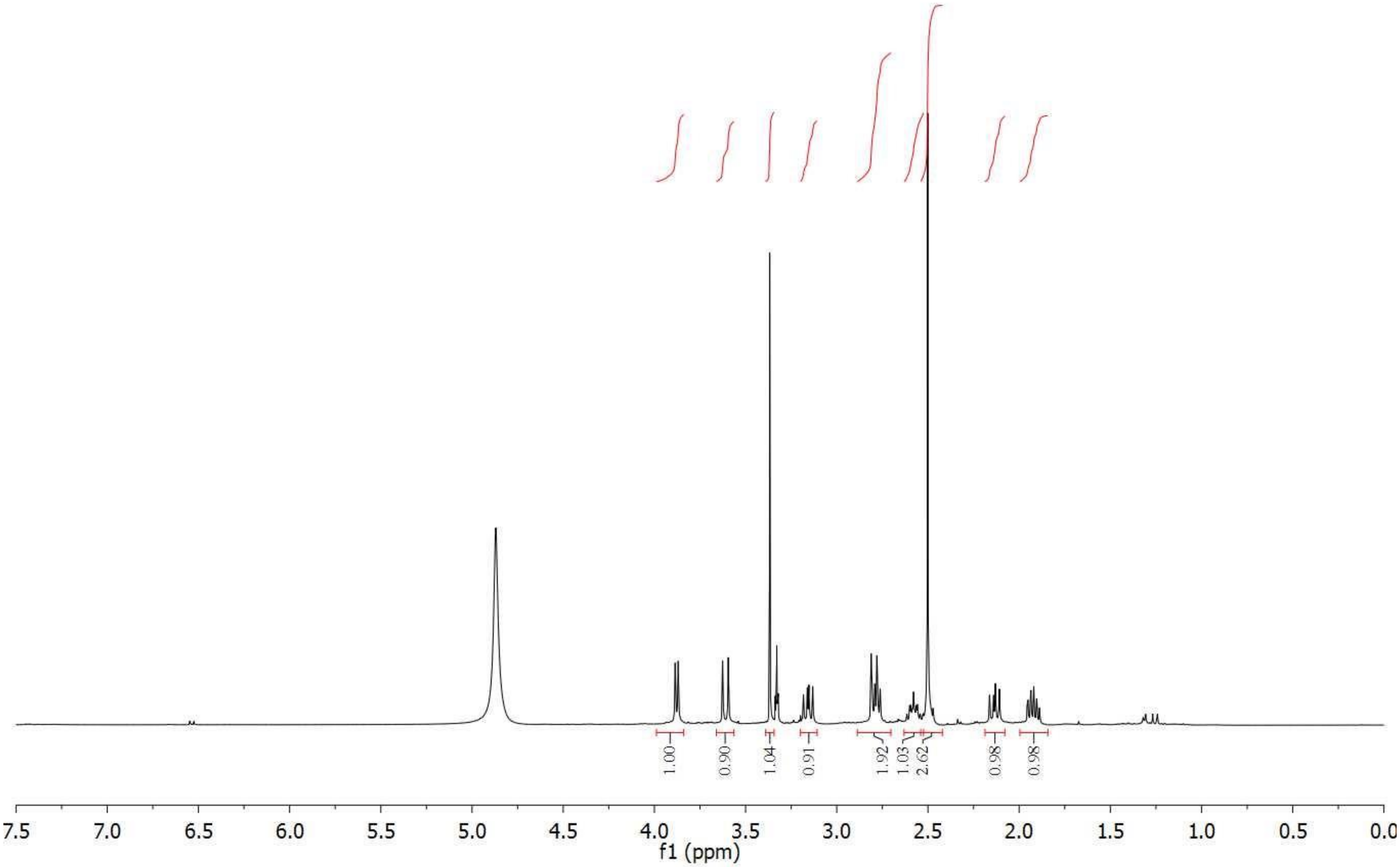
1.00
0.90
1.04
0.91
1.92
1.03
2.62
0.98
0.98
7.5
7.0
6.5
6.0
5.5
5.0
4.5
4.0
3.5
3.0
2.5
2.0
1.5
1.0
0.5
0.0
f1 (ppm)

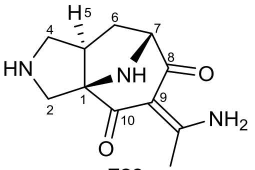
HN
NH
O
NH2
H 5
1
2
4
6
7
8
9
10

F23

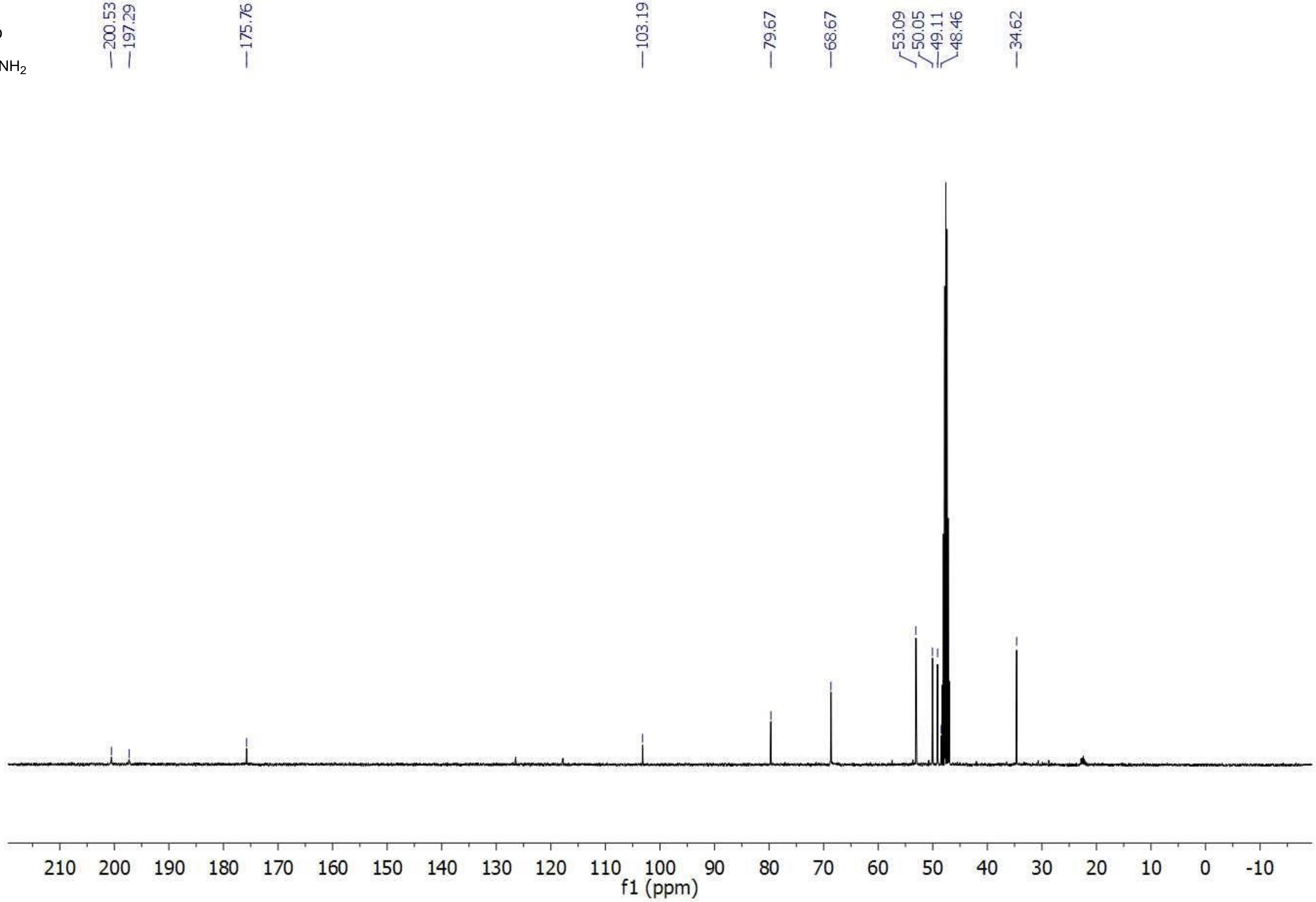
200.53
197.29
175.76
103.19
79.67
68.67
53.09
50.05
49.11
48.46
34.62
210 200 190 180 170 160 150 140 130 120 110 100 90 80 70 60 50 40 30 20 10 0 -10
f1 (ppm)

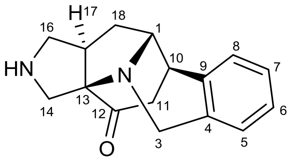
HN
N
O
H17
18
1
16
10
9
8
7
6
5
4
3
11
12
13
14

F24

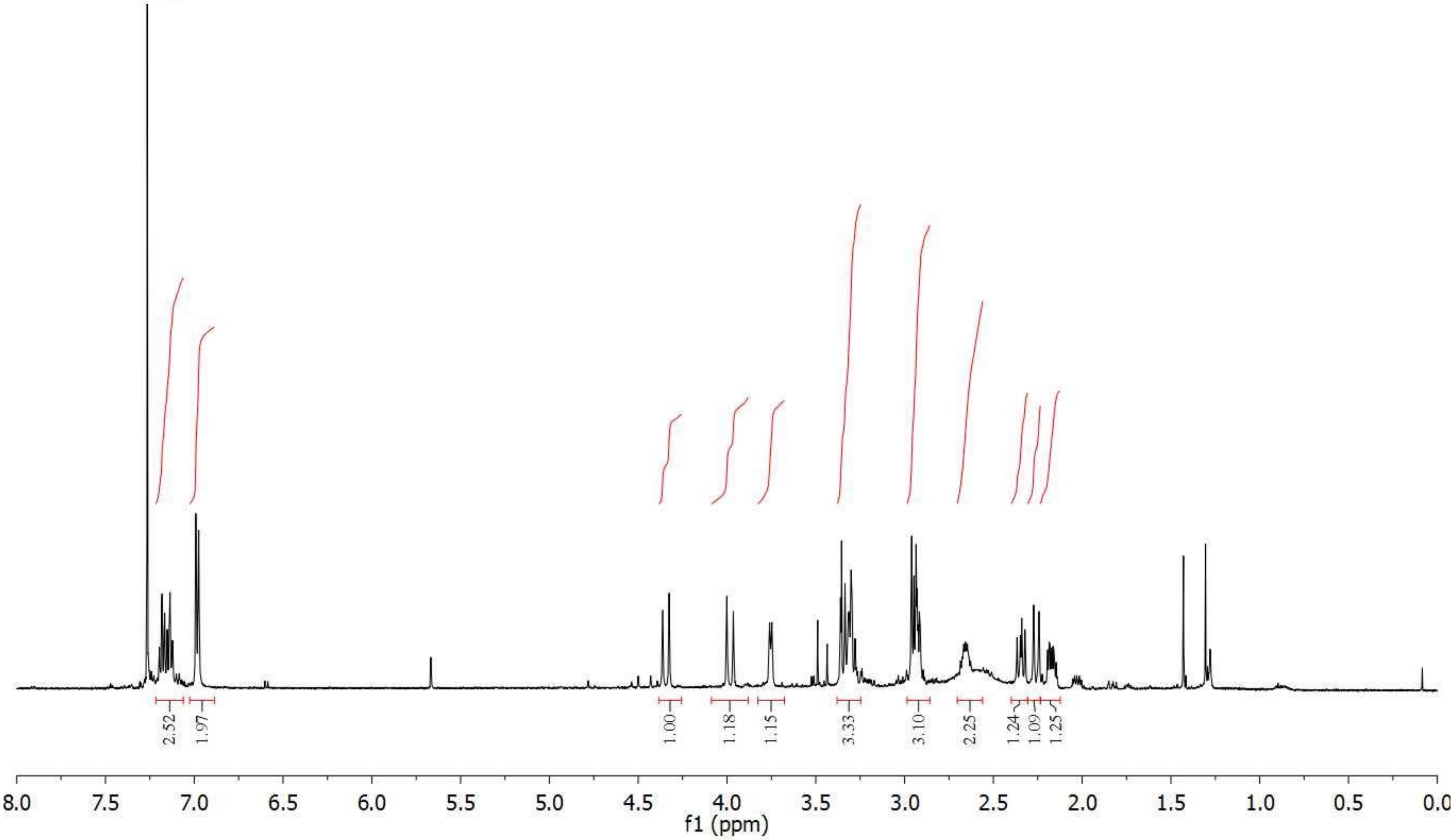
2.52
1.97
1.00
1.18
1.15
3.33
3.10
2.25
1.24
1.09
1.25
8.0
7.5
7.0
6.5
6.0
5.5
5.0
4.5
4.0
3.5
3.0
2.5
2.0
1.5
1.0
0.5
0.0
f1 (ppm)

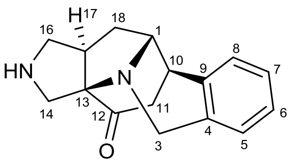
H
17
18
1
16
8
HN
N
10
9
7
13
11
6
12
14
3
4
5
O

F24

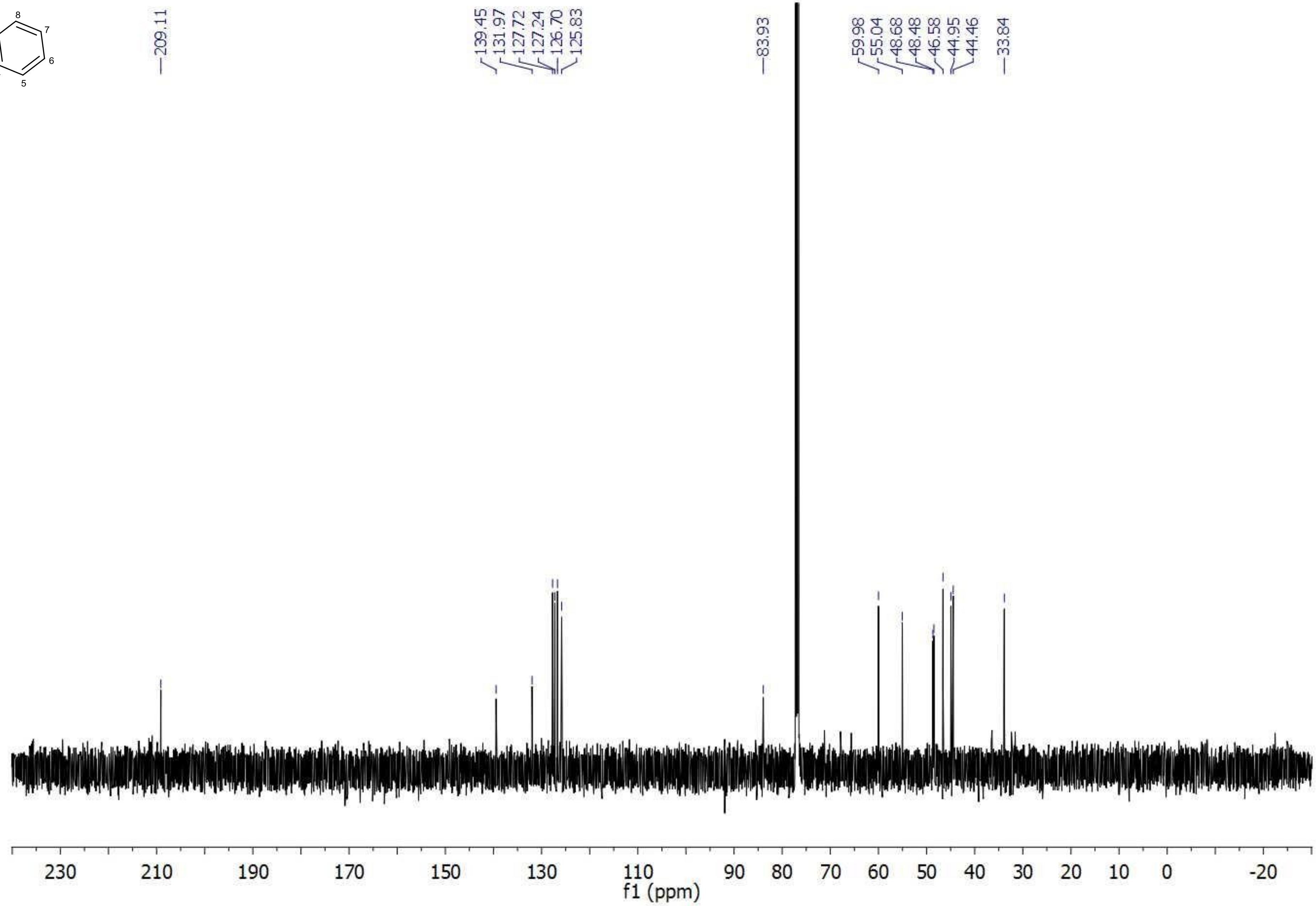
209.11
139.45
131.97
127.72
127.24
126.70
125.83
83.93
59.98
55.04
48.68
48.48
46.58
44.95
44.46
33.84
230
210
190
170
150
130
110
90
80
70
60
50
40
30
20
10
0
-20
f1 (ppm)

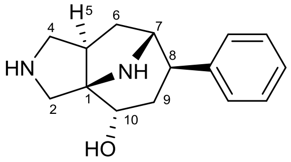

F25

($MeOD-d_4$ @ 333 K)

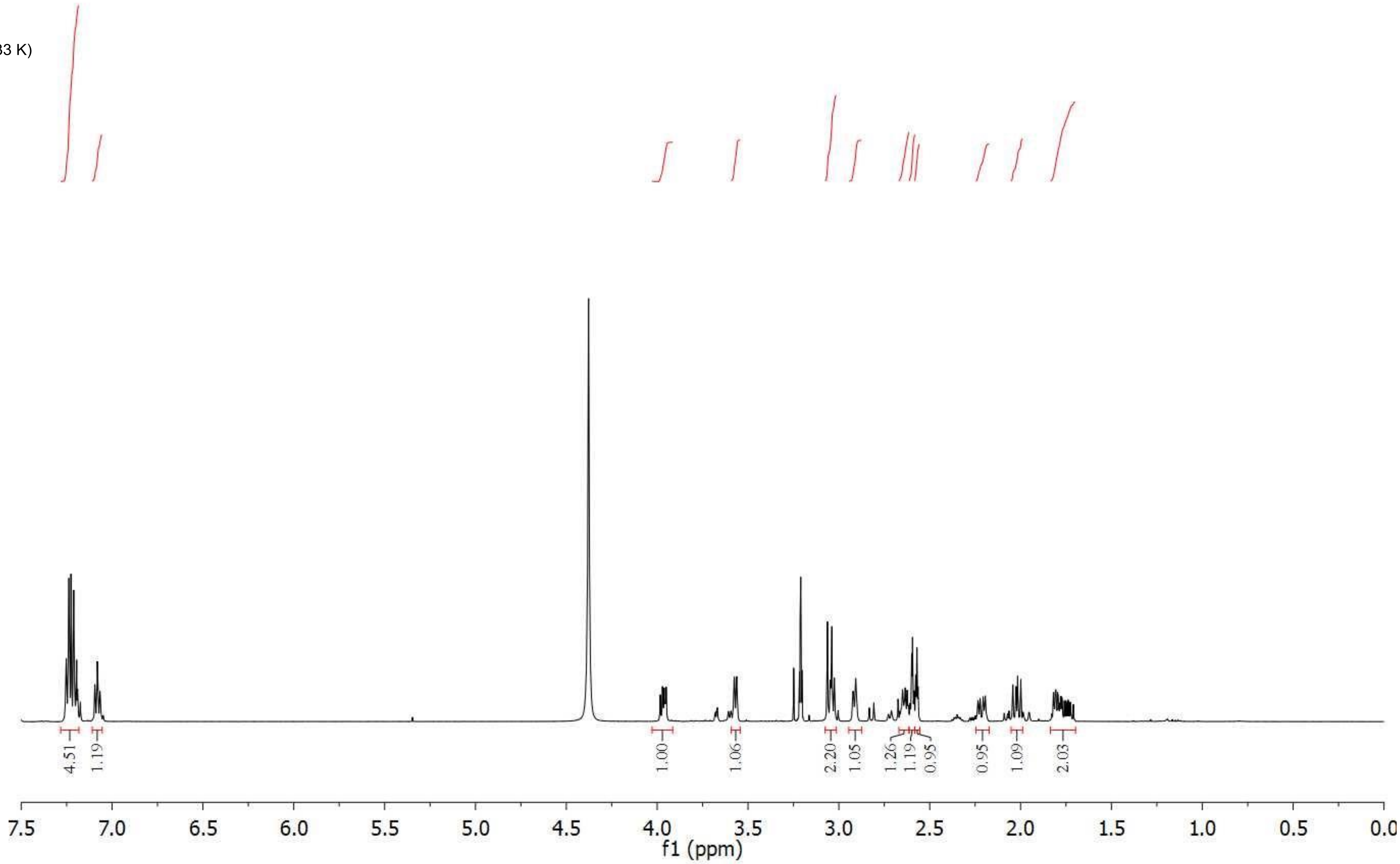

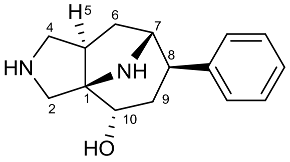

F25

(MeOD-d_4 @ 333 K)

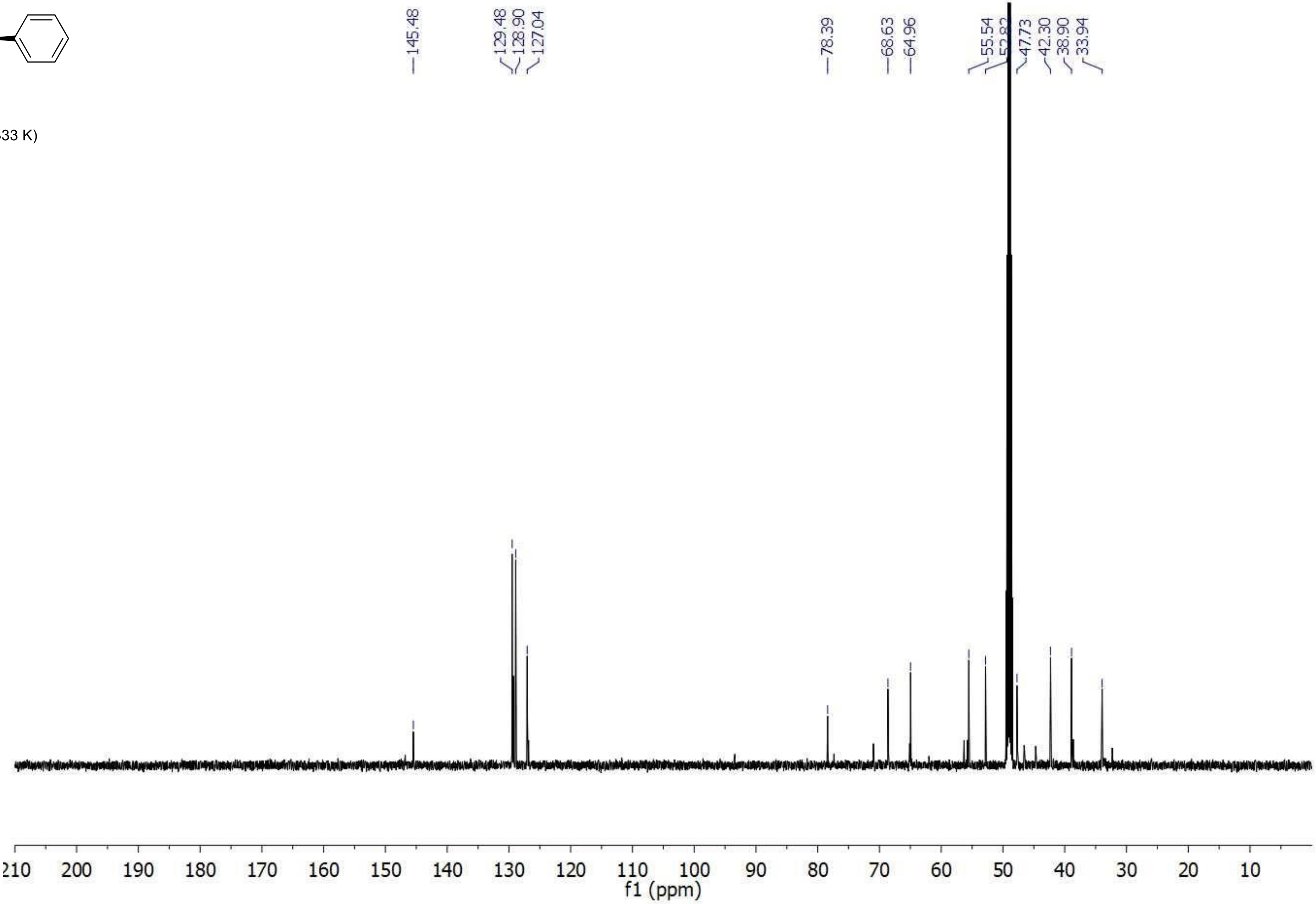

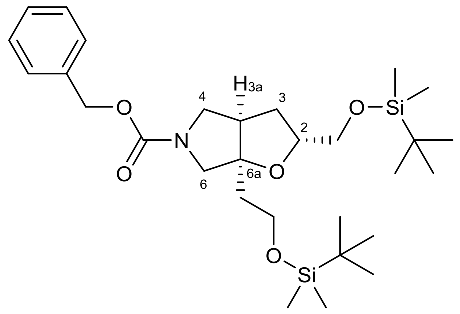
H3a
4
3
2
6
6a
O
N
Si

S44

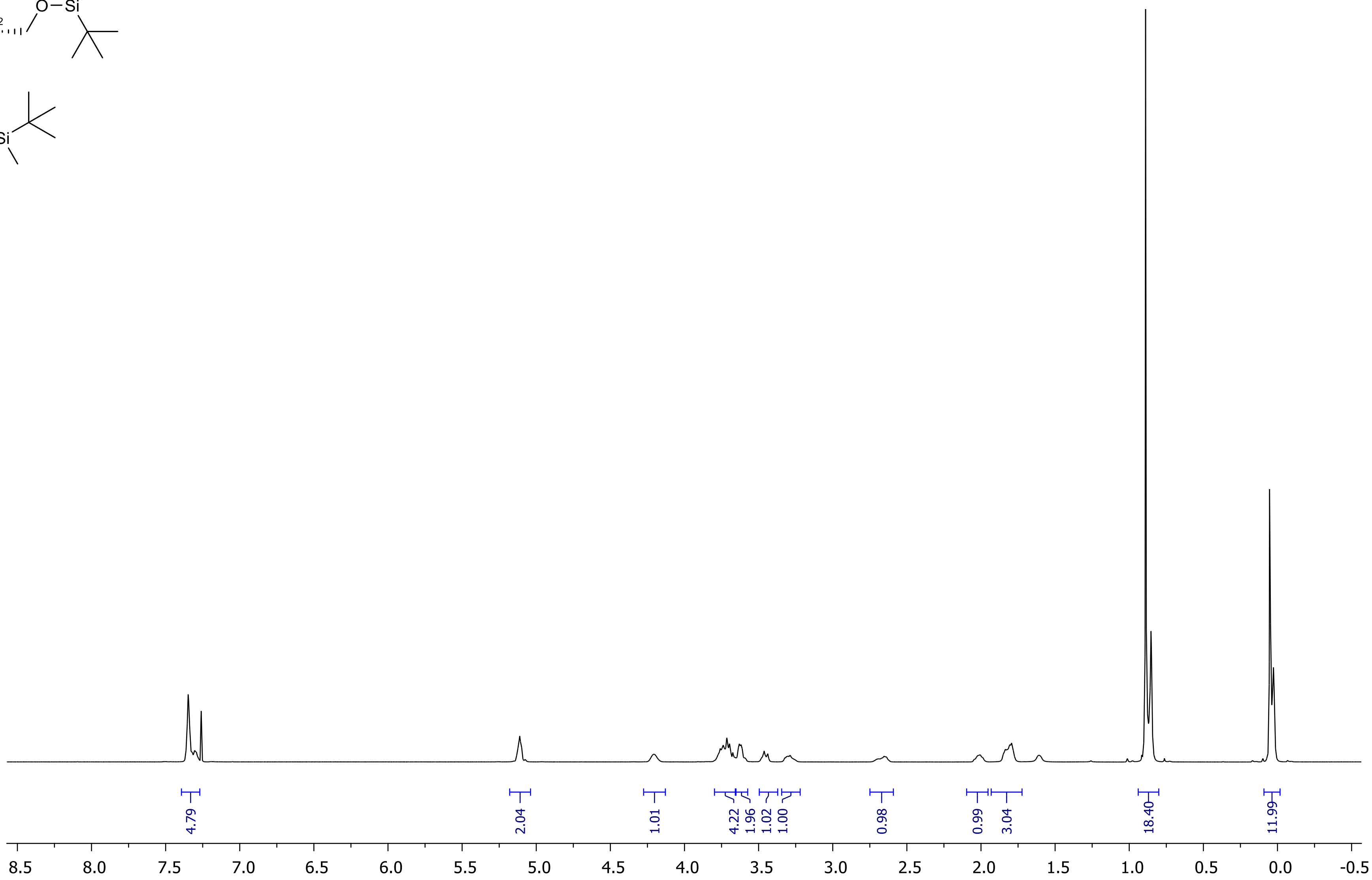
4.79
2.04
1.01
4.22
1.96
1.02
1.00
0.98
0.99
3.04
18.40
11.99
8.5
8.0
7.5
7.0
6.5
6.0
5.5
5.0
4.5
4.0
3.5
3.0
2.5
2.0
1.5
1.0
0.5
0.0
-0.5

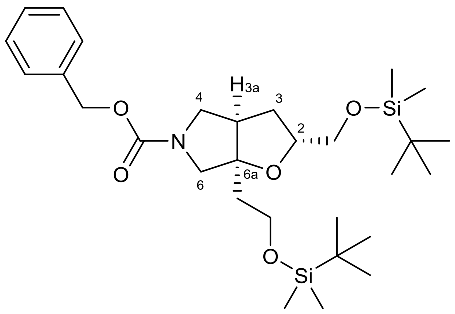
O
O
N
O
4
3
2
6
6a
O
O
Si
O
Si

S44

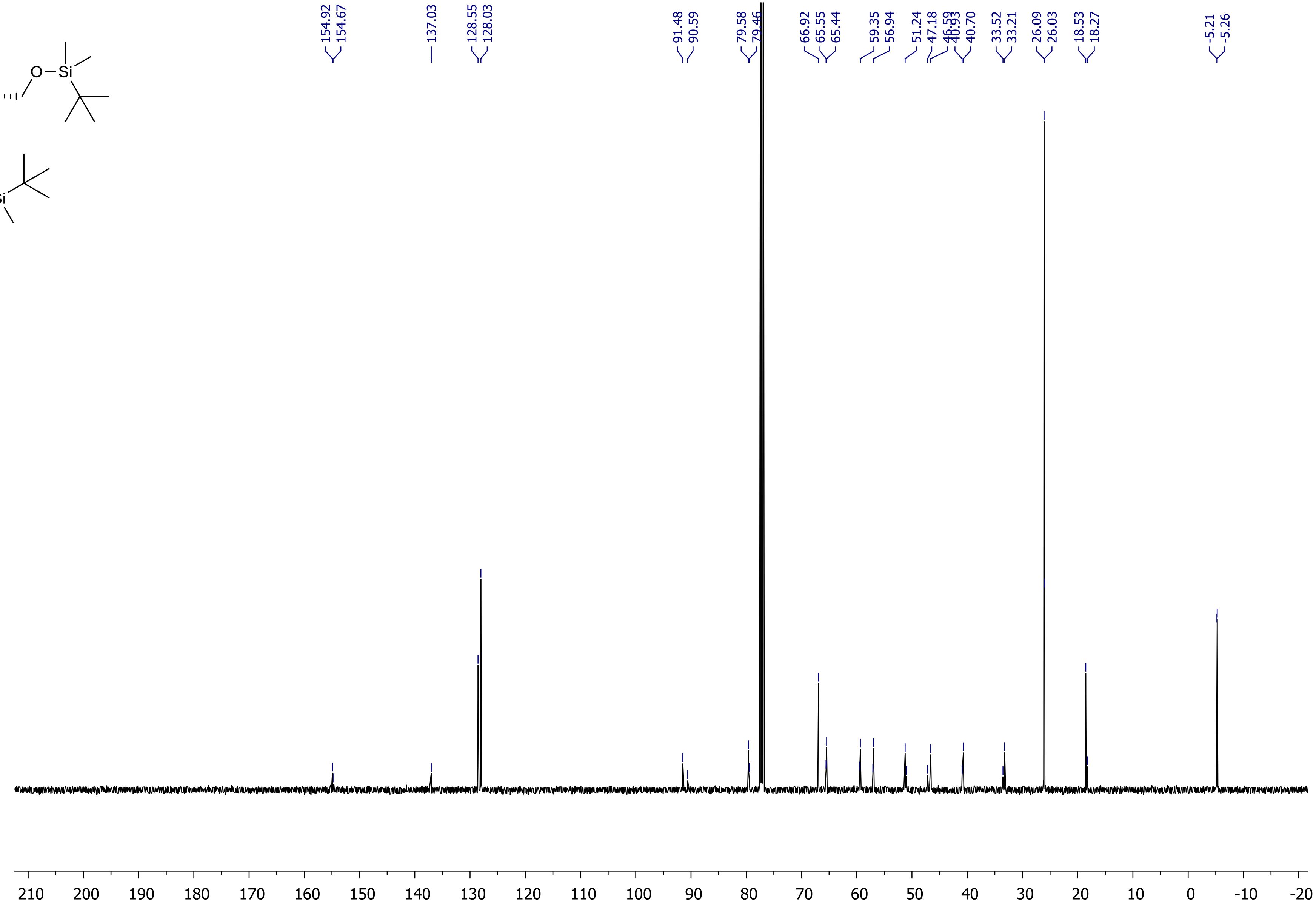
154.92
154.67
137.03
128.55
128.03
91.48
90.59
79.58
79.46
66.92
65.55
65.44
59.35
56.94
51.24
47.18
46.59
40.93
40.70
33.52
33.21
26.09
26.03
18.53
18.27
-5.21
-5.26
210
200
190
180
170
160
150
140
130
120
110
100
90
80
70
60
50
40
30
20
10
0
-10
-20

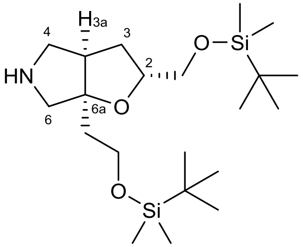
H3a
3
4
2
HN
O
Si
6a
O
6
O
Si

S45

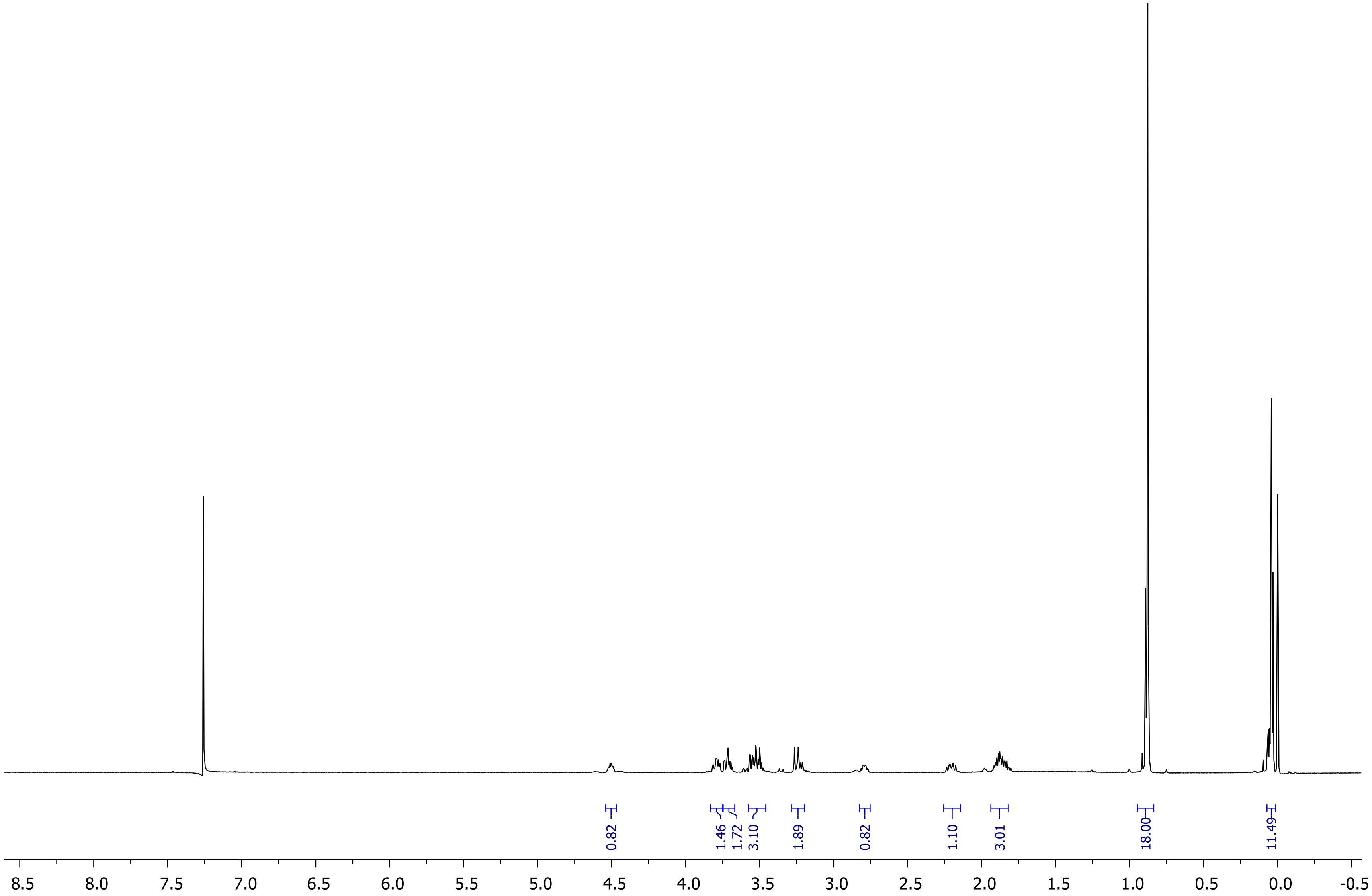
0.82
1.46
1.72
3.10
1.89
0.82
1.10
3.01
18.00
11.49
8.5
8.0
7.5
7.0
6.5
6.0
5.5
5.0
4.5
4.0
3.5
3.0
2.5
2.0
1.5
1.0
0.5
0.0
-0.5

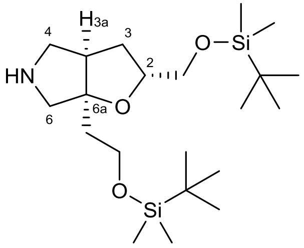
H3a
3
4
2
O
Si
HN
6a
O
6
O
Si

S45

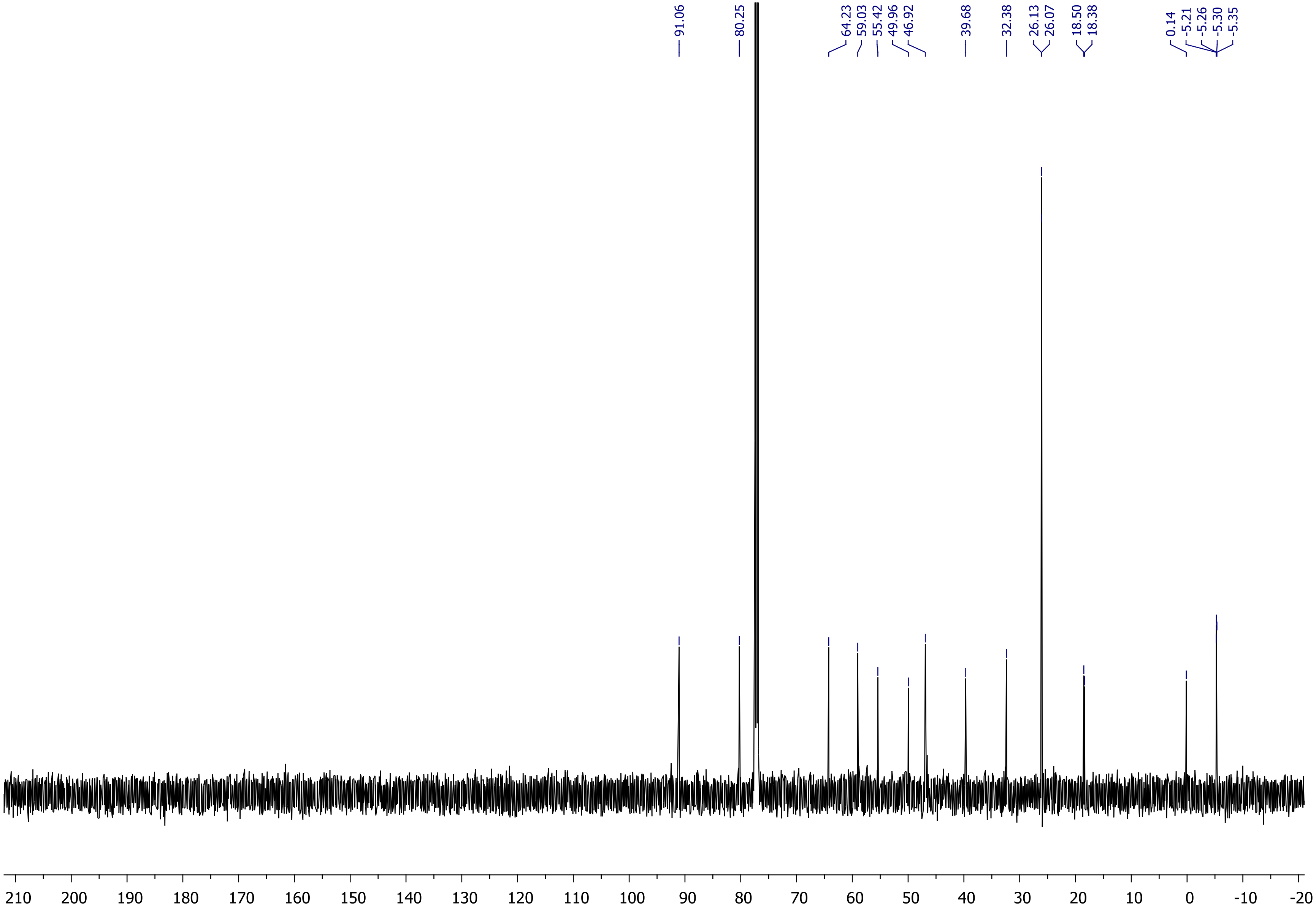
91.06
80.25
64.23
59.03
55.42
49.96
46.92
39.68
32.38
26.13
26.07
18.50
18.38
0.14
-5.21
-5.26
-5.30
-5.35
210 200 190 180 170 160 150 140 130 120 110 100 90 80 70 60 50 40 30 20 10 0 -10 -20

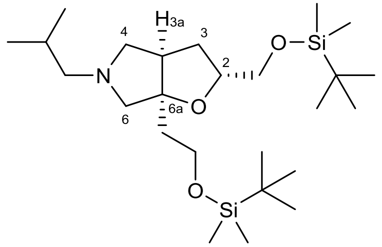

S46

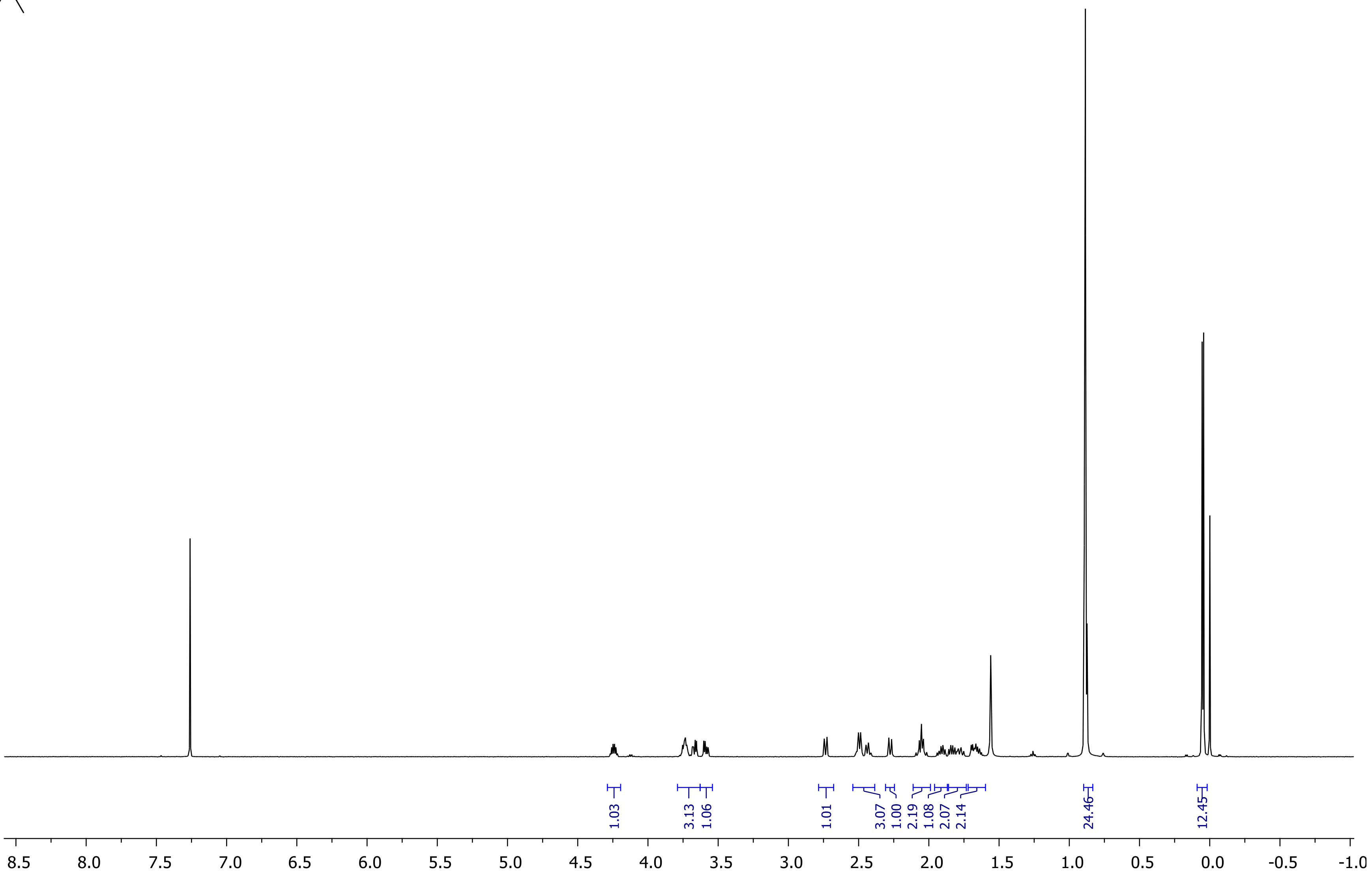
1.03
3.13
1.06
1.01
3.07
1.00
2.19
1.08
2.07
2.14
24.46
12.45
8.5
8.0
7.5
7.0
6.5
6.0
5.5
5.0
4.5
4.0
3.5
3.0
2.5
2.0
1.5
1.0
0.5
0.0
-0.5
-1.0

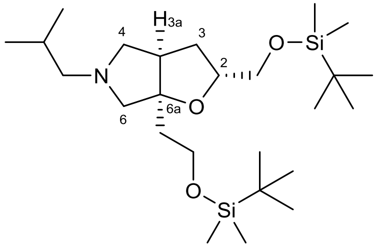
H3a
4
3
2
6
6a
N
O
O
Si

S46

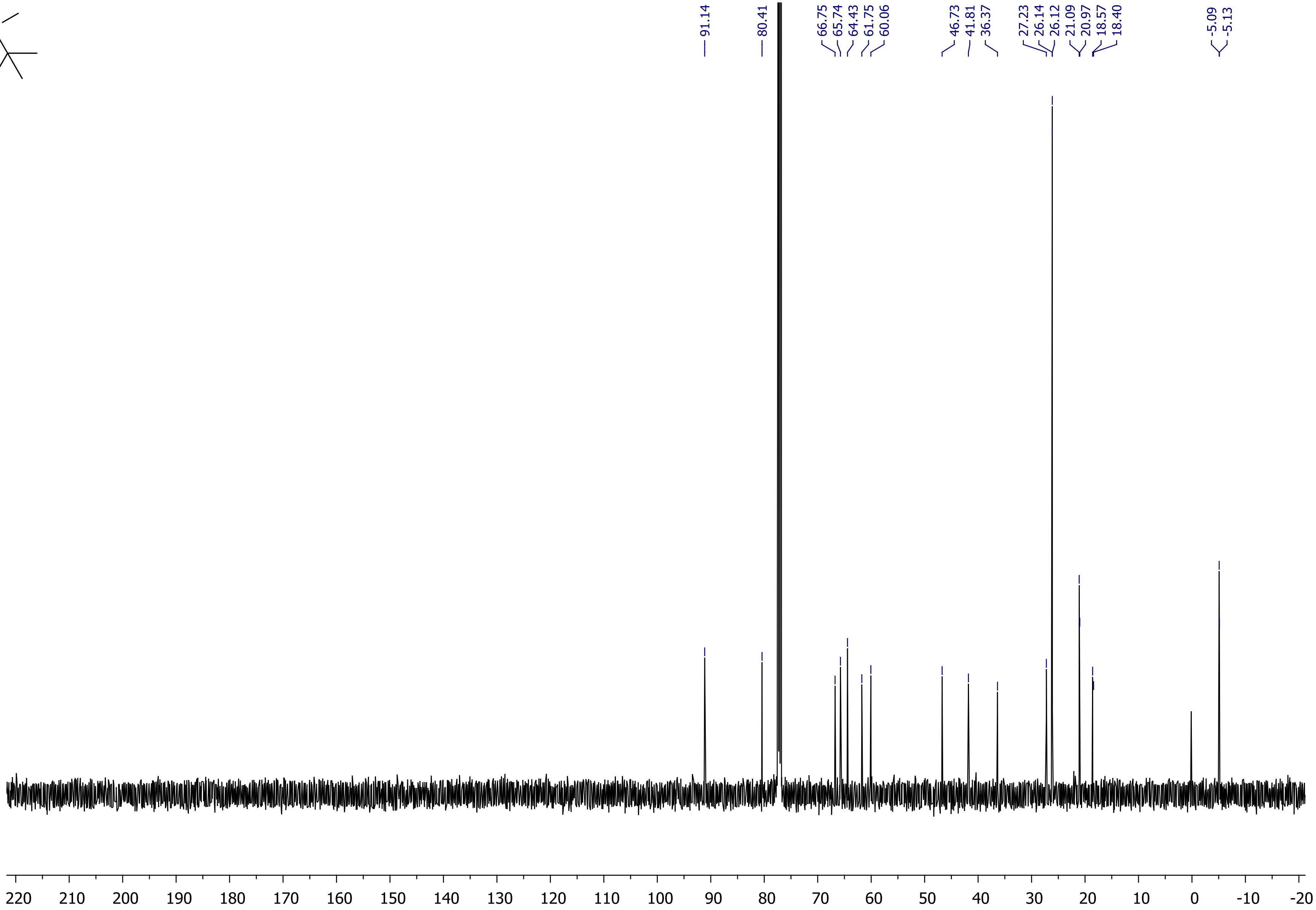
91.14
80.41
66.75
65.74
64.43
61.75
60.06
46.73
41.81
36.37
27.23
26.14
26.12
21.09
20.97
18.57
18.40
-5.09
-5.13
220 210 200 190 180 170 160 150 140 130 120 110 100 90 80 70 60 50 40 30 20 10 0 -10 -20

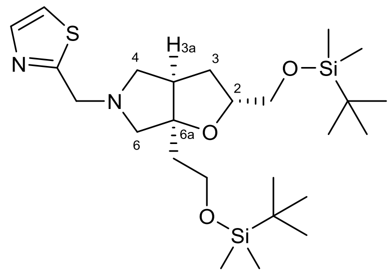

S
N
N
4
H3a
3
2
O
Si
6
6a
O
O
Si

S47

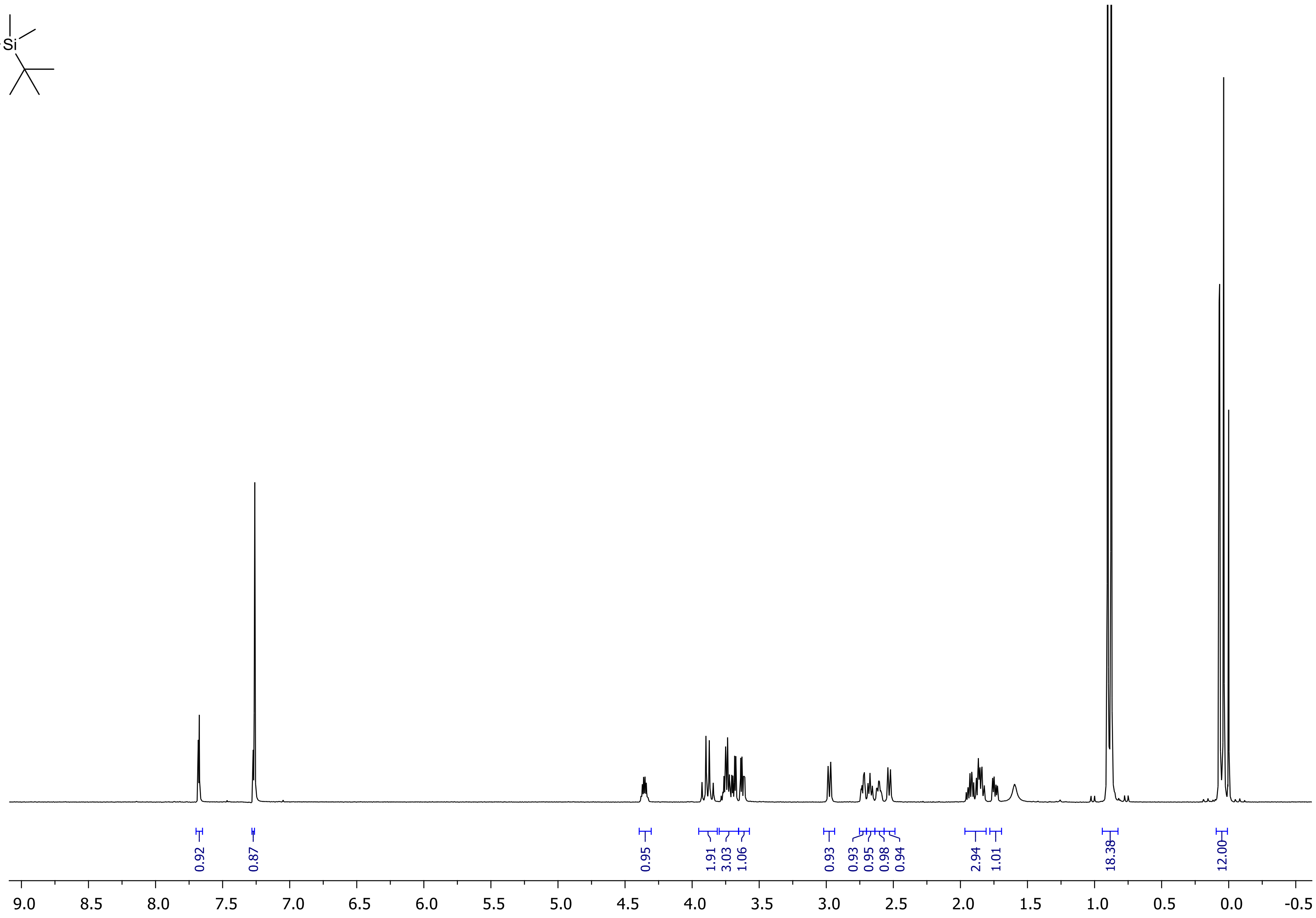

0.92
0.87
0.95
1.91
3.03
1.06
0.93
0.93
0.95
0.98
0.94
2.94
1.01
18.38
12.00
9.0
8.5
8.0
7.5
7.0
6.5
6.0
5.5
5.0
4.5
4.0
3.5
3.0
2.5
2.0
1.5
1.0
0.5
0.0
-0.5

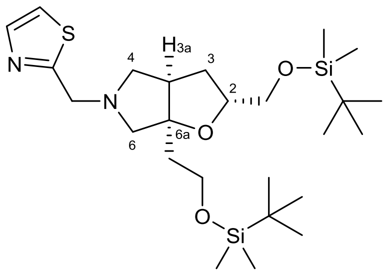

S
N
N
H3a
4
3
2
6
6a
O
O
Si
O
Si

S47

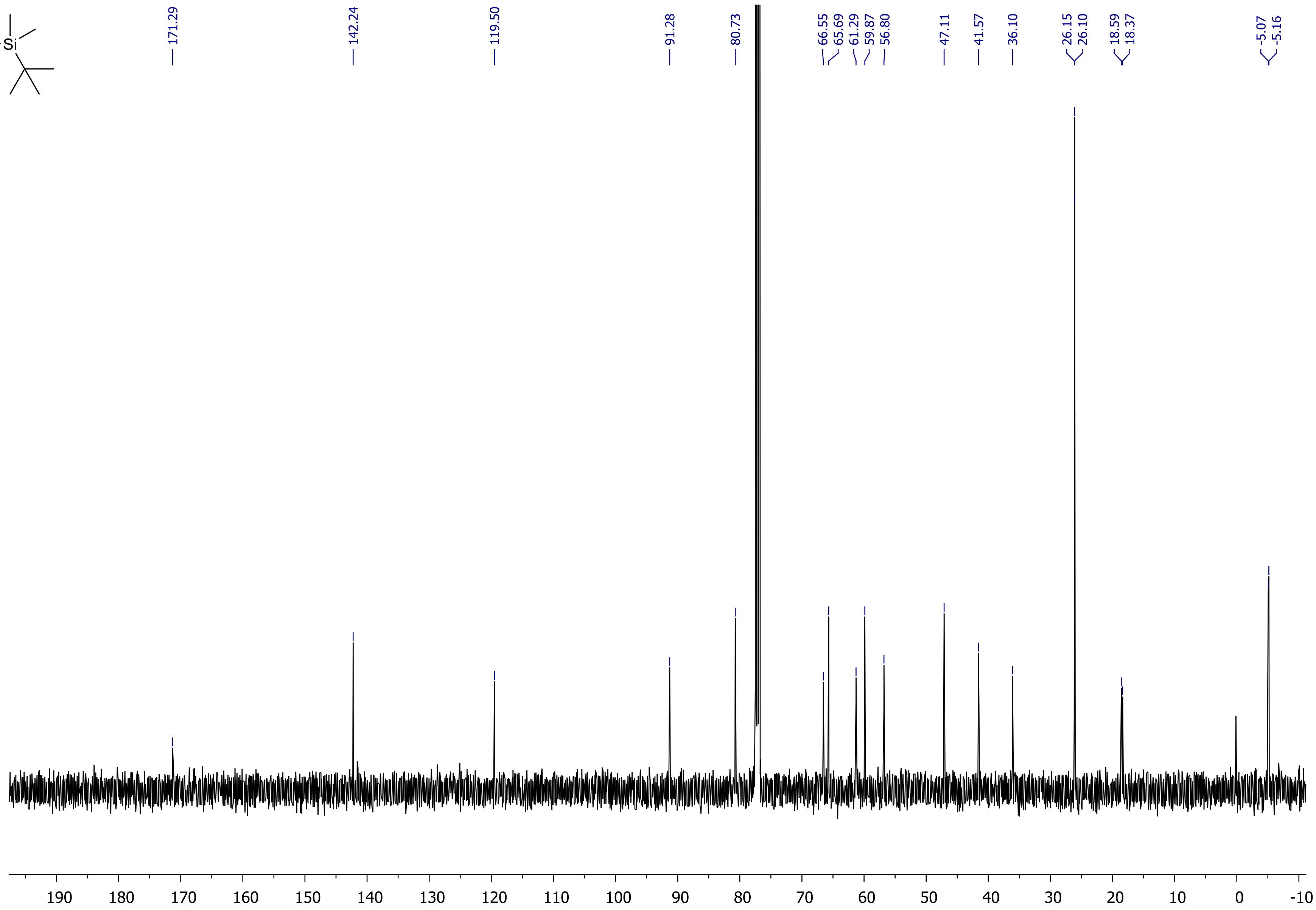

171.29
142.24
119.50
91.28
80.73
66.55
65.69
61.29
59.87
56.80
47.11
41.57
36.10
26.15
26.10
18.59
18.37
-5.07
-5.16
190
180
170
160
150
140
130
120
110
100
90
80
70
60
50
40
30
20
10
0
-10

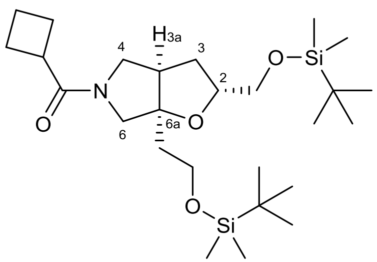

S48

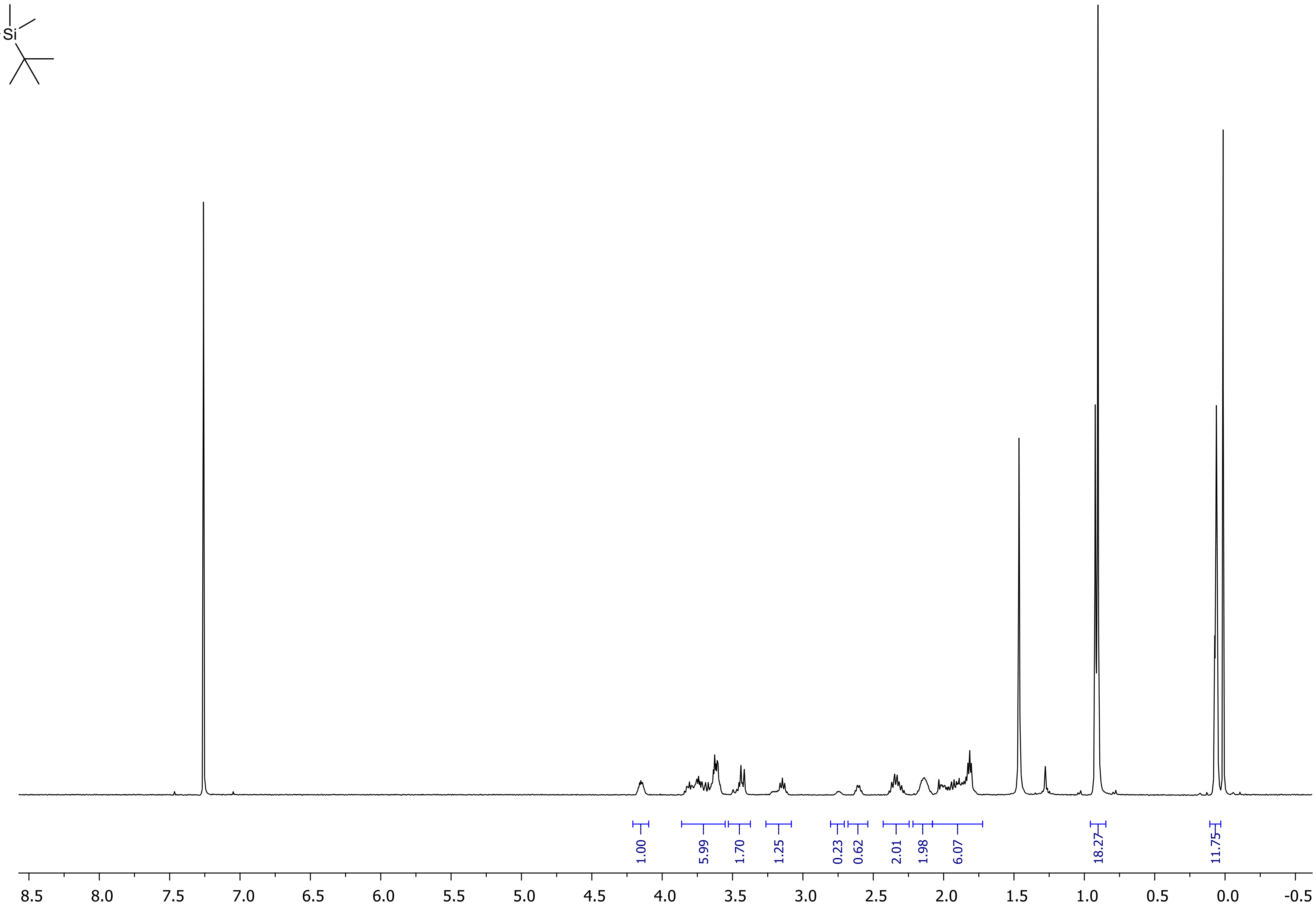
1.00
5.99
1.70
1.25
0.23
0.62
2.01
1.98
6.07
18.27
11.75
8.5 8.0 7.5 7.0 6.5 6.0 5.5 5.0 4.5 4.0 3.5 3.0 2.5 2.0 1.5 1.0 0.5 0.0 -0.5

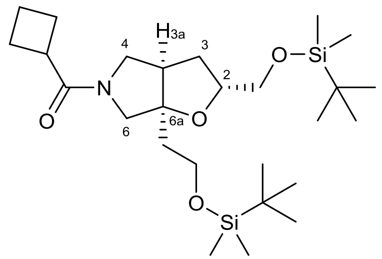
H3a
4
3
2
O
Si
N
O
6
6a
O
O
Si

S48

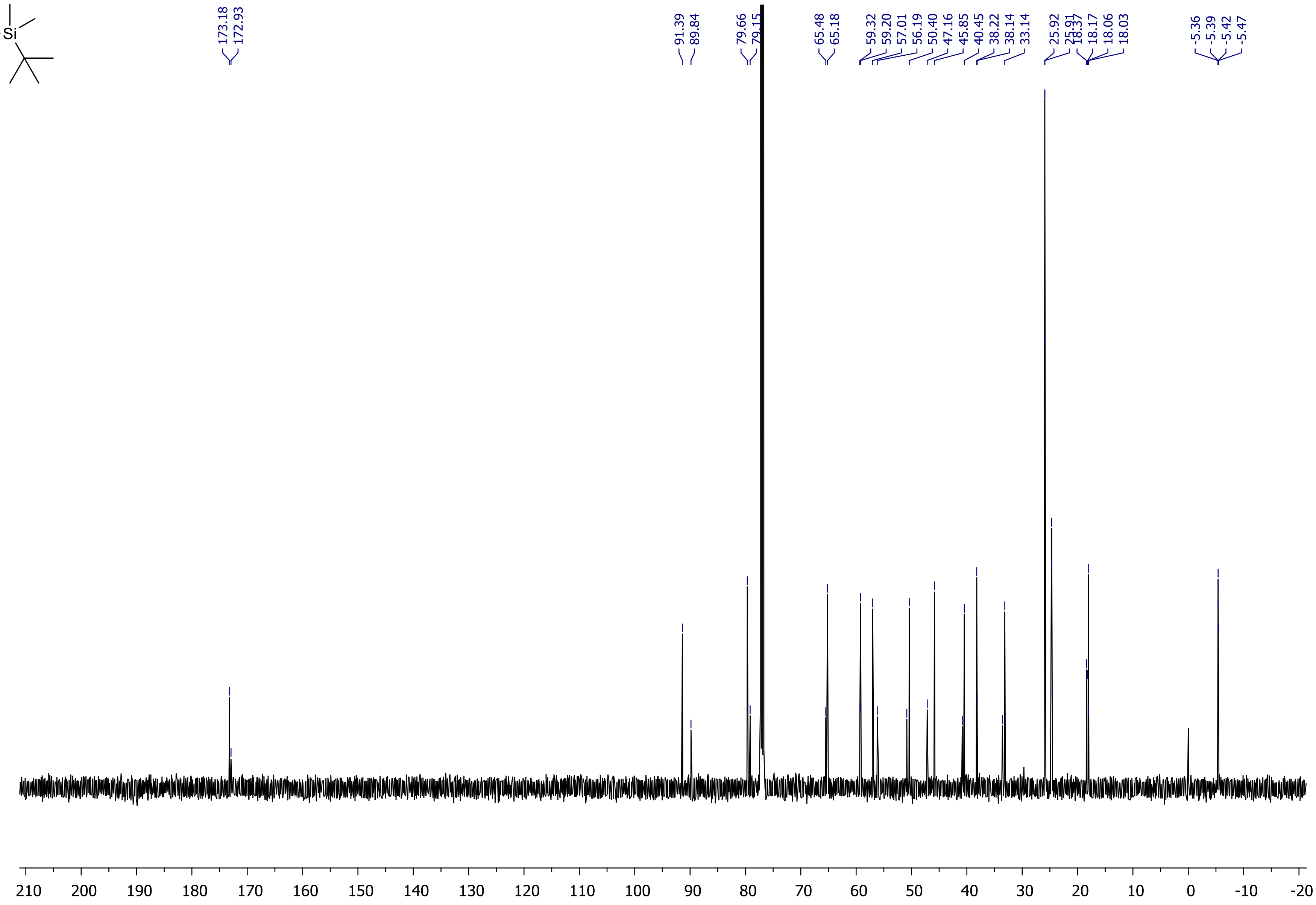
173.18
172.93
91.39
89.84
79.66
79.15
65.48
65.18
59.32
59.20
57.01
56.19
50.40
47.16
45.85
40.45
38.22
38.14
33.14
25.92
25.91
18.37
18.17
18.06
18.03
-5.36
-5.39
-5.42
-5.47
210
200
190
180
170
160
150
140
130
120
110
100
90
80
70
60
50
40
30
20
10
0
-10
-20

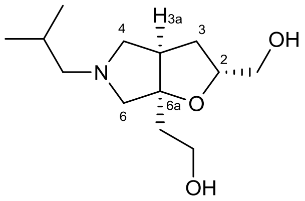
H3a
4
3
2
OH
N
6
6a
O
OH

F26

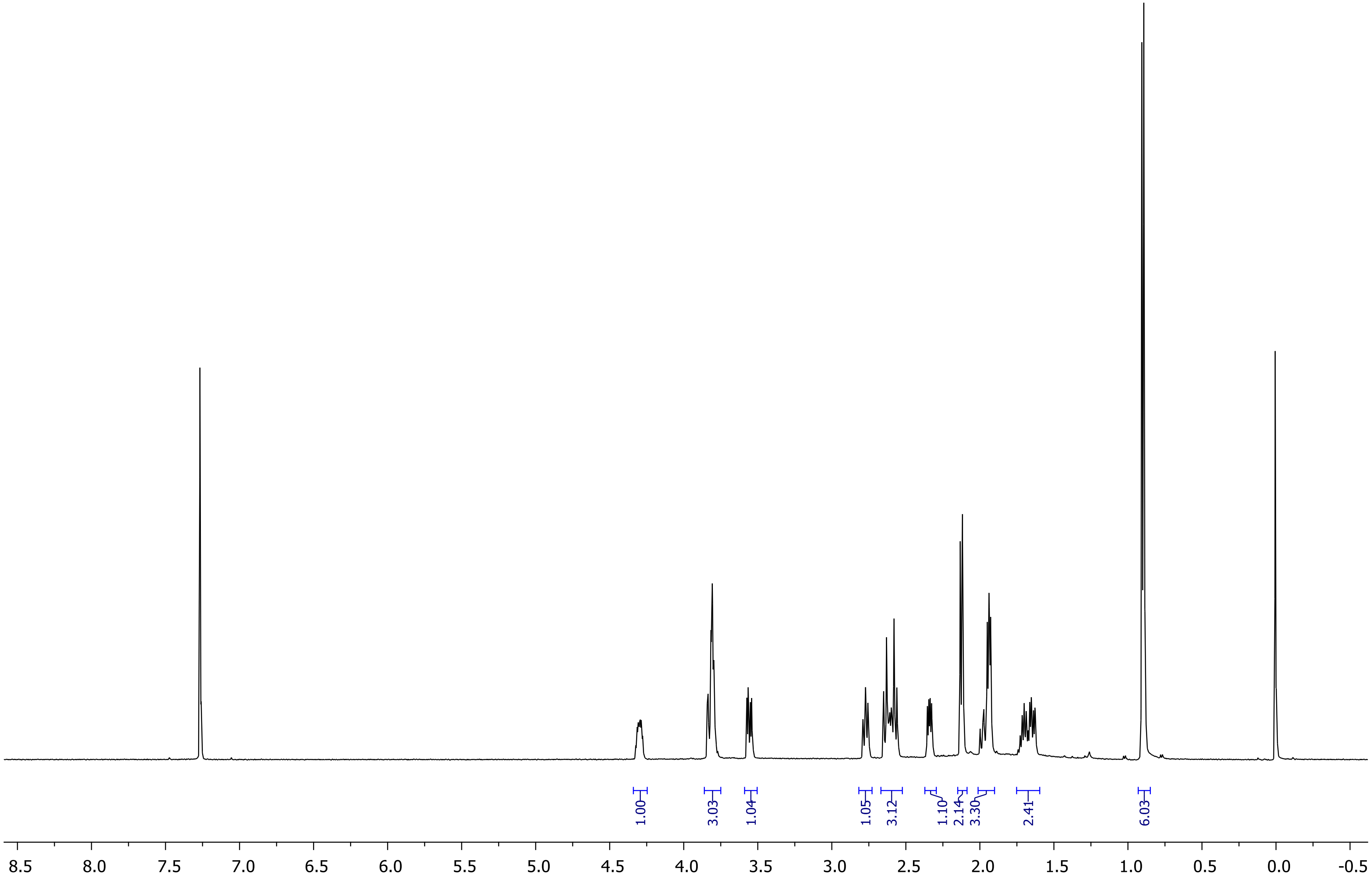
1.00
3.03
1.04
1.05
3.12
1.10
2.14
3.30
2.41
6.03
8.5
8.0
7.5
7.0
6.5
6.0
5.5
5.0
4.5
4.0
3.5
3.0
2.5
2.0
1.5
1.0
0.5
0.0
-0.5

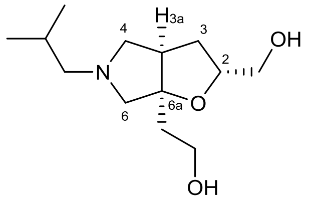

F26

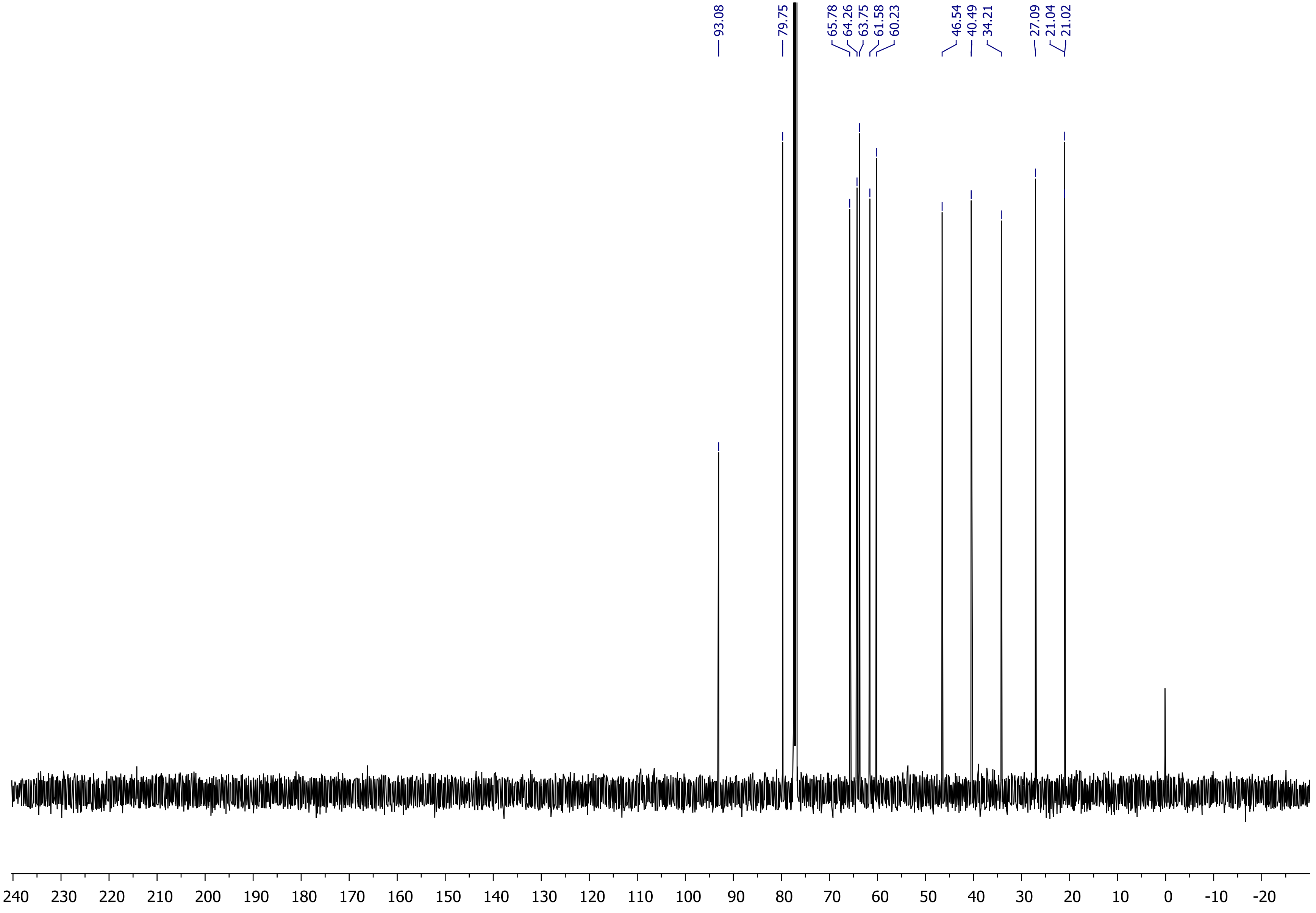
93.08
79.75
65.78
64.26
63.75
61.58
60.23
46.54
40.49
34.21
27.09
21.04
21.02
240 230 220 210 200 190 180 170 160 150 140 130 120 110 100 90 80 70 60 50 40 30 20 10 0 -10 -20

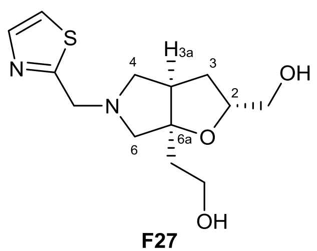
S
N
H3a
4
3
2
OH
N
6a
O
6
OH

F27

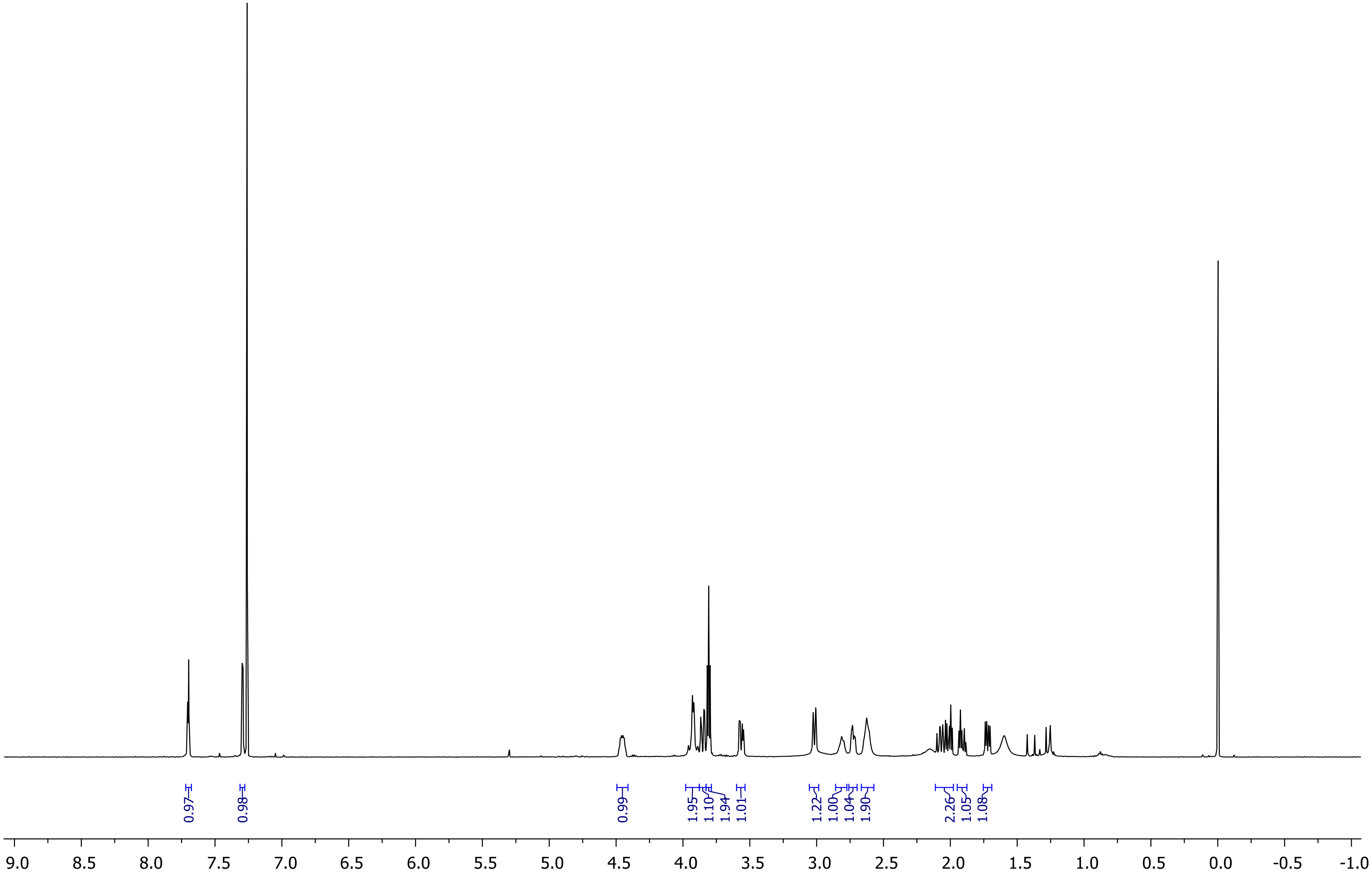
0.97
0.98
0.99
1.95
1.10
1.94
1.01
1.22
1.00
1.04
1.90
2.26
1.05
1.08
9.0
8.5
8.0
7.5
7.0
6.5
6.0
5.5
5.0
4.5
4.0
3.5
3.0
2.5
2.0
1.5
1.0
0.5
0.0
-0.5
-1.0

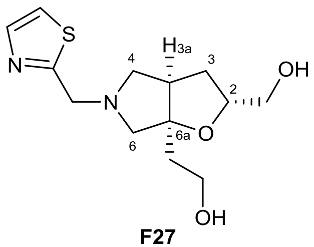
S
N
H3a
4
3
2
OH
N
6a
O
6
OH

F27

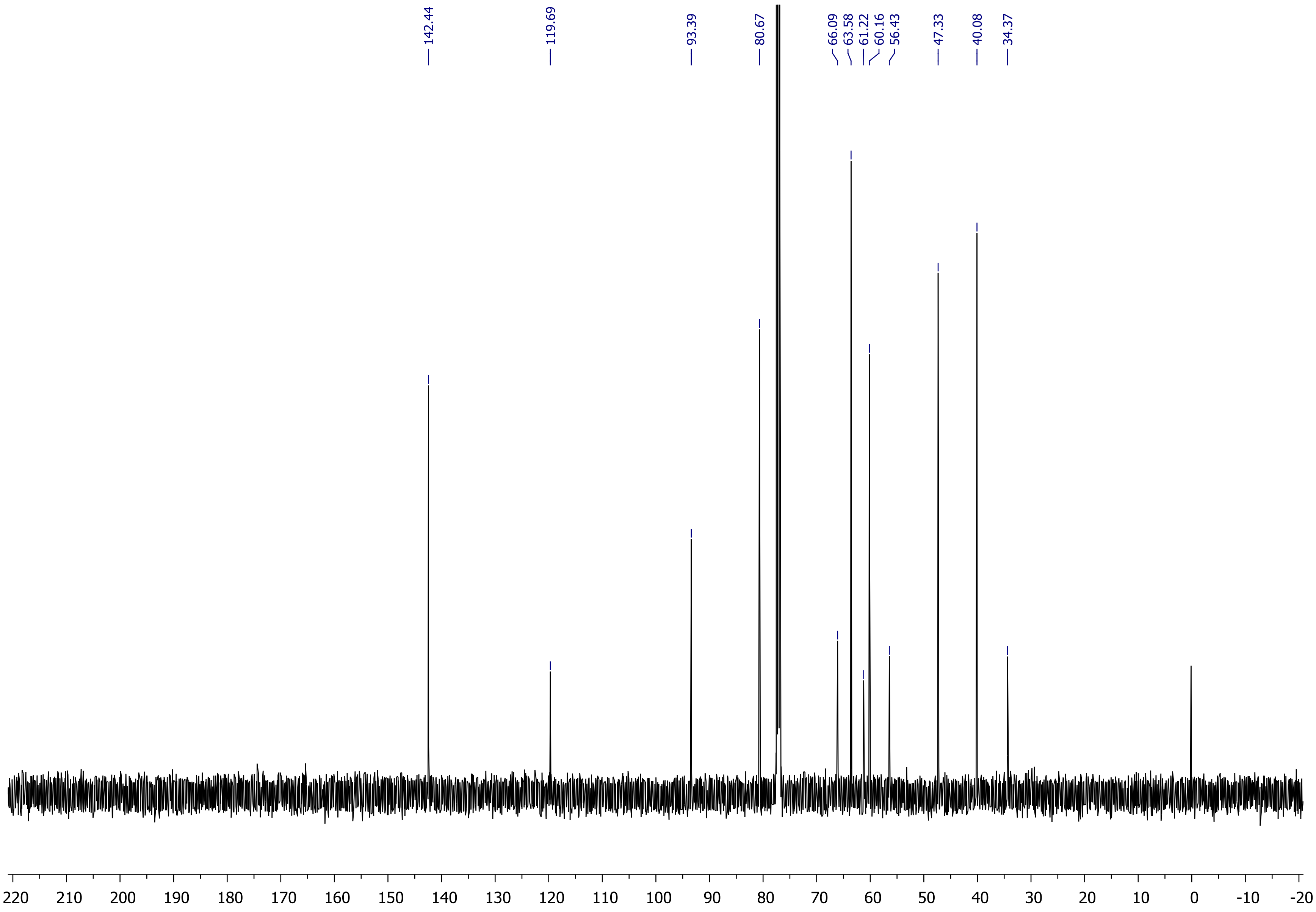
142.44
119.69
93.39
80.67
66.09
63.58
61.22
60.16
56.43
47.33
40.08
34.37
220 210 200 190 180 170 160 150 140 130 120 110 100 90 80 70 60 50 40 30 20 10 0 -10 -20

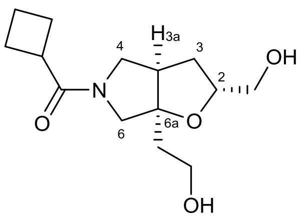
H3a
4
3
2
OH
N
O
6a
6
O
OH

F28

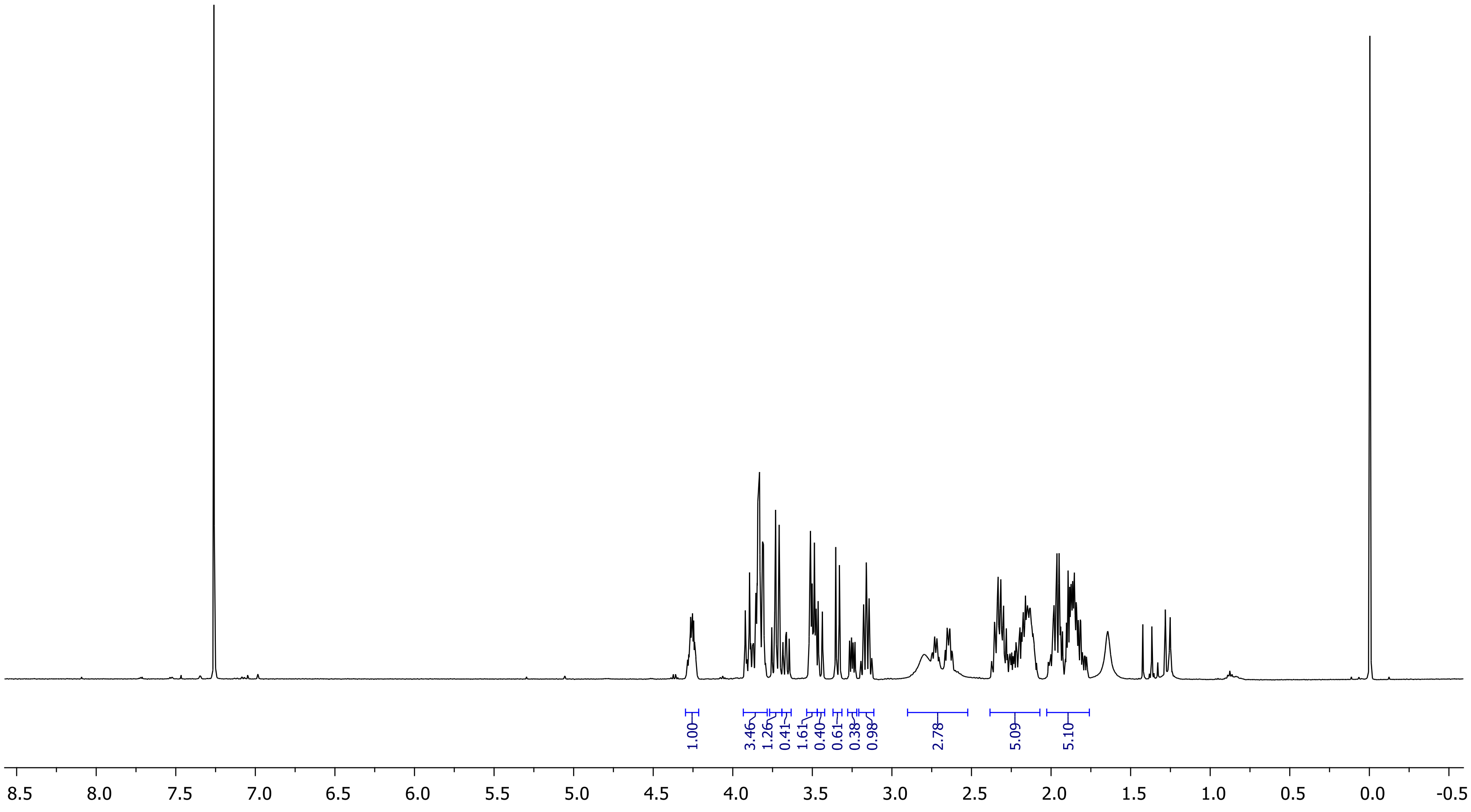
1.00
3.46
1.26
0.41
1.61
0.40
0.61
0.38
0.98
2.78
5.09
5.10
8.5
8.0
7.5
7.0
6.5
6.0
5.5
5.0
4.5
4.0
3.5
3.0
2.5
2.0
1.5
1.0
0.5
0.0
-0.5

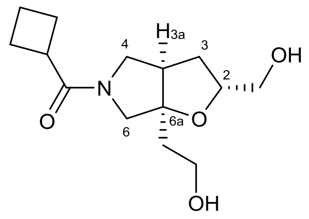
H3a
4
3
2
OH
N
O
6a
6
O
OH

F28

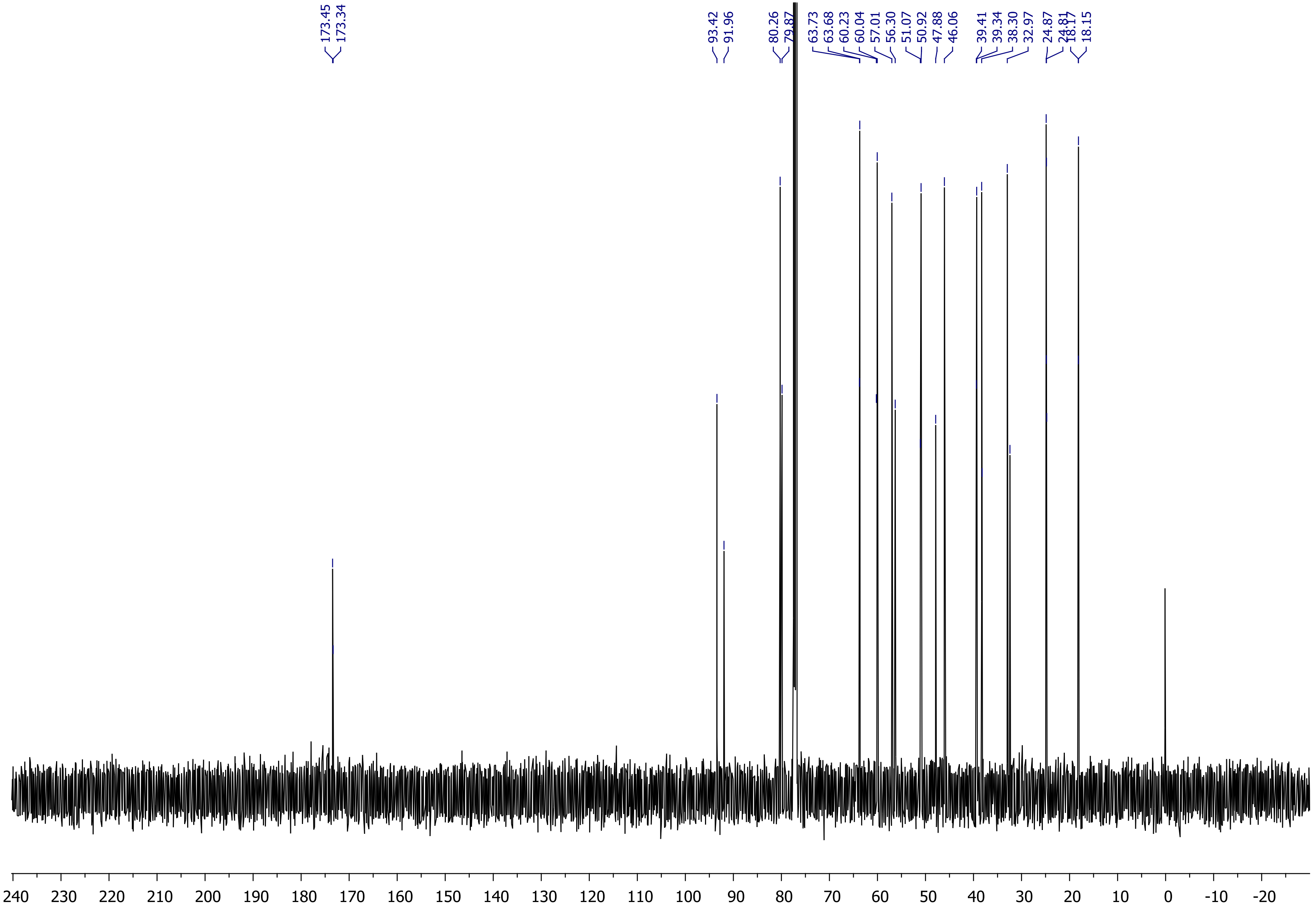
173.45
173.34
93.42
91.96
80.26
79.87
63.73
63.68
60.23
60.04
57.01
56.30
51.07
50.92
47.88
46.06
39.41
39.34
38.30
32.97
24.87
24.81
18.17
18.15
240 230 220 210 200 190 180 170 160 150 140 130 120 110 100 90 80 70 60 50 40 30 20 10 0 -10 -20

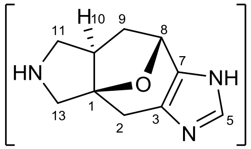

F29•2HCl
(MeOD-d_4 @ 333 K)

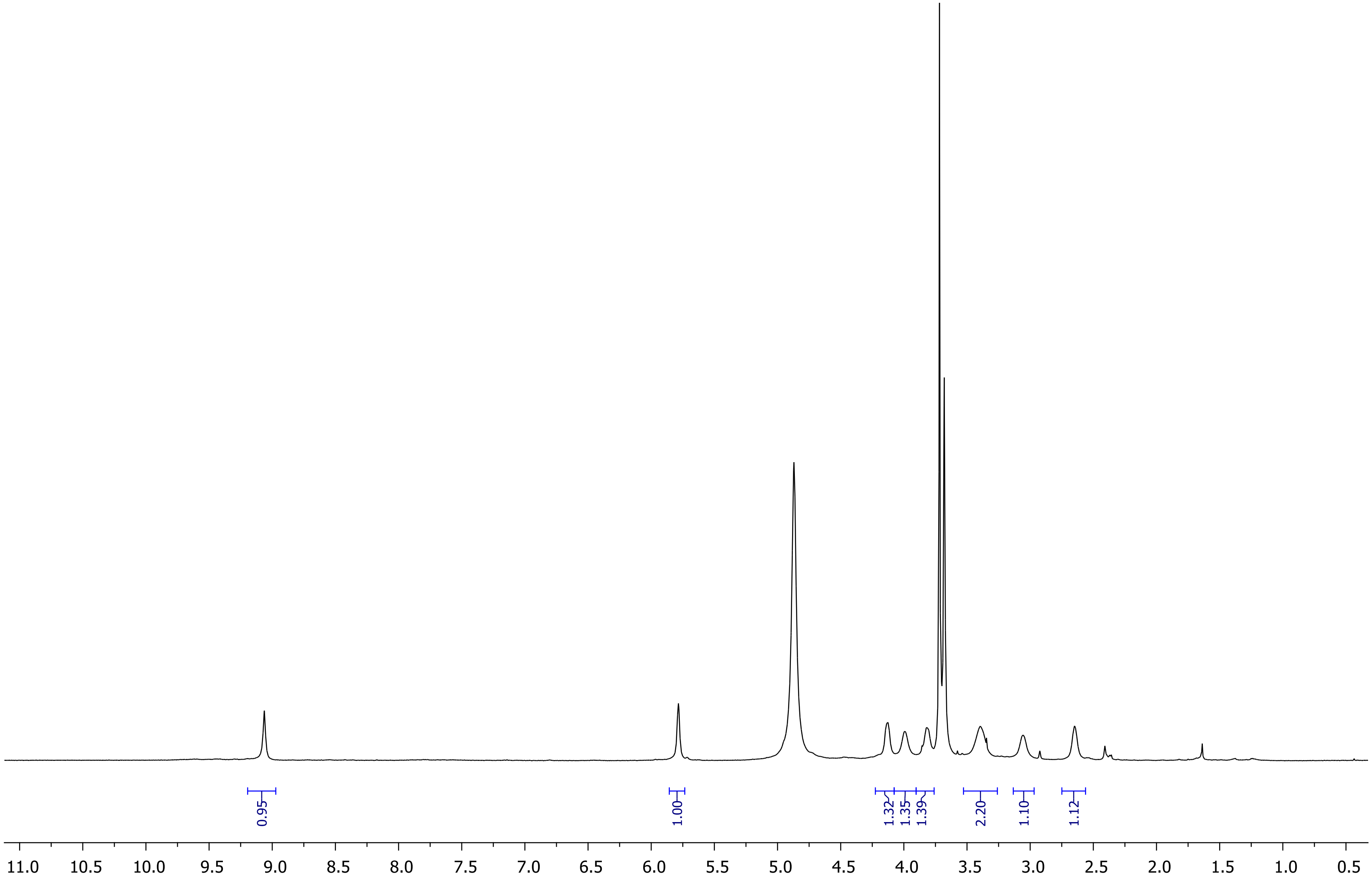

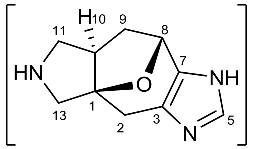

F29•2HCl
(MeOD-d_4 @ 333 K)

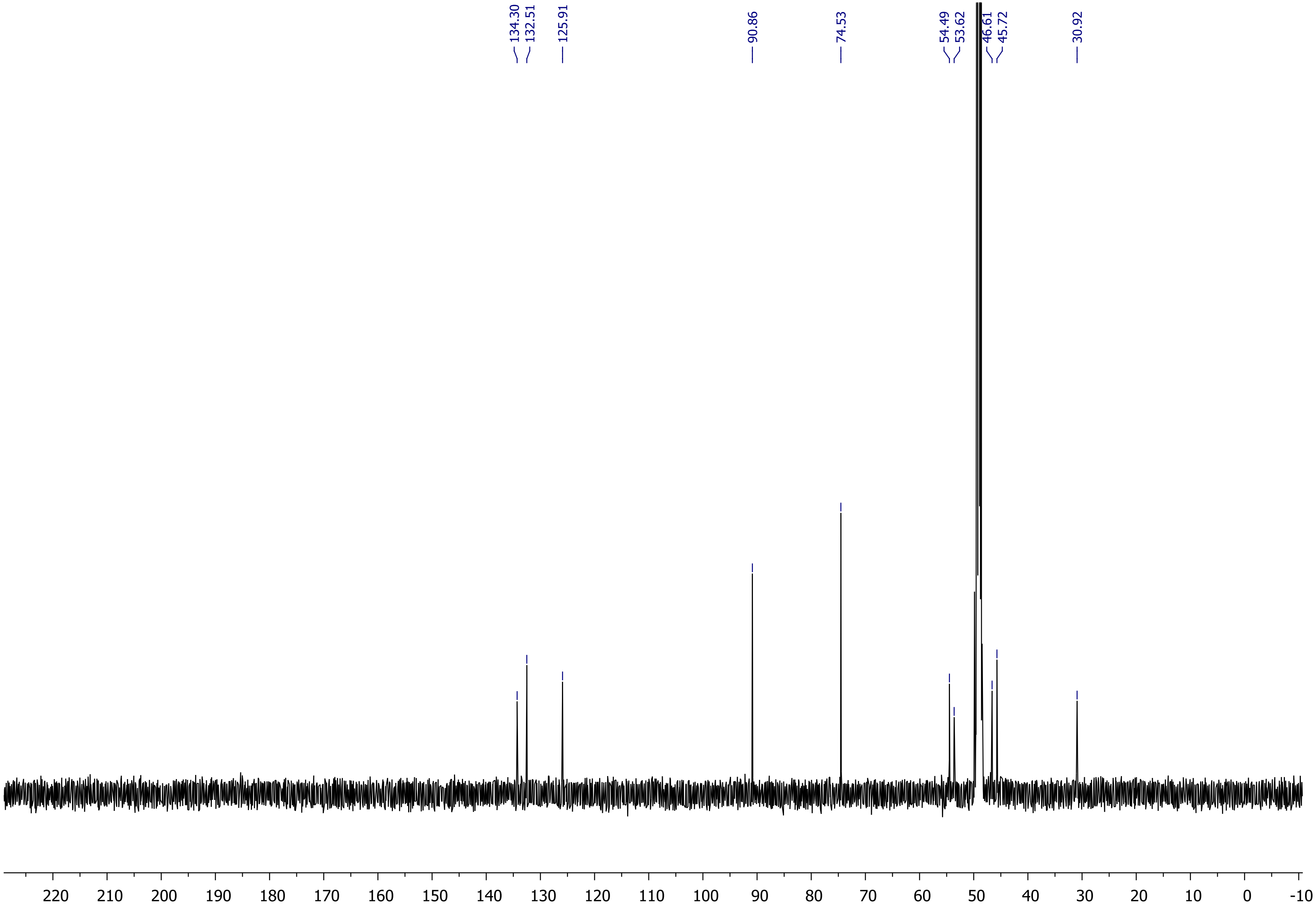

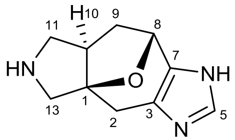
HN
NH
O
N
H
1
2
3
5
7
8
9
10
11
13

F29

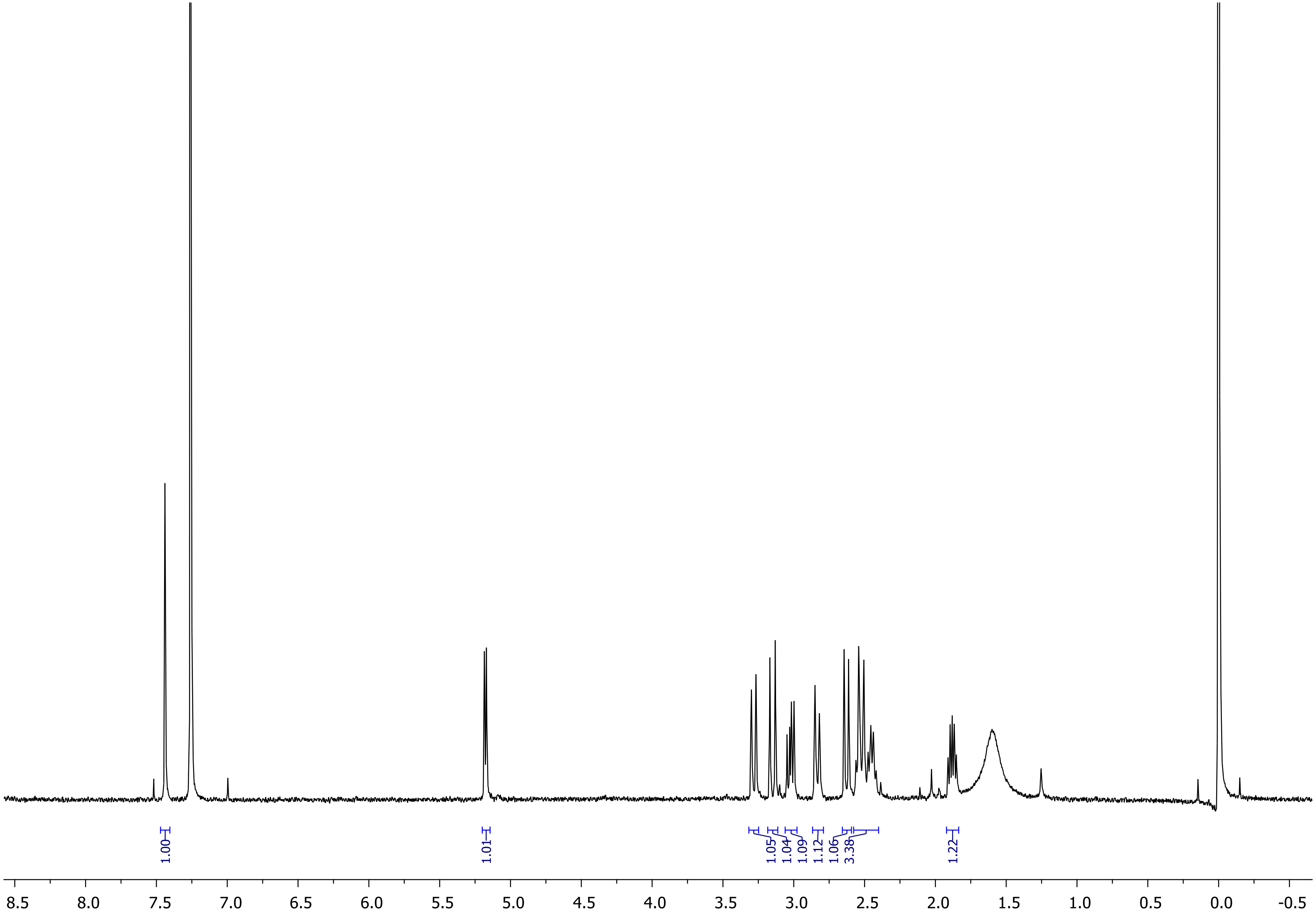
1.00
1.01
1.05
1.04
1.09
1.12
1.06
3.38
1.22
8.5
8.0
7.5
7.0
6.5
6.0
5.5
5.0
4.5
4.0
3.5
3.0
2.5
2.0
1.5
1.0
0.5
0.0
-0.5

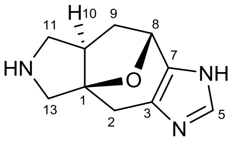
H
10
9
8
11
7
HN
O
NH
13
1
2
3
5
N

F29

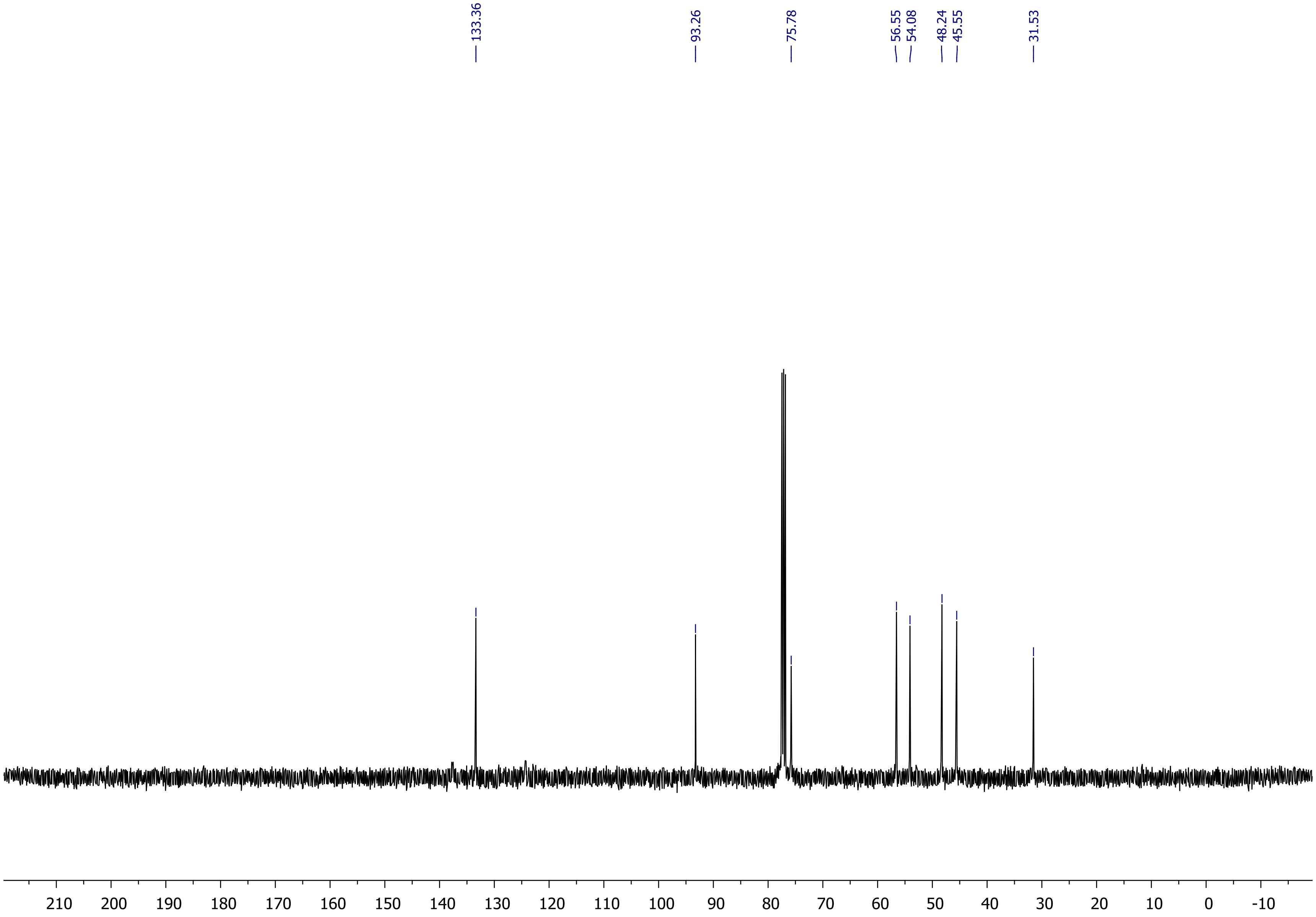
133.36
93.26
75.78
56.55
54.08
48.24
45.55
31.53
210
200
190
180
170
160
150
140
130
120
110
100
90
80
70
60
50
40
30
20
10
0
-10

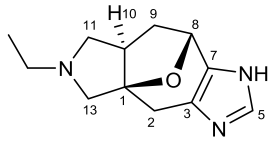

H
10
9
8
11
7
NH
N
O
13
1
2
3
N
5

F30

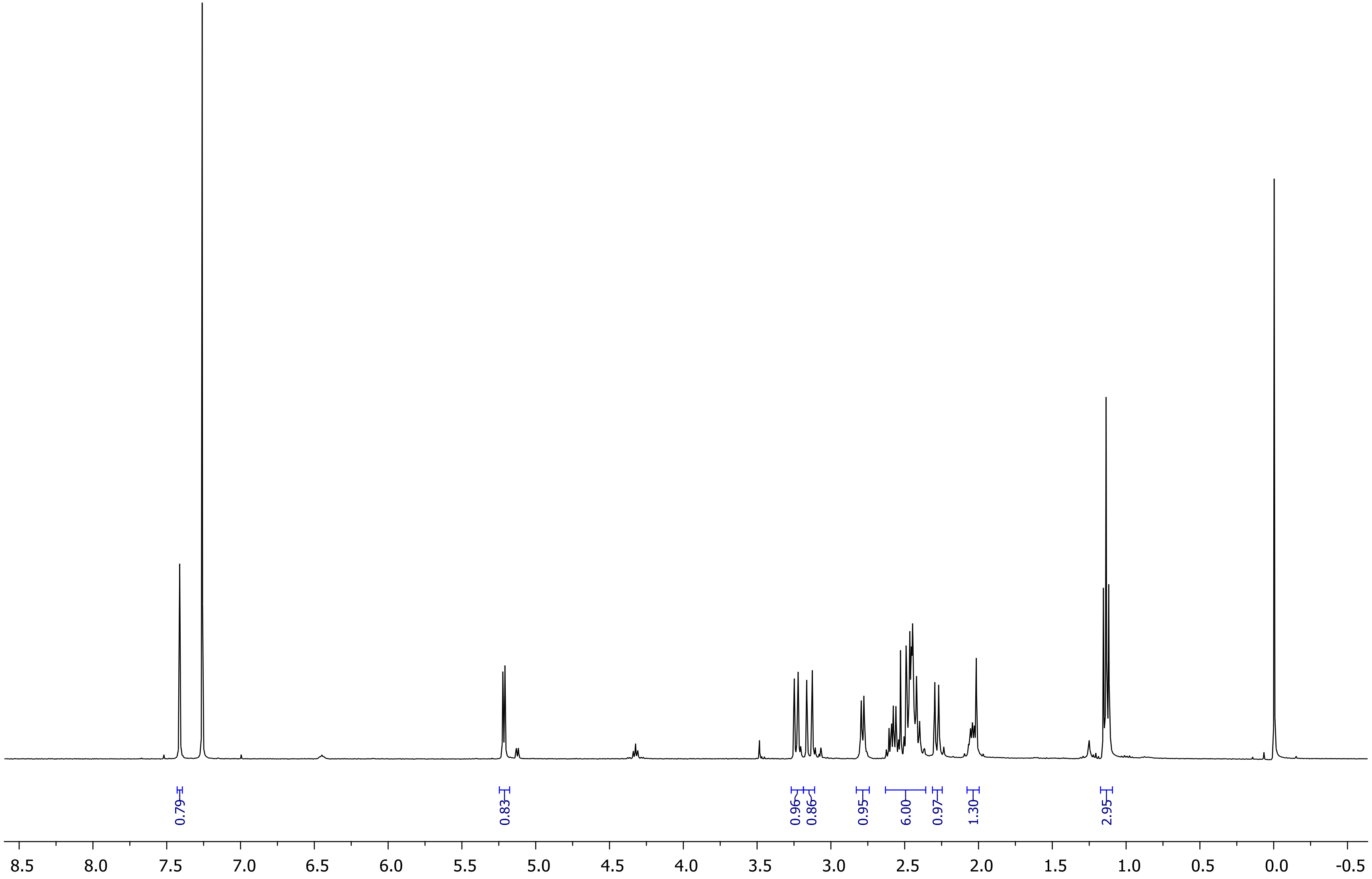

0.79
0.83
0.96
0.86
0.95
6.00
0.97
1.30
2.95
8.5
8.0
7.5
7.0
6.5
6.0
5.5
5.0
4.5
4.0
3.5
3.0
2.5
2.0
1.5
1.0
0.5
0.0
-0.5

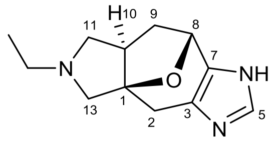

F30

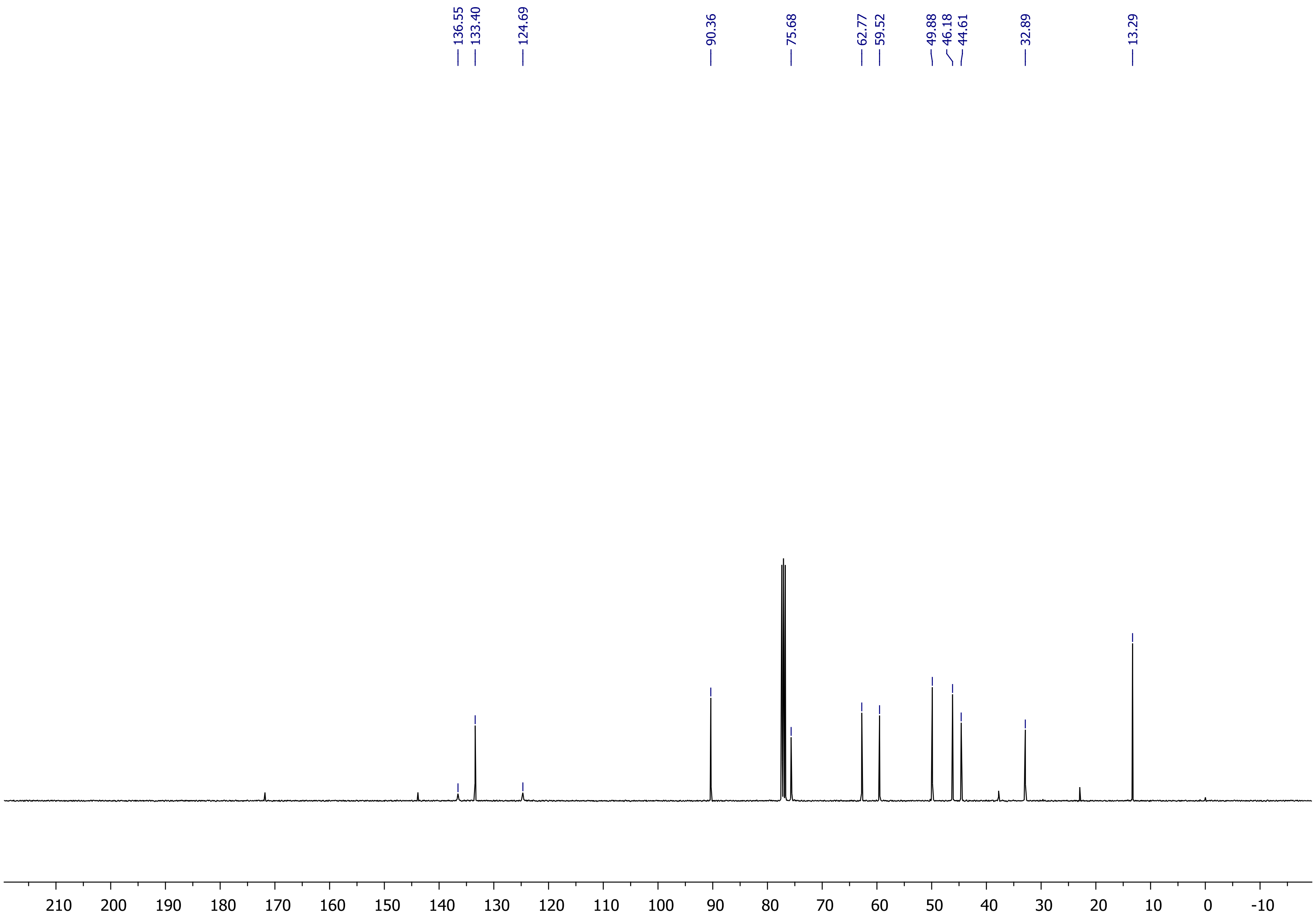
136.55
133.40
124.69
90.36
75.68
62.77
59.52
49.88
46.18
44.61
32.89
13.29
210
200
190
180
170
160
150
140
130
120
110
100
90
80
70
60
50
40
30
20
10
0
-10

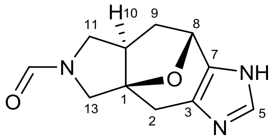

H
10
9
8
11
7
NH
N
O
O
13
1
2
3
N
5

F31

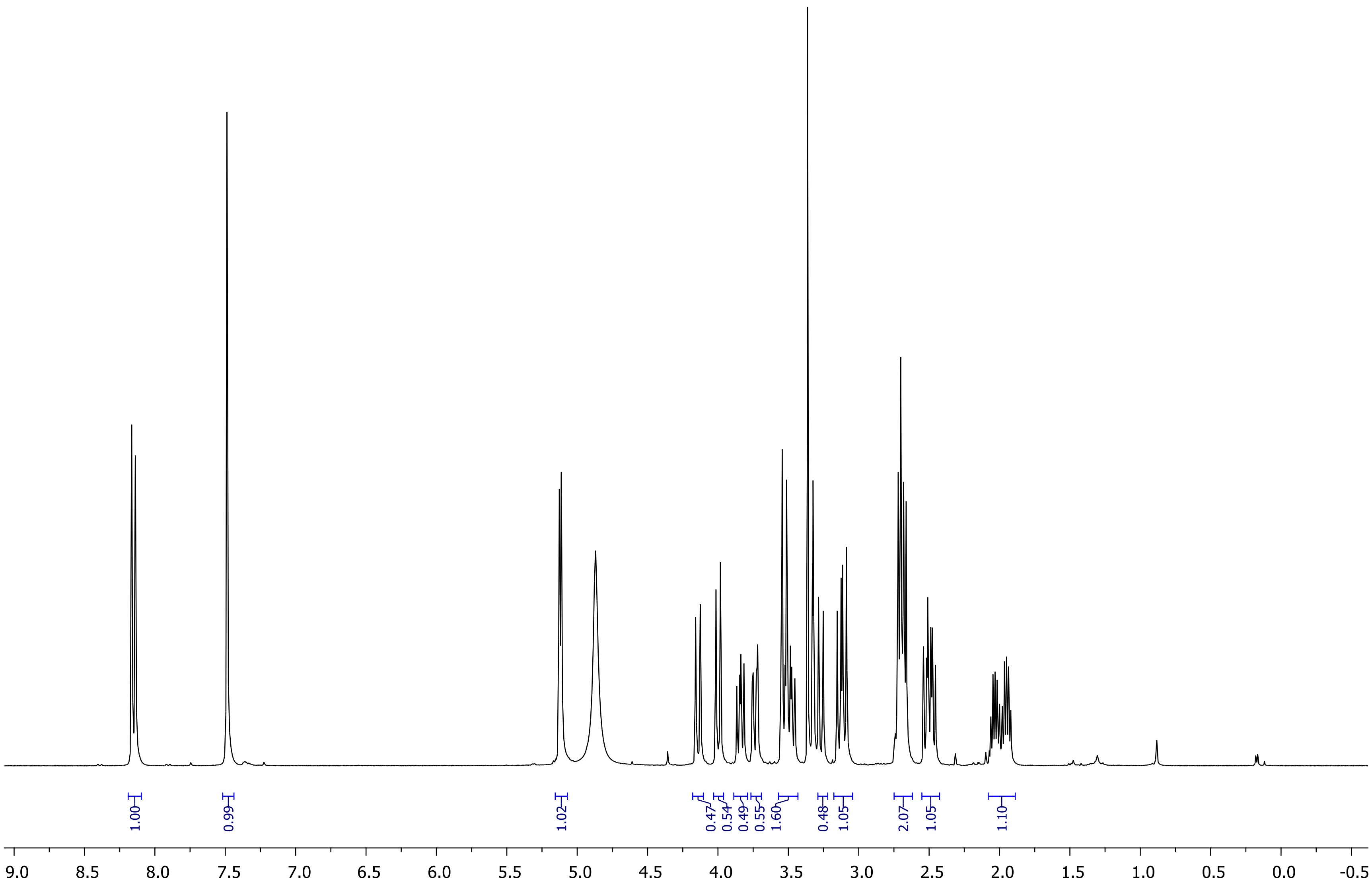

1.00
0.99
1.02
0.47
0.54
0.49
0.55
1.60
0.48
1.05
2.07
1.05
1.10
9.0
8.5
8.0
7.5
7.0
6.5
6.0
5.5
5.0
4.5
4.0
3.5
3.0
2.5
2.0
1.5
1.0
0.5
0.0
-0.5

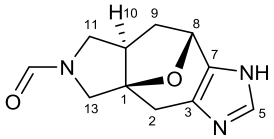
H
N
O
NH
N
1
2
3
5
7
8
9
10
11
13

F31

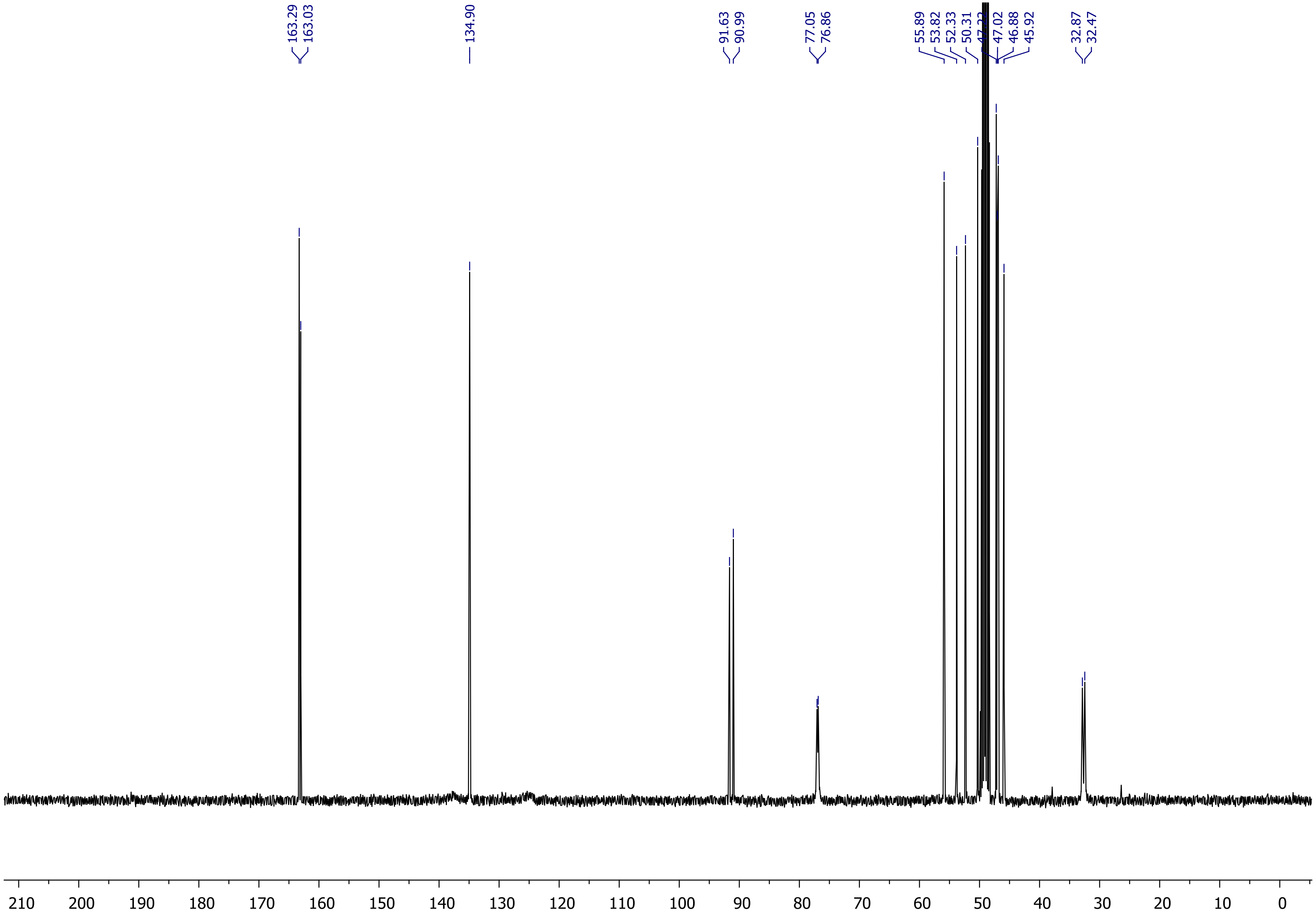
163.29
163.03
134.90
91.63
90.99
77.05
76.86
55.89
53.82
52.33
50.31
47.02
46.88
45.92
32.87
32.47
210
200
190
180
170
160
150
140
130
120
110
100
90
80
70
60
50
40
30
20
10
0

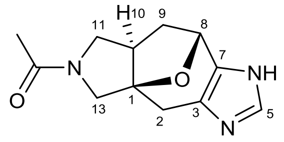

H10
9
8
11
7
NH
N
O
O
13
1
2
3
5
N

F32

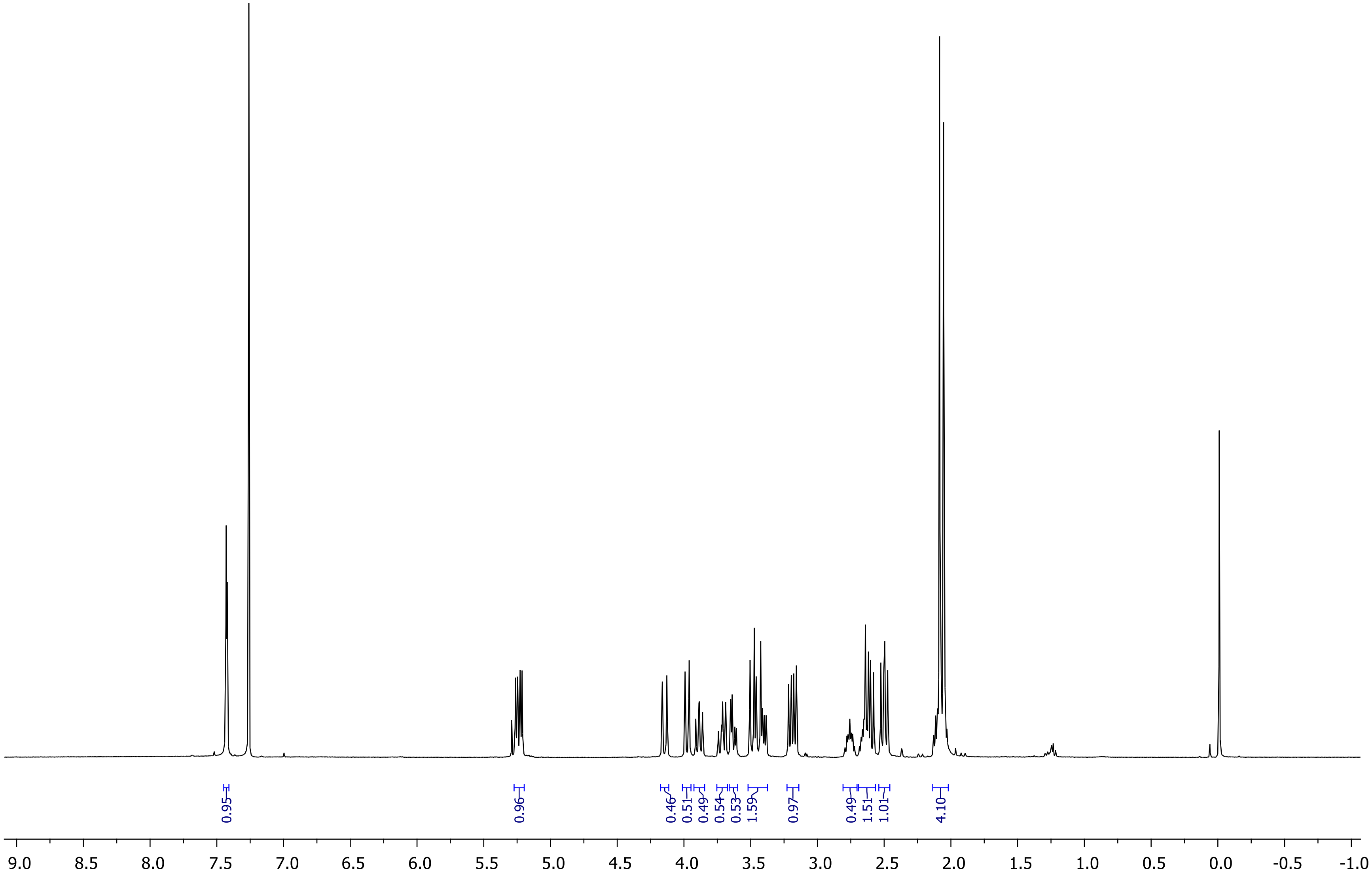

0.95
0.96
0.46
0.51
0.49
0.54
0.53
1.59
0.97
0.49
1.51
1.01
4.10
9.0
8.5
8.0
7.5
7.0
6.5
6.0
5.5
5.0
4.5
4.0
3.5
3.0
2.5
2.0
1.5
1.0
0.5
0.0
-0.5
-1.0

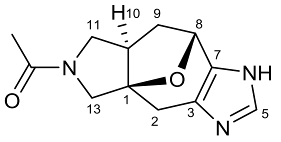

H
10
9
8
11
7
N
NH
O
O
13
1
2
3
N
5

F32

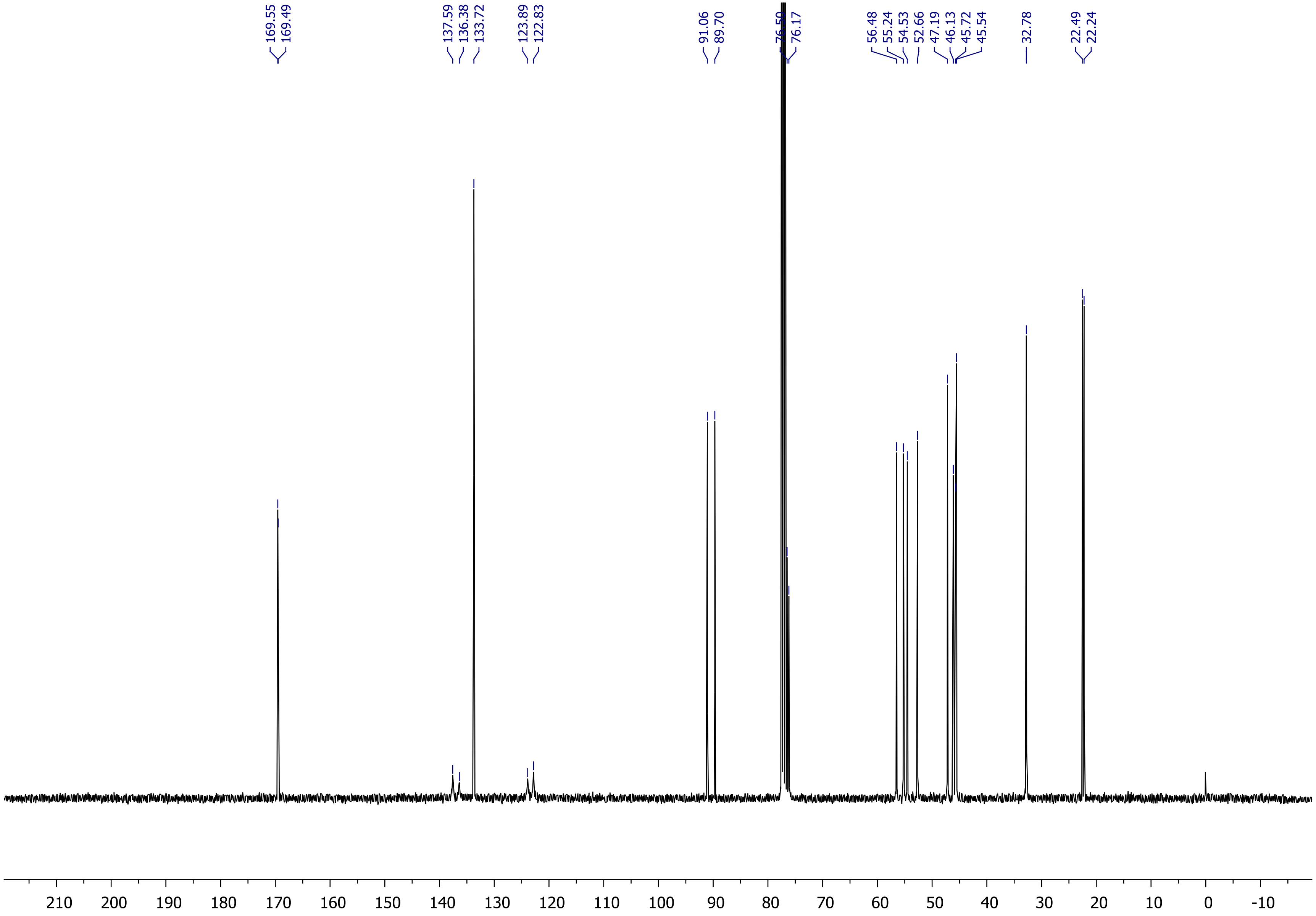

169.55
169.49
137.59
136.38
133.72
123.89
122.83
91.06
89.70
76.17
56.48
55.24
54.53
52.66
47.19
46.13
45.72
45.54
32.78
22.49
22.24
210 200 190 180 170 160 150 140 130 120 110 100 90 80 70 60 50 40 30 20 10 0 -10

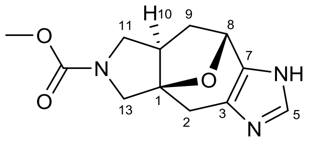
O
N
H
O
NH
N
11
10
9
8
7
13
1
2
3
5

F33

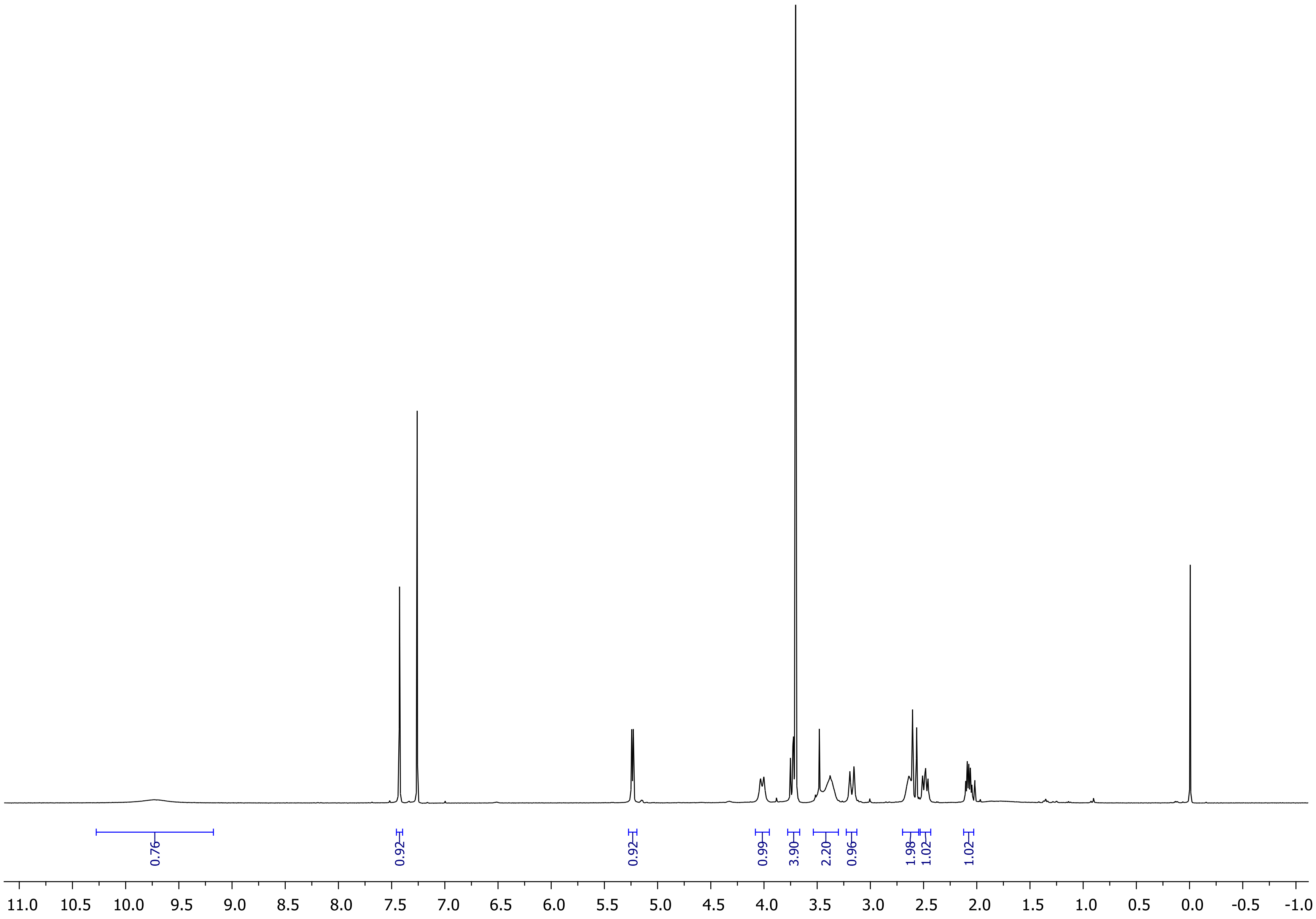
0.76
0.92
0.92
0.99
3.90
2.20
0.96
1.98
1.02
1.02
11.0 10.5 10.0 9.5 9.0 8.5 8.0 7.5 7.0 6.5 6.0 5.5 5.0 4.5 4.0 3.5 3.0 2.5 2.0 1.5 1.0 0.5 0.0 -0.5 -1.0

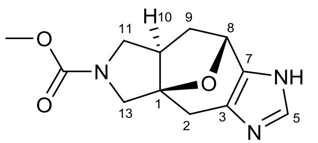
H
10
9
8
11
7
NH
O
N
O
O
13
1
2
3
N
5

F33

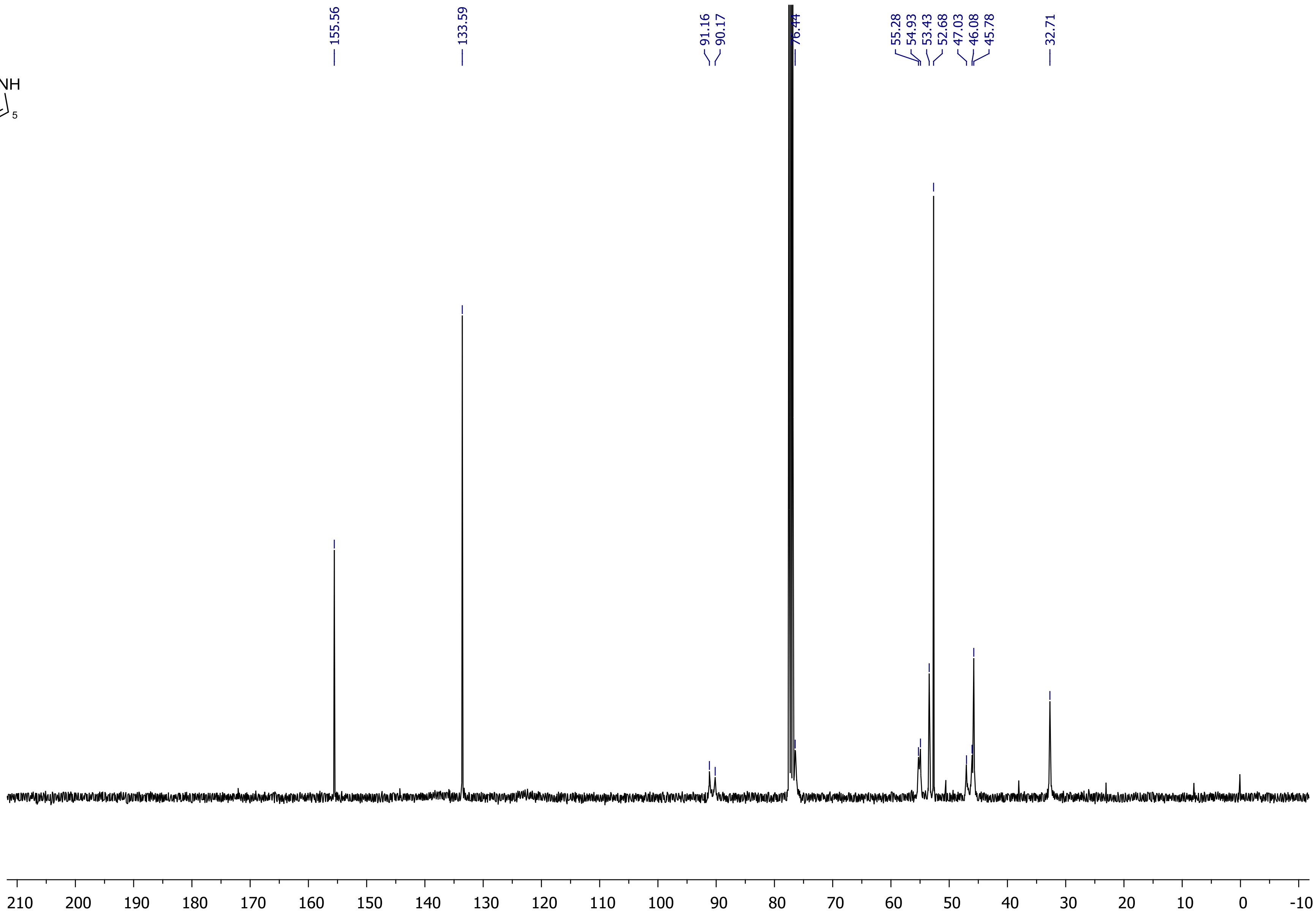
155.56
133.59
91.16
90.17
76.44
55.28
54.93
53.43
52.68
47.03
46.08
45.78
32.71
210
200
190
180
170
160
150
140
130
120
110
100
90
80
70
60
50
40
30
20
10
0
-10

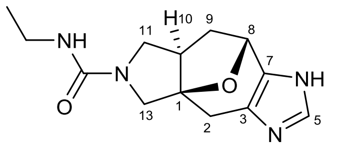
NH
N
O
H10
9
8
7
NH
5
N
3
2
1
13
11
O

F34

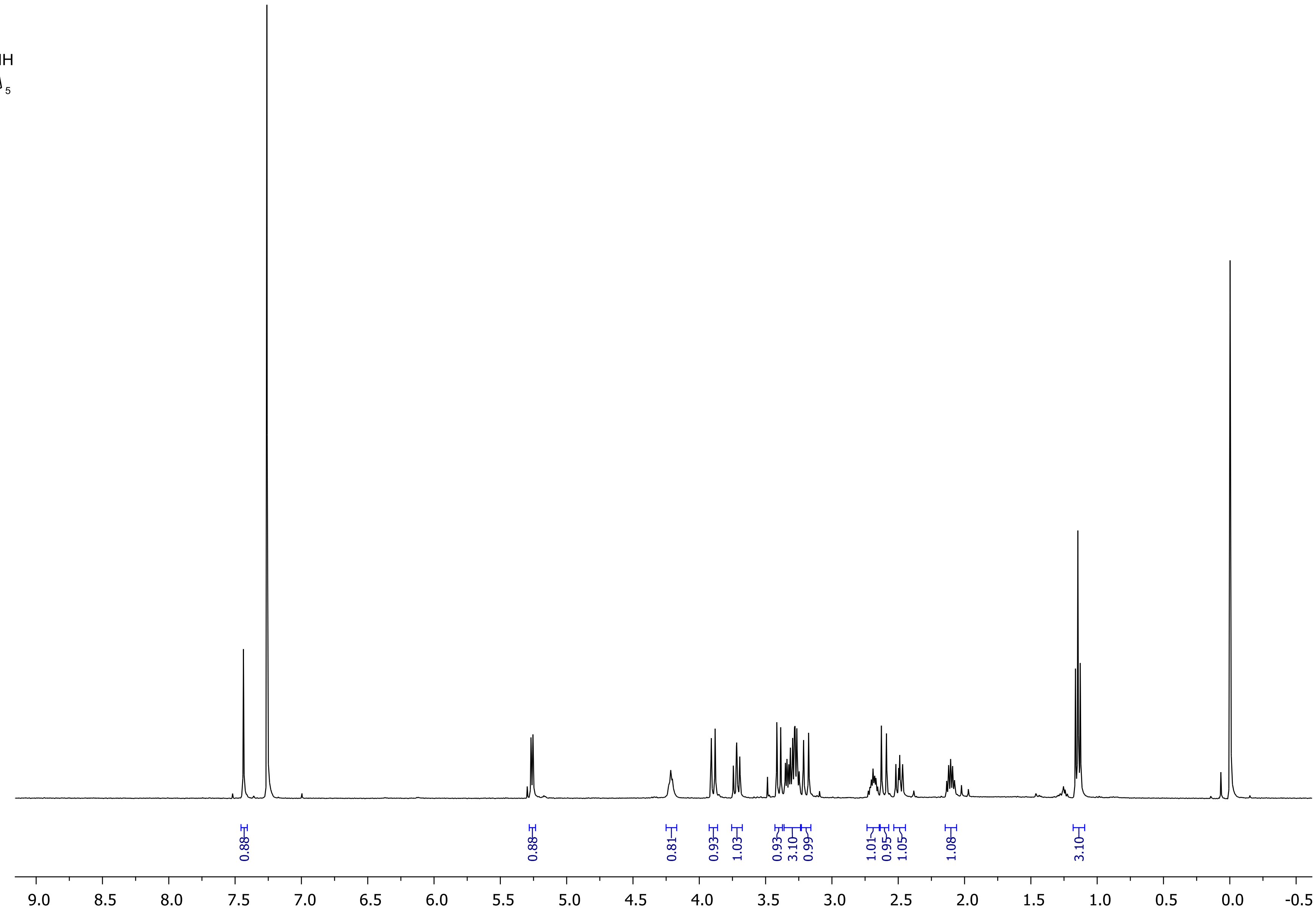
0.88
0.88
0.81
0.93
1.03
0.93
3.10
0.99
1.01
0.95
1.05
1.08
3.10
9.0 8.5 8.0 7.5 7.0 6.5 6.0 5.5 5.0 4.5 4.0 3.5 3.0 2.5 2.0 1.5 1.0 0.5 0.0 -0.5

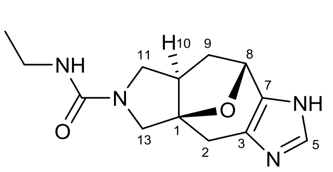
NH
H
10
9
8
11
7
NH
N
O
O
13
1
2
3
N
5

F34

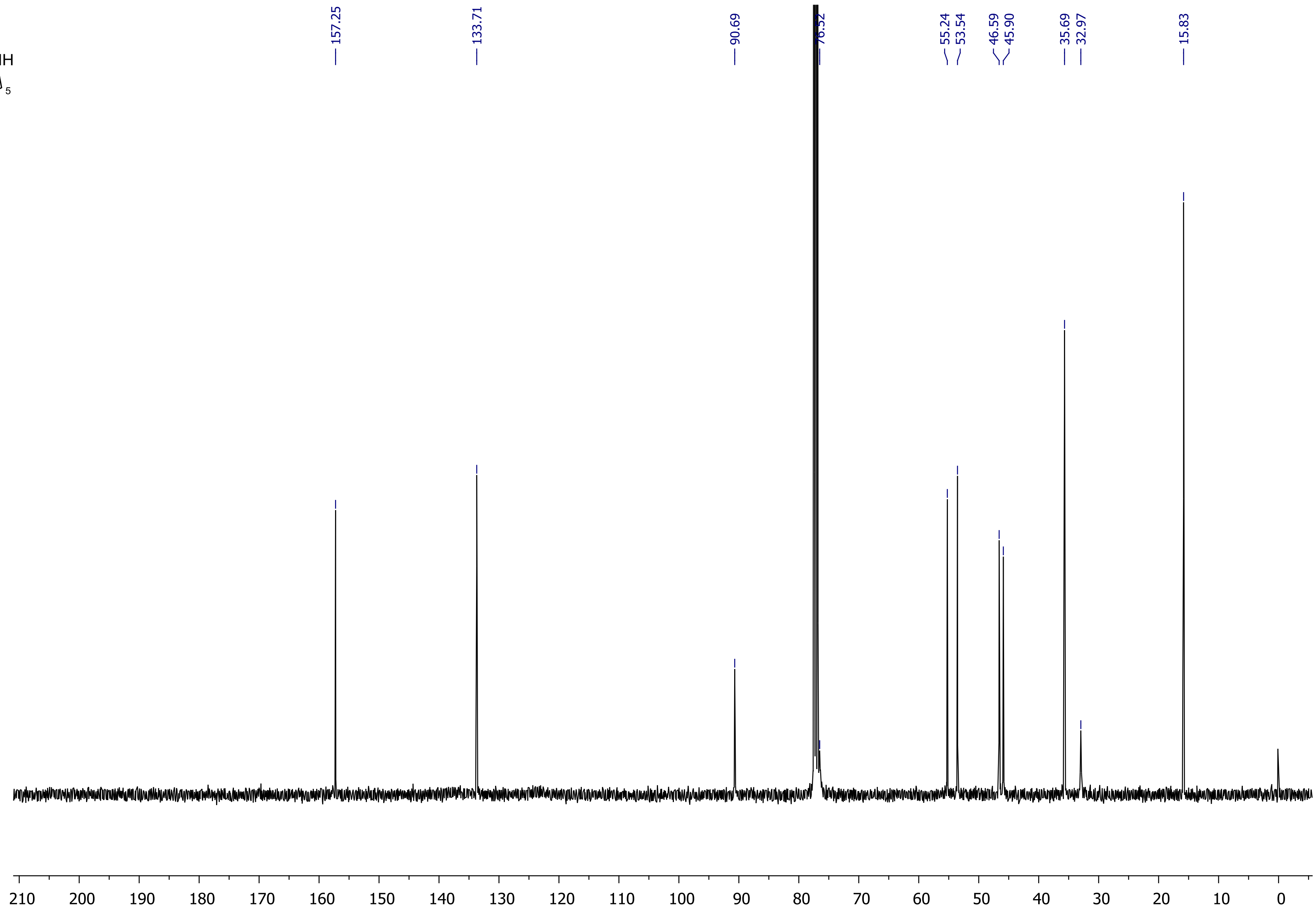
157.25
133.71
90.69
76.52
55.24
53.54
46.59
45.90
35.69
32.97
15.83
210 200 190 180 170 160 150 140 130 120 110 100 90 80 70 60 50 40 30 20 10 0

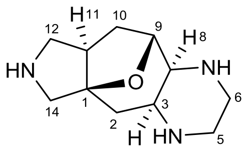
HN
NH
HN
O
H
H
H
1
2
3
5
6
8
9
10
11
12
14

F35

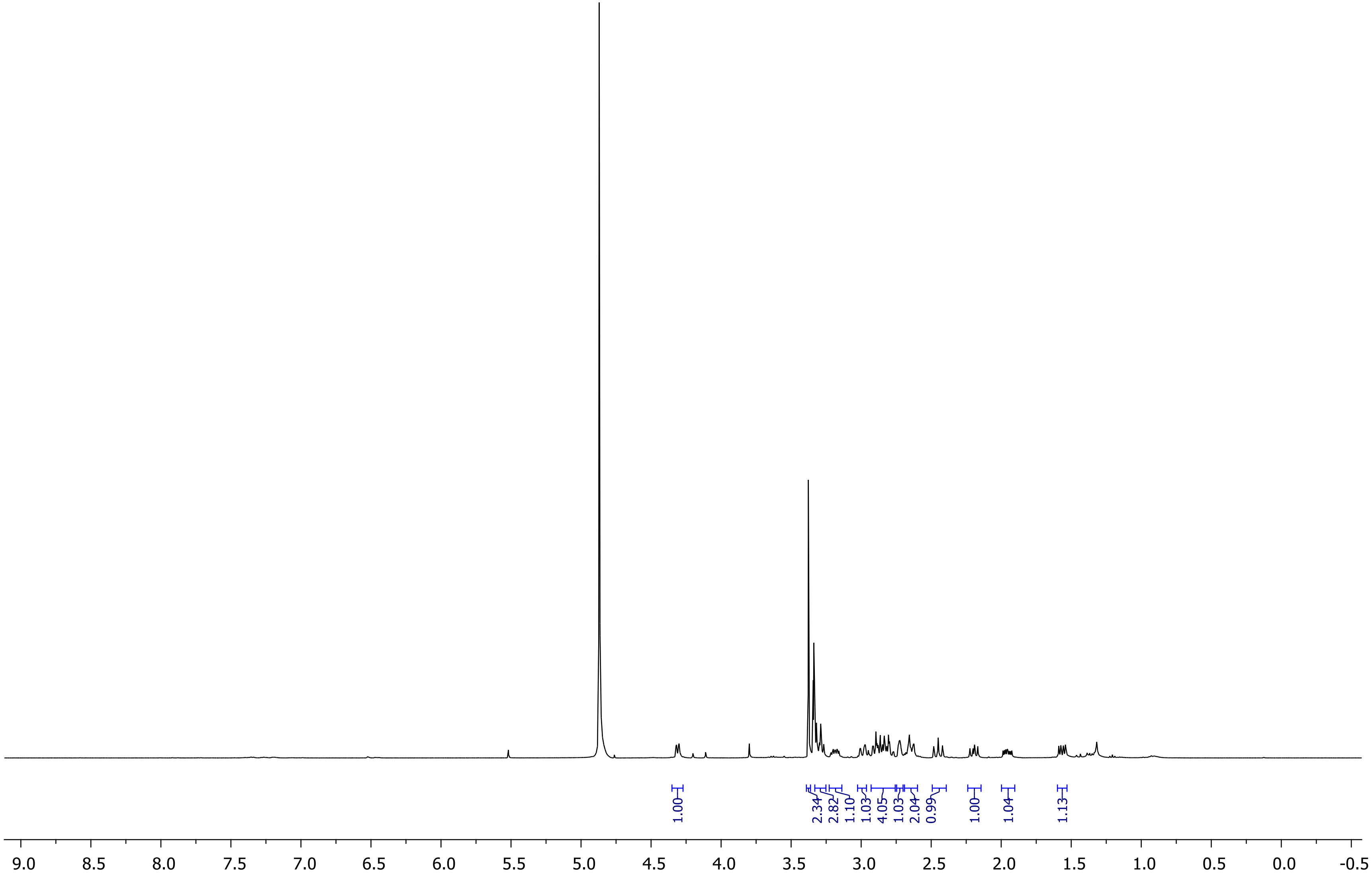
1.00
2.34
2.82
1.10
1.03
4.05
1.03
2.04
0.99
1.00
1.04
1.13
9.0
8.5
8.0
7.5
7.0
6.5
6.0
5.5
5.0
4.5
4.0
3.5
3.0
2.5
2.0
1.5
1.0
0.5
0.0
-0.5

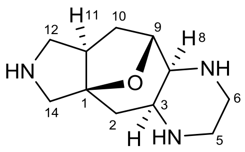

HN
NH
HN
O
H
H
H

F35

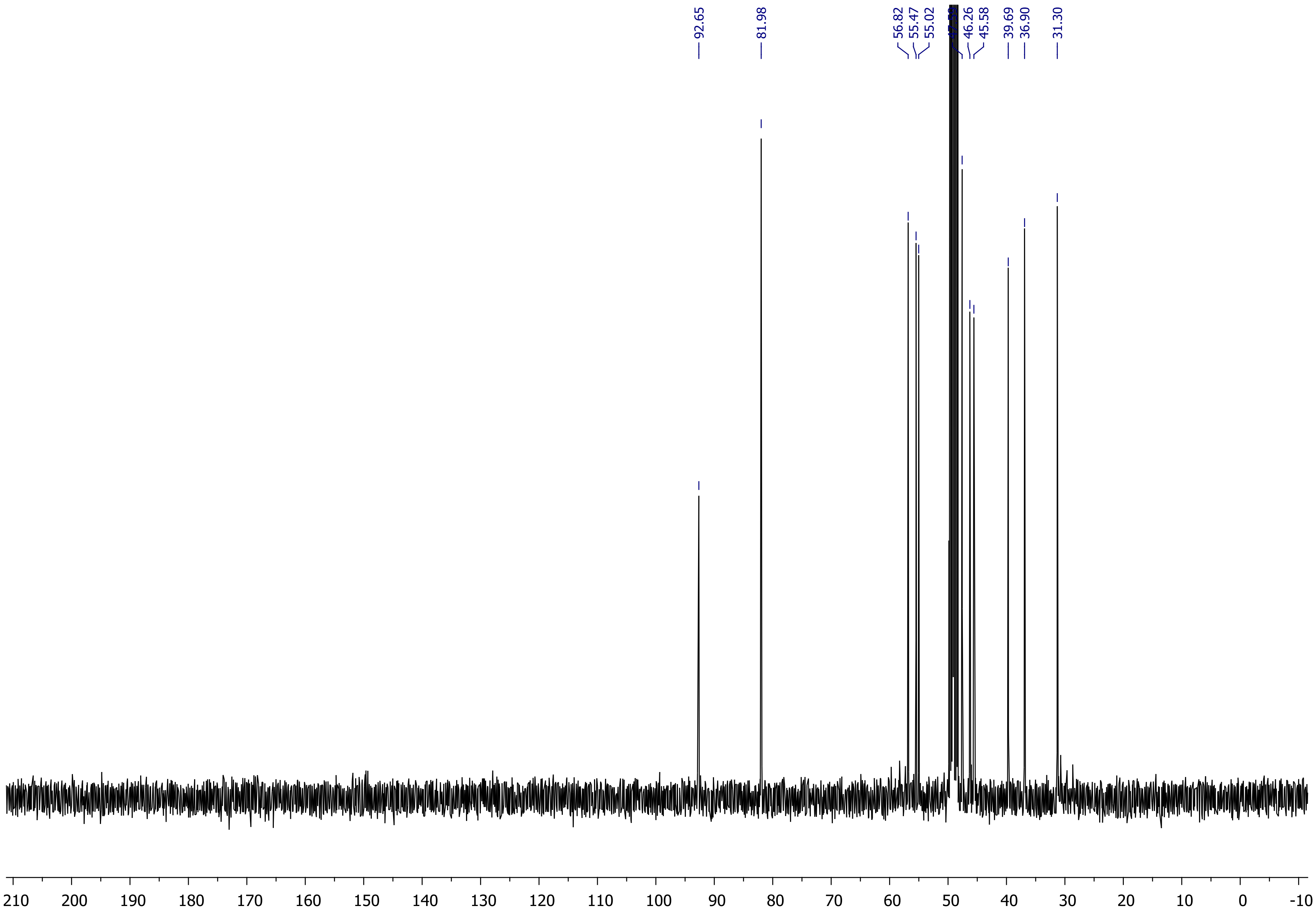

92.65
81.98
56.82
55.47
55.02
46.26
45.58
39.69
36.90
31.30
210
200
190
180
170
160
150
140
130
120
110
100
90
80
70
60
50
40
30
20
10
0
-10

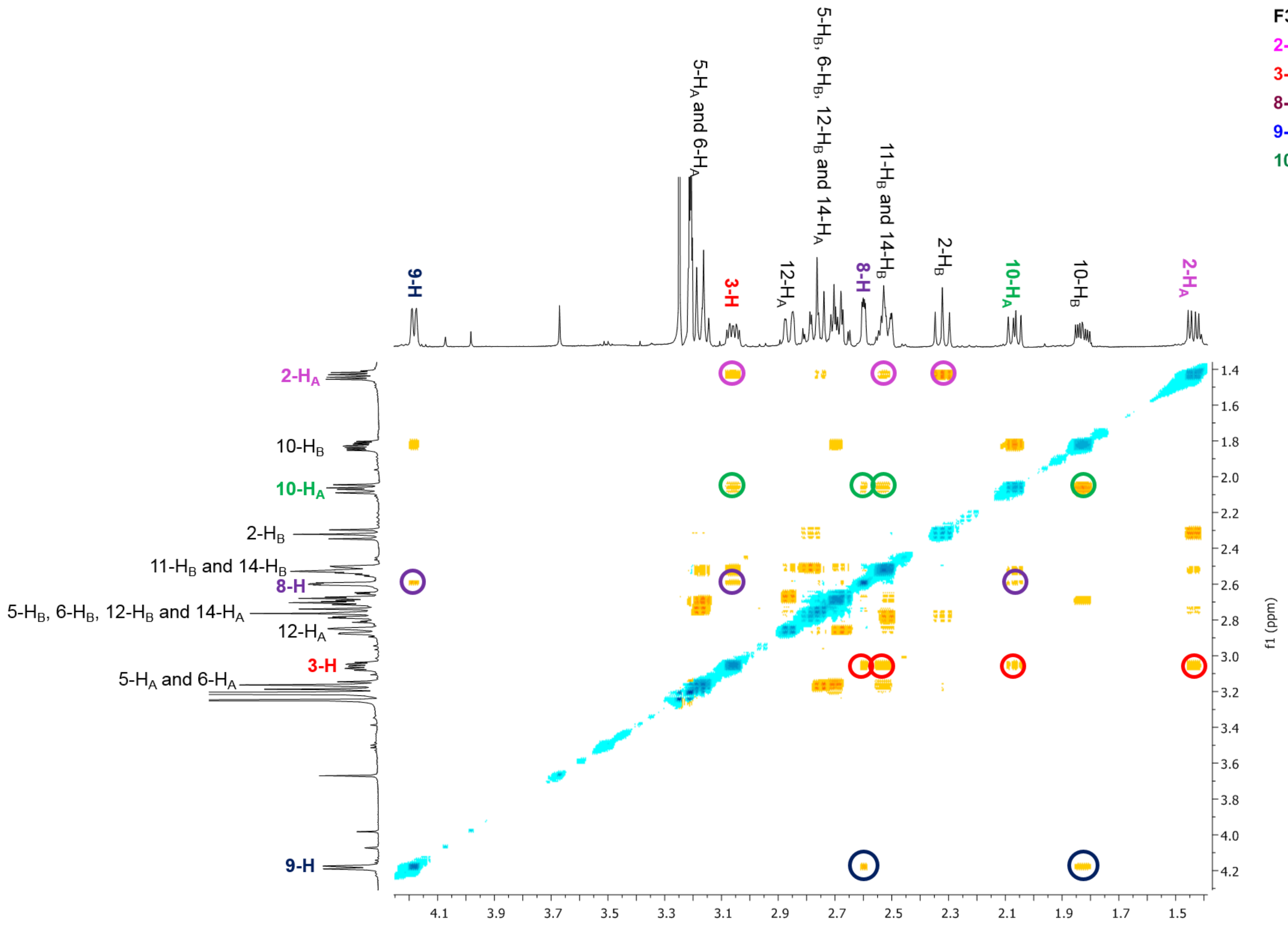

F35 NOESY correlations:

2-H$_A$: 2-H$_B$, 3-H, 11-H

3-H: 2-H$_A$, 8-H, 10-H$_A$, 11-H

8-H: 3-H, 9-H, 10-H$_A$

9-H: 8-H and 10-H$_B$

10-H$_A$: 3-H, 8-H, 10-H$_B$, 11-H

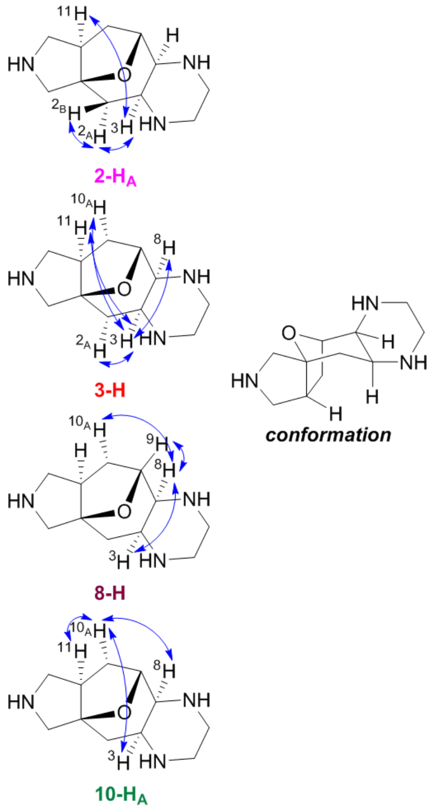

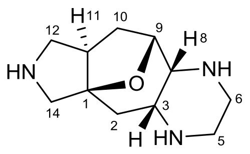
12
11
10
9
8
HN
NH
O
6
1
3
14
2
5
HN

F36

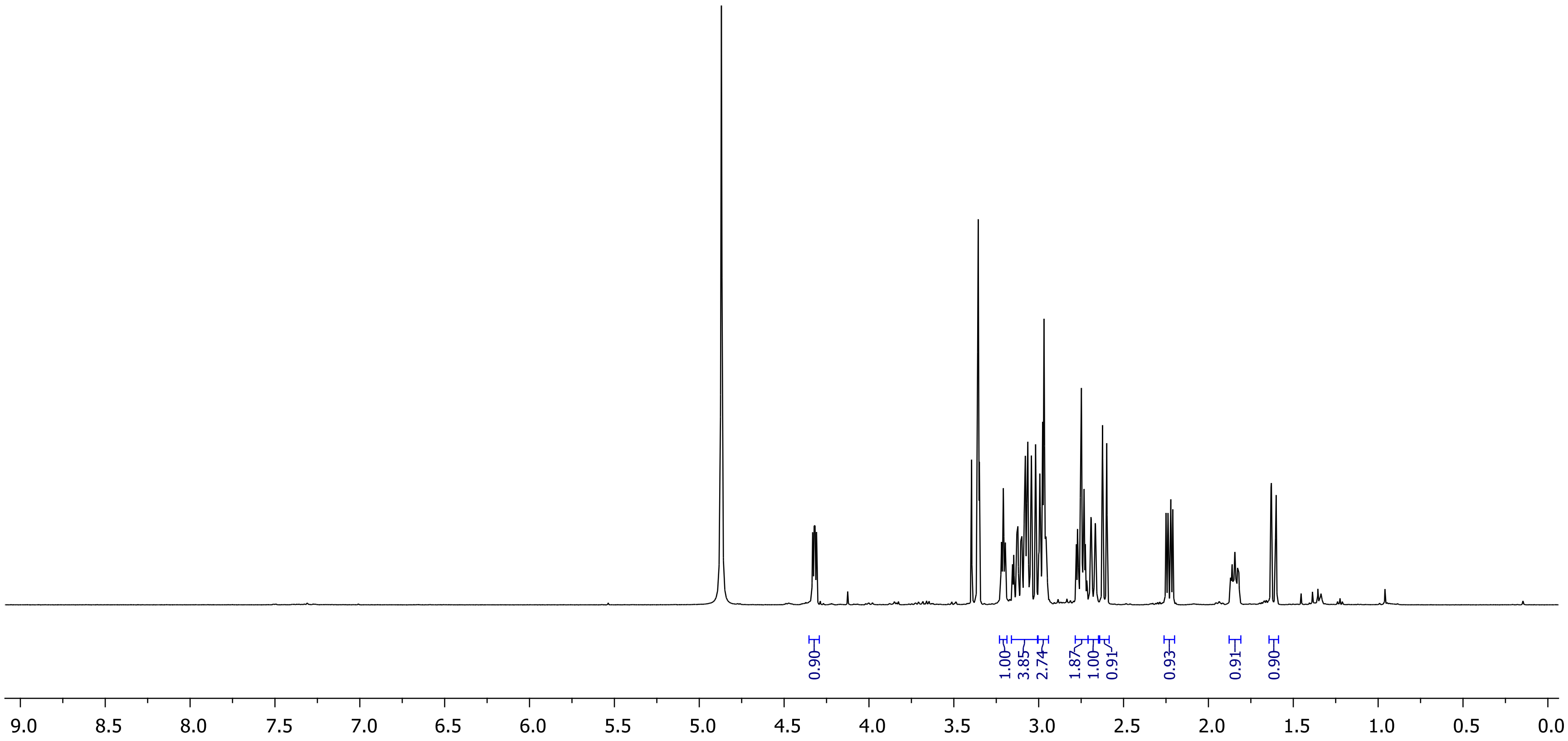
0.90
1.00
3.85
2.74
1.87
1.00
0.91
0.93
0.91
0.90
9.0
8.5
8.0
7.5
7.0
6.5
6.0
5.5
5.0
4.5
4.0
3.5
3.0
2.5
2.0
1.5
1.0
0.5
0.0

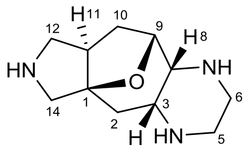

F36

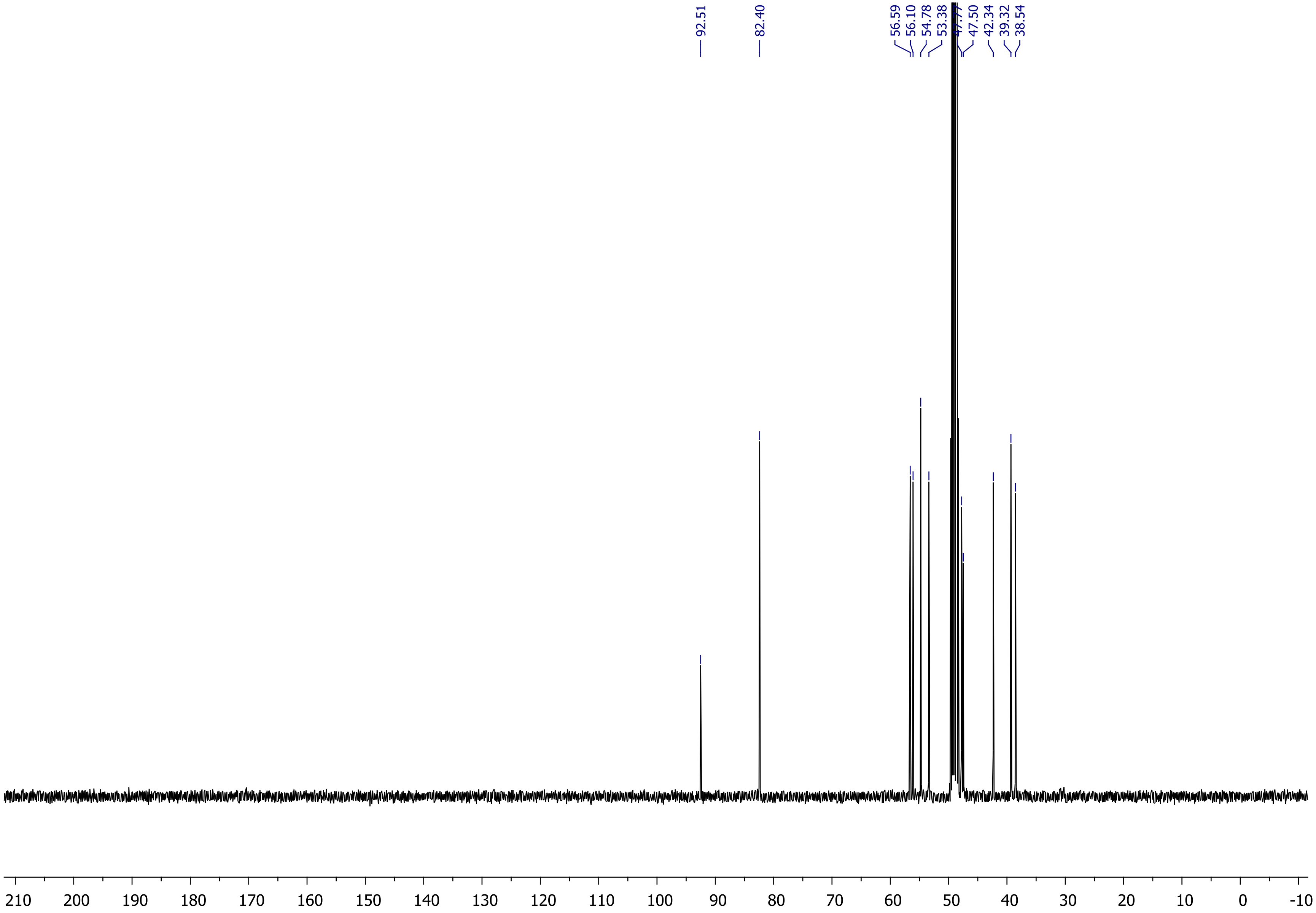
92.51
82.40
56.59
56.10
54.78
53.38
47.77
47.50
42.34
39.32
38.54
210
200
190
180
170
160
150
140
130
120
110
100
90
80
70
60
50
40
30
20
10
0
-10

F36 NOESY correlations:

2-H_A: 2-H_B, 3-H, 11-H, 14-H_A

2-H_B: 2-H_A, 3-H, 8-H, 14-H_A, 14-H_B

3-H: 2-H_A, 5-H_B, 8-H

9-H: 8-H and 10-H_B

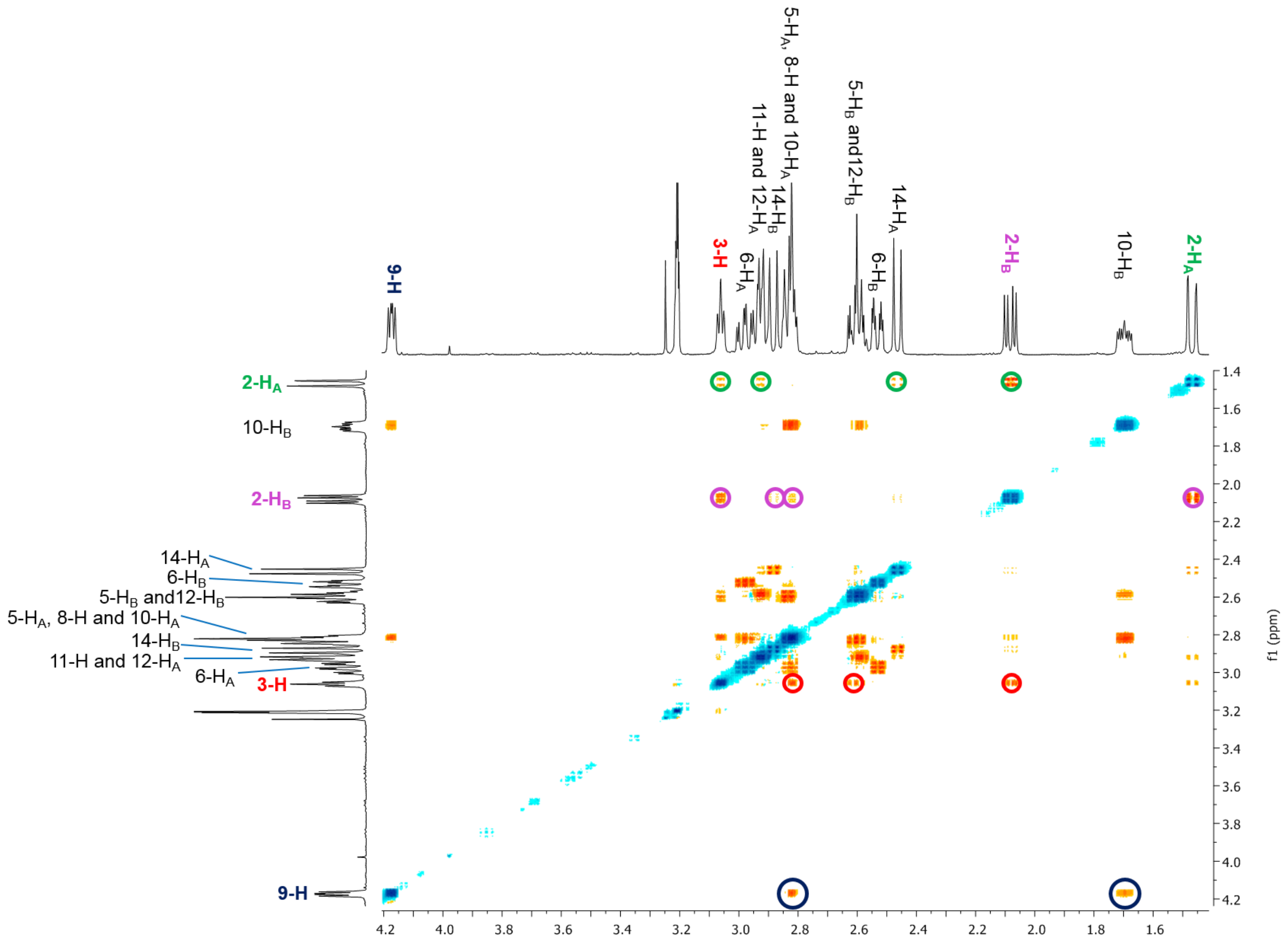

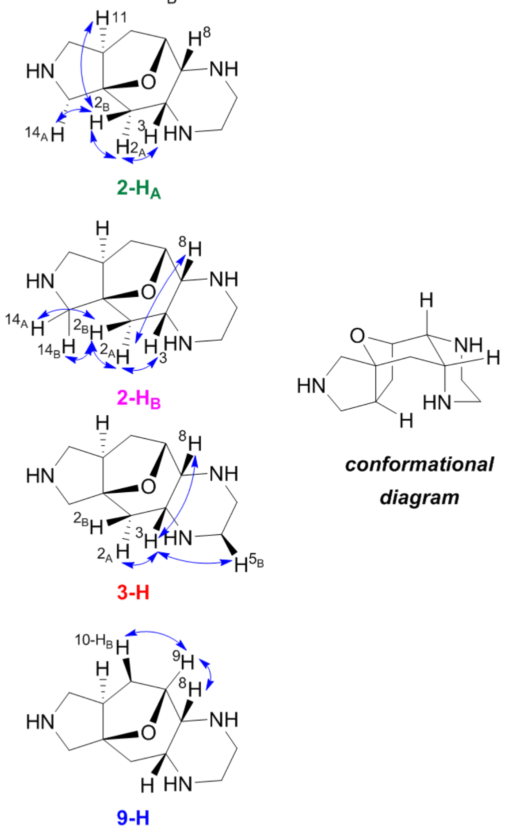

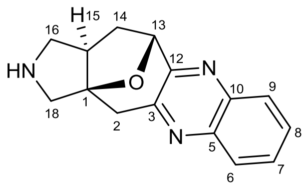

F37

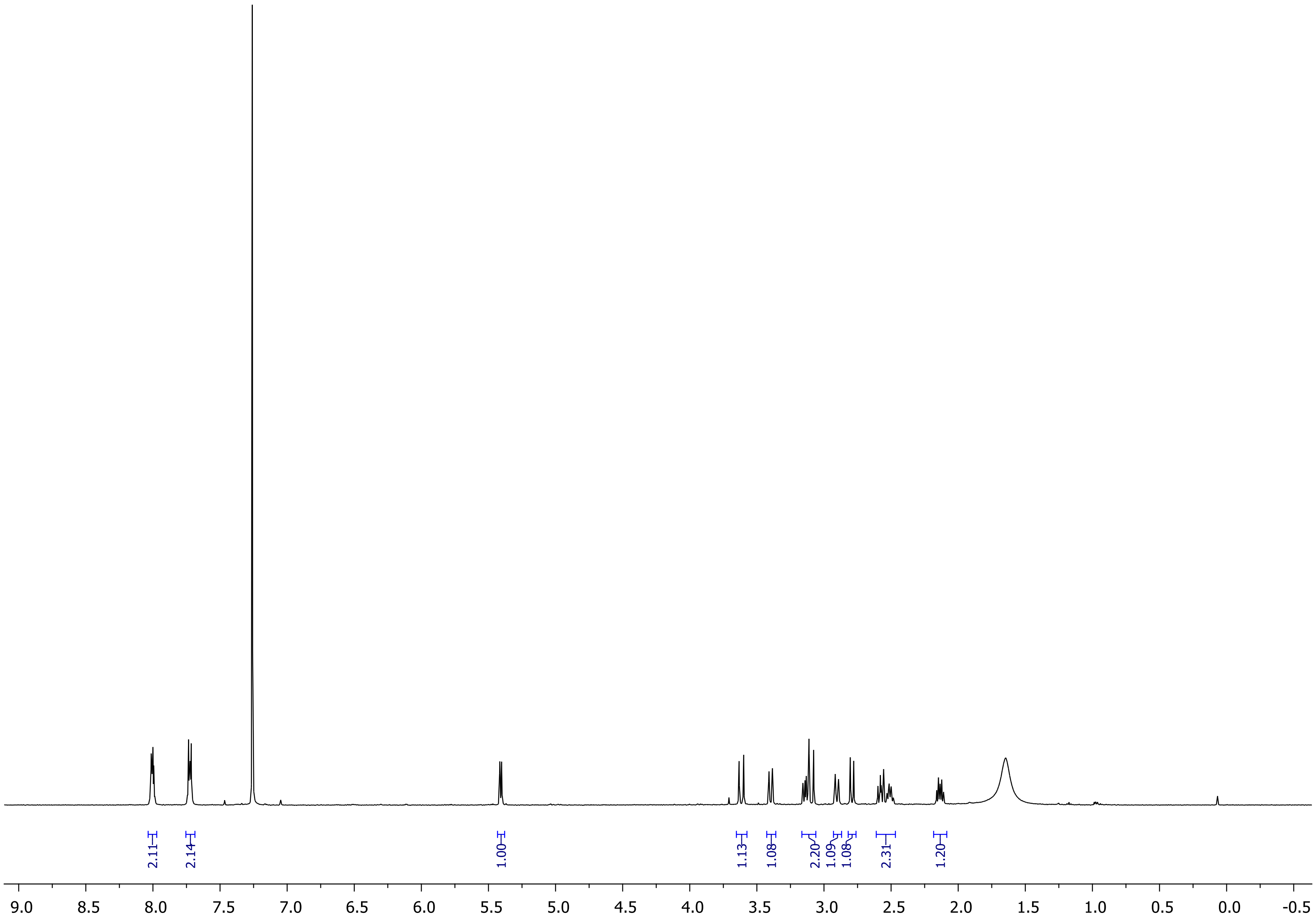
2.11
2.14
1.00
1.13
1.08
2.20
1.09
1.08
2.31
1.20
9.0
8.5
8.0
7.5
7.0
6.5
6.0
5.5
5.0
4.5
4.0
3.5
3.0
2.5
2.0
1.5
1.0
0.5
0.0
-0.5

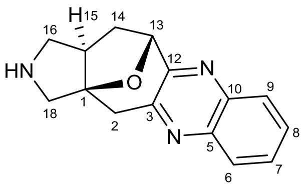

F37

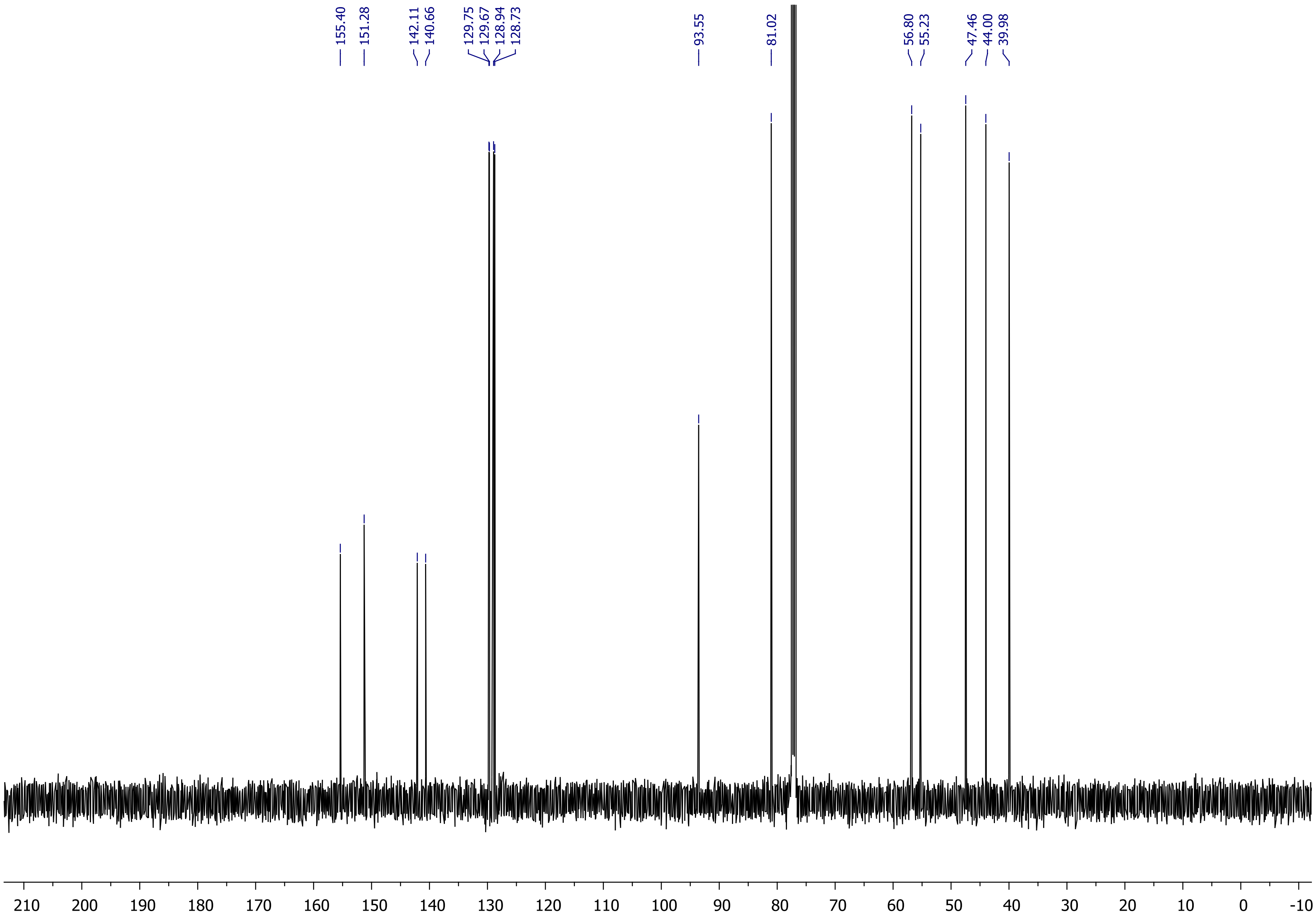

155.40
151.28
142.11
140.66
129.75
129.67
128.94
128.73
93.55
81.02
56.80
55.23
47.46
44.00
39.98
210 200 190 180 170 160 150 140 130 120 110 100 90 80 70 60 50 40 30 20 10 0 -10

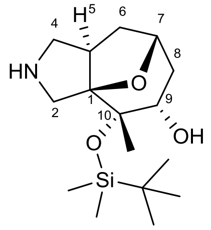

S49

carried forward crude

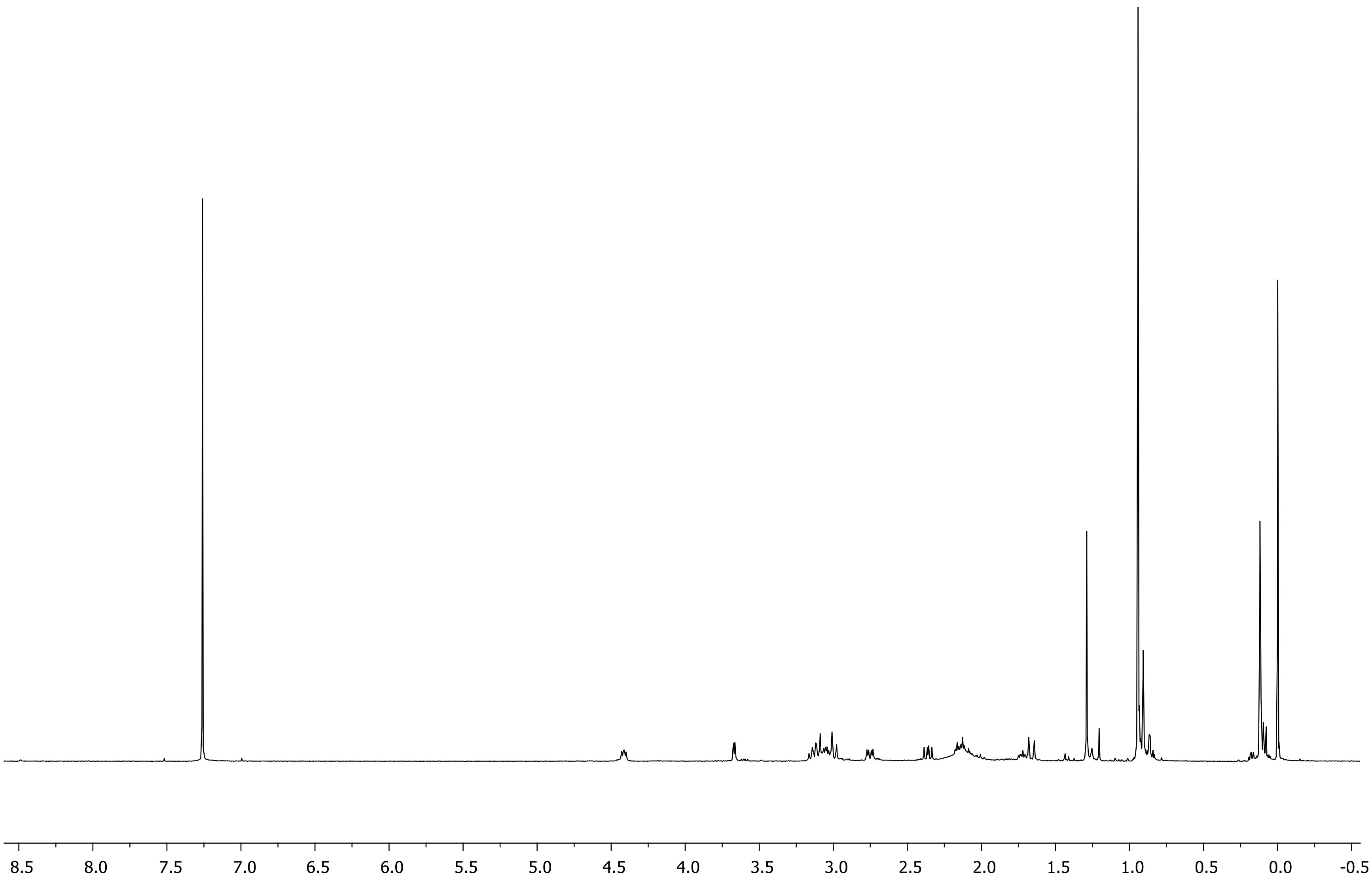

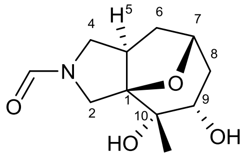

H
5
4
6
7
8
N
O
O
1
2
10
9
HO
OH

F38

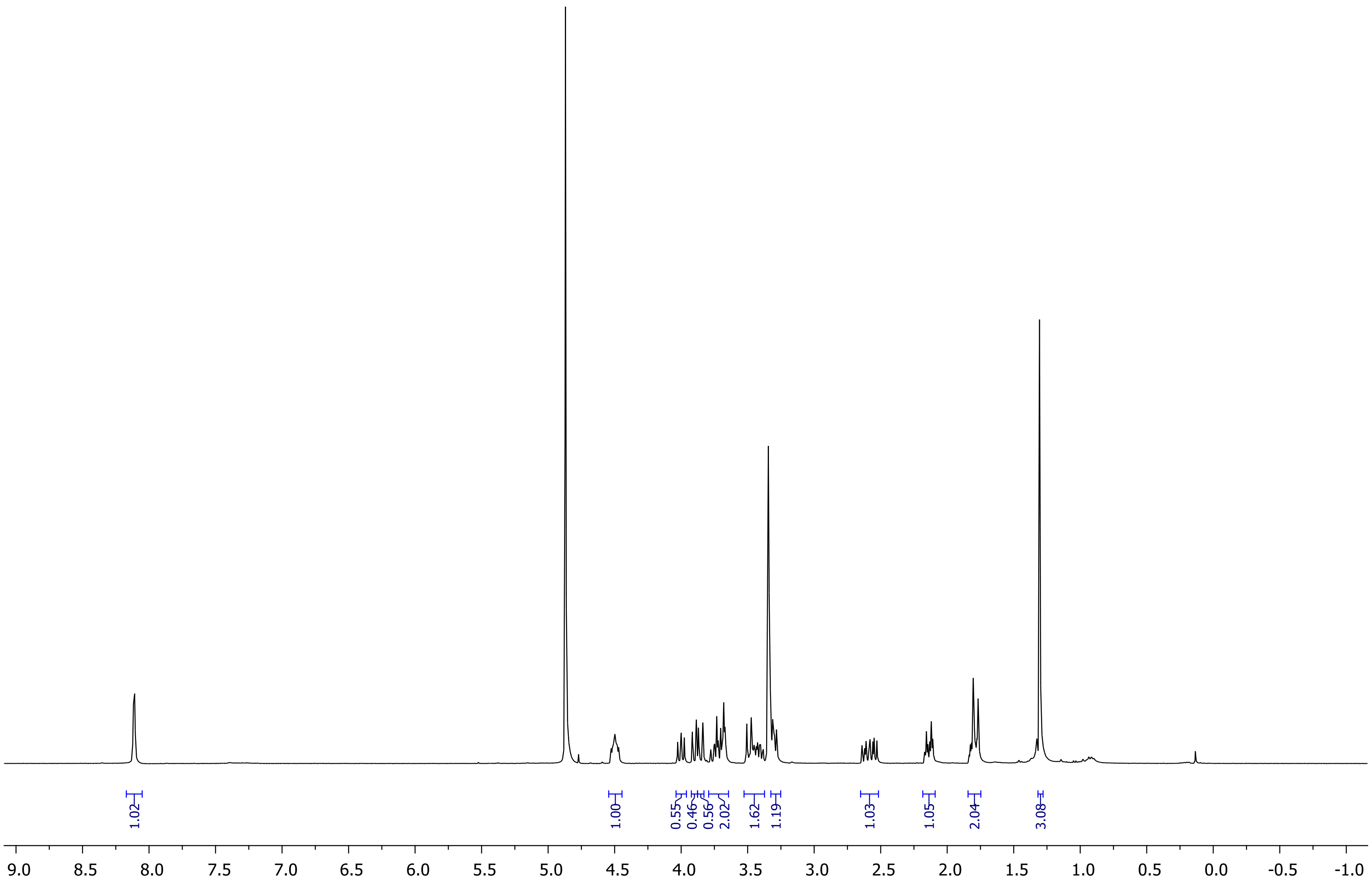

1.02
1.00
0.55
0.46
0.56
2.02
1.62
1.19
1.03
1.05
2.04
3.08
9.0
8.5
8.0
7.5
7.0
6.5
6.0
5.5
5.0
4.5
4.0
3.5
3.0
2.5
2.0
1.5
1.0
0.5
0.0
-0.5
-1.0

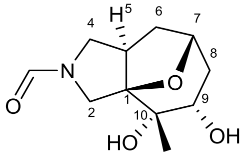
H
5
6
7
4
8
N
O
O
1
9
2
10
HO
OH

F38

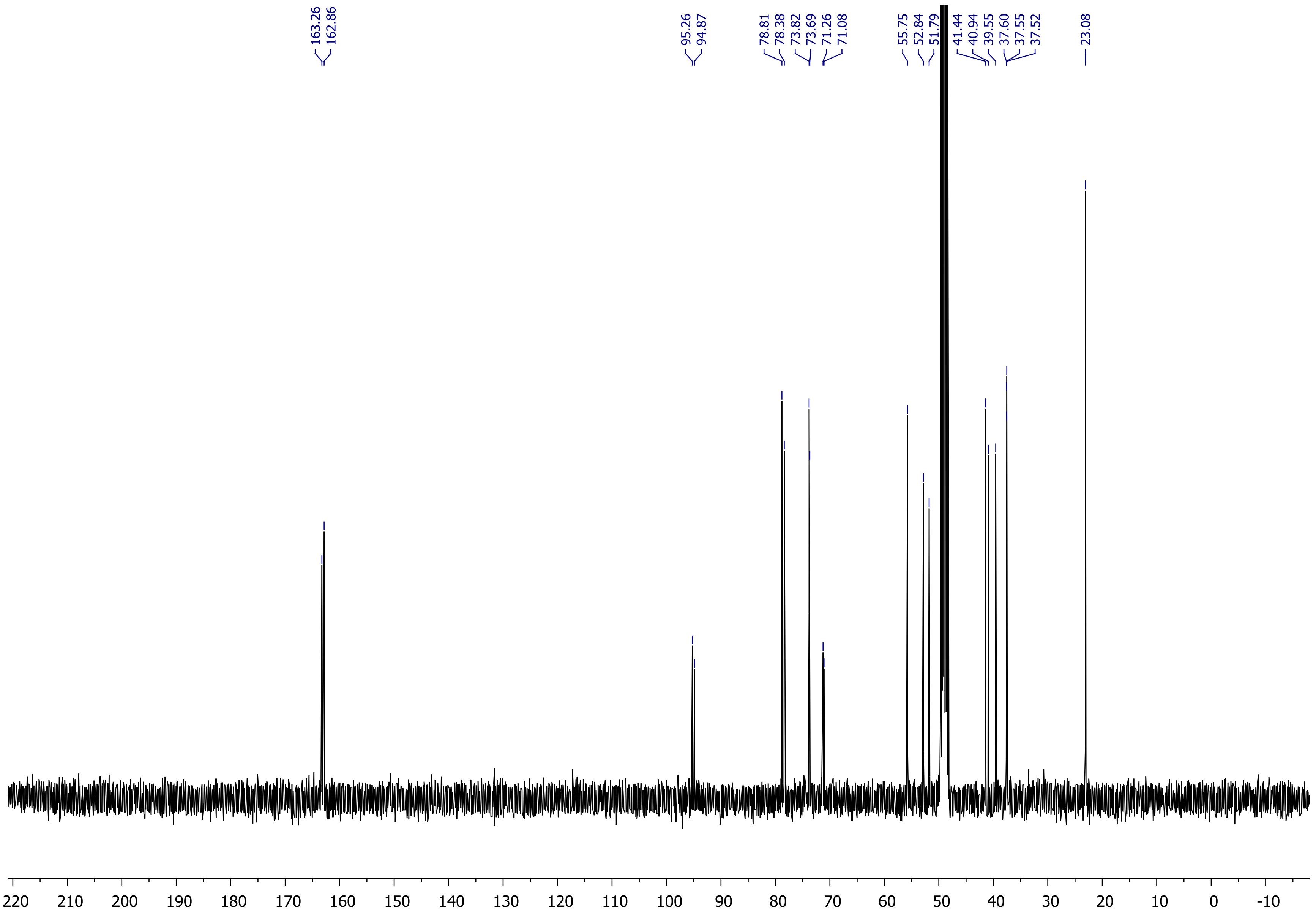
163.26
162.86
95.26
94.87
78.81
78.38
73.82
73.69
71.26
71.08
55.75
52.84
51.79
41.44
40.94
39.55
37.60
37.55
37.52
23.08
220
210
200
190
180
170
160
150
140
130
120
110
100
90
80
70
60
50
40
30
20
10
0
-10

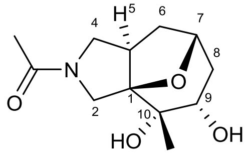

H5
4
6
7
8
N
O
1
2
10
9
HO
OH

F39

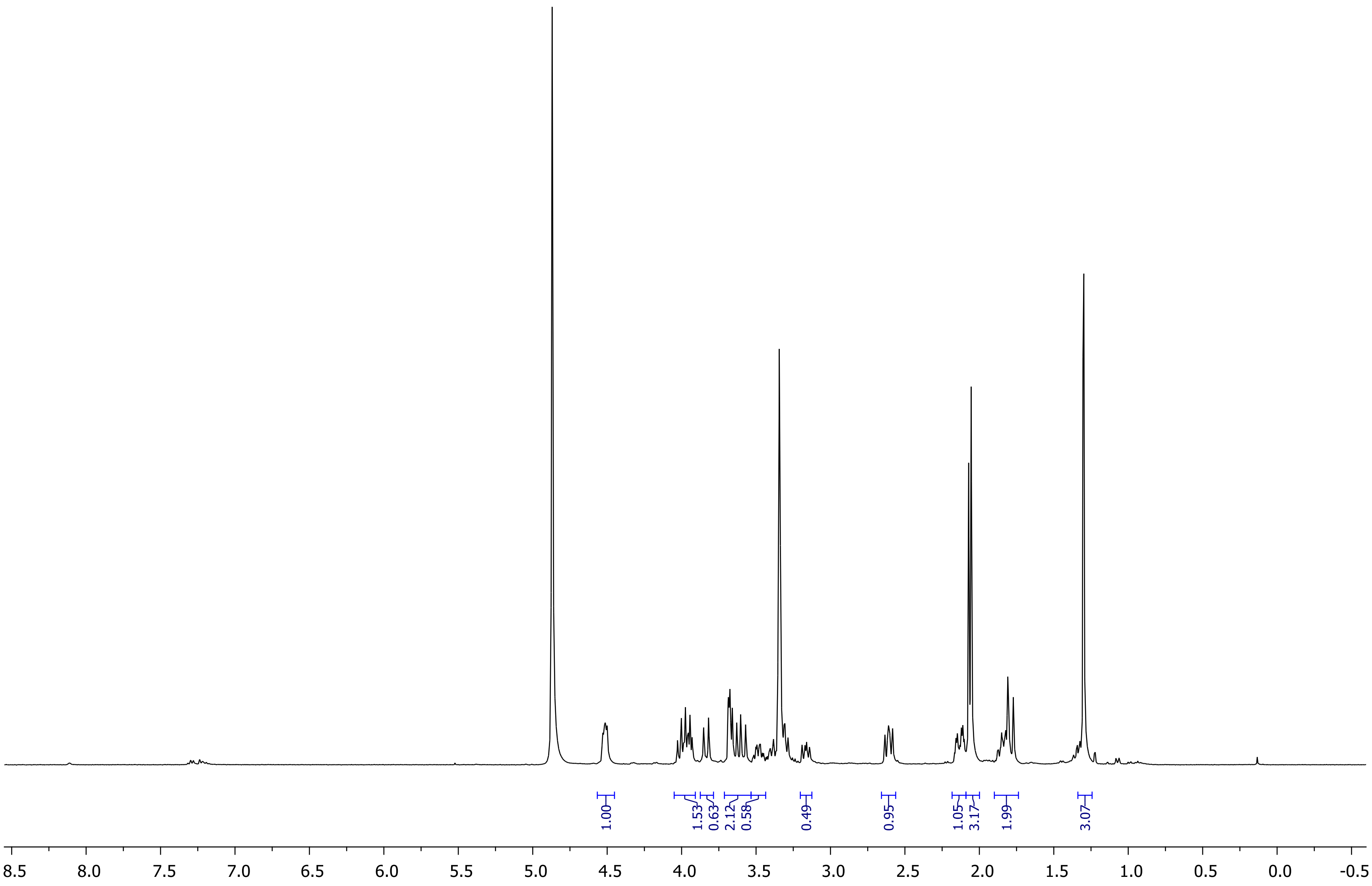

1.00
1.53
0.63
2.12
0.58
0.49
0.95
1.05
3.17
1.99
3.07
8.5
8.0
7.5
7.0
6.5
6.0
5.5
5.0
4.5
4.0
3.5
3.0
2.5
2.0
1.5
1.0
0.5
0.0
-0.5

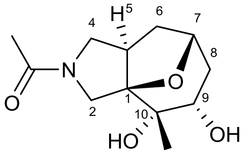

H5
4
6
7
8
N
O
O
2
1
10
9
HO
OH

F39

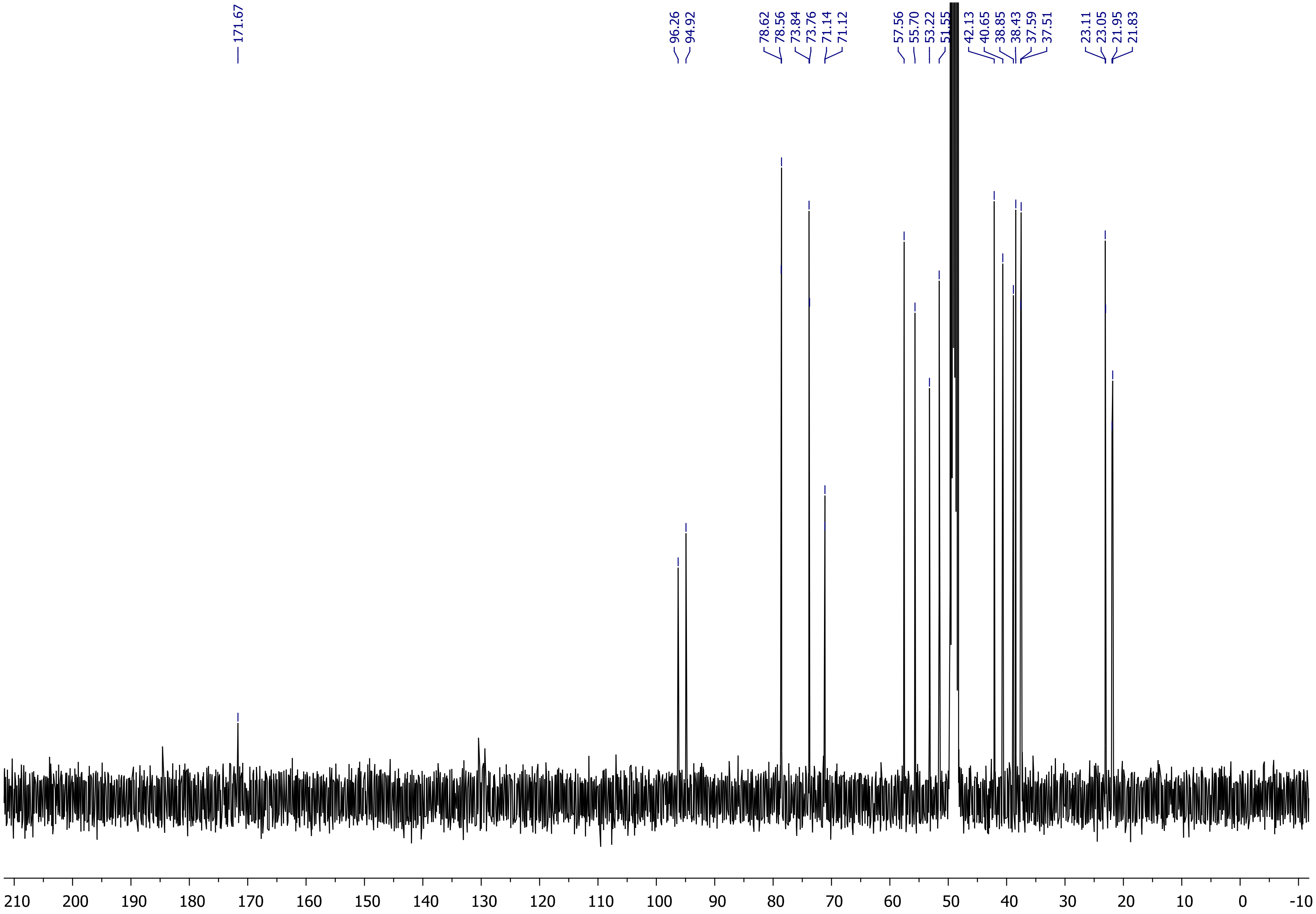

171.67
96.26
94.92
78.62
78.56
73.84
73.76
71.14
71.12
57.56
55.70
53.22
51.55
42.13
40.65
38.85
38.43
37.59
37.51
23.11
23.05
21.95
21.83
210 200 190 180 170 160 150 140 130 120 110 100 90 80 70 60 50 40 30 20 10 0 -10

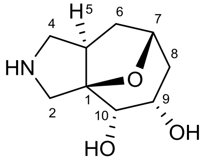
H 5
4
6
7
8
HN
O
1
2
10
9
OH
HO

F40

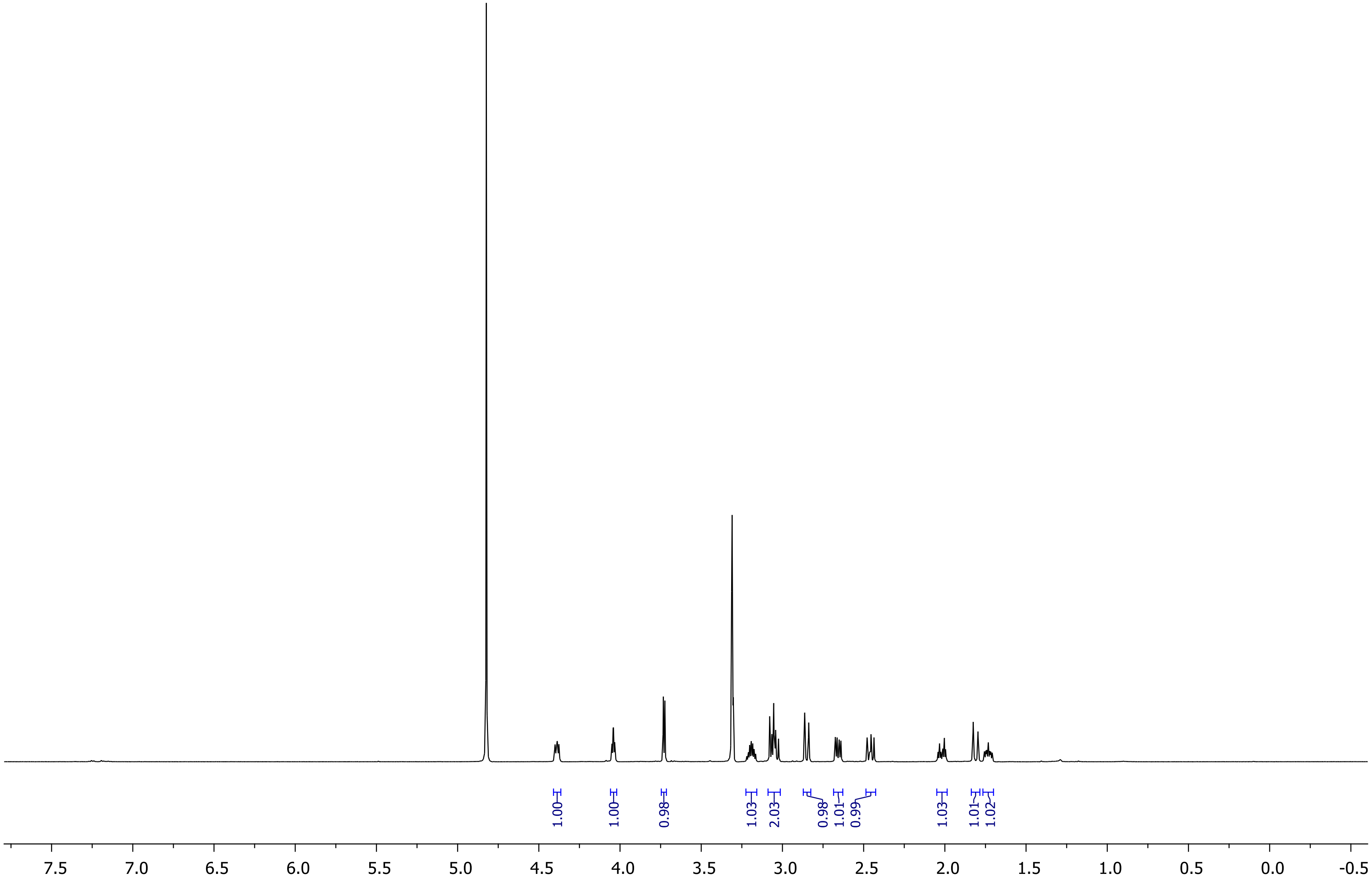
1.00
1.00
0.98
1.03
2.03
0.98
1.01
0.99
1.03
1.01
1.02
7.5
7.0
6.5
6.0
5.5
5.0
4.5
4.0
3.5
3.0
2.5
2.0
1.5
1.0
0.5
0.0
-0.5

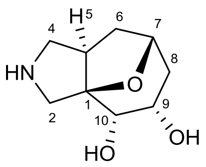
HN
O
OH
HO

F40

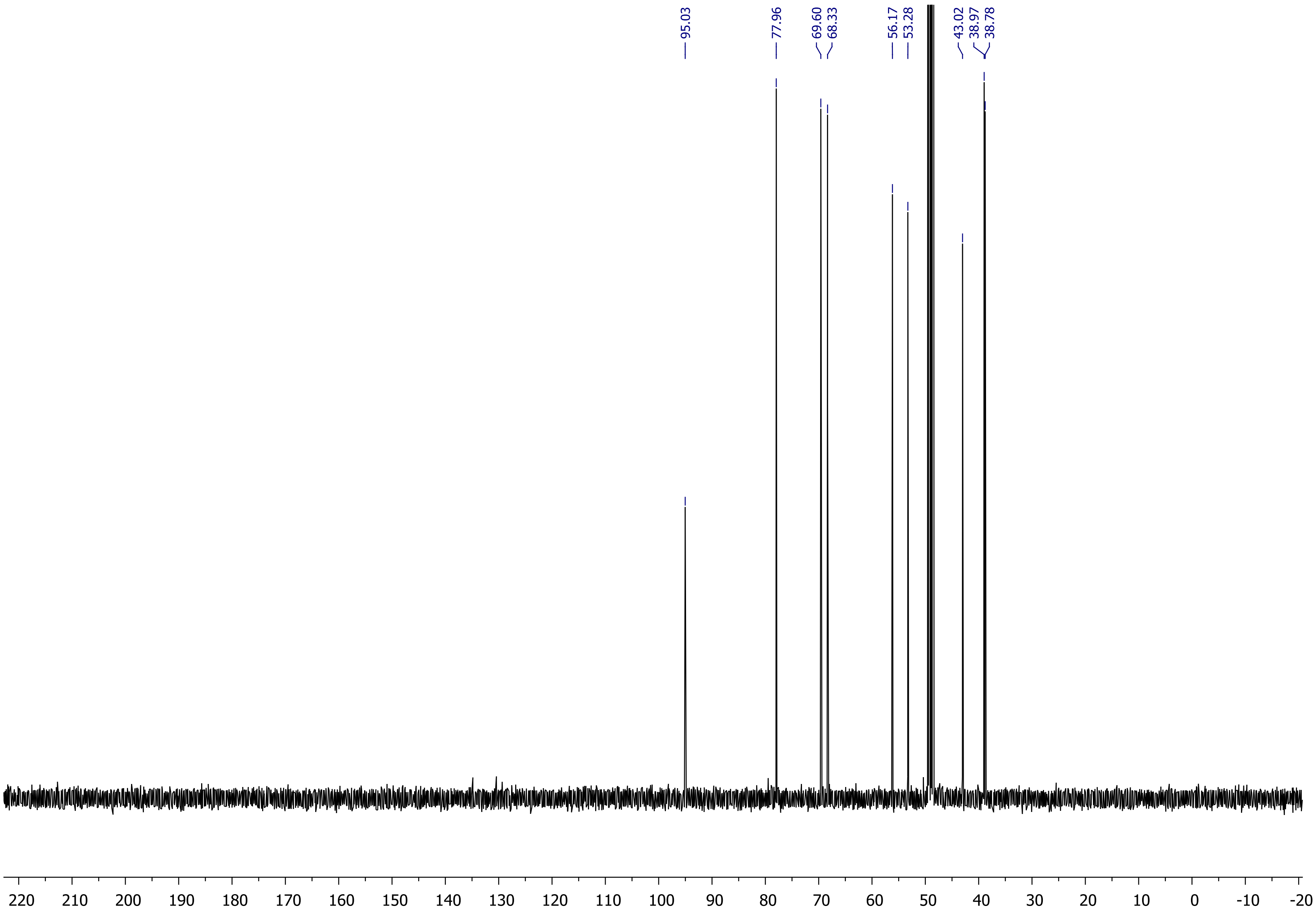
95.03
77.96
69.60
68.33
56.17
53.28
43.02
38.97
38.78
220 210 200 190 180 170 160 150 140 130 120 110 100 90 80 70 60 50 40 30 20 10 0 -10 -20

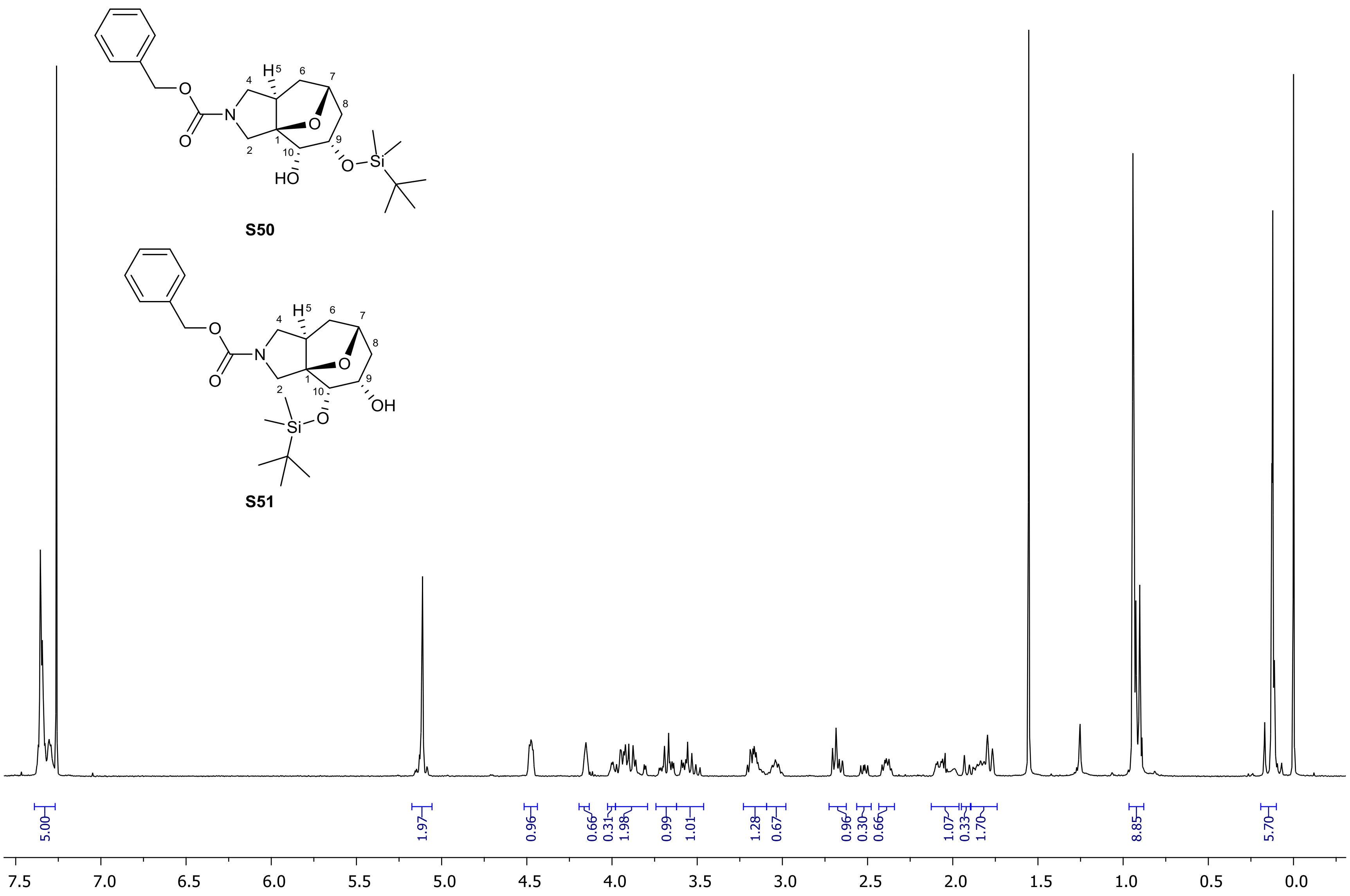

S50
S51
5.00
1.97
0.96
0.66
0.31
1.98
0.99
1.01
1.28
0.67
0.96
0.30
0.66
1.07
0.33
1.70
8.85
5.70
7.5 7.0 6.5 6.0 5.5 5.0 4.5 4.0 3.5 3.0 2.5 2.0 1.5 1.0 0.5 0.0

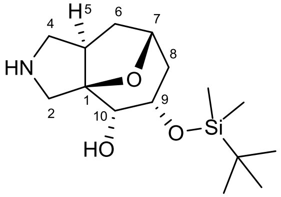
HN
HO
O
Si
H5
1
2
4
6
7
8
9
10

S52

+

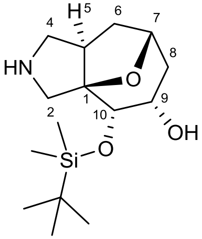
HN
OH
O
Si
H5
1
2
4
6
7
8
9
10

S53

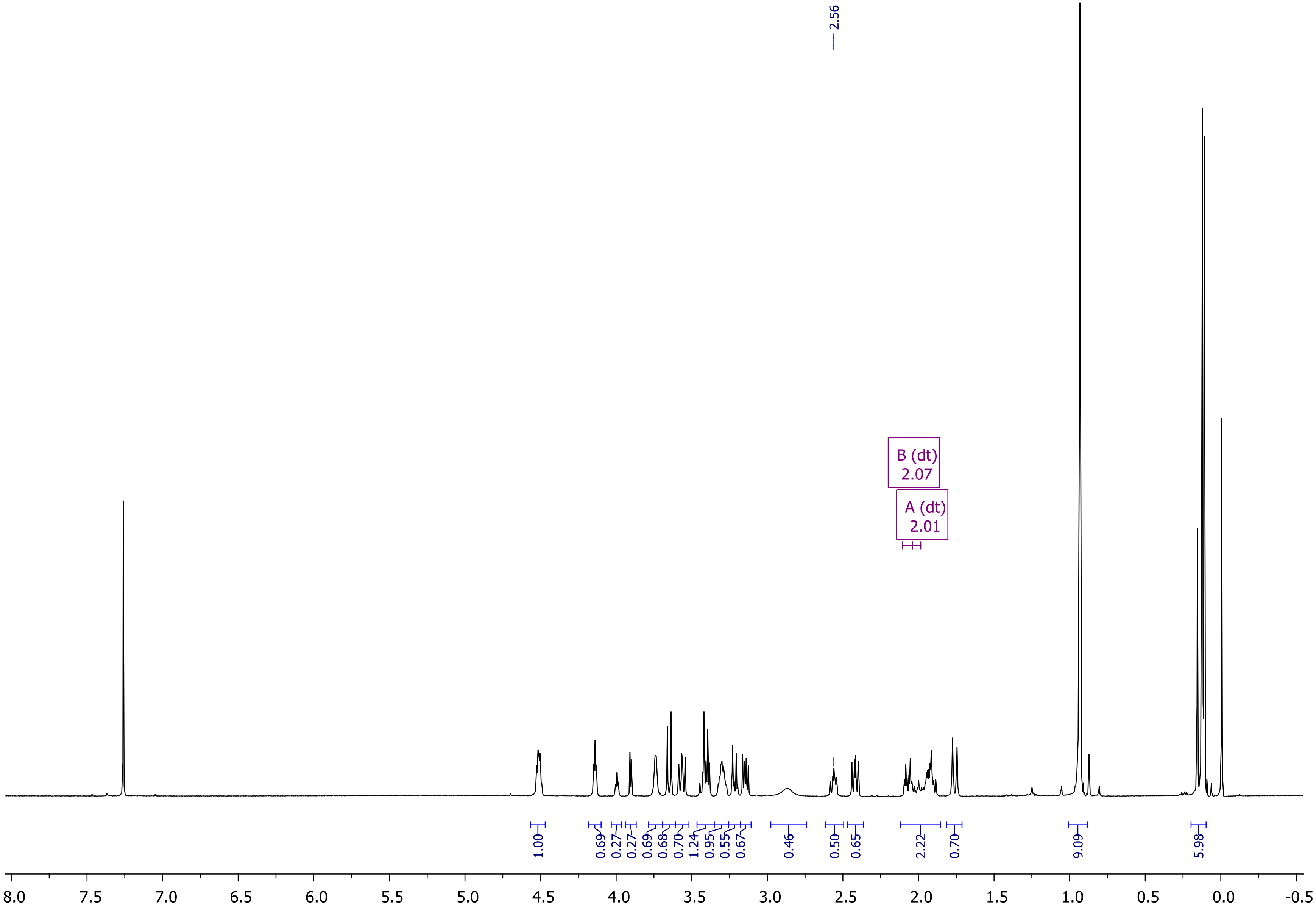
2.56
B (dt)
2.07
A (dt)
2.01
1.00
0.69
0.27
0.27
0.69
0.68
0.70
1.24
0.95
0.55
0.67
0.46
0.50
0.65
2.22
0.70
9.09
5.98
8.0
7.5
7.0
6.5
6.0
5.5
5.0
4.5
4.0
3.5
3.0
2.5
2.0
1.5
1.0
0.5
0.0
-0.5

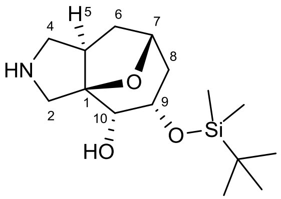

HN
O
HO
O
Si
H
1
2
4
5
6
7
8
9
10

S52

+

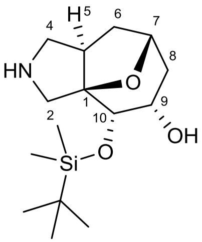

HN
O
OH
O
Si
H
1
2
4
5
6
7
8
9
10

S53

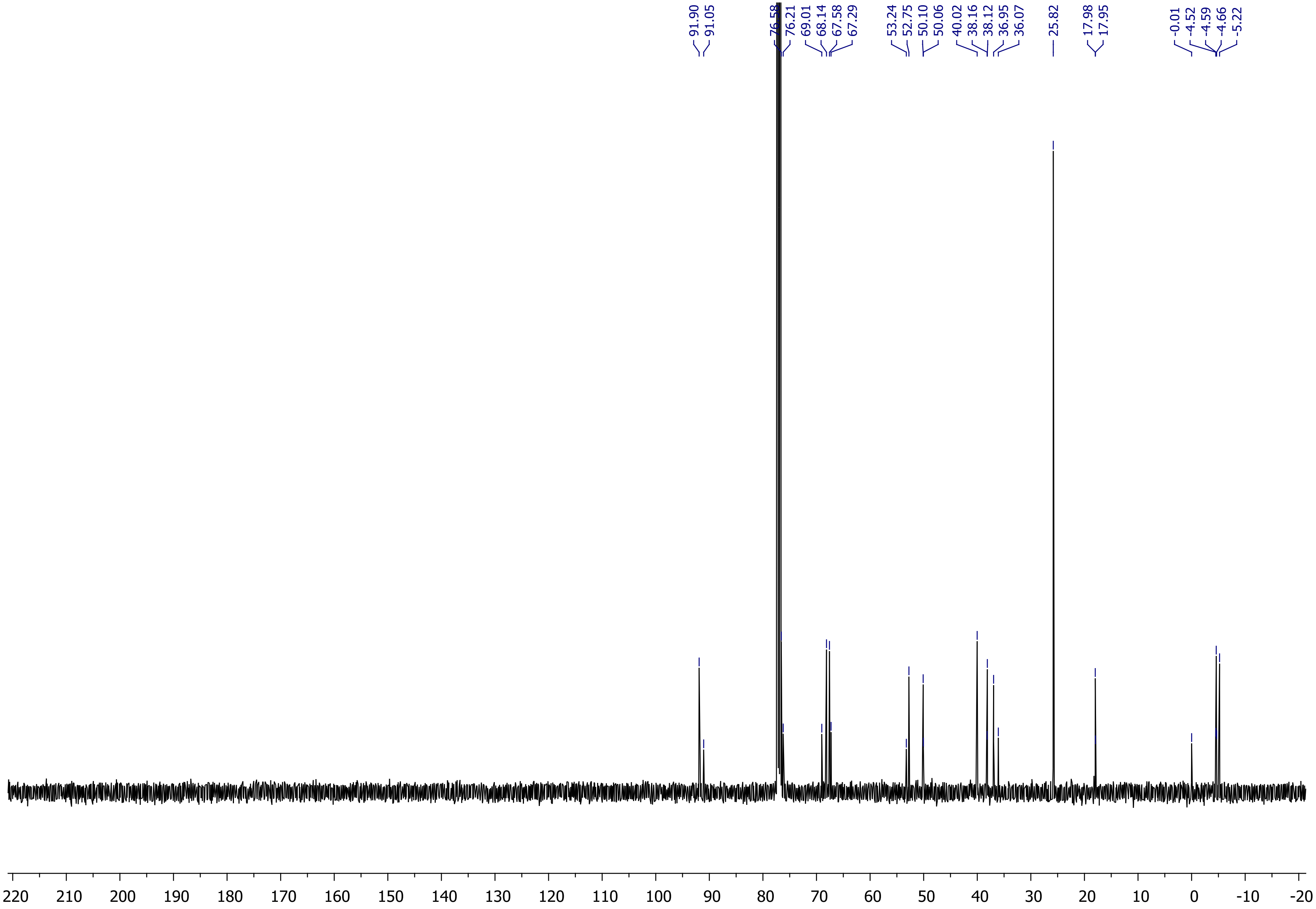

91.90
91.05
76.58
76.21
69.01
68.14
67.58
67.29
53.24
52.75
50.10
50.06
40.02
38.16
38.12
36.95
36.07
25.82
17.98
17.95
-0.01
-4.52
-4.59
-4.66
-5.22
220 210 200 190 180 170 160 150 140 130 120 110 100 90 80 70 60 50 40 30 20 10 0 -10 -20

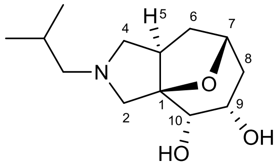
H
5
4
6
7
8
N
O
1
9
2
10
OH
HO

F41

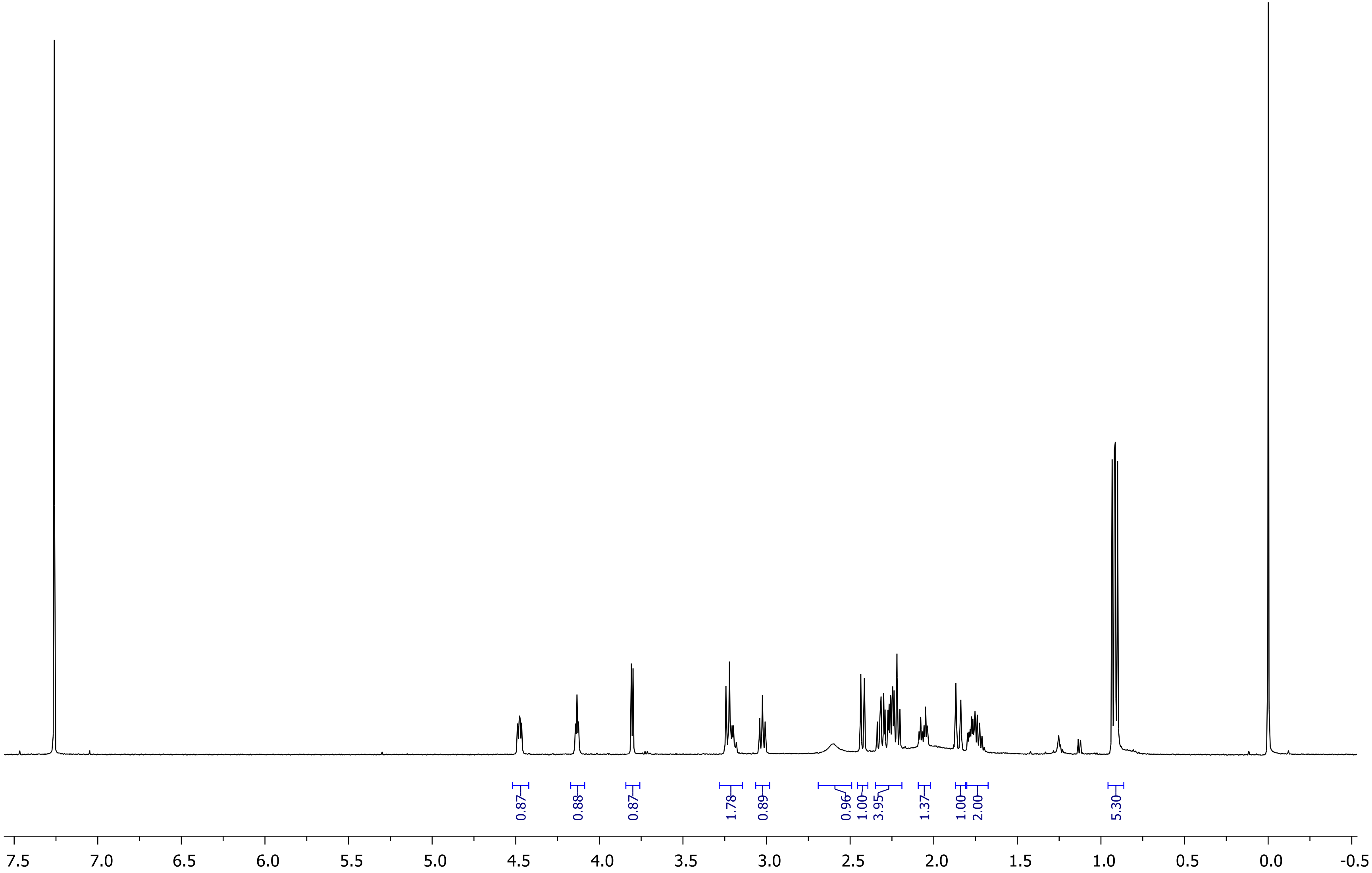
0.87
0.88
0.87
1.78
0.89
0.96
1.00
3.95
1.37
1.00
2.00
5.30
7.5
7.0
6.5
6.0
5.5
5.0
4.5
4.0
3.5
3.0
2.5
2.0
1.5
1.0
0.5
0.0
-0.5

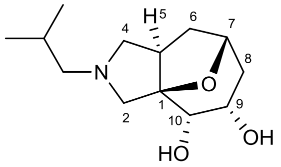
H
5
6
7
4
8
N
O
1
9
2
10
OH
HO

F41

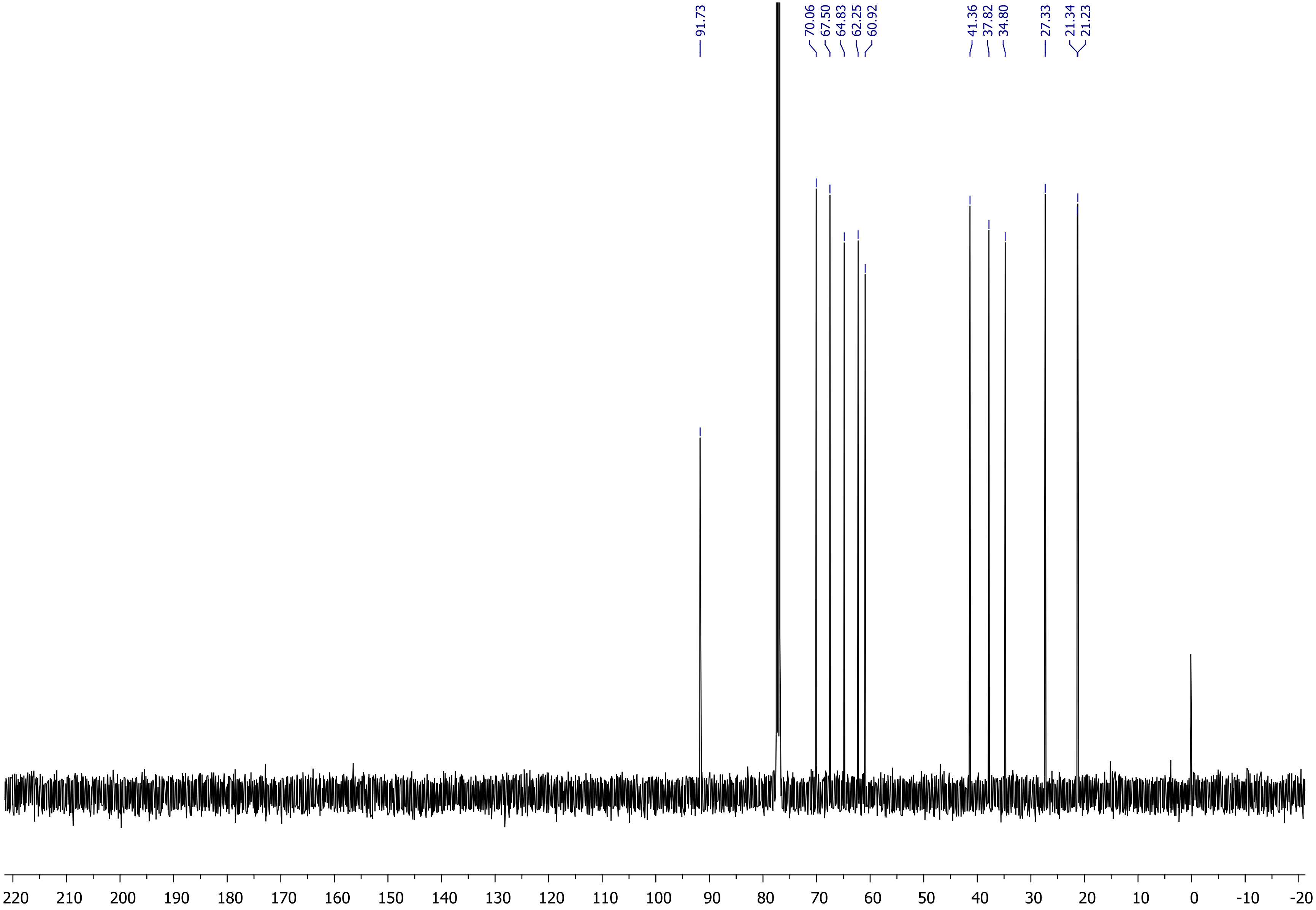
91.73
70.06
67.50
64.83
62.25
60.92
41.36
37.82
34.80
27.33
21.34
21.23
220 210 200 190 180 170 160 150 140 130 120 110 100 90 80 70 60 50 40 30 20 10 0 -10 -20

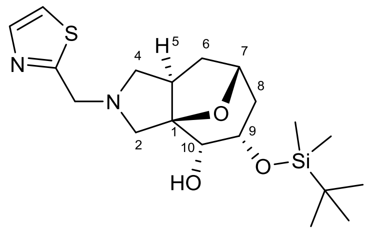

S54

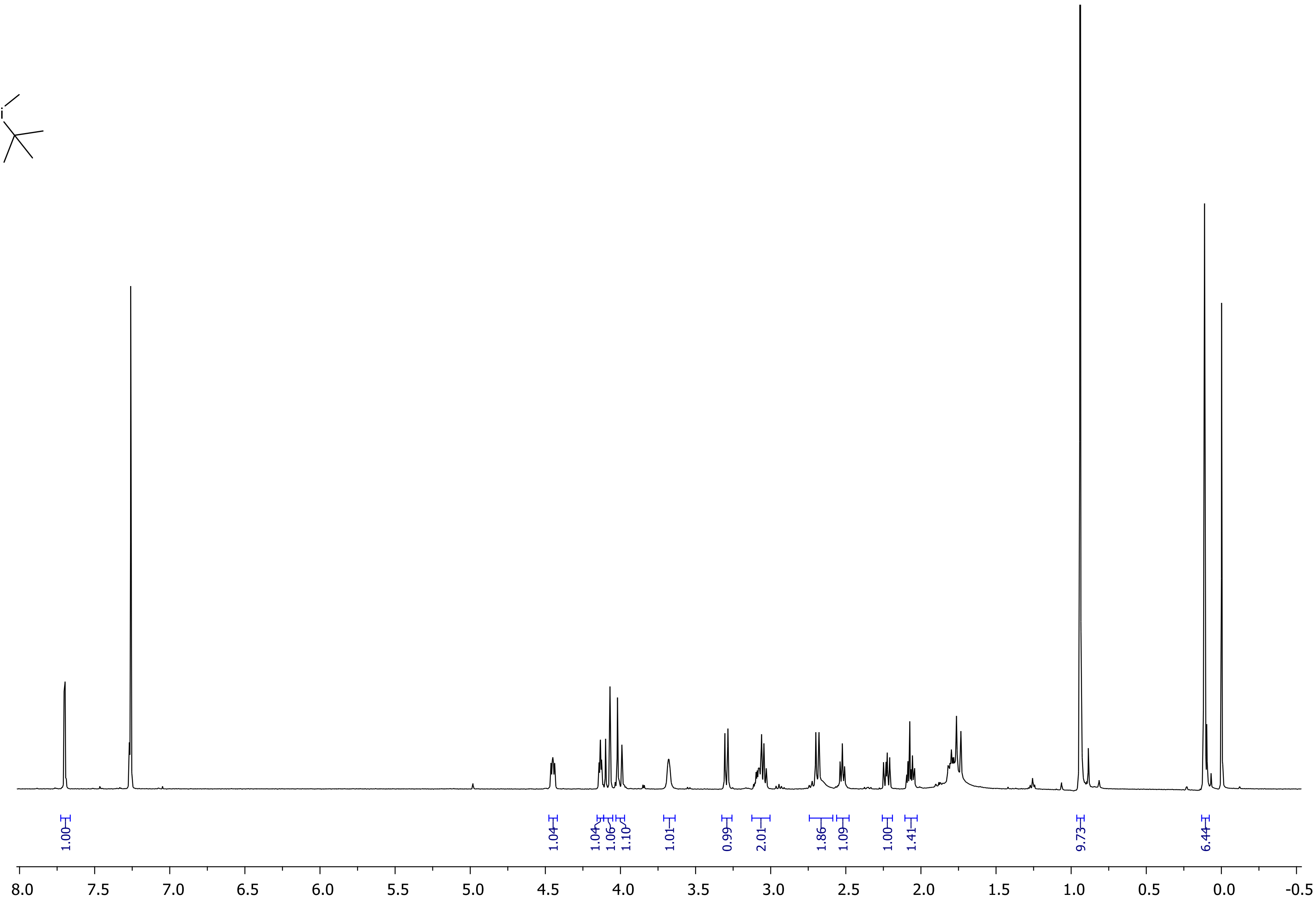

1.00
1.04
1.04
1.06
1.10
1.01
0.99
2.01
1.86
1.09
1.00
1.41
9.73
6.44
8.0
7.5
7.0
6.5
6.0
5.5
5.0
4.5
4.0
3.5
3.0
2.5
2.0
1.5
1.0
0.5
0.0
-0.5

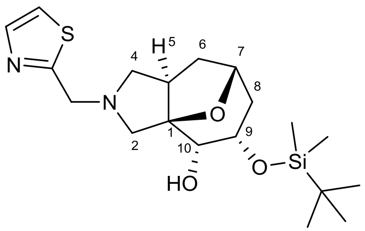
S
N
H
4
5
6
7
8
N
O
2
1
10
9
HO
O
Si

S54

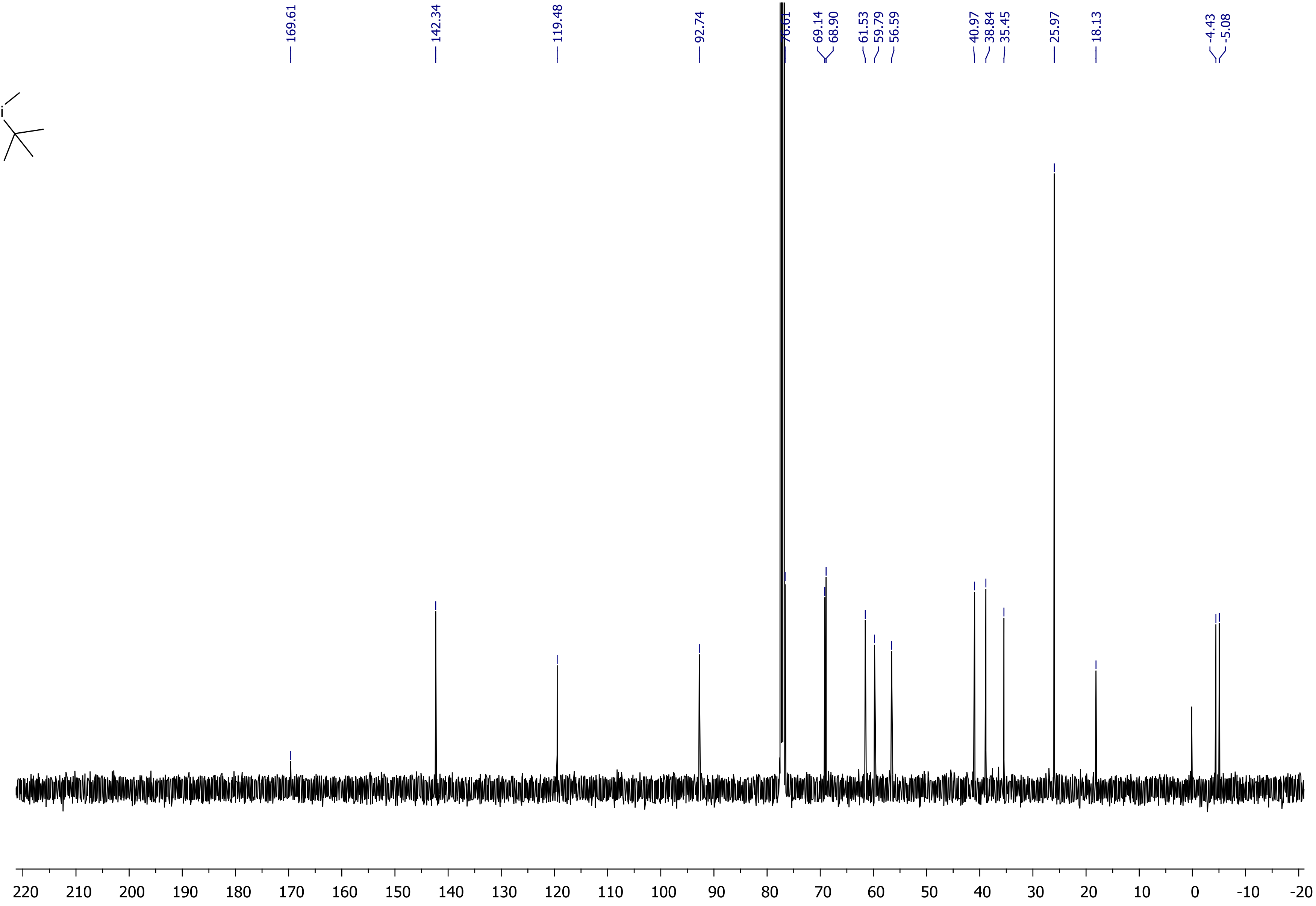
169.61
142.34
119.48
92.74
76.61
69.14
68.90
61.53
59.79
56.59
40.97
38.84
35.45
25.97
18.13
-4.43
-5.08
220
210
200
190
180
170
160
150
140
130
120
110
100
90
80
70
60
50
40
30
20
10
0
-10
-20

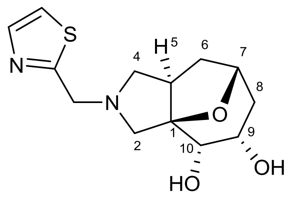
S
N
N
H
5
4
6
7
8
O
1
2
10
9
OH
HO

F42

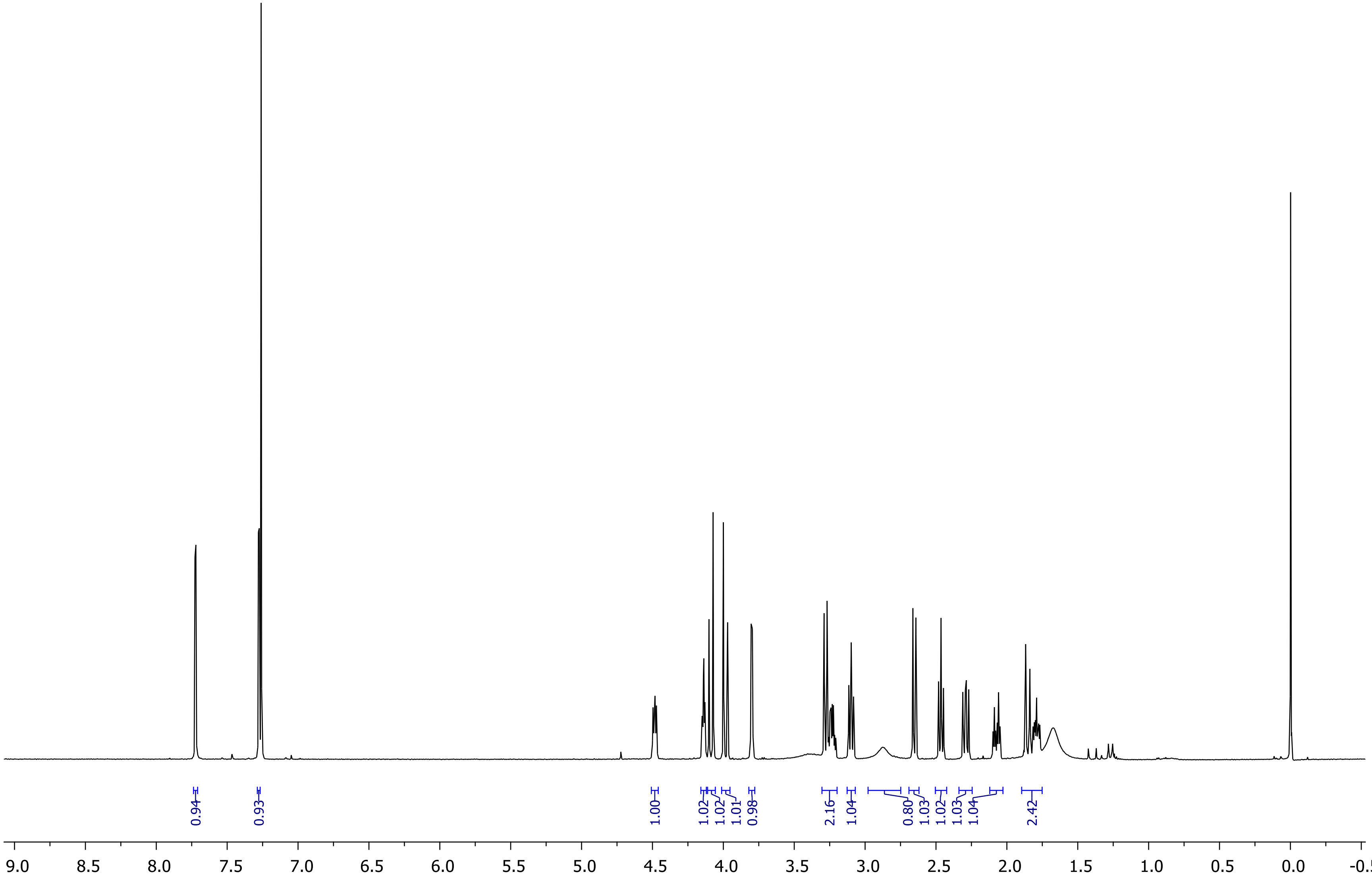
0.94
0.93
1.00
1.02
1.02
1.01
0.98
2.16
1.04
0.80
1.03
1.02
1.03
1.04
2.42
9.0
8.5
8.0
7.5
7.0
6.5
6.0
5.5
5.0
4.5
4.0
3.5
3.0
2.5
2.0
1.5
1.0
0.5
0.0
-0.5

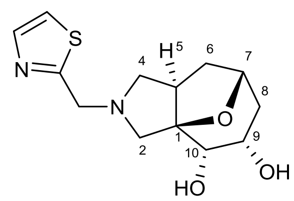

F42

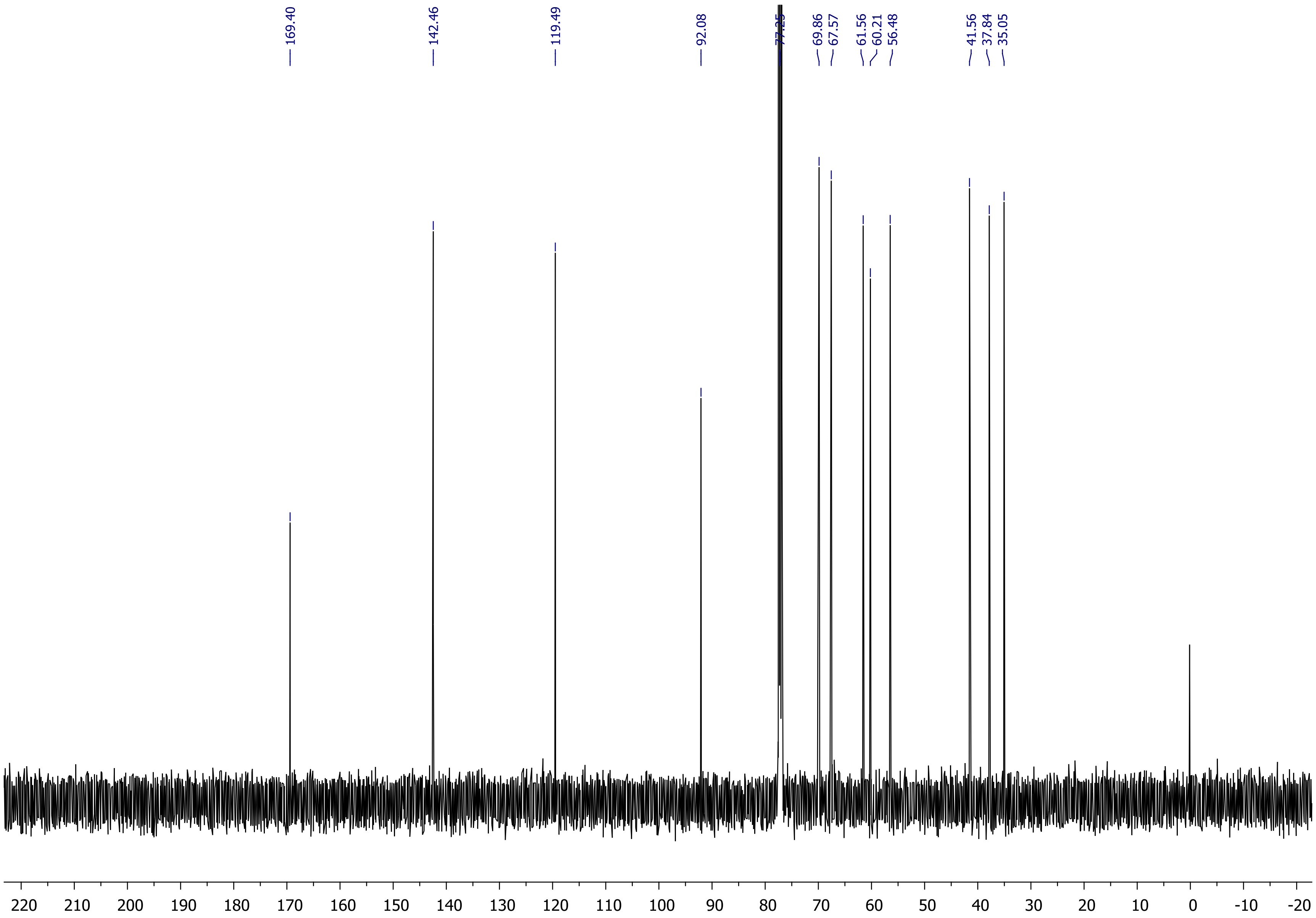
169.40
142.46
119.49
92.08
77.25
69.86
67.57
61.56
60.21
56.48
41.56
37.84
35.05
220 210 200 190 180 170 160 150 140 130 120 110 100 90 80 70 60 50 40 30 20 10 0 -10 -20

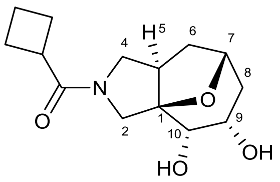
H
5
4
6
7
8
N
O
O
1
2
10
9
OH
HO

F43

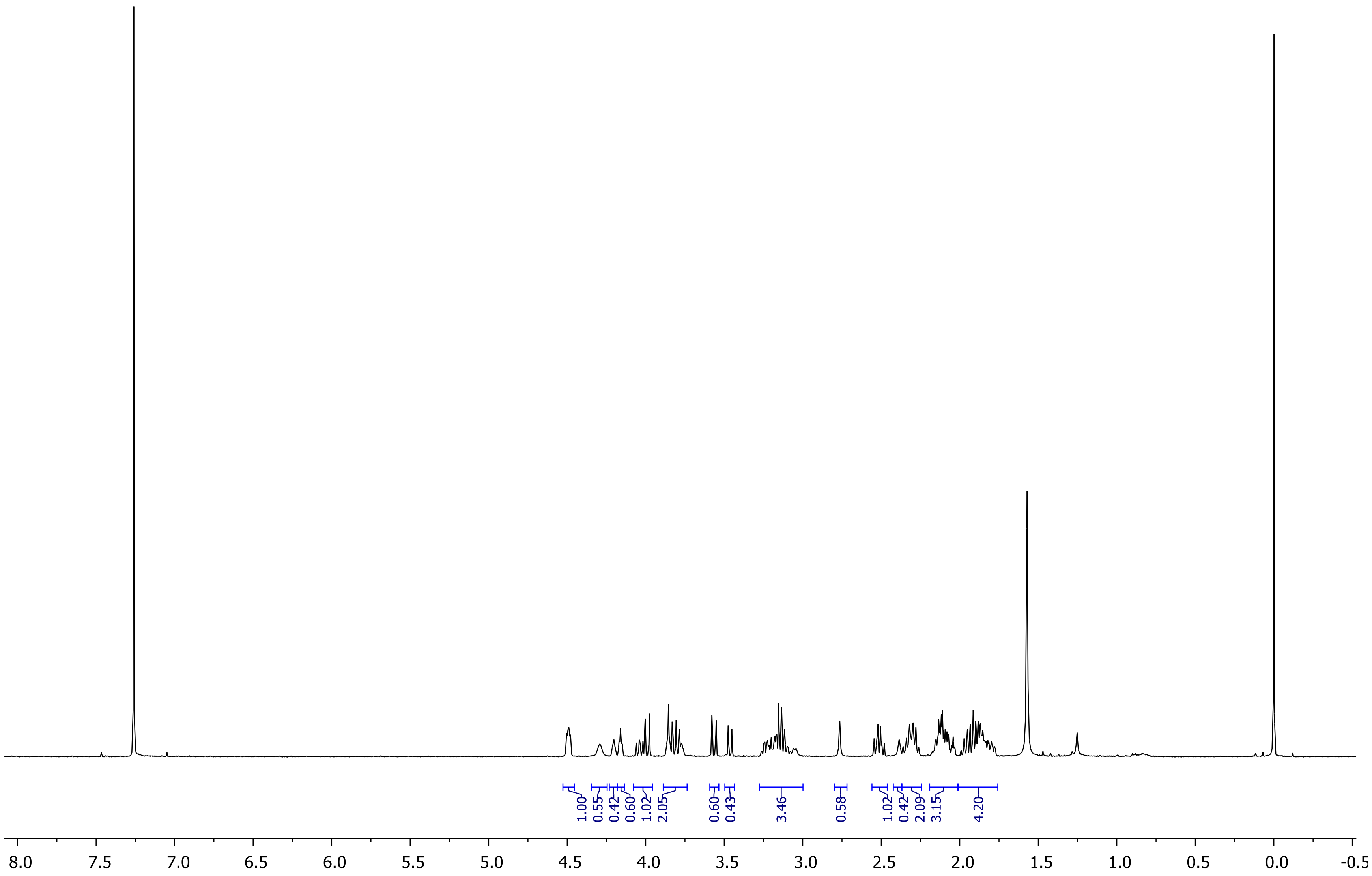
1.00
0.55
0.42
0.60
1.02
2.05
0.60
0.43
3.46
0.58
1.02
0.42
2.09
3.15
4.20
8.0
7.5
7.0
6.5
6.0
5.5
5.0
4.5
4.0
3.5
3.0
2.5
2.0
1.5
1.0
0.5
0.0
-0.5

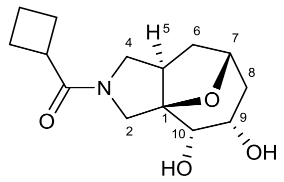

F43

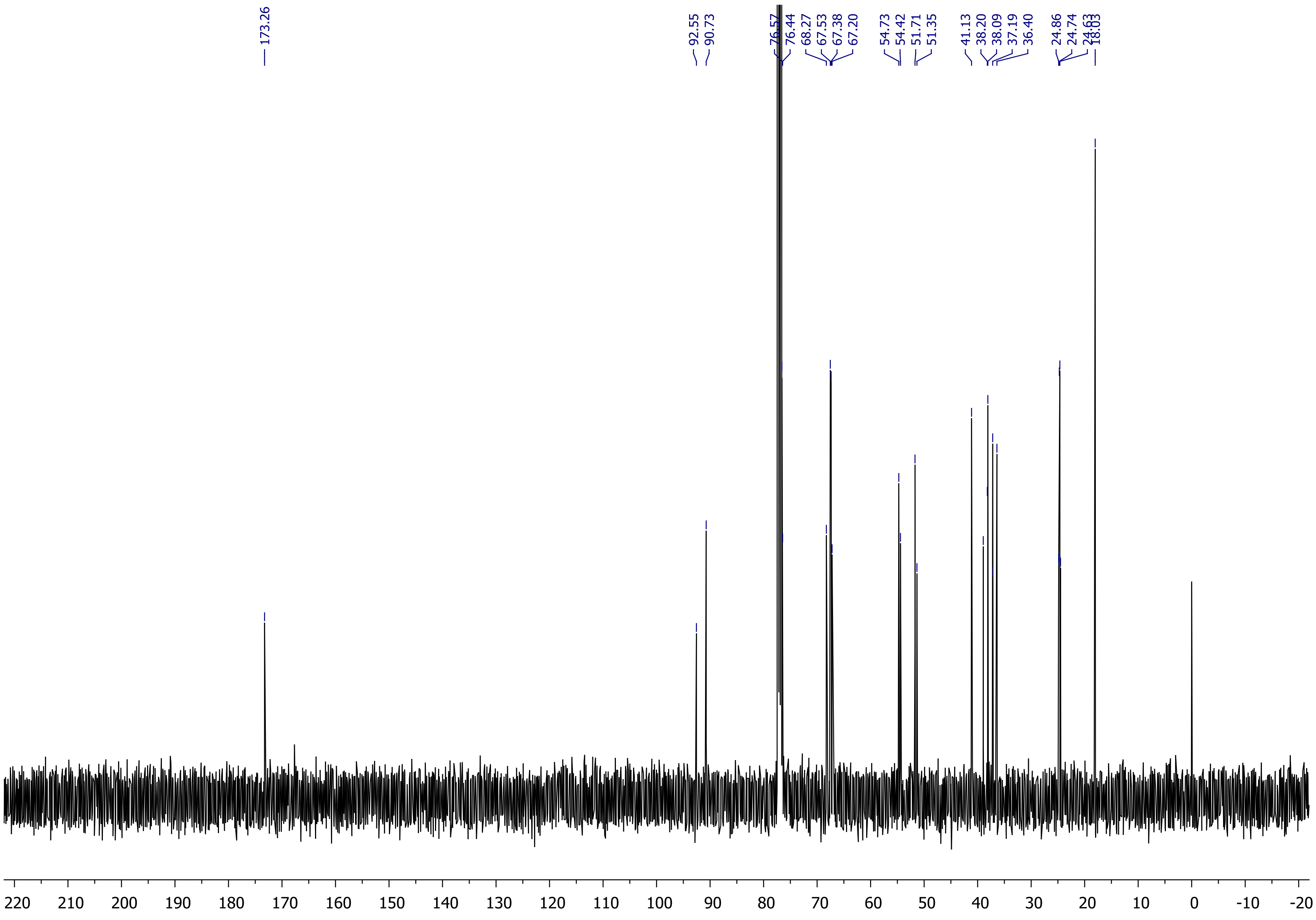
173.26
92.55
90.73
76.57
76.44
68.27
67.53
67.38
67.20
54.73
54.42
51.71
51.35
41.13
38.20
38.09
37.19
36.40
24.86
24.74
24.63
18.03
220 210 200 190 180 170 160 150 140 130 120 110 100 90 80 70 60 50 40 30 20 10 0 -10 -20

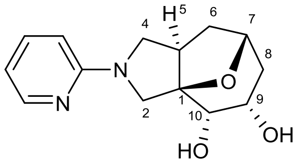
N
N
O
H
OH
HO
1
2
4
5
6
7
8
9
10

F44

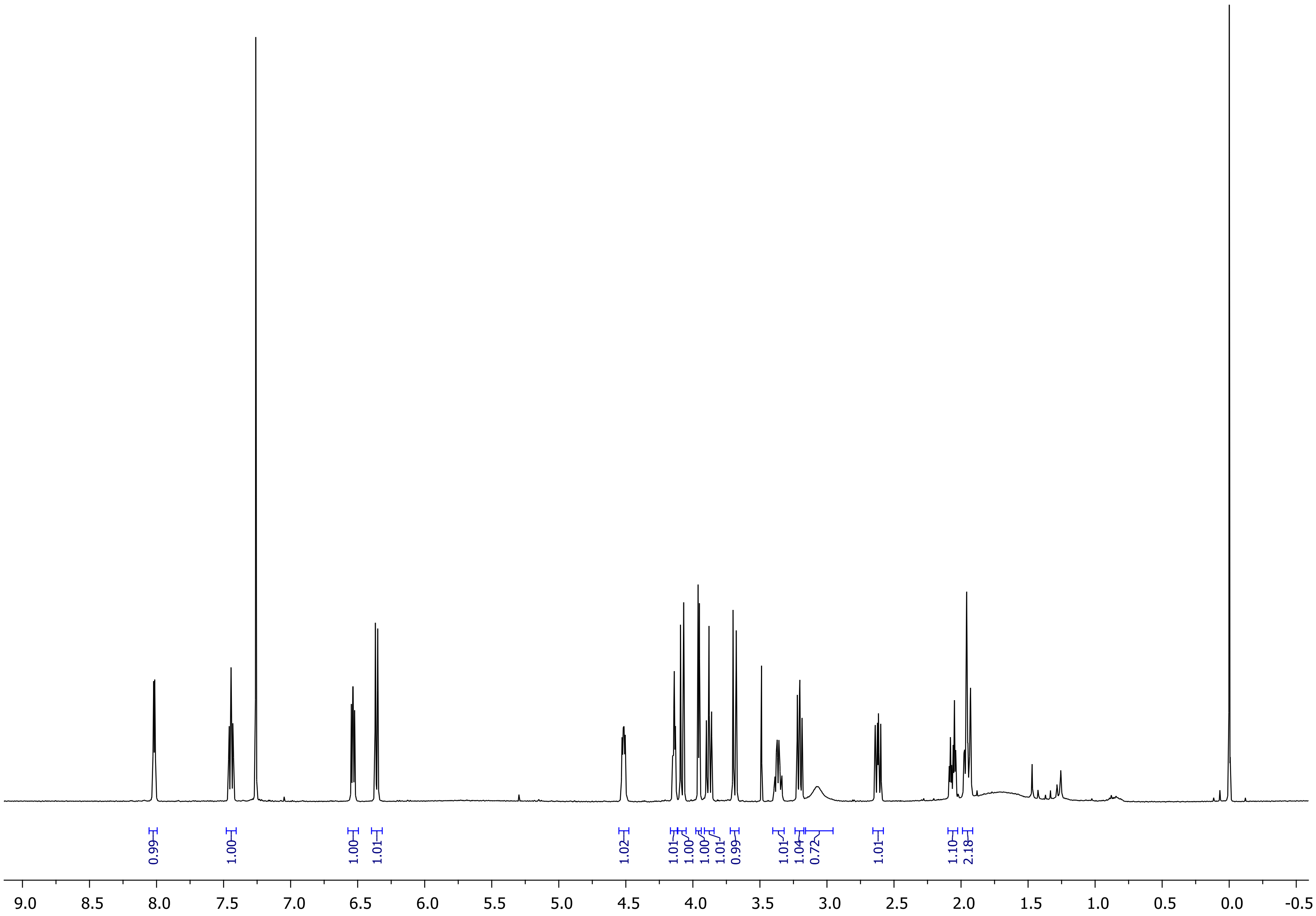
0.99
1.00
1.00
1.01
1.02
1.01
1.00
1.00
1.01
0.99
1.01
1.04
0.72
1.01
1.10
2.18
9.0 8.5 8.0 7.5 7.0 6.5 6.0 5.5 5.0 4.5 4.0 3.5 3.0 2.5 2.0 1.5 1.0 0.5 0.0 -0.5

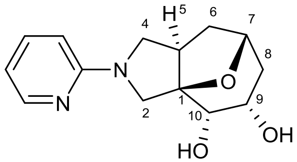

F44

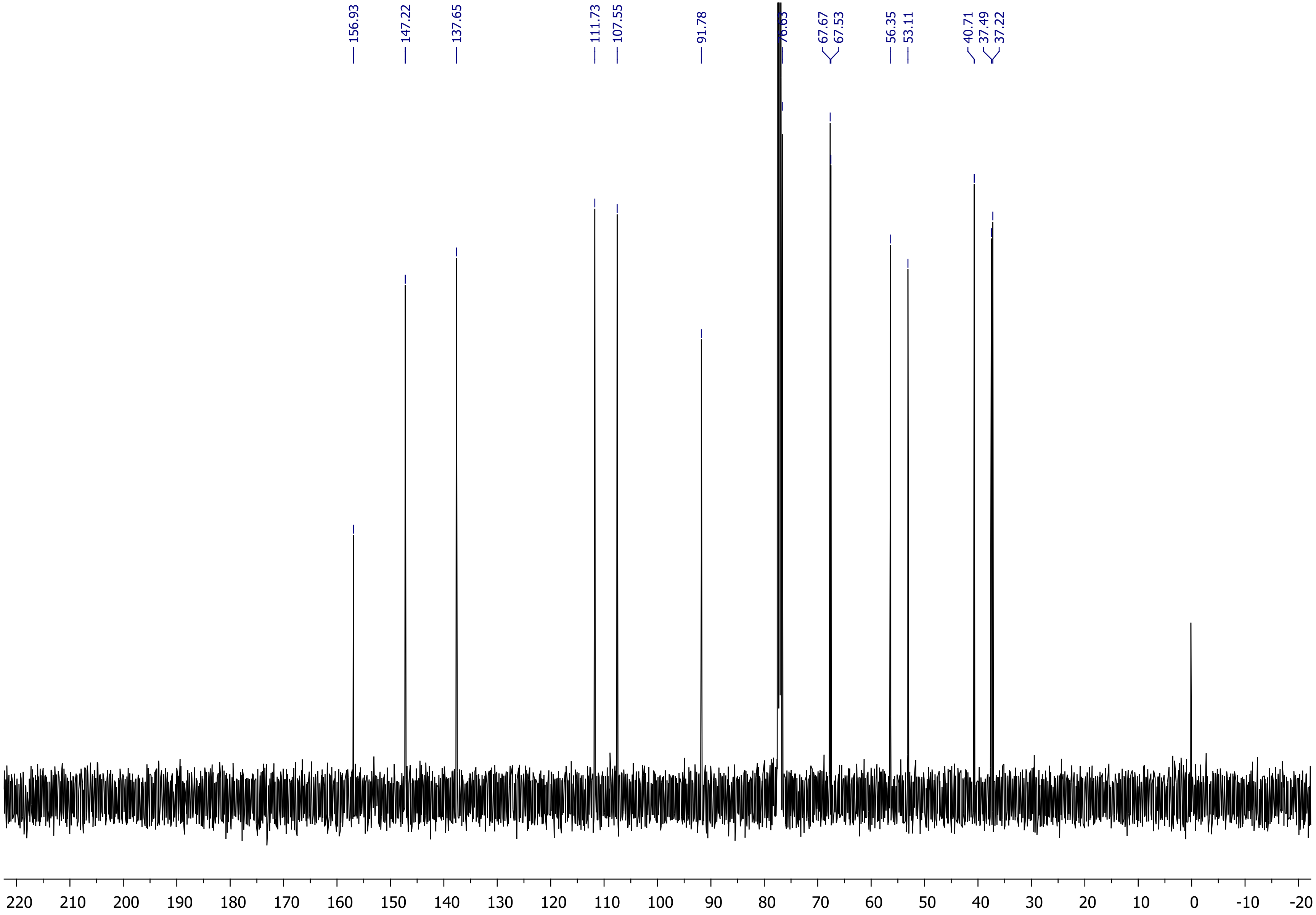
156.93
147.22
137.65
111.73
107.55
91.78
76.65
67.67
67.53
56.35
53.11
40.71
37.49
37.22
220 210 200 190 180 170 160 150 140 130 120 110 100 90 80 70 60 50 40 30 20 10 0 -10 -20

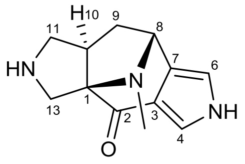

H10
9
8
11
7
6
HN
N
NH
13
1
2
3
4
O

F45

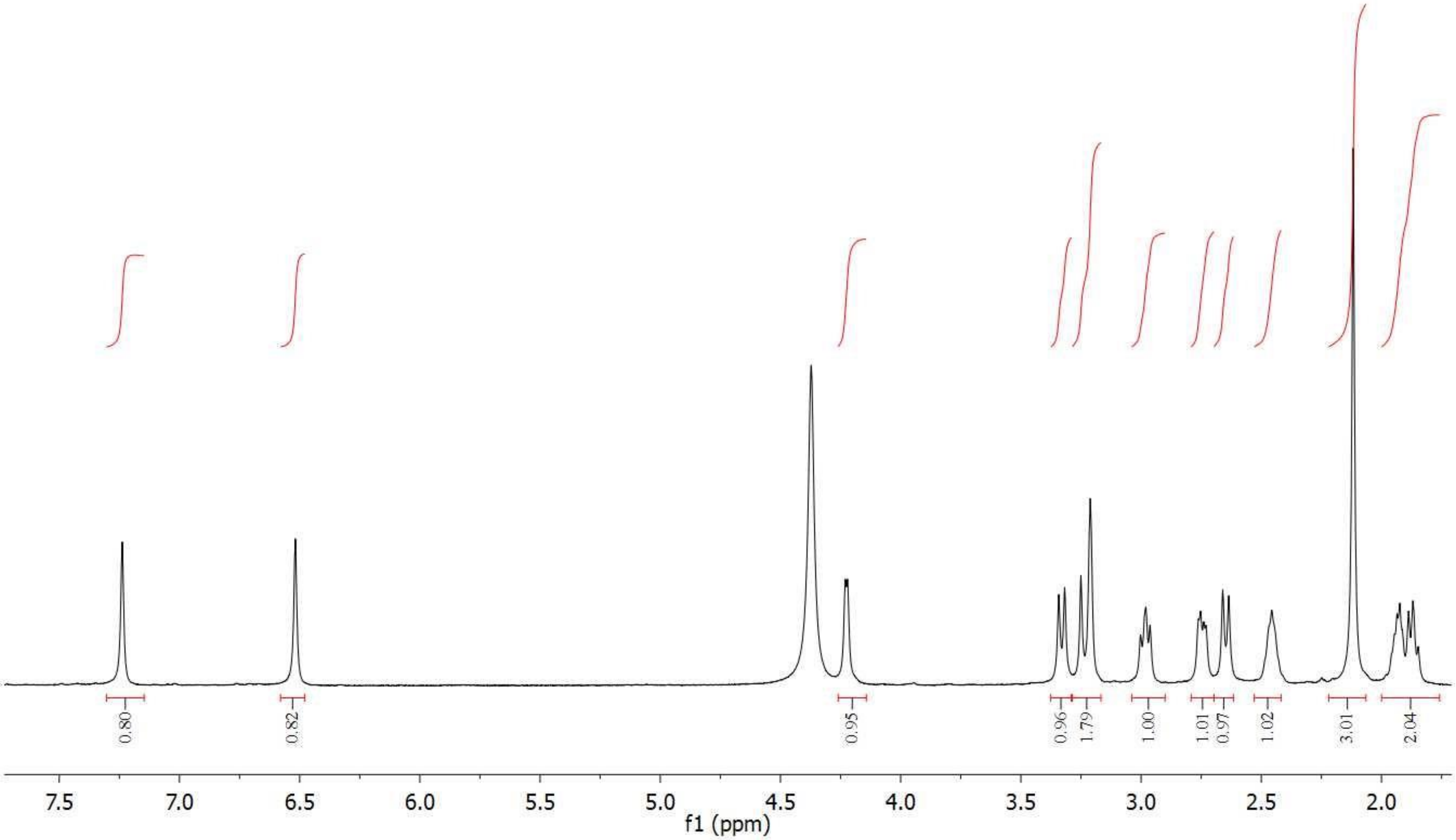

0.80
0.82
0.95
0.96
1.79
1.00
1.01
0.97
1.02
3.01
2.04
7.5
7.0
6.5
6.0
5.5
5.0
4.5
4.0
3.5
3.0
2.5
2.0
f1 (ppm)

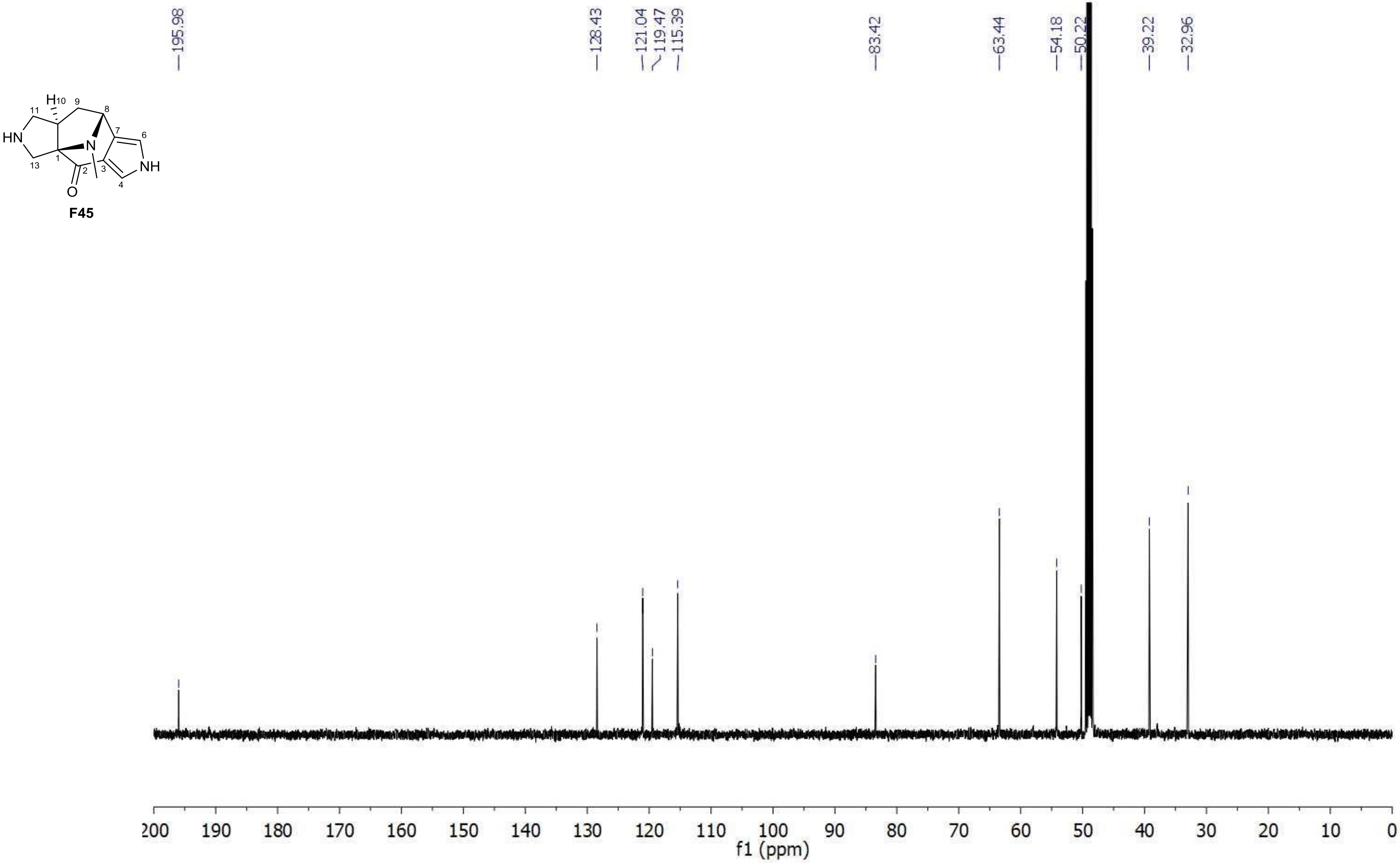

195.98
128.43
121.04
119.47
115.39
83.42
63.44
54.18
50.22
39.22
32.96
F45
200 190 180 170 160 150 140 130 120 110 100 90 80 70 60 50 40 30 20 10 0
f1 (ppm)

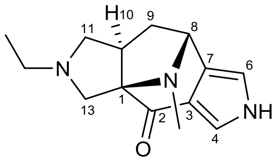

H10
N
NH
O

F46

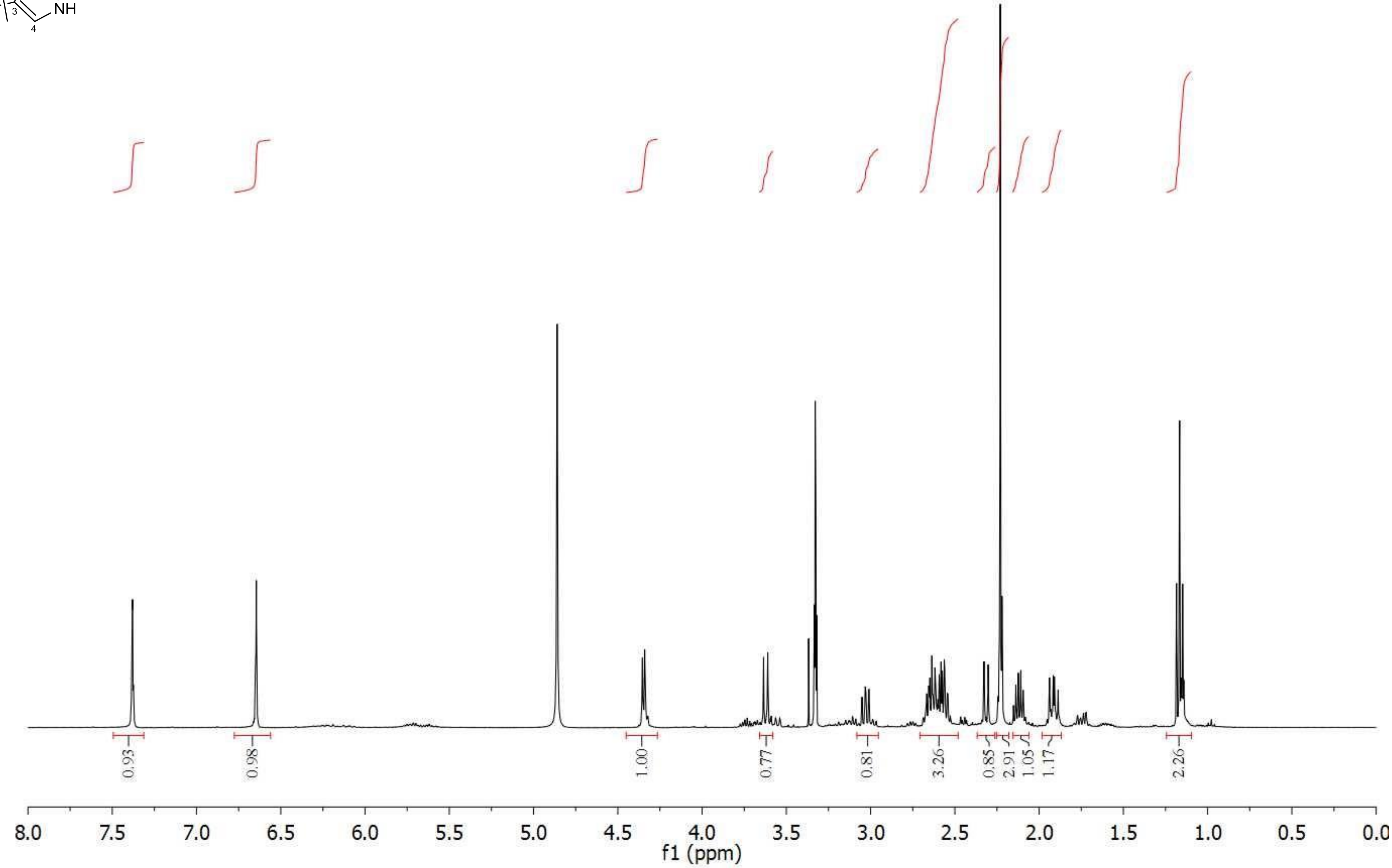

0.93
0.98
1.00
0.77
0.81
3.26
0.85
2.91
1.05
1.17
2.26
8.0 7.5 7.0 6.5 6.0 5.5 5.0 4.5 4.0 3.5 3.0 2.5 2.0 1.5 1.0 0.5 0.0
f1 (ppm)

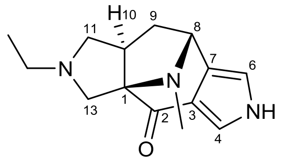
H10
9
8
11
7
6
N
N
NH
13
1
2
3
4
O

F46

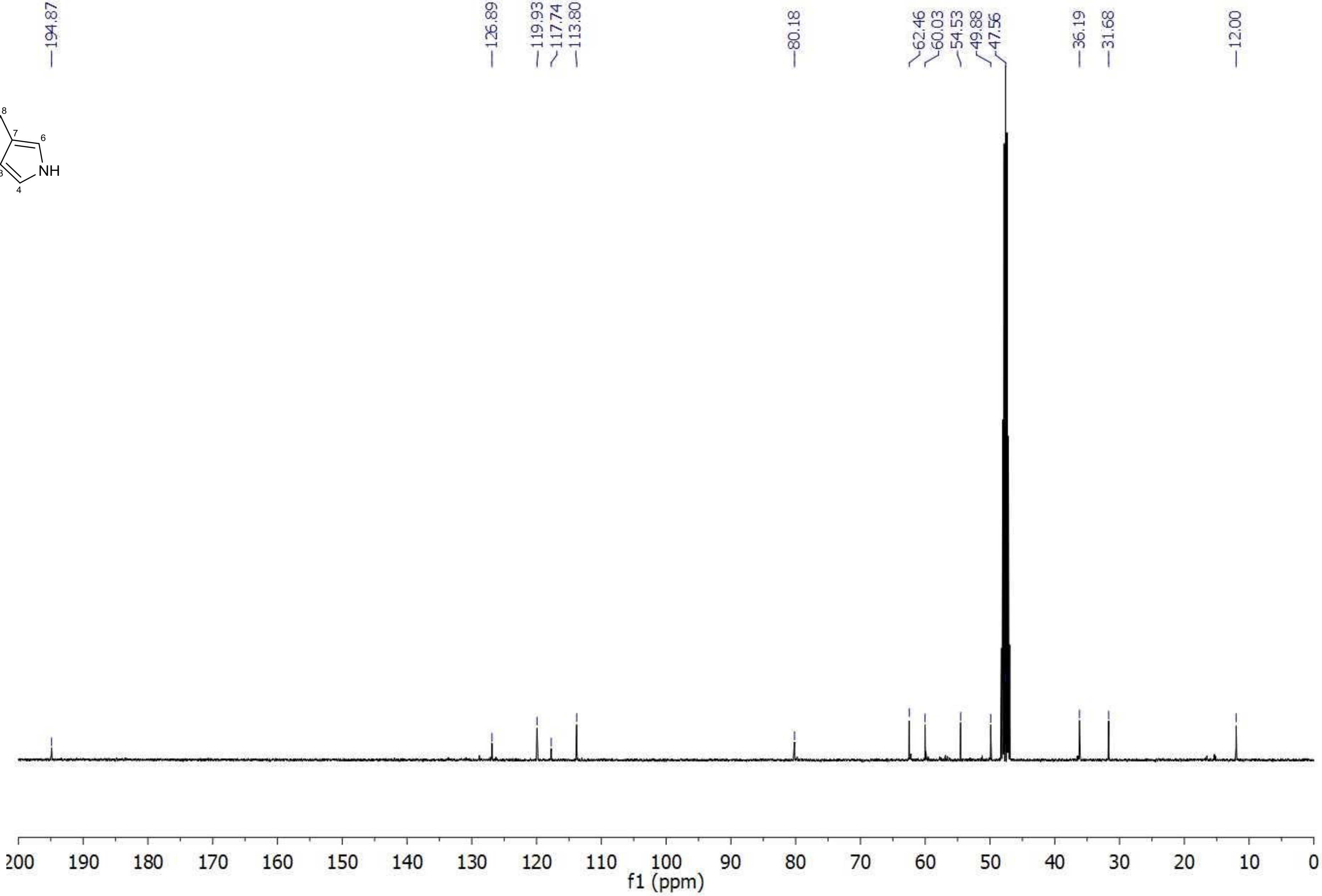
194.87
126.89
119.93
117.74
113.80
80.18
62.46
60.03
54.53
49.88
47.56
36.19
31.68
12.00
200
190
180
170
160
150
140
130
120
110
100
90
80
70
60
50
40
30
20
10
0
f1 (ppm)

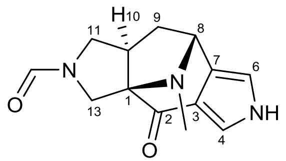
H10
9
8
11
7
6
N
N
NH
O
13
1
2
3
O
4

F47

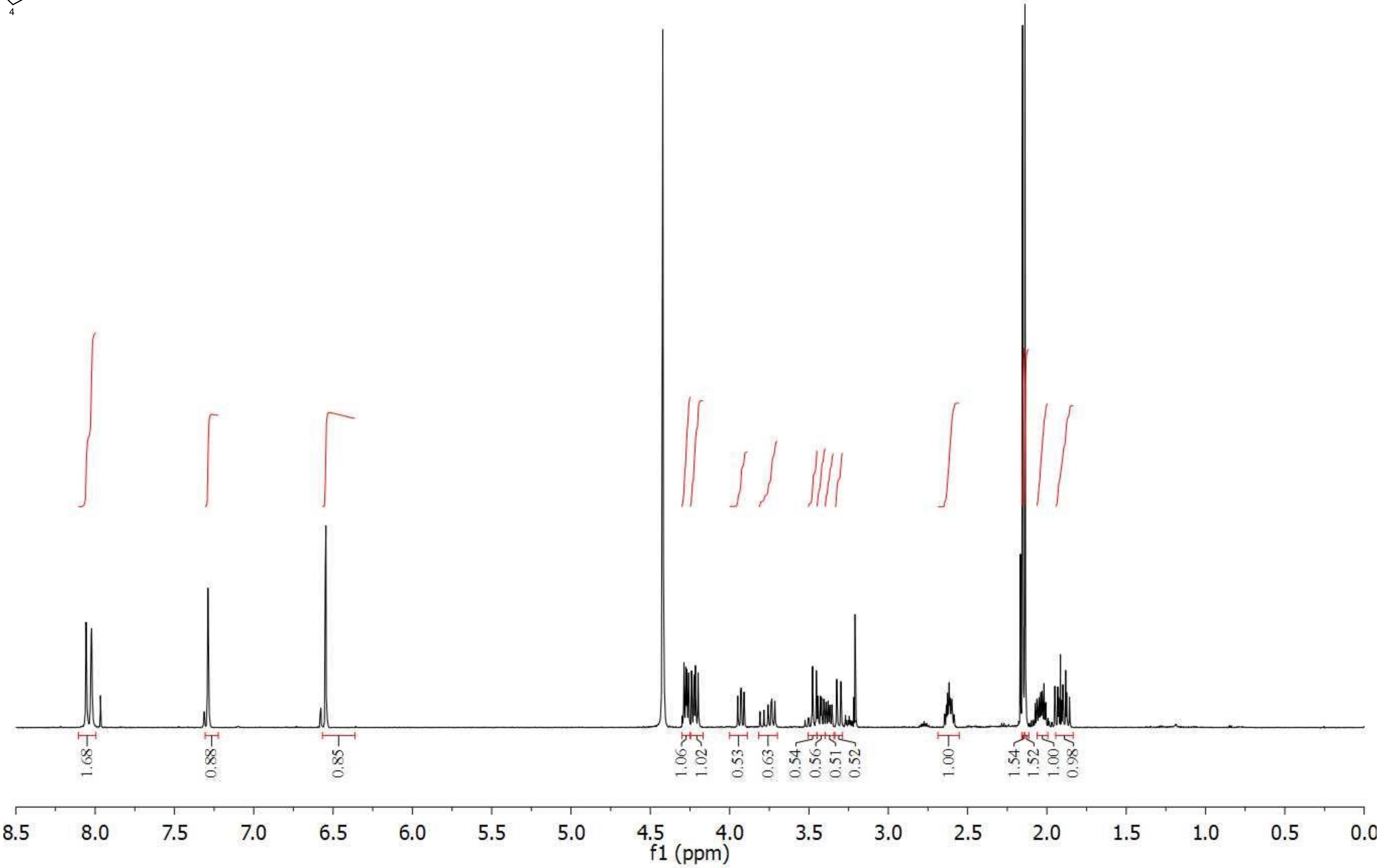
1.68
0.88
0.85
1.06
1.02
0.53
0.63
0.54
0.56
0.51
0.52
1.00
1.54
1.52
1.00
0.98
8.5
8.0
7.5
7.0
6.5
6.0
5.5
5.0
4.5
4.0
3.5
3.0
2.5
2.0
1.5
1.0
0.5
0.0
f1 (ppm)

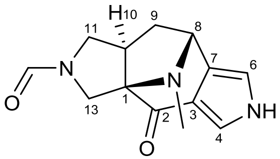
H10
9
8
11
7
6
N
N
1
13
2
3
NH
4
O
O

F47

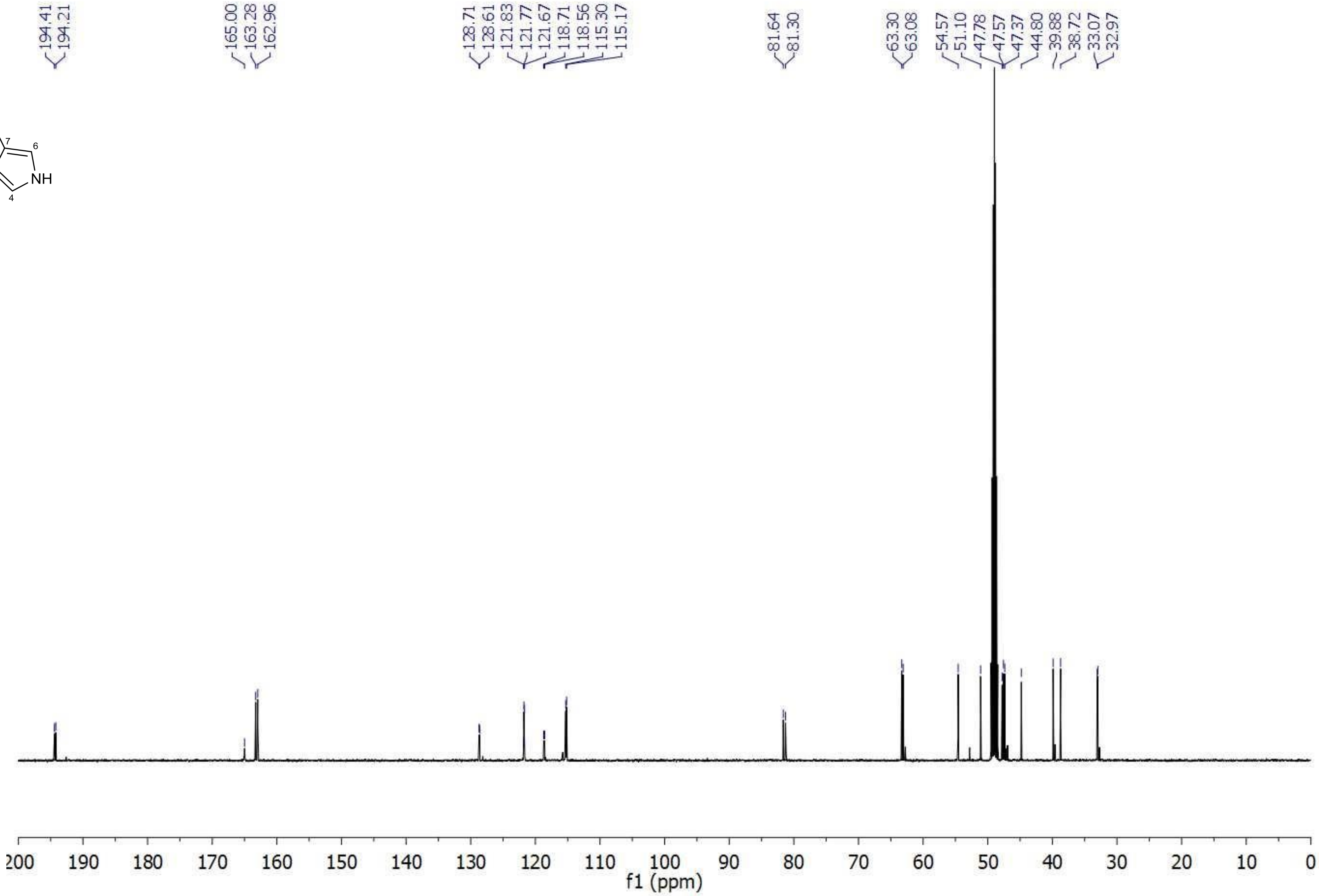
194.41
194.21
165.00
163.28
162.96
128.71
128.61
121.83
121.77
121.67
118.71
118.56
115.30
115.17
81.64
81.30
63.30
63.08
54.57
51.10
47.78
47.57
47.37
44.80
39.88
38.72
33.07
32.97
200 190 180 170 160 150 140 130 120 110 100 90 80 70 60 50 40 30 20 10 0
f1 (ppm)

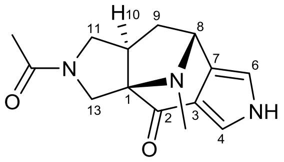

F48

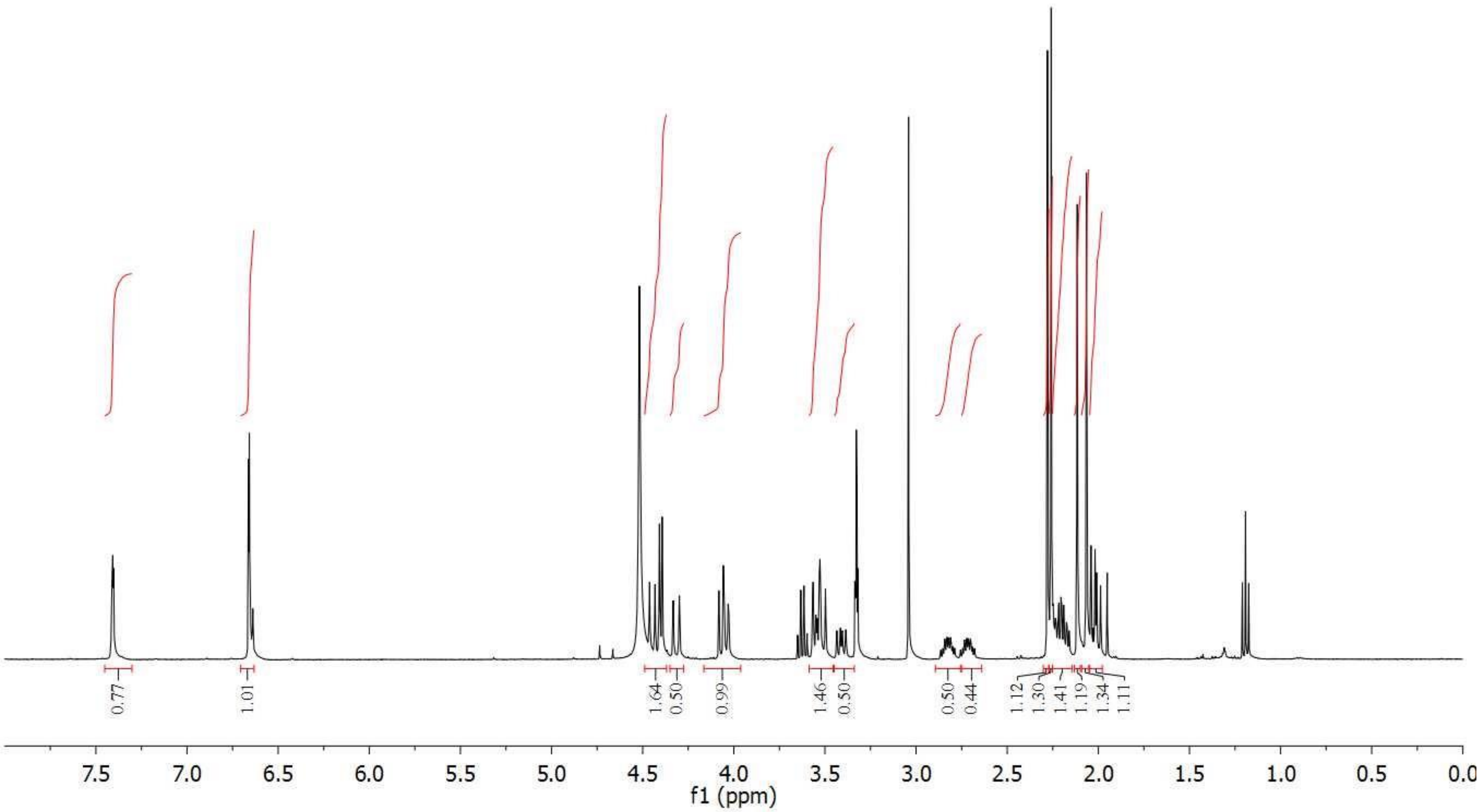

0.77
1.01
1.64
0.50
0.99
1.46
0.50
0.50
0.44
1.12
1.30
1.41
1.19
1.34
1.11
7.5 7.0 6.5 6.0 5.5 5.0 4.5 4.0 3.5 3.0 2.5 2.0 1.5 1.0 0.5 0.0
f1 (ppm)

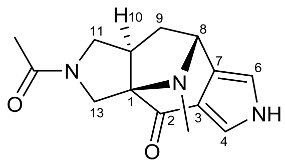
H
10
9
8
11
N
N
7
6
NH
13
1
2
3
4
O
O

F48

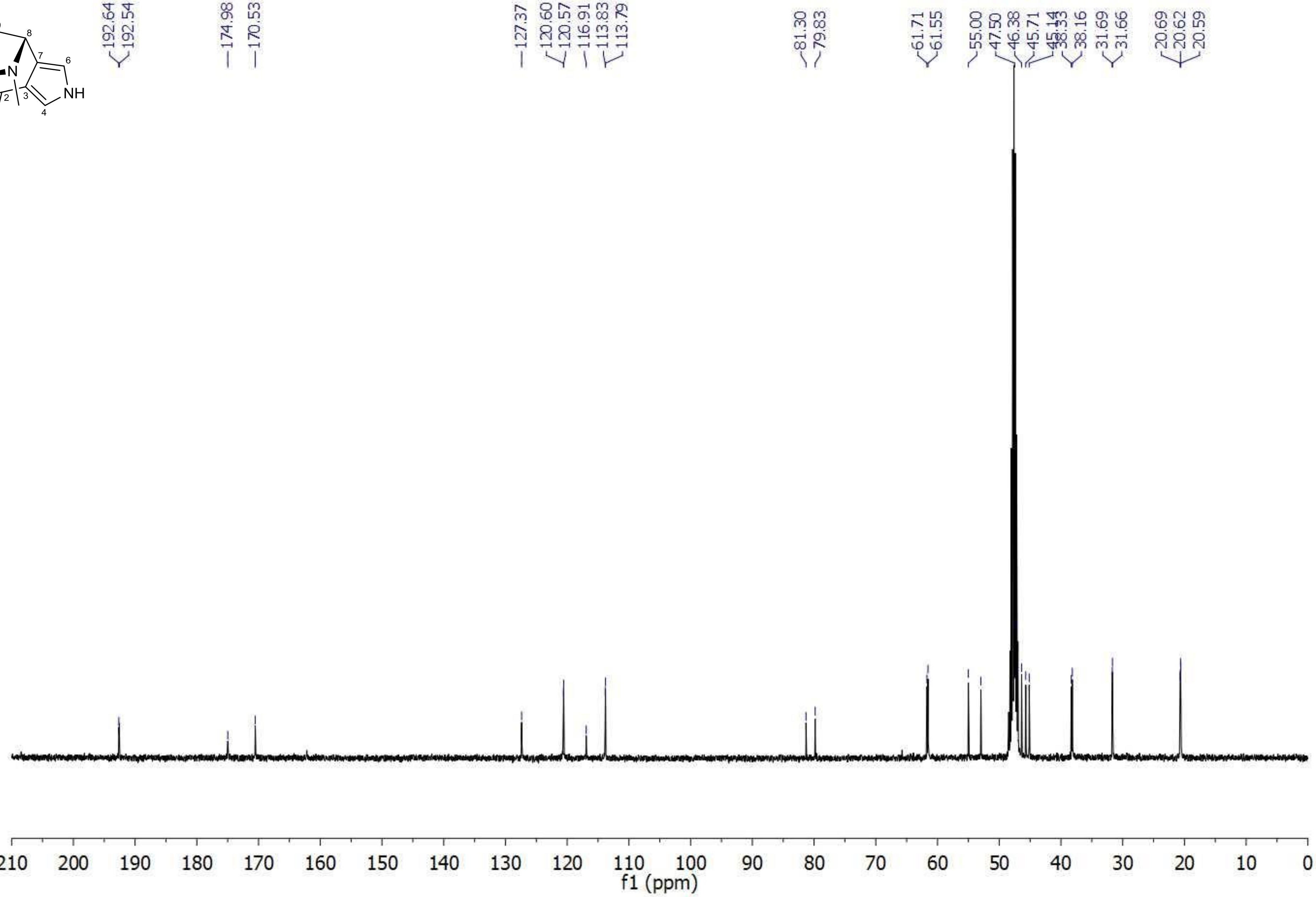
192.64
192.54
174.98
170.53
127.37
120.60
120.57
116.91
113.83
113.79
81.30
79.83
61.71
61.55
55.00
47.50
46.38
45.71
45.14
38.33
38.16
31.69
31.66
20.69
20.62
20.59
210 200 190 180 170 160 150 140 130 120 110 100 90 80 70 60 50 40 30 20 10 0
f1 (ppm)

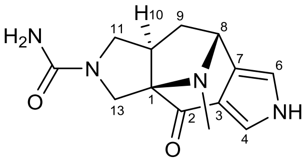

F49

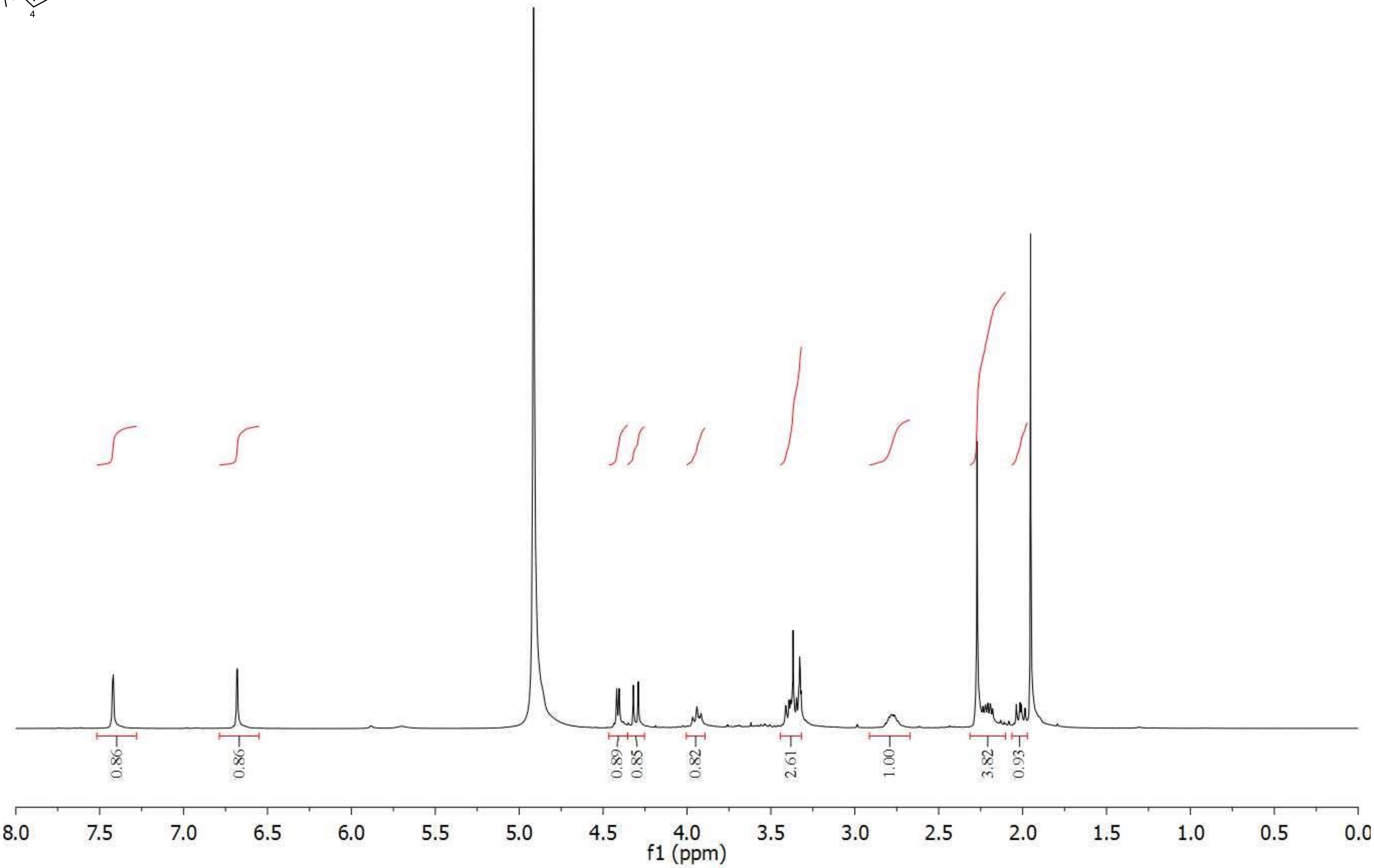

0.86
0.86
0.89
0.85
0.82
2.61
1.00
3.82
0.93
8.0
7.5
7.0
6.5
6.0
5.5
5.0
4.5
4.0
3.5
3.0
2.5
2.0
1.5
1.0
0.5
0.0
f1 (ppm)

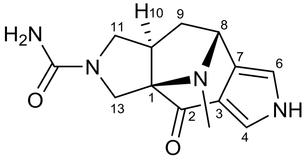

F49

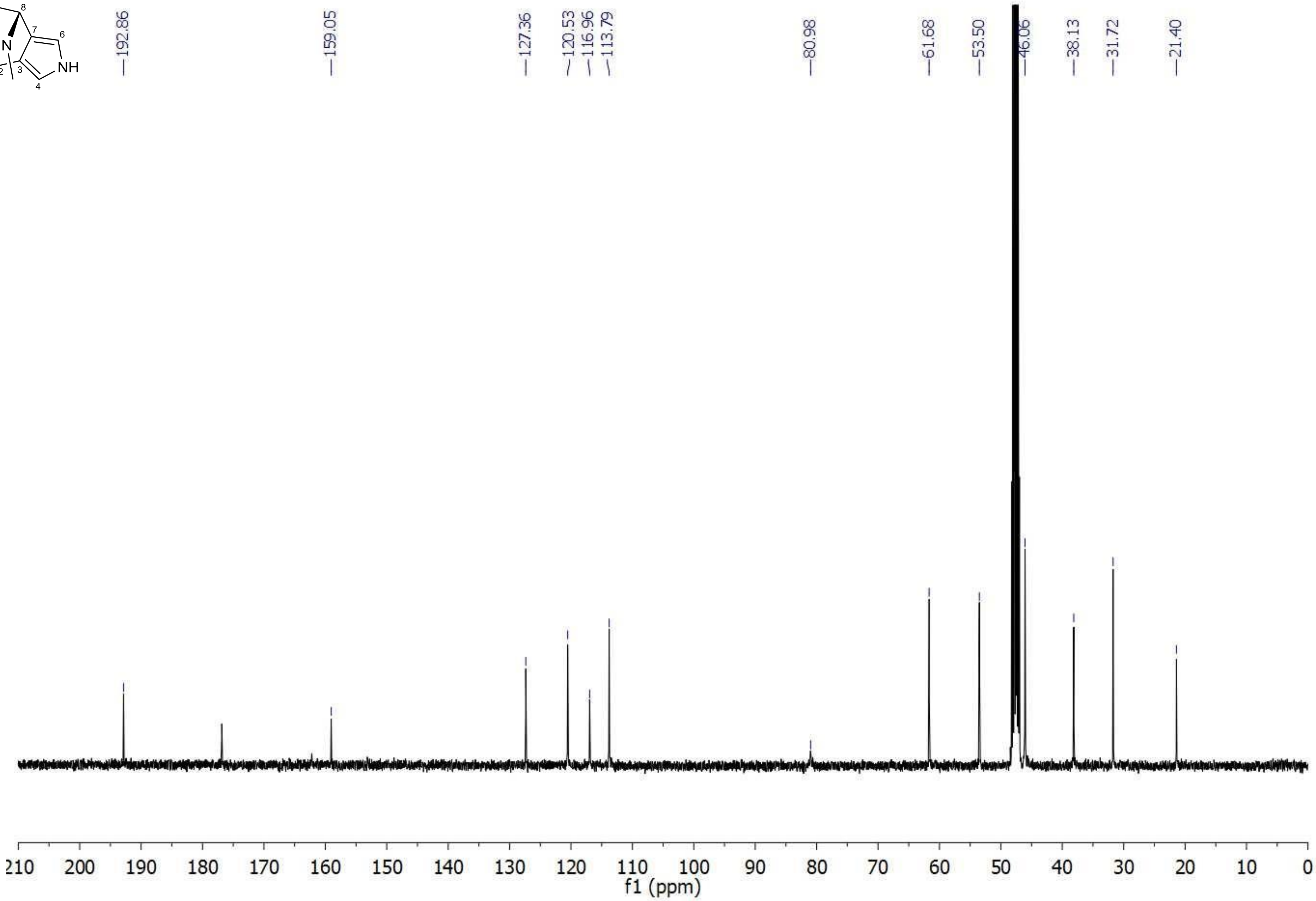

192.86
159.05
127.36
120.53
116.96
113.79
80.98
61.68
53.50
46.06
38.13
31.72
21.40
210 200 190 180 170 160 150 140 130 120 110 100 90 80 70 60 50 40 30 20 10 0
f1 (ppm)

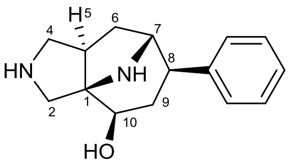

H
5
6
7
4
8
HN
NH
9
2
1
10
HO

F50

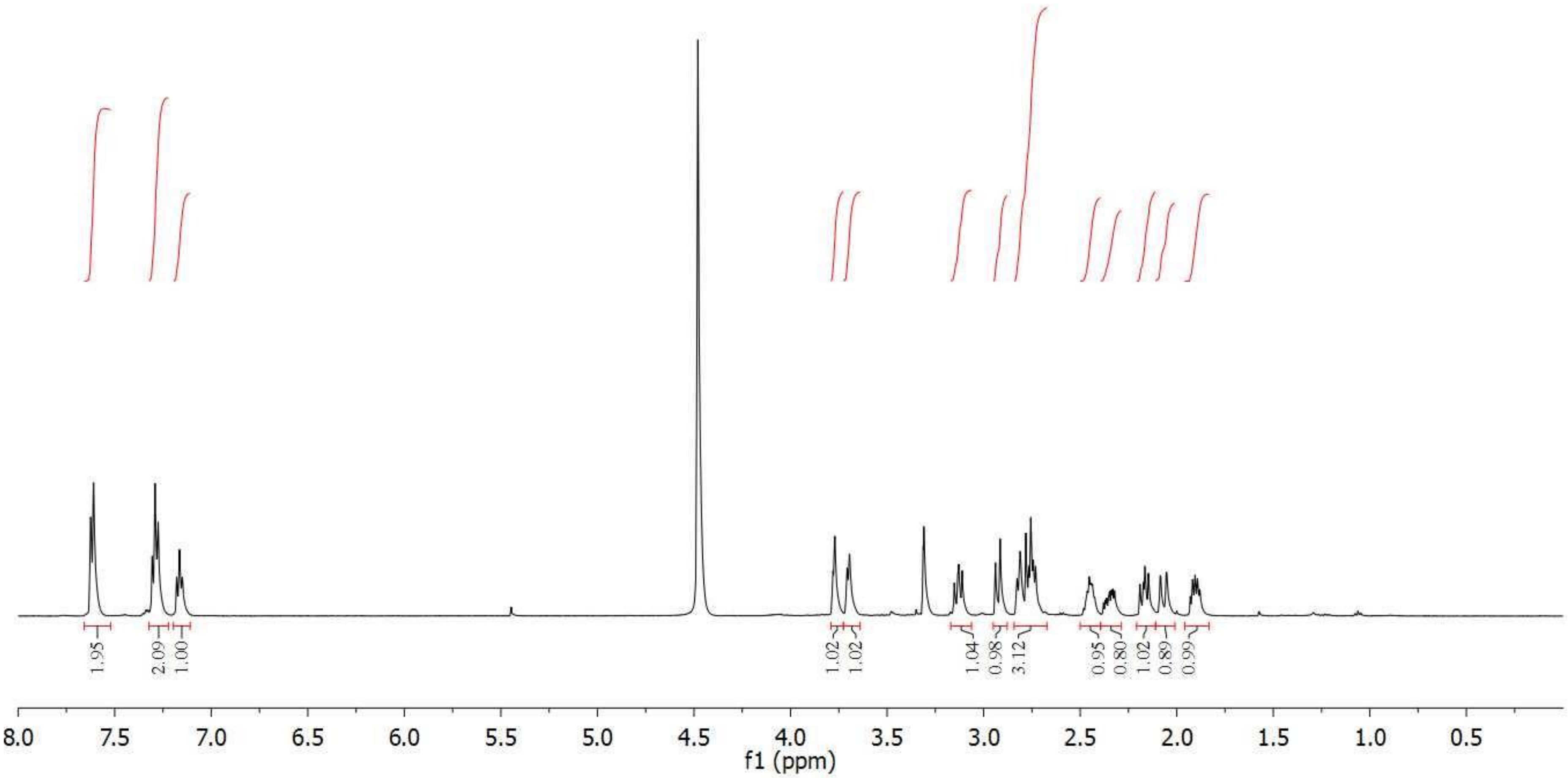

1.95
2.09
1.00
1.02
1.02
1.04
0.98
3.12
0.95
0.80
1.02
0.89
0.99
8.0
7.5
7.0
6.5
6.0
5.5
5.0
4.5
4.0
3.5
3.0
2.5
2.0
1.5
1.0
0.5
f1 (ppm)

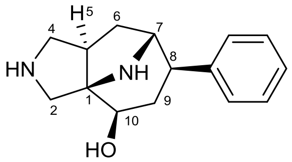
HN
NH
HO
H5
1
2
4
6
7
8
9
10

F50

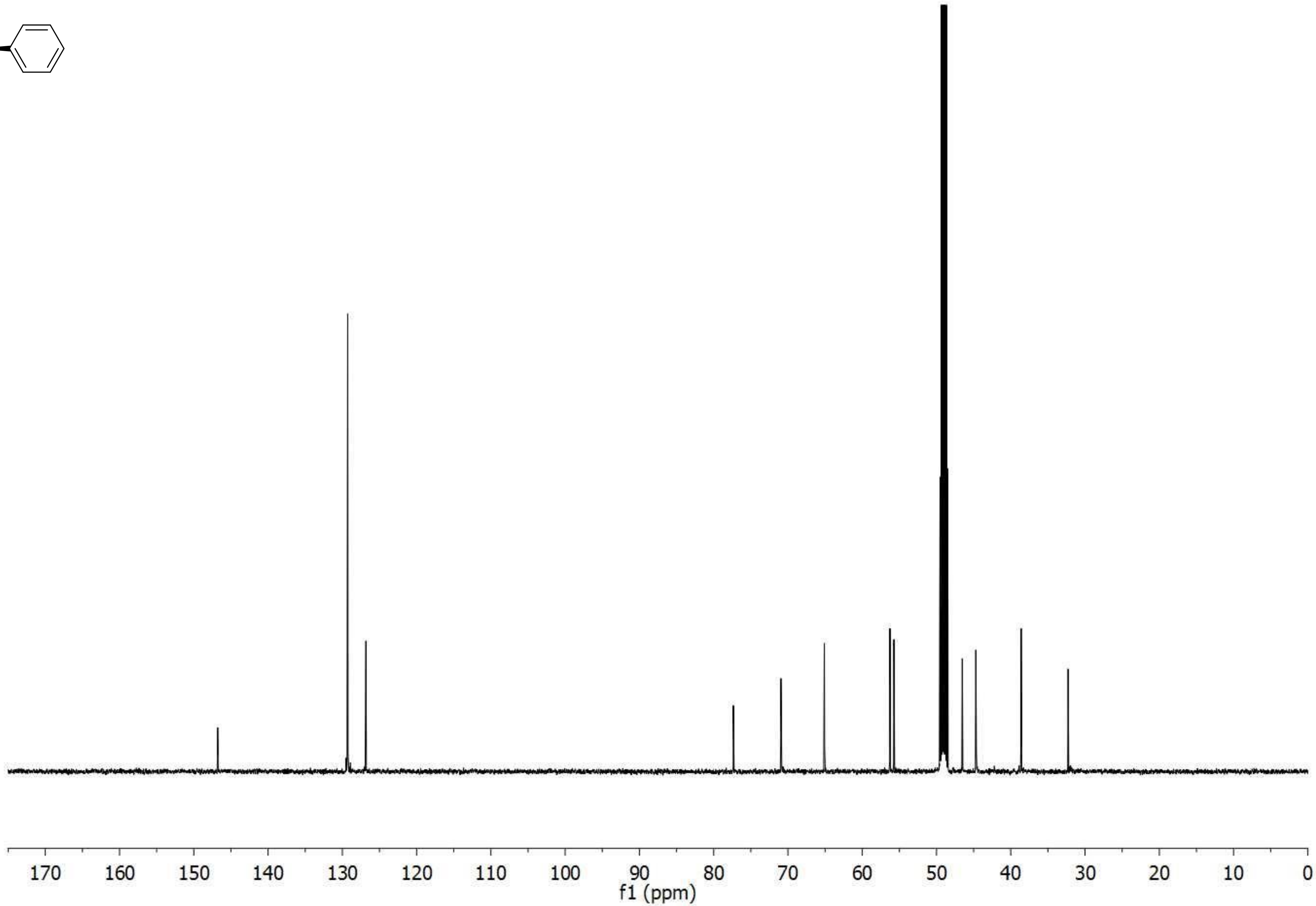
170 160 150 140 130 120 110 100 90 80 70 60 50 40 30 20 10 0
f1 (ppm)

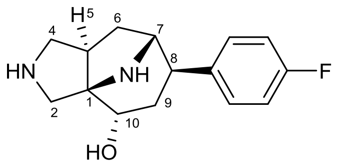
HN
NH
HO
F
H5

F51

+

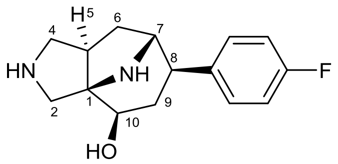
HN
NH
HO
F
H5

F52

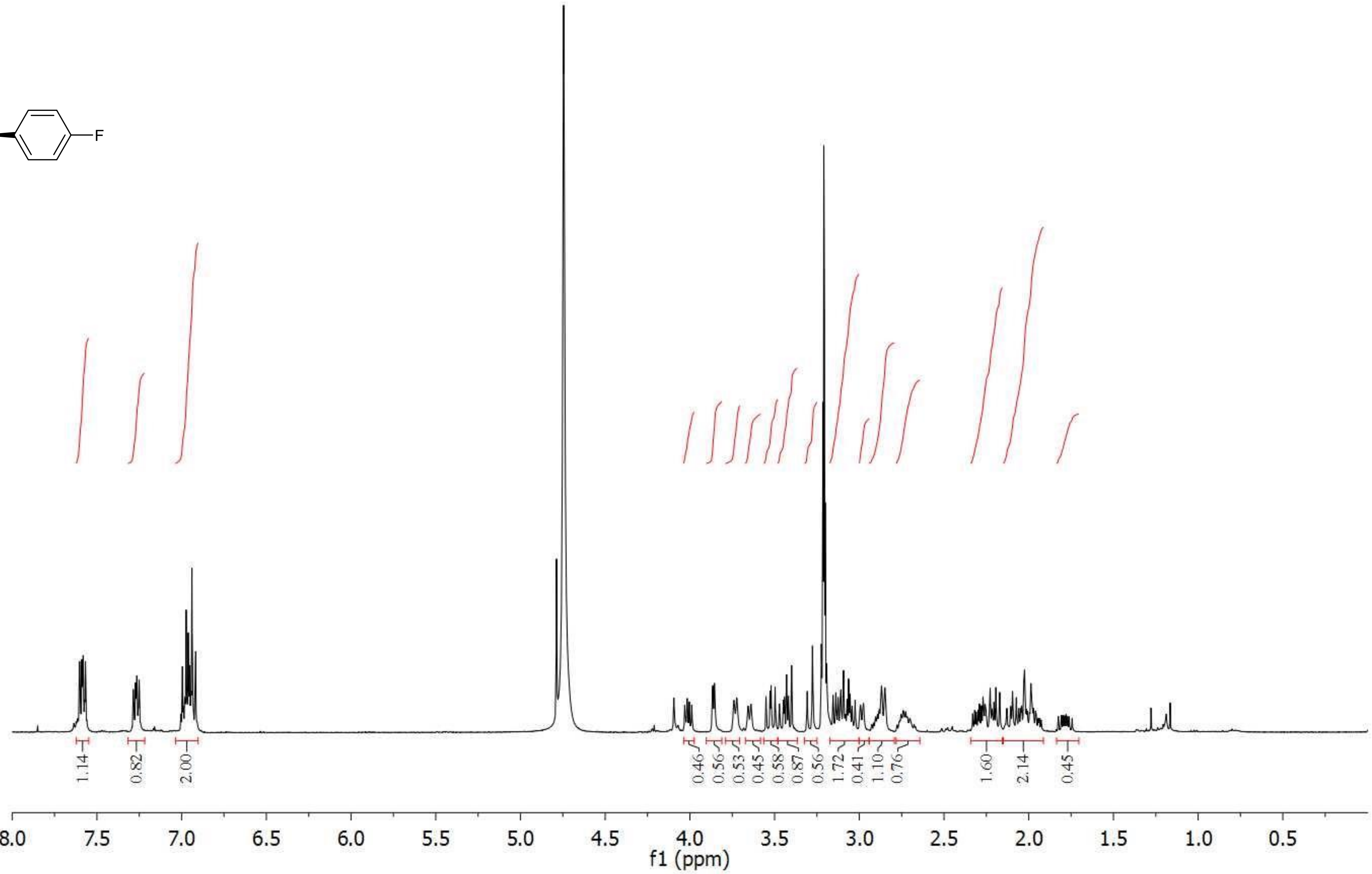
1.14
0.82
2.00
0.46
0.56
0.53
0.45
0.58
0.87
0.56
1.72
0.41
1.10
0.76
1.60
2.14
0.45
8.0
7.5
7.0
6.5
6.0
5.5
5.0
4.5
4.0
3.5
3.0
2.5
2.0
1.5
1.0
0.5
f1 (ppm)

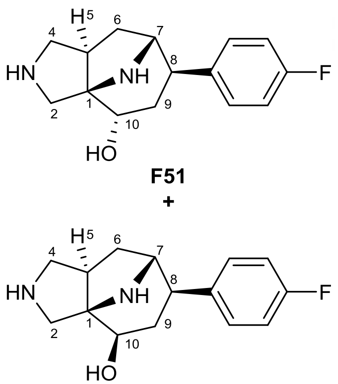

F51

+

F52

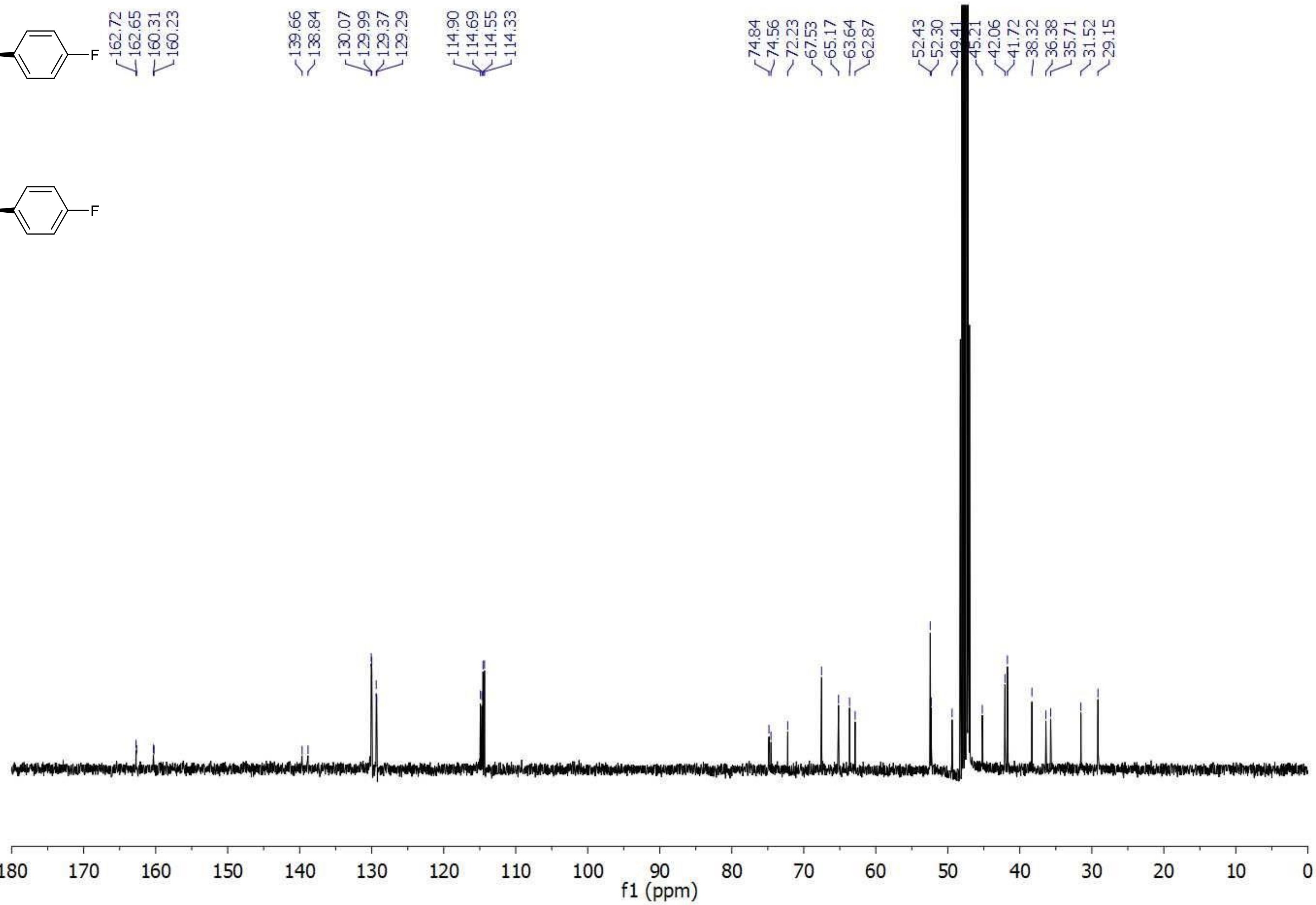

162.72
162.65
160.31
160.23
139.66
138.84
130.07
129.99
129.37
129.29
114.90
114.69
114.55
114.33
74.84
74.56
72.23
67.53
65.17
63.64
62.87
52.43
52.30
49.41
45.21
42.06
41.72
38.32
36.38
35.71
31.52
29.15
180
170
160
150
140
130
120
110
100
90
80
70
60
50
40
30
20
10
0
f1 (ppm)

8.0 **References** (including full references from the paper with >10 authors)

[S1] A. Chaikuad, A. M. Petros, O. Fedorov, J. Xu, S. Knapp, *Medhemcomm* **2014**, *5*, 1843–1848.

[S2] M. J. Harner, B. A. Chauder, J. Phan, S. W. Fesik, *J. Med. Chem.* **2014**, *57*, 9687–9692.

[S3] E. H. Demont, C. Chung, R. C. Furze, P. Grandi, A.-M. Michon, C. Wellaway, N. Barrett, A. M. Bridges, P. D. Craggs, H. Diallo, D. P. Dixon, C. Douault, A. J. Emmons, E. J. Jones, B. V. Karamshi, K. Locke, D. J. Mitchell, B. H. Mouzon, R. K. Prinjha, A. D. Roberts, R. J. Sheppard, R. J. Watson, P. Bamborough, *J. Med. Chem.* **2015**, *58*, 5649–5673.

[S4] G. Poncet-Montange, Y. Zhan, J. P. Bardenhagen, A. Petrocchi, E. Leo, X. Shi, G. R. Lee, P. G. Leonard, M. K. Geck Do, M. G. Cardozo, J. N. Andersen, W. S. Palmer, P. Jones, J. E. Ladbury, *Biochem. J.* **2015**, *466*.

[S5] P. Bamborough, C. Chung, E. H. Demont, R. C. Furze, A. J. Bannister, K. H. Che, H. Diallo, C. Douault, P. Grandi, T. Kouzarides, A.-M. Michon, D. J. Mitchell, R. K. Prinjha, C. Rau, S. Robson, R. J. Sheppard, R. Upton, R. J. Watson, *Angew. Chemie Int. Ed.* **2016**, *55*, 11382–11386.

[S6] P. Ertl, S. Roggo, A. Schuffenhauer, *J. Chem. Inf. Model.* **2008**, *48*, 68–74.

[S7] S. Miyazaki, S. Katoh, K. Adachi, H. Isoshima, S. Kobayashi, Y. Matsuzaki, W. Watanabe, K. Yamataka, S. Kiyonari, S. Wamaki, **2005**, US 2005/54645 A1.

[S8] A. H. Jadhav, H. Kim, *Tetrahedron Lett.* **2012**, *53*, 5338–5342.

[S9] C. S. Yeung, R. E. Ziegler, J. A. Porco, E. N. Jacobsen, *J. Am. Chem. Soc.* **2014**, *136*, 13614–13617.

[S10] G. Bianchi, C. De Micheli, R. Gandolfi, P. Grünanger, P. V. Finzi, O. V. de Pava, *J. Chem. Soc., Perkin Trans. 1* **1973**, 1148–1155.

[S11] V. Bavetsias, R. M. Lanigan, G. F. Ruda, B. Atrash, M. G. McLaughlin, A. Tumber, N. Y. Mok, Y.-V. Le Bihan, S. Dempster, K. J. Boxall, F. Jeganathan, S. B. Hatch, P. Savitsky, S. Velupillai, T. Krojer, K. S. England, J. Sejberg, C. Thai, A. Donovan, A. Pal, G. Scozzafava, J. M. Bennett, A.

Kawamura, C. Johansson, A. Szykowska, C. Gileadi, N. A. Burgess-Brown, F. von Delft, U. Oppermann, Z. Walters, J. Shipley, F. I. Raynaud, S. M. Westaway, R. K. Prinjha, O. Fedorov, R. Burke, C. J. Schofield, I. M. Westwood, C. Bountra, S. Müller, R. L. M. van Montfort, P. E. Brennan, J. Blagg, *J. Med. Chem.* **2016**, *59*, 1388–1409.

[S12] P. Filippakopoulos, S. Picaud, M. Mangos, T. Keates, J.-P. Lambert, D. Barsyte-Lovejoy, I. Felletar, R. Volkmer, S. Müller, T. Pawson, A.-C. Gingras, C. H. Arrowsmith, S. Knapp, *Cell* **2012**, *149*, 214–31.

[S13] P. M. Collins, J. T. Ng, R. Talon, K. Nekrosiute, T. Krojer, A. Douangamath, J. Brandao-Neto, N. Wright, N. M. Pearce, F. von Delft, *Acta Crystallogr. Sect. D Struct. Biol.* **2017**, *73*, 246–255.

[S14] M. Wojdyr, R. Keegan, G. Winter, A. Ashton, *Acta Crystallogr. Sect. A Found. Crystallogr.* **2013**, *69*, s299–s299.

[S15] T. Krojer, R. Talon, N. Pearce, P. Collins, A. Douangamath, J. Brandao-Neto, A. Dias, B. Marsden, F. von Delft, *Acta Crystallogr. Sect. D Struct. Biol.* **2017**, *73*, 267–278.

[S16] Smart, O.S. *et al.* Grade, version 1.102 Mar 29 2015. Cambridge, United Kingdom, Global Phasing Ltd (2011).

[S17] N. Pearce, A. R. Bradley, P. Collins, T. Krojer, R. Nowak, R. Talon, B. D. Marsden, S. Kelm, J. Shi, C. Deane, F. von Delft, *bioRxiv* **2016**, DOI: 10.1101/073411.

[S18] P. Emsley, B. Lohkamp, W. G. Scott, K. Cowtan, *Acta Crystallogr. Sect. D Biol. Crystallogr.* **2010**, *66*, 486–501.

[S19] G. N. Murshudov, P. Skubák, A. A. Lebedev, N. S. Pannu, R. A. Steiner, R. A. Nicholls, M. D. Winn, F. Long, A. A. Vagin, *Acta Crystallogr. Sect. D Biol. Crystallogr.* **2011**, *67*, 355–367.

[17b] Y. Wang, J.-Y. Wach, P. Sheehan, C. Zhong, C. Zhan, R. Harris, S. C. Almo, J. Bishop, S. J. Haggarty, A. Ramek, K. N. Berry, C. O'Herin, A. N. Koehler, A. W. Hung, D. W. Young, *ACS Med. Chem. Lett.* **2016**, *7*, 852–856.

[21b] S. Renner, W. A. L. van Otterlo, M. Dominguez Seoane, S. Möcklinghoff, B. Hofmann, S. Wetzel, A. Schuffenhauer, P. Ertl, T. I. Oprea, D. Steinhilber, L. Brunsveld, D. Rauh, H. Waldmann, *Nat. Chem. Biol.* **2009**, *5*, 585–592.

[25] P. Bamborough, C. Chung, E. H. Demont, R. C. Furze, A. J. Bannister, K. H. Che, H. Diallo, C. Douault, P. Grandi, T. Kouzarides, A.-M. Michon, D. J. Mitchell, R. K. Prinjha, C. Rau, S. Robson, R. J. Sheppard, R. Upton, R. J. Watson, *Angew. Chemie Int. Ed.* **2016**, *55*, 11382–11386.

[26] M. Korczynska, D. D. Le, N. Younger, E. Gregori-Puigjané, A. Tumber, T. Krojer, S. Velupillai, C. Gileadi, R. P. Nowak, E. Iwasa, S. B. Pollock, I. Ortiz Torres, U. Oppermann, B. K. Shoichet. D. G. Fujimori, *J. Med. Chem.* **2016**, *59*, 1580–1598.

[27] N. Igoe, E. D. Bayle, O. Fedorov, C. Tallant, P. Savitsky, C. Rogers, D. R. Owen, G. Deb, T. C. P. Somervaille, D. M. Andrews, N. Jones, A. Cheasty, H. Ryder, P. E. Brennan, S. Müller, S. Knapp, P. V. Fish, *J. Med. Chem.* **2017**, *60*, 668–680.

[31a] V. Bavetsias, R. M. Lanigan, G. F. Ruda, B. Atrash, M. G. McLaughlin, A. Tumber, N. Y. Mok, Y.-V. Le Bihan, S. Dempster, K. J. Boxall, F. Jeganathan, S. B. Hatch, P. Savitsky, S. Velupillai, T. Krojer, K. S. England, J. Sejberg, C. Thai, A. Donovan, A. Pal, G. Scozzafava, J. M. Bennett, A. Kawamura, C. Johansson, A. Szykowska, C. Gileadi, N. A. Burgess-Brown, F. von Delft, U. Oppermann, Z. Walters, J. Shipley, F. I. Raynaud, S. M. Westaway, R. K. Prinjha, O. Fedorov, R. Burke, C. J. Schofield, I. M. Westwood, C. Bountra, S. Müller, R. L. M. van Montfort, P. E. Brennan, J. Blagg, *J. Med. Chem.* **2016**, *59*, 1388–1409.

[31b] S. M. Westaway, A. G. S. Preston, M. D. Barker, F. Brown, J. A. Brown, M. Campbell, C. Chung, H. Diallo, C. Douault, G. Drewes, R. Eagle, L. Gordon, C. Haslam, T. G. Hayhow, P. G. Humphreys, G. Joberty, R. Katso, L. Kruidenier, M. Leveridge, J. Liddle, J. Mosley, M. Muelbaier, R. Randle, I. Rioja, A. Rueger, G. A. Seal, R. J. Sheppard, O. Singh, J. Taylor, P. Thomas, D. Thomson, D. M. Wilson, K. Lee, R. K. Prinjha, *J. Med. Chem.* **2016**, *59*, 1357–1369.

[32a] E. H. Demont, C. Chung, R. C. Furze, P. Grandi, A.-M. Michon, C. Wellaway, N. Barrett, A. M. Bridges, P. D. Craggs, H. Diallo, D. P. Dixon, C. Douault, A. J. Emmons, E. J. Jones, B. V. Karamshi, K. Locke, D. J. Mitchell, B. H. Mouzon, R. K. Prinjha, A. D. Roberts, R. J. Sheppard, R. J. Watson, P. Bamborough, *J. Med. Chem.* **2015**, *58*, 5649–5673.

[32b] G. Poncet-Montange, Y. Zhan, J. P. Bardenhagen, A. Petrocchi, E. Leo, X. Shi, G. R. Lee, P. G. Leonard, M. K. Geck Do, M. G. Cardozo, J. N. Andersen, W. S. Palmer, P. Jones, J. E. Ladbury, *Biochem. J.* **2015**, *466*.

[32c] P. Bamborough, C. Chung, E. H. Demont, R. C. Furze, A. J. Bannister, K. H. Che, H. Diallo, C. Douault, P. Grandi, T. Kouzarides, A.-M. Michon, D. J. Mitchell, R. K. Prinjha, C. Rau, S. Robson, R. J. Sheppard, R. Upton, R. J. Watson, *Angew. Chemie Int. Ed.* **2016**, *55*, 11382–11386.

[34] A. D. Morley, A. Pugliese, K. Birchall, J. Bower, P. Brennan, N. Brown, T. Chapman, M. Drysdale, I. H. Gilbert, S. Hoelder, A. Jordan, S. V. Ley, A. Merritt, D. Miller, M. E. Swarbrick, P. G. Wyatt, *Drug Discov. Today* **2013**, *18*, 1221–1227.
